# SynTemp: Efficient Extraction of Graph-Based Reaction Rules from Large-Scale Reaction Databases

**DOI:** 10.1021/acs.jcim.4c01795

**Published:** 2025-02-28

**Authors:** Tieu-Long Phan, Klaus Weinbauer, Marcos E. González Laffitte, Yingjie Pan, Daniel Merkle, Jakob L. Andersen, Rolf Fagerberg, Christoph Flamm, Peter F. Stadler

**Affiliations:** † Bioinformatics Group, Department of Computer Science &Interdisciplinary Center for Bioinformatics &School for Embedded and Composite Artificial Intelligence (SECAI), Leipzig University, Härtelstraße 16-18, D-04107 Leipzig, Germany; ‡ Department of Mathematics and Computer Science, University of Southern Denmark, DK-5230 Odense M, Denmark; § Machine Learning Research Unit, TU Wien Informatics, A-1040 Wien, Austria; ∥ Center for Scalable Data Analytics and Artificial Intelligence (ScaDS.AI), Leipzig University, D-04103 Leipzig, Germany; ⊥ Department of Theoretical Chemistry, University of Vienna, Währingerstraße 17, A-1090 Vienna, Austria; # Faculty of Technology, Bielefeld University, Postfach 10 01 31, D-33501 Bielefeld, Germany; ∇ Max Planck Institute for Mathematics in the Sciences, Inselstraße 22, D-04103 Leipzig, Germany; ° Facultad de Ciencias, Universidad National de Colombia, Bogotá CO-111321, Colombia; ◆ Center for non-coding RNA in Technology and Health, University of Copenhagen, Ridebanevej 9, DK-1870 Frederiksberg, Denmark; ¶ Santa Fe Institute, 1399 Hyde Park Rd., Santa Fe, New Mexico 87501, United States

## Abstract

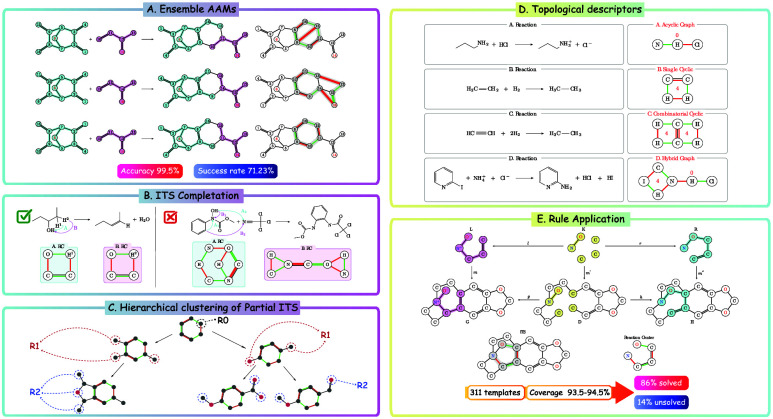

Reaction templates are graphs that represent the reaction center as well as the surrounding context in order to specify salient features of chemical reactions. They are subgraphs of *imaginary transition states*, which are equivalent to double pushout graph rewriting rules and thus can be applied directly to predict reaction outcomes at the structural formula level. We introduce here SynTemp, a framework designed to extract and hierarchically cluster reaction templates from large-scale reaction data repositories. Rule inference is implemented as a robust graph-theoretic approach, which first computes an atom–atom mapping (AAM) as a consensus over partial predictions from multiple state-of-the-art tools and then augments the raw AAM by mechanistically relevant hydrogen atoms and extracts the reactions center extended by relevant context. SynTemp achieves an exceptional accuracy of 99.5% and a success rate of 71.23% in obtaining AAMs on the *chemical reaction dataset*. Hierarchical clustering of the extended reaction centers based on topological features results in a library of 311 transformation rules explaining 86% of the reaction dataset.

## 1 Introduction

Recent advancements in Artificial Intelligence (AI) and data-driven methods have markedly accelerated the molecular design of drug candidates and materials. The development of recipes to construct a target compound from available starting materials by orchestrating a sequence of chemically viable reaction steps, i.e., *synthesis planning*, thus also is increasingly treated as a computational problem. Traditionally, it is most commonly approached by retrosynthetic analysis, formalized by E. J. Corey^1,2^ in the 1960s as the recursive decomposition of the product considering the reverse of synthetic reactions. This calls for the exploration of a vast array of potential chemical reactions that have not yet been observed for a specific set of reactants but are analogous to known chemical transformations. Modern synthesis planning therefore crucially relies on *reaction patterns* that summarize the accumulated knowledge about feasible chemical reactions. Here, we describe a computational framework, SynTemp, designed to extract such reaction patterns from large-scale repositories of chemical reactions and to make them available in the form of explicit transformation rules.

Significant data resources for chemical reactions include the United States Patent and Trademark Office (USPTO) database^3^ and the commercial platform Reaxys.^4^ In particular, the latter, with its collection of over 55 million manually curated reactions, has become a pivotal resource for implementing deep learning models in retrosynthesis.^5^ Efficient synthesis planning for novel compounds, by its very nature, requires reasoning by analogy. This can be greatly facilitated by a representation of reactions that provides transformation rules applicable to novel compounds and novel combinations of reactants. A very transparent, explicit model of chemical reactions is provided by graph grammars, implemented, e.g., in MØD.^6^ Here, molecules are encoded as graphs and reactions are defined by transformation rules that rewrite local patterns of chemical bonds.^7^ Specific transformation rules, e.g., for well-known *named reactions*, are not challenging to write manually; for instance, Synthia (or Chematica(8)) successfully applies this approach, in its complex synthesis planning utilizing a database of approximately 70,000 expert-encoded rules.^9,10^ This level of effort, however, is unlikely to scale to the entirety of present-day chemical knowledge^5^ and cannot keep pace with the continuing exponential increase in the number of reported reactions.^11^

Hence, automatic methods to mine comprehensive data repositories for transformation rules are urgently needed. Recent efforts in this direction include the study by Shuan Chen et al.,^12^ which utilizes a generalized-template-based graph neural network, and research by Lung-Yi Chen,^13^ focusing on extraction and curation of reaction templates. SynTemp aims to produce explicit, interpretable patterns. The graph-theoretical approach pursued here yields a representation that not only has an equivalent interpretation as a computationally operational reaction rule but also can form the basis for a mechanistic reaction classification.

Reaction rules, by definition, describe bond changes and thus establish a one-to-one mapping between the atoms of the reactants and the products in the reaction center. The inference of such rules, therefore, requires reaction data endowed with atom–atom maps (AAMs). Sizable repositories of reaction data, such as Reaxys(4) or the United States Patent and Trademark Office (USPTO) database,^3^ however, provide reaction data only as sets of reactant and product molecules without AAM information. AAM inference, therefore, is the crucial first step toward accurate reaction patterns.

Methods for computing AAMs can be divided into rule-based and machine learning-based (ML-based) approaches. Rule-based techniques, such as Automapper,^14^Indigo,^15^NameRXN,^16^RDTool,^17^ and a complex tool described by Jaworski,^18^ rely on combinatorial optimization criteria such as minimal chemical distance (MCD)^19^ or maximum-common subgraph (MCS).^20^ These rule-based solutions face challenges in practice. Most importantly, the optimization objectives of MCD and MCS only approximate the inference of the actual chemical mechanism. Therefore, even exact solutions of the corresponding combinatorial optimization problem may yield a chemically incorrect AAM.^18,21^ Computational cost, moreover, may become an issue since various variants of MCS are well-known NP-complete problems.^22^ As an alternative, several ML-based tools have become available in recent years, most prominently AMLGAM,^23^RXNMapper,^24^GraphormerMapper,^25^ and LocalMapper.^26^ These methods bypass the formulation of the task as a combinatorial optimization problem and avoid the computationally expensive subgraph matching process by utilizing data-driven methods. Despite significant progress, AAMs obtained from machine learning approaches are not perfect either, as demonstrated both by recent benchmarking efforts^21^ and the performance data provided in the publications that describe the individual tools.

The comparison of AAM predictions generated by different tools is not trivial. One practical issue is that each tool describes AAMs using its own atom numbering. It was only proved recently that the equivalence of AAMs could be reduced to isomorphism of certain auxiliary graphs,^27^ notably including the Condensed Graph of a Reaction (CGR),^28^ which was introduced much earlier under the name *Imaginary Transition State* (ITS) as a graph theoretical description of a chemical reaction.^29^ The ITS graph provides a faithful representation of a chemical reaction and thus contains the necessary information to identify the reaction center. More precisely, the reaction center corresponds to a subgraph of the ITS containing at least all atoms incident to bonds that change during the transition from reactants to products.^30^ These subgraphs of the ITS are equivalent to the Double Pushout (DPO) graph rewriting rules used by the MØD package.^6^ Reaction centers embedded in ITS graphs therefore provide a source of reaction rules that can be used directly in large-scale computational applications. A final technical challenge arises from the fact that the comparison of AAMs requires that they are completely specified.^27^ This is only possible if the reaction data entry is balanced, i.e., if all participating molecules are represented on both sides of the reaction. For the majority of the data in the major repositories, however, this is not the case. SynTemp therefore greatly profits from recent advances in reaction rebalancing.^31^

The SynTemp framework, introduced in this paper, utilizes the equivalence of ITS graphs and AAMs, as well as the fact that reaction patterns turn out to be equivalent to subgraphs of the ITS graph, as the basis for its chemical reaction modeling. The purpose of SynTemp is to extract a limited number of explicit reaction rules from a large collection of reaction data. In the following section, we describe in detail how this is achieved. Two key ingredients are (1) to consider a hierarchy of partial ITS graphs that contain the reaction centers as well as additional structural contexts of different sizes and (2) to leverage clustering to extract reaction patterns that are consistently observed in a large collection of reaction data.

Other methods for template extraction, such as RDChiral(32) which employs SMARTS strings, have been proposed. These methods, however, usually ignore critical components, in particular hydrogen atoms and byproducts. In contrast, SynTemp incorporates these elements and thus provides a more comprehensive and accurate framework for understanding and modeling reaction mechanisms. Moreover, SynTemp utilizes an explicit graph representation instead of operating on strings and thus completely avoids the well-known problems associated with such linear encodings.^33−35^

## 2 Theory and Methods

### 2.1 General Framework

The notation adopted in this study is derived from previous studies.^27,36^ In order to make the presentation easier to read, we relegate the full mathematical details to Supporting Section A.1 and restrict ourselves here to a high-level description of the framework.

**Figure 1:**
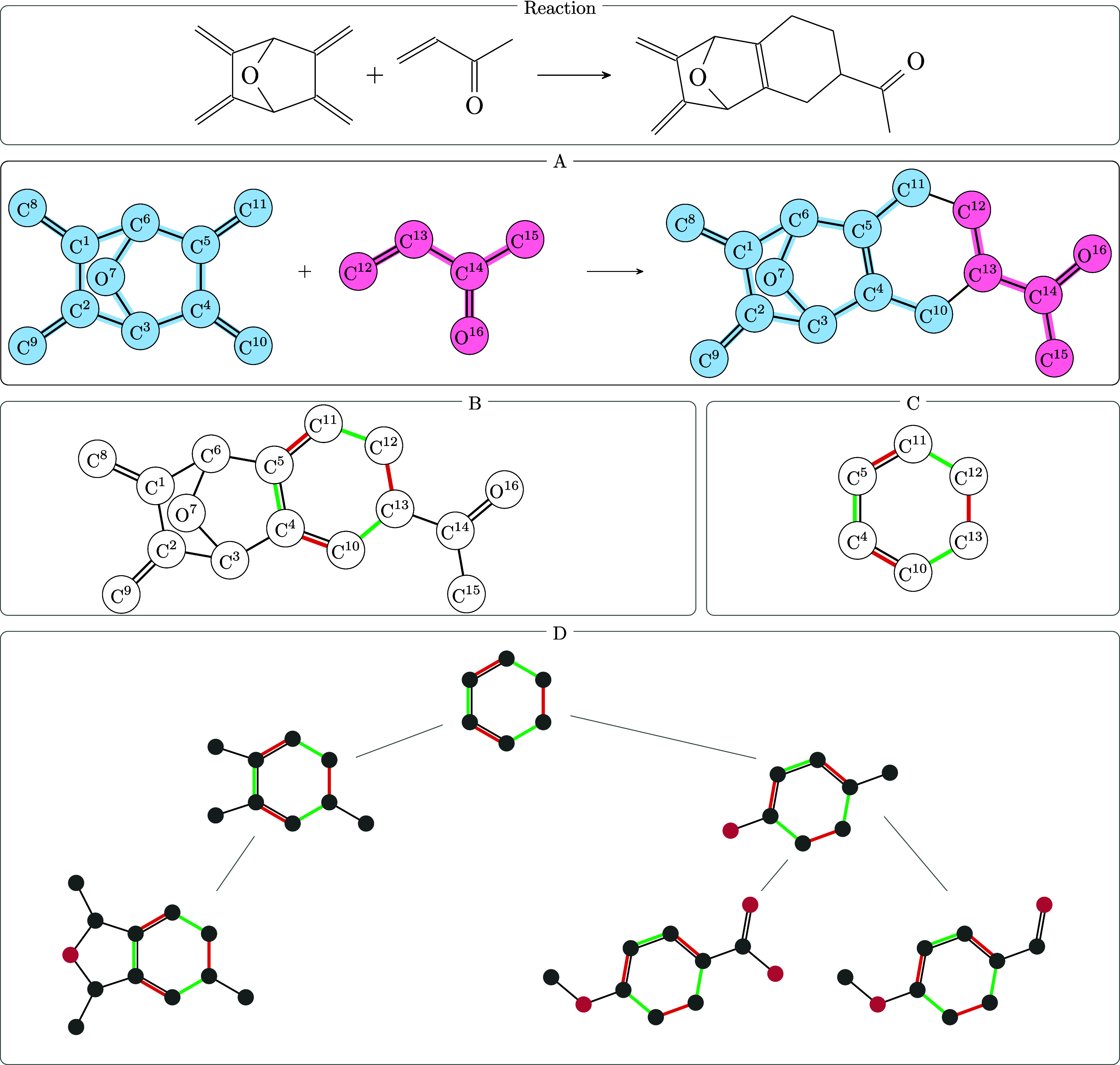
Procedure for extracting reaction rules in SynTemp. (A) Reaction with accurate atom–atom mapping between reactants and products. (B) Complete ITS graph. (C) Extracted reaction center. (D) Hierarchical clustering around the reaction center. Red edges signify breaking bonds; green edges show forming bonds.

SynTemp operates through four main stages, as outlined in Figure 1. The process initiates with a chemical reaction input in SMILES format, from which an AAM is inferred. Formally, we treat a chemical reaction as a map between two graphs *G* and *H* whose connected components are the reactant and product molecules, respectively. The AAM is simply an invertible map α : *V*(G) → *V*(*H*) between their vertex sets that preserves the atom types. A reaction, therefore, corresponds to a rearrangement of edges, i.e., of chemical bonds. As discussed in the introduction, no perfect solution exists for this task. We therefore leverage the agreement of multiple state-of-the-art AAM tools (RXNMapper, GraphormerMapper, LocalMapper, RDT) to determine trustworthy AAMs. Comparison of AAMs utilizes the theoretical framework developed in Section 2.3 as well as ensemble learning. Experimental procedures for the latter are described in more detail in Supporting Section A.2.

Once the AAM is defined, it dictates the construction of the ITS graph Υ(*G*,*H*,α), which combines the reactant and product structures. The vertices of *G* and *H* are identified in accordance with the bijection α and the edge set of the ITS graph contains the edges of both the reactant and product graphs *G* and *H*. Edge labels in the ITS graph distinguish between bonds that are modified or deleted from reactants, bonds introduced in products, or altered in bond type, and bonds that remain unchanged between *G* and *H*, see Figure 1B. An important practical complication is introduced by the fact that AAM tools typically do not map hydrogen atoms. However, the mechanisms of many important reactions involve the forming and breaking of bonds to hydrogen atoms. This situation can result in either a complete or partial ITS. In the case of a partial ITS, implicit hydrogens are inferred to accurately depict the reaction mechanism, as further elaborated on in Section 2.4.1.

The next phase involves identifying the reaction center within the ITS, consisting of the modified bonds and their adjacent atoms, and capturing the transformation core. However, this minimal reaction center alone is insufficient to describe all the preconditions necessary for the reaction. Thus, we extend this subgraph by adding additional vertices and bonds to obtain *extended reaction centers* as partial ITS graphs encoding the reaction rule, as discussed in Section 2.4.2.

Finally, these extended reaction centers are systematically classified into clusters using a hierarchical clustering approach. We utilize hierarchical clustering because it leads to a significant reduction of the computational cost associated with the iterative isomorphism checks required in graph clustering. By confining isomorphism checks to subgroups within the same reaction center, hierarchical clustering enhances the efficiency as the reaction center expands. For the details of this methodology, see Section 2.5. The resulting clusters form a library of template ITS graphs, which are converted into DPO rules in GML format for graph transformation systems such as MØD. The efficacy of these reaction rules is comprehensively evaluated in Section 2.6.

### 2.2 DPO Graph Rewriting and ITS Graphs

Chemical reactions can be modeled and studied through systems of rule-based rewriting of molecular graphs.^7,37,38^ These graph transformation systems require (1) rules that prescribe how a pattern in the input graph is to be changed within a larger substrate graph and (2) a definition of a pattern match. In DPO graph rewriting,^39^ both facets are expressed in terms of morphisms, i.e., maps, between graphs, providing a sound mathematical foundation in category theory. A rule is specified as a so-called span, , where *L*, *K*, and *R* are graphs and the arrows represent maps *l* : *V*(*K*) → *V*(*L*) and *r* : *V*(*K*) → *V*(*R*) which describe the embedding of *K* into *L* and *R* as a subgraph. The graph *L* describes the pattern in the reactants that is transformed into a product pattern *R*. The “context graph” *K* and the maps *l* and *r* establish the correspondence of vertices and edges that remain unchanged during the transformation from *L* to *R*. Since atoms do not change in the course of a chemical reaction, the maps *l* and *r* together define the AAM of the reaction. The edges of *E*(*K*) specify the chemical bonds that remain unchanged during the reaction, i.e., *xy* ∈ *E*(*K*) implies *l*(*xy*) ∈ *E*(*L*) and *r*(*xy*) ∈ *E*(*R*). From a chemical point of view, therefore, the DPO rule is a condensed representation of the reaction mechanism. It specifies which bonds change, and how, in the local vicinity of the reaction center. It is more general than the reaction center because the context graph *K* can also contain atoms and bonds that are not part of the reaction center.

**Figure 2:**
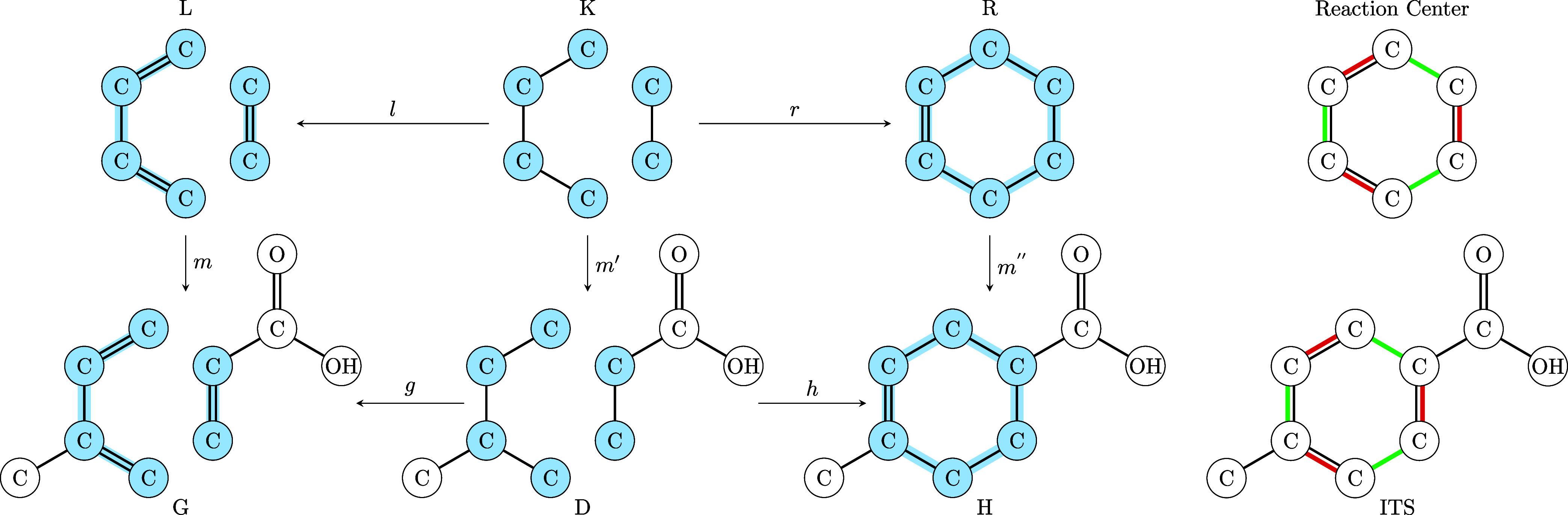
Illustration of the DPO graph rewriting technique. This diagram also shows the corresponding ITS and the reaction center of the DPO rule. *L*, *K*, *R* are the graphs, and *l* and *r* are the mappings, of the rule *p*. The maps *m* and *m*″ are bijections into the molecular graphs of the reaction *G* → *H. D* is the unchanged subgraph of the ITS, i.e., it contains the edges that are present in both *G* and *H*. It can be seen as the difference between the ITS and the reaction center.

Application of a rule  to a substrate *G* amounts to finding a position of the pattern *L* in the graph *G* and then replacing *L* by *R* at this position. This yields the graph *H* representing the reaction products. A graphical summary is presented in Figure 2. For a detailed mathematical explanation of the application of rules, we refer to the Supporting Section B.

### 2.3 Incomplete AAMs

**Figure 3:**
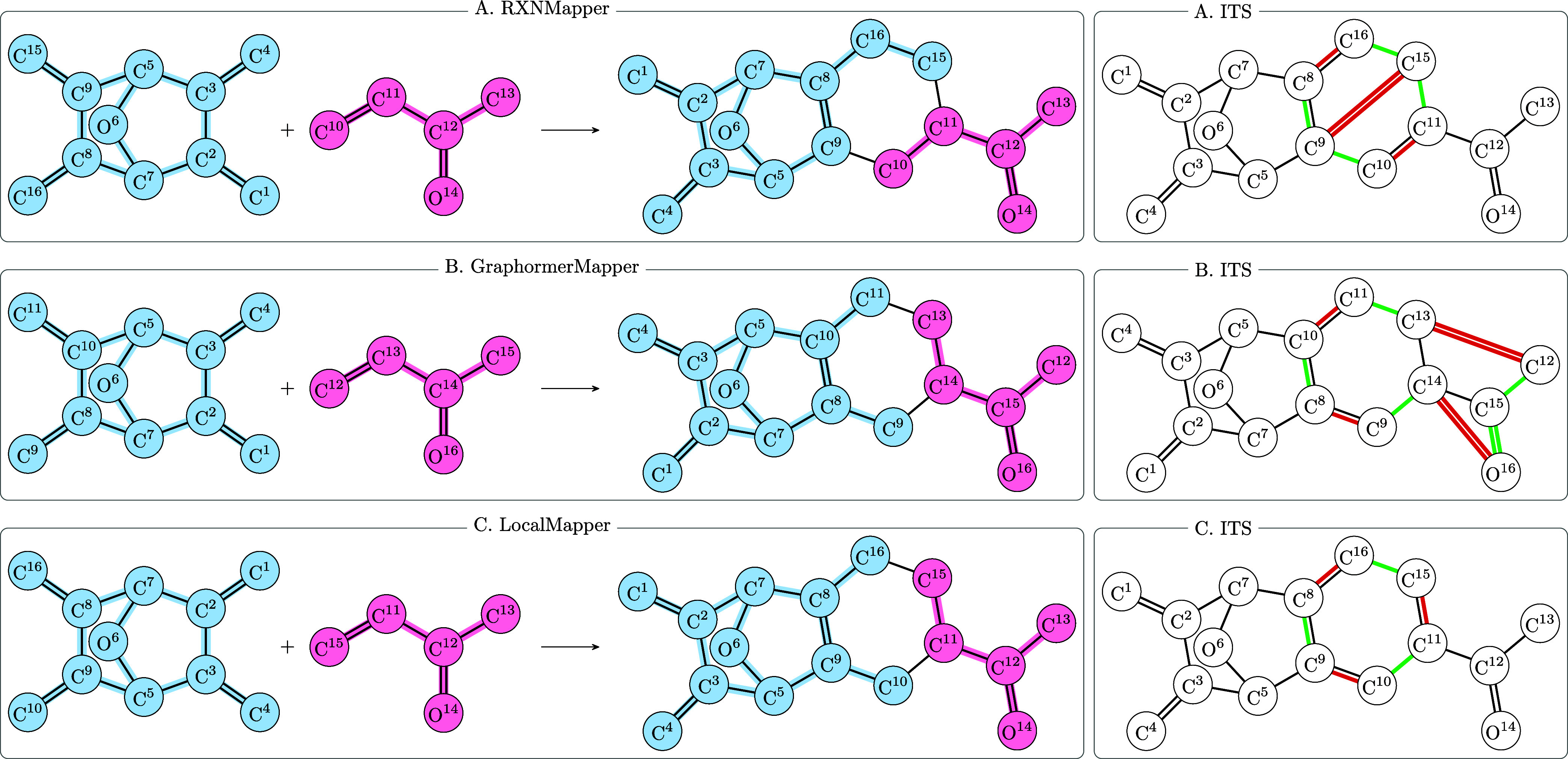
Differences in predicted AAMs and ITS graphs. (A) RXNMapper suggests a reaction center with two cycles of size four. (B) GraphormerMapper suggests a reaction center with two cycles of size three and six, respectively. (C) LocalMapper suggest a reaction center with a single cycle of size six.

The inference of reaction rules crucially depends on the accuracy of the ITS and thus on the underlying AAMs. While recent machine learning-based methods have been reported to yield improvement over older methods which solve combinatorial optimization problems, they are still subject to a non-negligible degree of uncertainty which may result in unequivalent AAMs and very different reaction centers. Figure 3 shows an example of a reaction for which the results of RXNMapper, GraphormerMapper, and LocalMapper are different.

The comparison of AAMs produced by two different tools is a nontrivial task. The reason is that each tool describes reactants and products in its own way. The same reaction thus appears as α : *V*(*G*) → *V*(*H*) for one tool and as β : *V*(*G*′) → *V*(*H*′) for another. For both, we can construct the ITS graphs Υ(*G*, *H*, α) and Υ(*G*′, *H*′, β), as well as the reaction center subgraphs Γ(*G*, *H*′, α) and Γ(*G*′, *H*′, β), as described in Section 2.4. The AAMs are equivalent if and only if the ITS graphs are isomorphic.^40^ In scenarios where reactions are balanced with complete atom–atom maps, the isomorphism between Υ(*G*, *H*, α) and Υ(*G*′, *H*′, β) can be evaluated, for example using the VF2 algorithm^41^ implemented in NetworkX, in order to determine their equivalence.

The situation is more complicated if a tool produces only a partial AAM, which in particular is the case for unbalanced reactions. A formal framework for partial AAMs has been described in our previous work.^36^ In general, the comparison of partial AAMs is a difficult problem. We therefore consider here only the special case that the AAMs cover the reaction center, i.e., that partial AAMs are “good” in the sense of Def. Six in our previous work.^36^ Some mathematical results that are directly relevant to our discussion are provided in Supporting Section A.2.1 since the complete formal statements require extensive notation borrowed from our previous work that we refrain from introducing in the main text for ease of presentation.

In cases of incomplete atom–atom mappings, the *sets* of atoms mapped by different tools will in general also be different and may vary substantially. This is in particular the case for tools like LocalMapper that focus primarily on the reaction center. The consistency of partial AAMs is formally defined in Def. Nine of our previous work.^36^ Here we will be content with a much simpler condition:

For two different reactions *G* → *H* and *G*′ → *H*′, the idea from^27,36^ can be used to compare *extended reaction centers*. We define these as subgraphs Ψ and Ψ′ of the ITS graphs Υ(*G*, *H*, α) and Υ(*G*′, *H*′, β) that contain the reaction centers Γ(*G*, *H*, α) and Γ(*G*′, *H*′, β) as well as all vertices in both reactant and product graphs within a distance at most *r* from a vertex in the reaction centers. If the two (complete) AAMs α and β are consistent, then Ψ and Ψ′ must be isomorphic (for proof of this statement, see Proposition 3 in Supporting Section A.2.1). It is important to note that the converse is *not* true, i.e., isomorphism of extended reaction centers does not imply consistency of the reaction maps. Counterexamples such as the one in Figure 9 of our previous work,^36^ however, turn out to be very rare for chemical reaction data. We therefore use Ψ ≅ Ψ′ as an efficiently testable condition. For the comparison of AAMs, we employed this *approximate condition*, as detailed in Supporting Section A.2.2. This condition for AAMs comparison facilitated the development of ensemble atom mappings.

### 2.4 Reaction Centers

Starting from a (partial) AAM α, a (partial) ITS is readily constructed in linear time. In practice, one can start with an edgeless graph comprising the vertices of *G* endowed with the double labels (*a*_*G*_(*x*), *a*_*H*_(α(*x*))) for all *x* ∈ *V*(*G*) and insert edges by iterating over the edge lists of *G* and *H*, at the same time recording the edge labels—see also.^27^ In the same manner, the reaction center Γ can be obtained directly from the (partial) AAM restricted to the reaction vertices, inserting reaction edges only.

#### 2.4.1 Completing the Reaction Center

Computational tools may return only partial AAMs, in particular ones which do not represent hydrogen atoms. While hydrogens can usually be suppressed safely in structural formula because simple valency rules imply the missing hydrogens, this is no longer true for subgraphs of the ITS. In particular, if only the reaction center extracted from a partial AAM is known, the reattachment of hydrogens cannot be unambiguously determined. As a consequence, without a complete representation of the reaction mechanism, deriving comprehensive mechanistic insights from “hydrogen-deficient” fragments of the ITS becomes challenging. Moreover, hydrogens that take part in the reaction must be present in reactant molecules, necessitating their inclusion in the reaction rules of a chemical rewriting system such as MØD. We therefore strive to extract the most information-rich representation of the ITS patterns.

Consider the example in Figure 4.1. Here, either hydrogen atom H^1^ or H^2^ is transferred to the hydroxy group. From a chemical point of view, hydrogen atoms H^1^ and H^2^ are equivalent, even though this equivalence is not readily apparent to computational methods tasked with inserting hydrogens. Such methods may yield two alternative reaction centers depicted in Figure 4.1A,B. In this case, these graphs are isomorphic, indicating that the reaction centers in A and B are interchangeable. The reaction in Figure 4.2 shows, however, that there is not always a unique hydrogen completion of the ITS. In this particular example, the resulting reaction center graphs, Figure 4.2A,B, are not isomorphic, highlighting the complexity and ambiguity inherent in achieving hydrogen completeness.

**Figure 4:**
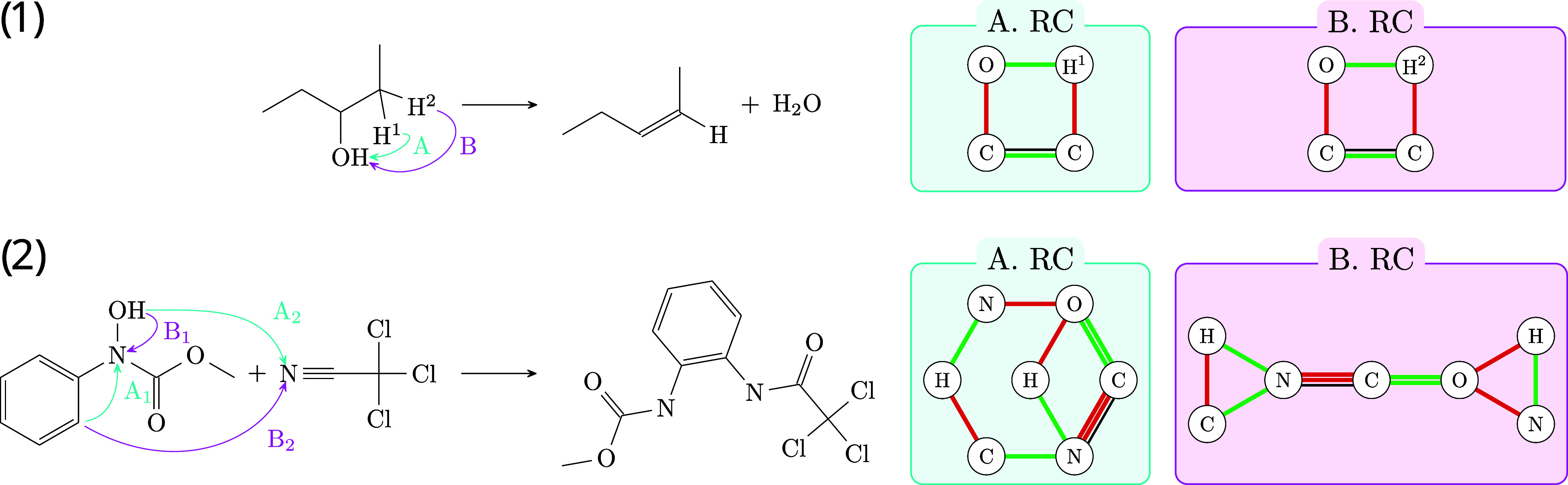
First row shows an unambiguous hydrogen insertion resulting in identical reaction center (RC) graphs (1A) and (1B). The second row illustrates an ambiguous hydrogen transfer in two scenarios with opposite transfer directions. In (2A), hydrogen moves from OH to CN and from a benzene ring to N–OH. Scenario (2B) demonstrates the reverse, with hydrogen transferring from the benzene ring to CN and from OH to N–OH. Instance (2A) is chemically accurate, leading to the formation of RC characterized by four- and six-membered cycles.

In practice, the precise mapping of hydrogen atoms may not always be possible. The insertion of hydrogen can thus be regarded as a special case within the extension of the reaction center, which is detailed in Section 2.4.2. Here, we augment the vertex labels by the attribute number_of_hydrogens in *G*, *H*, and Υ ≔Υ(*G*, *H*, α), and include atoms with a change in the number of attached hydrogens as part of the reaction center. If the changes in Υ involve a single pair of hydrogens, there exists a unique solution. However, if multiple pairs are involved, the situation escalates in complexity, yielding a number of combinations that grows exponentially with the number of pairs of hydrogens that undergo change. In cases where multiple potential solutions exist, we handle hydrogen reattachment by considering multiple AAMs in the subsequent analysis steps. If all AAMs α are identical, or if all extended ITS graphs Υ(*G*′, *H*′, α′) are isomorphic for all the possible extensions α′, *G*′, and *H*′ of, respectively, α, *G*, and *H*, due to addition of hydrogens, then we adopt a single representative as the complete ITS.

Hydrogen atoms may furthermore be problematic by originating from environmental sources such as solvents. Ideally, this would be explicit in the reaction data that we use as input. Usually, however, this is not the case. To manage this, we decompose reactions into sequences that account for “borrowed” environmental hydrogens. For a more detailed discussion, we refer to our recent study.^36^ In SynTemp, we initially exclude external hydrogen atom in the construction of the ITS database and add relevant hydrogen later for each mechanistic step.

#### 2.4.2 Extended Reaction Centers

Reaction mechanisms are oftentimes not only influenced by reaction vertices and edges but also by adjacent structural components such as functional groups that determine the local chemical environment. It is desirable, therefore, to consider patterns *L* in DPO rules that also include features that are not altered during the reaction. Formally this can be achieved by considering subgraphs *Q* of the ITS Υ(*G*, *H*, α) containing the reaction center as a proper subgraph, Γ ⊂ *Q* ⊆ Υ(*G*, *H*, α). The choice of the graph *Q* is the difficult issue here.

It stands to reason that the necessary context around the reaction center cannot be determined from a single reaction. Such information can be inferred by considering a set of reactions with the same reaction mechanism. A (mechanistically defined) reaction type or *named reaction*, such as the Diels–Alder reaction in Figure 1C above, however, is usually not annotated in most reaction data records. We therefore start from a (large) collection  of reactions and use a clustering approach to at least approximate a classification into mechanistically equivalent classes. Following ideas from Hendrickson’s seminal publication,^42^ we first subdivide  into classes determined by isomorphic reaction centers. That is, we set ρ′ ∼ ρ″ for two reactions  if and only if Γ(ρ′) ≅ Γ(ρ″).

The computation of the equivalence classes is straightforward: iterating over , one checks whether Γ(ρ) is isomorphic to a reaction center Γ′ previously obtained. If so, ρ is added to set . Otherwise, Γ(ρ) defines a new class and ρ is inserted into the new set . As a result, we obtain a collection of subsets  of  whose members share a common reaction center Γ. For a detailed mathematical explanation, we refer to Supporting Section A.4.

### 2.5 Clustering of Partial ITS graphs

**Figure 5:**
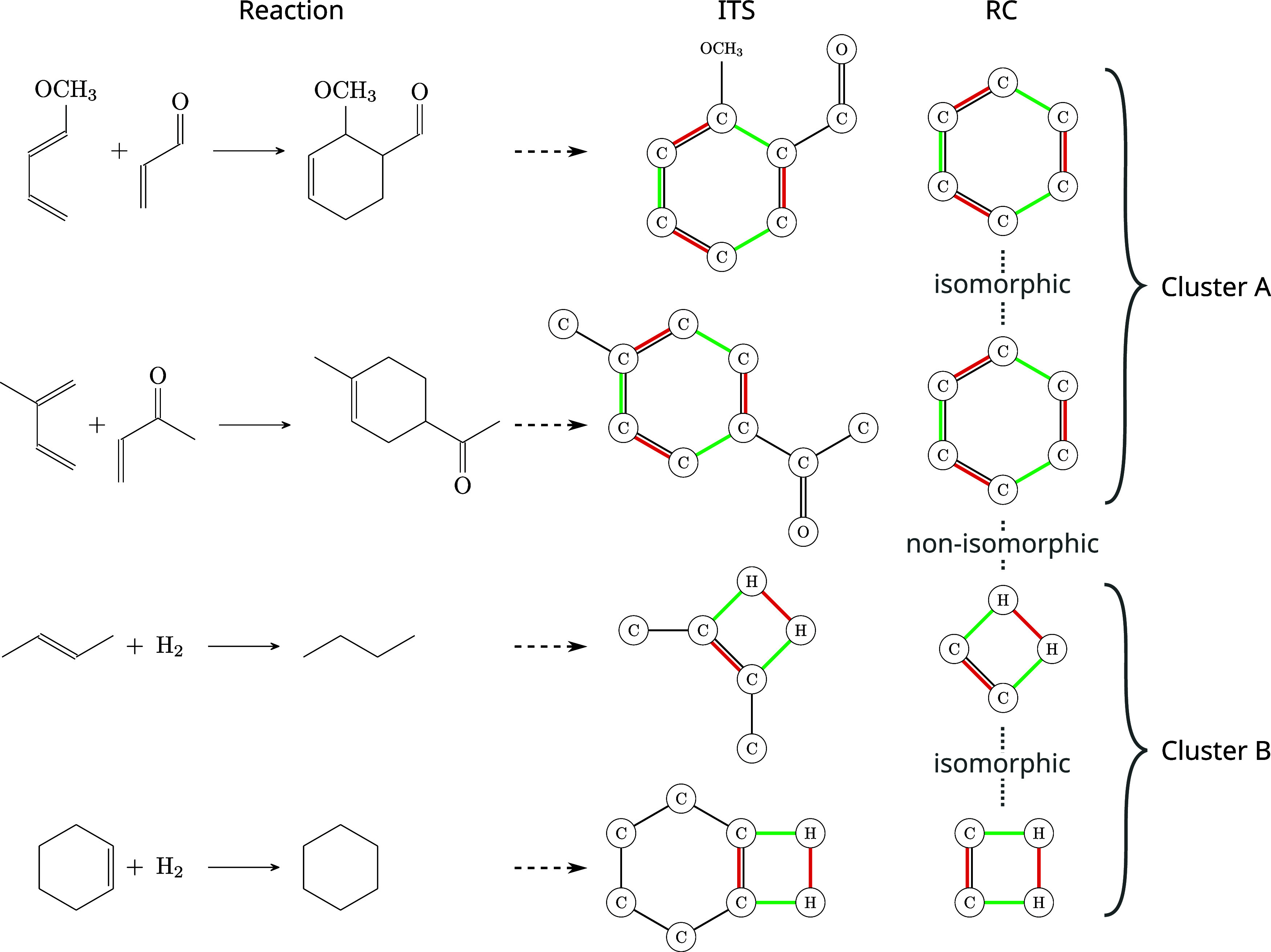
Process of rule extraction and clustering. SynTemp constructs high-confidence ITS graphs based on the congruence of multiple existing AAM tools for a given set of input reactions. Different reactions might have the same underlying mechanism and hence yield isomorphic reaction centers. Further, depending on the desired contexts, a reaction can yield multiple rules of various sizes but with isomorphic reaction centers. These rules can be clustered according to reaction center isomorphisms. The example shows the extracted ITS and reaction center (RC) of two different Diels–Alder reactions with an ortho and para product and of two hydrogenation reactions. The mechanisms in each pair are isomorphic and can be clustered. However, the Diels–Alder RC and the hydrogenation RC are nonisomorphic and belong to different clusters.

For a set of reactions , we compute the partial ITS graphs *Q*_*r*_^(*i*)^ for each reaction , where *r* = 0, 1, 2,... denotes the *expansion radius*, i.e., the maximal number of bonds that an atom in the graph *Q*_*r*_^(*i*)^ is away from the reaction center. Thus, Γ_*i*_ = *Q*_0_^(*i*)^ coincides with the reaction center. This collection of partial ITS graphs can be partitioned further into sets of unique reaction patterns by verifying isomorphisms (see Alg. S1). Our clustering method, which is illustrated in Figure 5, does not employ machine learning but categorizes reaction centers based on isomorphism. For larger values of *r*, however, this becomes computationally demanding for large data sets. We therefore make use of the fact that these partial ITS graphs of the extended reaction centers form a hierarchical structure, which is an immediate consequence of Cor. Six in Supporting Section B.

In order to construct this hierarchy , we add a formal root as a parent of the nonisomorphic reaction centers. For each reaction *p*, we check whether RC *Q*_0_^(*p*)^ is isomorphic to a child of the root. If so, we proceed to the children of the root and check for isomorphism *Q*_1_^(*p*)^, and so on. If the isomorphism tests fail, the pattern *Q*_*r*_^(*p*)^ is new and is inserted as a new child of the node in  for which the isomorphism test of its parent, i.e., *Q*_*r*–1_^(*p*)^, succeeded. Novel reaction centers are correspondingly inserted as children of the formal root. In practice, the insertion into  is interleaved with the construction of graphs *Q*_*r*_^(*p*)^. For details, we refer to Alg. S2, further elucidated by the illustrative example in Figure 1D. We show in Supporting Section B that the hierarchical approach substantially reduces computational cost.

### 2.6 Application of Reaction Rules

The final result of the SynTemp pipeline is a collection of partial ITS graphs which describe reaction rules at different resolutions depending on the extent to which the reaction centers have been expanded. We refer to these partial ITS graphs as *reaction templates*, while reserving the term *reaction rules* for encoding of these data as DPO rewriting rules.

The template graphs are transformed into DPO rules, encoded in GML format for direct use with MØD. In order to check the validity and usefulness of the extracted rules, we apply a rule to a set of reactants of a known reaction and check whether the application of the graph rewriting rule recovers the expected products. ITS graphs also implicitly encode the reverse of a reaction. To this end, it suffices to exchange the first and second entry in the tuple of edge labels. Making use of this symmetry, we also tested whether the application of the reverse rule to a set of product molecules could recover the reactants.

The performance of rule application is quantified as the *coverage*, defined as the fraction of reactions correctly recovered by applying a rule to a set of reactants. However, in many cases, templates also match with a set of reactants other than the one from the rule extracted. In these cases, we obtain predictions for novel reactions. We quantify this as the *novelty rate*, defined as the fraction of successful applications of the rule that results in a novel reaction.

### 2.7 Dataset

We compare AAM methods and ensemble learning techniques using two subsets. Their reaction type distributions are illustrated in Figure S5.

- *Chemical reaction data sets*: Golden (1785 reactions),^21^NatComm (491 reactions), and USPTO_3K (3000 reactions).^26^
- *Biochemical reaction data sets*: Recon3D (382 reactions)^23^ and EColi (273 reactions).

To enhance comparison, we assess *success rate*, which is the proportion of reactions achieving atom mapping, and *accuracy*, which compares generated atom maps against ground truth in the reported database.

We inferred partial ITS graphs from a subset of the USPTO_50K dataset, categorized into ten classes by Schneider et al.^43^ Following the approach of Coley et al.,^44^ we split the dataset in an 8:1:1 ratio, allocating 80% for reaction template extraction. With no learning in rule extraction, validation (10%) and test (10%) sets serve for direct SynTemp evaluation. We reduced unbalanced reactions by using SynRBL(31) to suggest missing compounds.

## 3 Computational Results

In this section, we report on our computational results. Our experiments can be summarized as follows. SynTemp processes reaction SMILES for ensemble atom mapping and ITS graph completion, including hydrogen inference. We verify SynTemp’s robustness through theoretical and computational analyses in Supporting Section A.2. Optimal AAM tools selection is based on comprehensive benchmarking in Section 3.1. Hierarchical clustering and reaction center extension generate *reaction templates*, which are analyzed using topological descriptors (Section 3.2). These templates are converted into DPO rules or *reaction rules* in GML format, with efficacy assessed through additional benchmarking (Section 3.3) across various radii.

All experiments were conducted using Python 3.11 on an Intel(R) Core(TM) i7–8700 CPU @ 3.20 GHz with 12 cores, running Fedora 37.

### 3.1 Ensemble Atom Mapping

**Figure 6:**
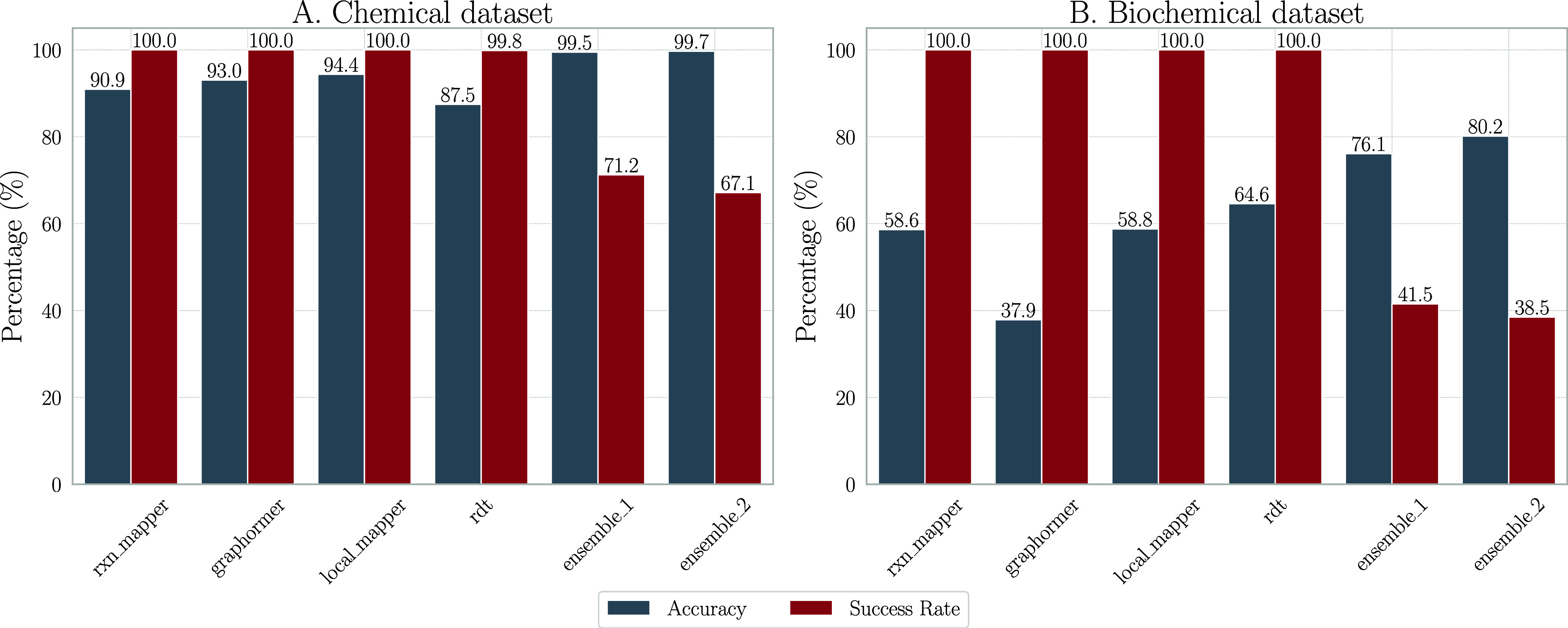
(A) and (B) present the benchmarking results for the chemical and biochemical data sets, respectively, using accuracy and success rate as evaluation metrics.

As evidenced in Supporting Section A.2.3, SynTemp exhibited superior robustness compared to CGRTools(28) in AAMs comparison. Notably, CGRTools employs the Condensed Graph of Reaction for comparing AAMs. We then utilized SynTemp to integrate state-of-the-art tools utilizing partial ITS graphs comparison to refine the accuracy and reliability of AAMs. This evaluation involved an assessment of four state-of-the-art tools (RXNMapper 0.3.0, GraphormerMapper 1.75, LocalMapper 0.1.4, and RDTool 2.4.1) alongside two ensemble strategies, applied across five distinct data sets. For technical details on the benchmarking procedure and the choice of tools, we refer to Supporting Section A.2. The results are summarized in Figure 6.

RXNMapper was the fastest tool (14 ms per reaction), followed by GraphormerMapper (78 ms per reaction) and LocalMapper (105 ms per reaction) while RDTool was the slowest (4.5 s) due to its extensive computations of maximum-common subgraphs between reactants and products for mapping. More detailed timing data are compiled in Table S2. To enhance accuracy, we utilized two ensemble strategies: *Ensemble_1* with RXNMapper, GraphormerMapper, and LocalMapper, and *Ensemble_2*, which extends *Ensemble_1* by including RDTool.

RDTool was the only tool that could not complete all reactions in the Golden and NatComm data sets, see Figure 6A,B. These results suggest that *Ensemble_2*, which includes RDTool, may not be efficient for processing large-scale databases. Additionally, the *Biochemical Reaction Dataset* posed more challenges compared to the *Chemical Reaction Dataset*, with processing times more than doubling.

Machine learning-based techniques were highly effective with the *Chemical Reaction Dataset*, achieving accuracies over 90%, see Figure 6A. Conversely, their performance was less impressive on the *Biochemical Reaction Dataset*, with accuracies falling below 60%, as shown in Figure 6B. Notably, RDTool outperforms the other machine learning-based tools on the *Biochemical Reaction Dataset*, attaining an accuracy of approximately 64.58%. This discrepancy may be attributed to the training focus of most machine learning tools on organic rather than biochemical reactions, which adversely affects their performance on the *Biochemical Reaction Dataset*. In both data sets, ensemble methods achieved the highest accuracies. In the *Chemical Reaction Dataset*, the difference in performance between *Ensemble_1* and *Ensemble_2* is marginal, at 99.47 and 99.69%, respectively. However, employing *Ensemble_2* decreased the success rate from 71.23 to 67.14% and increased computational expenses due to the integration of RDTool. Ensemble strategies were effective on the *Biochemical Reaction Dataset*, yielding accuracies of 76.10% for *Ensemble_1* and 80.16% for *Ensemble*_2, but the overall success rate was still below 45%, indicating that *Ensemble_1* is particularly beneficial for the *Chemical Reaction Dataset*.

Figure S7A compares the performance of the alternative AAM inference methods with respect to varying numbers of bond changes. As the number of bond changes increases, the accuracy of single AAM techniques diminishes. Notably, LocalMapper is most effective among single techniques for fewer bond changes (1–2), RXNMapper excels at medium bond changes (3–4), and RDTool dominates with higher bond changes (6–8). Conversely, ensemble techniques consistently maintain an accuracy above 90%, with a significant negative impact only when an AAM tool’s performance severely declines. Further analysis of cycle descriptors within the reaction center (Figure S7B) reveals that LocalMapper outperforms other single techniques at lower cycle counts (1), while GraphormerMapper surpasses others as the number of cycle descriptors increases. Ensemble techniques continue to outperform single options.

When analyzing the trade-off between success rate and accuracy in the Golden dataset, a standard benchmark, LocalMapper achieved an accuracy of 100% at a high confidence level, corresponding to a success rate of 53.3%. In contrast, RXNMapper reached an accuracy of 95.1% with a success rate of only 19.7%.^26^ Our ensemble strategies on the Golden dataset, *Ensemble_1* and *Ensemble_2*, attained accuracies of 99.47 and 99.69%, with success rates of 71.23 and 67.14%, respectively. The trade-off between success rate and accuracy was more favorable in *Ensemble_1* compared to *Ensemble_2*, LocalMapper, and RXNMapper.

At present, *Ensemble_1* constitutes the base choice for predicting AAMs with near-perfect accuracy. Moreover, we note that the methods provided by SynTemp for comparing AAMs yield an improvement in efficacy compared to CGRTools. Taken together, this allows SynTemp to process a large fraction of the available reaction bases with near-perfect accuracy.

### 3.2 Template Analysis

The initial dataset from which we extracted reaction patterns consisted of 40,012 reactions (see Section 2.7 for details on the dataset). About 86% (34,395) of reactions were successfully identified by consensus according to *Ensemble_1* and subsequent reinsertion of missing hydrogens in the reaction center. The hierarchical clustering of the partial ITS graphs with different expansion radii, as described in Supporting Section B.2, significantly reduced the computational efforts required for template extraction. It reduces the processing time for the entire data from approximately 1.5 h to 1.7 min for a maximal expansion radius of *r* = 3. In the end, we obtained 313 *raw templates* (*Q*_raw_) omitting hydrogen atoms, and 311 *complete templates* (*Q*_complete_) incorporating hydrogen atoms. These data are compiled in Table S3.

These reaction templates were then classified based on the topology of the reaction center, detailed in Table 1. The most relevant topological feature for our purposes is the cycle structure. We made use of the well-known fact that every graph can be decomposed into 2-connected components (where any two vertices are located on a common cycle), and a tree-like remainder that may in turn consist of several mutually disconnected parts. Each of the 2-connected components can be represented by a minimal cycle basis (MCB). Even though the MCB of a graph is not unique in general, it can be shown that all MCBs have the same number of cycles with the same length.^45,46^ Thus, we could characterize the reaction center by the list of cycle lengths of any of its MCBs, augmented by a zero for every tree-like component. To obtain a uniquely defined descriptor, we stipulated that the list of cycle lengths is sorted in ascending order.

**Table 1:** Classification of Reaction Templates

**descriptor**	**category**	**description**
reaction type	elementary (simple)	single-step reactions, involving acyclic or simple cyclic structures.
	non-elementary (complicated)	multistep reactions, involving combinatorial or complex cyclic structures.
topological type	acyclic graph	structures without cyclic elements.
	single cyclic	structures with a single cyclic component.
	combinatorial cyclic	structures with multiple cycles.
	hybrid graph	hybrid structures combining cyclic and acyclic elements.
cycle length	measures the minimal cycle basis in the reaction center graph, assigning a value of zero to acyclic centers.	

**Figure 7:**
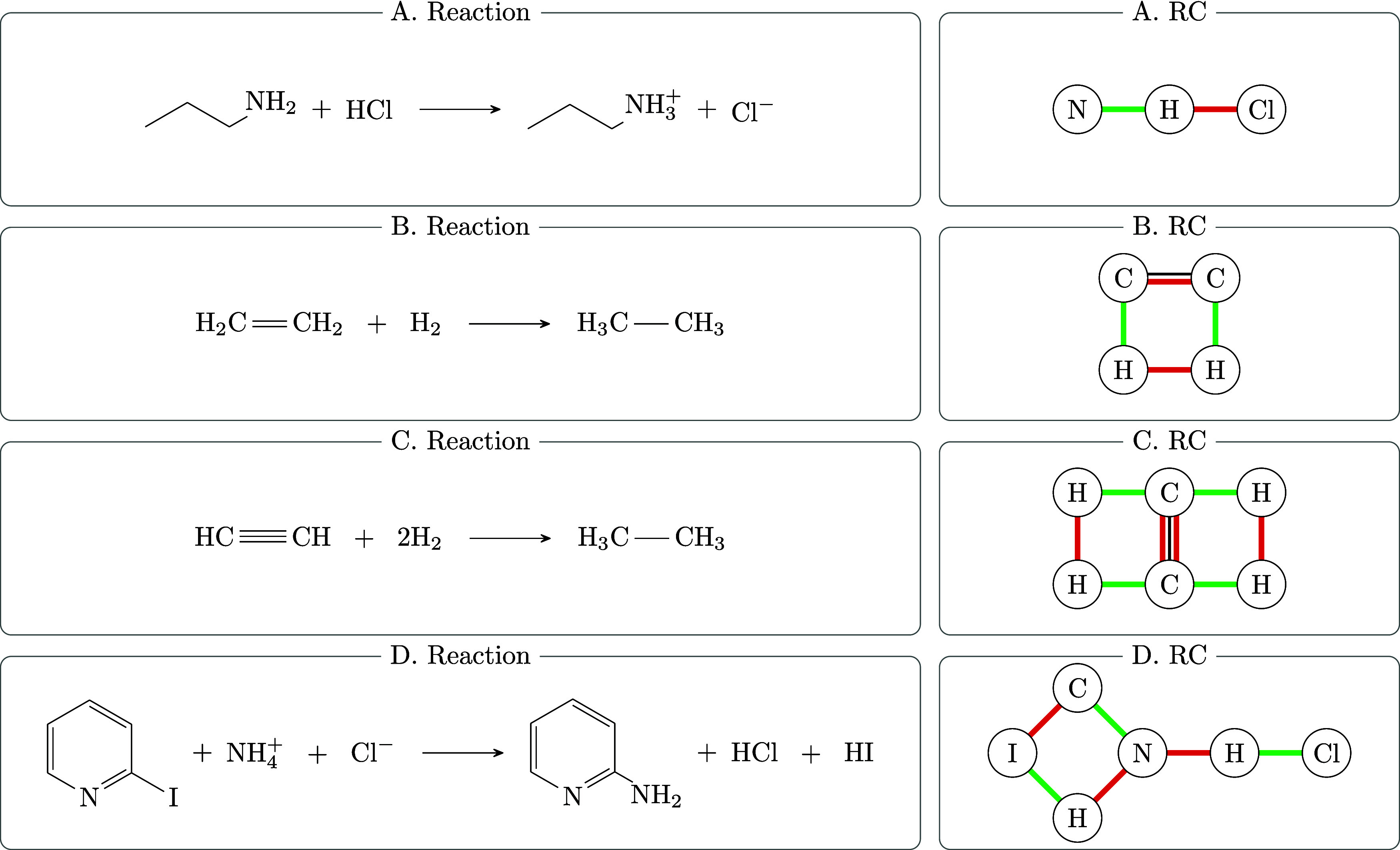
Examples of reaction patterns cataloged in the database, showcasing different topological structures with (A) *Acyclic Graph* [0], (B) *Single Cyclic* [4], (C) *Combinatorial Cyclic* [4,4], and (D) *Hybrid Graph* [0,4].

The reaction center in Figure 7A is *Acyclic*, consisting of a single tree, here just a path of length 2. The correponding descriptor is [0]. Figure 7B shows an *Elementary* reaction, classified as *Single Cyclic* in the terminology of Table 1. Since the reaction center consists of a single cycle of length 4, its descriptor is [4]. In Figure 7C, a *Complicated* reaction is presented, featuring a *Combinatorial Cyclic* topology. In this example, there is a unique MCB comprising two 4-cycles, corresponding to the descriptor [4,4]. Finally, Figure 7D depicts a *Complicated* reaction with a *Hybrid Graph* topology, which integrates acyclic and cyclic elements with cycle lengths of [0,4].

**Figure 8:**
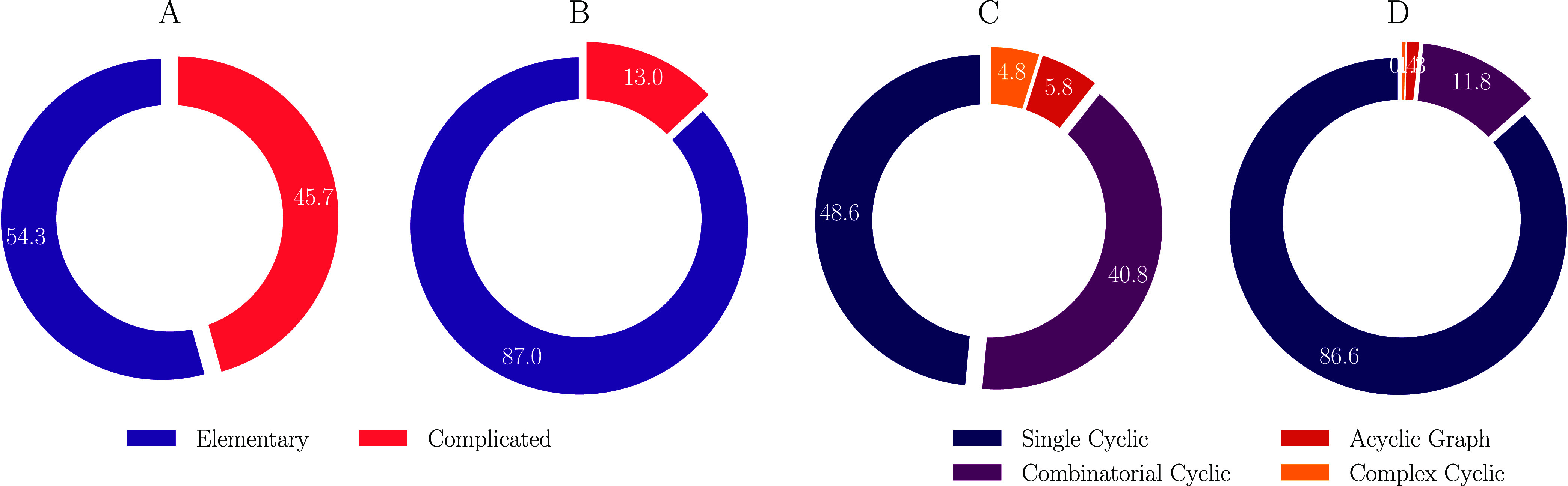
Pie charts display analysis of reaction types (Panels A and B), topological types (panels C and D), for the ”database” and the ”template library”, respectively.

We conducted an analysis of these descriptors, evaluating them both within the context of the entire database consisting of 34,395 reactions for which we identified the reaction centers (hereafter referred to as the “database”), and our template library, which comprised 311 distinct templates. The overwhelming majority of the database, 87.0%, was categorized as elementary (and thus single-step) reactions, see Figure 8A. This percentage decreased to 54.3% of the 311 templates collected in the template library, Figure 8B. More detailed statistics can be found in Table S4.

Further analysis of the topological configurations revealed that the *Single Cyclic* type was by far the most abundant reaction type, comprising 86.6% of the reaction centers across the “database” as depicted in Figure 8C. This dominance persisted in the template library, where *Single Cyclic* remained the most common configuration, accounting for 48.6%.

**Figure 9:**
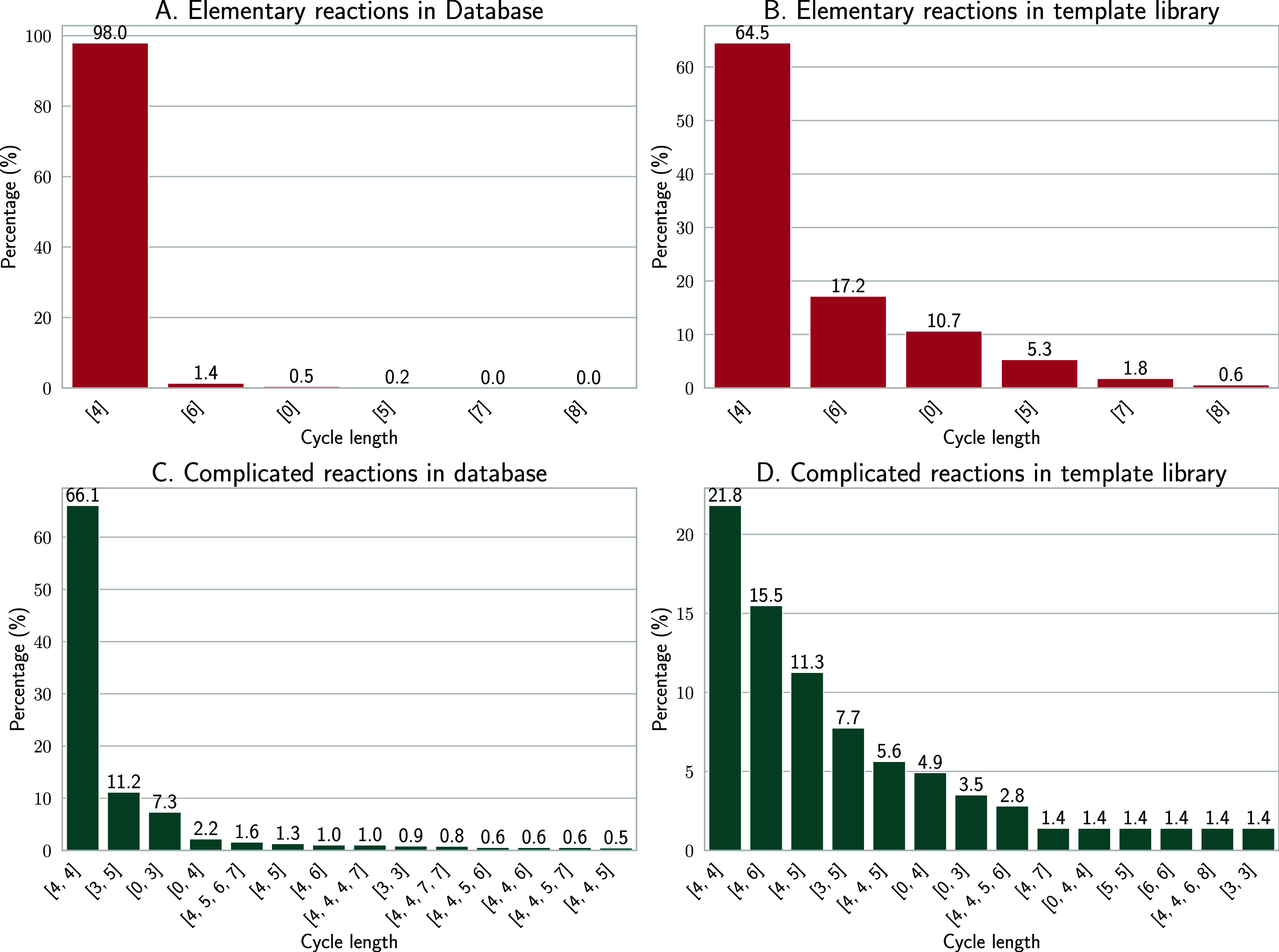
Cycle length analysis for *Elementary* reactions (panels A and B) and *Complicated* reactions (panels C and D).

We further analyzed the cycle length in the reaction center of both elementary and complex reactions, as shown in Figure 9 and summarized in Table S5. In the subset of elementary reactions, comprising both *Acyclic Graphs* with a single connected component and *Single Cyclic* types, we found that more than 98% of the entire “database” was of type [4], i.e., the reaction center was formed by a single cycle of length 4. Six-membered cycles, i.e., type [6], account for 1.4% of the reactions. Similar patterns were observed in our “template library”, with four-membered cycles at 64.5% and six-membered cycles at 17.2%, see Figure 9B. Four-membered cycles are typically associated with transition states in 1,2-addition reactions or nonconjugated reactions, whereas six-membered cycles are common in 1,4-addition reactions, where conjugated effects play a significant role.

For more intricate templates, such as *Combinatorial Cyclic* and *Hybrid Graph*, similar patterns were observed, as shown in Figure 9C,D. Notably, more than 66.1% of these feature combinations involve two four-membered cycles in the “database”, making it the most prevalent structural configuration. In the template library, this arrangement is similarly dominant, accounting for 21.8% of all templates. This is followed by condensed systems of four- and six-membered cycles, which constitute 11.2% of the structures in the “database” and 15.5% in the “template library”, respectively.

Analyzing the cycle lengths of the reaction center could provide insights into the reaction steps involved. To this end, we took the number of entries in the list of cycle length as an estimate for the number of steps. We verified on a subset of 100 reactions that this estimator is plausible from a chemical point of view. See Supporting File 2. In the database, 87.0% of reactions were single-step, similar to the proportion of elementary reactions. Only 10.98% were two-step, and less than 2% were more than two-step. The “template library” showed a slightly different from “database”, with 54.34% single-step, 33.12% two-step, and less than 13% more than two-step reactions. More detailed statistics can be found in Table S4.

A more detailed inspection of the 311 *Q*_complete_ entries in the library of reaction patterns revealed that a subset of 20 rules disproportionately accounts for 80% of all cataloged reactions, as depicted in Figure S8A. The two most prominent rules are *amide formation* (*Rule 8*) and *amine alkylation* (*Rule 2*), as illustrated in Figure S8B and S8C, respectively. Detailed descriptions of these 20 rules are provided in Table S6. The entire library of rules is available in the Supporting File 3.

The processing of 40,012 reactions, including ITS graph extraction, clustering, and conversion to DPO rules, required approximately 23 min on a system equipped with an Intel(R) Core(TM) i7–8700 CPU @ 3.20 GHz, featuring 12 CPU and 96 GB of RAM, averaging 34 ms per reaction. While this rate is efficient for medium-sized data sets, scaling up to millions of reactions poses significant computational challenges.

### 3.3 Application of Reaction Rules and Benchmarking Study

**Figure 10:**
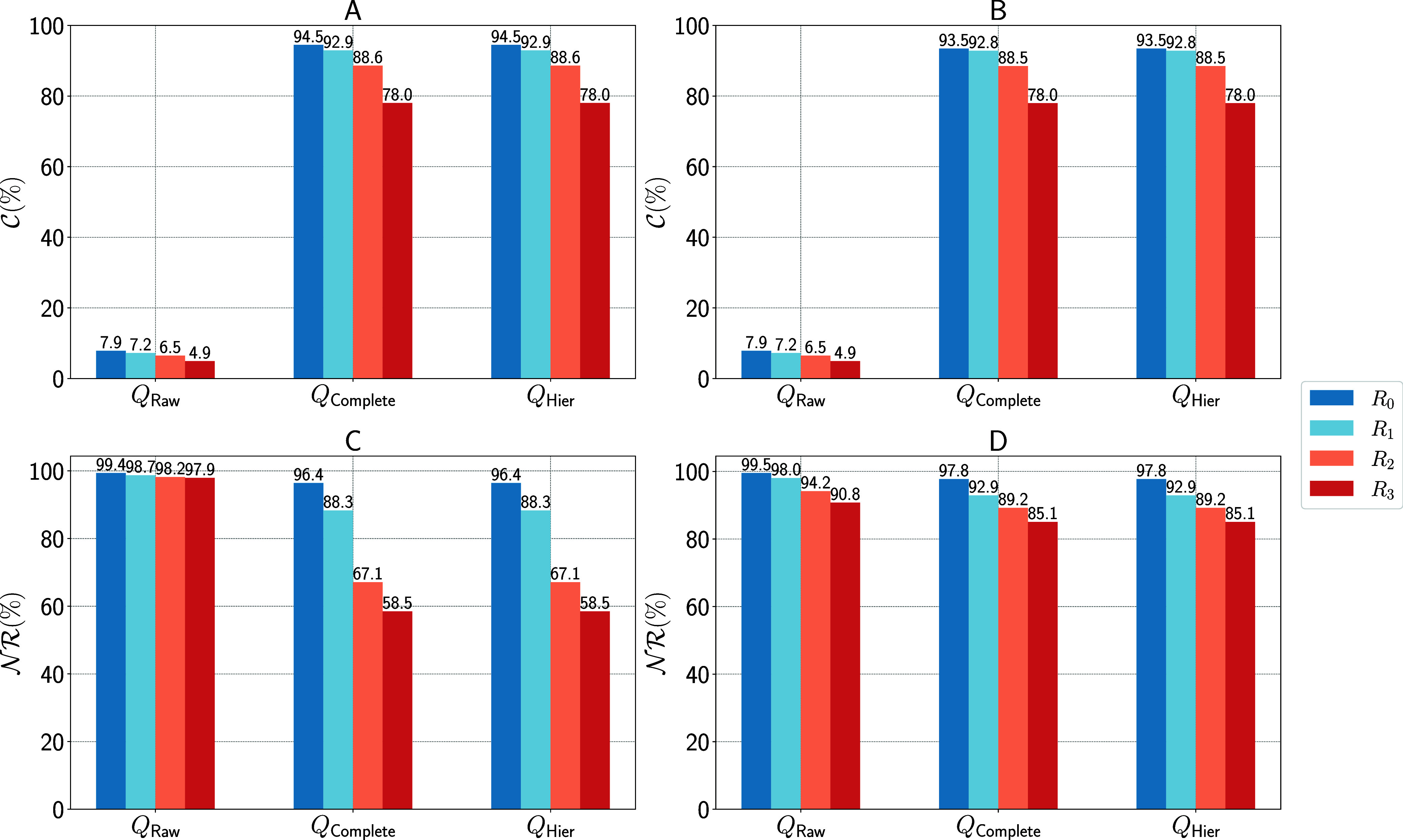
Performance of rule applications on the validation set of three different template types (*Q*_raw_, *Q*_complete_, *Q*_hier_) across varying radii. Panels (A) and (B) depict the  for forward and backward predictions, respectively. Panels (C) and (D) illustrate the  for forward and backward predictions.

In this section, we executed a series of rule application experiments across varying radii of templates to analyze the quality of the *reaction templates/rules* based on two metrics: *novelty rate* () and *coverage* (). We observed a distinct inverse relationship between the radius of templates and these two metrics. This relationship is demonstrated in Figure 10 (validation set) and Figure S9 (test set). An increase in the radius leads to the expansion of template rules, significantly reducing the likelihood of subgraph morphisms with input molecules, as reported in Table S3. Consequently, this results in a lower number of generated solutions, illustrating a practical trade-off between  and . While a decrease in  can simplify the search for optimal solutions, it may concurrently diminish  and thereby restrict potential exploratory pathways within chemical reaction networks.

As shown in Figure 10, the performance of *Q*_raw_ is notably inferior, evidenced by the high  and a  below 10% across all tested radii. This is a consequence of ignoring bond changes that involve hydrogens. Since *Q*_raw_ does not capture them, the corresponding rewriting rule is not sufficient to describe the transformation completely, i.e., the application of the rule to the reactants does not lead to product molecule(s) but to graphs that leave hydrogens attached to their original neighbors in the reactants and thus may result in an abnormal increase in valence bonds for certain atoms. We conclude, therefore, that most reactions involve hydrogens, and thus *Q*_raw_ rules, in general, are not applicable to complete representations of the molecules. In contrast, other template types significantly improve effectiveness, with  ranging from 78 to 94.5%.

An interesting finding was the relative consistency in  observed for both forward and backward predictions, as evidenced in Figure 10A,B, where the values exhibit only slight variations. In contrast, the  showed a marked disparity between forward and backward predictions, as depicted in Figure 10C,D, respectively. This significant difference could be attributed to the generally smaller number of molecules involved in backward predictions compared to forward predictions, simplifying the subgraph isomorphism and potentially leading to a higher number of solutions. This observation is further elaborated upon in Tables S7 and S8 for validation and test set, respectively. These findings highlight the complexities inherent in backward prediction tasks, making them notably more challenging than their forward counterparts.

The computational cost for rule application increases with expansion radius *r* used in the templates *Q*_*r*_. As illustrated in Figure S10, the progression *R*_0_ to *R*_1_ of *Q*_complete_ incurs a 2.5-fold increase in processing time. This increase becomes more pronounced, surging to a 20-fold rise at *R*_2_ and escalating further to a 50-fold increase at *R*_3_. Such exponential growth in computational demand underscores the need for more efficient processing strategies. In response, we have implemented a hierarchical rule application, denoted as *Q*_hier_, making use of the hierarchical structure of the templates. The processing time for *Q*_hier_ is significantly lower than that of *Q*_complete_, with only a 60% increase in processing time from *R*_0_ to *R*_3_ compared to the 50-fold increase observed with *Q*_complete_. Despite these differences in processing time, both approaches maintain consistent *coverage* and *novelty* across all choices of the extension radius *r*, see Figure 10A,B. Consequently, *Q*_hier_ emerges as the more efficient choice, enabling equal outcomes with reduced resource expenditure.

We finally compared the graph-theoretical approach described here with SMARTS-based templates, which are typically used in machine learning-driven retrosynthetic models.^47−49^ To extract reaction SMARTS from the training dataset covering 80% of the USPTO_50K we employed the RDChiral toolkit.^32^ We obtained 11,647 templates that were successfully applied to 93.3% of the entries in the 10% test set. In a comparative analysis, while SynTemp extracted only 311 reaction rules from the same dataset, it achieved remarkable *coverage* of 94.5% for forward prediction and 93.5% for backward prediction. The reactions that were not covered by our rules can be attributed to the presence of 194 ambiguous hydrogen atoms and 5423 nonequivalent AAMs, which together constitute 14.04% of the training set.

**Figure 11:**
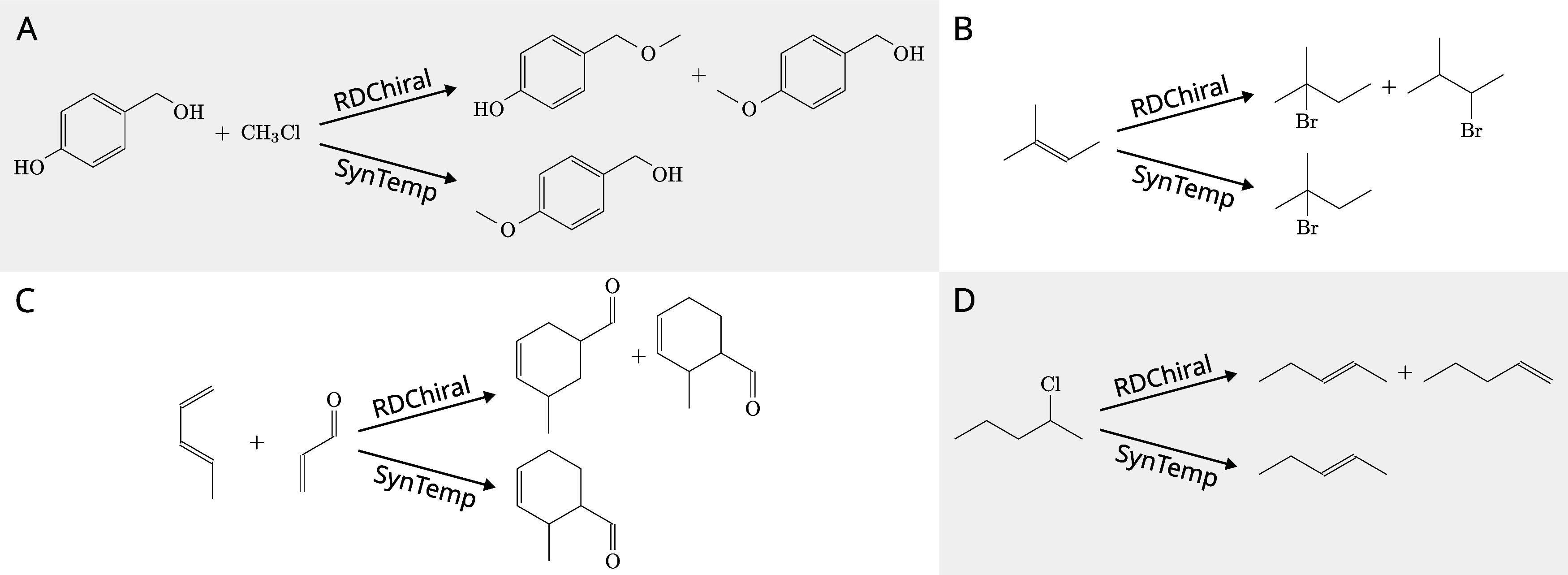
Comparison of template application differences between the standard RDChiral implementation and SynTemp across various cases: (A) Etherification; (B) Hydrobromide addition; (C) Asymmetric Diels–Alder reaction; (D) Elimination reaction.

The application of coarse-grained rules or templates in Figure 11 using RDChiral leads to two potential products. Extending the context graphs, i.e., using the hierarchy of templates in SynTemp, successfully eliminates the less viable synthesis route. This refined approach holds promise in synthesis planning, in particular, if the context is effectively and precisely expanded beyond the current arbitrary extensions.

About one-eighth of the USPTO reaction data are composite reactions. It is of interest, therefore, to investigate whether their ITS graphs can be derived as the consecutive application of two or more single-step reactions. To this end, we used the 169 single-step rules from *Q*_complete_ and combined them into more than 16,000 two-step rules. Somewhat surprisingly, only three of the rules in the template library are composites of rules for single-step reactions observed in the same data. These three cases are shown in Figure S11. We suspect that the direct rule composition fails because (a) the composite reactions take place in different parts of the molecule, and hence there is little or no overlap of the reaction centers of the individual steps, and (b) the templates used for the consecutive steps may involve inconsistent extended contexts. Given a library of patterns for single-step reactions, it is also possible to ask whether a given reaction can be explained by a sequence of single-step reactions.^50^ In contrast to rule composition, this approach does not require a substantial overlap of the reaction centers of consecutive rules.

## 4 Discussion and Conclusions

This contribution presents SynTemp, a framework for automatic reaction template/rule extraction. SynTemp supplies reaction rules as ITS subgraphs, equivalent to DPO graph rewriting rules, for immediate application to new substrates via MØD. The graphs contain the reaction center as a subgraph and thus describe a classification of reaction that refines e.g., the classification proposed in ref (42). SynTemp successfully tackles key technical problems associated with the inference of interpretable reaction patterns, namely the large-scale computation of reliable AAMs, the comparison of AAMs based on information, and the efficient clustering of pattern graphs with varying detail levels beyond the reaction center.

The present implementation uses an approximation for AAMs comparison on partial ITS graphs that delivers accuracies well above 90% on benchmark sets, but may occasionally return false positives. Computationally inferred AAMs form the basis of our approach to extract reaction templates. To this end, we integrated multiple state-of-the-art AAM tools to enhance accuracy. Specifically, we combined RXNMapper, GraphormerMapper, and LocalMapper, achieving an accuracy of 99.47% with a success rate of 71.23% on the *Chemical Reaction Dataset*. The ensemble approach of extracted templates to atom–atom mapping exhibits high confidence in correctly representing the underlying chemical mechanisms.

In the absence of extensive collections of reaction rules against which our results could be compared, we opted to maximize accuracy and confidence in the rule sets at the expense of *coverage*. We ignore nonequivalent AAMs and ambiguous hydrogens, which account for approximately 14% of the database. The *coverage* results in rule application within radii zero being limited to 93.5–94.5%, which is only slightly better than the 93.3% *coverage* achieved by RDChiral using SMARTS templates. However, this is achieved by a library of reaction patterns that, at present, contains only 311 entries, compared to more than 10,000 SMARTS, indicating a drastic difference in generalization ability.

In the present implementation, the context around the reaction center is simply defined by an expansion radius, i.e., it includes all atoms within a given graph-theoretical distance. Refining this context part of the rules remains an interesting research question. One promising approach is graph alignments, for which methods have recently become available.^40^ These could be used to distinguish common from variable parts of the context.

SynTemp can efficiently process 50,000 reactions within minutes on personal hardware. The effectiveness of SynTemp – and all similar tools–heavily depends on the accuracy and efficiency of the AAM inference process. The performance is therefore tied to the quality of the reaction data that are used as input. Unbalanced reactions in general compromise the reliability of results and complicate the inference process. The success rate of SynRBL in imputing missing compounds in the Reaxys database is approximately 76%.^31^ Reaction patterns inferred by SynTemp may contribute to future improvements of tools for correcting reaction data since they can help improve the coverage of reaction types in machine learning classifiers^51,52^ and improve the voting process among AAM tools with new methods such as the cycle descriptor for classifying reaction center topologies.

Given the prevalence of multistep reactions in the dataset, SynTemp leverages MØD’s rule composition features to explain these reactions as constituting single-step components.^50,53^ Reactions involving fewer steps are generally more chemically plausible, suggesting lower energy barriers and streamlined processes. This adds credibility to combinatorial AAM mapping approaches that seek reaction centers composed of few cycles, as advocated in the study of Mann et al.^54^ Nevertheless, there is no guarantee that the true mechanism minimizes descriptor lengths. Complex mechanisms may also form bonds that are broken again in subsequent steps and thus do not appear at all in the ITS and reaction center graphs. To address this issue at least in part, overlay graphs that extend the ITS have been introduced by.^55^ These graphs detail both the imaginary transition states and transient bonds within multistep reactions. Integrating this with recent insights into partial ITS graphs^36^ could significantly advance both our understanding and computational capabilities of multistep reaction mechanisms. Notably, a formal framework for rule composition is absent in SMARTS, highlighting the benefits of explicit graph transformations for modeling reactions. Finally, the correct and unambiguous completion of hydrogens in the ITS graphs, as a special issue in the context of AAM inference, also remains a topic for future improvements.

## Data Availability

The data sets supporting the conclusions of this article are available in the SynTemp repository: https://github.com/TieuLongPhan/SynTemp/tree/main/Data. The source code is available at: https://github.com/TieuLongPhan/SynTemp.

## Supporting Information Available

Contains detailed methodologies, mathematical explanations, and additional tables and figures that support the manuscript (PDF)
Detail of 100 reactions with reaction steps and cycle length (PDF)
Rules library extracted from the training set using the SynTemp package (PDF)
