# Supplementary material for: SynTemp: Efficient Extraction of Graph-Based Reaction Rules from Large-Scale Reaction Databases
Source: J Chem Inf Model. 2025 Feb 28;65(6):2882–96. doi: 10.1021/acs.jcim.4c01795 (PMC11938280; doi:10.1021/acs.jcim.4c01795)
Supplement: Supplementary file 1 — ci4c01795_si_001.pdf [file ci4c01795_si_001.pdf]

Supporting Information

SynTemp: Efficient Extraction of Graph-Based Reaction Rules from Large-Scale Reaction Databases

Tieu-Long Phan,[*,†,‡] Klaus Weinbauer,[†,¶] Marcos E. González Laffitte,[†,§] Yingjie Pan,[‖,‡] Daniel Merkle,[⊥,‡] Jakob L. Andersen,[‡] Rolf Fagerberg,[‡] Christoph Flamm,[‖] and Peter F. Stadler[†,#,‖,@,△,∇]

†*Bioinformatics Group, Department of Computer Science & Interdisciplinary Center for Bioinformatics & School for Embedded and Composite Artificial Intelligence (SECAI), Leipzig University, Härtelstraße 16–18, D-04107 Leipzig, Germany*

‡*Department of Mathematics and Computer Science, University of Southern Denmark, DK-5230 Odense M, Denmark*

¶*Machine Learning Research Unit, TU Wien Informatics, A-1040 Wien, Austria*

§*Center for Scalable Data Analytics and Artificial Intelligence (ScaDS.AI), Leipzig University, D-04103, Leipzig, Germany*

‖*Department of Theoretical Chemistry, University of Vienna, Währingerstraße 17, A-1090, Vienna, Austria*

⊥*Faculty of Technology, Bielefeld University, Postfach 10 01 31, D-33501, Bielefeld, Germany*

#*Max Planck Institute for Mathematics in the Sciences, Inselstraße 22, D-04103, Leipzig,*

Germany

@Facultad de Ciencias, Universidad National de Colombia,Bogotá, Colombia

△Center for non-coding RNA in Technology and Health, University of Copenhagen, Ridebanevej 9, DK-1870, Frederiksberg, Denmark

∇Santa Fe Institute, 1399 Hyde Park Rd., Santa Fe, NM, 87501, USA

E-mail: tieu@bioinf.uni-leipzig.de

A Mathematical Results

A.1 Notation and Preliminaries

Molecules and Reactions. The notation adopted in this section is based on two previous publications.[1,2] A chemical reaction $G \longrightarrow H$ is represented by a pair of labeled graphs G and H, where the reactant and product molecules are the connected components of G and H, respectively. Throughout, we consider labeled graphs without multiple edges. Given a graph G, we write $V(G)$ and $E(G)$ for the vertex and edge sets of G. Formally, vertex and edge labels are treated as functions $a_G : V(G) \to L_v$ and $b_G : E(G) \to L_e$ from the vertices and edges, respectively, into suitable non-empty label sets L_v and L_e. Vertex labels designate atom types and edge labels designate bond types. We admit optional loops with labels that annotate information about the electronic state of an atom such as a net charge, oxidation number, or unpaired electron, while vertex labels are reserved for atom types. In that manner, all changes in electron distributions in a molecule are encoded by edges and their labels. While this separation of atom type and electronic state of an atom is not necessary, it simplifies some aspects of the presentation by avoiding the need to explicitly discuss changes in vertex labels in the course of a reaction.

A graph G' is a subgraph of G if $V(G') \subseteq V(G)$, $E(G') \subseteq E(G)$, and $xy \in E(G')$ implies $x, y \in V(G')$. Moreover, G' is the subgraph of G induced by $W \subseteq V(G)$ if $V(G') = W$ and

for all $x, y \in V(G')$ we have $xy \in E(G')$ if and only if $xy \in E(G)$. Two labeled graphs G and G' are isomorphic if there exists a bijection $\varphi : V(G) \to V(G')$ that preserves adjacency and labels, that is (1) $xy \in E(G)$ if and only if $\varphi(x)\varphi(y) \in E(G')$, and (2) $a_{G'}(\varphi(x)) = a_G(x)$ for all $x \in V(G)$ and $b_{G'}(\varphi(x)\varphi(y)) = b_G(xy)$ for all $xy \in E(G')$. The map φ is an isomorphism from G to G'. If φ is an isomorphism from G to itself, then it is called an automorphism of G.

Atom-atom maps. An AAM for a reaction $G \longrightarrow H$ is simply a bijective map $\alpha : V(G) \to V(H)$ that preserves vertex labels. Recall that information e.g. on charges is carried by a loop if required. Since AAM tools generate their own vertex numbering rather than making use of a user-defined input numbering, they operate on different, albeit isomorphic graphs. AAMs therefore need to be compared w.r.t. isomorphism rather than identity.[1,3] Two AAMs $\alpha : V(G) \to V(H)$ and $\beta : V(G') \to V(H')$ are said to be equivalent if and only if there are isomorphisms $\varphi : V(G) \to V(G')$ and $\psi : V(H) \to V(H')$ such that $\beta \circ \varphi = \psi \circ \alpha$, where $\circ$ denotes the concatenation of maps, which we write from left to right, i.e., $(\psi \circ \alpha)(x) = \psi(\alpha(x))$ for all $x \in V(G)$.

ITS graph. The ITS graph of a reaction $G \longrightarrow H$ with AAM α, denoted by $\Upsilon(G, H, \alpha)$, is defined as follows: (i) Corresponding atoms in G and H, i.e., vertices $x \in V(G)$ and $\alpha(x)$ are identified and associate with the label $a_\Upsilon(x) = (a_G(x), a_H(\alpha(x)))$; (ii) xy is an edge in the ITS graph if $xy \in E(G)$ or $\alpha(x)\alpha(y) \in E(H)$. The labels are defined as $b_\Upsilon(xy) = (b_G(xy), b_H(\alpha(x)\alpha(y)))$ if $xy \in E(G)$ and $\alpha(x)\alpha(y) \in E(H)$. If there is an edge in G only, we use $b_\Upsilon(xy) = (a_G(xy), \varepsilon)$. Analogously, for an edge in H only we set $b_\Upsilon(xy) = (\varepsilon, b_H(\alpha(x)\alpha(y)))$. In this work, the symbol ε in the label pairs is a reserved constant implying that a bond between to atoms x and y is either broken or inserted. Changes in the electronic state of an atom are indicated by the label at a loop and do not need separate consideration.

Partial AAMs. A practical complication arises from the fact that most AAM tools in general do not produce a complete AAM $\alpha : V(G) \to V(H)$ but return only a partial map α'. In particular, α' may not cover hydrogen atoms, and – depending on the tool being used

– may also omit some heavy atoms. The partial bijection $\alpha' : U \to W$, with $U \subseteq V(G)$ and $W \subseteq V(H)$, may be considered as an AAM between the induced subgraphs $G[U]$ and $H[W]$. Clearly, the ITS graph for the restricted map is the subgraph of the ITS of the full reaction *induced* by the mapped vertices:

$$\Upsilon(G[U], H[W], \alpha') = \Upsilon(G, H, \alpha)[U] \tag{1}$$

Reaction center. The atoms and bonds taking part in a reaction constitute the reaction center.[4] More precisely, given an AAM $\alpha : V(G) \to V(H)$, vertex $x \in V(G)$ is contained in the reaction center if it, or its counterpart $\alpha(x) \in V(H)$ is incident to a bond that changes during the reaction. This can be expressed in terms of the ITS graph $\Upsilon := \Upsilon(G[U], H[W], \alpha')$: an edge $xy \in E(\Upsilon)$ is a *reaction edge* if and only if the edge label $b_\Upsilon(xy) =: (b_1, b_2)$ satisfies $b_1 \neq b_2$. In this case, the bond is either formed, broken, or changes its bond type. A vertex is called a *reaction vertex* if it is incident to a reaction edge in the ITS. The *reaction center* $\Gamma := \Gamma(G, H, \alpha)$ is the subgraph of the ITS Υ formed by all and only the reaction edges and the vertices incident to them, i.e., induced by the changing edges.

A.2 Comparison of Partial ITS and Ensemble Learning

A.2.1 Consistency of Partial ITS Graphs

In this section, we expand the body of mathematical results on partial ITS graphs that some of us developed in[2] . The notation follows this publication. As in,[2] we assume that different tools provide their predicted AAMs in terms of their own renumbering of atoms (vertices). Thus, AAMs produced by different tools for the same input are not defined on the same (input) graphs but rather on isomorphic representations. We consider two complete AAMs $\alpha : V(G) \to V(H)$ and $\beta : V(G') \to V(H')$, where $G \cong G'$ and $H \cong H'$ are isomorphic pairs of graphs. Thus $G \longrightarrow H$ and $G' \longrightarrow H'$ represent the same chemical reaction.

In the following, we denote by Ψ_r and Ψ'_r the (unique) extended reaction centers at radius r, i.e. subgraphs of $\Upsilon(G, H, \alpha)$ and $\Upsilon(G', H', \beta)$, that contain the reaction centers $\mathit{\Gamma}(G, H, \alpha)$ and $\mathit{\Gamma}(G', H', \beta)$, as well as all vertices in the reactant and product graphs within the same distance of at most r from a vertex in the reaction centers. Our starting point is Def. 9 in.[2]

Definition A.1. For two reactions $G \longrightarrow H$ and $G' \longrightarrow H'$, two partial AAMs $\pi : U \to W$ with $U \subseteq V(G)$ and $W \subseteq V(H)$, and $\pi' : U' \to W'$ with $U' \subseteq V(G')$ and $W' \subseteq V(H')$, are said to be *consistent*, if there are isomorphisms $\varphi : V(G) \to V(G')$ for $G \cong G'$ and $\psi : V(H) \to V(H')$ for $H \cong H'$, such that the union $\gamma := \pi \cup (\psi^{-1} \circ \pi' \circ \varphi) : U \cup \varphi^{-1}(U') \to W \cup \psi^{-1}(W')$ is well-defined and bijective.

The following technical result confirms that the "overlap" of two consistent partial AAMs is indeed a common induced subgraph of the corresponding partial ITS graphs.

Proposition 1. *Let $\pi : U \to W$ and $\pi' : U' \to W'$ be consistent partial AAMs, and set $\tilde{U} := U \cap \varphi^{-1}(U')$ and $\tilde{W} := W \cap \psi^{-1}(W')$. Let $\tilde{\pi}$ be the restriction of π to $\tilde{U}$. Then the partial ITS graph $\Upsilon(G[\tilde{U}], H[\tilde{W}], \tilde{\pi})$ is well-defined and isomorphic to a common induced subgraph of $\Upsilon(G[U], H[W], \pi)$ and $\Upsilon(G'[U'], H'[W'], \pi')$.*

Proof. Since π and π' are consistent there are isomorphisms $\varphi : V(G) \to V(G')$ for $G \cong G'$ and $\psi : V(H) \to V(H')$ for $H \cong H'$ such that the map $\gamma : U \to W$ given by $\gamma(x) := \pi \cup (\psi^{-1} \circ \pi' \circ \varphi(x))$ is a well-defined function and in particular bijective. The well-definedness of γ implies that $\pi(x) = \psi^{-1}(\pi'(\varphi(x)))$ holds for all $x \in \tilde{U}$, and since the restrictions of γ and of all these maps to $\tilde{U}$ are also bijections, we can write $\psi(\pi(x)) = \pi'(\varphi(x))$. Denote by $\tilde{\pi}$ and $\tilde{\pi}'$ the restrictions of π and π', respectively, to $\tilde{U}$ and to $\varphi(\tilde{U})$, so we can rewrite the last expression as $\tilde{\psi} \circ \tilde{\pi} = \tilde{\pi}' \circ \tilde{\varphi}$, where $\tilde{\varphi}$ is the restriction of φ to $\tilde{U}$ and $\tilde{\psi}$ of ψ to $\pi(\tilde{U})$. Note that $\tilde{\varphi}$ and $\tilde{\psi}$ are isomorphisms for the graphs $G[\tilde{U}] \cong G'[\tilde{\varphi}(\tilde{U})]$, and $H[\tilde{\pi}(\tilde{U})] \cong H'[\tilde{\psi}(\tilde{\pi}(\tilde{U}))] = H'[\tilde{\pi}'(\tilde{\varphi}(\tilde{U}))]$, respectively. Thus, $\tilde{\pi}$ and $\tilde{\pi}'$ are, by definition, equivalent AAMs over these (induced) graphs. By Corollary 1 in,[1] this equivalence is characterized by the isomorphism of the graphs $\Upsilon(G[\tilde{U}], H[\tilde{\pi}(\tilde{U})], \tilde{\pi}) \cong \Upsilon(G'[\tilde{\varphi}(\tilde{U})], H'[\tilde{\pi}'(\tilde{\varphi}(\tilde{U}))], \tilde{\pi}')$. Moreover, note that the

following (induced) subgraph relations hold: $\Upsilon(G[\tilde{U}], H[\tilde{\pi}(\tilde{U})], \tilde{\pi}) \subseteq \Upsilon(G[U], H[W], \pi)$ and $\Upsilon(G'[\tilde{\varphi}(\tilde{U})], H'[\tilde{\pi}'(\tilde{\varphi}(\tilde{U}))], \tilde{\pi}') \subseteq \Upsilon(G'[U'], H'[W'], \pi')$.

Finally, we show that $\tilde{\pi}(\tilde{U}) = \tilde{W}$. Consider $y \in \tilde{\pi}(\tilde{U}) \subseteq W$. Then there exists $x \in \tilde{U} = U \cap \tilde{\varphi}^{-1}(U')$ such that $\tilde{\pi}(x) = y$. Thus there is also $z \in U'$ for which $\tilde{\varphi}(x) = z$, or equivalently $\tilde{\varphi}^{-1}(z) = x$. Using the observation at the beginning of the proof we obtain $y = \tilde{\pi}(x) = \tilde{\psi}^{-1}(\tilde{\pi}'(\tilde{\varphi}(x))) = \tilde{\psi}^{-1}(\tilde{\pi}'(\tilde{\varphi}(\tilde{\varphi}^{-1}(z)))) = \tilde{\psi}^{-1}(\tilde{\pi}'(z))$ where $\tilde{\pi}'(z) \in W'$, and thus $y \in \tilde{\psi}^{-1}(W')$. Therefore, $y \in W \cap \tilde{\psi}^{-1}(W') \subseteq \tilde{W}$, implying that $\tilde{\pi}(\tilde{U}) \subseteq \tilde{W}$. By similar arguments $\tilde{W} \subseteq \tilde{\pi}(\tilde{U})$. Therefore $\Upsilon(G[\tilde{U}], H[\tilde{\pi}(\tilde{U})], \tilde{\pi}) = \Upsilon(G[\tilde{U}], H[\tilde{W}], \tilde{\pi})$, from which the statement of the proposition follows. □

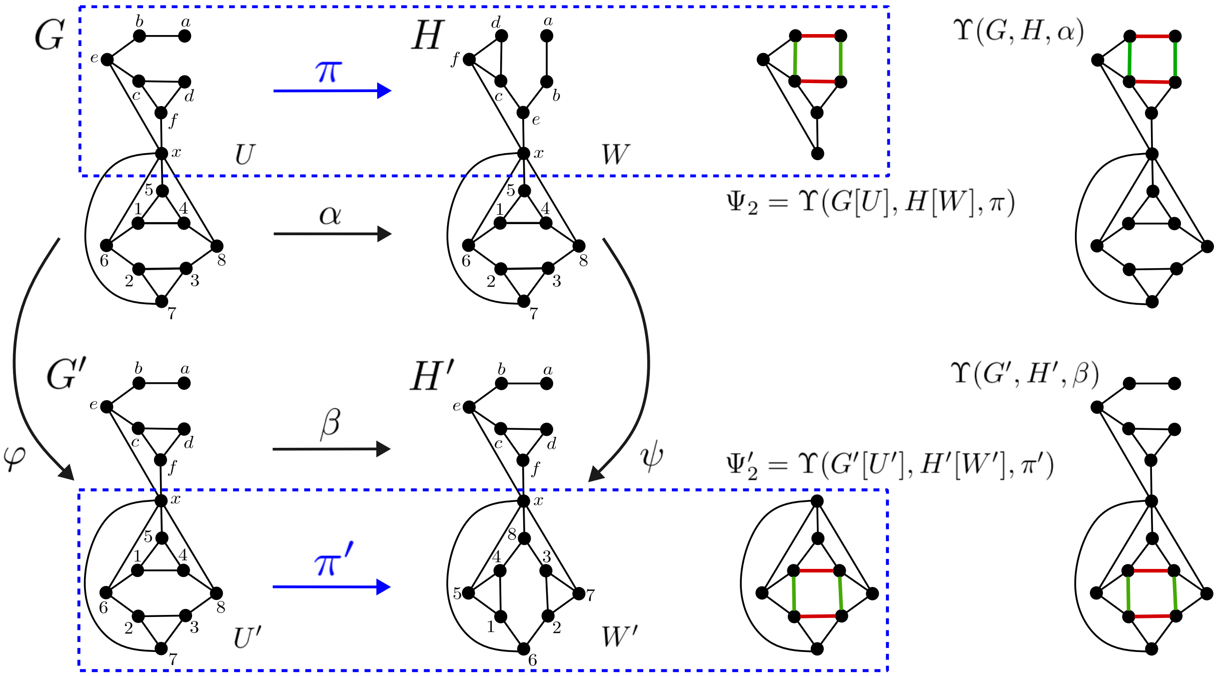

Figure S1: Consistency of π and π' is not sufficient to imply isomorphism of the reaction center graphs Ψ_{r_0} and Ψ'_{r_0}. Consider $G \simeq H \simeq G' \simeq H'$. The partial maps π for $G[U] \longrightarrow H[W]$ and π' for $G'[U'] \longrightarrow H'[W']$ are consistent through the isomorphisms $\varphi : V(G) \to V(G')$ and $\psi : V(H) \to V(H')$, by only mapping in common the vertex x into itself, i.e., $\pi(x) = \psi^{-1}(\pi'(x)) = x$, while $U \setminus \{x\}$ and $\varphi^{-1}(U') \setminus \{x\}$ remain disjoint. Despite these maps being consistent and producing isomorphic reaction centers $\Gamma(G[U], H[W], \pi)$ and $\Gamma(G'[U'], H'[W'], \pi')$, the extended reaction centers Ψ_{r_0} and Ψ'_{r_0} at radius $r_0 = 2$ are not isomorphic. By Proposition 3 below, furthermore, it follows that the complete maps α and β are not consistent, and thus also not equivalent, which is also verified by the non-isomorphism of the ITS graphs $\Upsilon(G, H, \alpha)$ and $\Upsilon(G', H', \beta)$.

Let us now consider two partial AAMs π and π' that correspond to extended reaction centers Ψ_{r_0} and Ψ'_{r_0}, resp., for a fixed expansion radius $r_0 > 0$. Given that these subgraphs include the reaction centers and *all* vertices at a distance at most r_0 from the reaction center, it is tempting to conjecture that consistency of π and π' implies the isomorphism $\Psi_{r_0} \cong \Psi'_{r_0}$. Somewhat surprisingly, this is not true, however. A counterexample can be found in Figure S1. The situation simplifies, nonetheless, if π and π are complete AAMs because in this case consistency becomes the same as equivalence. For completeness, we include a proof of this simple technical result:

Lemma 2. *Let $\alpha : V(G) \to V(H)$ and $\beta : V(G') \to V(H')$ be complete AAMs for a balanced reaction $G \longrightarrow H$. Then, α and β are consistent if and only if α and β are equivalent AAMs.*

Proof. First we suppose that α and β are consistent. Since they are complete AAMs, following the notation in Proposition 1 by setting $\pi = \alpha$ and $\pi' = \beta$, we see that $\tilde{U} = U \cap \varphi^{-1}(U') = V(G) \cap \varphi^{-1}(V(G')) = V(G)$ and $\tilde{W} = W \cap \psi^{-1}(W') = V(H) \cap \psi^{-1}(V(H')) = V(H)$ for isomorphisms $\varphi : V(G) \to V(G')$ and $\psi : V(H) \to V(H')$. But in this case we also have $\Upsilon(G[U], H[W], \pi) = \Upsilon(G, H, \alpha)$ and $\Upsilon(G'[U'], H'[W'], \pi') = \Upsilon(G', H', \beta)$, and since these (induced) graphs have all the same order, following again the notation of the proposition we get $\Upsilon(G, H, \alpha) = \Upsilon(G[\tilde{U}], H[\tilde{W}], \tilde{\pi}) \cong \Upsilon(G', H', \beta)$, which implies the equivalence of α and β by Corollary 1 of.[1]

Suppose, on the other hand, that α and β are equivalent AAMs. By definition there exist isomorphisms $\varphi : V(G) \to V(G')$ and $\psi : V(H) \to V(H')$ such that $\psi \circ \alpha = \beta \circ \varphi$ holds, and equivalently we can write $\alpha = \psi^{-1} \circ \beta \circ \varphi$. Then, following the notation stablished in the definition of the consistency of (partial) AAMs above, we see that $\gamma = \pi \cup (\psi^{-1} \circ \pi' \circ \varphi) = \alpha \cup (\psi^{-1} \circ \beta \circ \varphi) = \alpha$ is a well-defined and bijective function from $U \cup \varphi^{-1}(U') = V(G)$ to $W \cup \psi^{-1}(W') = V(H)$, meaning that α and β are consistent AAMs, which completes the proof. □

As a consequence, we can now show that the extended reaction centers are also isomor-

phic:

Proposition 3. *Let $\alpha : V(G) \to V(H)$ and $\beta : V(G') \to V(H')$ be two consistent complete atom maps. Then the extended reaction centers Ψ_r and Ψ'_r are isomorphic for every radius $r \geq 0$.*

Proof. Since α and β are consistent, then they are also equivalent by Lemma 2, and from Corollary 1 of[1] it follows that $\Upsilon(G, H, \alpha) \cong \Upsilon(G', H', \beta)$. Given any isomorphism η from $\Upsilon := \Upsilon(G, H, \alpha)$ to $\Upsilon' := \Upsilon(G', H', \beta)$, moreover, the restriction η' of η to any subgraph $S \subseteq \Upsilon$, is also an isomorphism from S to $\eta'(S) := \Upsilon'[\eta'(E(S))] \subseteq \Upsilon'$. Consider the restriction η_0 of η to $\Psi_0 := \Gamma(G, H, \alpha)$. Then $\eta_0(\Psi_0) = \Gamma(G', H', \beta) = \Psi'_0$, since these subgraphs contain all and only the reaction edges, which proves the statement for $r = 0$. Graph isomorphisms in particular preserve paths. Thus, all vertices in Υ at a given distance from $\Gamma(G, H, \alpha)$ must have a corresponding image in Υ' under any isomorphism η, preserving their adjacency and distances from $\Gamma(G', H', \beta)$. Therefore, the restriction η_r of η to the extended reaction center Ψ_r is also an isomorphism from Ψ_r to Ψ'_r, since these subgraphs are unique w.r.t the inclusion of the reaction centers and the closure under vertices at distance at most r from them, which holds for every $r > 0$ as required. □

A.2.2 Comparison of Atom-atom Mappings

As described in,[2] the comparison of partial AAMs is a non-trival task. Here we use a pragmatic approximation: Given two partial AAMs $\varphi : U \to W$ for subsets $U \subseteq V(G)$ and $W \subseteq V(H)$ and $\varphi : U' \to W'$ for subsets $U' \subseteq V(G')$ and $W' \subseteq V(H')$ we compute the overlap of mapped atom using an isomorphism between G and G' and H and H'. Then we check whether the restrictions of the ITS graphs to these common vertices are isomorphic. In order to speed up comparisons of predicted AAMs with the annotated ground truth, we first check whether the reaction centers are isomorphic. If true, we checked whether the smaller partial ITS graph is a subgraph of the larger one. Although this procedure is not exact, it provides a very stringent test in practice.

For comparative analysis of SynTemp against CGRTools for AAMs comparison, the USPTO_3K dataset[5] from the study of Shuan et.al. was utilized directly, using two types of ground truth. According to,[5] LocalMapper demonstrated a 100% accuracy rate for these datasets. Consequently, we adopted LocalMapper as our primary ground truth, denoted as $\mathcal{U}_1$. Furthermore, to ensure an unbiased comparison, we established an additional ground truth, $\mathcal{U}_2$, based on a distinct criterion. Instead of depending solely on LocalMapper, which provided accurate results, this alternative benchmark derives from the reaction that produced the maximum number of atom mappings, each verified as correct by a chemist. In this analysis, to ensure consistency and avoid discrepancies arising from different software versions, we directly utilized the correctness values reported in the USPTO_3K dataset,[5] rather than reproducing the values ourselves.

Furthermore, we conducted a detailed benchmarking study of four atom-atom mapping tools: RXNMapper 0.3.0, GraphormerMapper 1.75, LocalMapper 0.1.4, and RDTool 2.4.1. Recent benchmarking studies by Lin et al.[6] have shown that ChemAxon, Indigo, and NameRXN perform suboptimally; therefore, they were excluded from our analysis. In order to increase the accuracy we considered two ensemble strategies integrating predictions of the following subsets of tools:

- *Ensemble_1*: RXNMapper, GraphormerMapper, and LocalMapper.
- *Ensemble_2*: Extends *Ensemble_1* by incorporating RDTool.

We checked for isomorphism of the reaction centers Γ_i obtained with tool i using the VF2 algorithm. Instances in which the tools disagreed were considered unsuccessful, and no prediction of the AAM was returned.

A.2.3 Benchmarking Results

In this analysis, we examined the validation of two chemical datasets ($\mathcal{U}_1$ and $\mathcal{U}_2$) using the CGRTools. Our findings indicated that LocalMapper aligned perfectly with the ground truth

for $\mathcal{U}_1$ (100% accuracy), highlighting its efficacy. However, when the alternative dataset $\mathcal{U}_2$ was introduced, `CGRTools` exhibited significant performance discrepancies, as detailed in Table S1. `GraphormerMapper`, in this case, produced results with high consistency, achieving an accuracy of 95.10% relative to the ground truth. These variations suggest that `CGRTools` performance is sensitive to dataset specifics, such as the number of atom mappings. Conversely, our method, `SynTemp`, demonstrated superior consistency and reliability maintaining robust accuracy across different datasets, as documented in Table S1.

Table S1: Comparison of atom-atom map validation tools

	`RXNMapper`	`GraphormerMapper`	`LocalMapper`
`CGRTools` $\mathcal{U}_1$ (%)	92.63	93.20	100.00
`CGRTools` $\mathcal{U}_2$ (%)	91.27	95.10	98.10
`SynTemp` (%)[1]	93.53	95.10	100.00
Ground Truth (%)	93.53	95.10	100.00

Despite achieving the same accuracy as the ground truth, we further explored the discrepancies in individual reaction verification results between `SynTemp` and the ground truth. Notably, we identified two reactions mapped by `RXNMapper` with contrasting assessments, as depicted in Figure S2. For the first reaction (Figure S2A-B), chemists confirmed that `RXNMapper` correctly recognized ethanol and ethanolate in solution as equivalent, while our tool labeled this identification as erroneous. In aqueous solutions, ethanol and ethanolate are often considered interchangeable due to their equilibrium, governed by the equilibrium constant. However, from the perspective of organic chemical mechanisms, it is crucial to distinguish between these two species. Ethanolate, being a conjugate base of ethanol, exhibits significantly higher nucleophilic activity. This distinction is critical in nucleophilic substitution reactions, where ethanolate can effectively participate, whereas ethanol typically cannot due to its relatively lower nucleophilicity. Thus, despite their apparent similarity in analytical chemistry, their roles in organic reactions, particularly in terms of reactivity and mechanism, are fundamentally different. Conversely, for the subsequent reaction (Figure S2C-D), chemists deemed the reaction incorrect, whereas our evaluation classified it as

correct. Manual verification subsequently validated the accuracy of `RXNMapper` for the second reaction. This posed limitations in human verification processes, especially when assessing thousands of reactions where misjudgments are feasible. Consequently, `SynTemp` demonstrated enhanced stringency and accuracy compared to the traditional manual evaluations conducted by chemists.

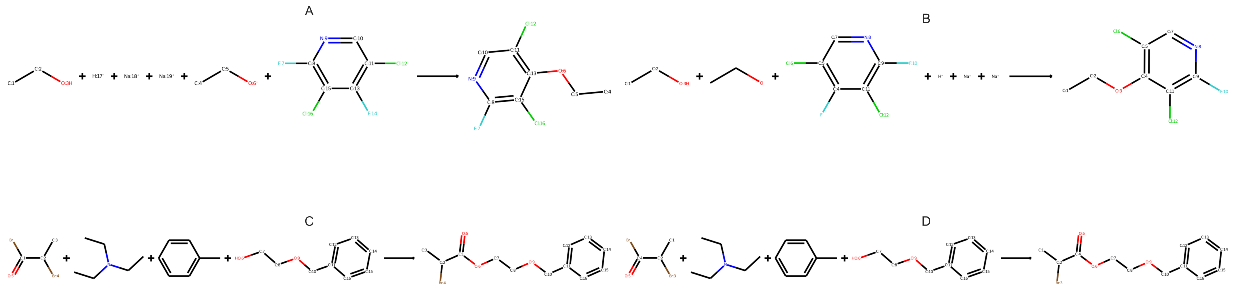

Figure S2: Conflict verification between chemists and `SynTemp` for selected reactions in the USPTO_3K dataset. (A) Ground truth mapping of reaction index 192, (B) `RXNMapper` mapping for the same reaction, marked as correct by chemists but deemed incorrect by `SynTemp`. (C) Ground truth mapping of reaction index 2157, (D) `RXNMapper` mapping for this reaction, marked as incorrect by chemists but validated as correct by `SynTemp`.

A challenge posed by tautomerism[7,8] is the occurrence of "chemically equivalent" atoms that do not share a common orbit within any automorphism of the molecular graph. This issue is particularly pertinent when comparing AAMs and corresponding ITS graphs, as non-isomorphic mappings can still accurately represent the same chemical reaction. A notable example of this phenomenon is illustrated in Figure S3. `CGRTools` struggle to verify the correctness of certain reactions because they do not account for tautomerization effects. Specifically, in acetic acid, the functional groups O^3 and O^4H are treated as distinct, despite their ability to tautomerize and exchange hydrogens. In contrast, `SynTemp` effectively addresses this limitation by enumerating all potential tautomers, thus verifying the reaction as correct. Consequently, `SynTemp` is considerably more accurate than `CGRTools` in this regard.

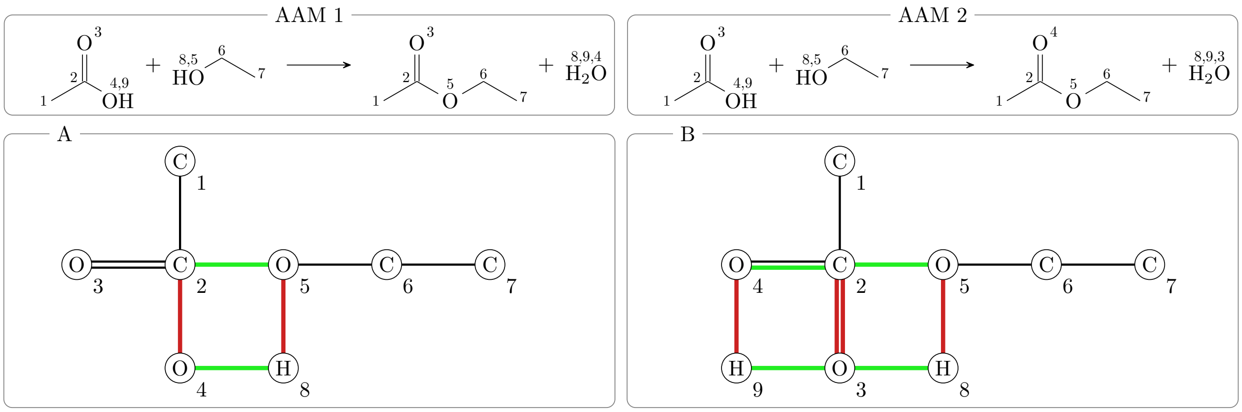

Figure S3: The effect of tautomerism results in chemically identical AAMs for structures with O3 and O4, highlighting their symmetry. However, there is no isomorphism observed between their corresponding ITS graphs. In these graphs, green lines indicate newly forming bonds, red lines represent broken bonds, and orange lines denote bonds that are broken twice

A.3 ITS Graphs and DPO Graph Transformation Rules

The application of the rule p to an input graph G requires, first, that the pattern specified by the left graph L appears in G, i.e., L is (isomorphic to) a subgraph of G and hence there exists a matching morphism $m : V(L) \to V(G)$ that is injective and preserves edges. Second, it must be possible to determine the graphs D and H together with matching morphisms $m' : V(K) \to V(D)$ and $m'' : V(R) \to V(H)$ and subgraph embeddings g and h such that $m \circ l = g \circ m'$ and $m'' \circ r = h \circ m'$. As shown in the study of Andersen et al.,[9] these conditions are necessary and sufficient to satisfy the more general requirements for DPO graph rewriting in the case of chemical systems, where rules always induce bijections on the vertices. In particular, the maps g and h together determine the AAM α for transforming the reactant graph G into the product graph H upon application of the rule p. The application of p with the help of specific matching morphism m to G unambiguously determines the product graph H. This is called a direct derivation and is denoted by $G \overset{p,m}{\Rightarrow} H$. A direct derivation, by definition, determines the embeddings g and h and thus also the AAM $\alpha = h \circ g^{-1}$. Therefore, it also unambiguously determines the ITS graph $\Upsilon(G, H, \alpha)$ of the reaction.

The graph D, moreover, is the subgraph of the ITS graph comprising the non-reaction edges. The edges appearing in G but not in D are those that are broken or change their order,

while those in H are the ones that are newly formed or have their order changed as well. The rule p itself also defines an ITS-like graph $\Upsilon(L, R, r \circ l^{-1})$ that captures the extended reaction center, i.e., all bonds that change together with necessary "context" prescribed by K. It is not difficult to verify that the ITS graph of the rule $\Upsilon(L, R, r \circ l^{-1})$ is (isomorphic to) a subgraph – but not necessarily an induced subgraph – of the ITS $\Upsilon(G, H, \alpha)$. By construction $\Upsilon(L, R, r \circ l^{-1})$ contains the reaction center, i.e., all bonds that change during the reaction. A more formal discussion based on[1] is summarized as follows:

Proposition 4. *Consider a reaction* $G \longrightarrow H$ *with AAM* α *and let* $\Gamma \subseteq \Upsilon(G, H, \alpha)$ *be a subgraph of the ITS that contains the reaction center. Then there is a unique DPO rule* $p = (L \overset{l}{\leftarrow} K \overset{r}{\rightarrow} R)$ *with* $\Upsilon(L, R, r \circ l^{-1})$ *isomorphic to* Γ. *Moreover, there exists a matching morphism* $m : V(L) \to V(G)$ *such that* $G \overset{p,m}{\Rightarrow} H$ *is well-defined.*

Proposition 4 essentially establishes a 1-1 correspondence between DPO rules and subgraphs of ITS graphs. Hence it reduces the problem of inferring reaction rules to the graph theoretical problem of identifying informative subgraphs.

A.4 Extended Reaction Centers

Definition A.2. For a given connected ITS Υ, the graph $Q_j = Q_j(\Upsilon)$ is obtained from the reaction center Γ by adding all paths of length at most j starting at a vertex of Γ.

Lemma 5. *The family of graphs* $\mathcal{Q}(\Upsilon) := \{Q_j | j \geq 0\}$ *is finite, nested (i.e.,* $Q_{j-1} \subseteq Q_j$*), and contains* $\Gamma = Q_0$ *and* $\Upsilon = Q_{|V(\Upsilon)|-1}$ *as its minimal and maximal elements, respectively. Moreover,* $\Upsilon[V(Q_{j-1})] = Q_j[V(Q_{j-1})]$ *for all* $j \geq 0$.

Proof. Since Υ is finite, it contains only finitely many subgraphs. By construction $Q_0 = \Gamma \in \mathcal{Q}(\Upsilon)$. Since a graph cannot contain a path longer than $n - 1$, where n is the number of vertices, and given that Υ is connected, then Q_{n-1} contains all paths and therefore all edges of Υ. Thus we have $Q_{|\Upsilon|-1} = \Upsilon$. Moreover, every path from v to x of length j is an extension of a path of length $j - 1$ from v to some x' by the edge $x'x$. Thus Q_j contains

all vertices and edges of Q_{j-1}, i.e., $Q_{j-1} \subseteq Q_j$. Now let $x, y \in V(Q_{j-1})$ be adjacent in Υ but not in Q_{j-1}. Then, by construction, there is a path of length at most $j-1$ from some $v \in V(\Gamma)$ to x and from $v' \in V(\Gamma)$ to y. These paths can be extended by the edge xy to paths on length at most j from v to y and v' to x, respectively, and thus $xy \in E(Q_j)$. That is, Q_j contains all edges of Υ induced by $V(Q_{j-1})$ and only these since $Q_j \subseteq \Upsilon$. □

Let us now consider the extended reaction centers $Q_r^{(p)}$ and $Q_{r'}^{(q)}$ of two different reactions p and q with extension radii r and r', respectively. As a consequence of Lemma 5 we have

Corollary 6. *If* $Q_r^{(p)} \cong Q_r^{(q)}$ *then* $Q_s^{(p)} \cong Q_s^{(q)}$ *for all* $0 \leq s \leq r$.

Proof. Any isomorphism of $Q_r^{(p)}$ and $Q_{r'}^{(q)}$ in particular must map reaction vertices to reaction vertices, and thus induce a isomorphism on the reaction edges. Since path lengths are preserved by isomorphisms, the same is true for all vertices $i = 1, 2, \ldots$ steps away from the reaction center, and thus $Q_s^{(p)} \cong Q_s^{(q)}$. □

B Clustering of Partial ITS Graphs

B.1 Clustering algorithms

Given a set of N reactions, we extract N ITS graphs $\{\Upsilon_1, \Upsilon_2, \ldots, \Upsilon_N\}$, each producing M_i reaction templates (with expansion radii r) denoted by $Q_r^{(i)}$ for $i = 1, \ldots, N$ and $r = 1, \ldots, M_i$. Alg. S1 summarizes the implementation of the clustering of this set by isomorphisms:

Algorithm S1 `Cluster(`$Q_r^{(i_1)}, Q_r^{(i_2)}, \ldots, Q_r^{(i_L)}$`)`

Input: set $\mathcal{S} \leftarrow \{Q_r^{(i_1)}, Q_r^{(i_2)}, \ldots, Q_r^{(i_L)}\}$ of $L \geq 2$ templates obtained w.r.t radius r
Output: Partition $\mathcal{T}$ of $\mathcal{S}$ in isomorphism classes
$\mathcal{T} \leftarrow \emptyset$
while $|\mathcal{S}| > 0$ **do**
    $Q^* \leftarrow$ `Select_Arbitrary_Element`$(\mathcal{S})$
    $\mathcal{S}' \leftarrow \mathcal{S} \setminus \{Q^*\}$
    $\mathcal{C} \leftarrow \{Q^*\}$
    for all $Q \in \mathcal{S}'$ **do**
        if `VF2_isomorphic`(Q, Q^*) **then**
            $\mathcal{C} \leftarrow \mathcal{C} \cup \{Q\}$
            $\mathcal{S} \leftarrow \mathcal{S} \setminus \{Q\}$
        end if
    end for
    $\mathcal{T} \leftarrow \mathcal{T} \cup \{\mathcal{C}\}$
end while
return $\mathcal{T}$

We can then expand the "radius" r of $\Gamma = Q_0^{(i)}$ based on Υ_i to form new instances $Q_r^{(i)}$. For better computation, we employ a hierarchical approach, that is, we apply the clustering process starting from the initialization step with these expanded templates, refining each parent-cluster into a child-clusters only when necessary. If it is not possible to expand a certain $Q_r^{(i)}$, that $Q_r^{(i)}$ is carried over for backtracking purposes. The details are given in Alg. S2.

Algorithm S2 `Hierarchical_Clustering`$(kMax;\ \Upsilon_1, \Upsilon_2, \ldots, \Upsilon_N)$

Input: integer $kMax \geq 1$ and set of ITS graphs $\mathcal{Y} \leftarrow \{\Upsilon_1, \Upsilon_2, \ldots, \Upsilon_N\}$
Output: Hierarchical clustering $\mathcal{H}$ of templates up to user-specified radius $kMax$
$\mathcal{T}_0 \leftarrow$ `Cluster`$(\{Q_0^{(i)} \subseteq \Upsilon_i \mid i = 1, \ldots, N\})$ ▷ $\subseteq$ means subgraph
$\mathcal{H} \leftarrow \{\mathcal{T}_0\}$
for $r = 1$ to $kMax$ **do**
    $\mathcal{T}_r \leftarrow \emptyset$
    $T' \leftarrow \emptyset$
    for each C in $\mathcal{T}_{r-1}$ **do**
        $C' \leftarrow \emptyset$
        for each $Q_{r-1}^{(i)}$ in C **do**
            if can expand $Q_{r-1}^{(i)}$ **then**
                $C' \leftarrow C' \cup \{Q_r^{(i)} \subseteq \Upsilon_i\}$ ▷ $Q_{r-1}^{(i)}$ is used to obtain $Q_r^{(i)}$
            else
                $T' \leftarrow T' \cup \{\{Q_{r-1}^{(i)}\}\}$ ▷ $Q_{r-1}^{(i)}$ is carried over for backtracking
            end if
        end for
        $T' \leftarrow T' \cup \{C'\}$
    end for
    for each C in T' **do**
        if $|C| > 1$ **then**
            $\mathcal{T}_r \leftarrow \mathcal{T}_r \cup$ `Cluster`(C) ▷ refinement of cluster C into child clusters
        else
            $\mathcal{T}_r \leftarrow \mathcal{T}_r \cup \{C\}$
        end if
    end for
    $\mathcal{H} \leftarrow \mathcal{H} \cup \{\mathcal{T}_r\}$
end for
return $\mathcal{H}$

B.2 Computational Experiment on Clustering Performance

To demonstrate the efficacy of hierarchical clustering, we performed a comparative benchmark between the conventional empirical clustering approach (outlined in Alg. S1) and hierarchical clustering (described in Alg. S2). Across various radius expansions, hierarchical clustering consistently completed within two minutes, indicating significant efficiency. In contrast, the time required for the empirical clustering method increased substantially, from about one minute at a radius of zero to nearly 95 minutes at a radius of three, as shown in Figure S4A. The cumulative sum plot in Figure S4B further highlights the substantial reduction in computational costs associated with hierarchical clustering. Importantly, both methods produced a consistent number of templates, as reported in Table S3.

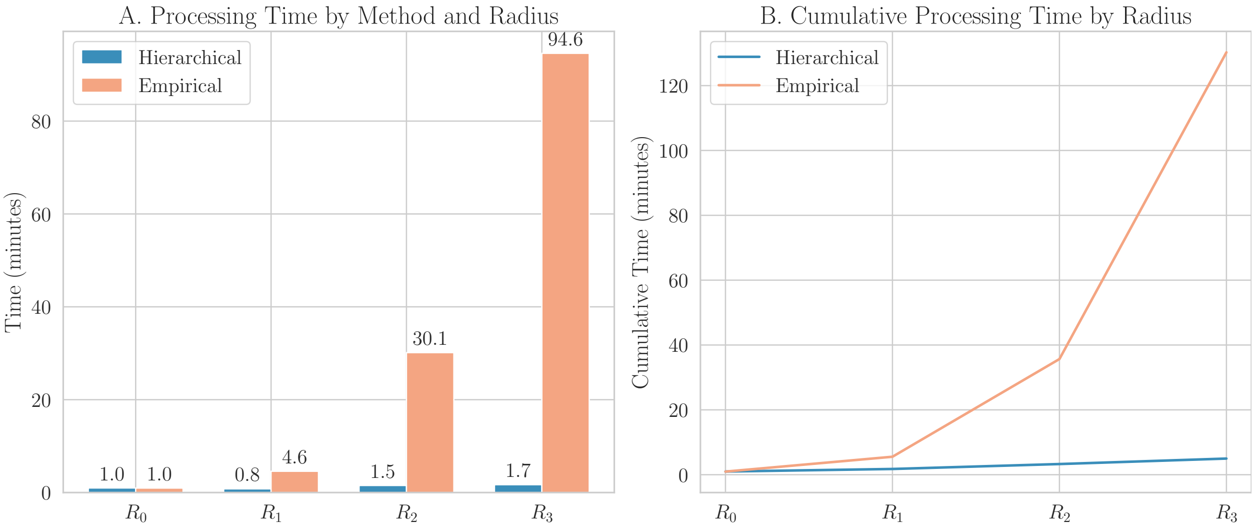

Figure S4: Comparison of computational processing times between empirical clustering and hierarchical clustering methods.

C Additional Figures and Tables

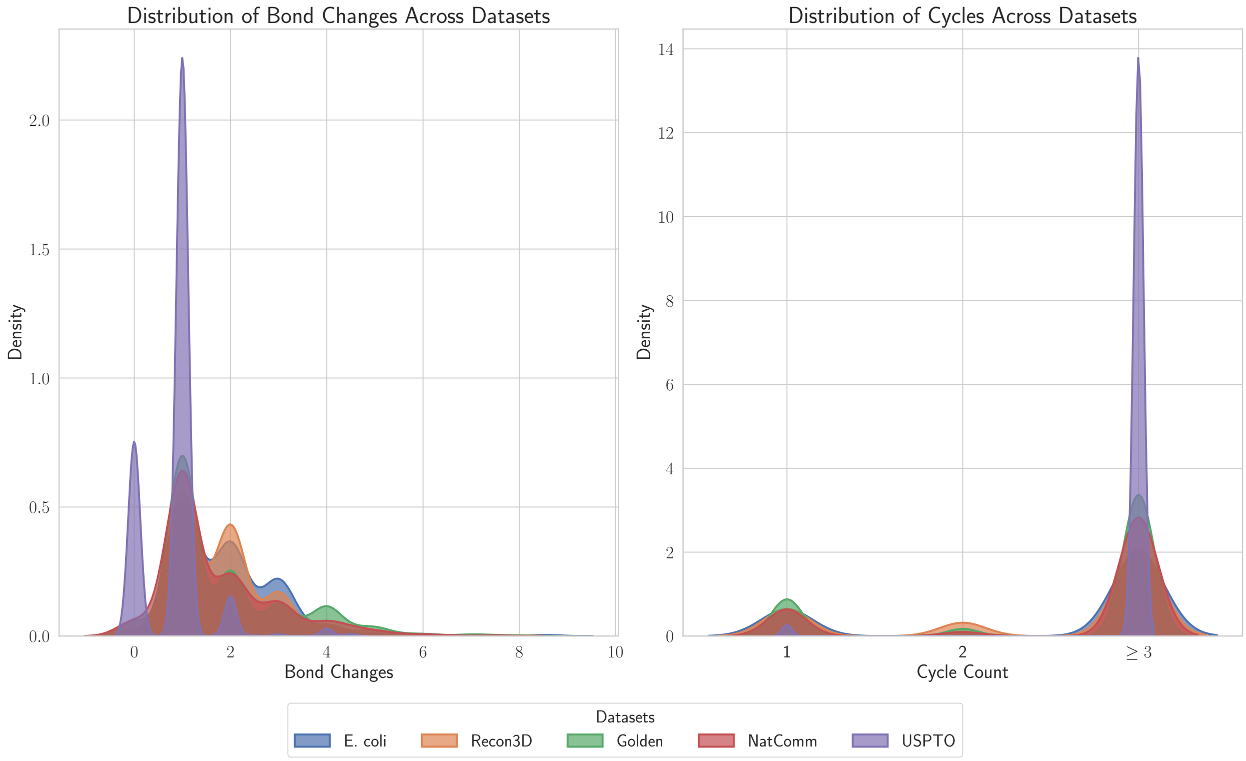

Figure S5: Distribution of reaction types across datasets

Table S2: Processing time (seconds) per dataset for different AAM tools

**Dataset**	`RXNMapper`	`GraphormerMapper`	`LocalMapper`	`RDT`
`E. coli`	5.13	24.71	40.29	1189.93
`Recon3D`	9.88	45.72	83.69	3156.96
`USPTO_3K`	40.38	245.45	311.18	12281.49
`Golden`	20.30	120.64	154.83	5028.39
`NatComm`	5.34	30.89	38.85	1053.32
**Average per Reaction**	0.014	0.078	0.105	4.502

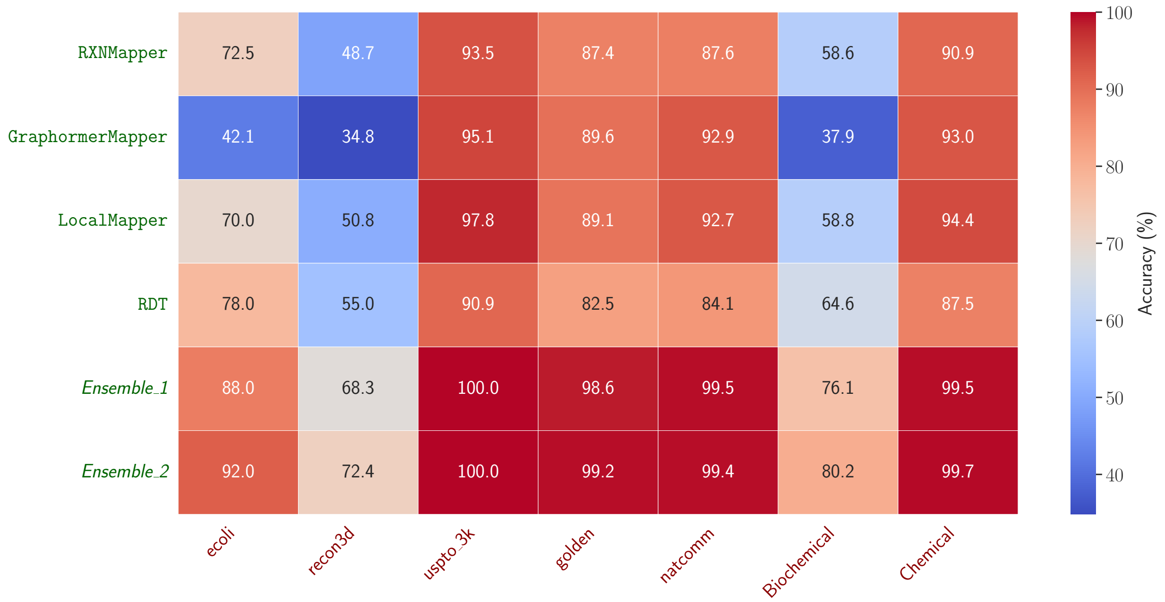

Figure S6: AAMs benchmarking results heatmap

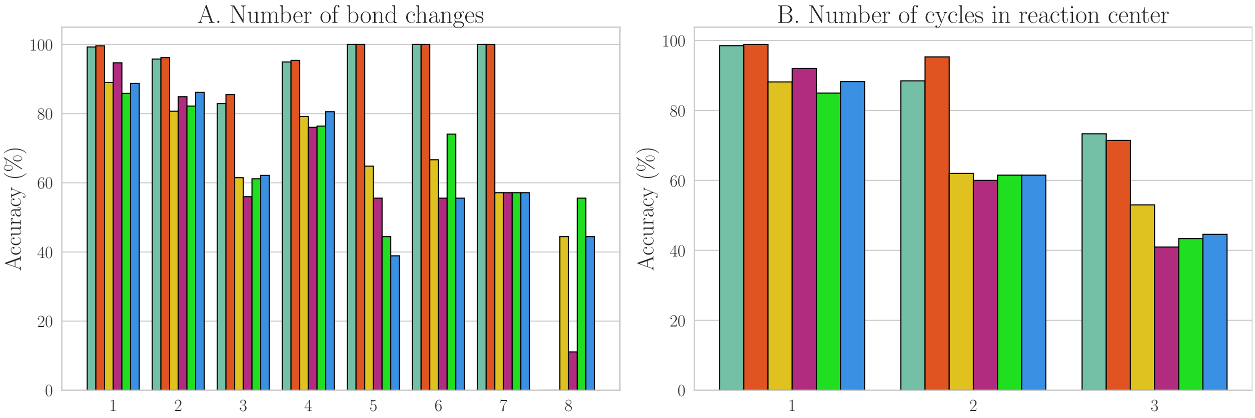

Figure S7: Performance analysis of each mapping tool based on (C) bond changes and (D) cycles within the reaction center.

Table S3: Statistical results of rule extraction for different template types

**Category**	R_0	R_1	R_2	R_3
Q_{raw}	313	1577	9798	22248
Q_{complete}	311	1552	9699	22104
Q_{hier}	311	1552	9699	22104

Table S4: Distribution of topological descriptors within the "template library" and "database"

Descriptors	Category	Template Library (%)	Database (%)
**Reaction Type**	*Elementary*	54.34	86.97
	Complicated	45.66	13.03
**Topological Type**	*Single Cyclic*	48.55	86.57
	Combinatorial Cyclic	40.84	11.78
	Hybrid Graph	4.82	1.25
	Acyclic	5.79	0.4
**Reaction Step**	1	54.34	86.969
	2	33.12	10.980
	3	7.07	0.346
	4	3.86	0.692
	5	1.29	0.160
	6	0.32	0.003

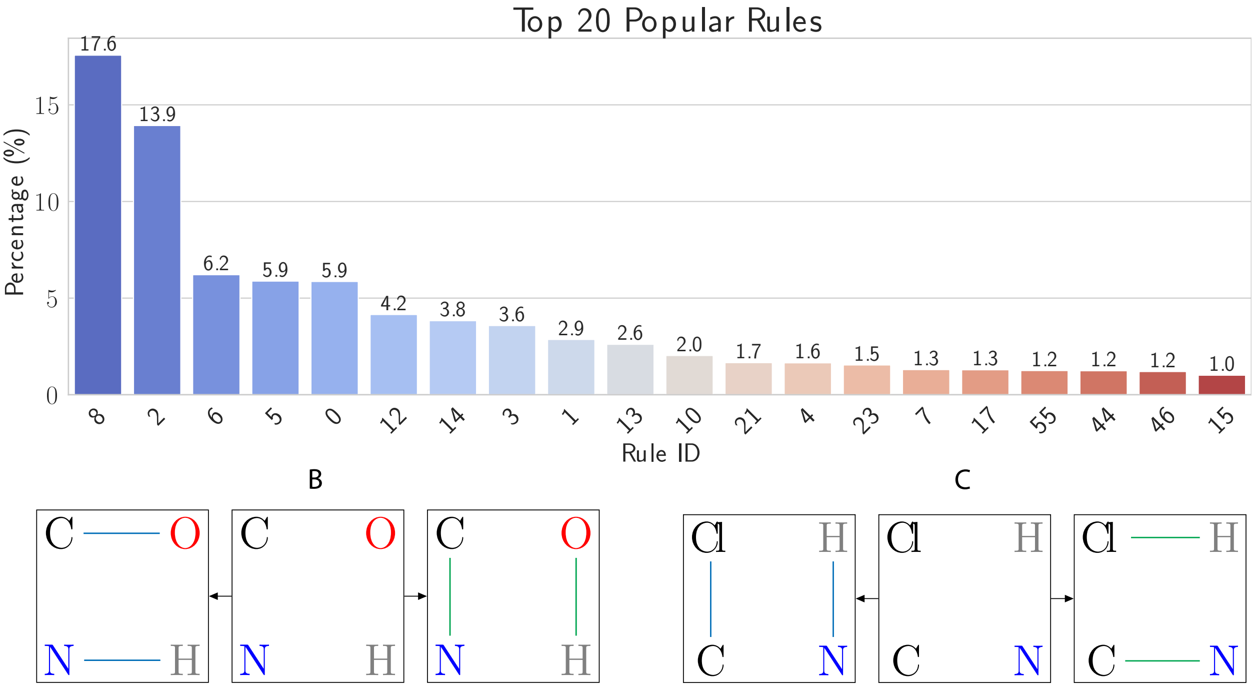

Figure S8: (A) Distribution of the top 20 rules observed in the dataset. (B) Illustration of *Rule 8* showing amide formation. (C) Illustration of *Rule 2* depicting amine alkylation.

Table S5: Analysis of hierarchical descriptors and cycle length proportions across the 'template library" and "database"

Reaction Type	Topological Type	Cycle Length	Template Library (%)	Database (%)
Elementary	*Acyclic Graph*	(0,)	100.0	100.0
	Single Cyclic	(4,)	72.19	98.4
		(6,)	19.21	1.39
		(5,)	5.96	0.17
		(7,)	1.99	0.03
		(8,)	0.66	0.0
Complicated	*Combinatorial Cyclic*	(4, 4)	24.41	73.12
		(3, 3)	1.57	0.96
		(4, 4, 4)	0.79	0.32
		(4, 5, 5)	0.79	0.22
		(3, 5)	8.66	12.37
		(4, 5)	12.6	1.46
		(4, 6)	17.32	1.16
		(4, 4, 5)	6.3	0.54
		(6, 6)	1.57	0.32
		(4, 5, 7)	0.79	0.32
		(5, 5)	1.57	0.07
		(5, 5, 6)	0.79	0.17
		(6, 6, 6)	0.79	0.07
		(4, 4, 6)	0.79	0.64
		(4, 7)	1.57	0.12
		(3, 4)	0.79	0.02
		(4, 4, 4, 7)	0.79	1.16
		(4, 5, 6, 6)	0.79	0.1
		(4, 4, 5, 6)	3.15	0.67
		(4, 5, 5, 6, 8)	0.79	0.2
		(4, 4, 6, 8)	1.57	0.07
		(5, 7, 7)	0.79	0.02
		(5, 5, 5)	0.79	0.02
		(4, 5, 6)	0.79	0.2
		(4, 4, 5, 7)	0.79	0.64
		(4, 6, 8)	0.79	0.02
		(4, 4, 6, 6, 8)	0.79	0.02
		(4, 6, 7, 9)	0.79	0.07
		(5, 6, 8, 9)	0.79	0.02
		(4, 4, 4, 5, 7)	0.79	0.12
	Hybrid Graph	(0, 3)	33.33	76.33
		(0, 4)	46.67	22.97
		(0, 4, 4)	13.33	0.46
		(0, 4, 4, 4, 5, 7)	6.67	0.23

Table S6: Overview of top 20 reaction rules

Rule ID	Mechanistic description	General mechanism	Percentage (%)
8	Amide formation	$A_N + E$	17.9%
2	Amine alkylation (chloro)	S_N	14.2%
6	Amid hydrolysis	$A_N + E$	6.3%
5	Ester hydrolysis	$A_N + E$	6.0%
0	Amine alkylation (bromo)	S_N	6.0%
12	Boron-mediated carbon-carbon bond formation (bromide)	Suzukicoupling	4.2%
14	Reductive amination	$A_N + A_R$	3.9%
3	Hydrolysis of C-Cl bond	S_N	3.6%
1	Hydrolysis of C-Br bond	S_N	2.9%
13	S-Cl bond reaction with NH	$A_N + E$	2.7%
10	Hydroiodination of alcohols	S_N	2.1%
21	Amine alkylation (iodo)	S_N	1.7%
4	Reaction involving NH and C=N	A_N	1.7%
23	Carbonyl reduction	A_R	1.6%
7	Alkene reduction	A_R	1.3%
17	Amine alkylation (fluoro)	S_N	1.3%
55	Reduction of carboxyl group	A_N	1.2%
44	Imination	A_N	1.2%
46	Boron-mediated carbon-carbon bond formation (chloride)	Suzukicoupling	1.2%
15	Sulfur-chlorine hydrolysis	$A_N + E$	1.0%

Table S7: Summary of validation set prediction metrics

Direction	Type	$\mathcal{C}$ (%)				Avg. Solution				$\mathcal{NR}$ (%)			
		R_0	R_1	R_2	R_3	R_0	R_1	R_2	R_3	R_0	R_1	R_2	R_3
Forward	Q_{raw}	7.86	7.24	6.50	4.92	16.69	8.24	3.55	2.62	99.38	98.72	98.19	97.93
	Q_{complete}	94.50	92.92	88.60	78.03	67.52	21.77	5.20	3.17	96.41	88.31	67.11	58.48
	Q_{hier}	94.50	92.92	88.60	78.03	67.52	21.77	5.20	3.17	96.41	88.31	67.11	58.48
Backward	Q_{raw}	7.86	7.24	6.50	4.92	13.74	2.70	0.86	0.45	99.54	98.05	94.17	90.77
	Q_{complete}	93.46	92.84	88.50	77.97	72.49	21.96	13.89	9.38	97.75	92.92	89.22	85.07
	Q_{hier}	93.46	92.84	88.50	77.97	72.49	21.96	13.89	9.38	97.75	92.92	89.22	85.07

Table S8: Summary of test set prediction metrics

Direction	Type	$\mathcal{C}$ (%)				Avg. Solution				$\mathcal{NR}$ (%)			
		R_0	R_1	R_2	R_3	R_0	R_1	R_2	R_3	R_0	R_1	R_2	R_3
Forward	Q_{raw}	7.92	7.70	6.62	5.08	16.46	8.11	3.50	2.55	99.41	98.66	98.17	97.83
	Q_{complete}	93.98	92.46	87.39	76.71	66.01	21.42	5.12	3.13	96.44	88.43	67.57	59.39
	Q_{hier}	93.98	92.46	87.39	76.71	66.01	21.42	5.12	3.13	96.44	88.43	67.57	59.39
Backward	Q_{raw}	7.92	7.70	6.62	5.08	13.63	2.66	0.86	0.45	99.52	97.90	93.73	89.83
	Q_{complete}	93.14	92.38	87.33	76.67	72.28	22.05	13.96	9.40	97.82	92.96	89.31	85.21
	Q_{hier}	93.14	92.38	87.33	76.67	72.28	22.05	13.96	9.40	97.82	92.96	89.31	85.21

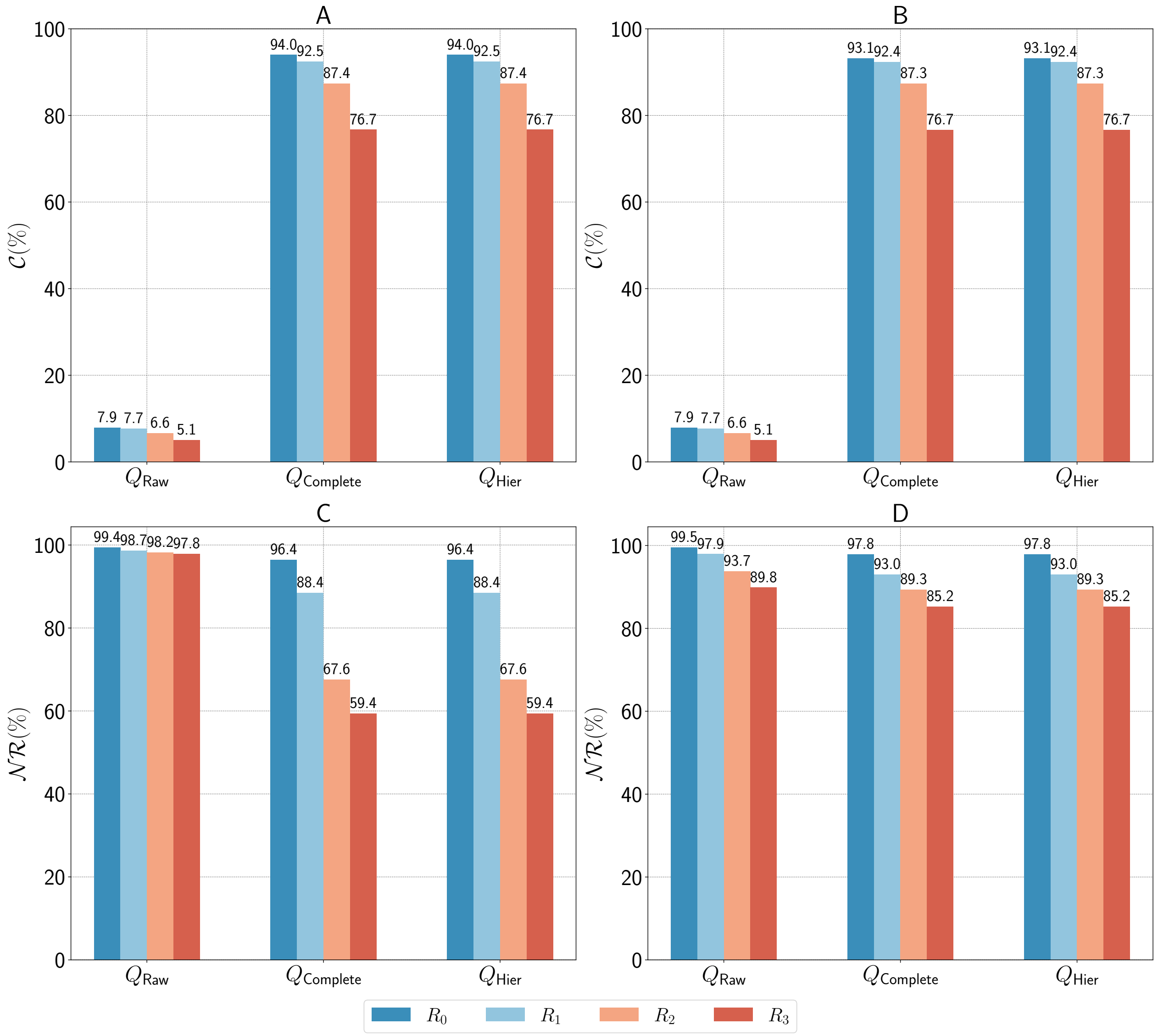

Figure S9: Performance of rule applications on the tes set of three different template types (Q_{raw}, Q_{complete}, Q_{hier}) across varying radii. Panels (A) and (B) depict the $\mathcal{C}\%$ for forward and backward predictions, respectively. Panels (C) and (D) illustrate the $\mathcal{NR}\%$ for forward and backward predictions.

Table S9: Comparative benchmarking of rule application processing times across various radii for validation and testing set

Type	Validation Hours				Test Hours			
	R_0	R_1	R_2	R_3	R_0	R_1	R_2	R_3
Q_{raw}	4.18	8.93	42.79	119.58	3.59	6.9	37.74	116.71
Q_{complete}	2.80	5.75	44.53	106.67	1.89	5.56	53.83	114.32
Q_{hier}	2.14	2.19	3.11	3.41	2.16	1.98	2.99	3.32

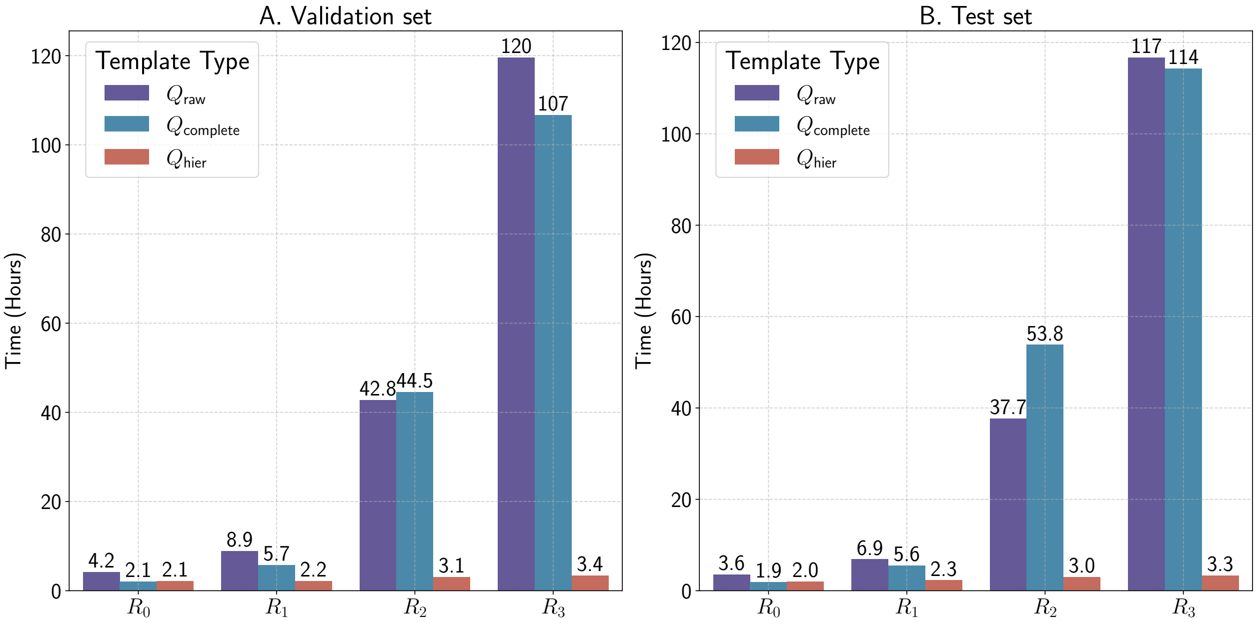

Figure S10: Processing time for each template type across various radii for (A) validation and (B) test set.

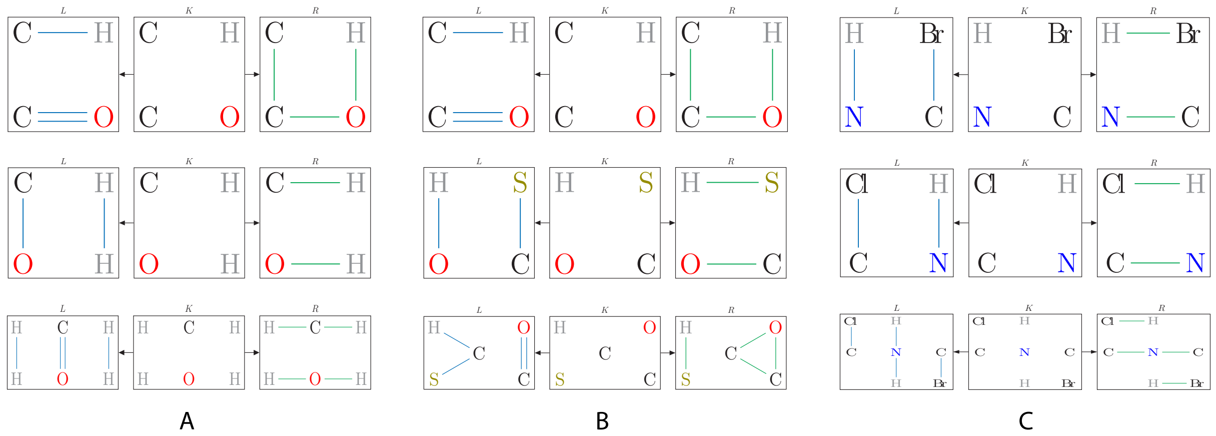

Figure S11: Illustrations of rule compositions in chemical processes: (A) *Rule 36*, resulting from the combination of *Rule 23* and *Rule 58*; (B) *Rule 170*, formed by the integration of *Rule 42*; and (C) *Rule 99* and *Rule 238*, derived from the combination of *Rule 0* and *Rule 2*.

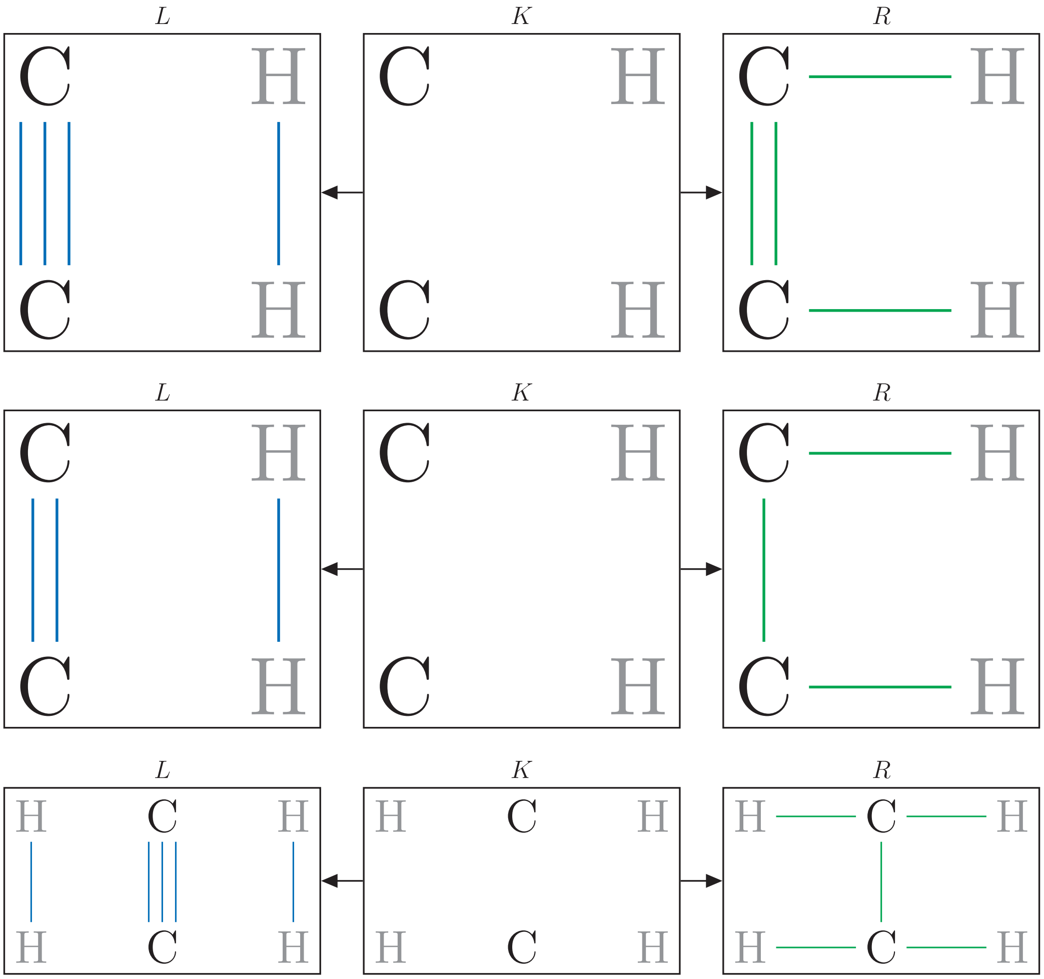

Figure S12: Illustration of rule Composition in Hydrogenation reactions. (A) DPO rule for hydrogenating an alkyne to an alkene. (B) DPO rule for hydrogenating an alkene to an alkane. (C) The resulting composed rule for hydrogenating an alkyne directly to an alkane.[1]

References

(1) Laffitte, M. E. G.; Beier, N.; Domschke, N.; Stadler, P. F. Comparison of atom maps. *MATCH: Comm. Math. Comp. Chem* **2023**, *90*, 75–102.

(2) González Laffitte, M. E.; Weibauer, K.; Phan, T.-L.; Beier, N.; Domschke, N.; Flamm, C.; Gatter, T.; Merkle, D.; Stadler, P. F. Partial Imaginary Transition State (ITS) Graphs: A Formal Framework for Research and Analysis of Atom-to-Atom Maps of Unbalanced Chemical Reactions and Their Completions. *Symmetry* **2024**, *16*, 1217.

(3) Osório, N.; Vilaça, P.; Rocha, M. A critical evaluation of automatic atom mapping algorithms and tools. 11th International Conference on Practical Applications of Computational Biology & Bioinformatics. 2017; pp 257–264.

(4) Körner, R.; Apostolakis, J. Automatic determination of reaction mappings and reaction center information. 1. The imaginary transition state energy approach. *J Chem Inf Model* **2008**, *48*, 1181–1189.

(5) Chen, S.; An, S.; Babazade, R.; Jung, Y. Precise atom-to-atom mapping for organic reactions via human-in-the-loop machine learning. *Nature Communications* **2024**, *15*, 2250.

(6) Lin, A.; Dyubankova, N.; Madzhidov, T. I.; Nugmanov, R. I.; Verhoeven, J.; Gimadiev, T. R.; Afonina, V. A.; Ibragimova, Z.; Rakhimbekova, A.; Sidorov, P.; others Atom-to-atom mapping: a benchmarking study of popular mapping algorithms and consensus strategies. *Molecular Informatics* **2022**, *41*, 2100138.

(7) Martin, Y. C. Let's not forget tautomers. *Journal of computer-aided molecular design* **2009**, *23*, 693–704.

(8) Sayle, R. A. So you think you understand tautomerism? *Journal of Computer-Aided Molecular Design* **2010**, *24*, 485–496.

(9) Andersen, J. L.; Flamm, C.; Merkle, D.; Stadler, P. F. Inferring Chemical Reaction Patterns Using Graph Grammar Rule Composition. *J. Syst. Chem.* **2013**, *4*, 4.
