# Supplementary material for: SynTemp: Efficient Extraction of Graph-Based Reaction Rules from Large-Scale Reaction Databases
Source: J Chem Inf Model. 2025 Feb 28;65(6):2882–96. doi: 10.1021/acs.jcim.4c01795 (PMC11938280; doi:10.1021/acs.jcim.4c01795)

R-id = 43057 with reaction step = 3

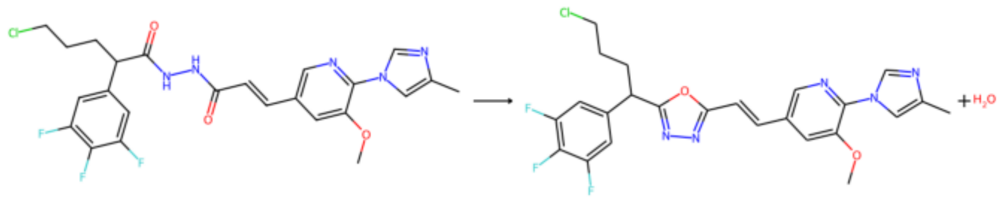

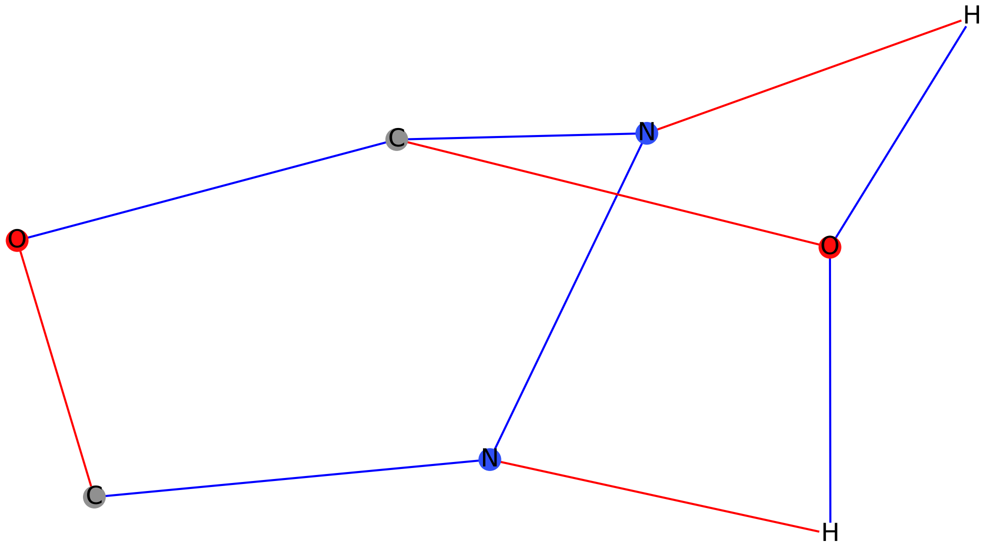

R-id = 11255 with reaction step = 1

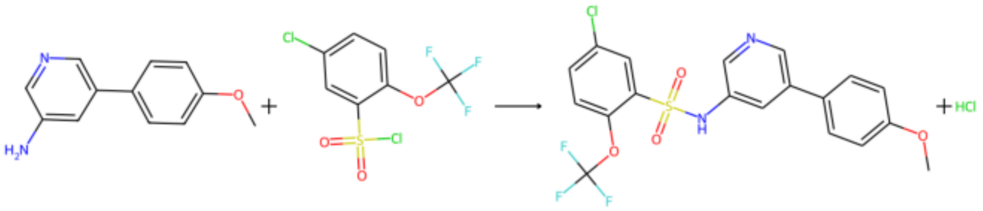


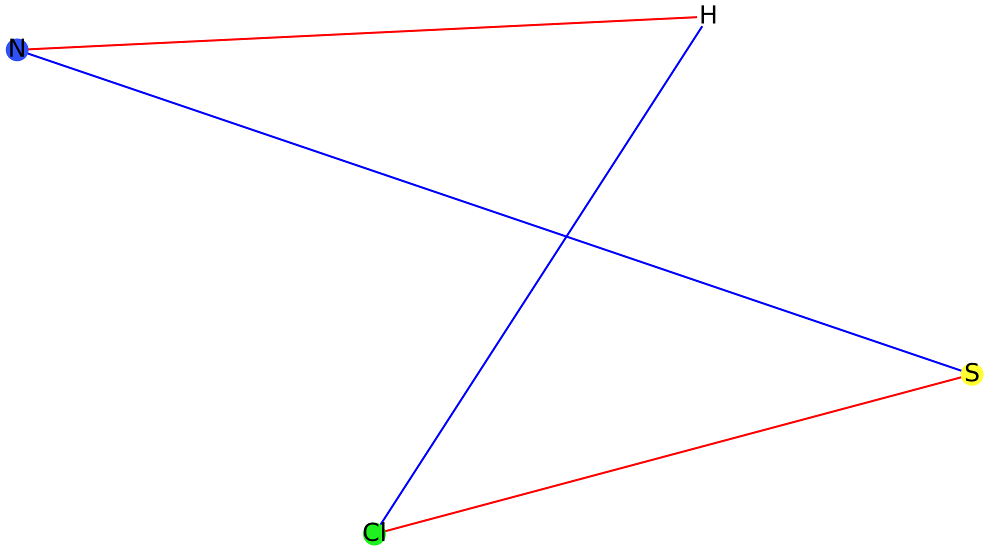

R-id = 44385 with reaction step = 4

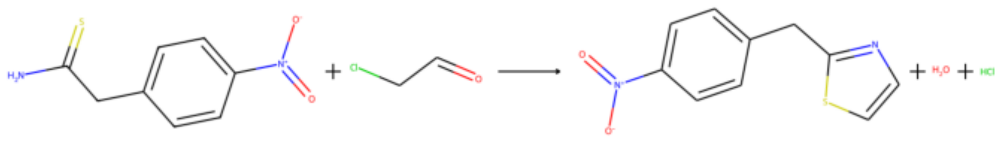


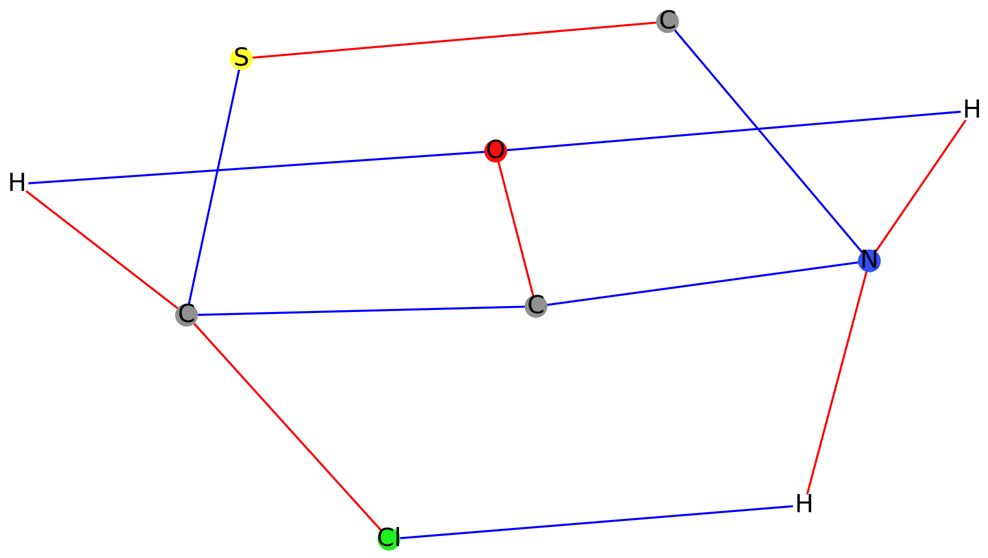

R-id = 49194 with reaction step = 1

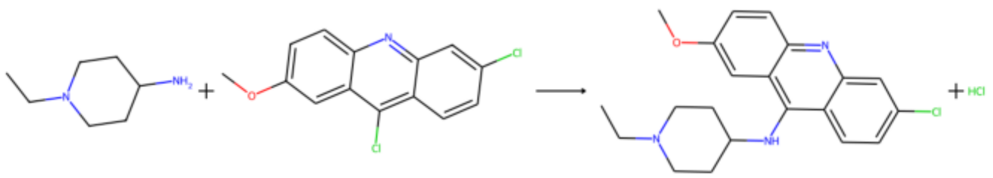


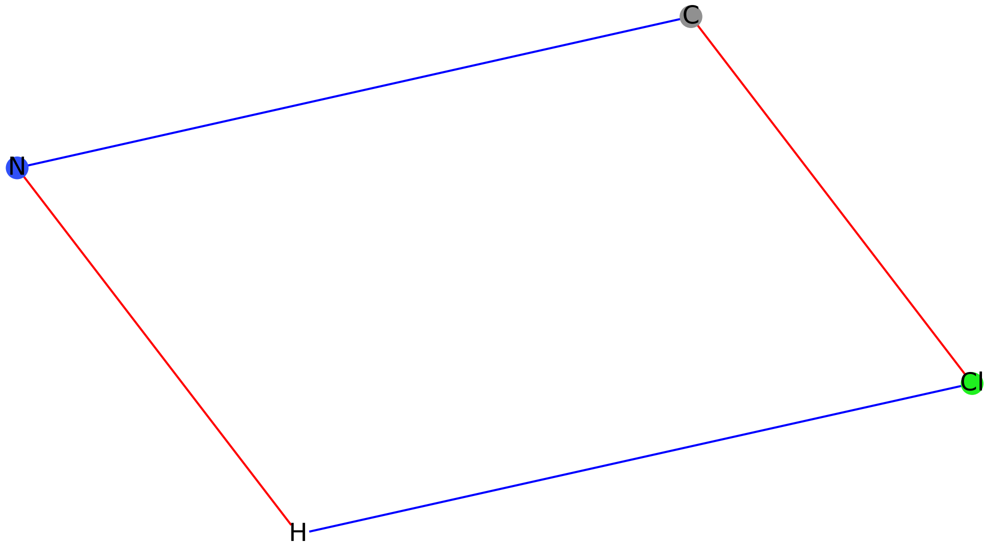

R-id = 13017 with reaction step = 1

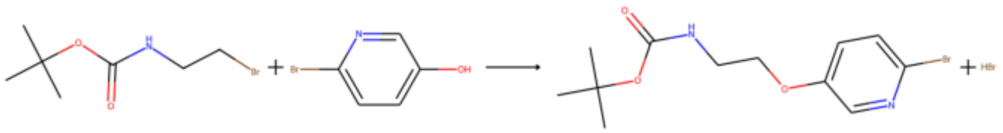


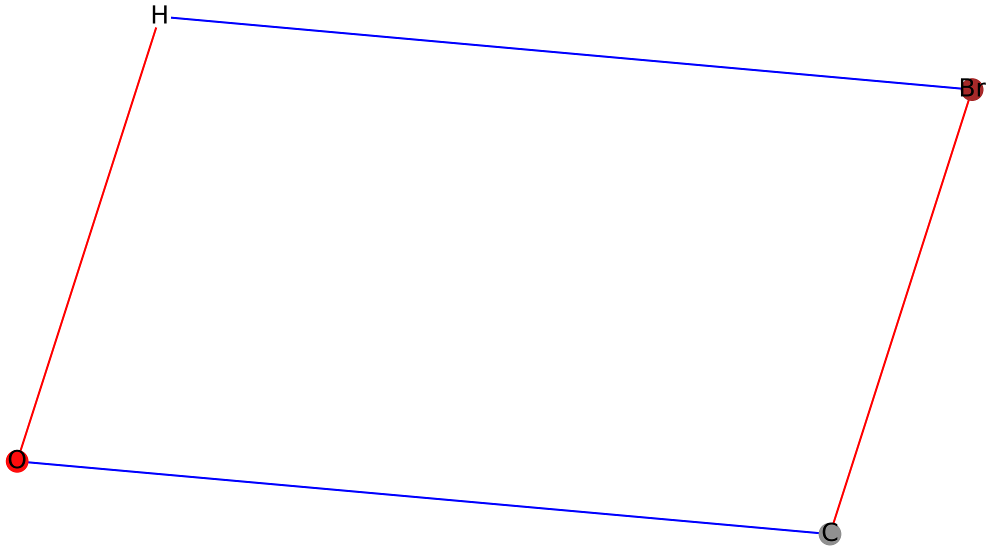

R-id = 6162 with reaction step = 2

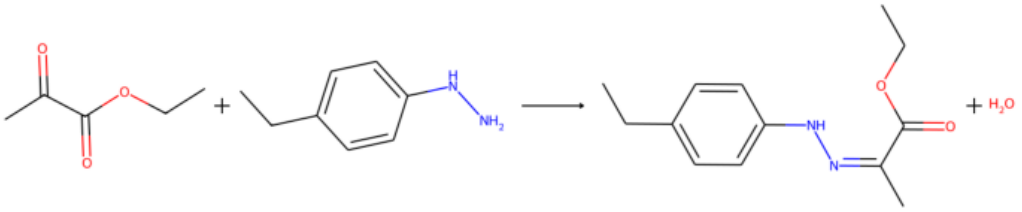


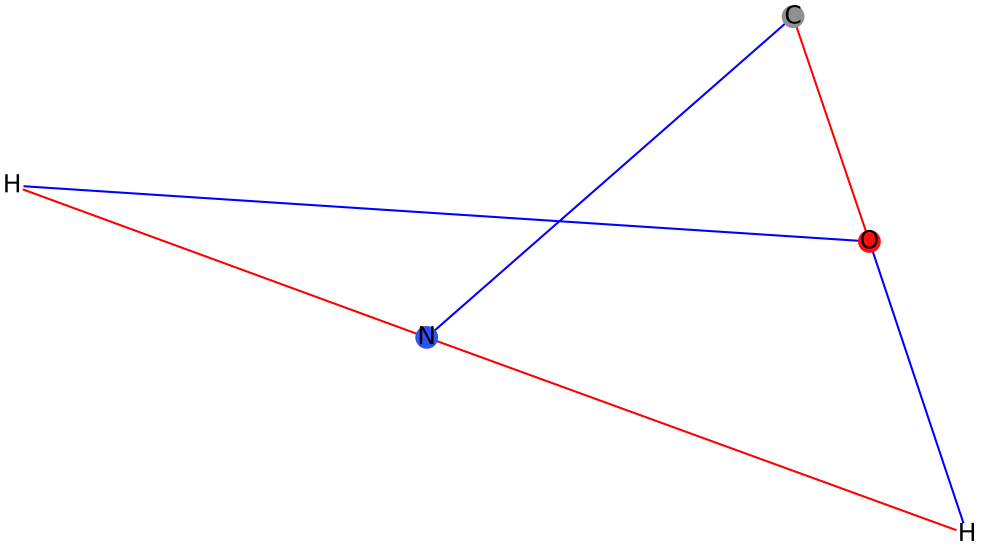

R-id = 3503 with reaction step = 1

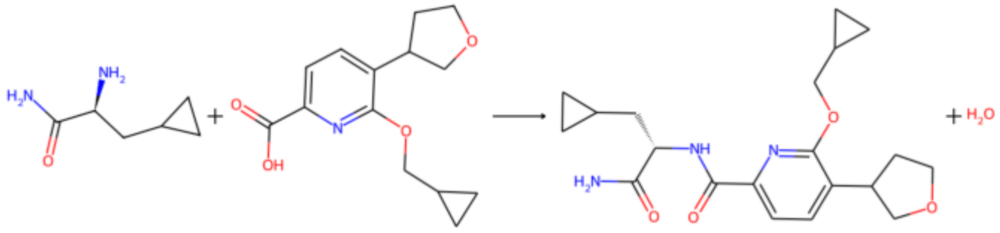


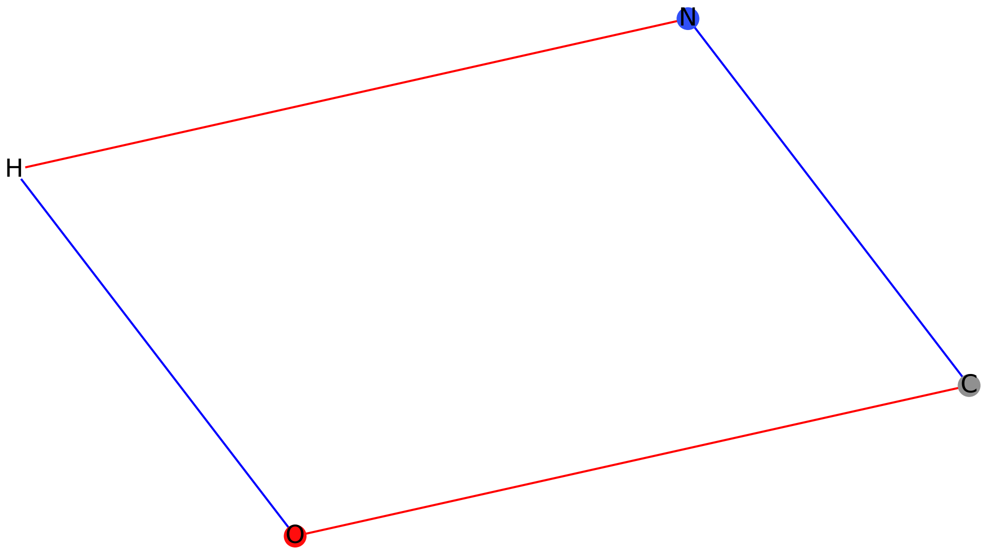

R-id = 14346 with reaction step = 1

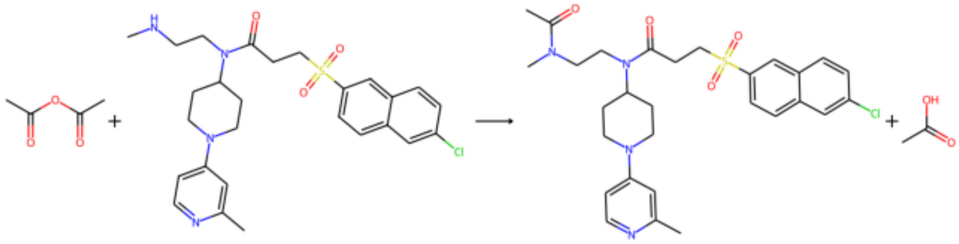


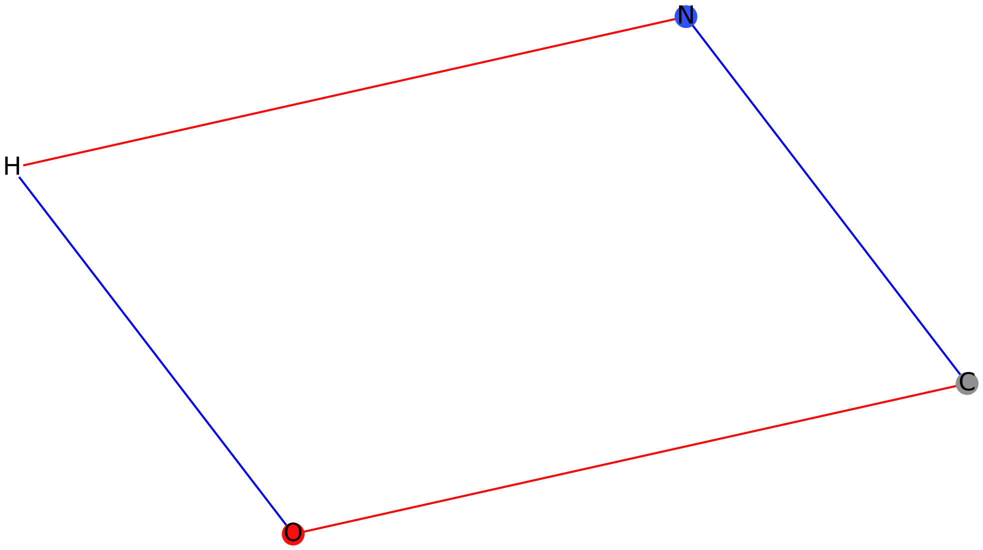

R-id = 42966 with reaction step = 1

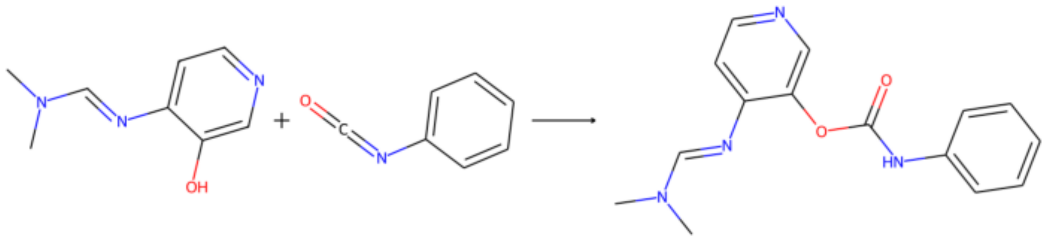

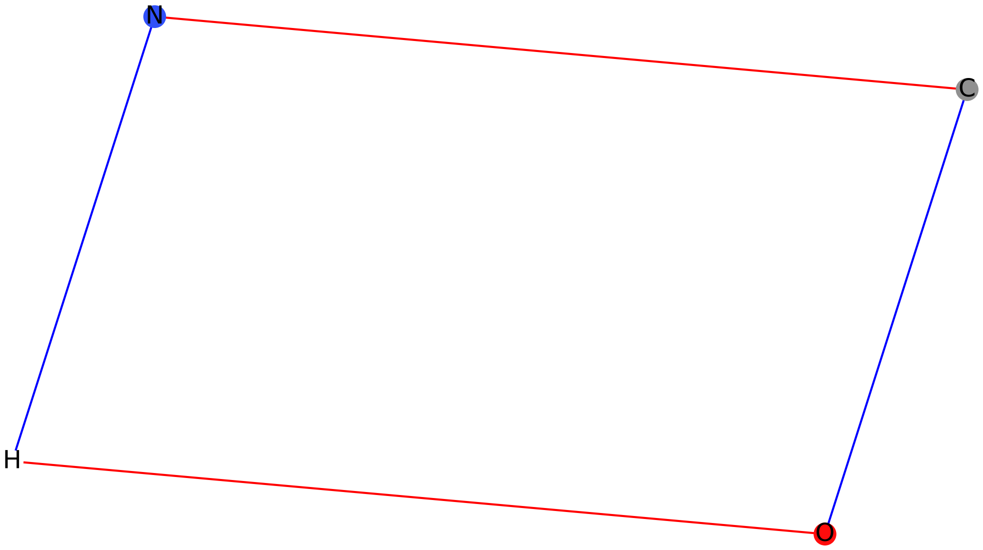

R-id = 5106 with reaction step = 1

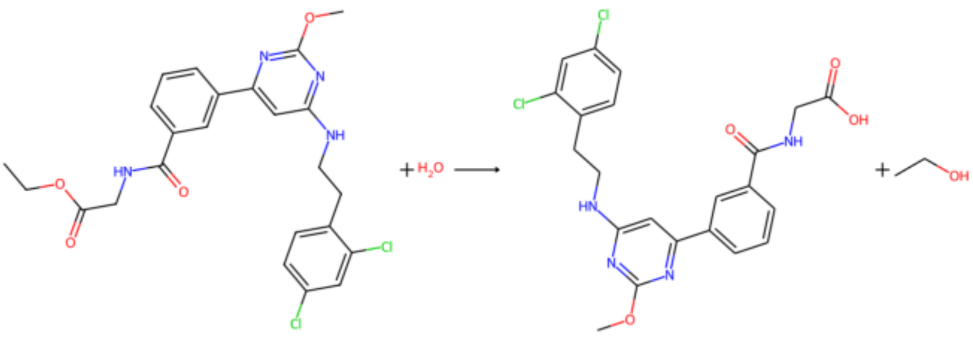


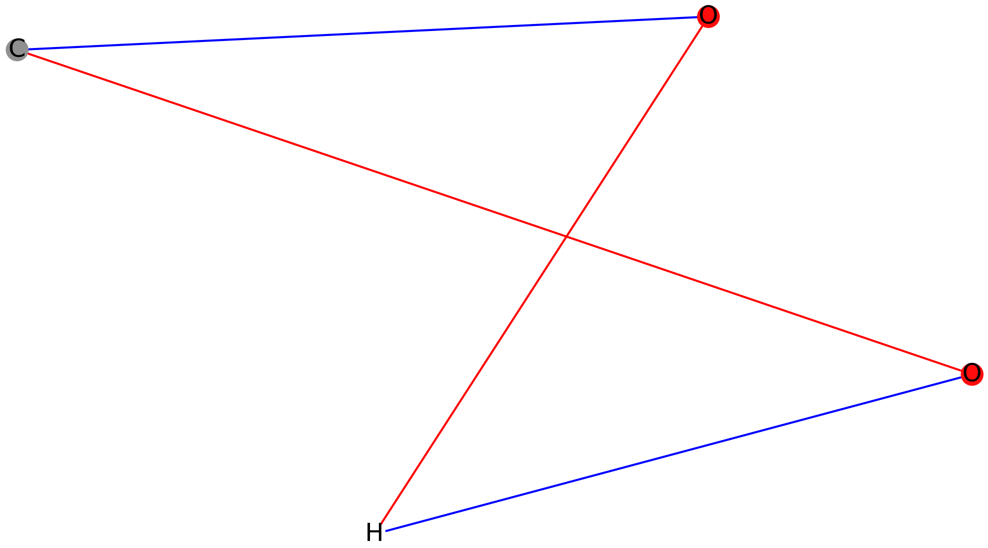

R-id = 49521 with reaction step = 4

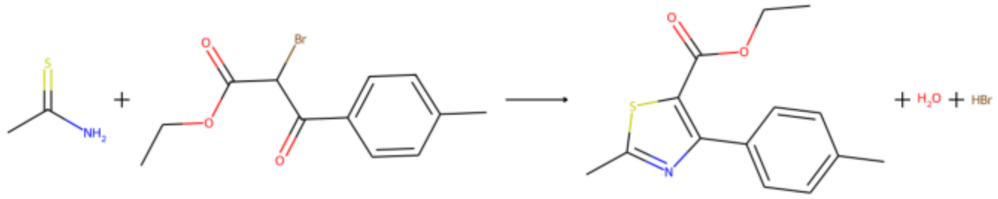


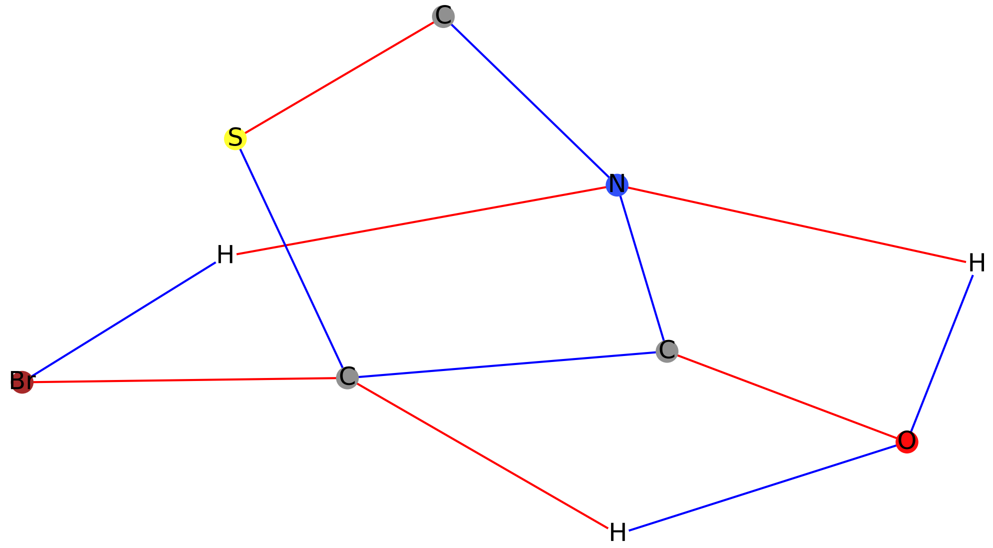

R-id = 30477 with reaction step = 2

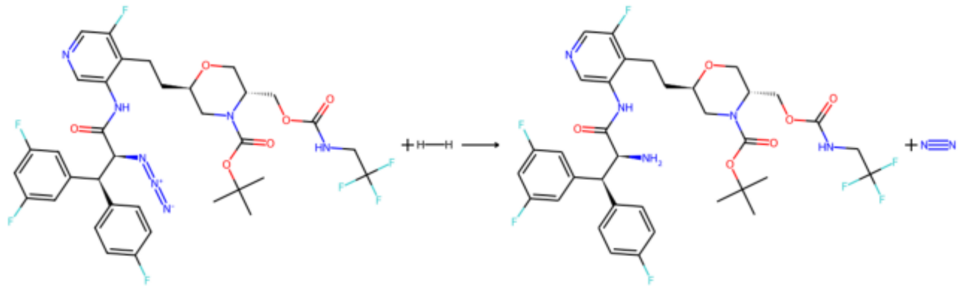

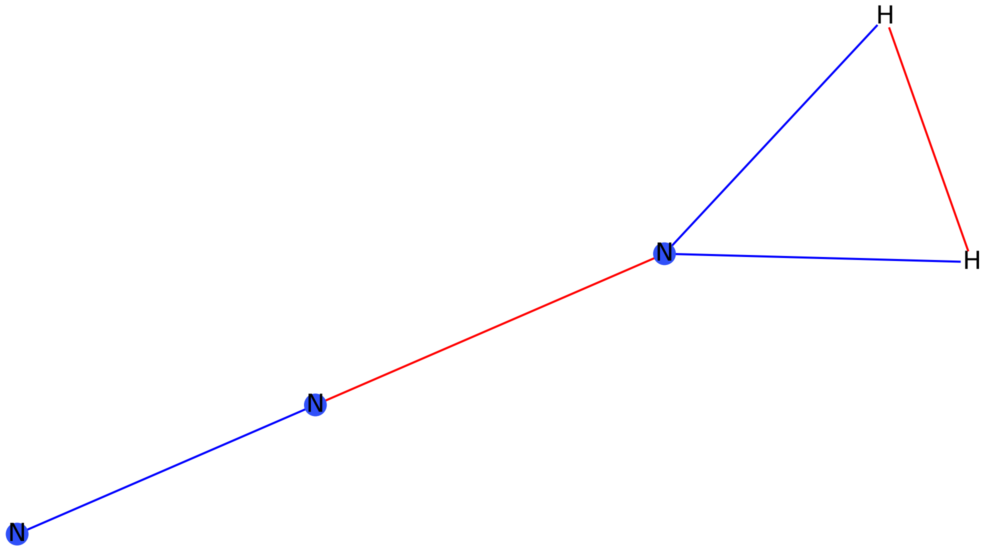

R-id = 1735 with reaction step = 2

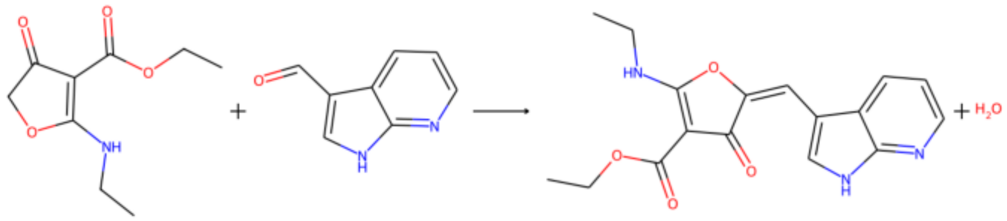


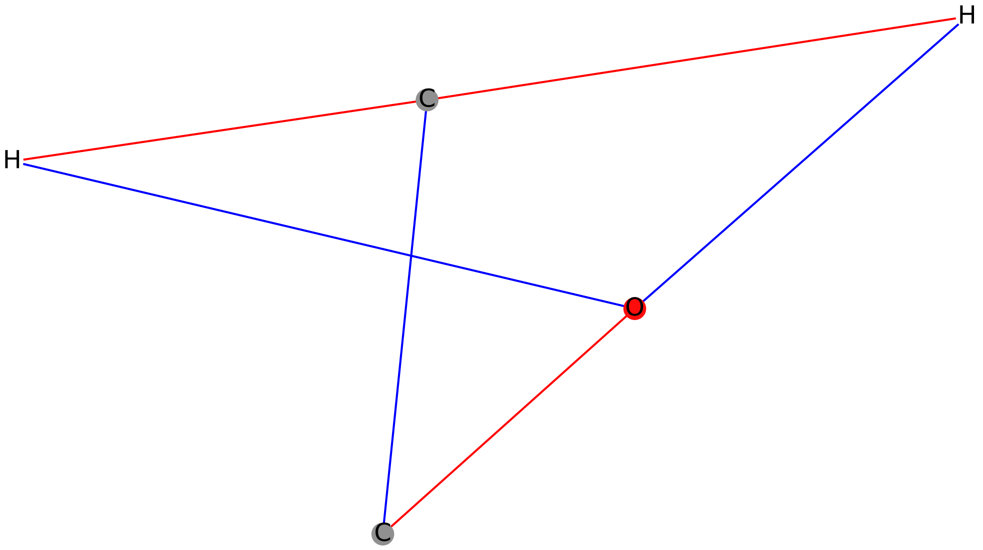

R-id = 6125 with reaction step = 4

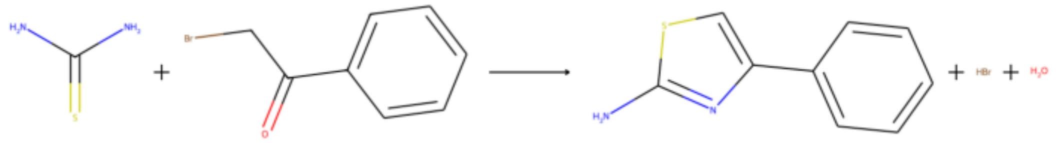


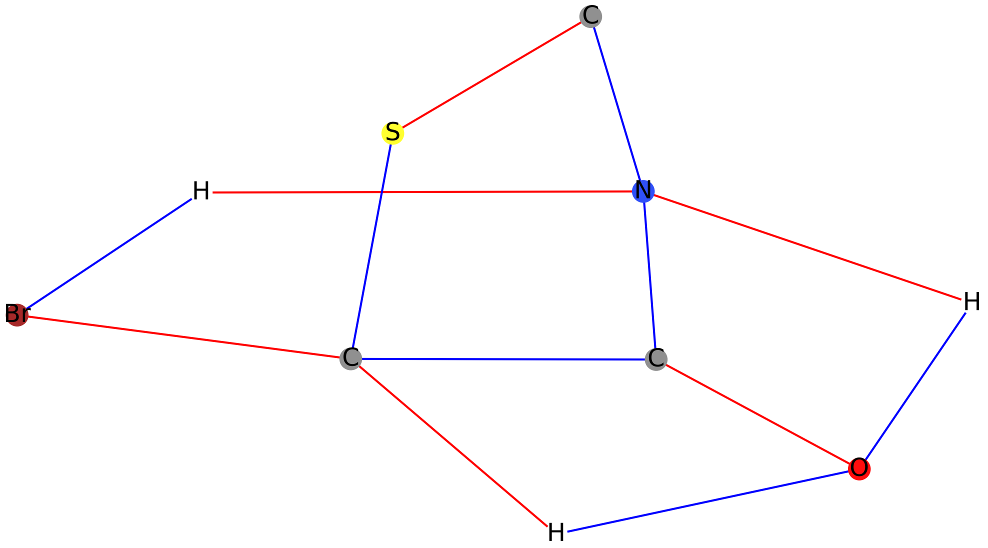

R-id = 38030 with reaction step = 4

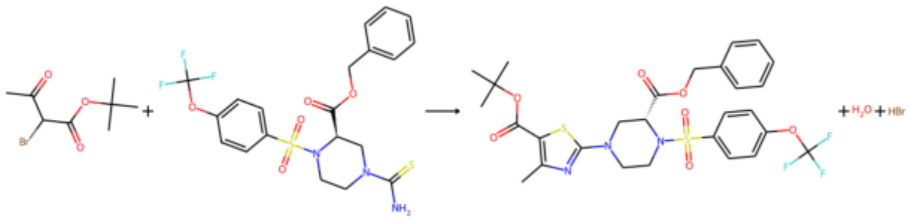

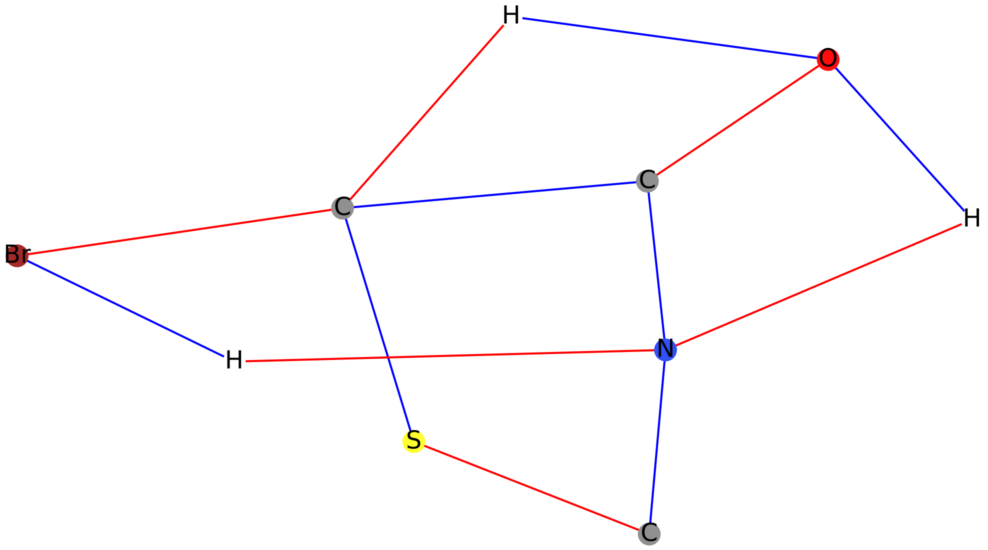

R-id = 13067 with reaction step = 3

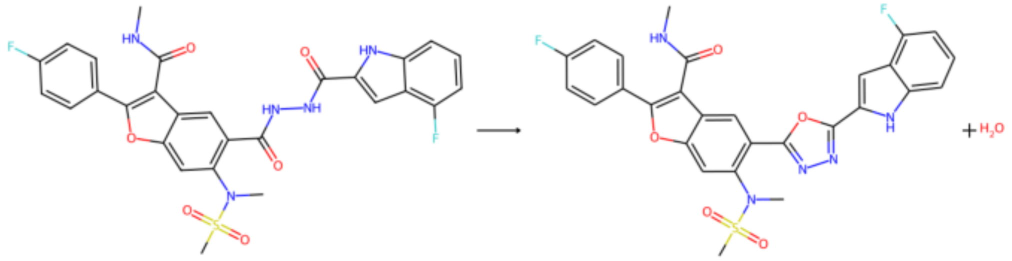

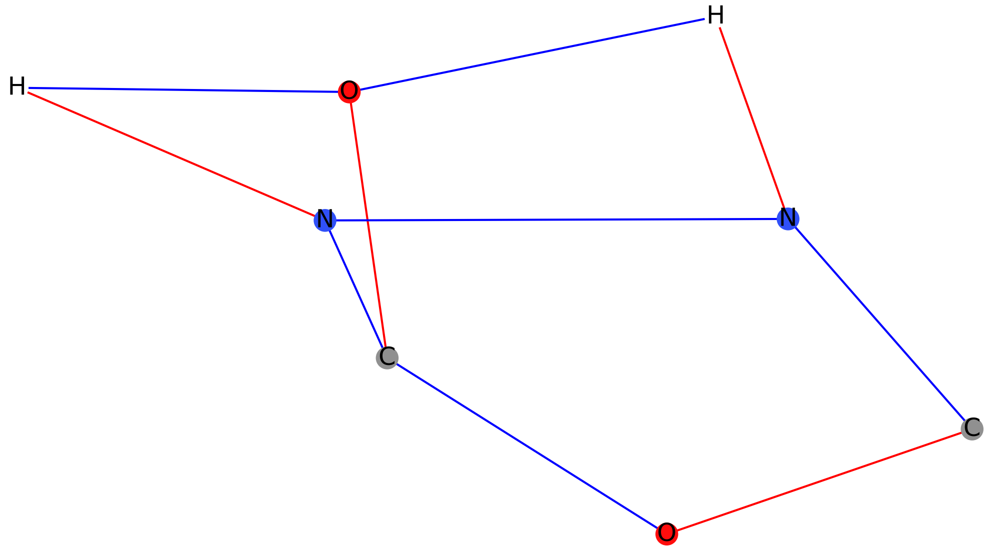

R-id = 40364 with reaction step = 3

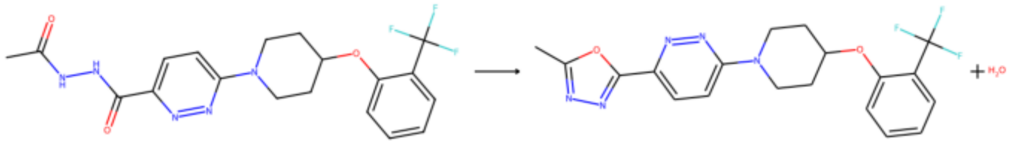


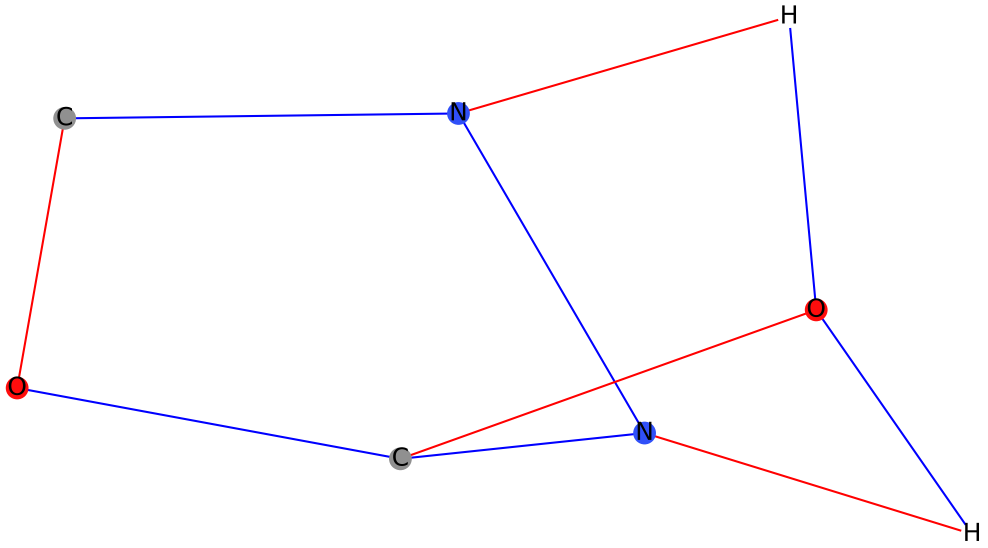

R-id = 28971 with reaction step = 1

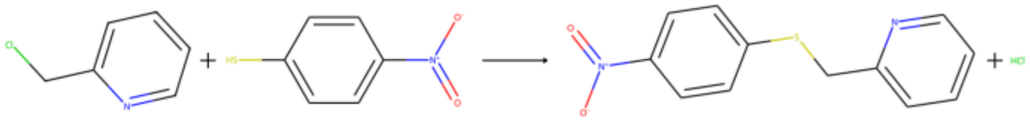

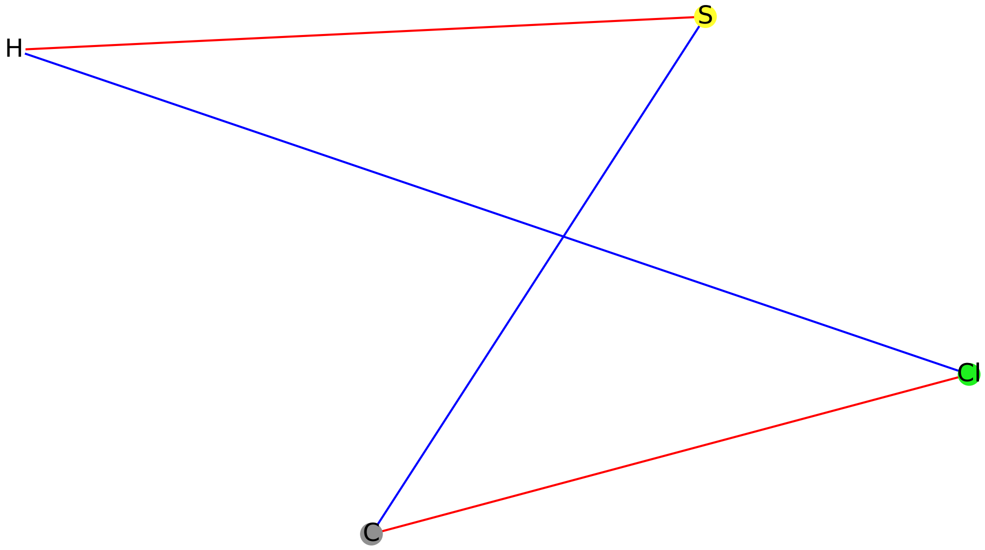

R-id = 26895 with reaction step = 2

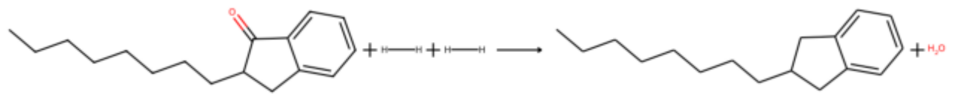

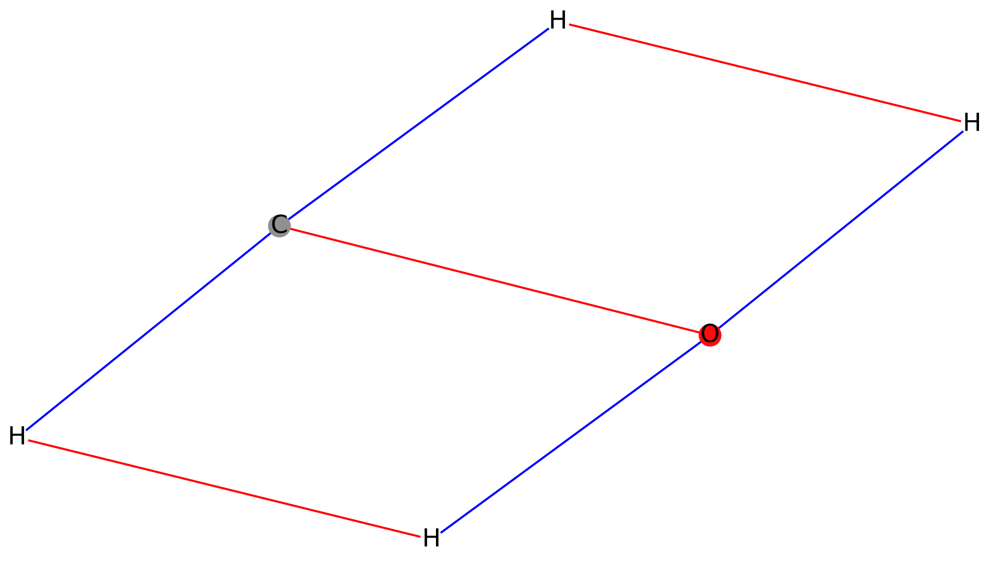

R-id = 32999 with reaction step = 4

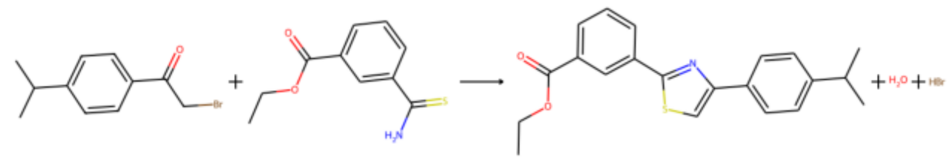

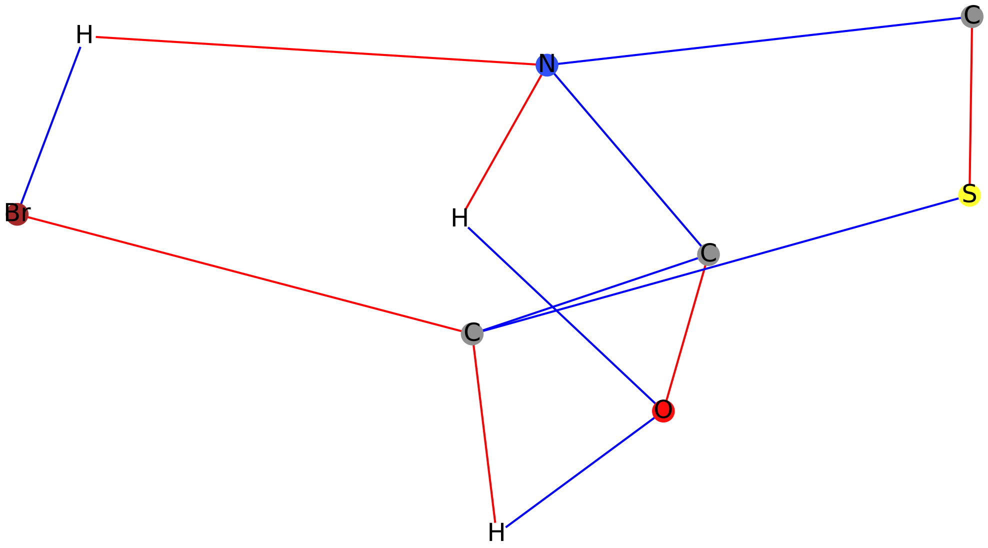

R-id = 5626 with reaction step = 1

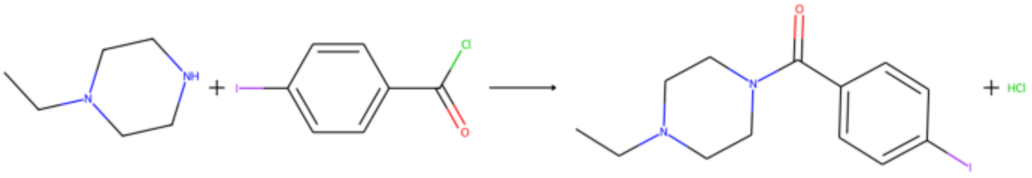


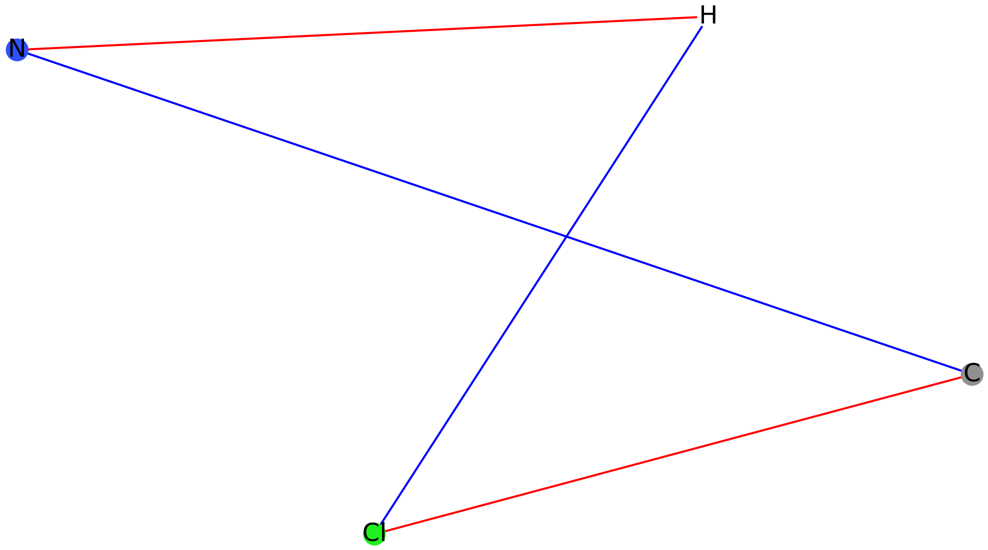

R-id = 21867 with reaction step = 1

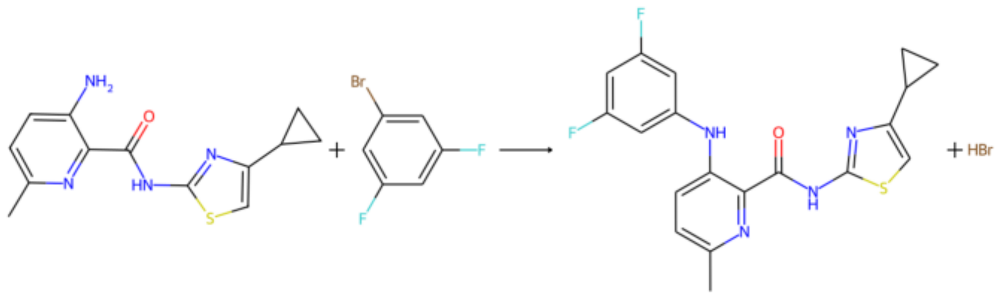

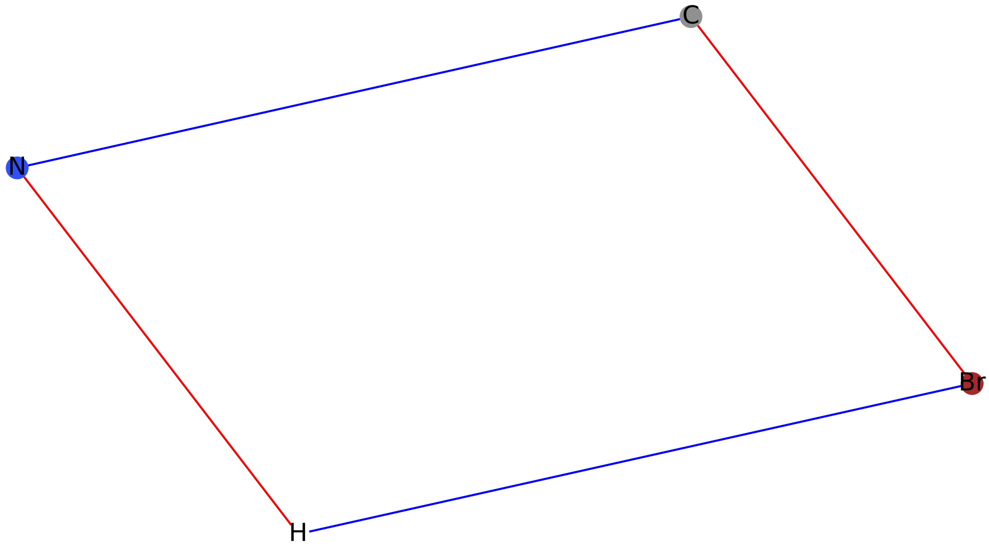

R-id = 3081 with reaction step = 3

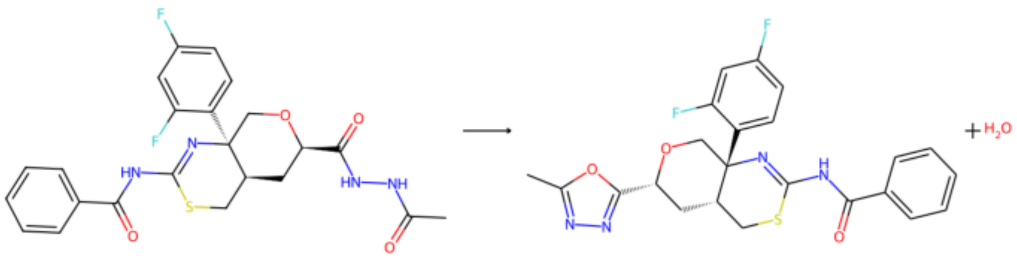


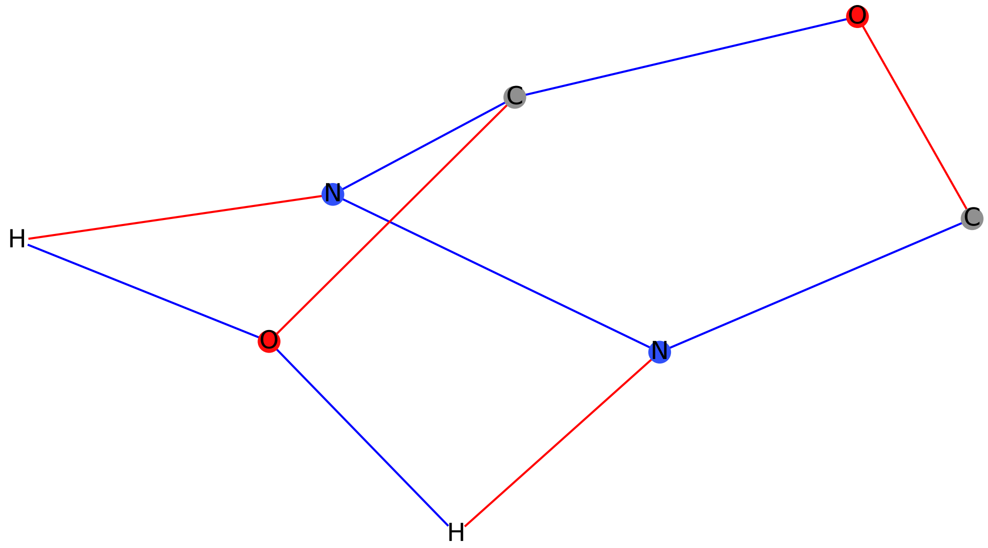

R-id = 13997 with reaction step = 1

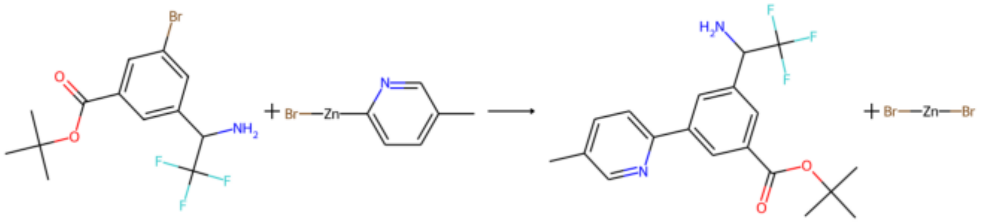


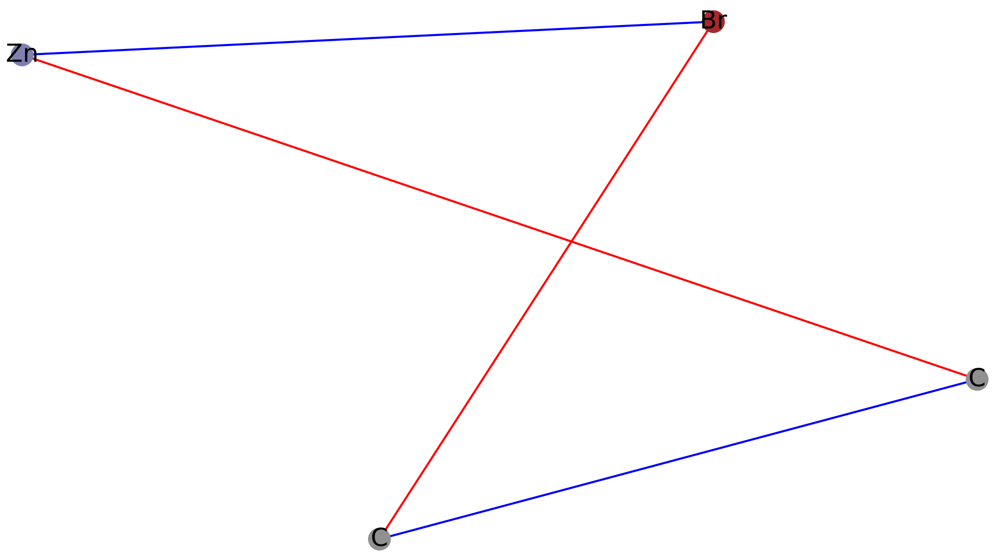

R-id = 6732 with reaction step = 4

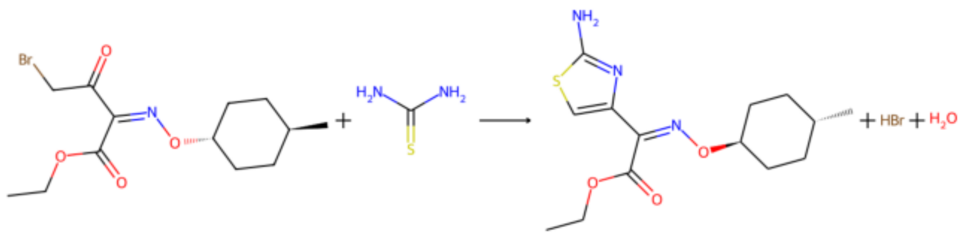


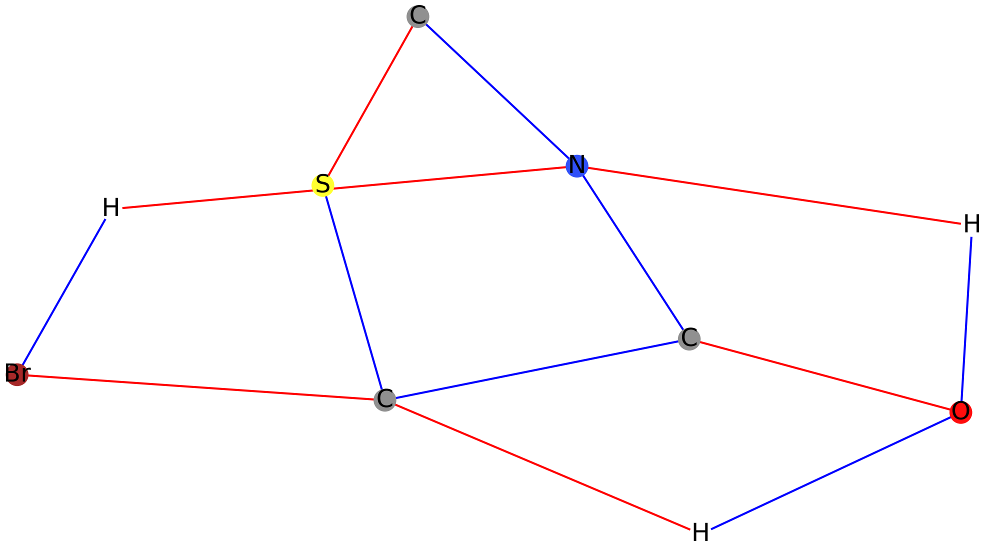

R-id = 40915 with reaction step = 2

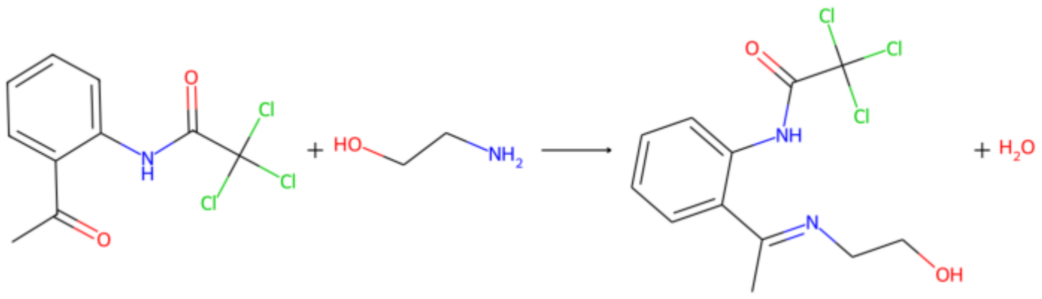


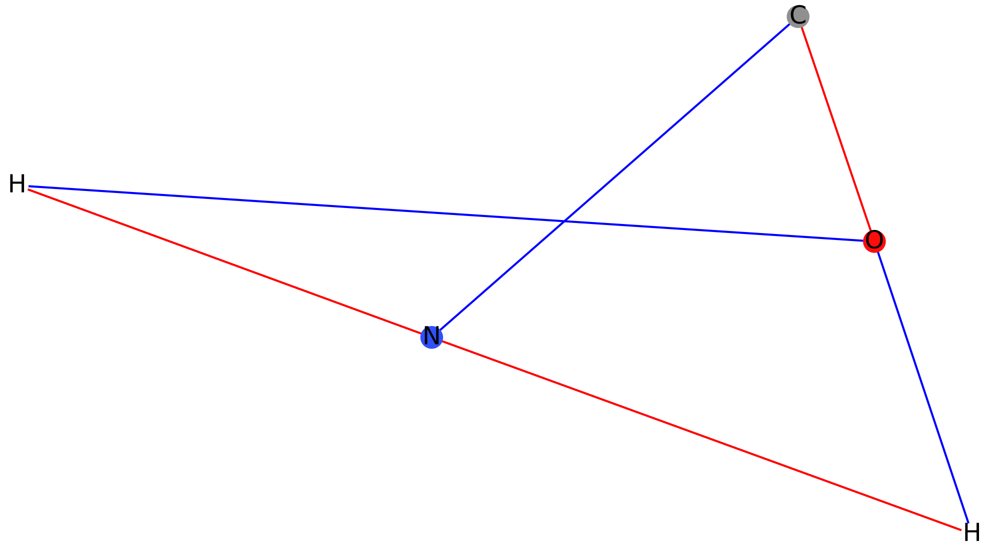

R-id = 45032 with reaction step = 1

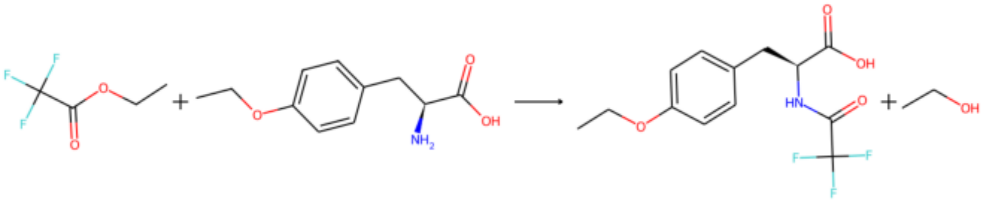


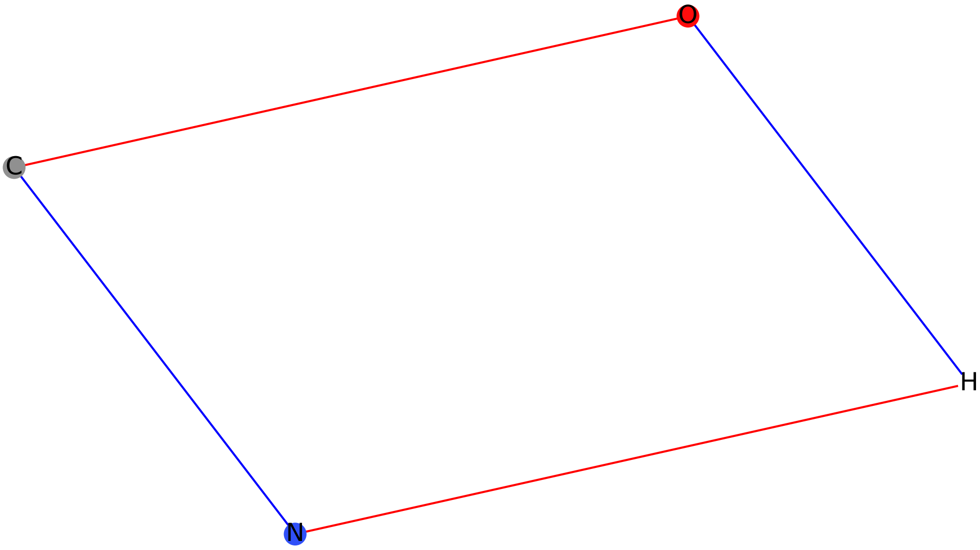

R-id = 39233 with reaction step = 1

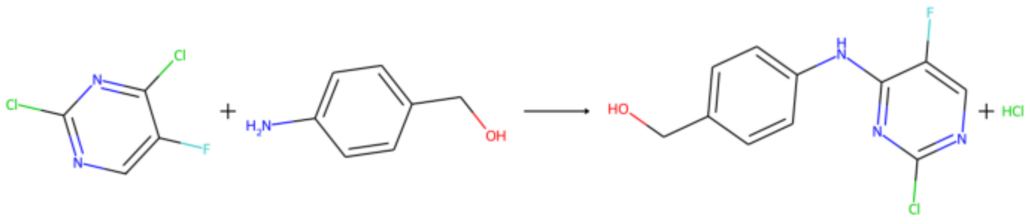


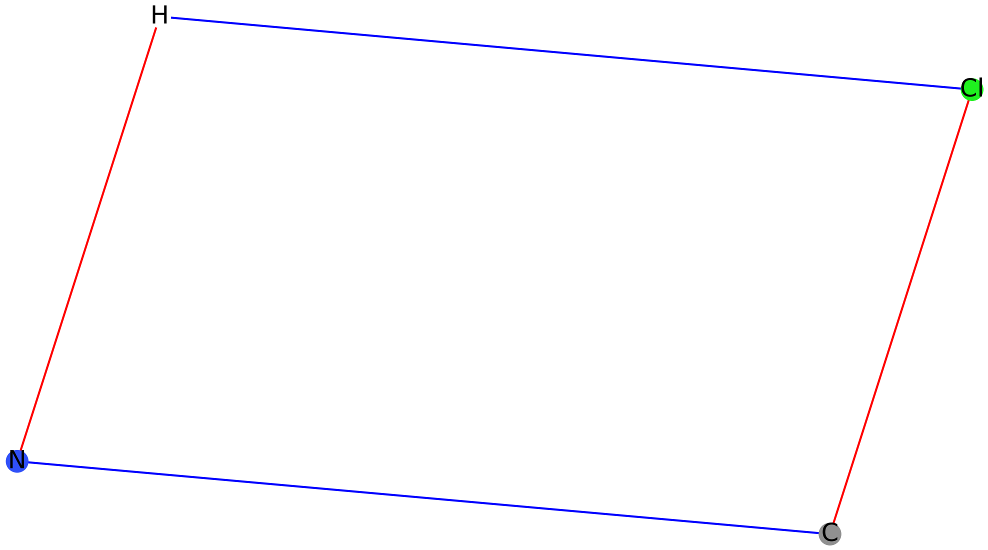

R-id = 15225 with reaction step = 2

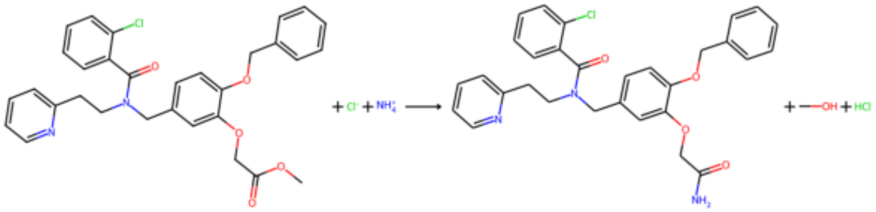


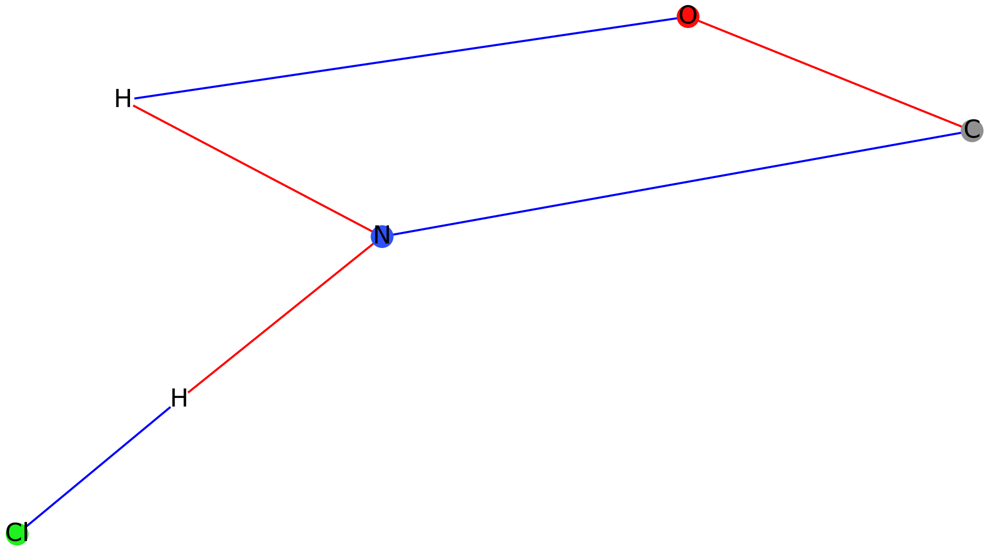

R-id = 18176 with reaction step = 1

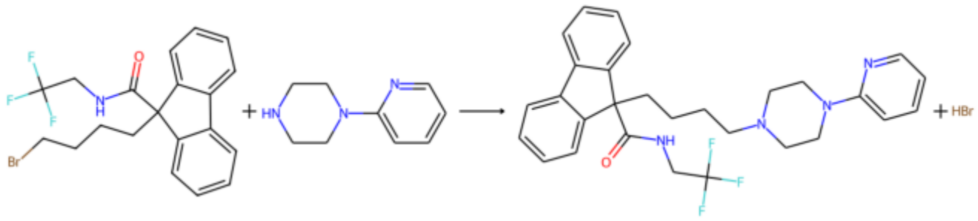


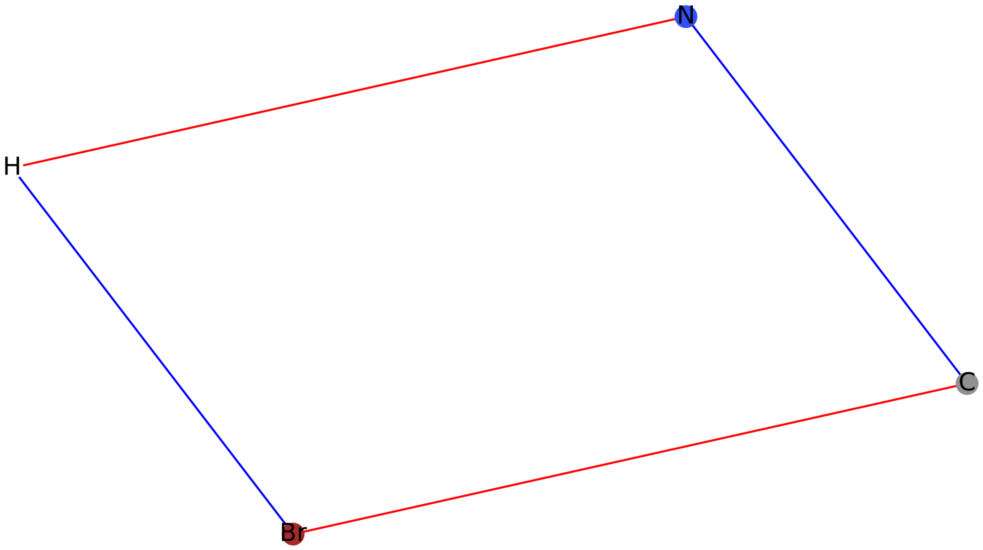

R-id = 18863 with reaction step = 4

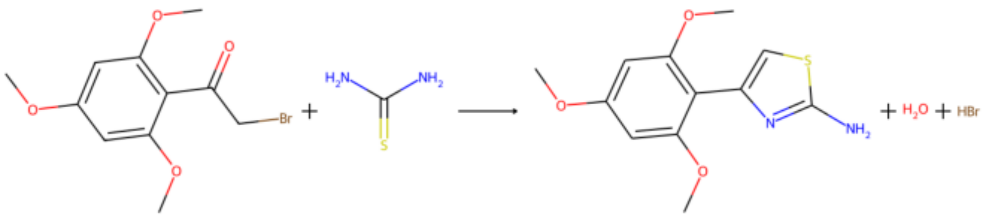


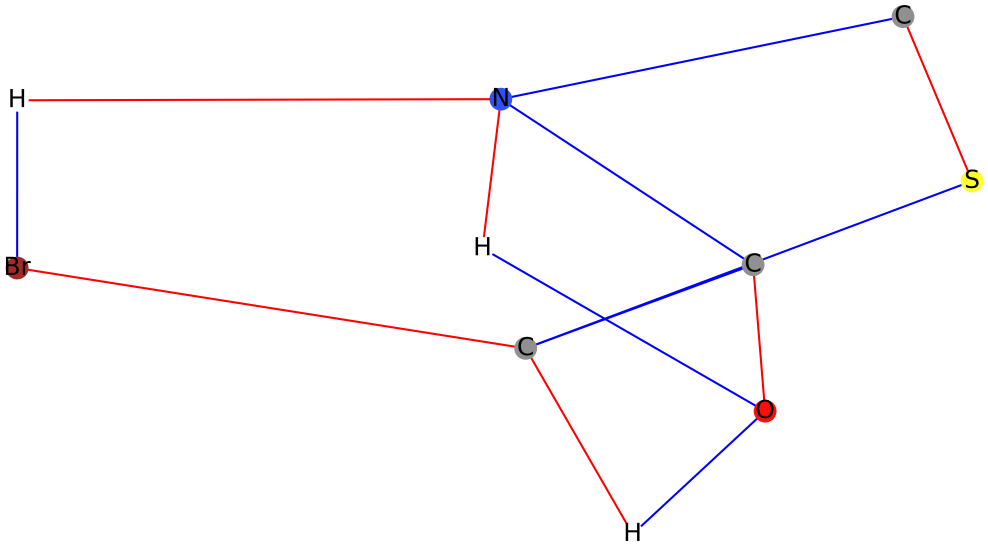

R-id = 32071 with reaction step = 1

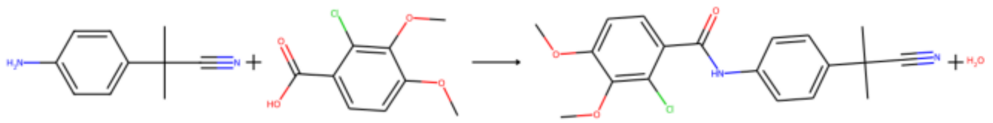


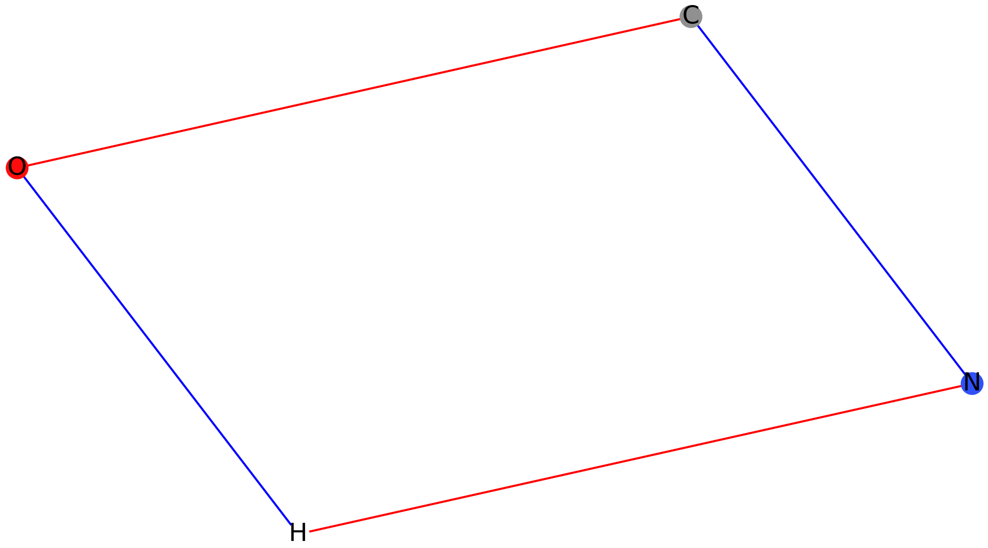

R-id = 9454 with reaction step = 1

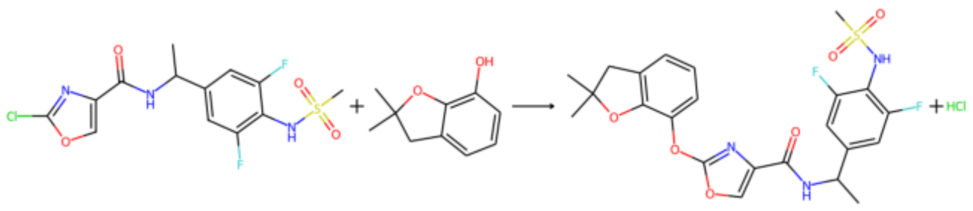

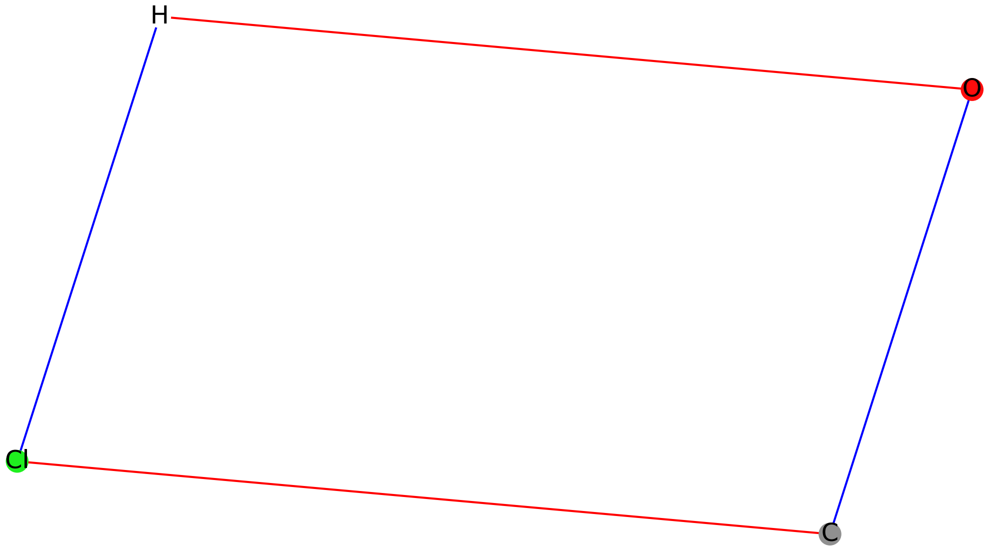

R-id = 22546 with reaction step = 1

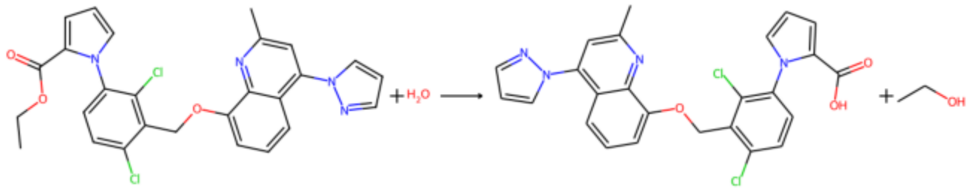

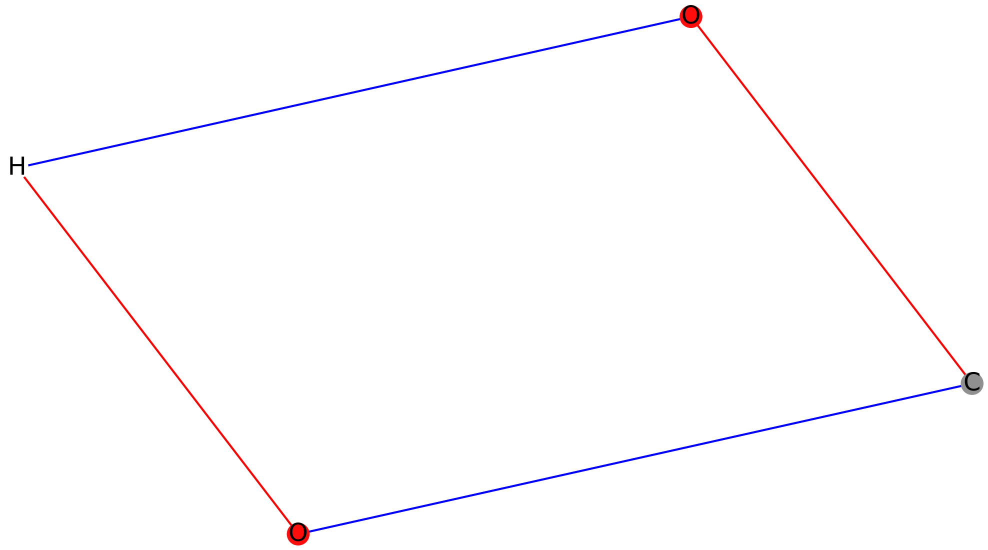

R-id = 20279 with reaction step = 3

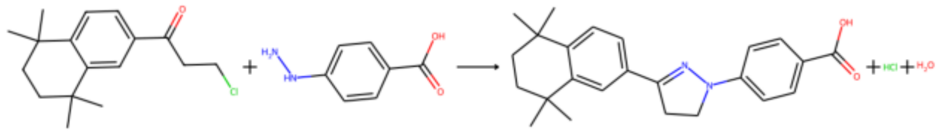


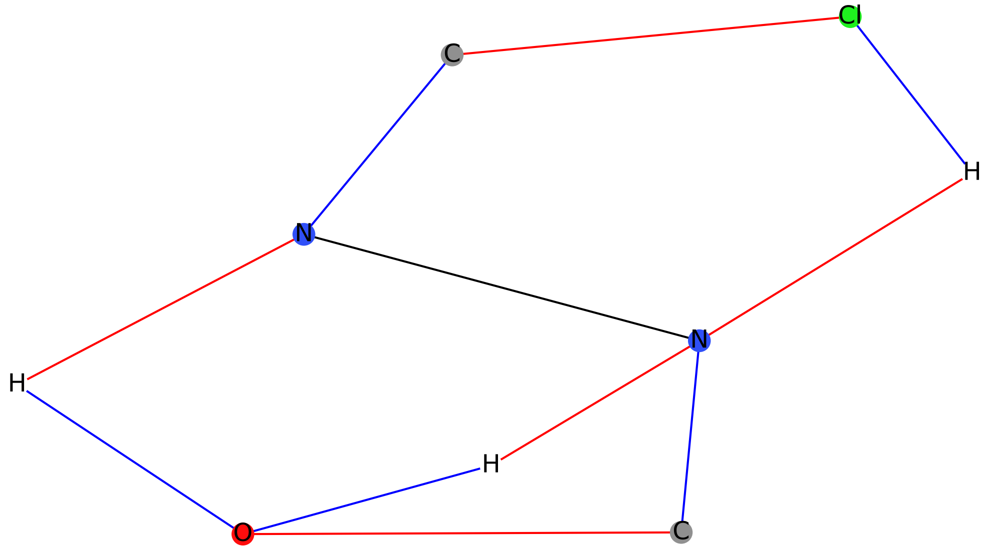

R-id = 1316 with reaction step = 2

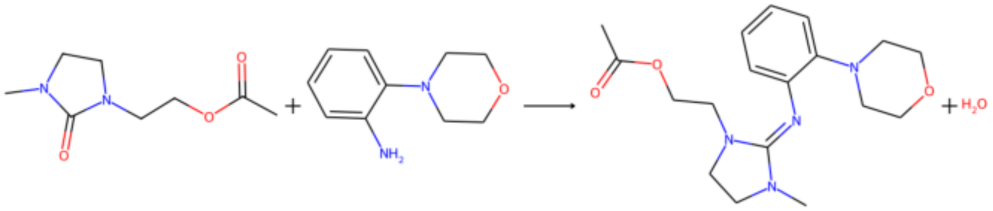

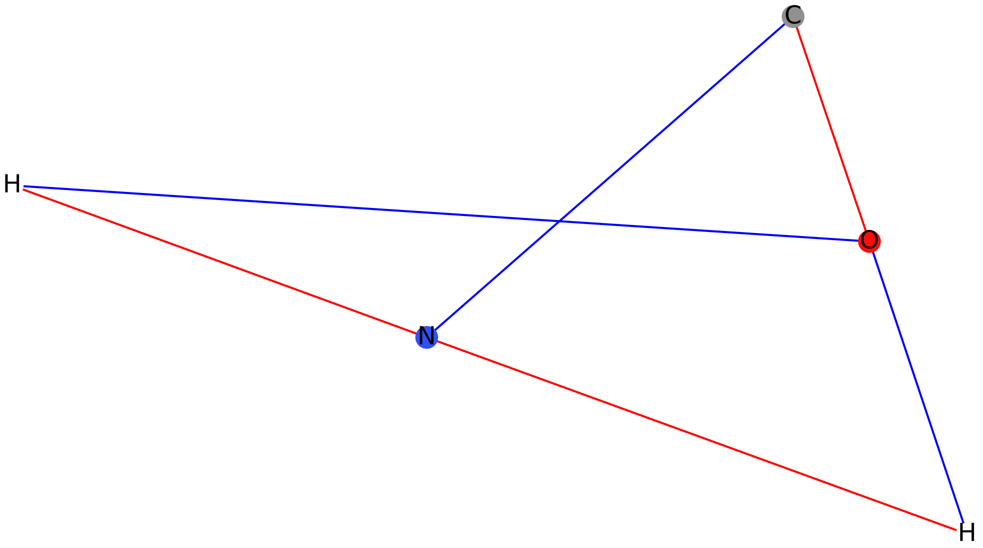

R-id = 17417 with reaction step = 1

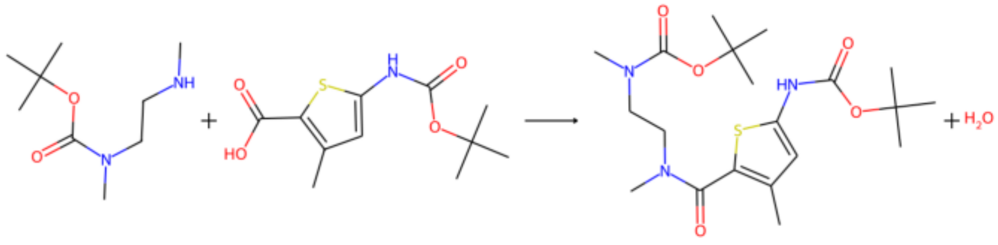

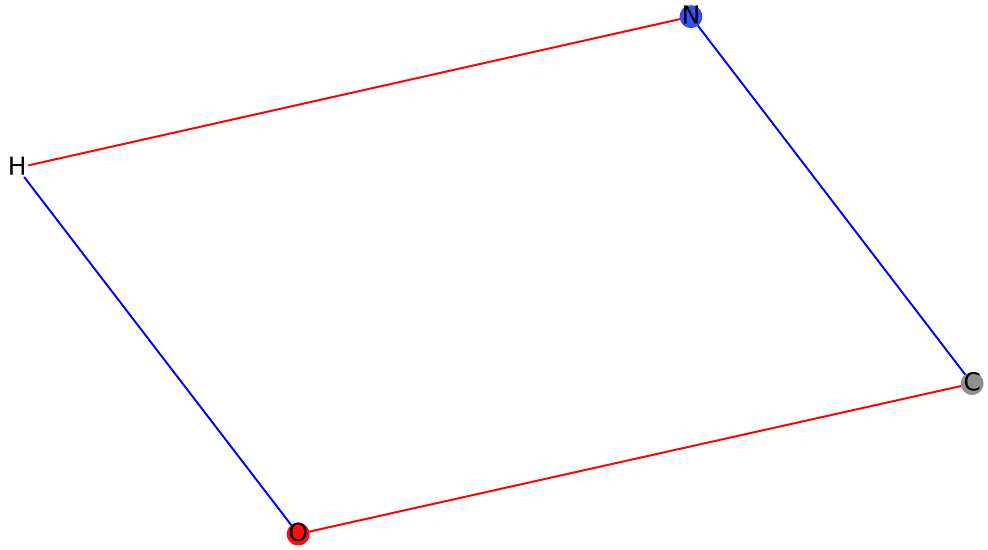

R-id = 36545 with reaction step = 2

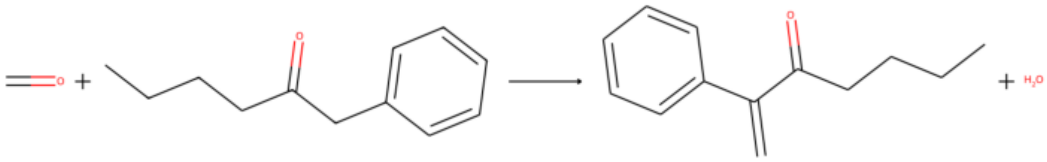


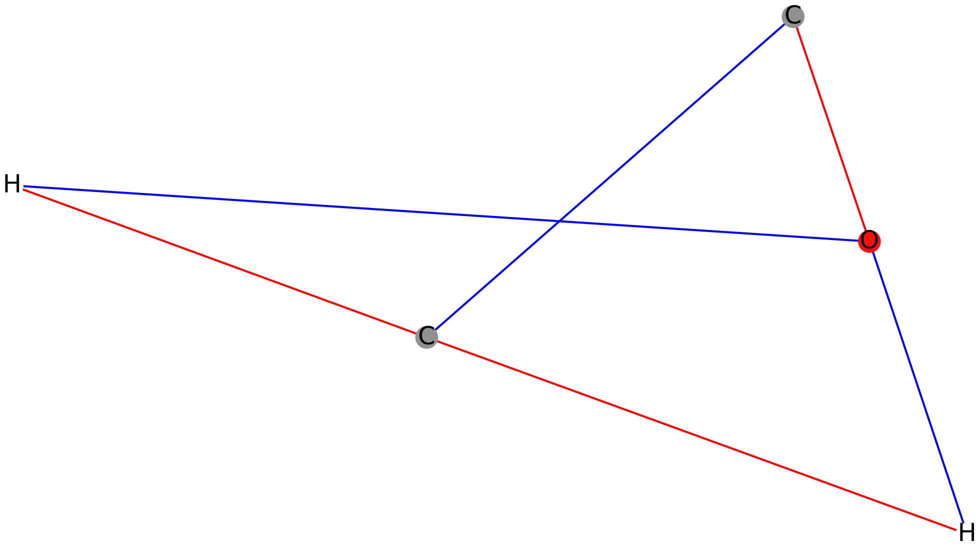

R-id = 32633 with reaction step = 1

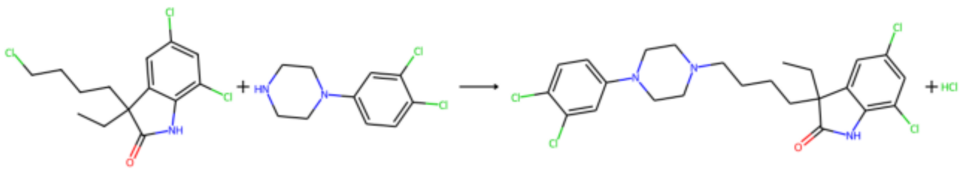


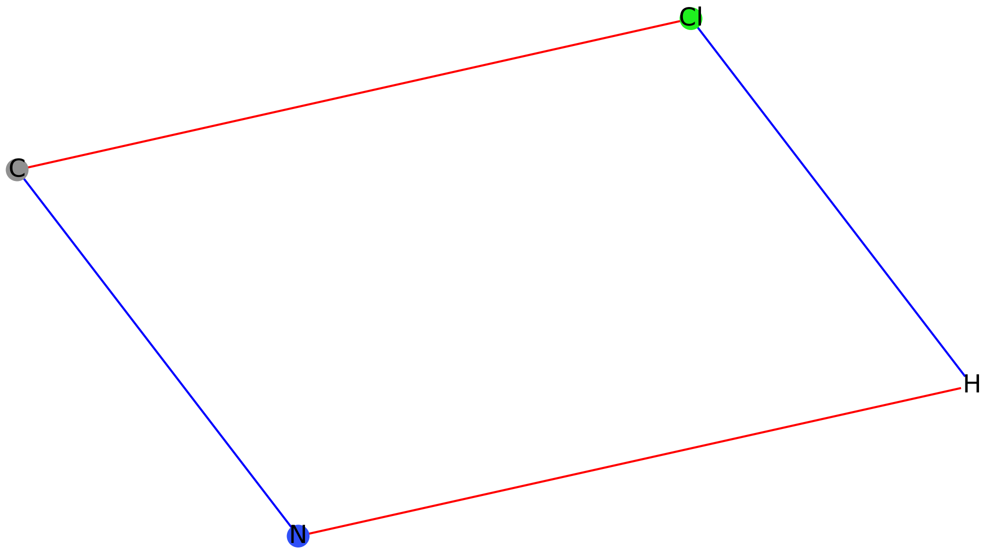

R-id = 25896 with reaction step = 1

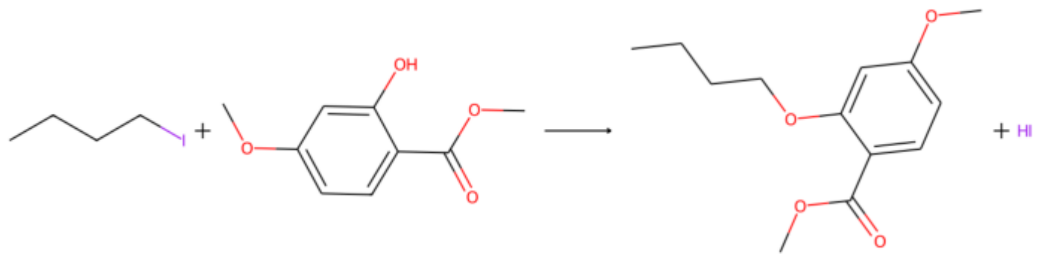


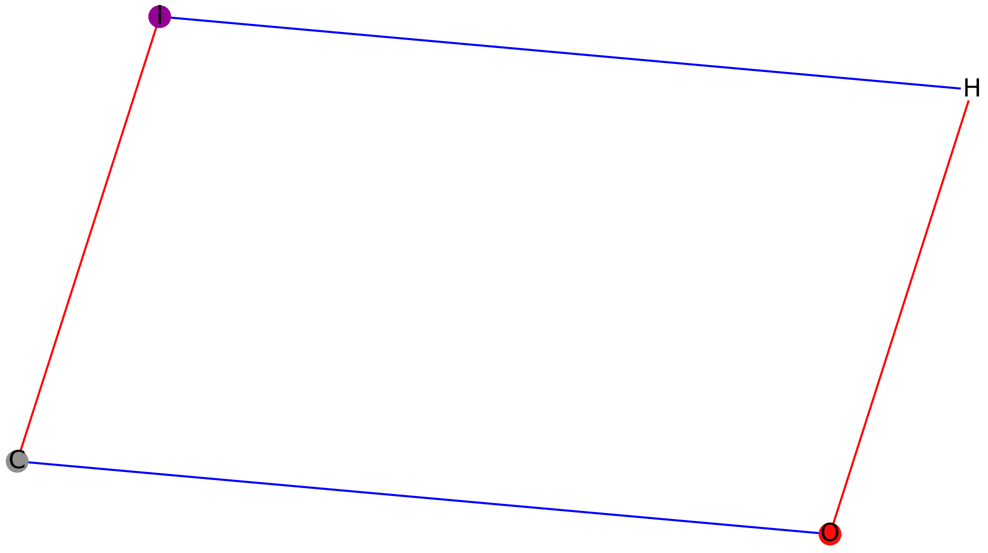

R-id = 49322 with reaction step = 3

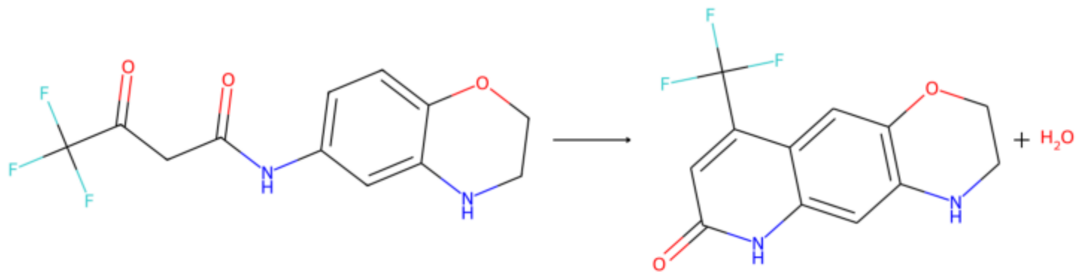


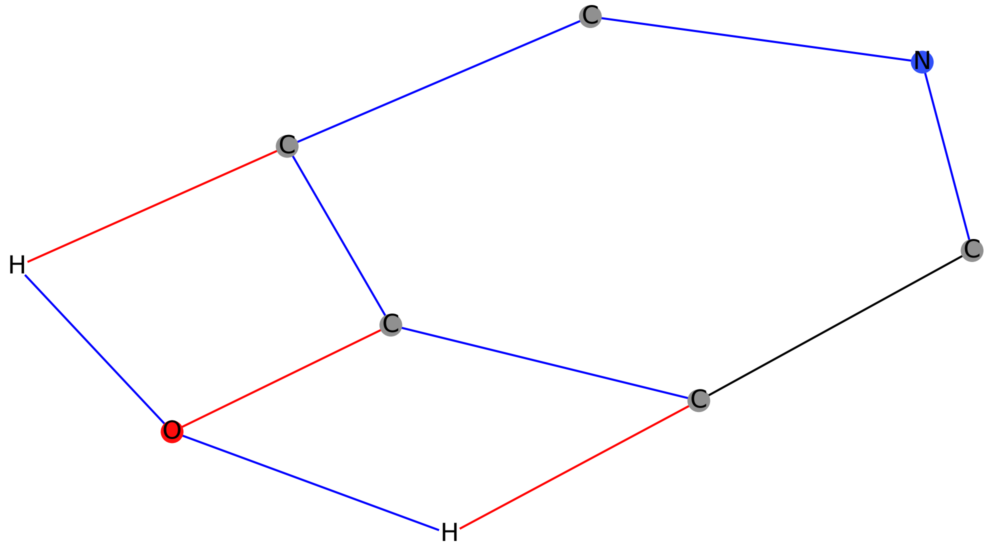

R-id = 37637 with reaction step = 3

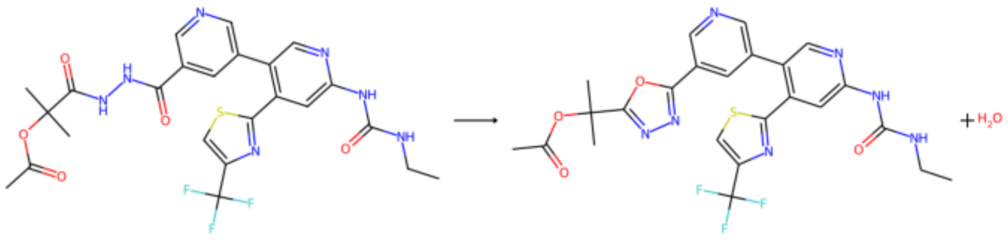


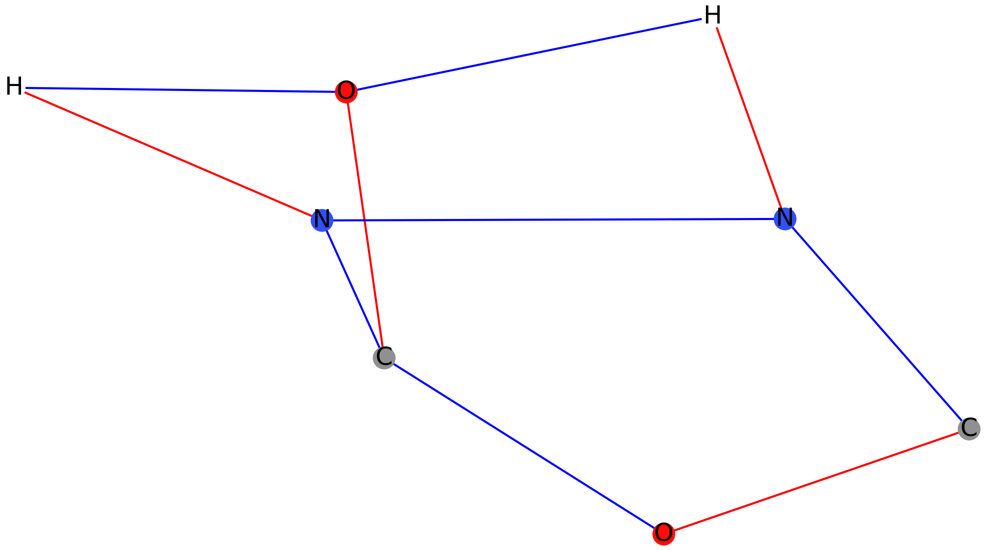

R-id = 10421 with reaction step = 4

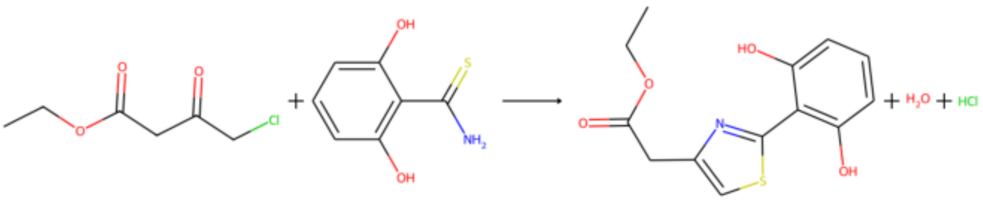


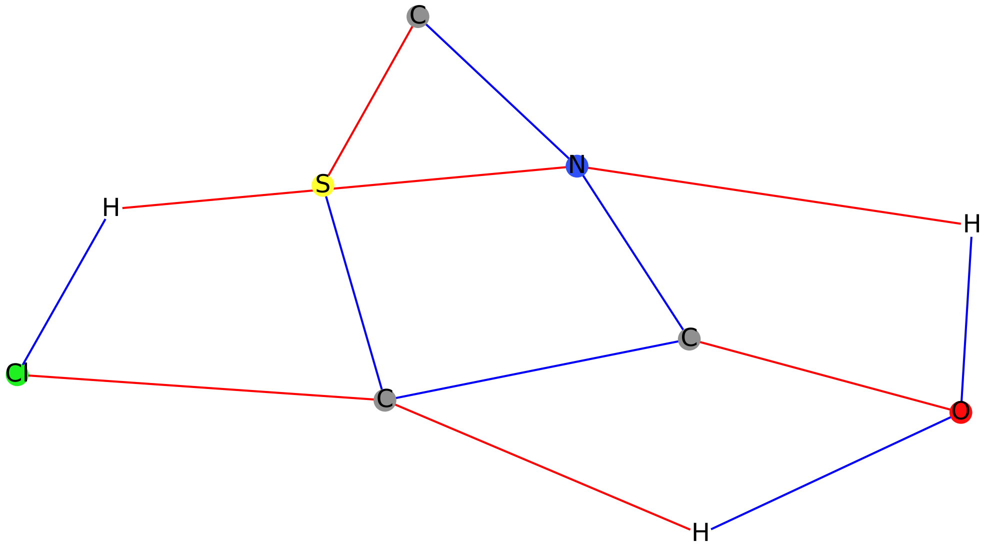

R-id = 24448 with reaction step = 1

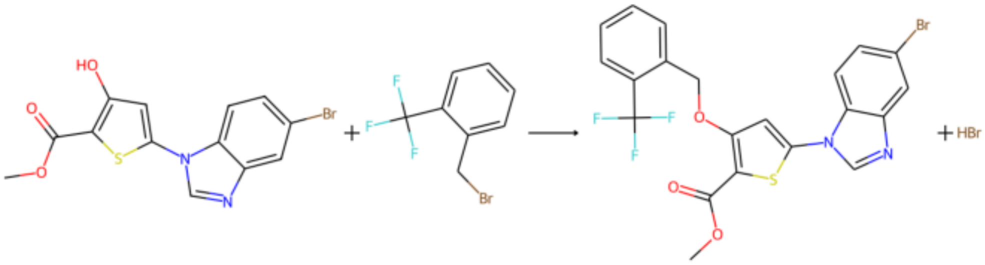


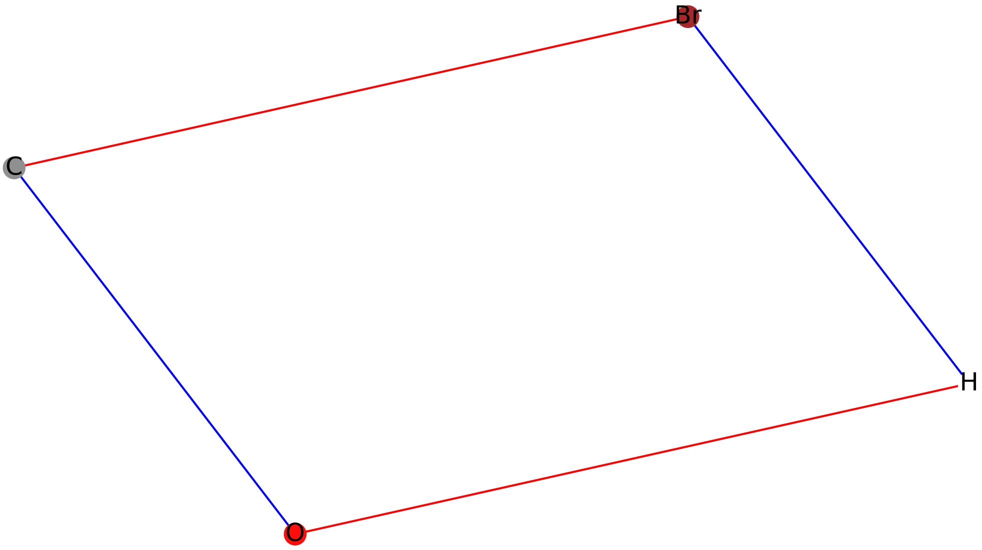

R-id = 11070 with reaction step = 2

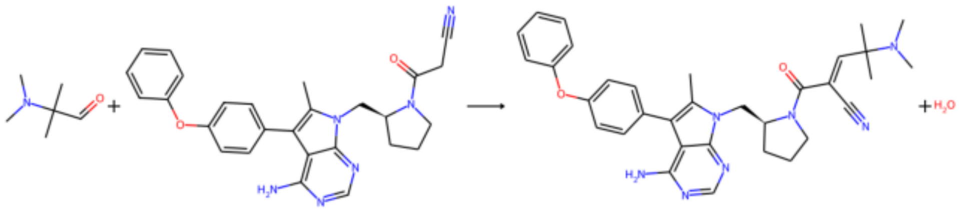

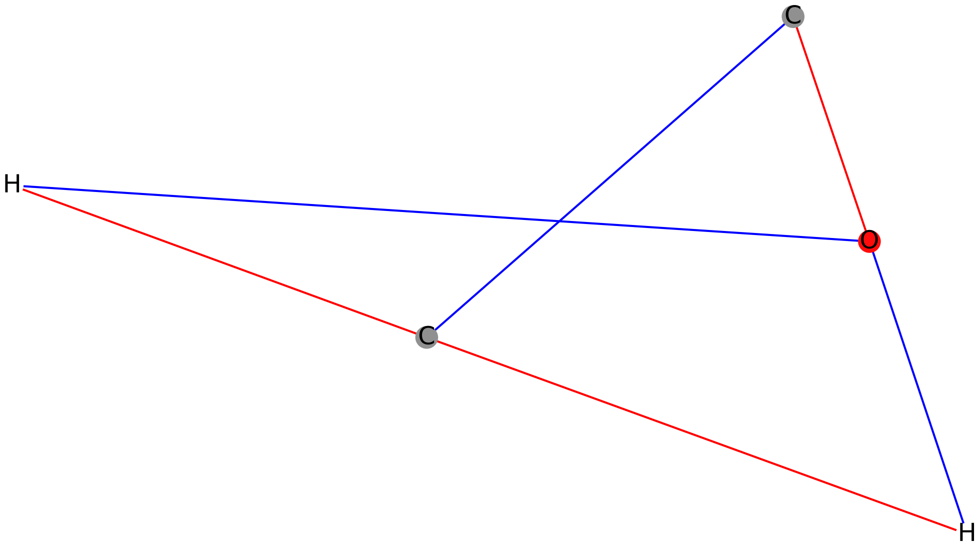

R-id = 11849 with reaction step = 1

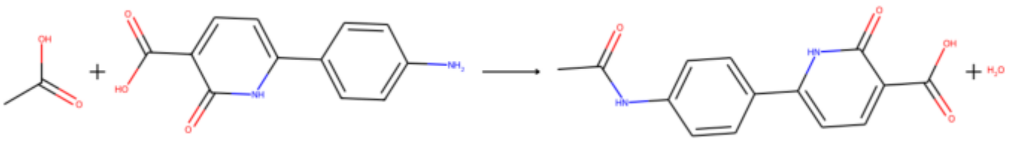


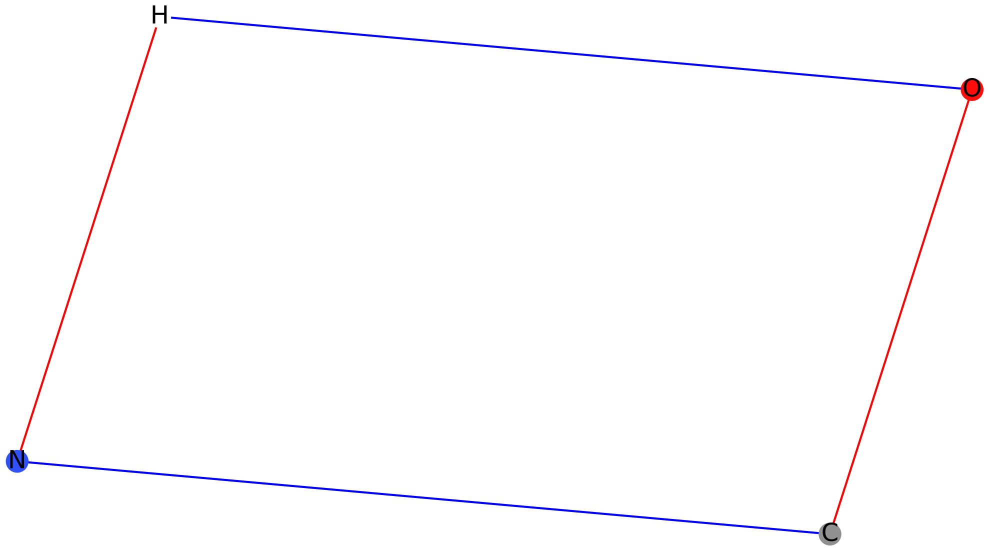

R-id = 14487 with reaction step = 1

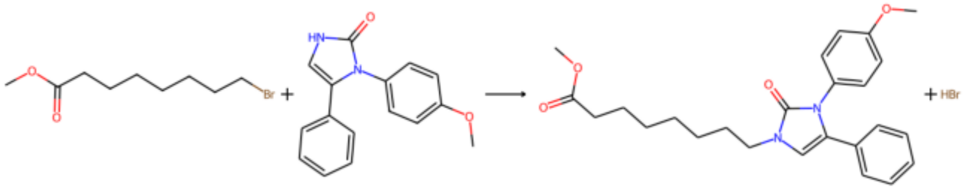


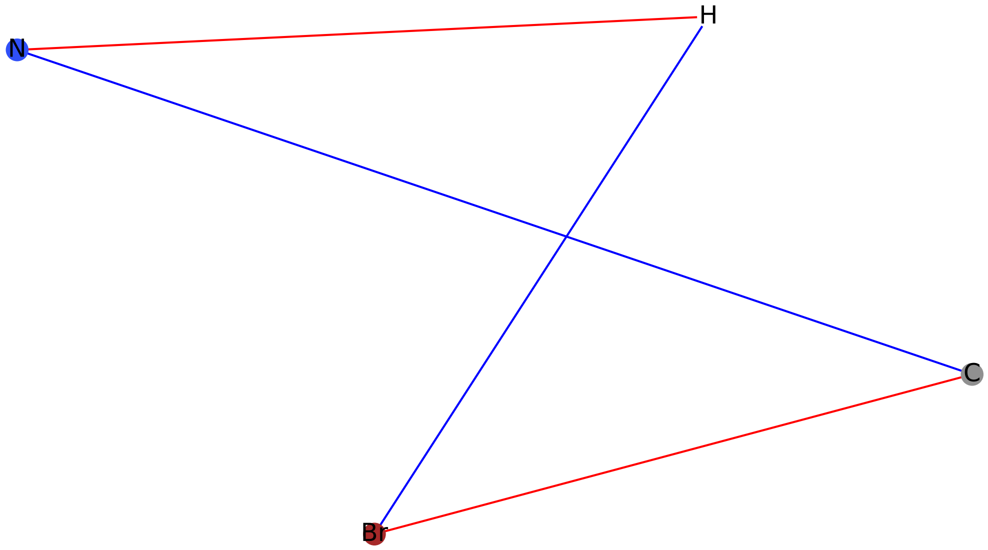

R-id = 15011 with reaction step = 2

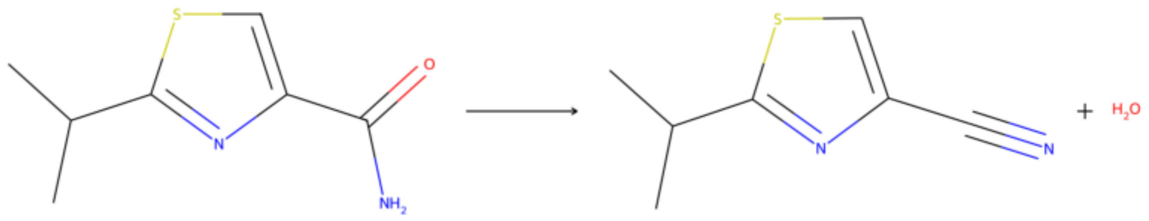


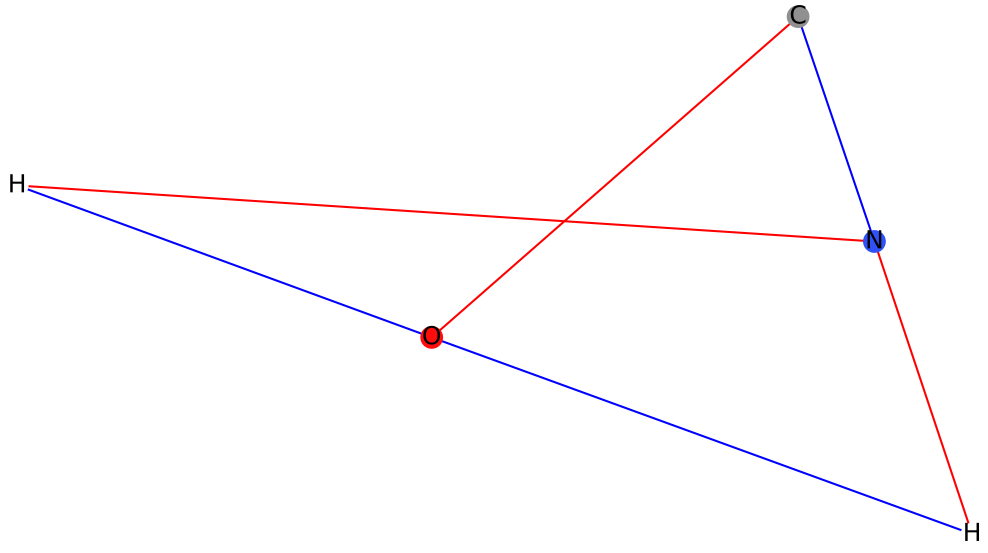

R-id = 22758 with reaction step = 1

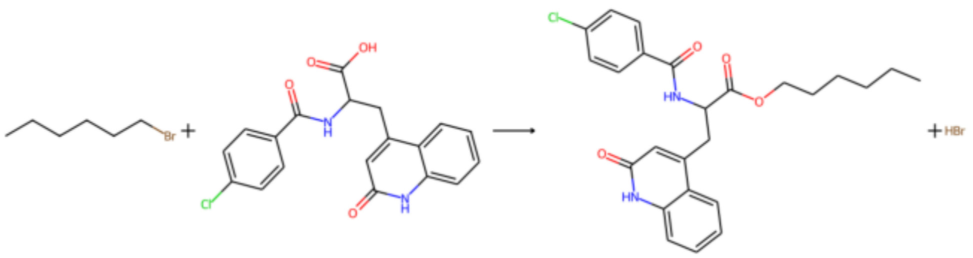


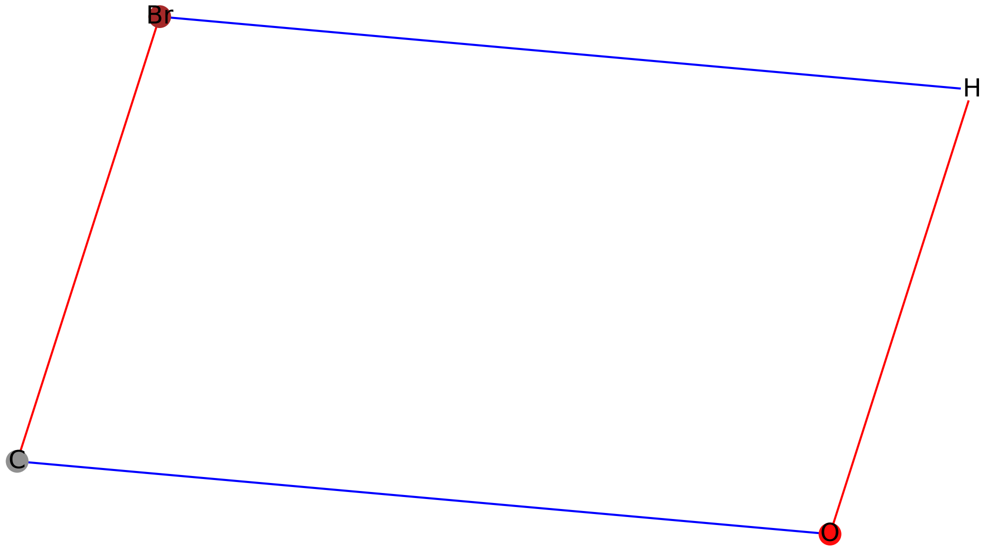

R-id = 28048 with reaction step = 1

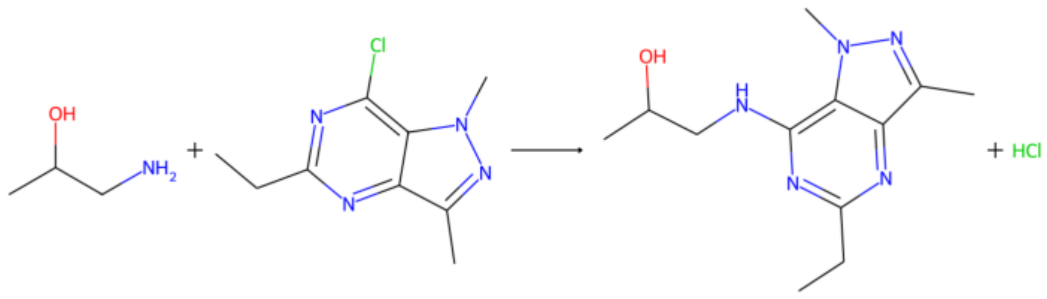


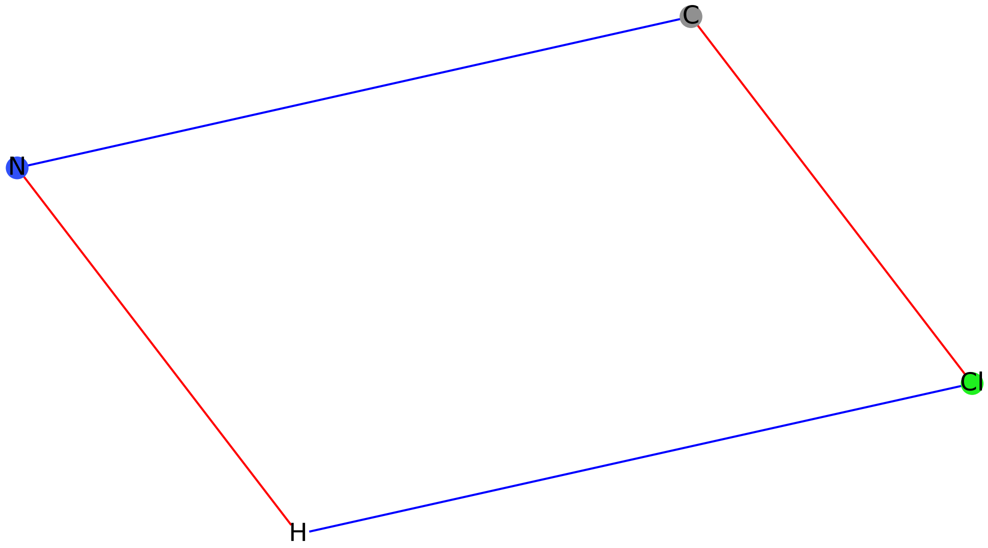

R-id = 29963 with reaction step = 1

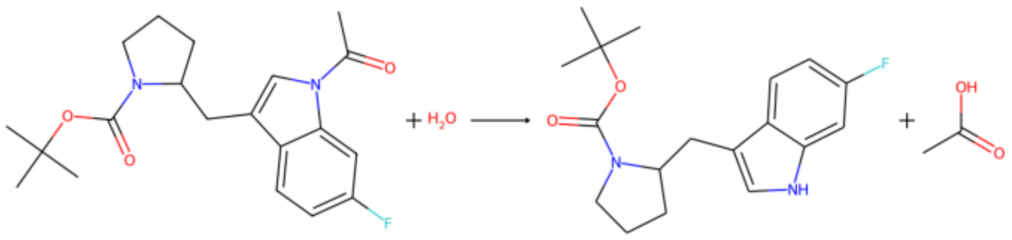

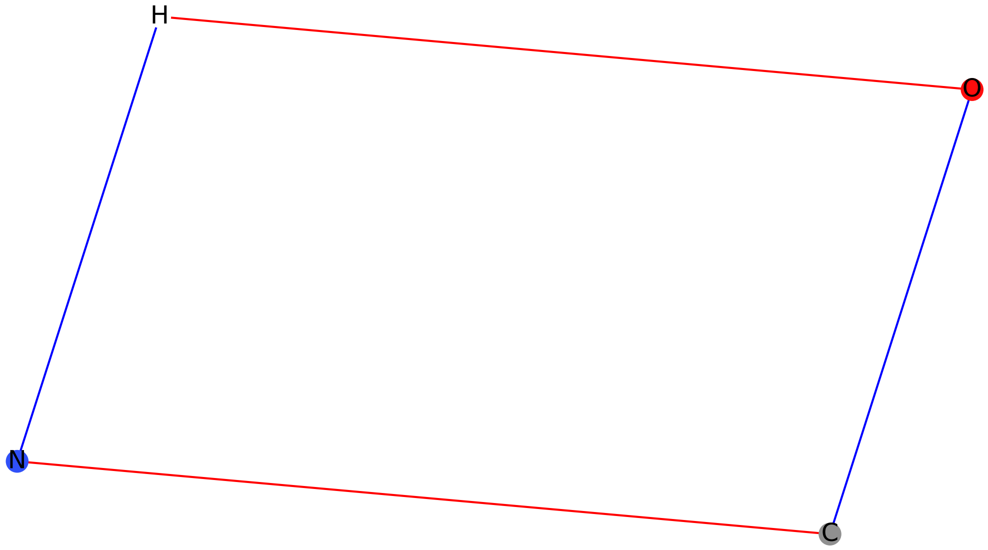

R-id = 7608 with reaction step = 2

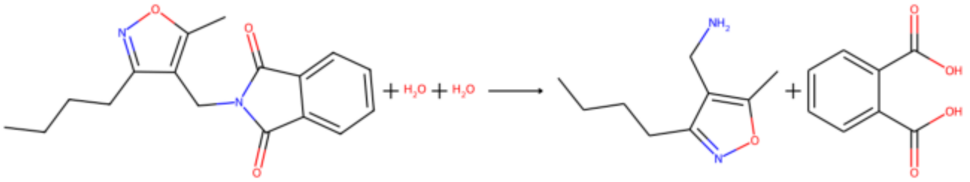

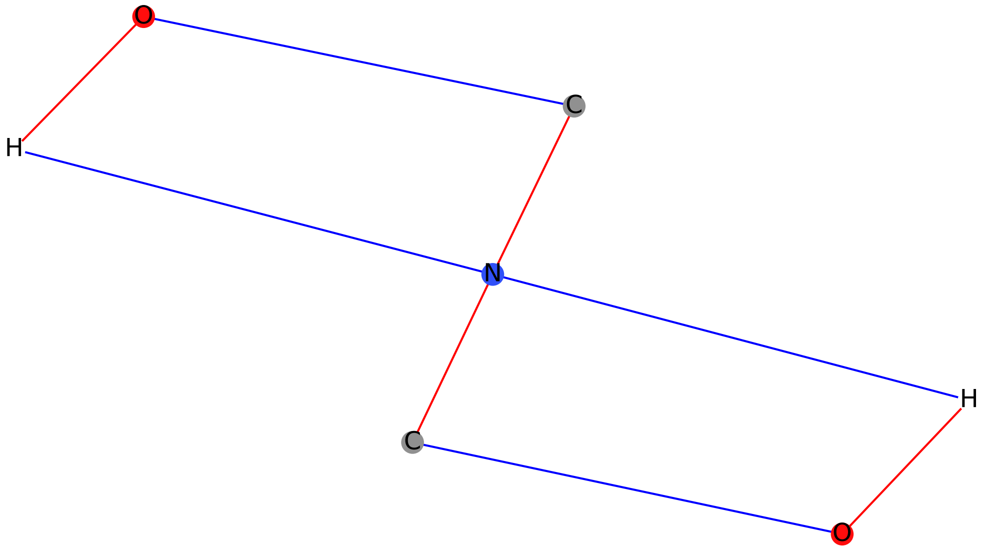

R-id = 38210 with reaction step = 1

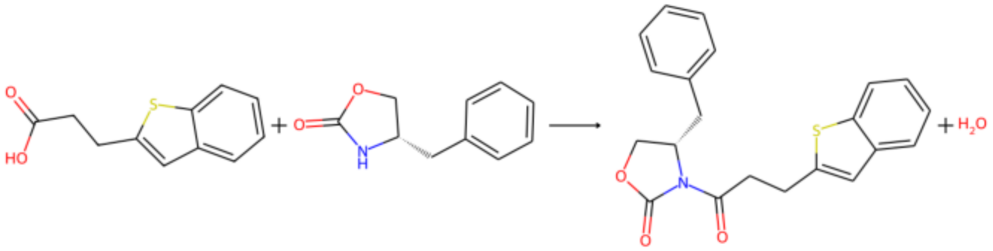


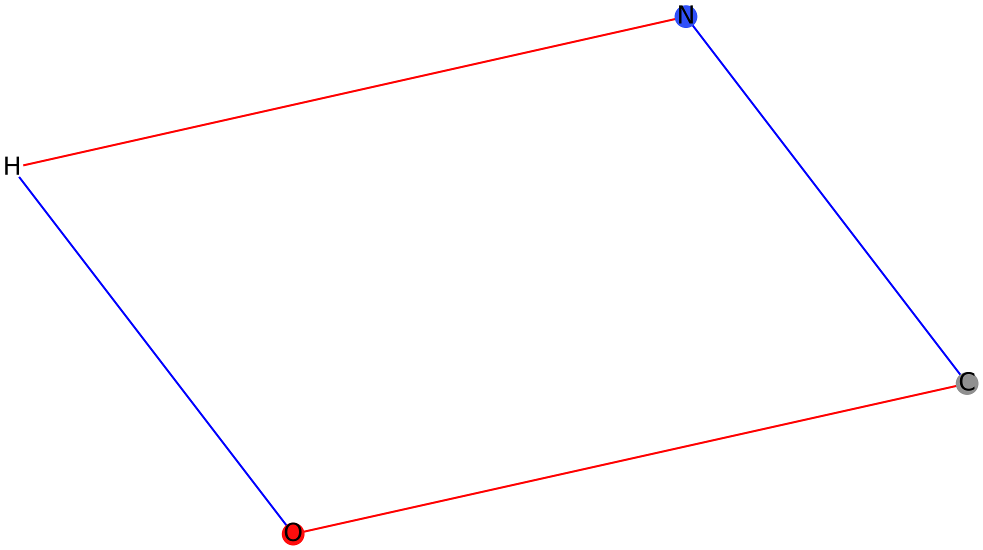

R-id = 41729 with reaction step = 2

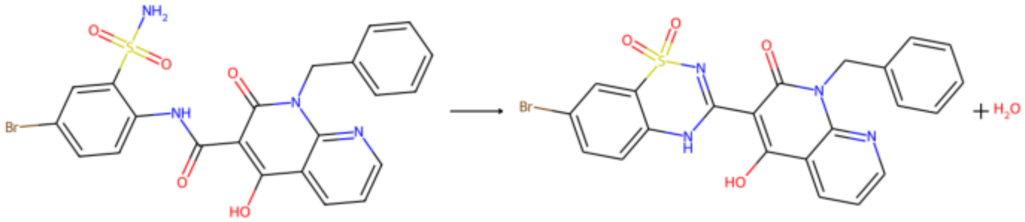


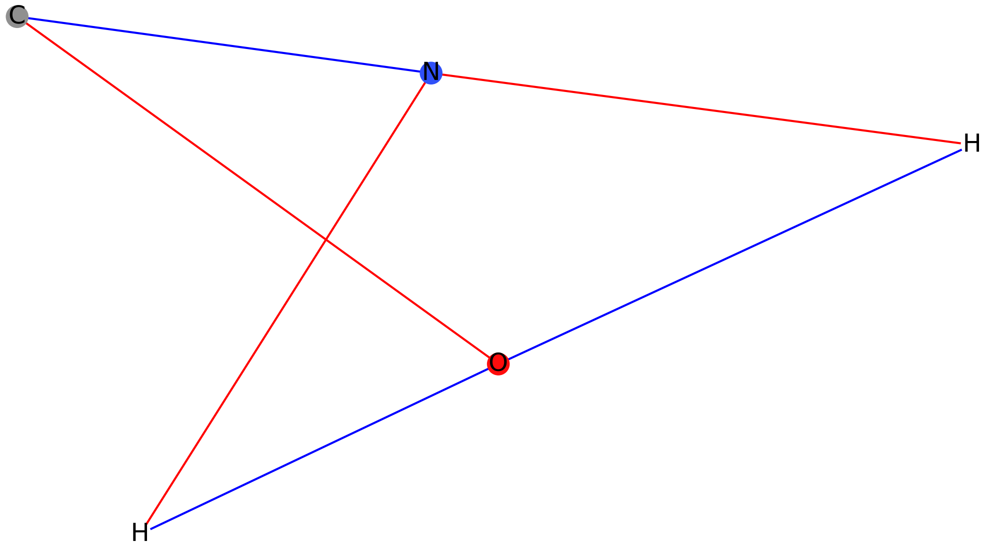

R-id = 34718 with reaction step = 1

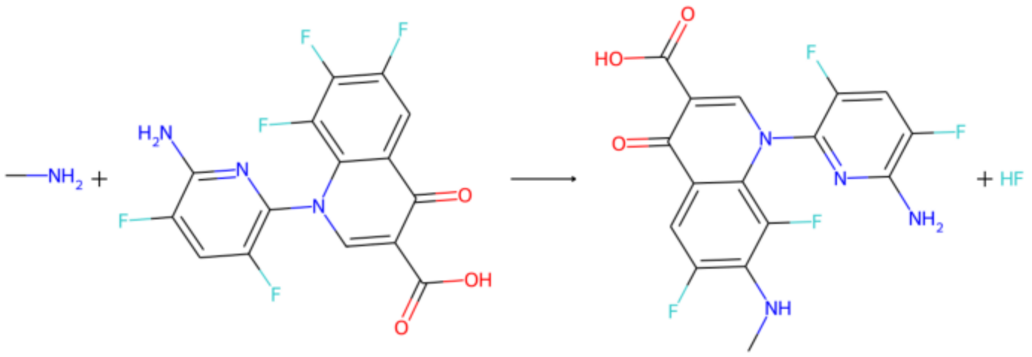

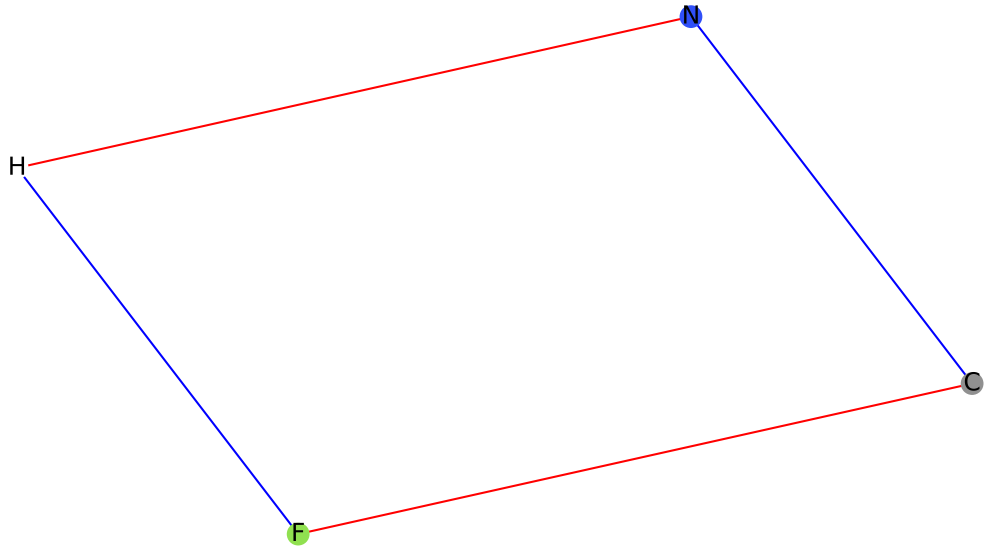

R-id = 49169 with reaction step = 1

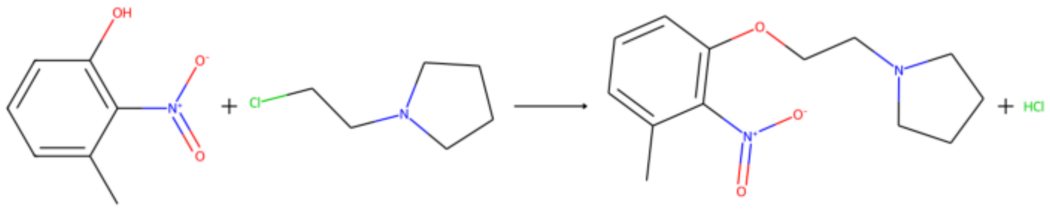


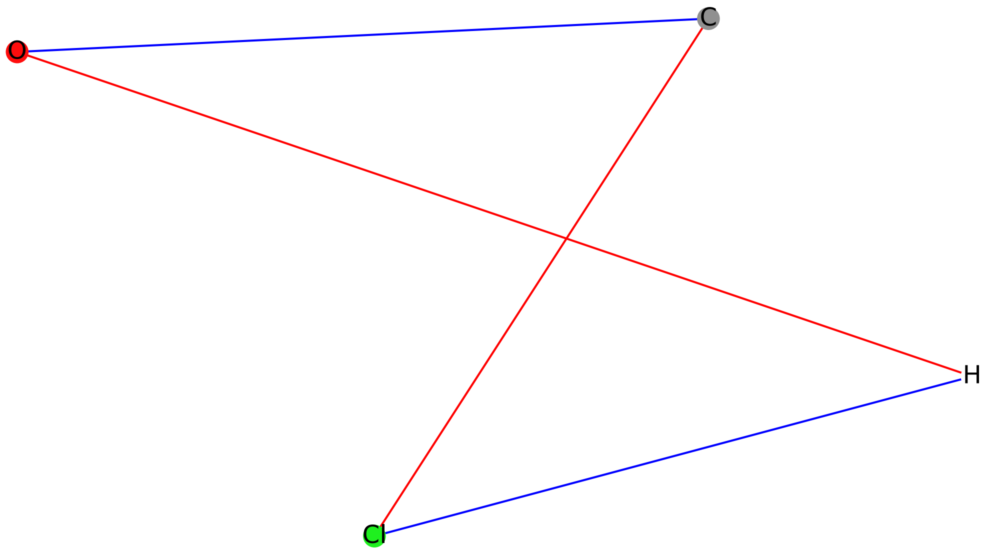

R-id = 13444 with reaction step = 2

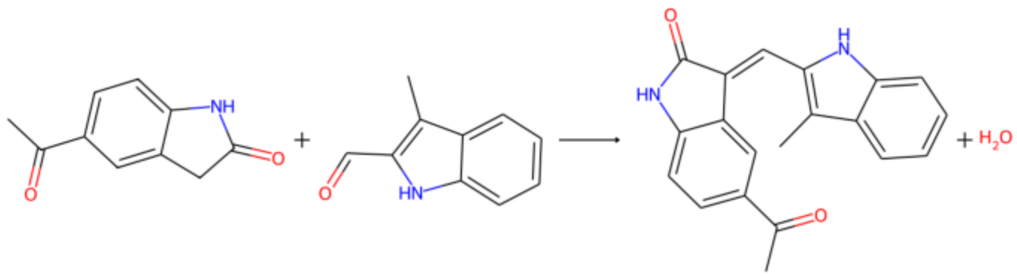

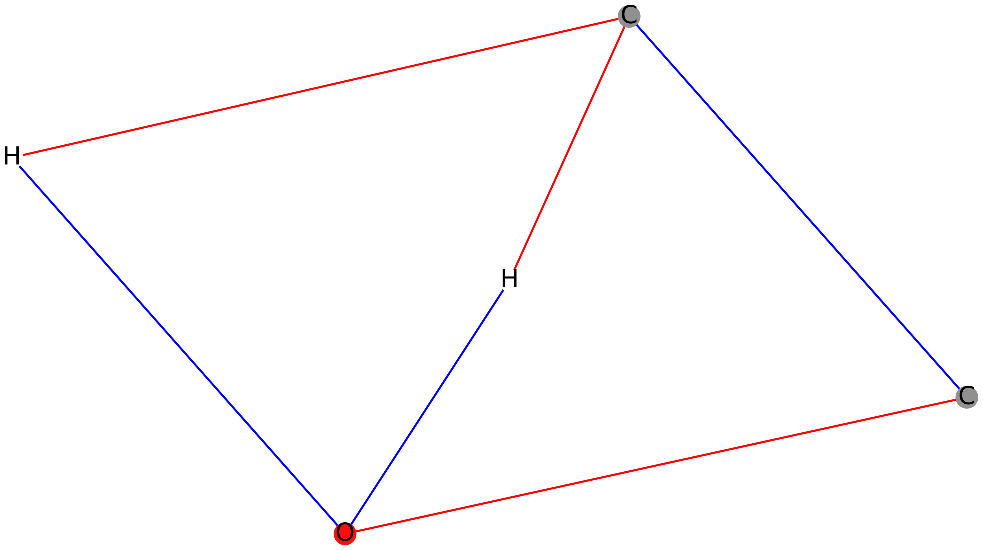

R-id = 30085 with reaction step = 1

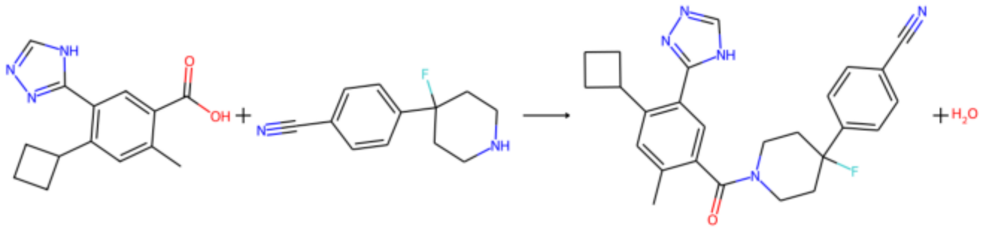


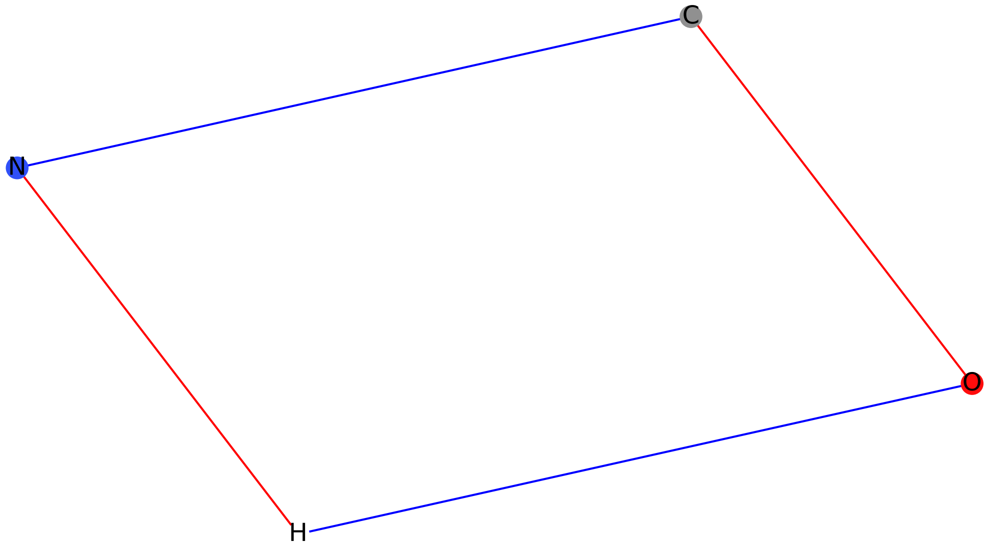

R-id = 40496 with reaction step = 1

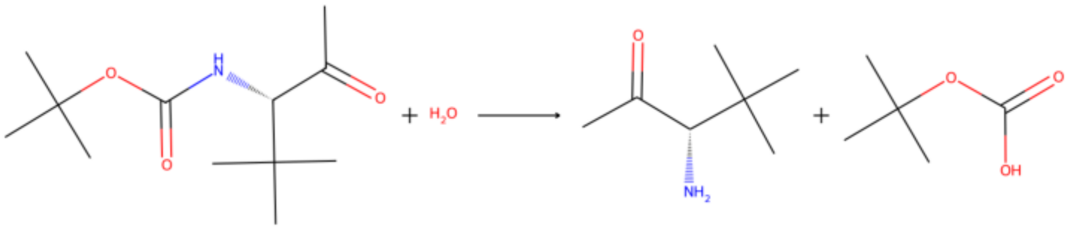


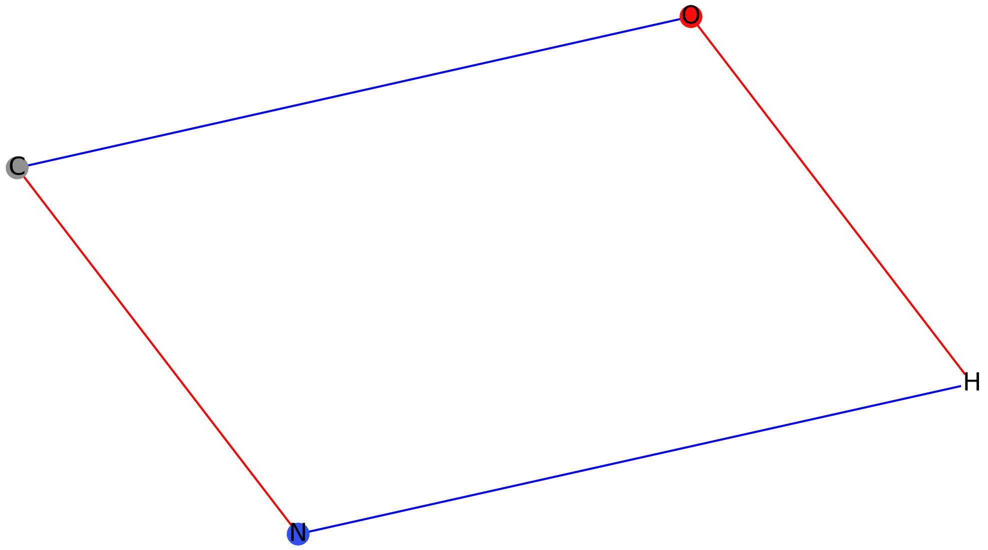

R-id = 27815 with reaction step = 1

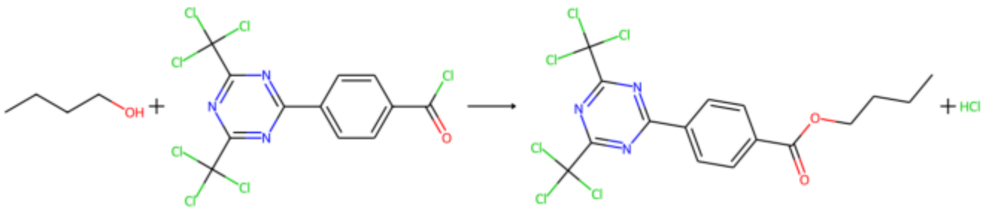


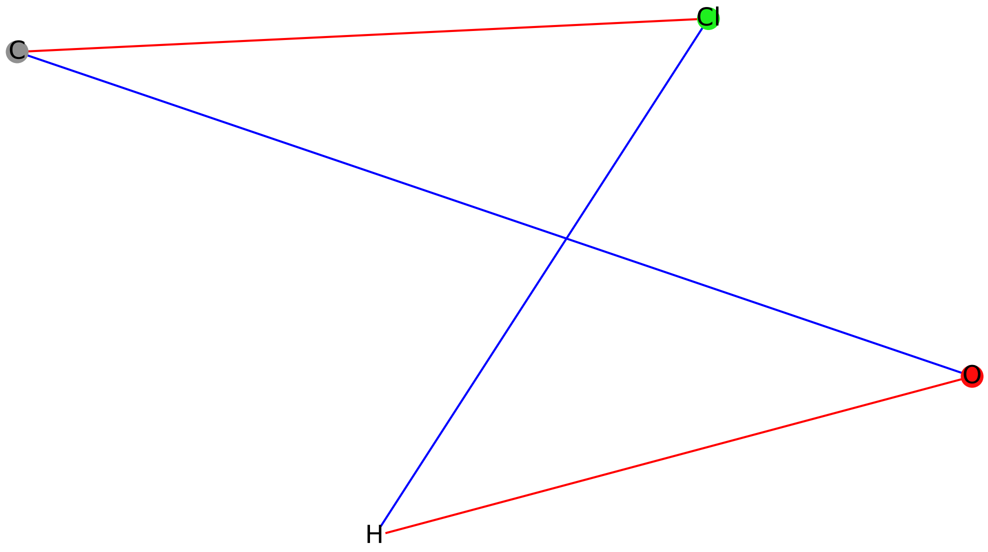

R-id = 13184 with reaction step = 1

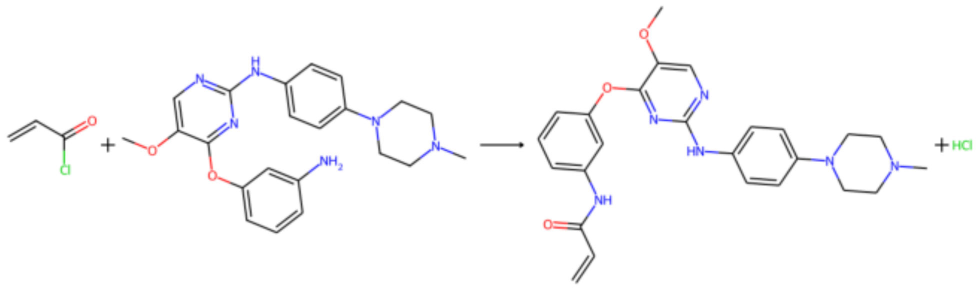

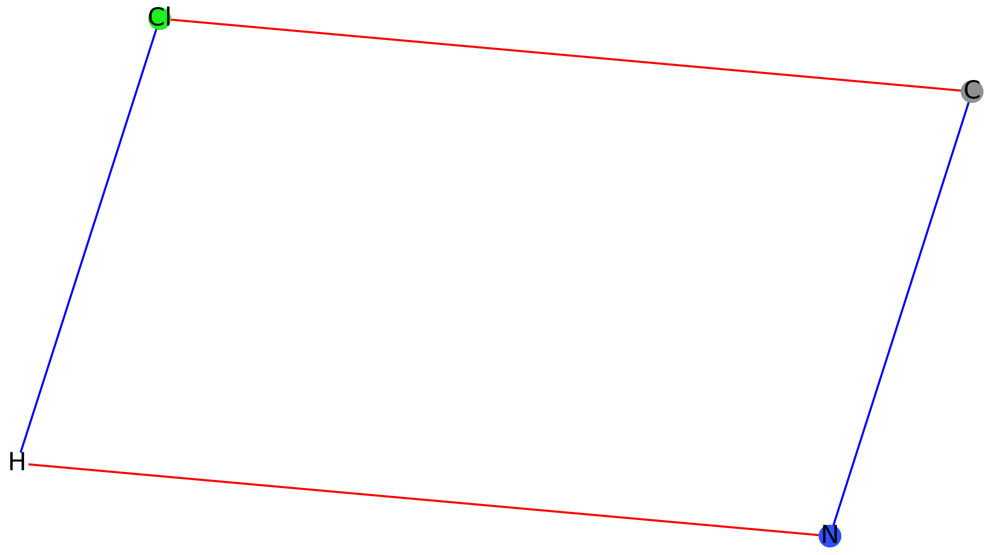

R-id = 9837 with reaction step = 2

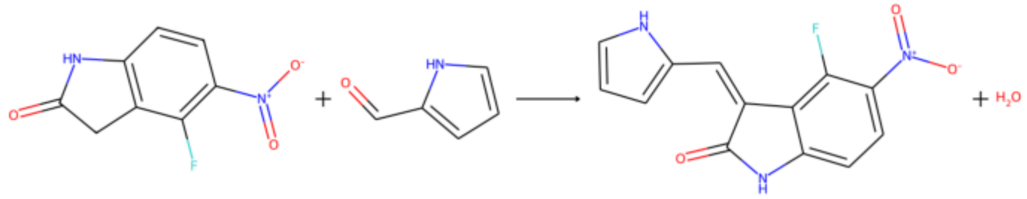


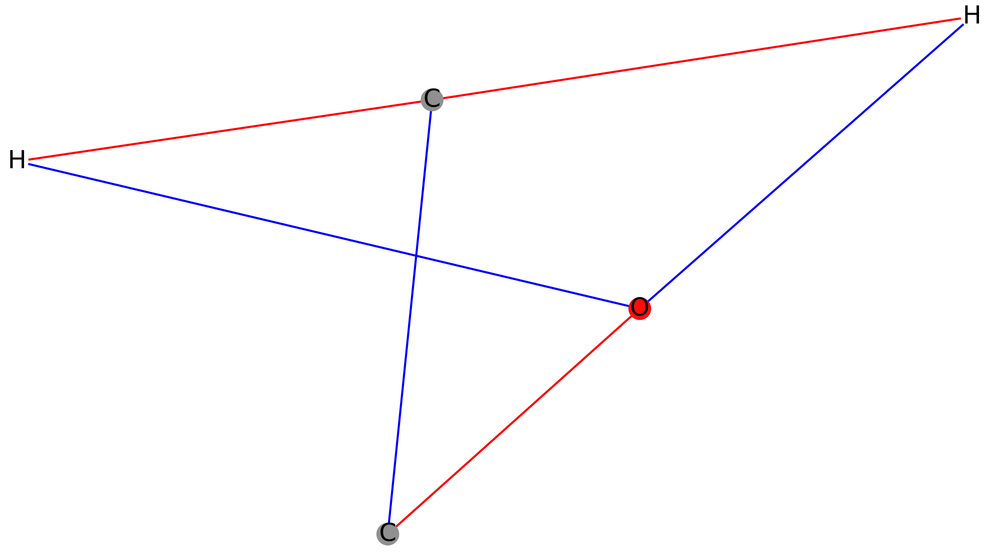

R-id = 21469 with reaction step = 2

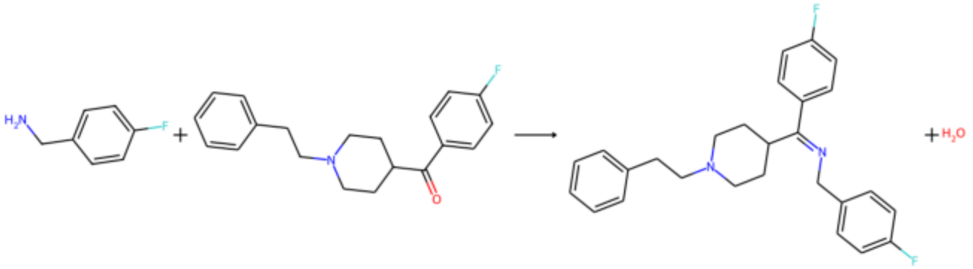


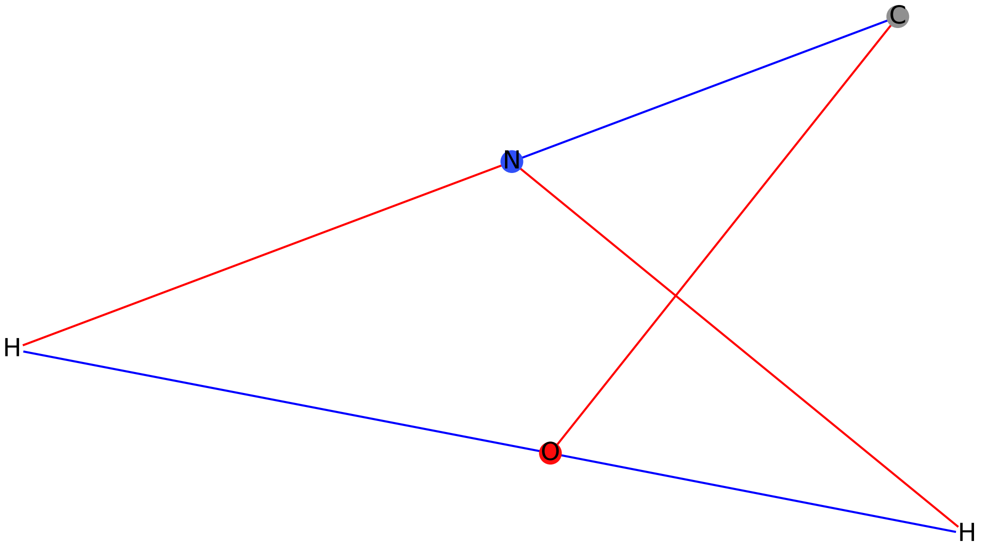

R-id = 43591 with reaction step = 1

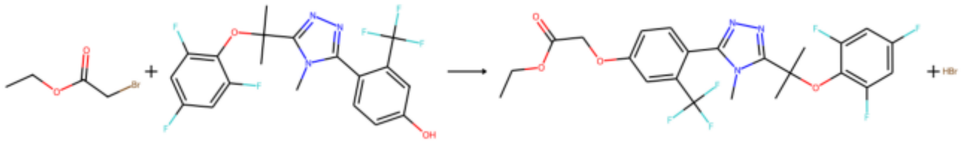

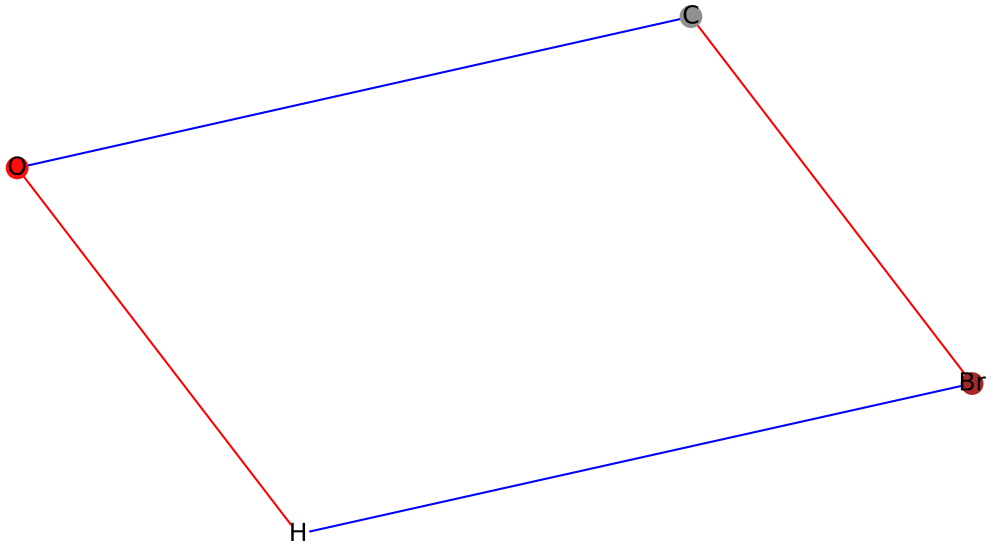

R-id = 30927 with reaction step = 2

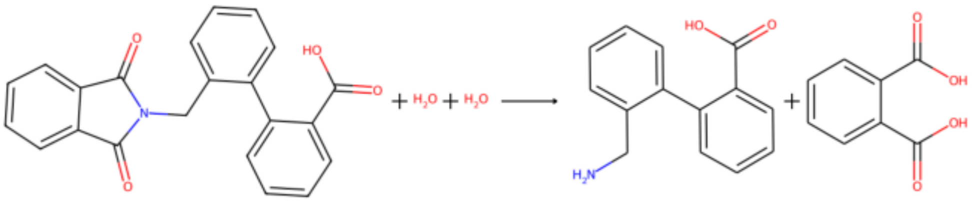

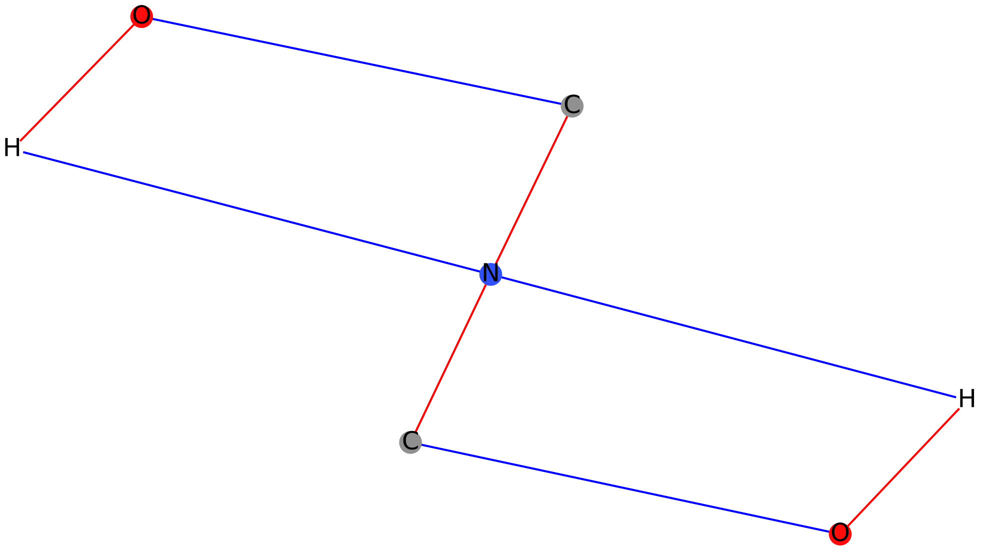

R-id = 7026 with reaction step = 2

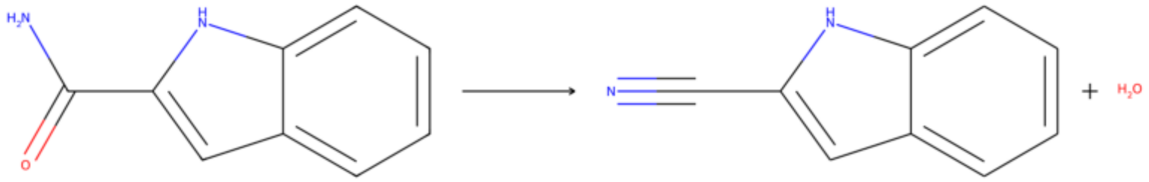


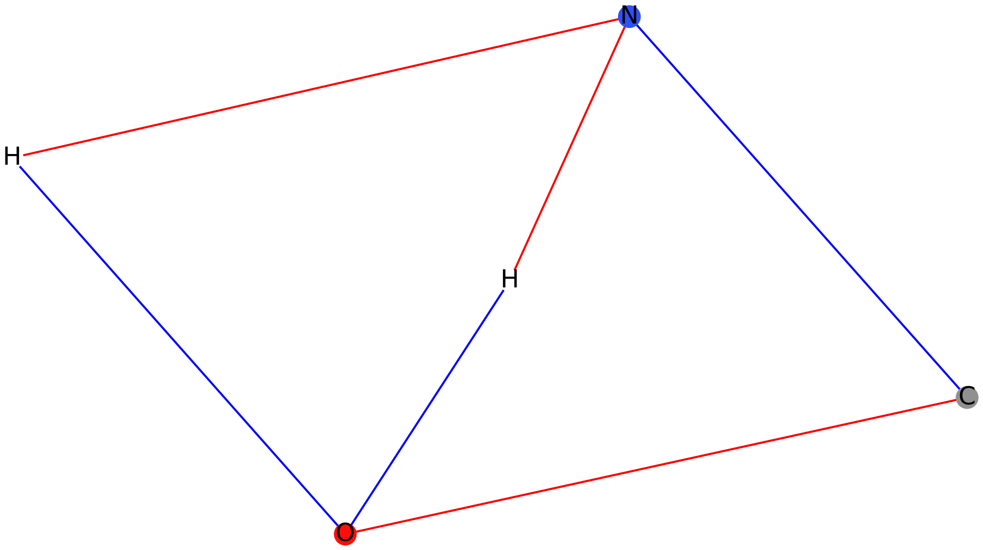

R-id = 9266 with reaction step = 1

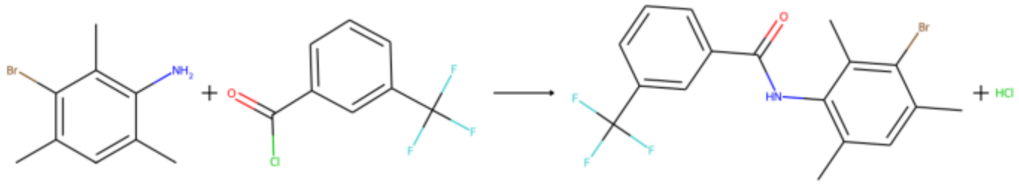


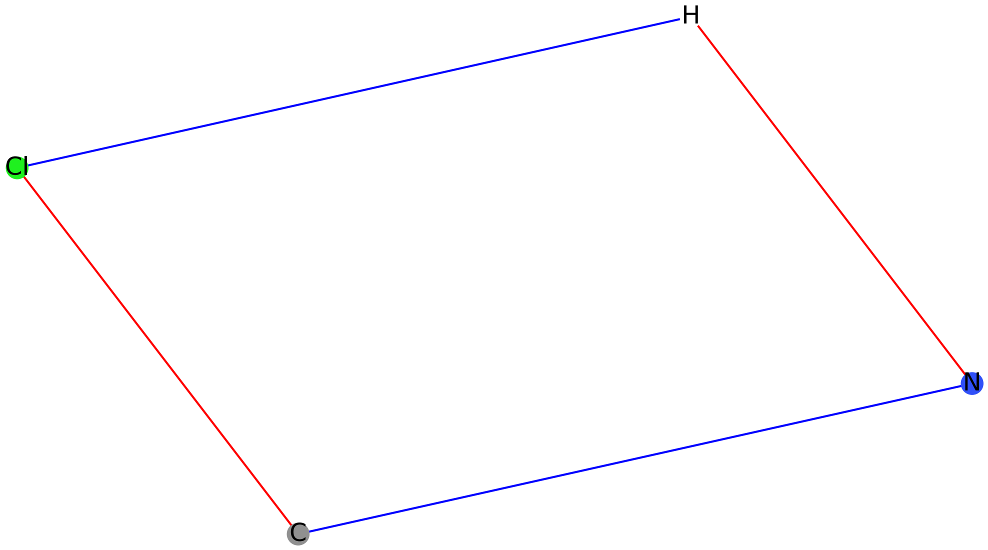

R-id = 16518 with reaction step = 1

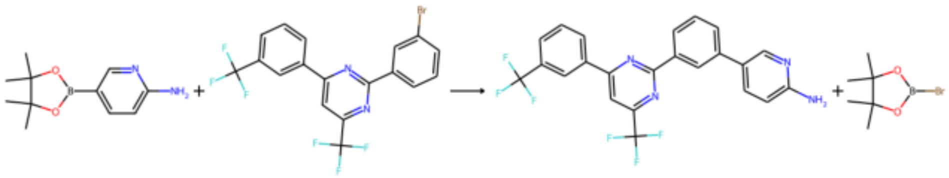

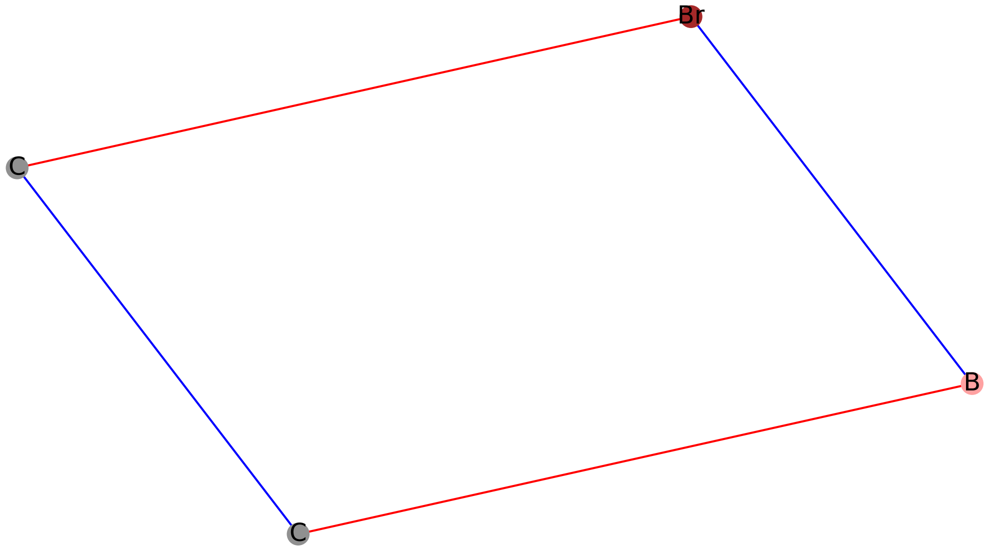

R-id = 49127 with reaction step = 2

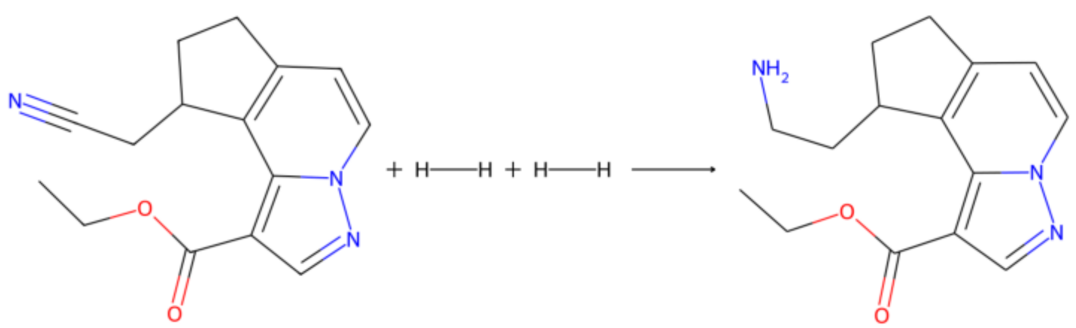


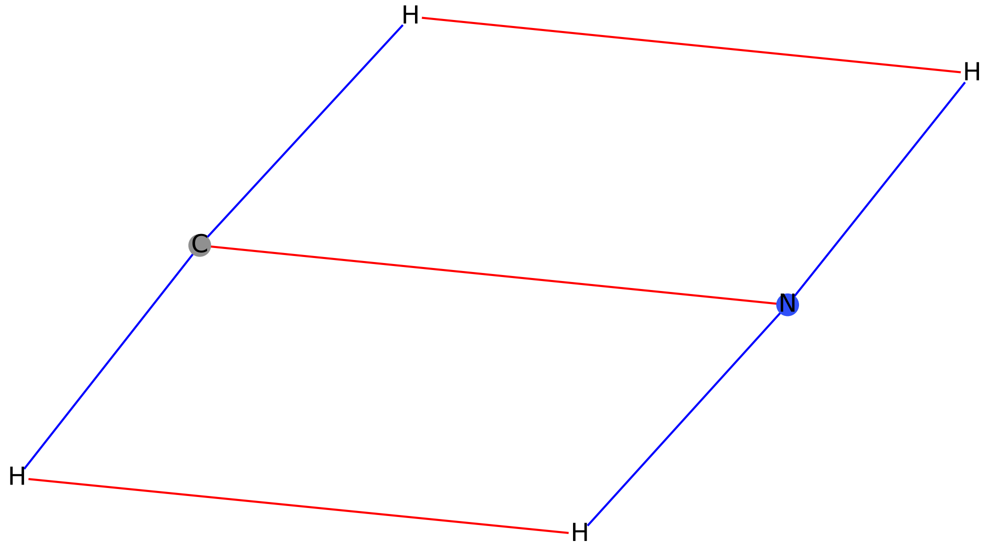

R-id = 33275 with reaction step = 1

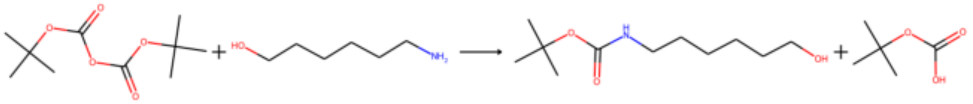


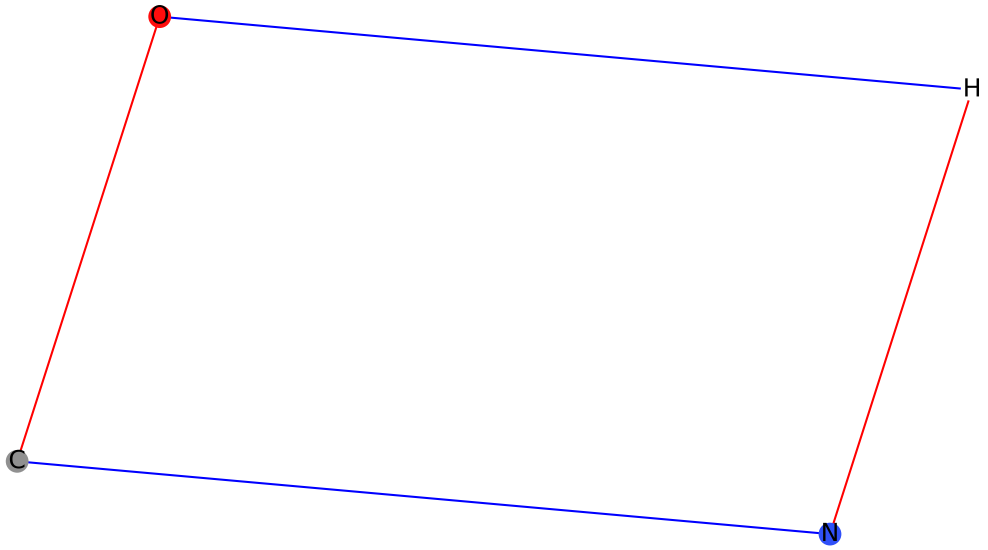

R-id = 18662 with reaction step = 2

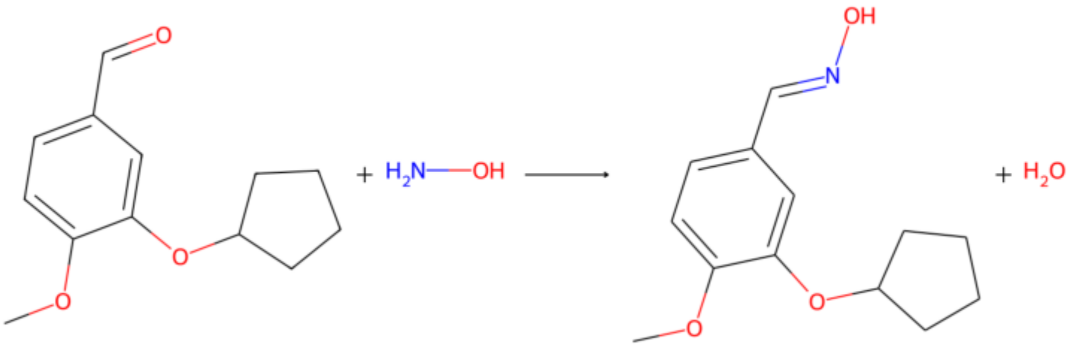


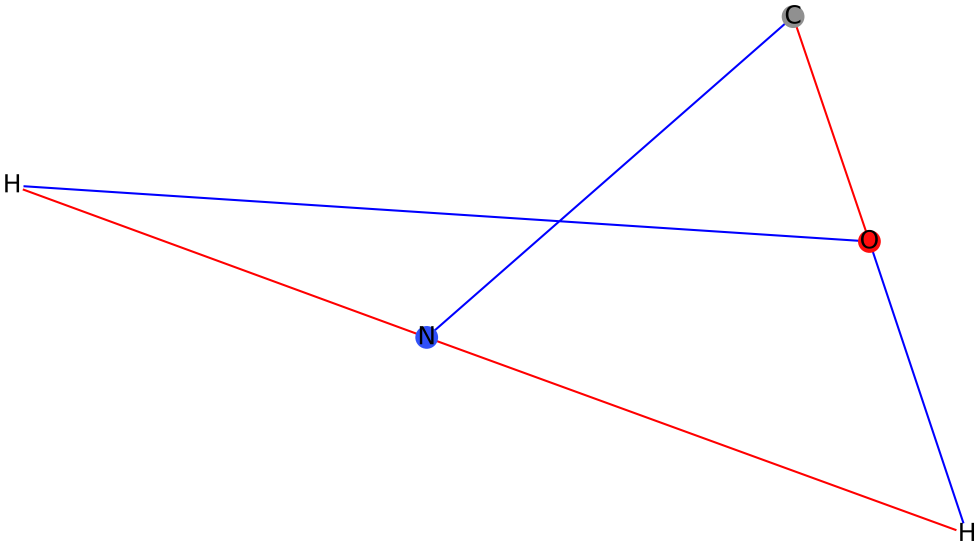

R-id = 17597 with reaction step = 1

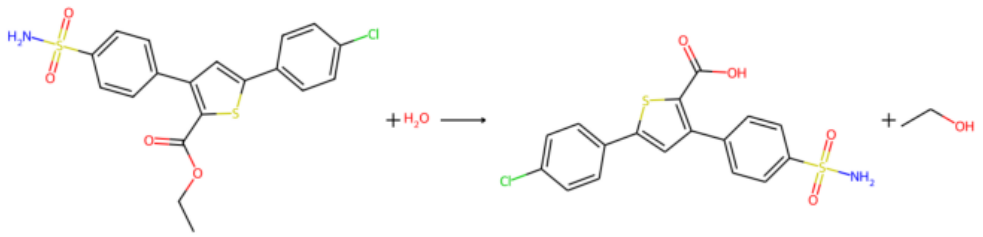


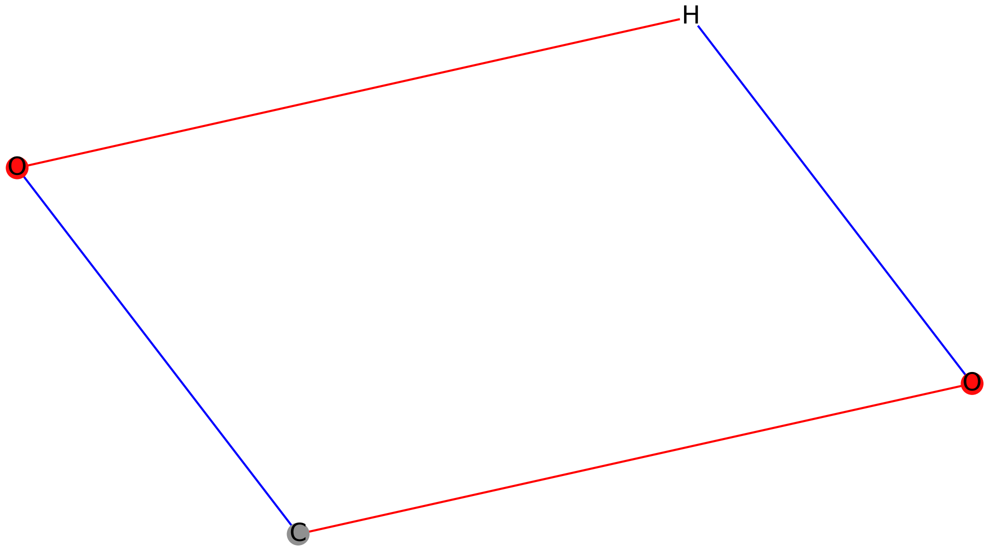

R-id = 43832 with reaction step = 1

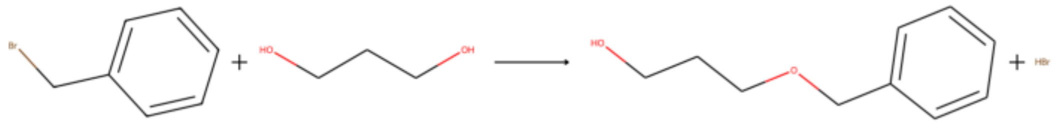


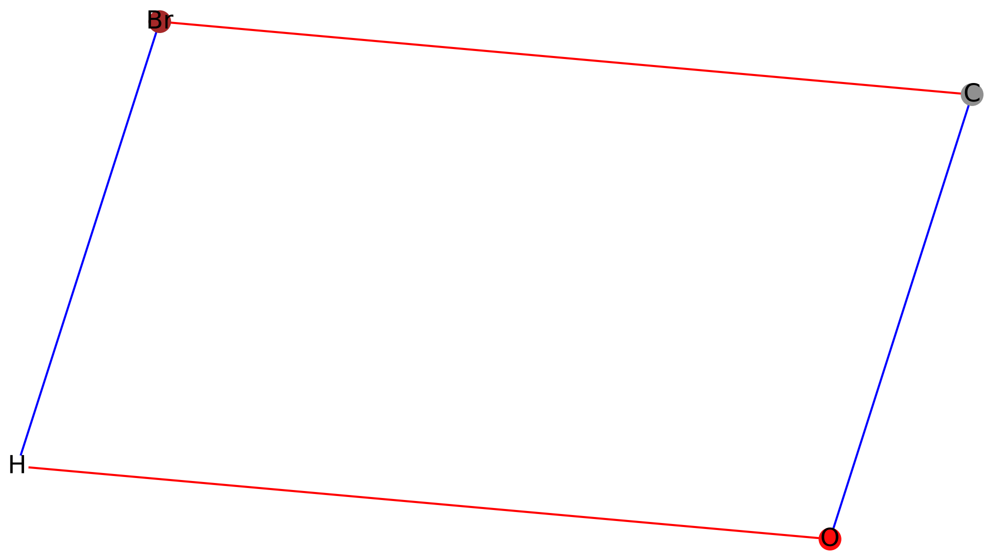

R-id = 21182 with reaction step = 1

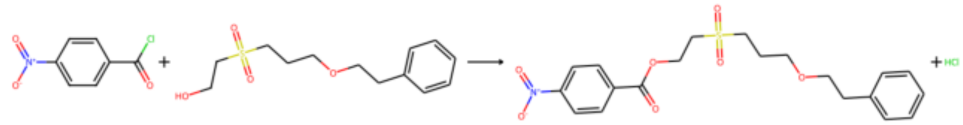

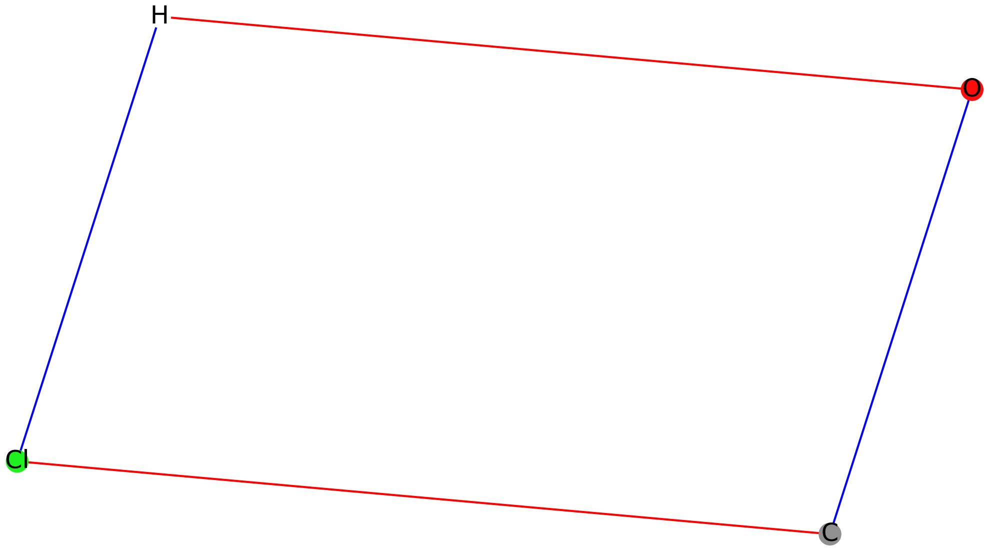

R-id = 9715 with reaction step = 2

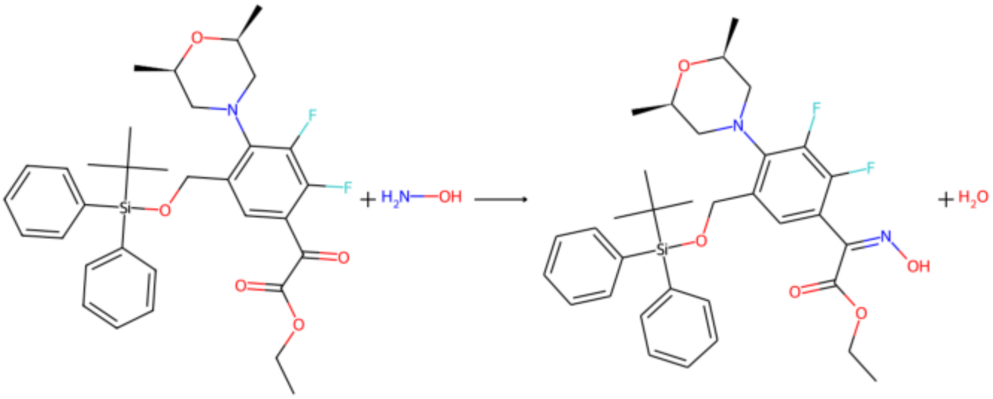


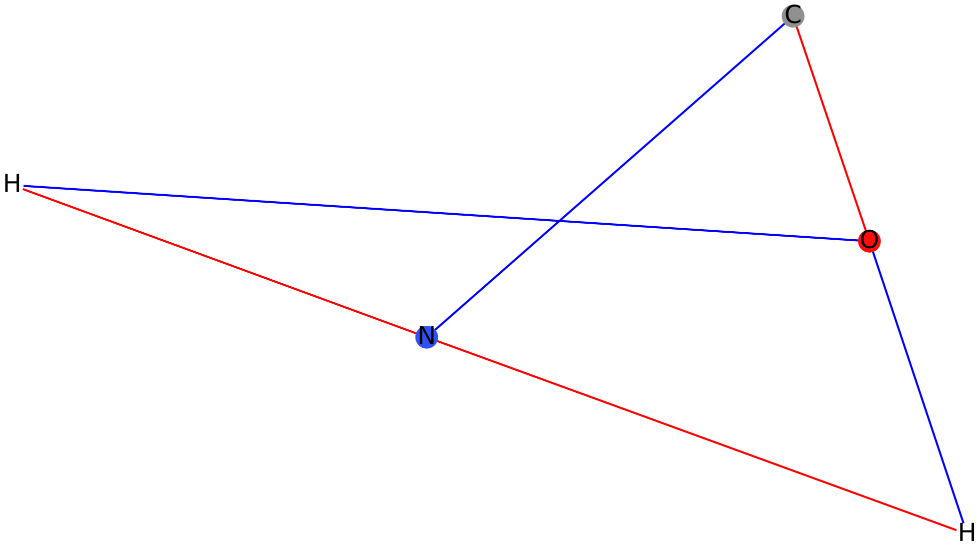

R-id = 12590 with reaction step = 2

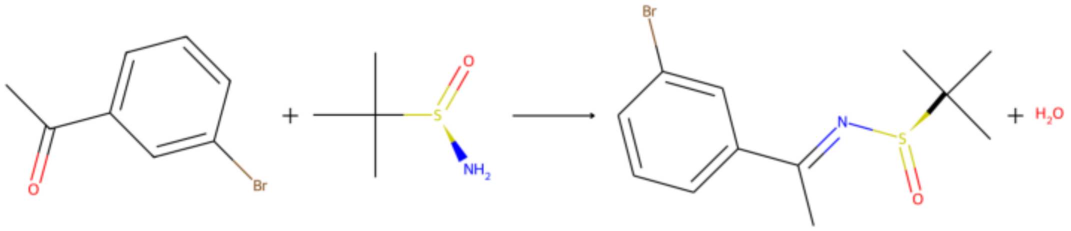


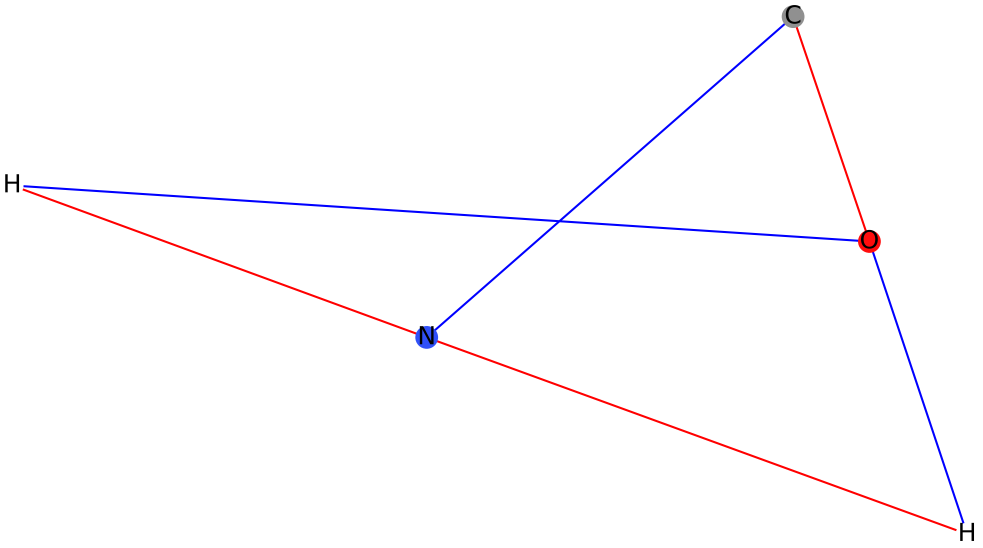

R-id = 1917 with reaction step = 1

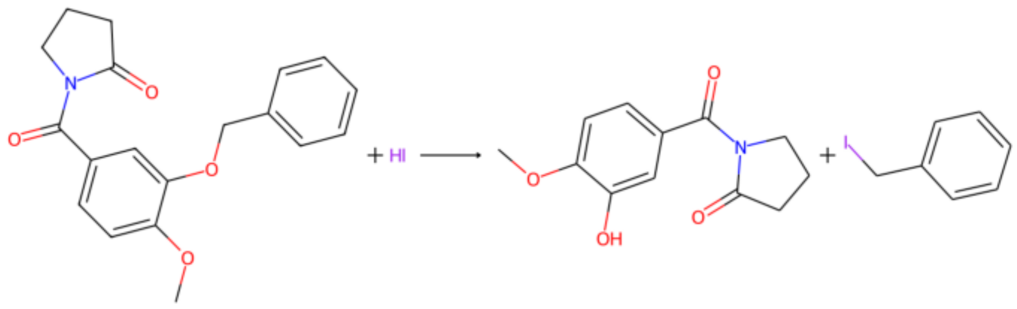

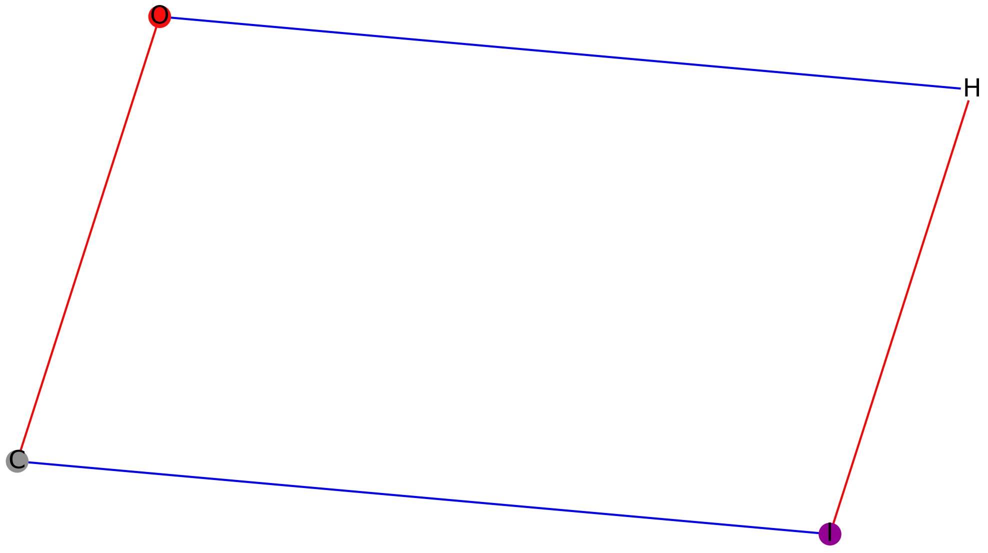

R-id = 29175 with reaction step = 1

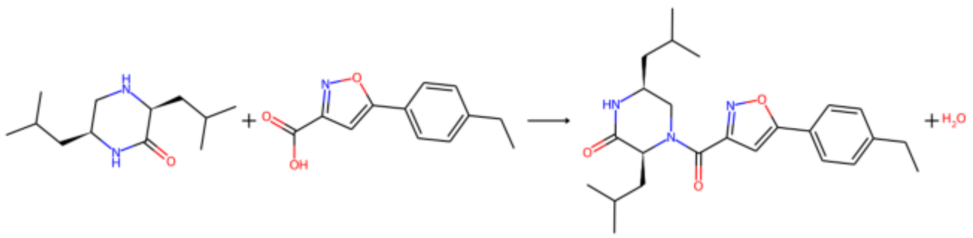

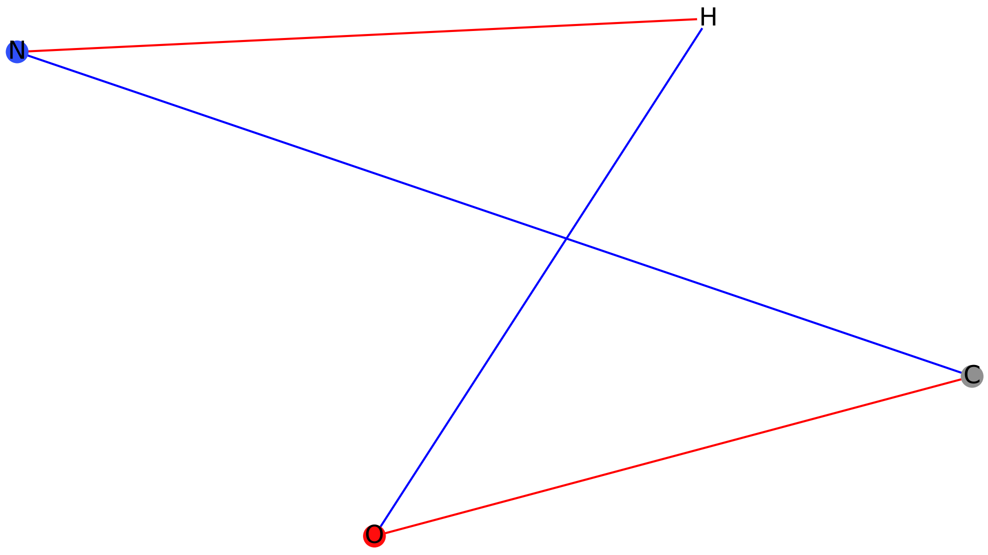

R-id = 8838 with reaction step = 3

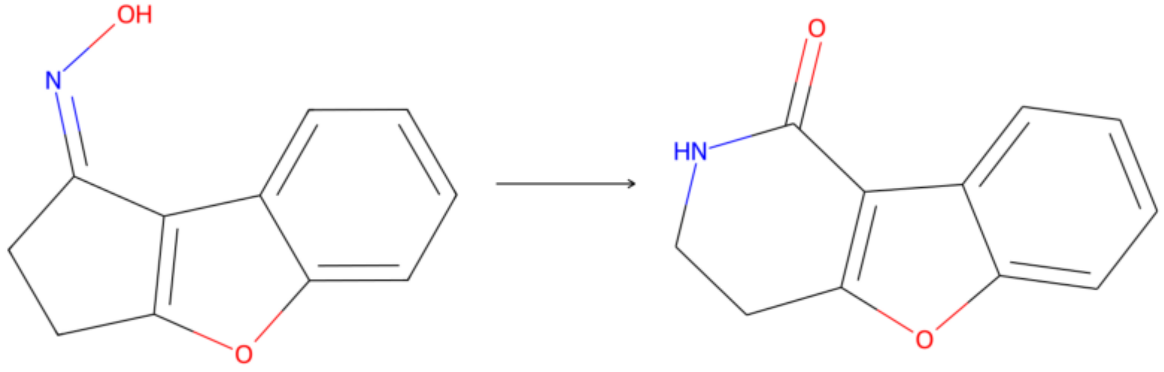


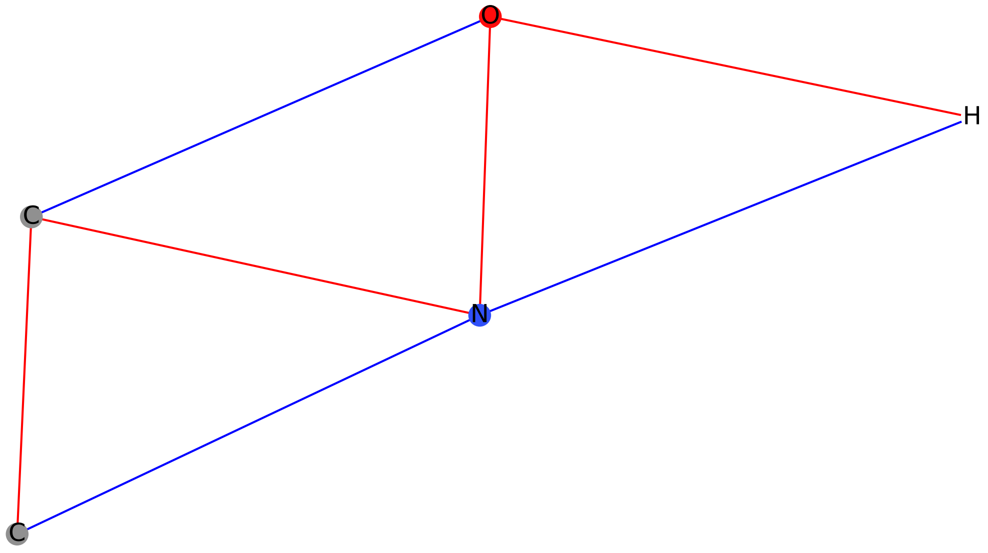

R-id = 31985 with reaction step = 1

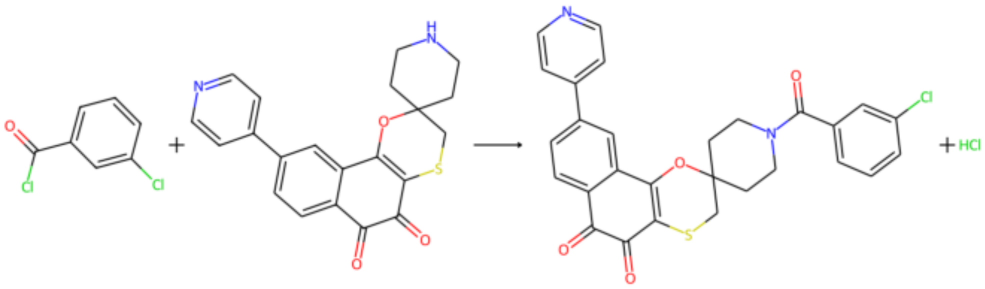


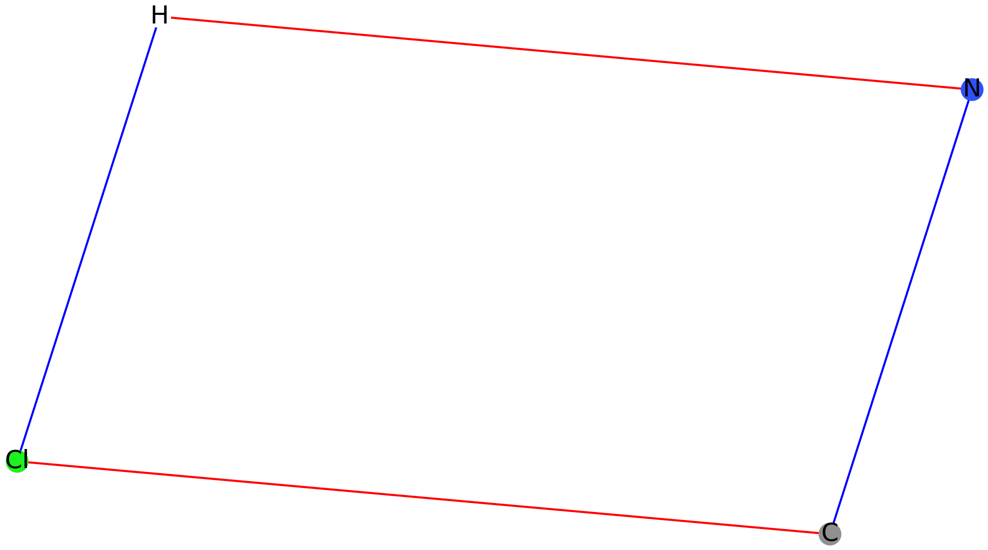

R-id = 41544 with reaction step = 1

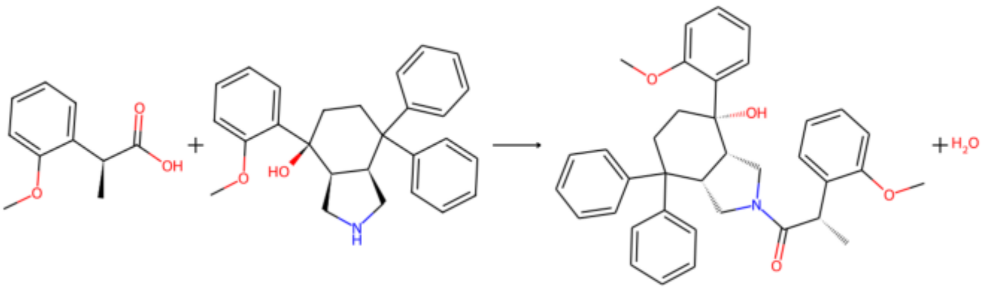


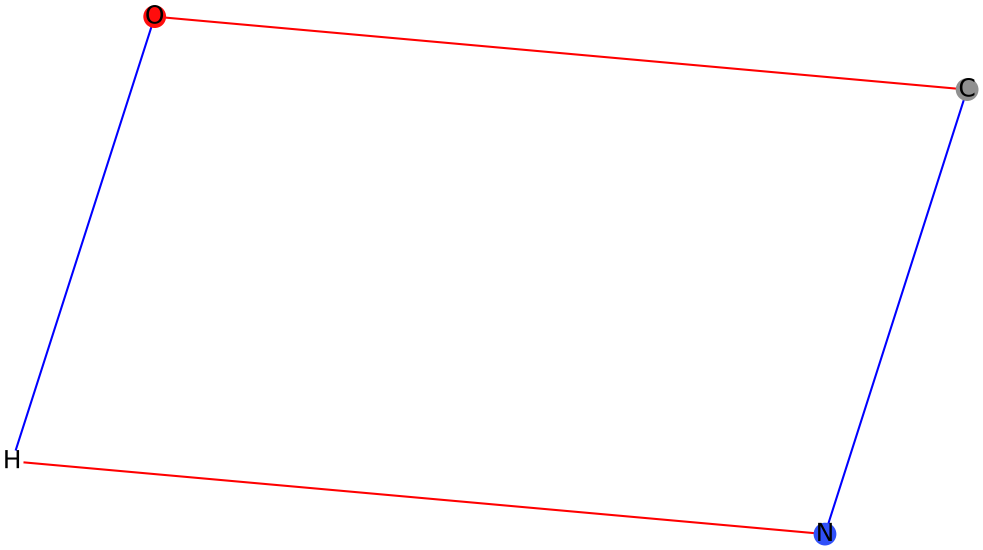

R-id = 6153 with reaction step = 4

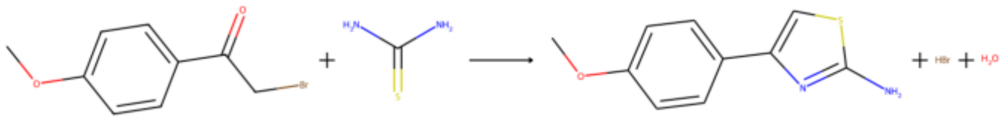

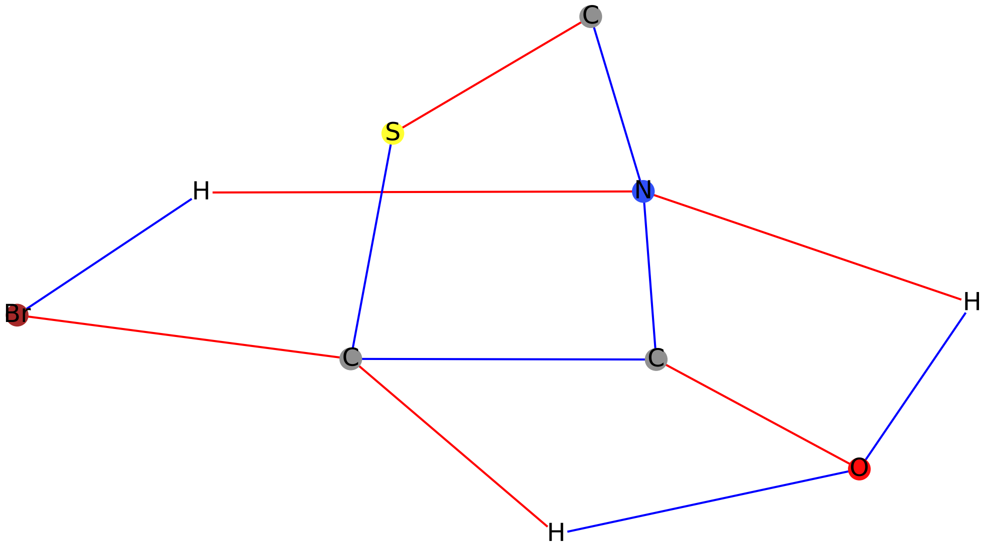

R-id = 19398 with reaction step = 1

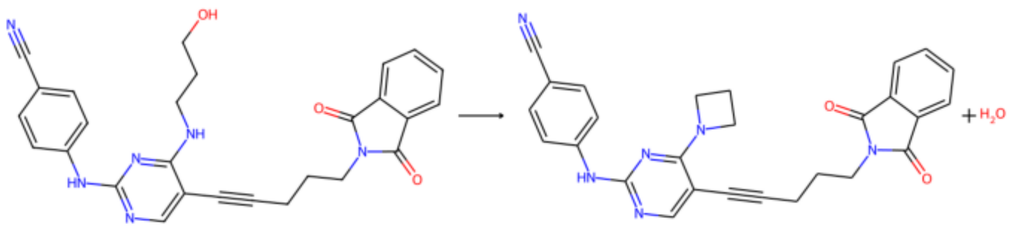


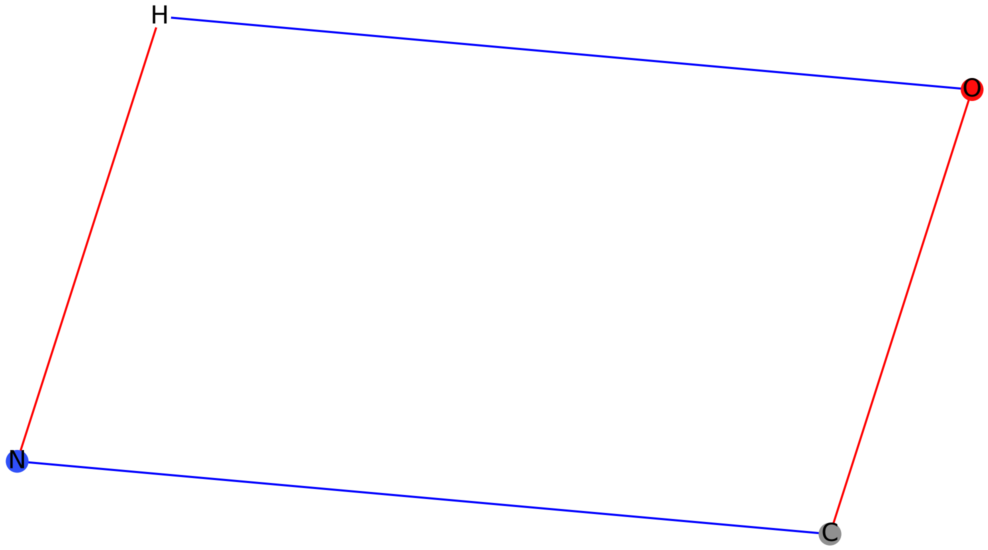

R-id = 14459 with reaction step = 1

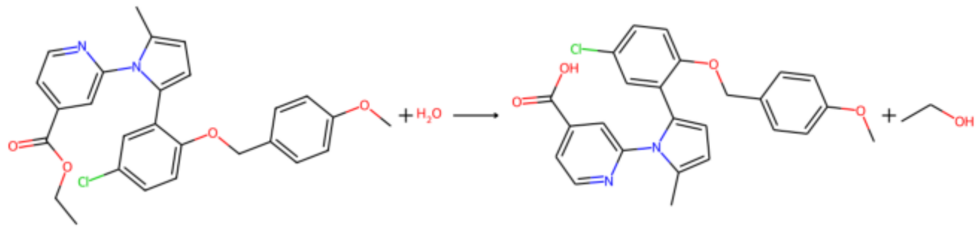


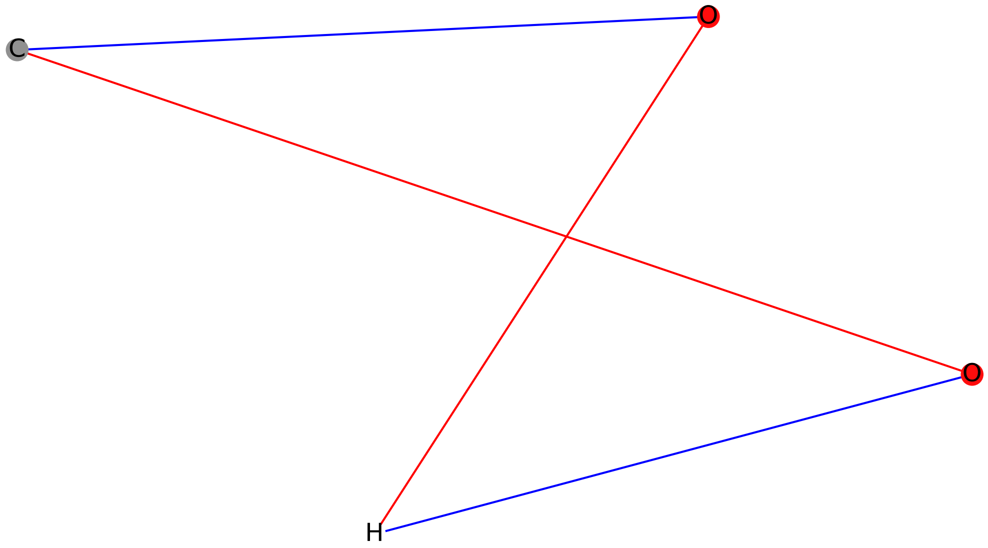

R-id = 29597 with reaction step = 2

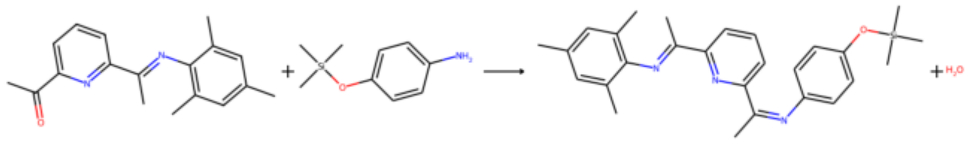


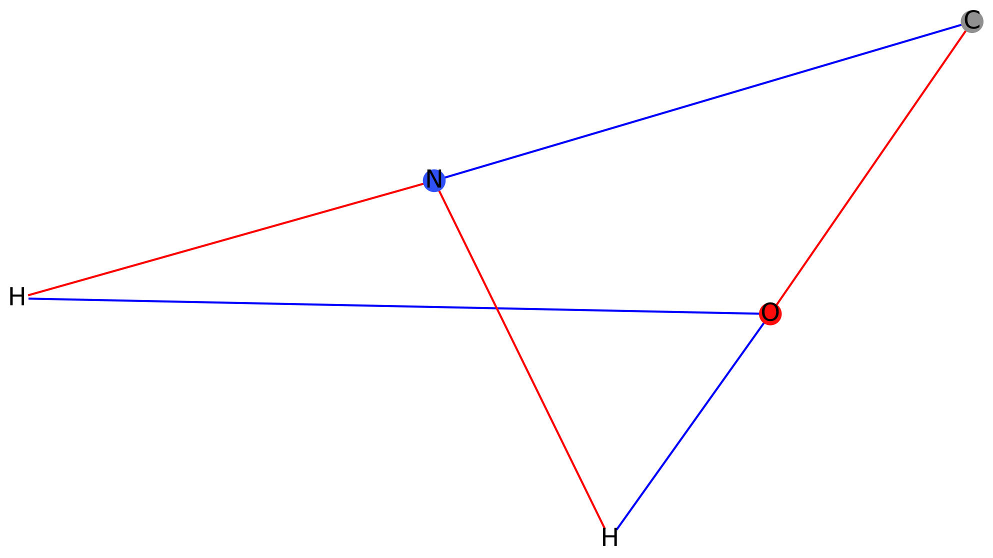

R-id = 38640 with reaction step = 1

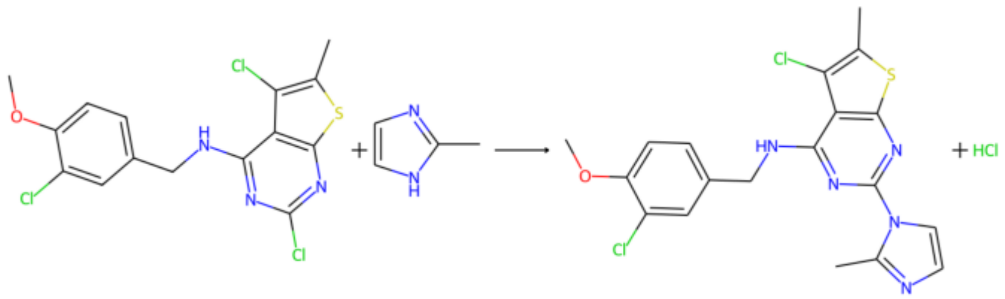

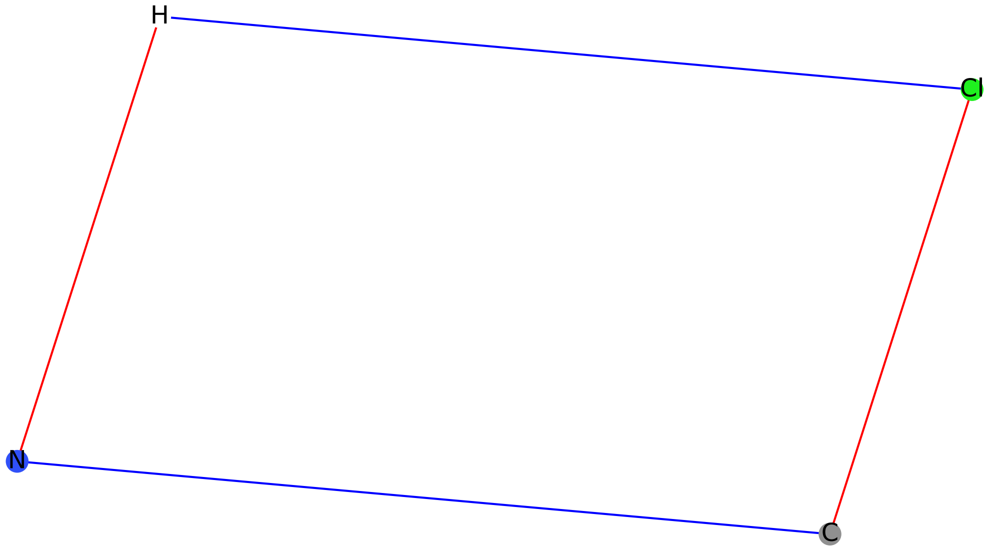

R-id = 31718 with reaction step = 2

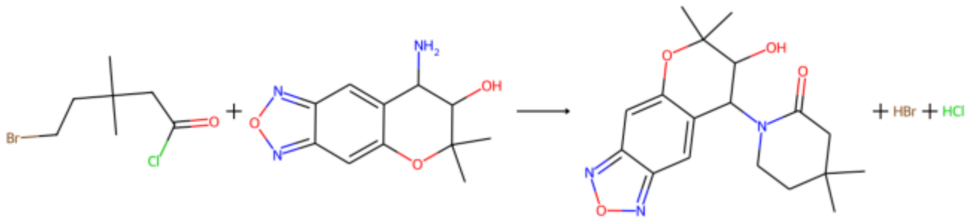


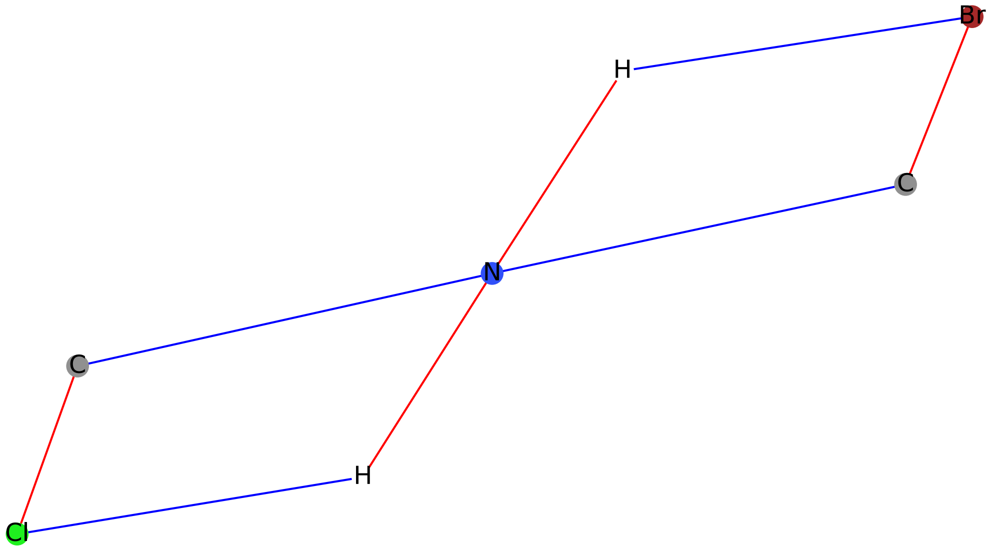

R-id = 39339 with reaction step = 1

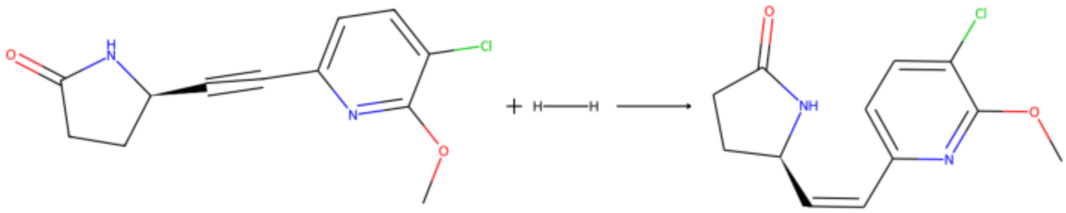


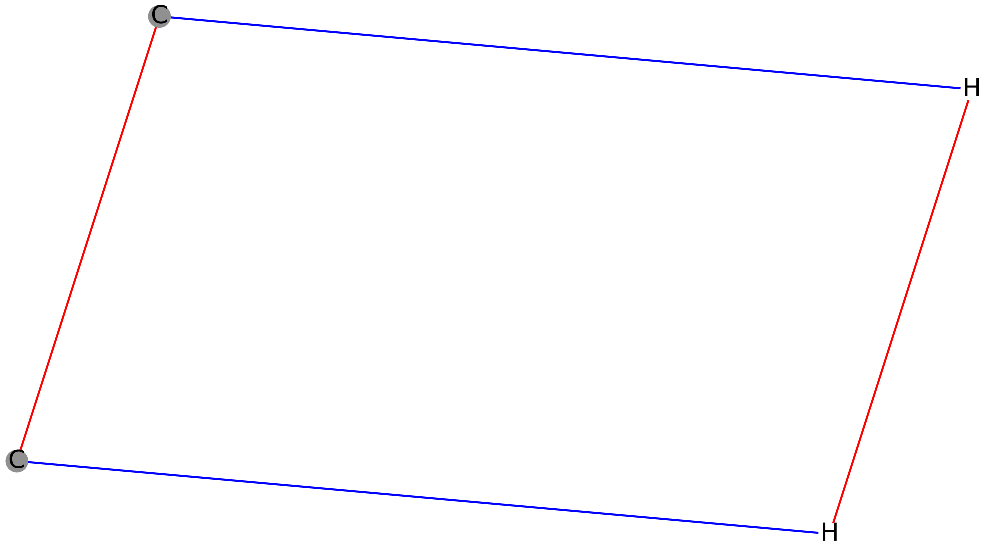

R-id = 22685 with reaction step = 1

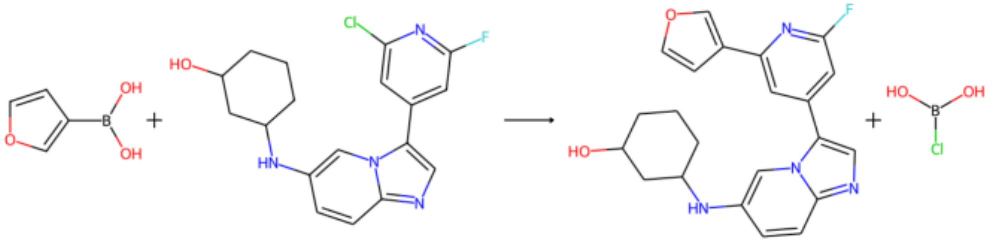


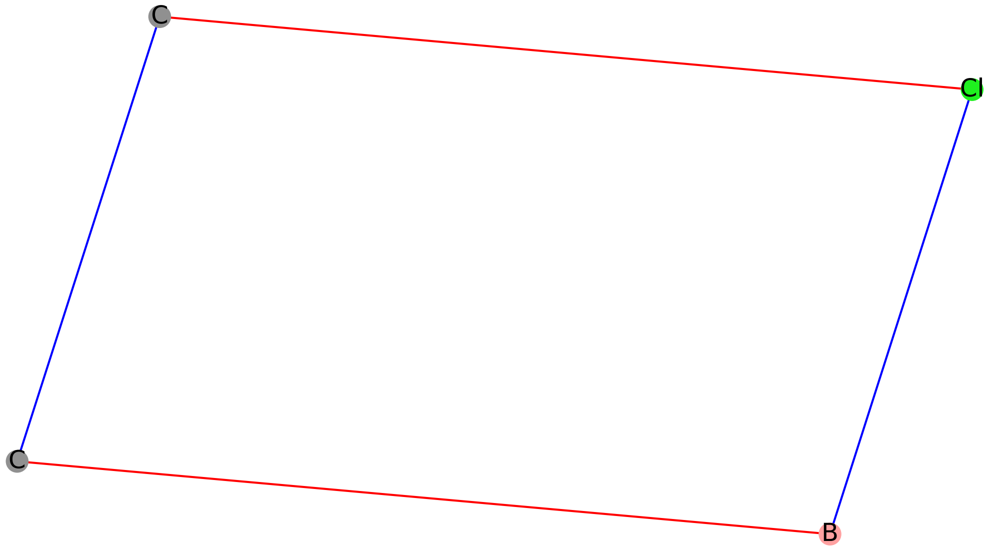

R-id = 25057 with reaction step = 4

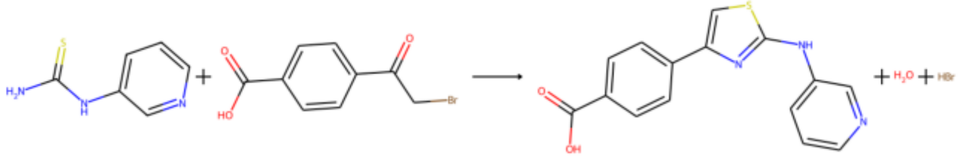


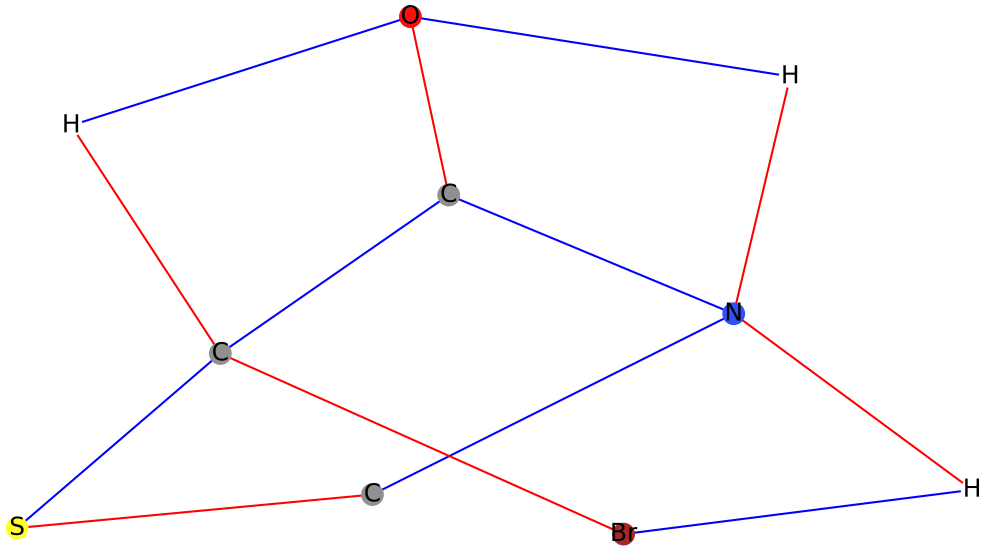

R-id = 11791 with reaction step = 2

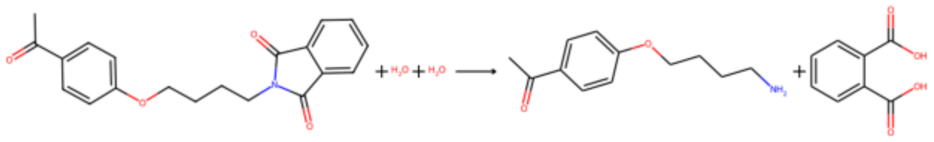

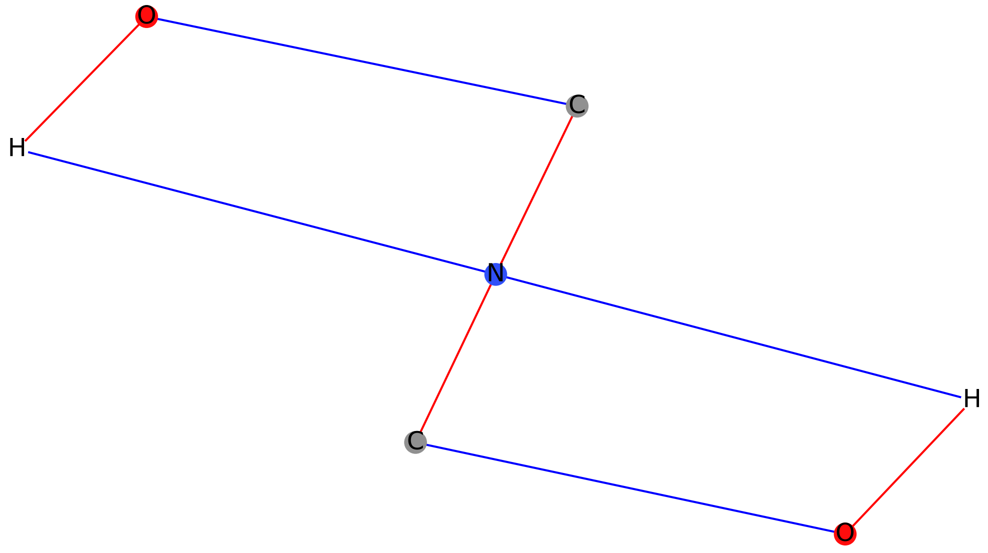

R-id = 36860 with reaction step = 3

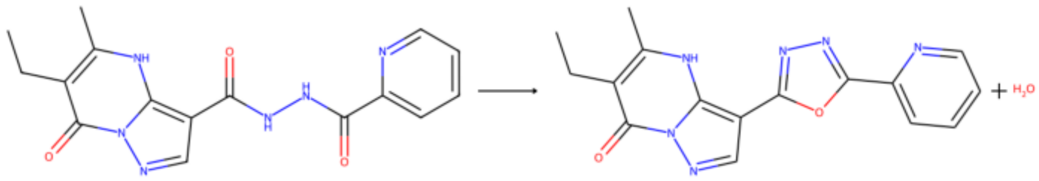


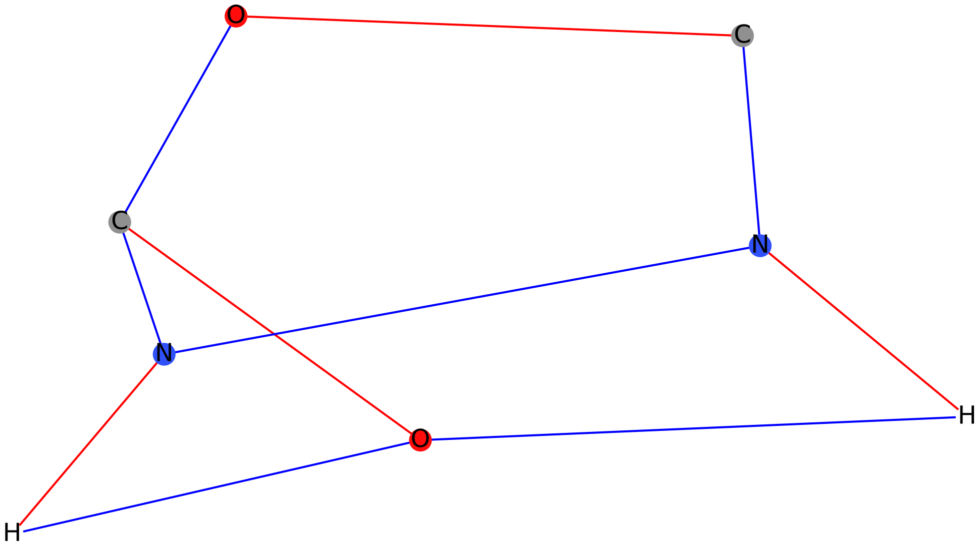

R-id = 37389 with reaction step = 2

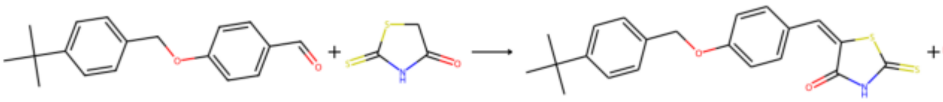

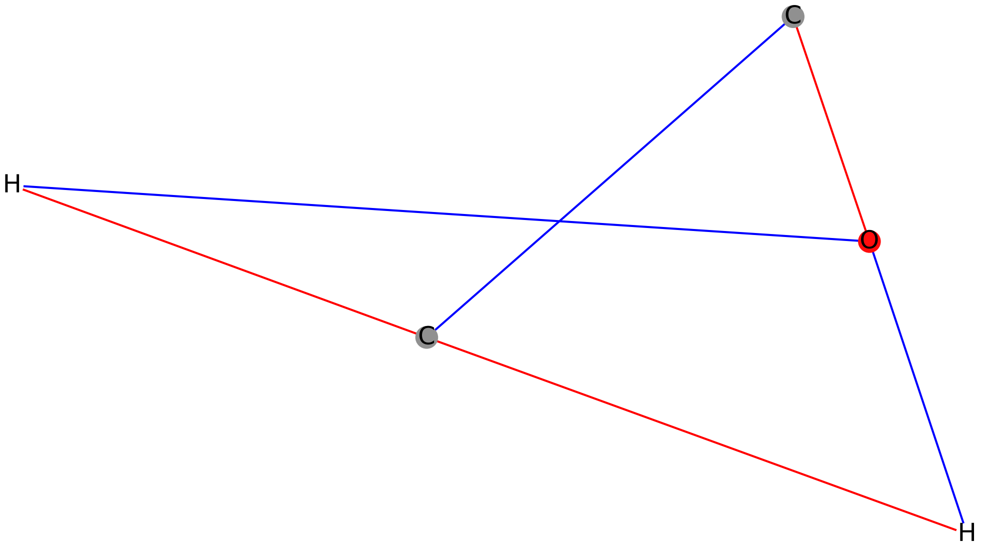

R-id = 35502 with reaction step = 1

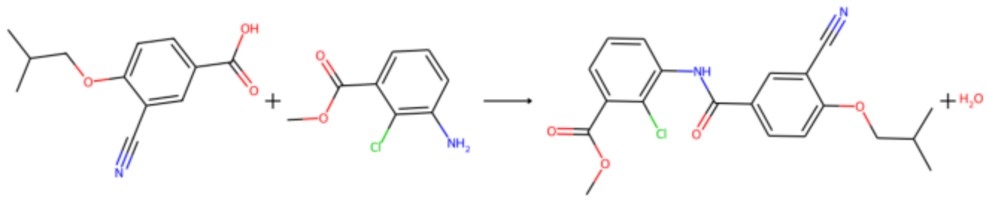


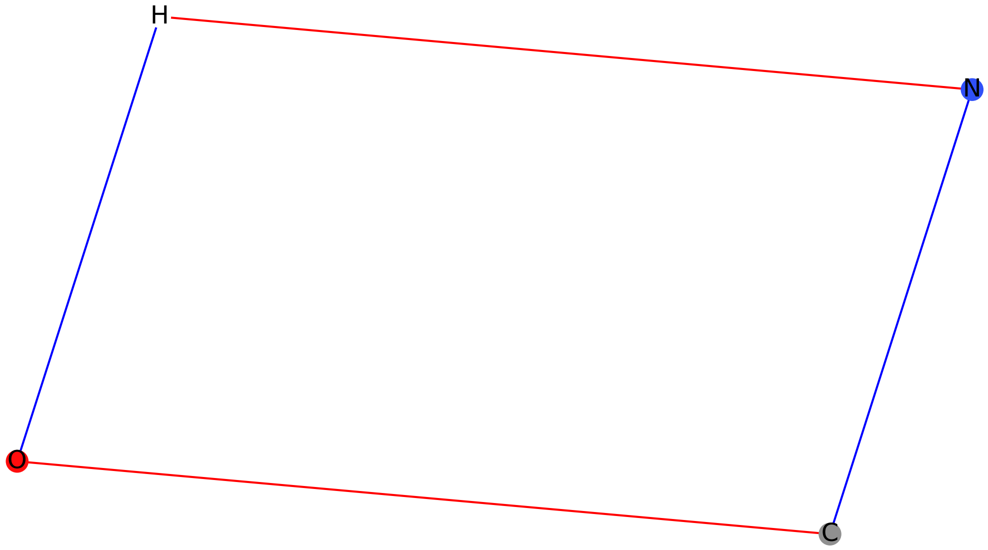

R-id = 11890 with reaction step = 3

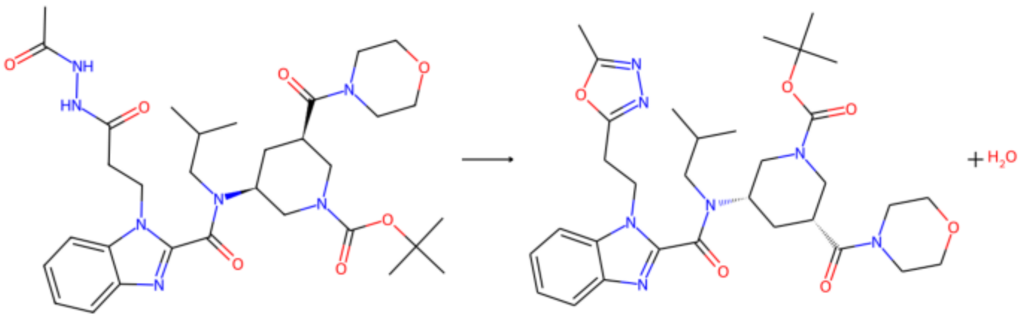

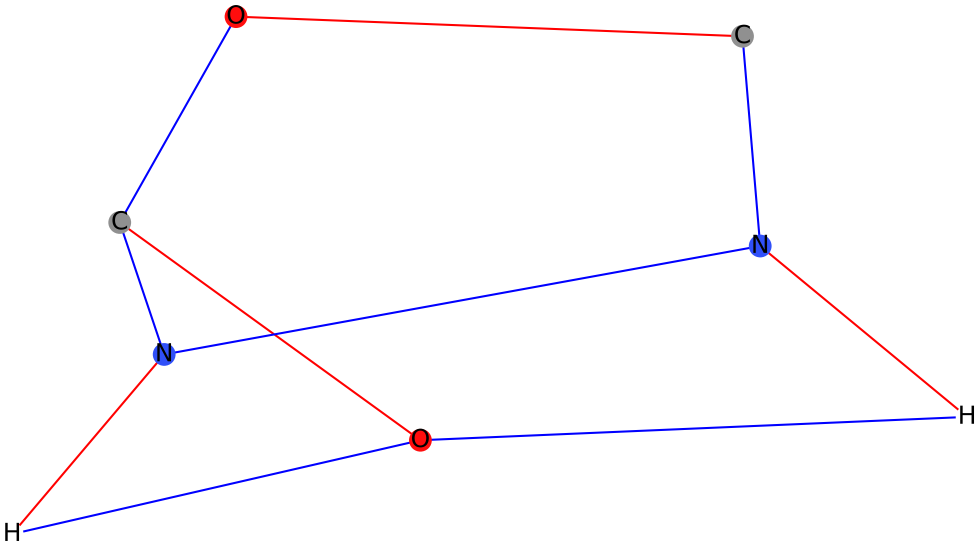

R-id = 20537 with reaction step = 1

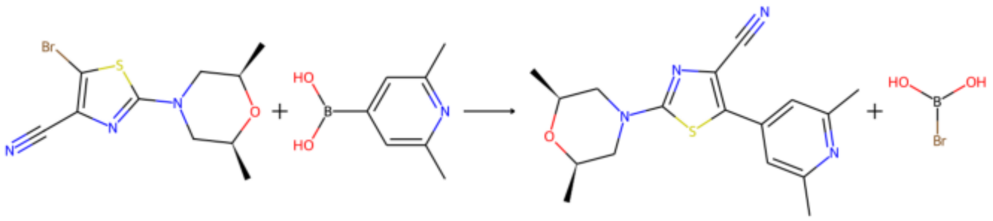


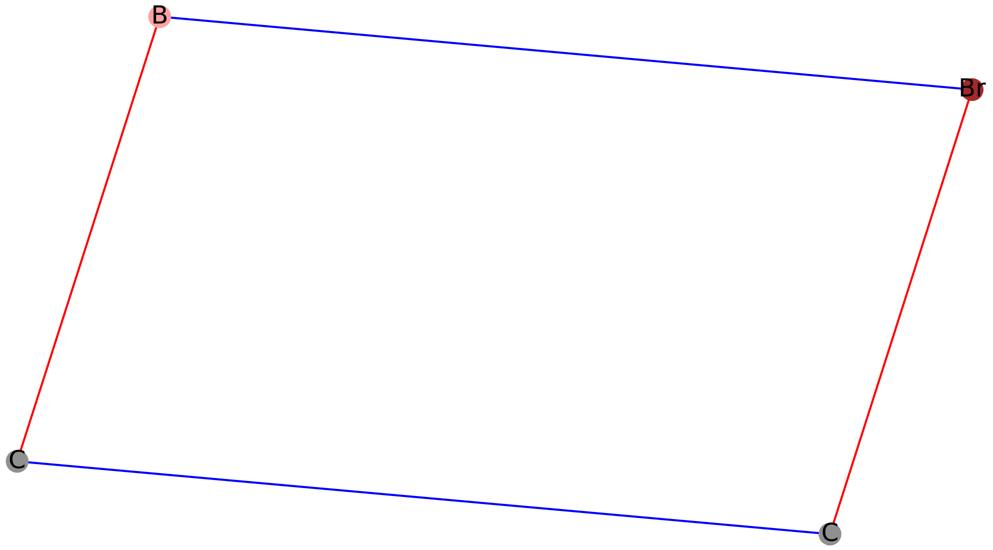

R-id = 27866 with reaction step = 1

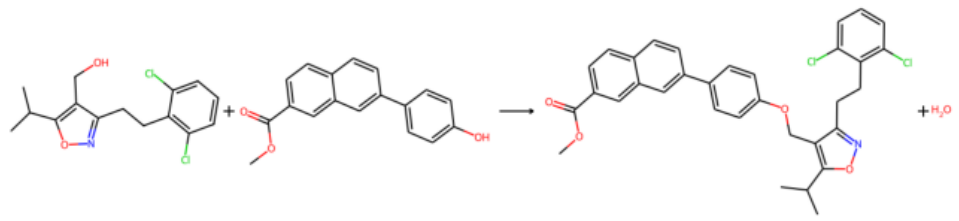


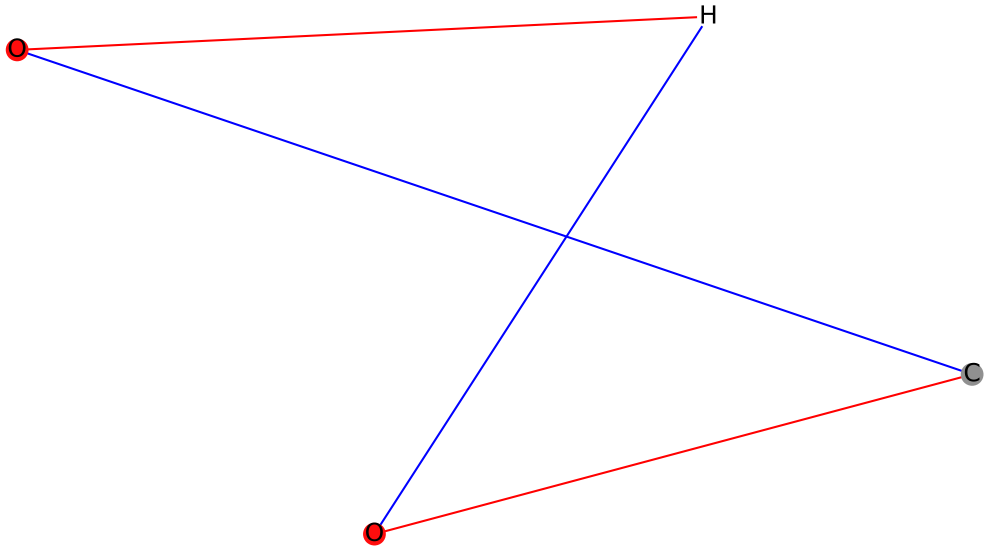

R-id = 41476 with reaction step = 4

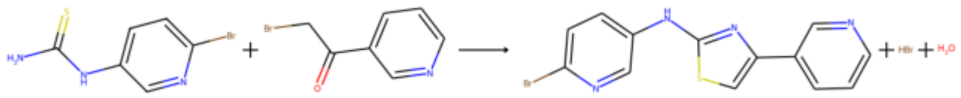

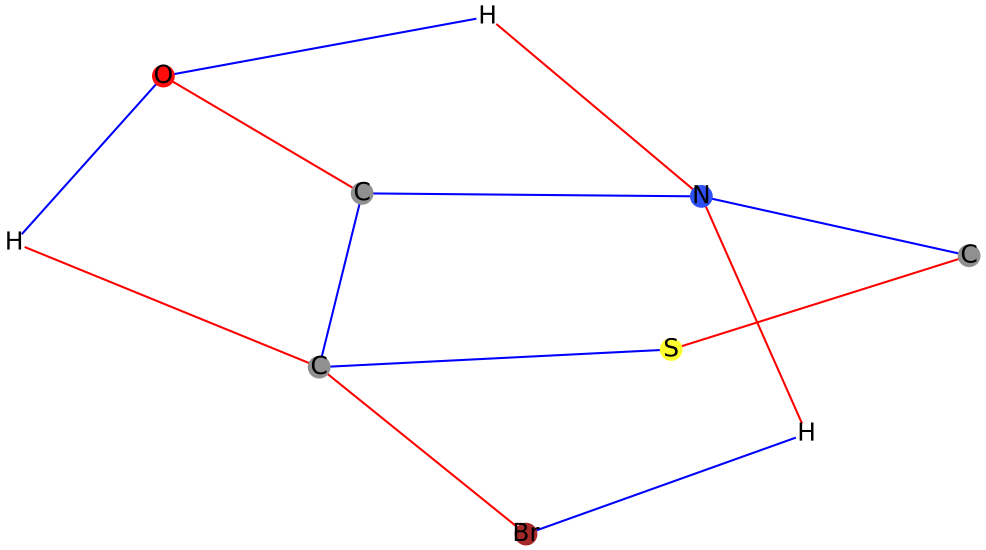

R-id = 21245 with reaction step = 2

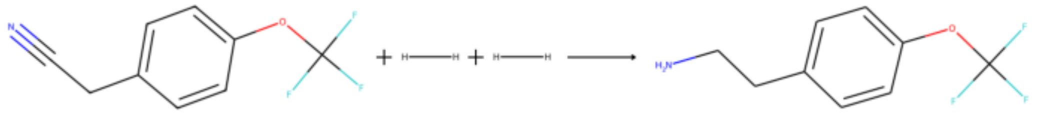


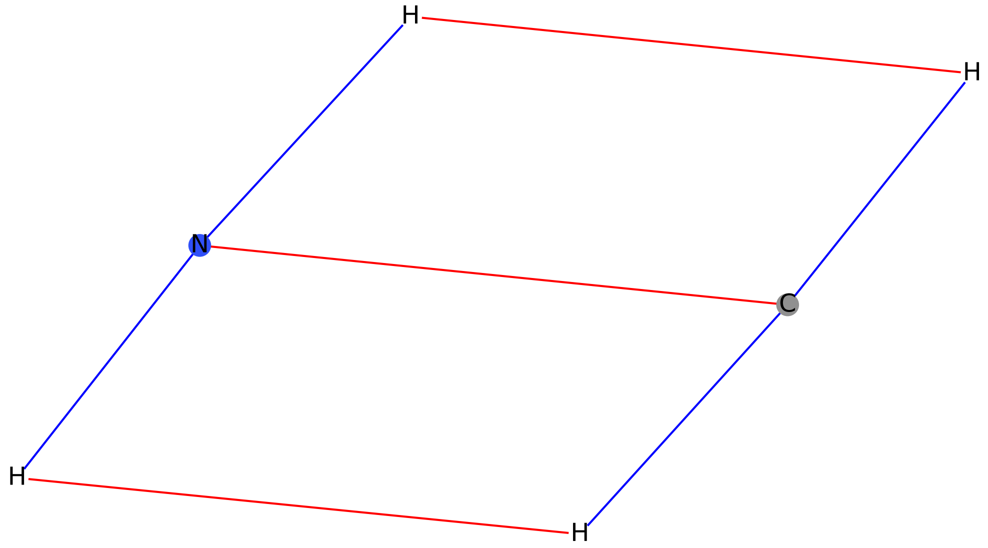

R-id = 3349 with reaction step = 1

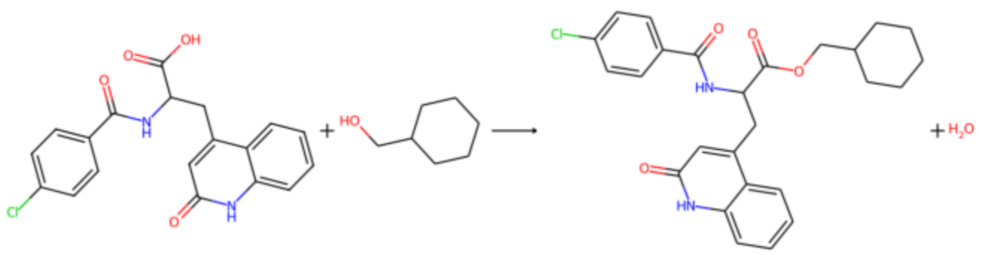


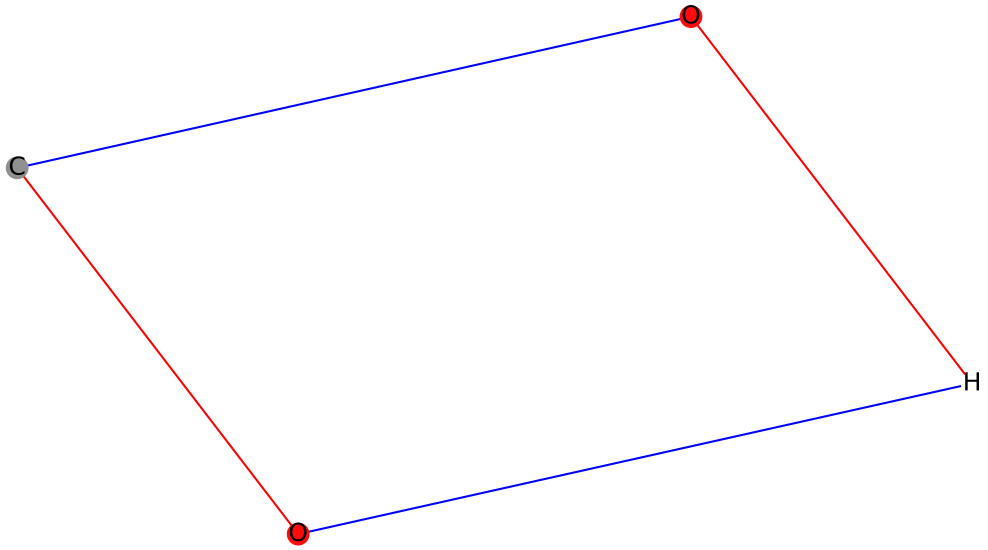

Supplement: Supplementary file 2 — ci4c01795_si_002.pdf [file ci4c01795_si_002.pdf]
