# Supplementary material for: SynTemp: Efficient Extraction of Graph-Based Reaction Rules from Large-Scale Reaction Databases
Source: J Chem Inf Model. 2025 Feb 28;65(6):2882–96. doi: 10.1021/acs.jcim.4c01795 (PMC11938280; doi:10.1021/acs.jcim.4c01795)
Supplement: Supplementary file 3 — ci4c01795_si_003.pdf [file ci4c01795_si_003.pdf]

Summary

September 16, 2024

Contents

0.0.1 0 8
0.0.2 1 8
0.0.3 2 9
0.0.4 3 10
0.0.5 4 10
0.0.6 5 11
0.0.7 6 12
0.0.8 7 12
0.0.9 8 13
0.0.10 9 14
0.0.11 10 14
0.0.12 11 15
0.0.13 12 15
0.0.14 13 16
0.0.15 14 16
0.0.16 15 17
0.0.17 16 17
0.0.18 17 18
0.0.19 18 18
0.0.20 19 19
0.0.21 20 19
0.0.22 21 20
0.0.23 22 20
0.0.24 23 21
0.0.25 24 22
0.0.26 25 22
0.0.27 26 23
0.0.28 27 23
0.0.29 28 24
0.0.30 29 25
0.0.31 30 25
0.0.32 31 26
0.0.33 32 26
0.0.34 33 27
0.0.35 34 28
0.0.36 35 28
0.0.37 36 29
0.0.38 37 29
0.0.39 38 30
0.0.40 39 31
0.0.41 40 31

0.0.42 41 32
0.0.43 42 33
0.0.44 43 33
0.0.45 44 34
0.0.46 45 35
0.0.47 46 35
0.0.48 47 36
0.0.49 48 36
0.0.50 49 37
0.0.51 50 38
0.0.52 51 38
0.0.53 52 39
0.0.54 53 39
0.0.55 54 40
0.0.56 55 40
0.0.57 56 41
0.0.58 57 42
0.0.59 58 42
0.0.60 59 43
0.0.61 60 44
0.0.62 61 44
0.0.63 62 45
0.0.64 63 46
0.0.65 64 46
0.0.66 65 47
0.0.67 66 48
0.0.68 67 48
0.0.69 68 49
0.0.70 69 50
0.0.71 70 50
0.0.72 71 51
0.0.73 72 52
0.0.74 73 52
0.0.75 74 53
0.0.76 75 53
0.0.77 76 54
0.0.78 77 54
0.0.79 78 55
0.0.80 79 56
0.0.81 80 56
0.0.82 81 57
0.0.83 82 58
0.0.84 83 58
0.0.85 84 59
0.0.86 85 59
0.0.87 86 60
0.0.88 87 60
0.0.89 88 61
0.0.90 89 62
0.0.91 90 62

0.0.92 91 . . . 63
0.0.93 92 . . . 64
0.0.94 93 . . . 64
0.0.95 94 . . . 65
0.0.96 95 . . . 65
0.0.97 96 . . . 66
0.0.98 97 . . . 66
0.0.99 98 . . . 67
0.0.100 99 . . . 68
0.0.101 100 . . . 68
0.0.102 101 . . . 69
0.0.103 102 . . . 70
0.0.104 103 . . . 70
0.0.105 104 . . . 71
0.0.106 105 . . . 71
0.0.107 106 . . . 72
0.0.108 107 . . . 72
0.0.109 108 . . . 73
0.0.110 109 . . . 73
0.0.111 110 . . . 74
0.0.112 111 . . . 74
0.0.113 112 . . . 75
0.0.114 113 . . . 75
0.0.115 114 . . . 76
0.0.116 115 . . . 76
0.0.117 116 . . . 77
0.0.118 117 . . . 78
0.0.119 118 . . . 78
0.0.120 119 . . . 79
0.0.121 120 . . . 79
0.0.122 121 . . . 80
0.0.123 122 . . . 81
0.0.124 123 . . . 81
0.0.125 124 . . . 82
0.0.126 125 . . . 83
0.0.127 126 . . . 83
0.0.128 127 . . . 84
0.0.129 128 . . . 85
0.0.130 129 . . . 85
0.0.131 130 . . . 86
0.0.132 131 . . . 87
0.0.133 132 . . . 87
0.0.134 133 . . . 88
0.0.135 134 . . . 89
0.0.136 135 . . . 89
0.0.137 136 . . . 90
0.0.138 137 . . . 90
0.0.139 138 . . . 91
0.0.140 139 . . . 91
0.0.141 140 . . . 92

0.0.142 141 92
0.0.143 142 93
0.0.144 143 94
0.0.145 144 94
0.0.146 145 95
0.0.147 146 95
0.0.148 147 96
0.0.149 148 96
0.0.150 149 97
0.0.151 150 98
0.0.152 151 98
0.0.153 152 99
0.0.154 153 99
0.0.155 154 100
0.0.156 155 101
0.0.157 156 101
0.0.158 157 102
0.0.159 158 103
0.0.160 159 103
0.0.161 160 104
0.0.162 161 105
0.0.163 162 105
0.0.164 163 106
0.0.165 164 106
0.0.166 165 107
0.0.167 166 108
0.0.168 167 108
0.0.169 168 109
0.0.170 169 109
0.0.171 170 110
0.0.172 171 110
0.0.173 172 111
0.0.174 173 112
0.0.175 174 112
0.0.176 175 113
0.0.177 176 113
0.0.178 177 114
0.0.179 178 115
0.0.180 179 115
0.0.181 180 116
0.0.182 181 117
0.0.183 182 117
0.0.184 183 118
0.0.185 184 118
0.0.186 185 119
0.0.187 186 119
0.0.188 187 120
0.0.189 188 120
0.0.190 189 121
0.0.191 190 121

0.0.192 191 . . . 122
0.0.193 192 . . . 122
0.0.194 193 . . . 123
0.0.195 194 . . . 124
0.0.196 195 . . . 124
0.0.197 196 . . . 125
0.0.198 197 . . . 125
0.0.199 198 . . . 126
0.0.200 199 . . . 126
0.0.201 200 . . . 127
0.0.202 201 . . . 127
0.0.203 202 . . . 128
0.0.204 203 . . . 128
0.0.205 204 . . . 129
0.0.206 205 . . . 129
0.0.207 206 . . . 130
0.0.208 207 . . . 131
0.0.209 208 . . . 131
0.0.210 209 . . . 132
0.0.211 210 . . . 132
0.0.212 211 . . . 133
0.0.213 212 . . . 133
0.0.214 213 . . . 134
0.0.215 214 . . . 134
0.0.216 215 . . . 135
0.0.217 216 . . . 135
0.0.218 217 . . . 136
0.0.219 218 . . . 137
0.0.220 219 . . . 137
0.0.221 220 . . . 138
0.0.222 221 . . . 138
0.0.223 222 . . . 139
0.0.224 223 . . . 139
0.0.225 224 . . . 140
0.0.226 225 . . . 140
0.0.227 226 . . . 141
0.0.228 227 . . . 141
0.0.229 228 . . . 142
0.0.230 229 . . . 142
0.0.231 230 . . . 143
0.0.232 231 . . . 143
0.0.233 232 . . . 144
0.0.234 233 . . . 145
0.0.235 234 . . . 145
0.0.236 235 . . . 146
0.0.237 236 . . . 147
0.0.238 237 . . . 147
0.0.239 238 . . . 148
0.0.240 239 . . . 148
0.0.241 240 . . . 149

0.0.242 241 . . . 149
0.0.243 242 . . . 150
0.0.244 243 . . . 150
0.0.245 244 . . . 151
0.0.246 245 . . . 151
0.0.247 246 . . . 152
0.0.248 247 . . . 152
0.0.249 248 . . . 153
0.0.250 249 . . . 153
0.0.251 250 . . . 153
0.0.252 251 . . . 154
0.0.253 252 . . . 154
0.0.254 253 . . . 155
0.0.255 254 . . . 155
0.0.256 255 . . . 156
0.0.257 256 . . . 157
0.0.258 257 . . . 157
0.0.259 258 . . . 158
0.0.260 259 . . . 158
0.0.261 260 . . . 158
0.0.262 261 . . . 159
0.0.263 262 . . . 160
0.0.264 263 . . . 160
0.0.265 264 . . . 161
0.0.266 265 . . . 162
0.0.267 266 . . . 162
0.0.268 267 . . . 163
0.0.269 268 . . . 163
0.0.270 269 . . . 164
0.0.271 270 . . . 165
0.0.272 271 . . . 165
0.0.273 272 . . . 166
0.0.274 273 . . . 166
0.0.275 274 . . . 167
0.0.276 275 . . . 167
0.0.277 276 . . . 168
0.0.278 277 . . . 168
0.0.279 278 . . . 169
0.0.280 279 . . . 169
0.0.281 280 . . . 170
0.0.282 281 . . . 171
0.0.283 282 . . . 171
0.0.284 283 . . . 172
0.0.285 284 . . . 172
0.0.286 285 . . . 173
0.0.287 286 . . . 173
0.0.288 287 . . . 174
0.0.289 288 . . . 174
0.0.290 289 . . . 175
0.0.291 290 . . . 176

0.0.292 291 . . . 176
0.0.293 292 . . . 177
0.0.294 293 . . . 177
0.0.295 294 . . . 178
0.0.296 295 . . . 179
0.0.297 296 . . . 179
0.0.298 297 . . . 180
0.0.299 298 . . . 180
0.0.300 299 . . . 181
0.0.301 300 . . . 181
0.0.302 301 . . . 182
0.0.303 302 . . . 183
0.0.304 303 . . . 183
0.0.305 304 . . . 184
0.0.306 305 . . . 184
0.0.307 306 . . . 185
0.0.308 307 . . . 185
0.0.309 308 . . . 186
0.0.310 309 . . . 187
0.0.311 310 . . . 187

0.0.1 0

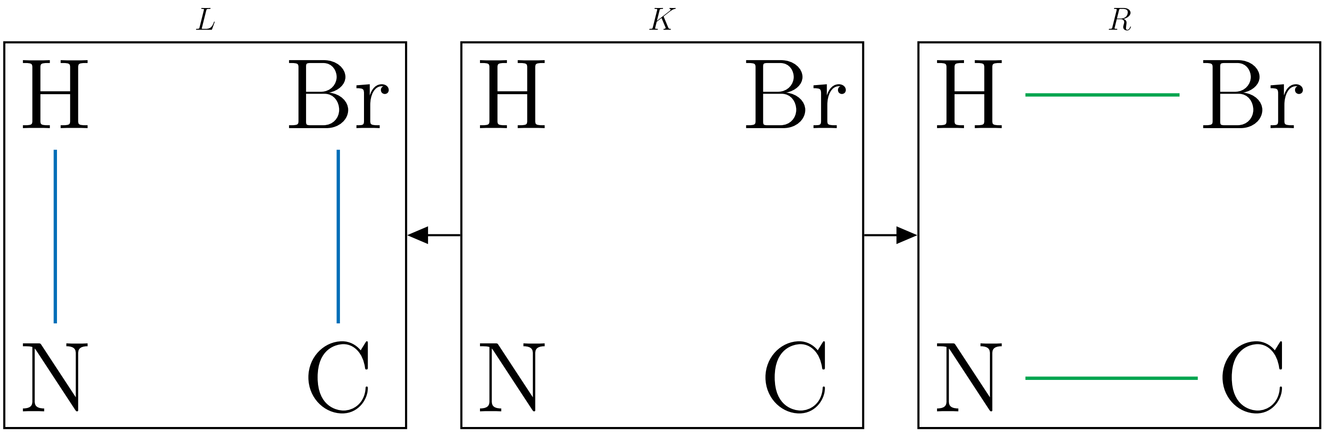

Files: out/001_r_0_10300000_{L, K, R}

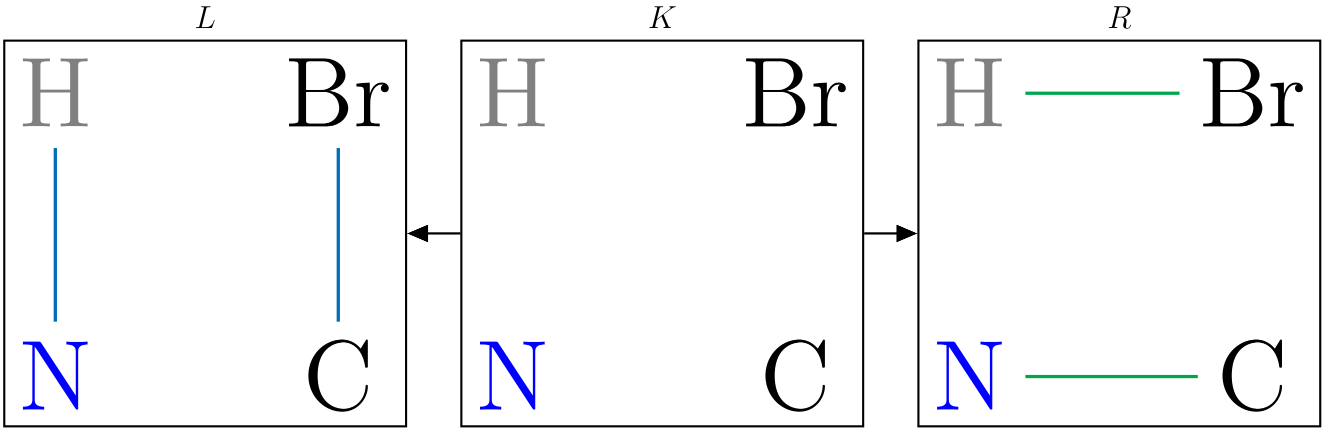

Files: out/003_r_0_11300100_{L, K, R}

0.0.2 1

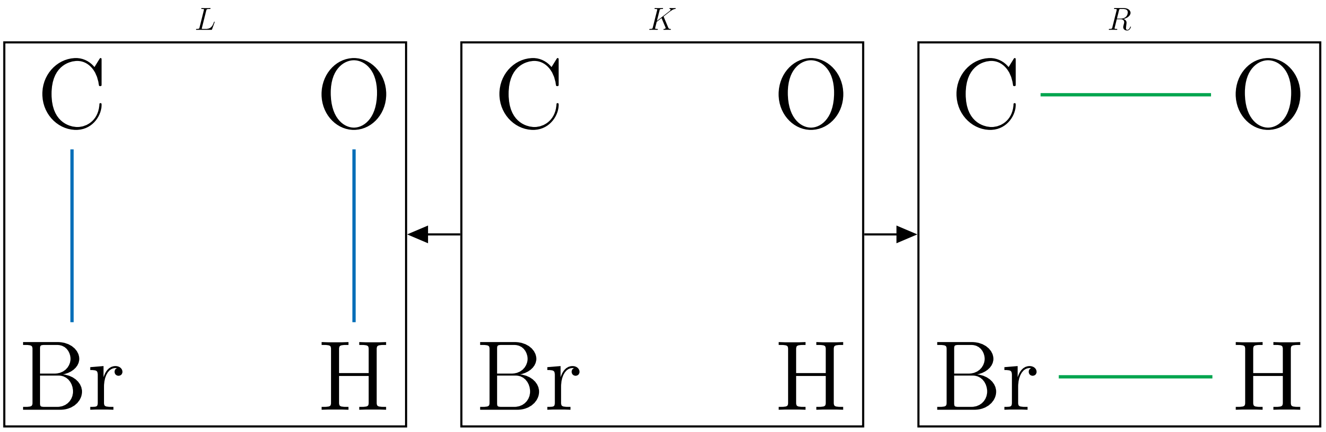

Files: out/006_r_1_10300000_{L, K, R}

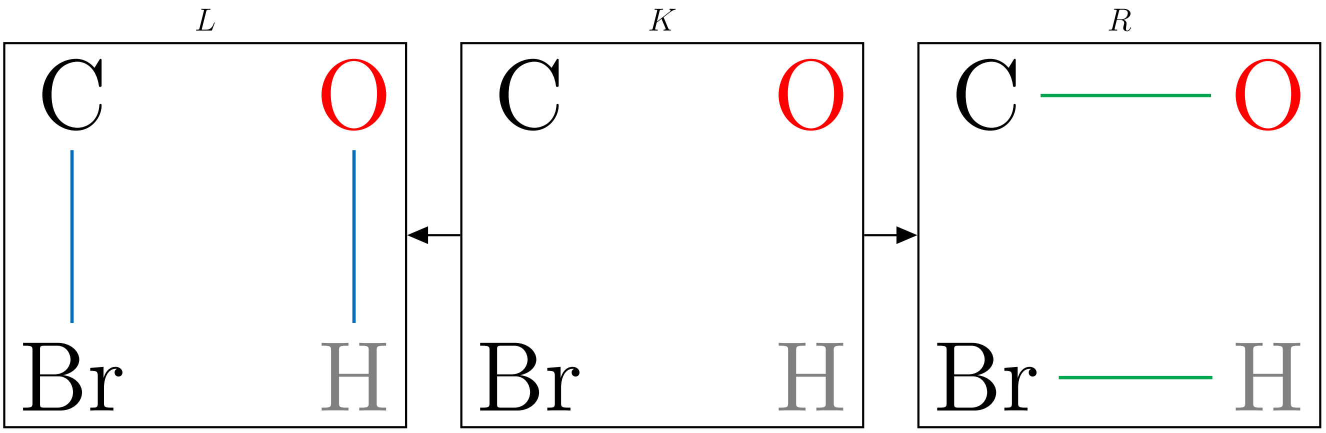

Files: out/008_r_1_11300100_{L, K, R}

0.0.3 2

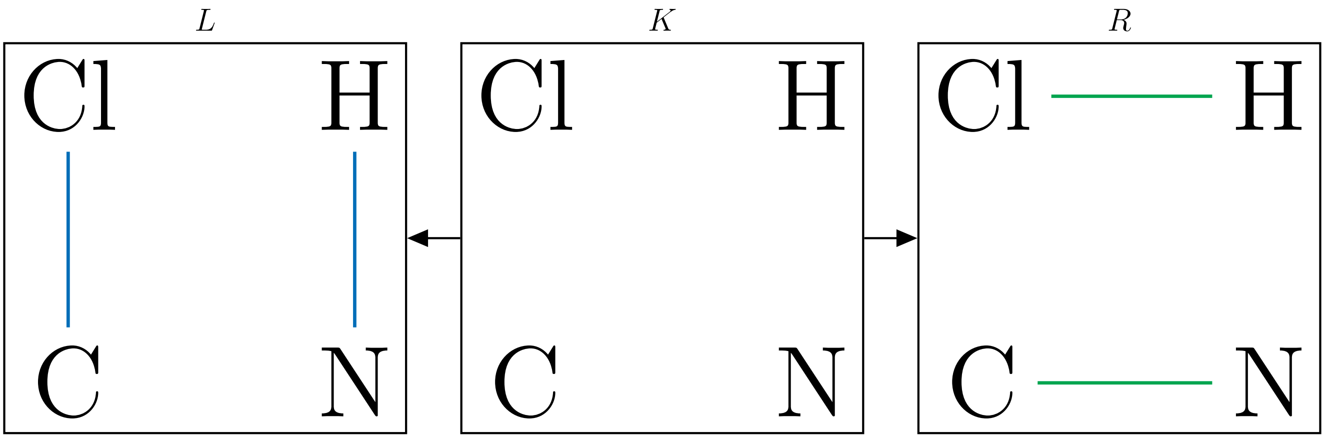

Files: out/011_r_2_10300000_{L, K, R}

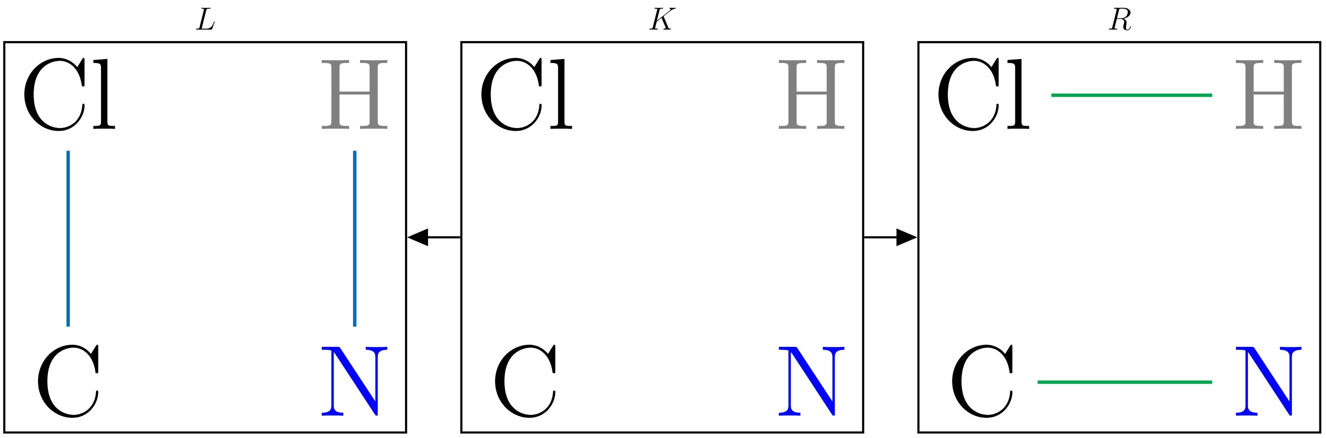

Files: out/013_r_2_11300100_{L, K, R}

0.0.4 3

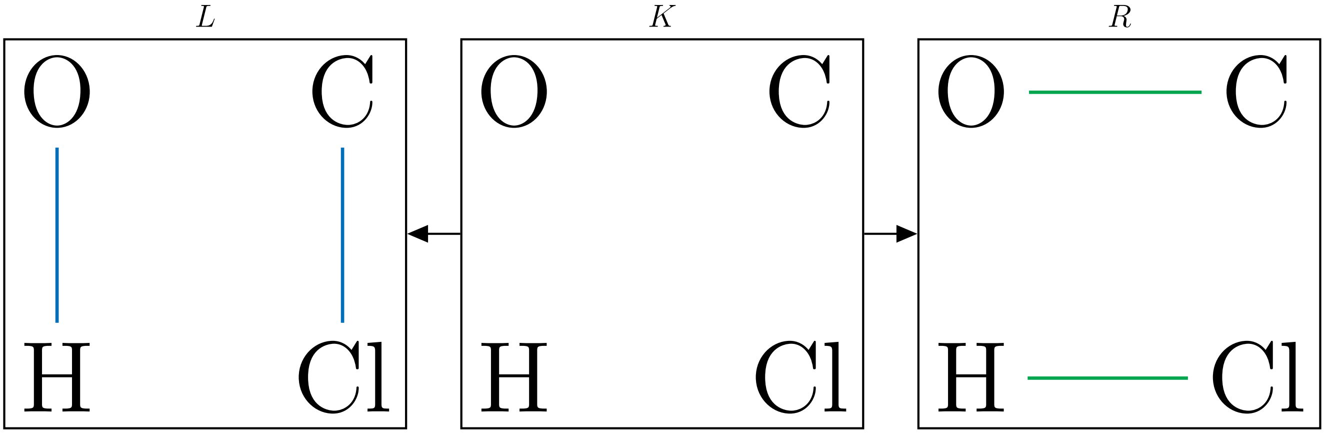

Files: out/016_r_3_10300000_{L, K, R}

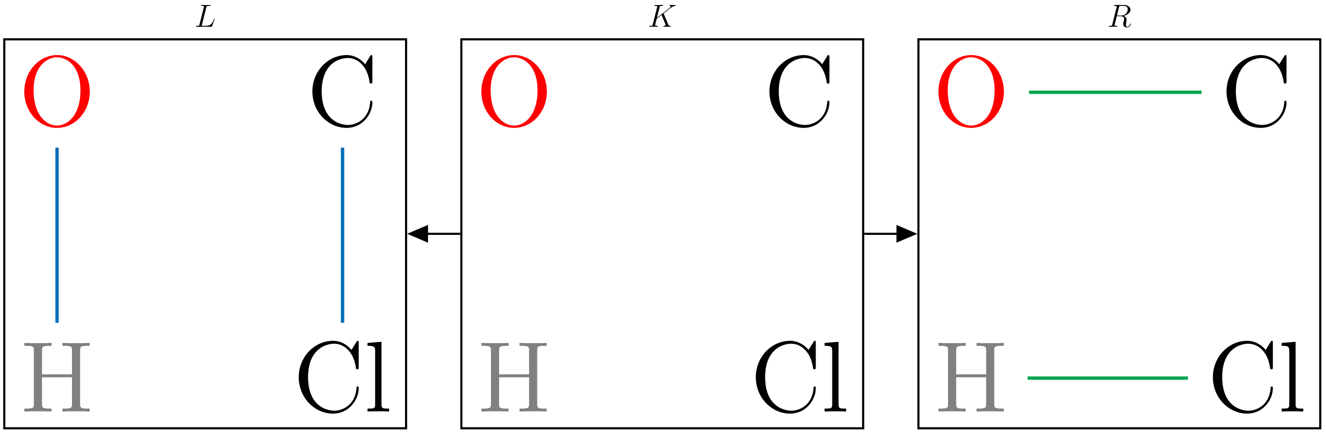

Files: out/018_r_3_11300100_{L, K, R}

0.0.5 4

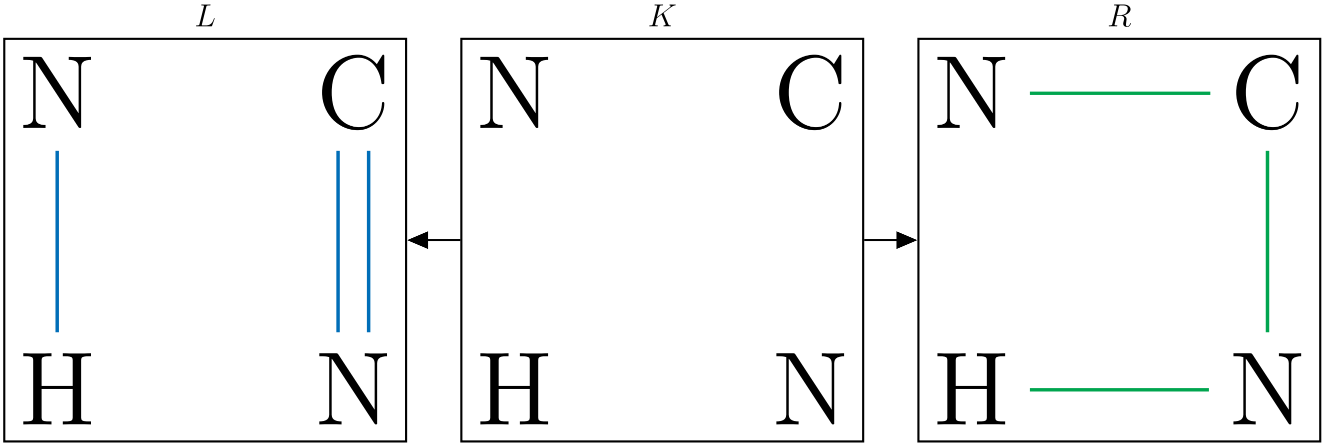

Files: out/021_r_4_10300000_{L, K, R}

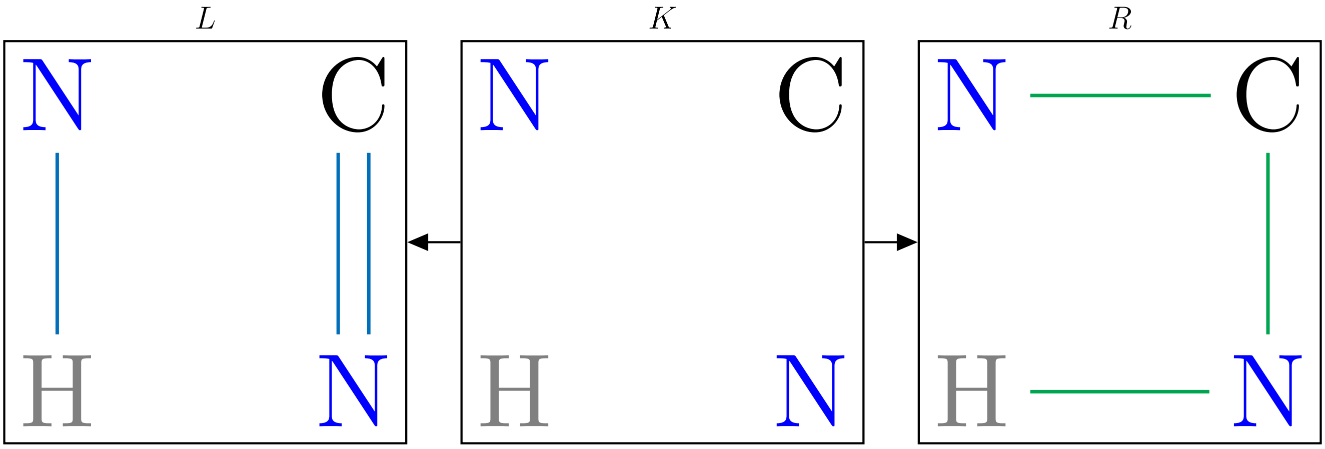

Files: out/023_r_4_11300100_{L, K, R}

0.0.6 5

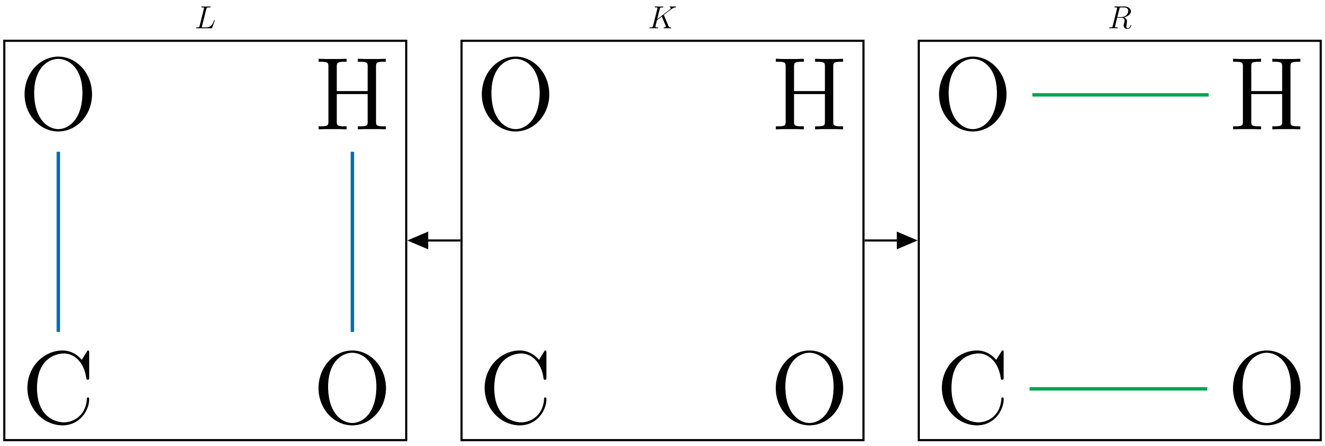

Files: out/026_r_5_10300000_{L, K, R}

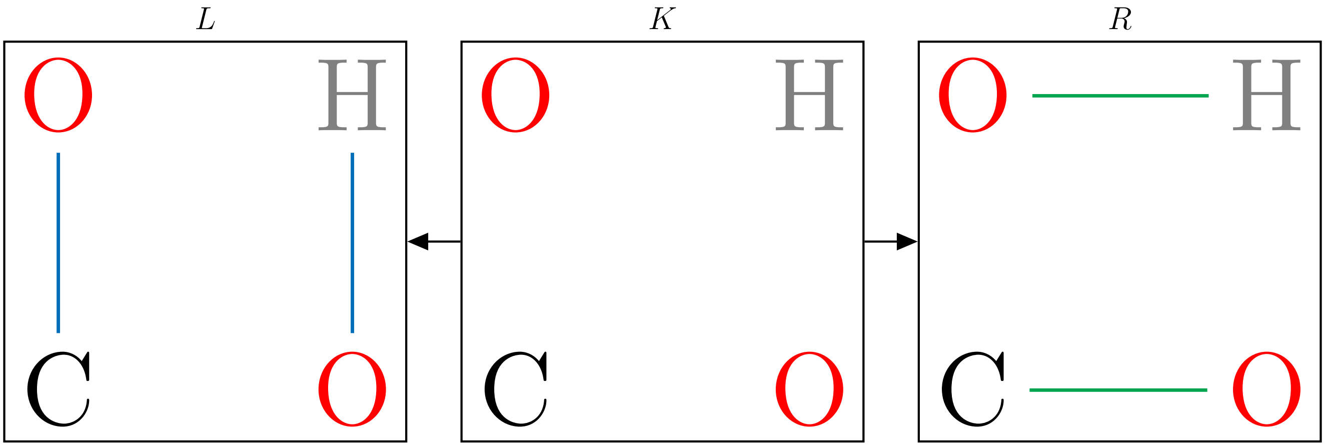

Files: out/028_r_5_11300100_{L, K, R}

0.0.7 6

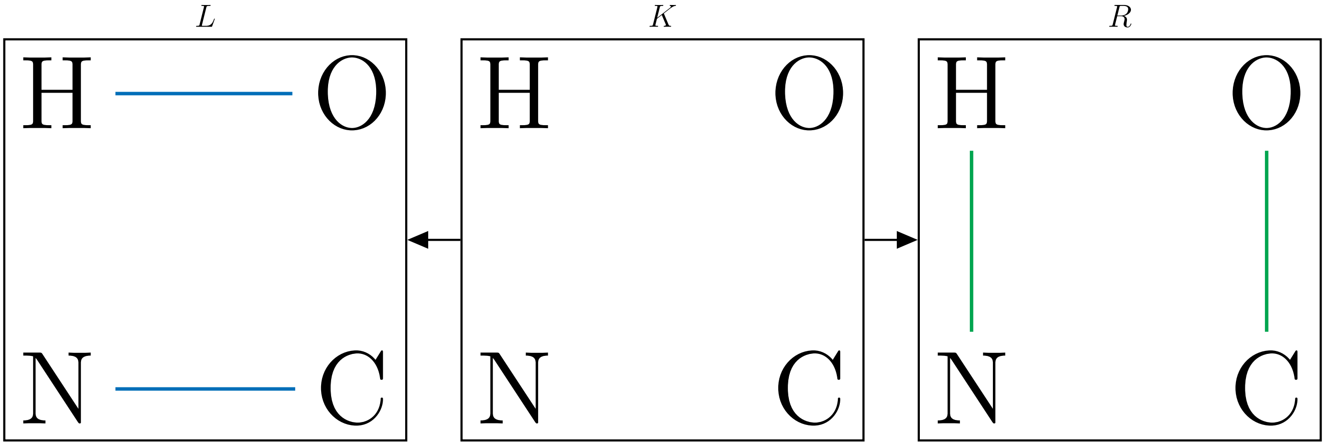

Files: out/031_r_6_10300000_{L, K, R}

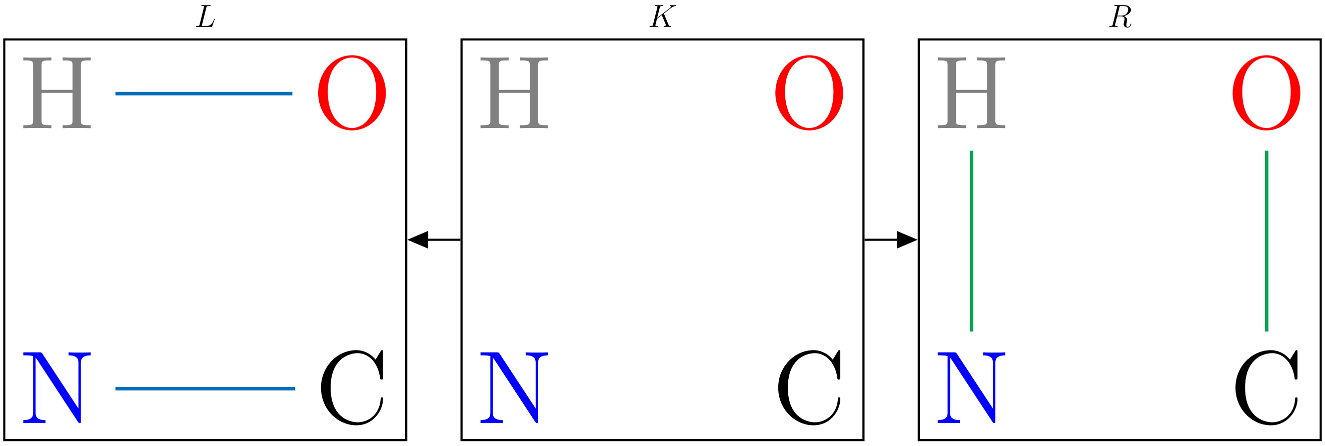

Files: out/033_r_6_11300100_{L, K, R}

0.0.8 7

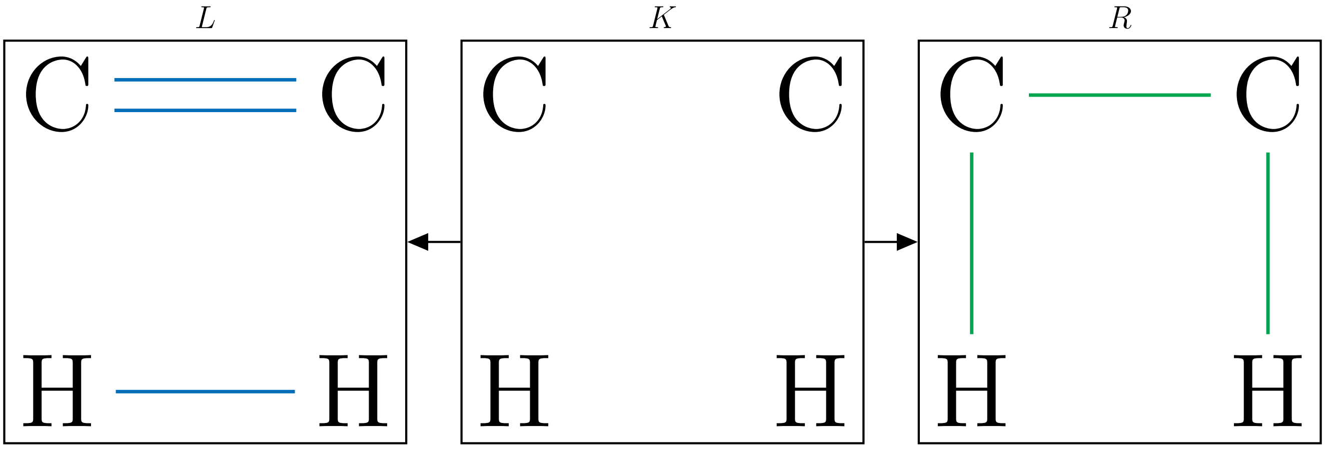

Files: out/036_r_7_10300000_{L, K, R}

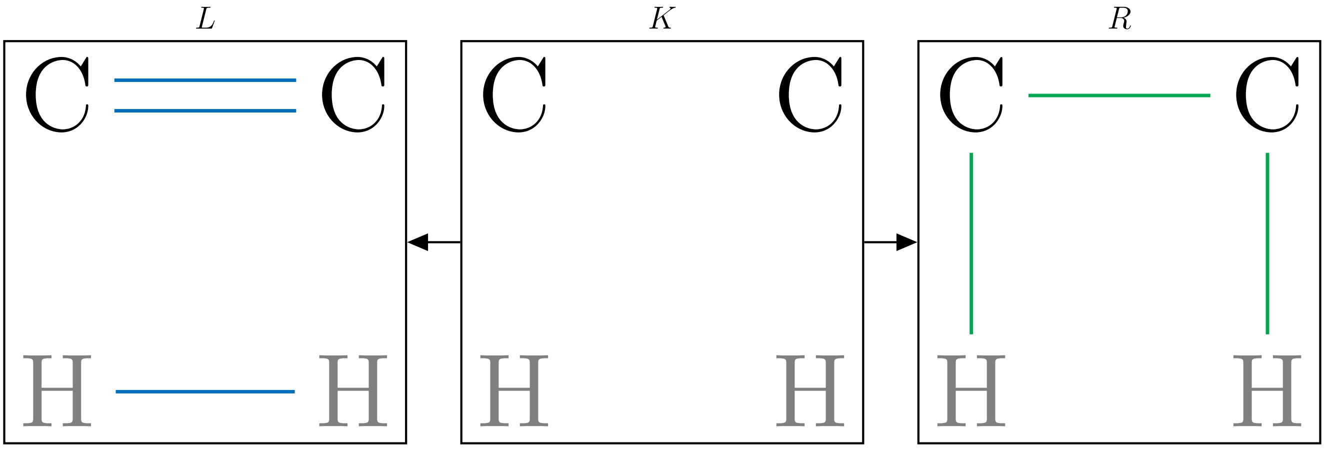

Files: out/038_r_7_11300100_{L, K, R}

0.0.9 8

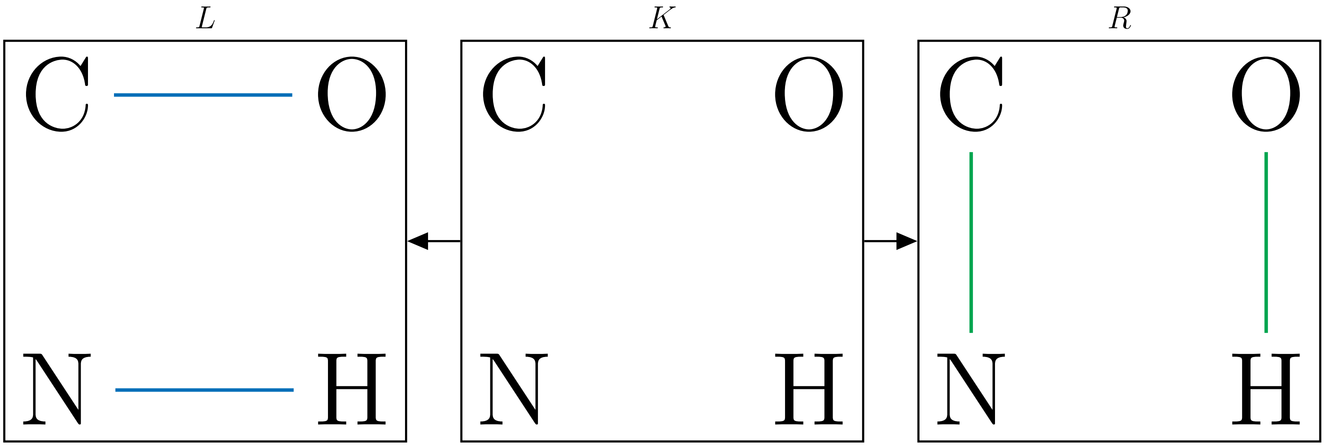

Files: out/041_r_8_10300000_{L, K, R}

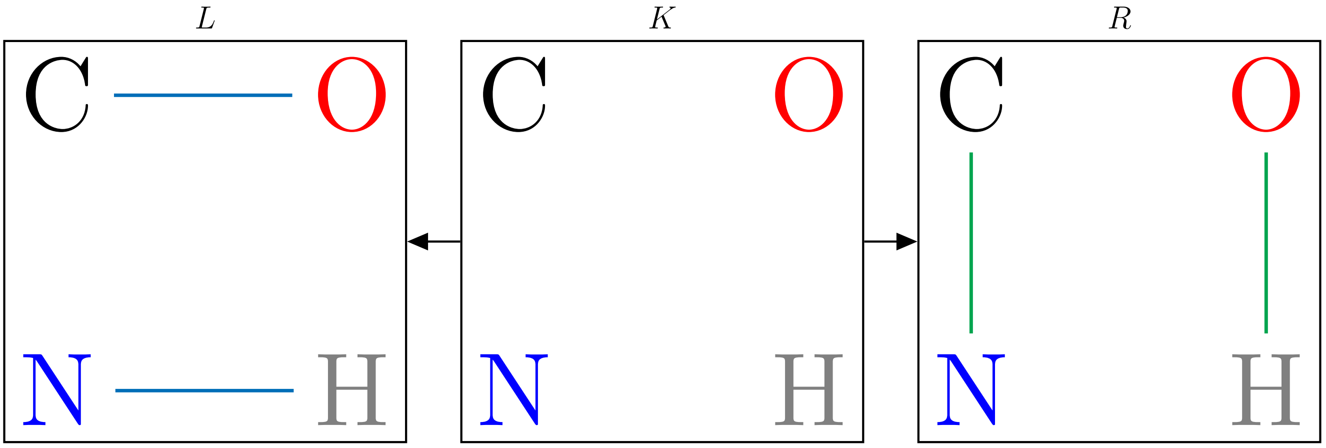

Files: out/043_r_8_11300100_{L, K, R}

0.0.10 9

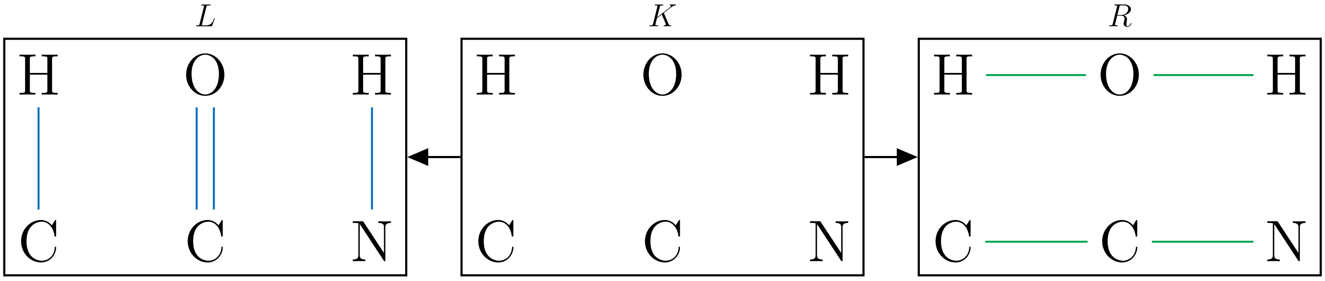

Files: out/046_r_9_10300000_{L, K, R}

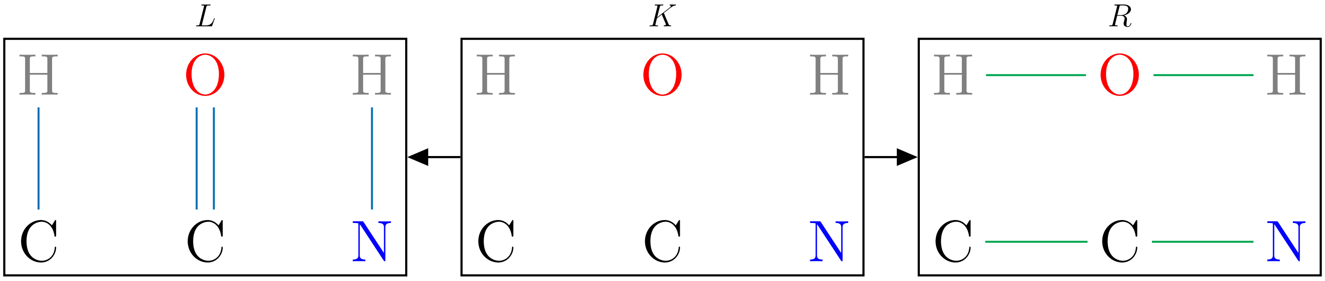

Files: out/048_r_9_11300100_{L, K, R}

0.0.11 10

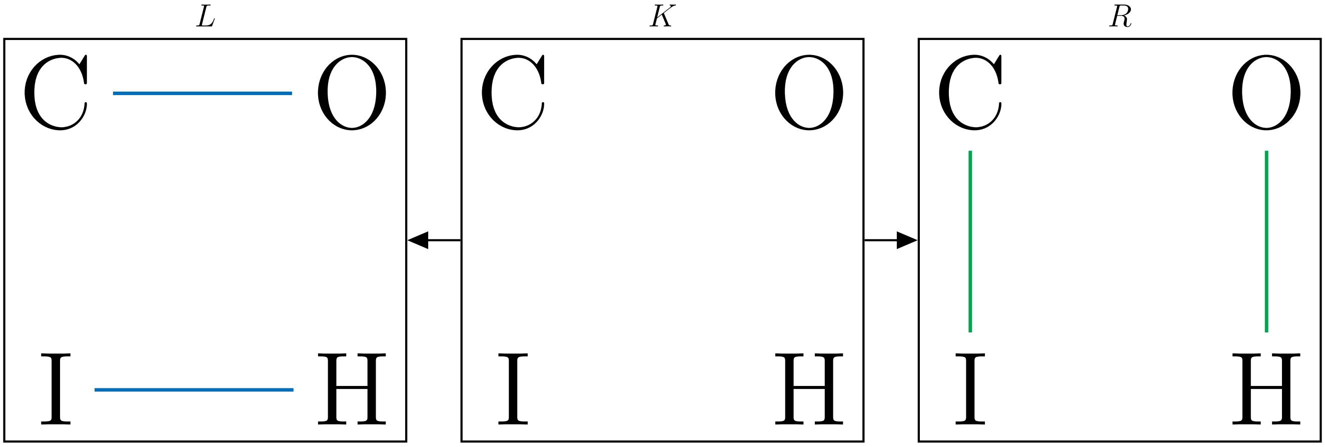

Files: out/051_r_10_10300000_{L, K, R}

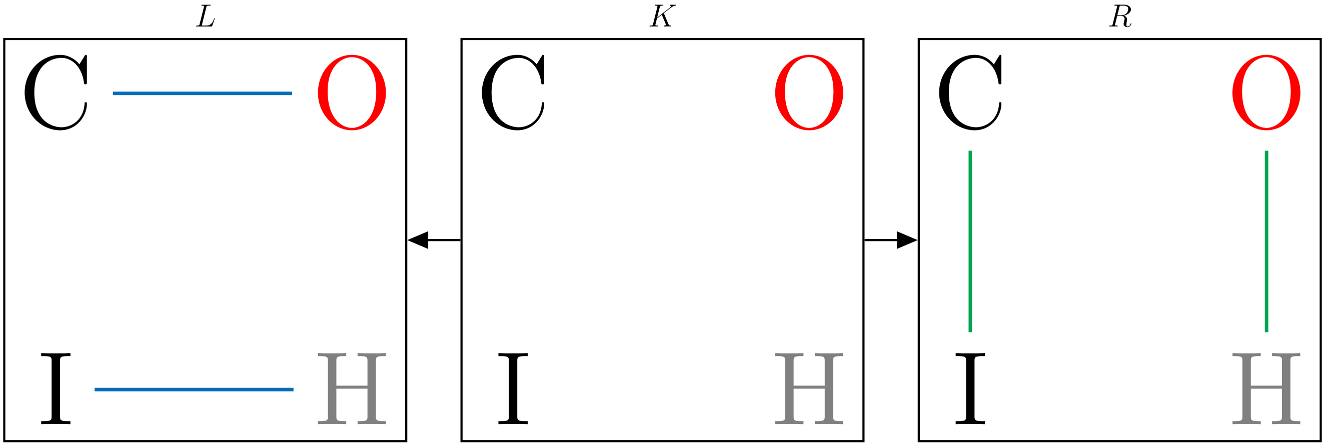

Files: out/053_r_10_11300100_{L, K, R}

0.0.12 11

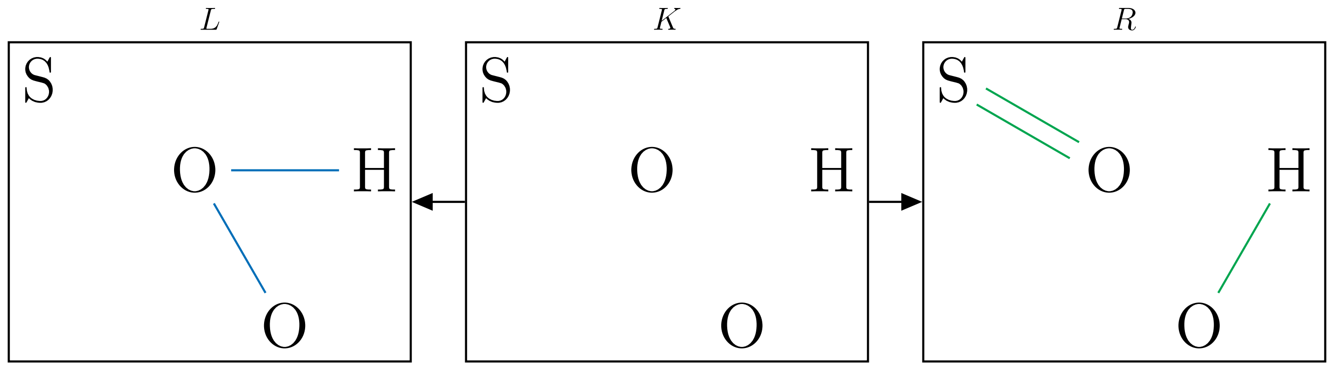

Files: out/056_r_11_10300000_{L, K, R}

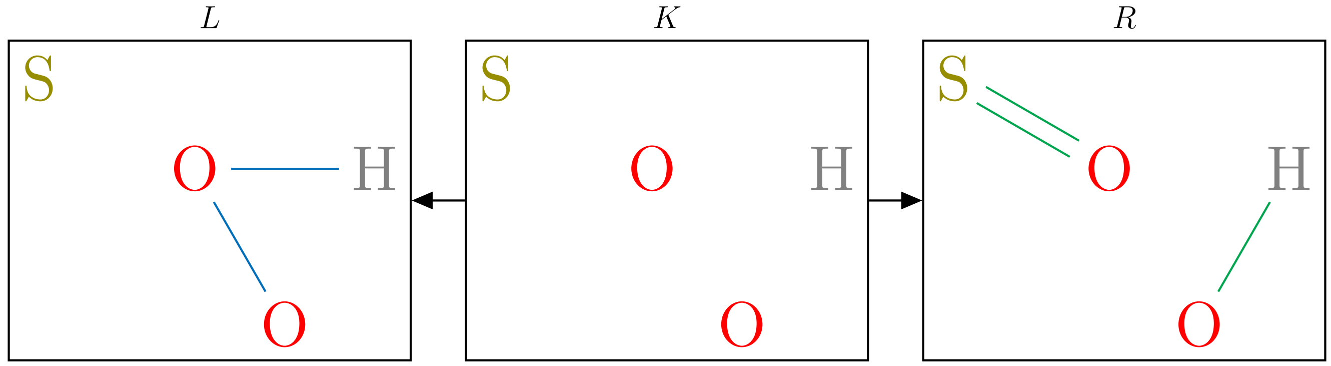

Files: out/058_r_11_11300100_{L, K, R}

0.0.13 12

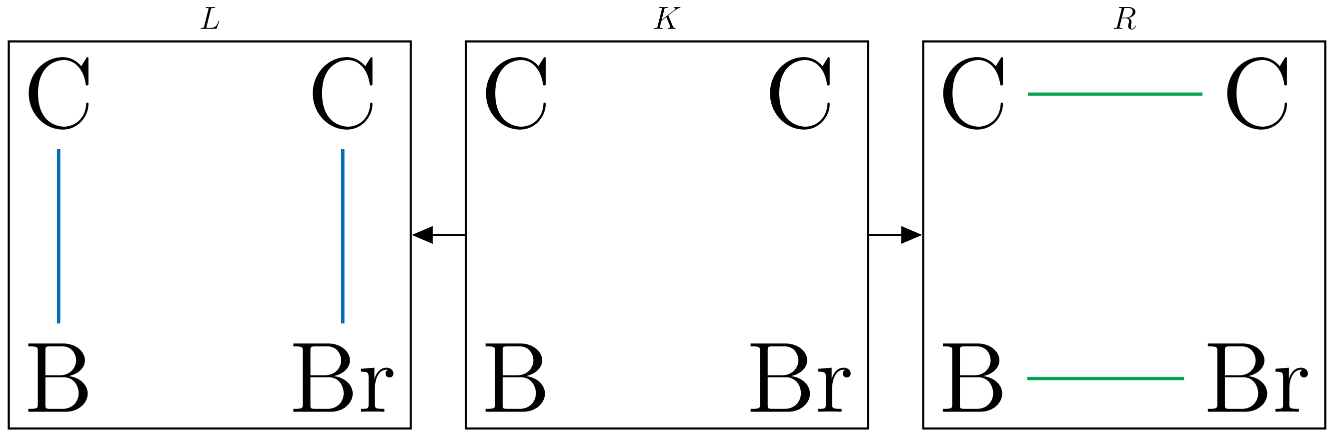

Files: out/061_r_12_10300000_{L, K, R}

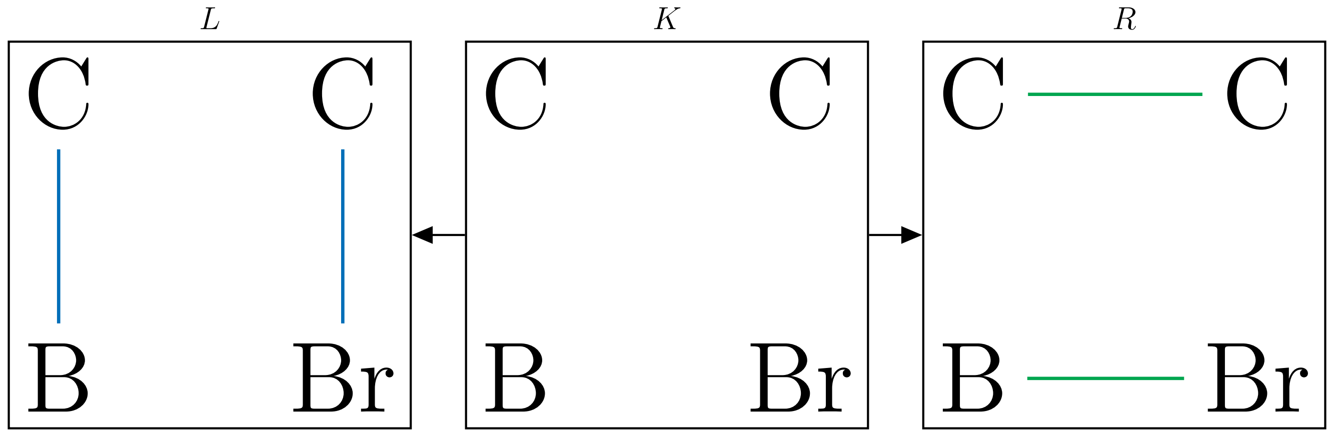

Files: out/063_r_12_11300100_{L, K, R}

0.0.14 13

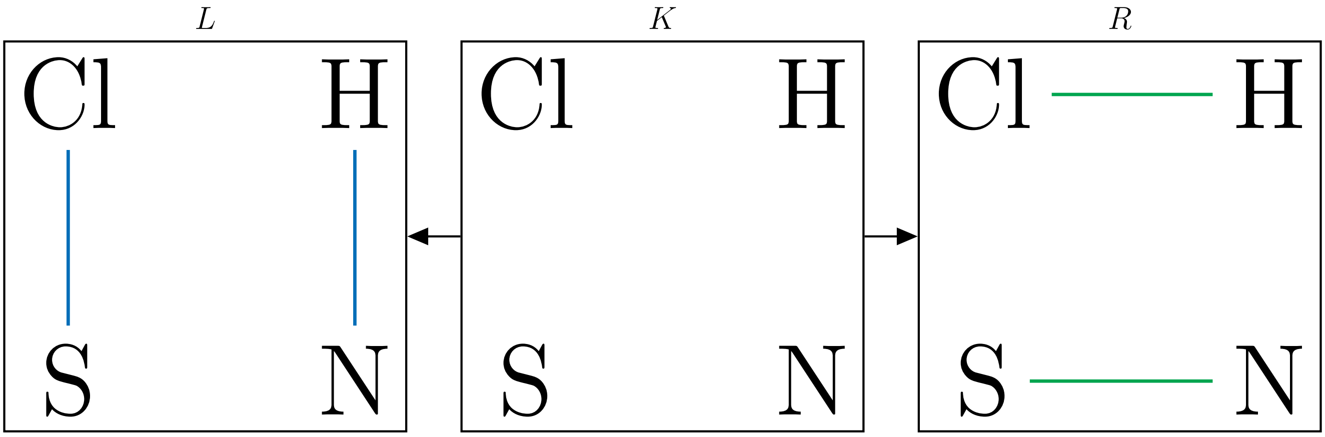

Files: out/066_r_13_10300000_{L, K, R}

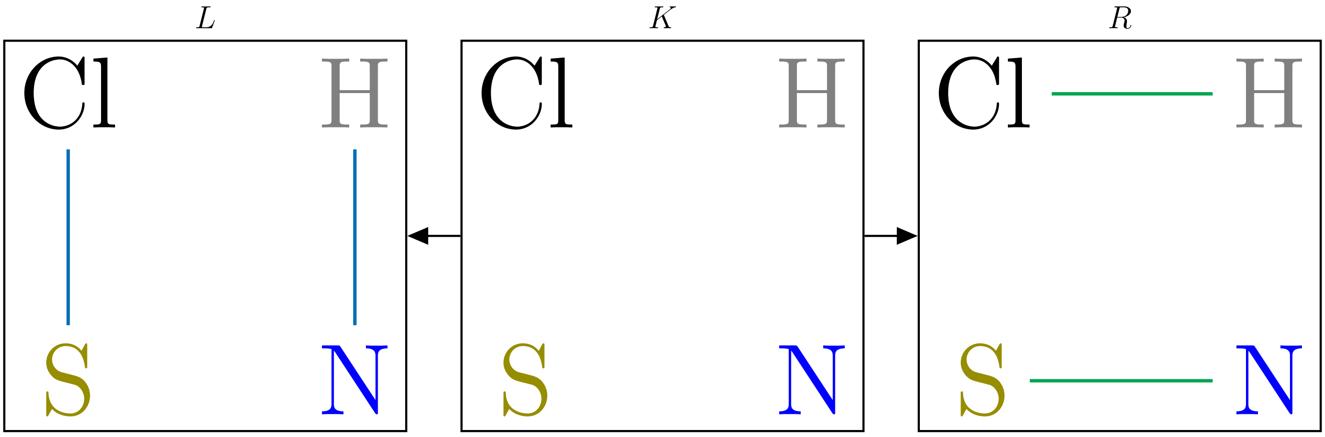

Files: out/068_r_13_11300100_{L, K, R}

0.0.15 14

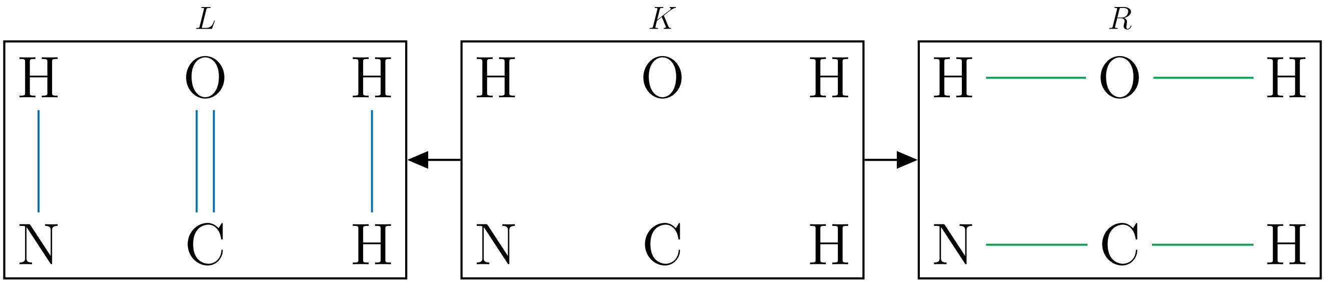

Files: out/071_r_14_10300000_{L, K, R}

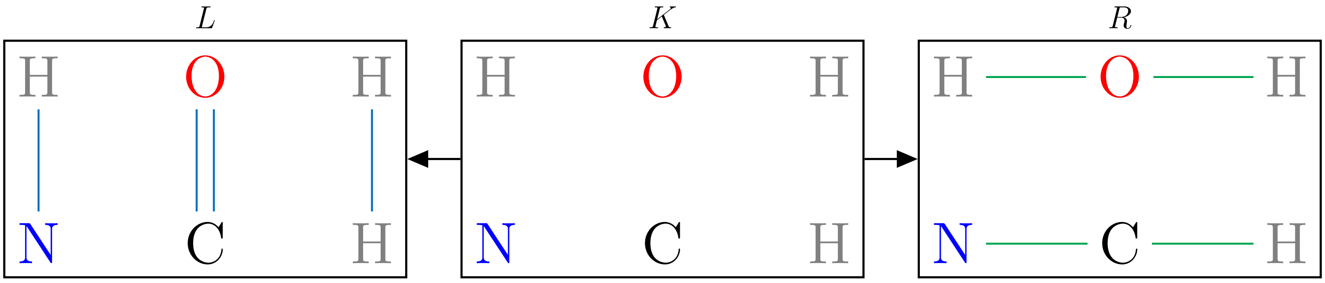

Files: out/073_r_14_11300100_{L, K, R}

0.0.16 15

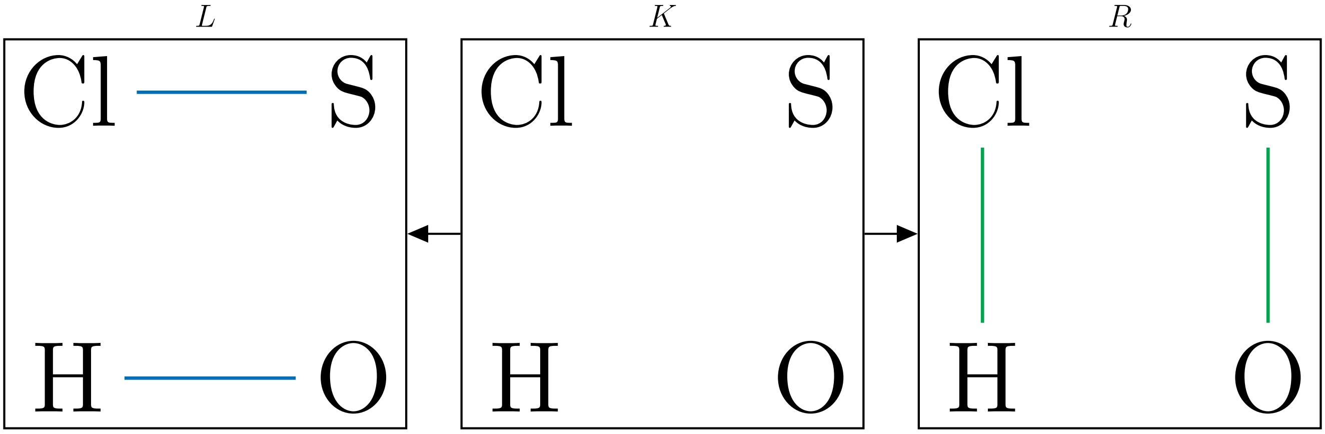

Files: out/076_r_15_10300000_{L, K, R}

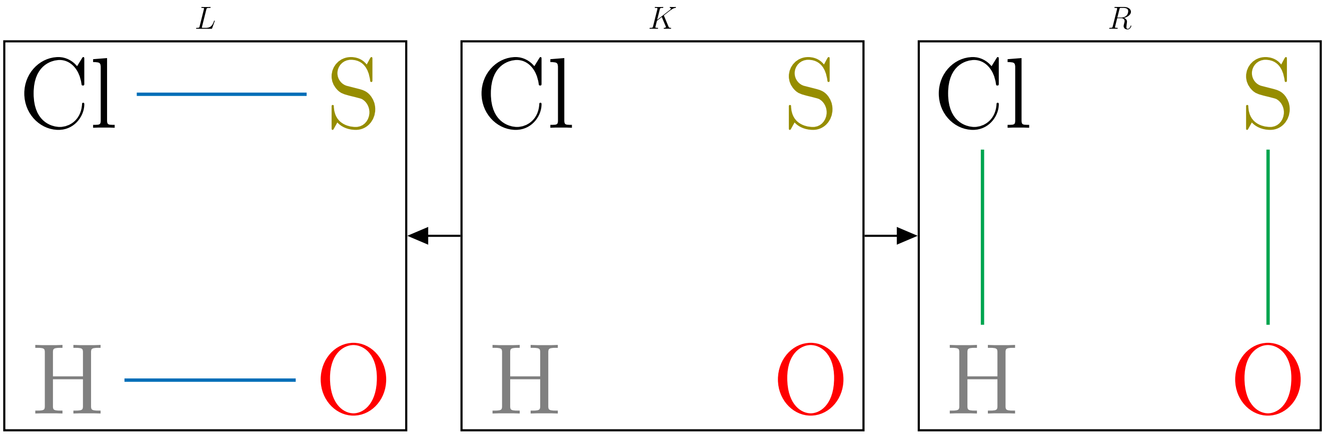

Files: out/078_r_15_11300100_{L, K, R}

0.0.17 16

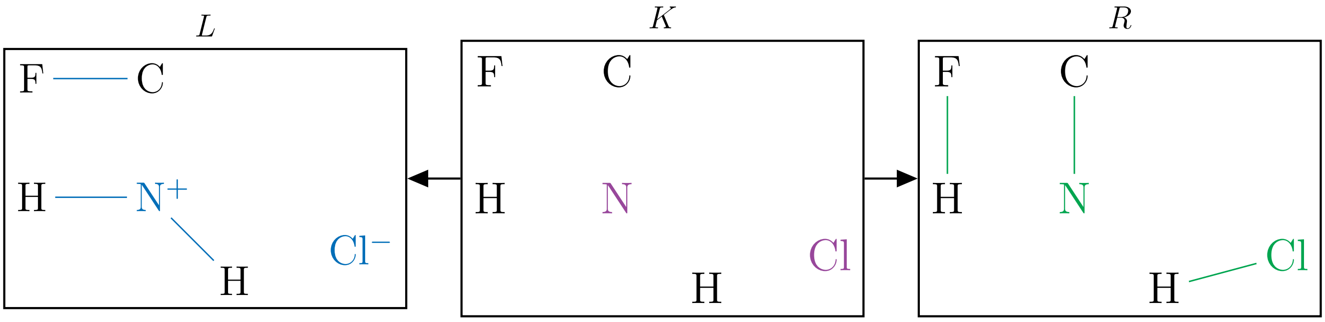

Files: out/081_r_16_10300000_{L, K, R}

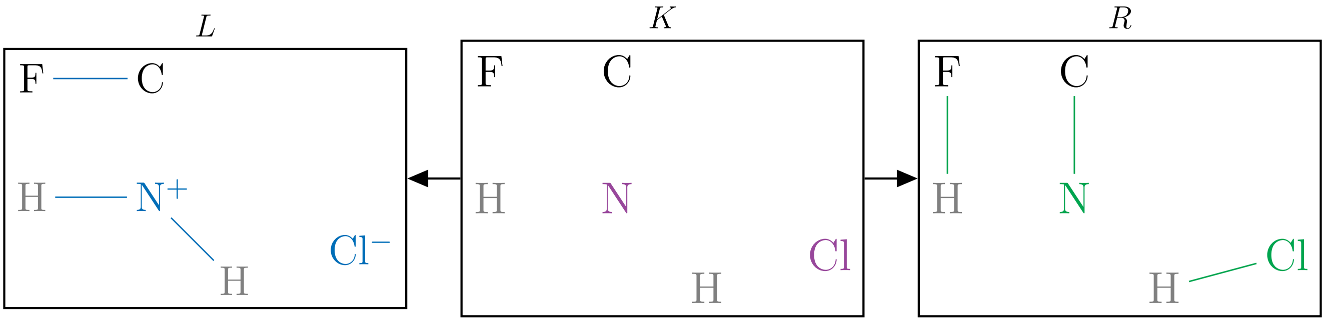

Files: out/083_r_16_11300100_{L, K, R}

0.0.18 17

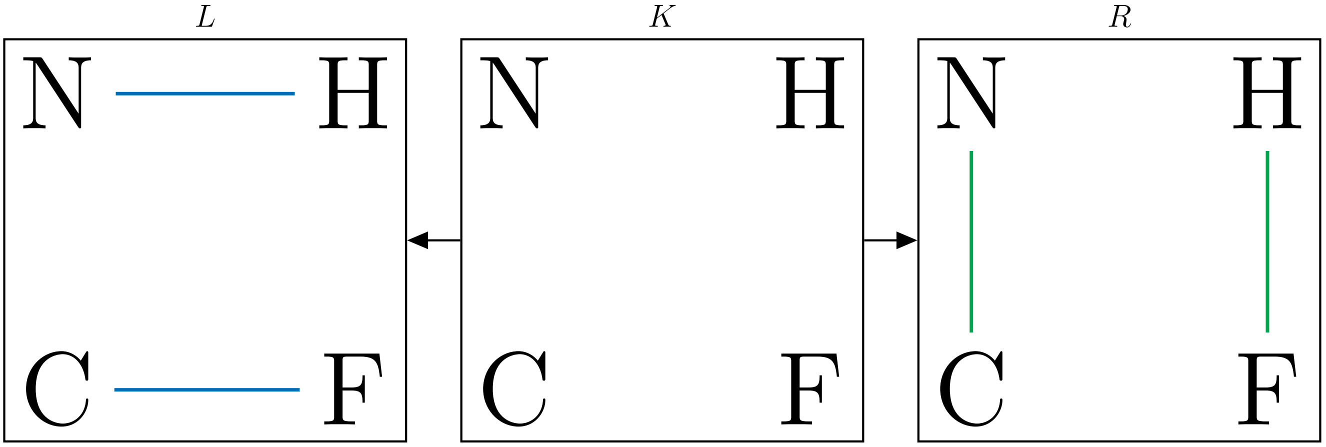

Files: out/086_r_17_10300000_{L, K, R}

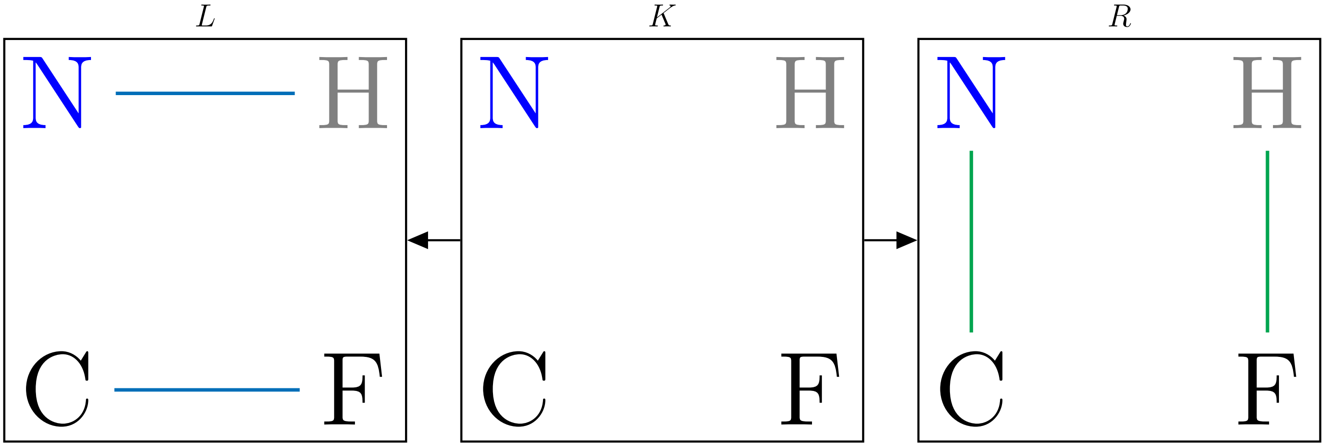

Files: out/088_r_17_11300100_{L, K, R}

0.0.19 18

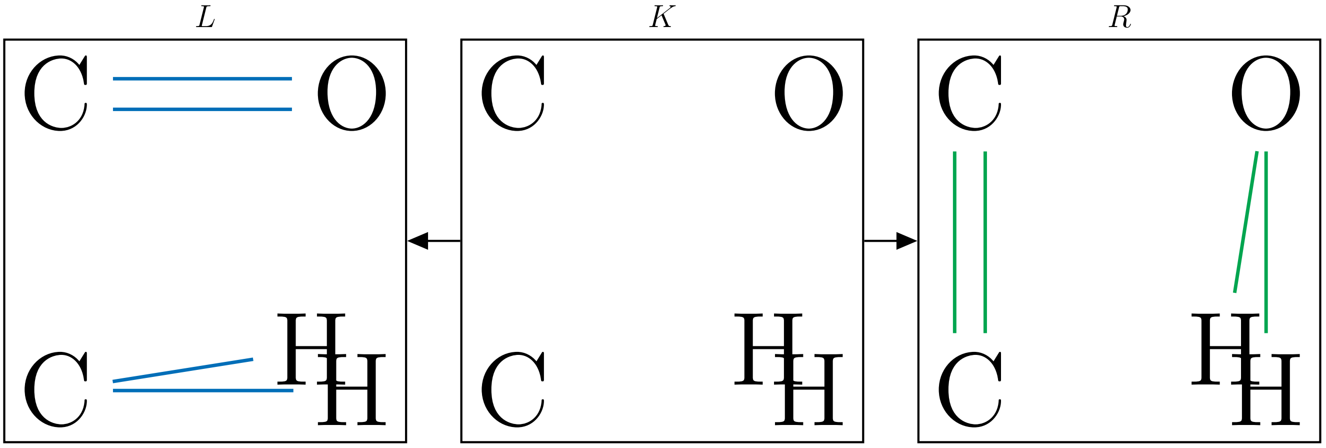

Files: out/091_r_18_10300000_{L, K, R}

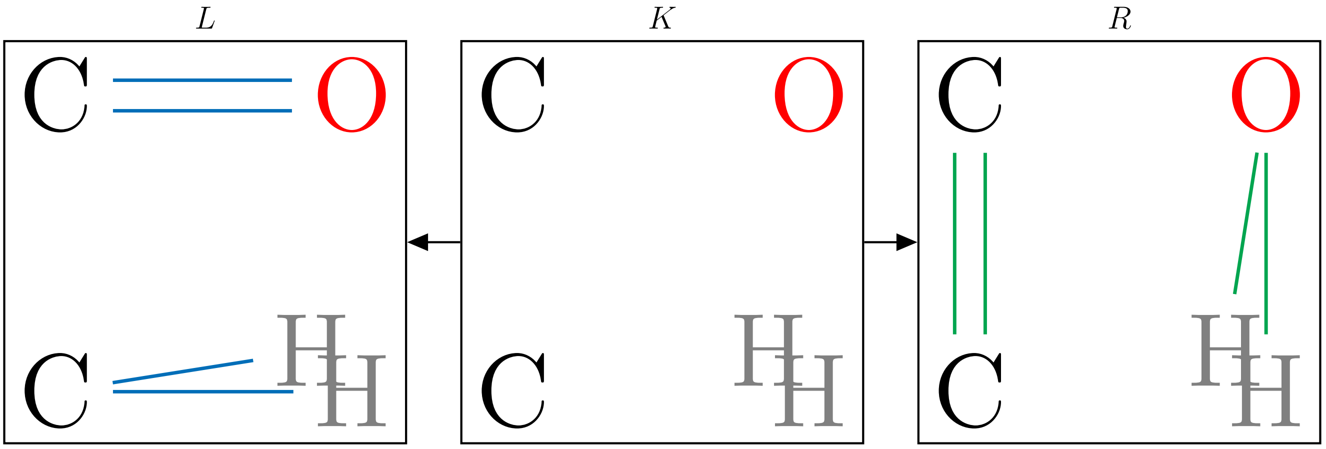

Files: out/093_r_18_11300100_{L, K, R}

0.0.20 19

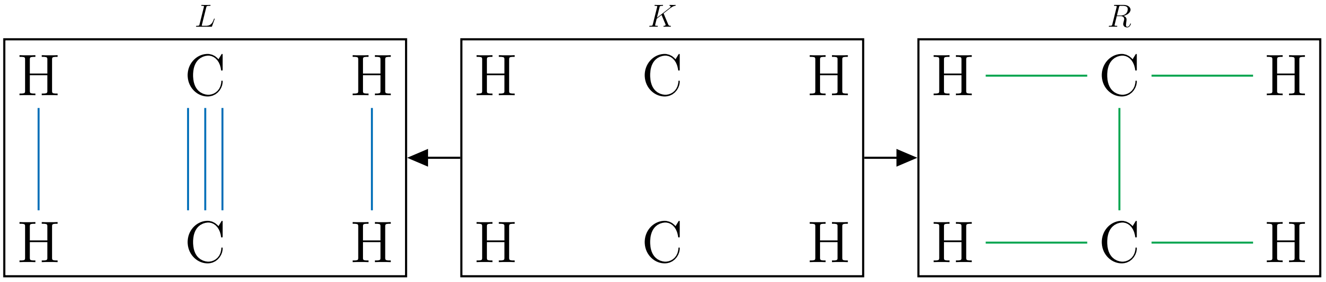

Files: out/096_r_19_10300000_{L, K, R}

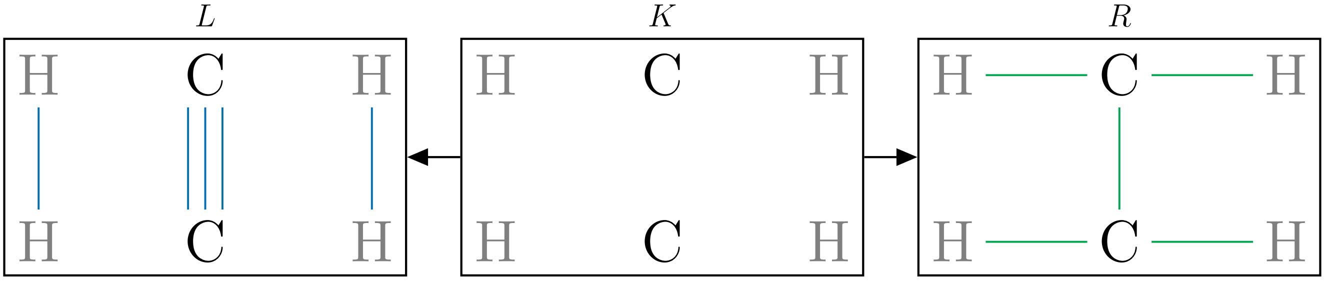

Files: out/098_r_19_11300100_{L, K, R}

0.0.21 20

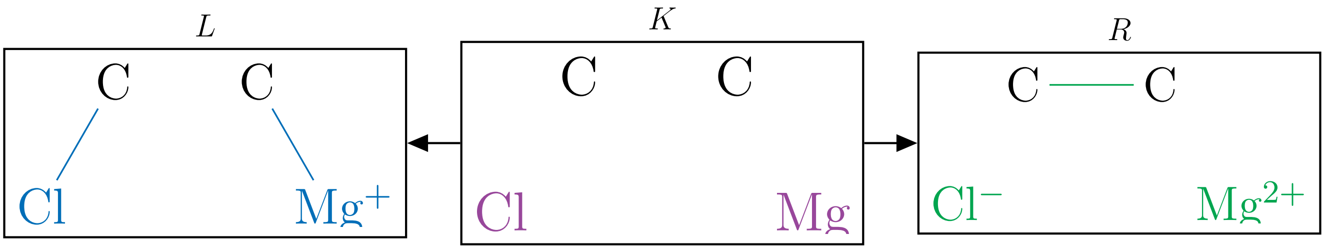

Files: out/101_r_20_10300000_{L, K, R}

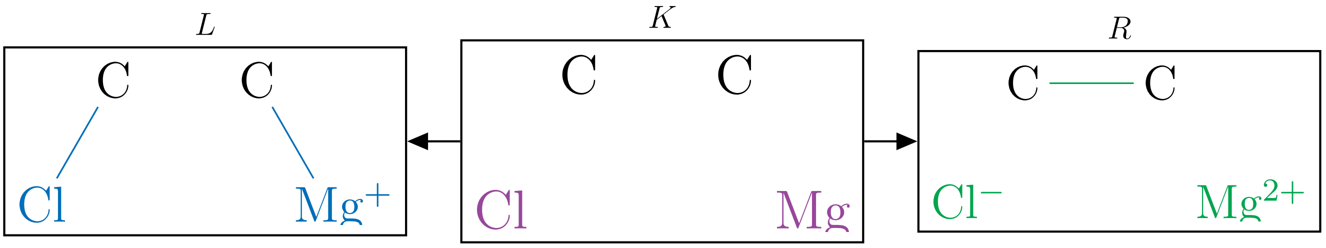

Files: out/103_r_20_11300100_{L, K, R}

0.0.22 21

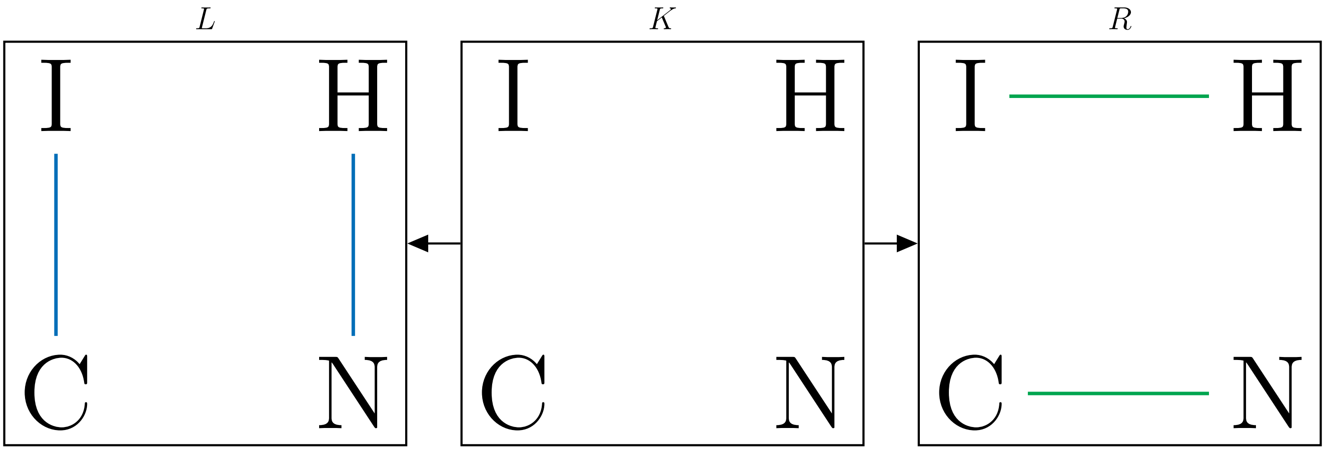

Files: out/106_r_21_10300000_{L, K, R}

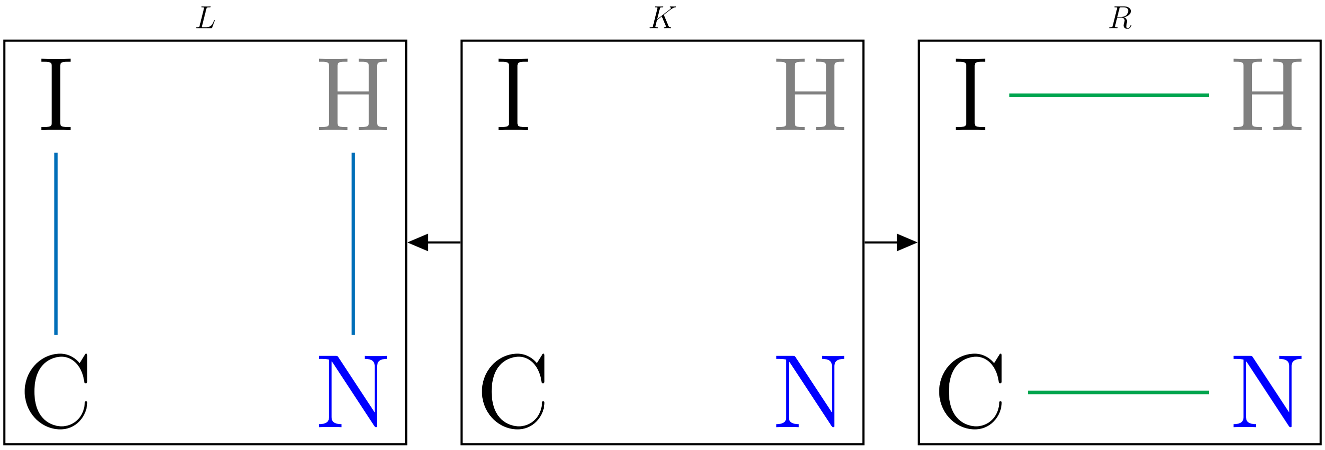

Files: out/108_r_21_11300100_{L, K, R}

0.0.23 22

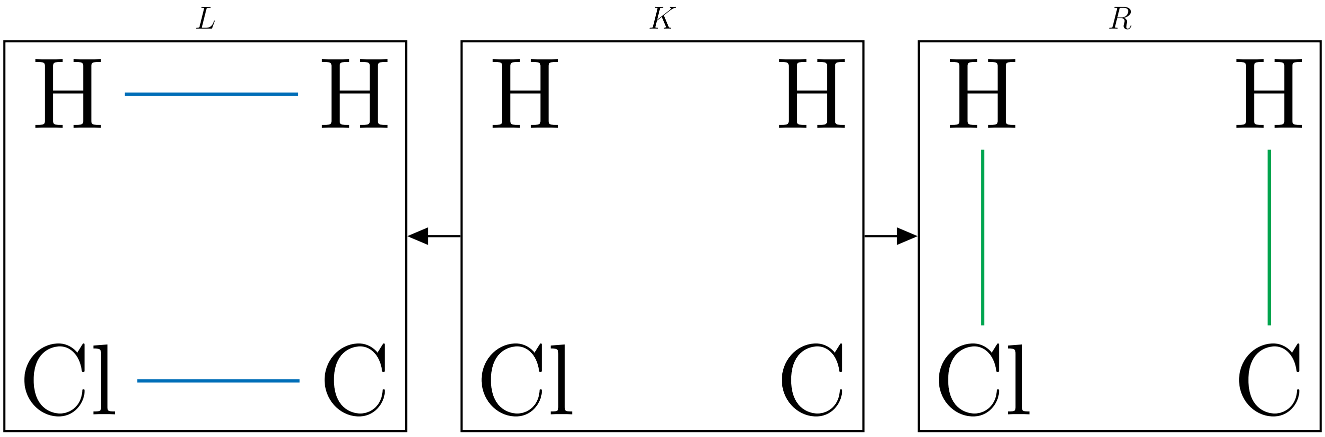

Files: out/111_r_22_10300000_{L, K, R}

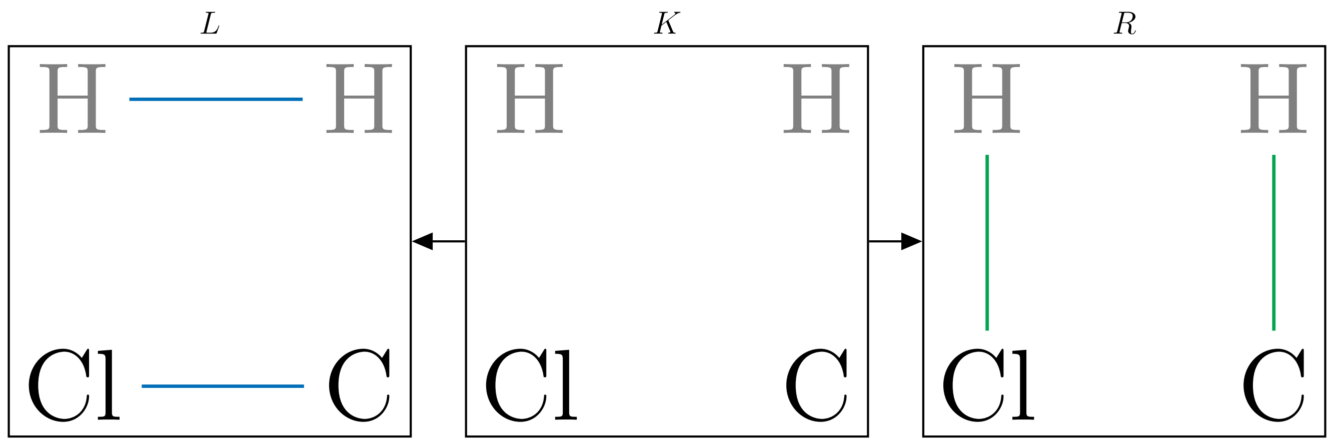

Files: out/113_r_22_11300100_{L, K, R}

0.0.24 23

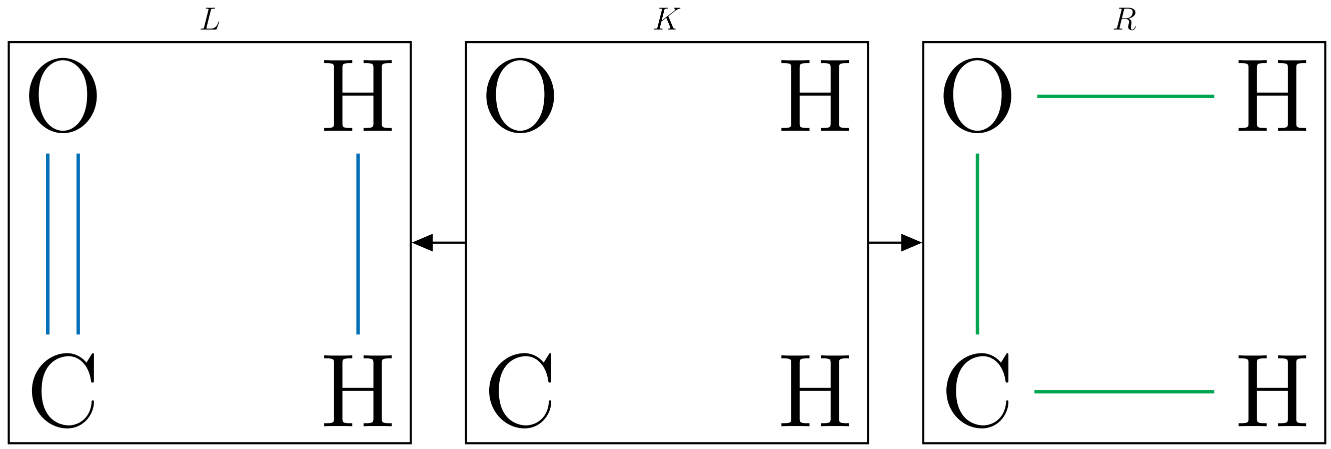

Files: out/116_r_23_10300000_{L, K, R}

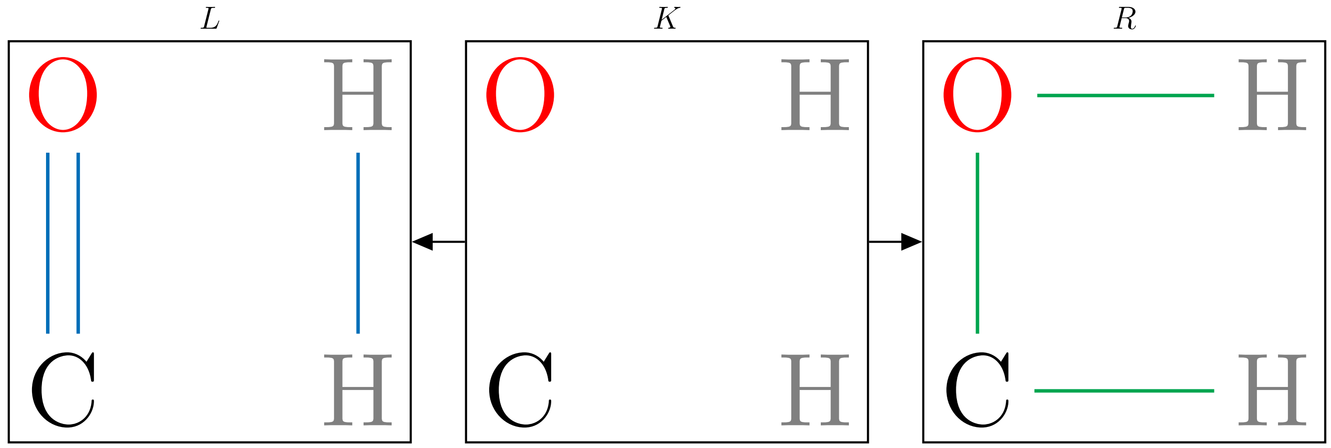

Files: out/118_r_23_11300100_{L, K, R}

0.0.25 24

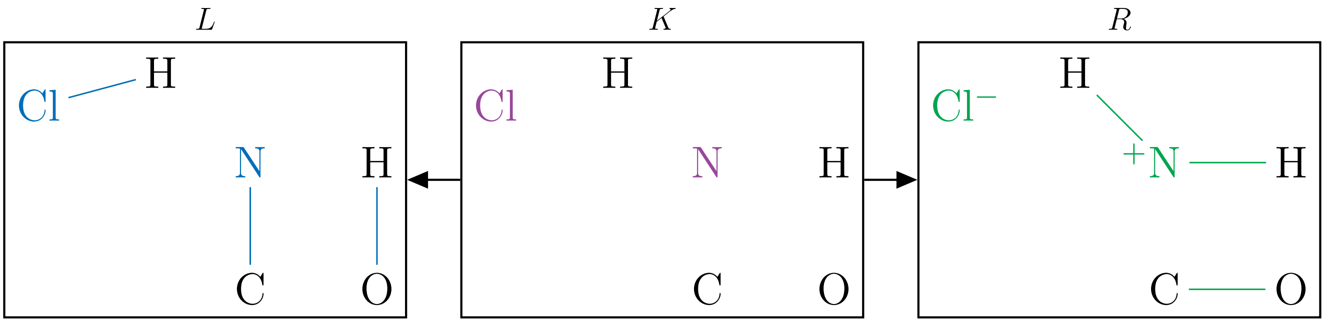

Files: out/121_r_24_10300000_{L, K, R}

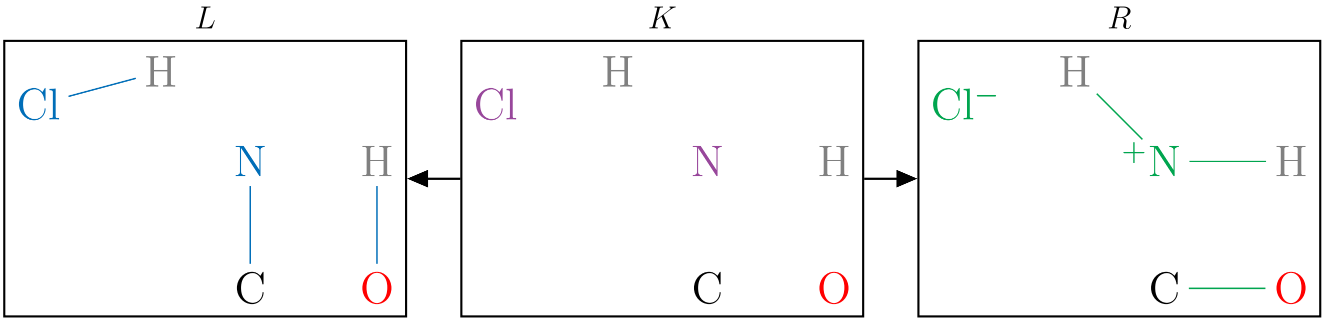

Files: out/123_r_24_11300100_{L, K, R}

0.0.26 25

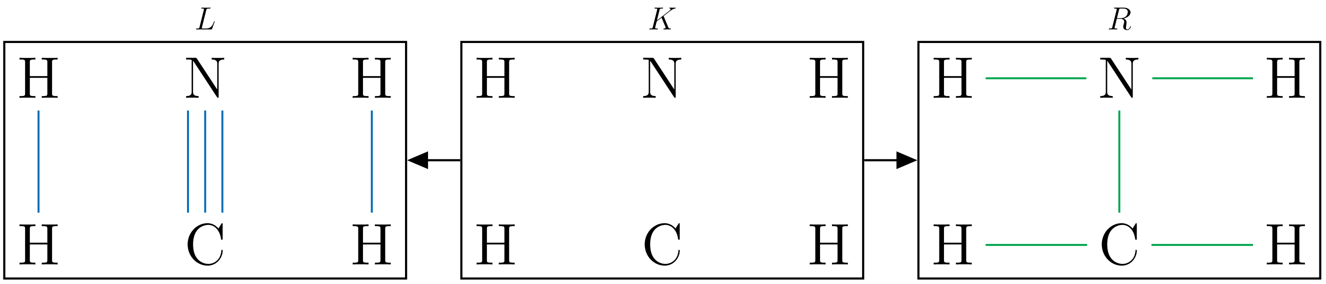

Files: out/126_r_25_10300000_{L, K, R}

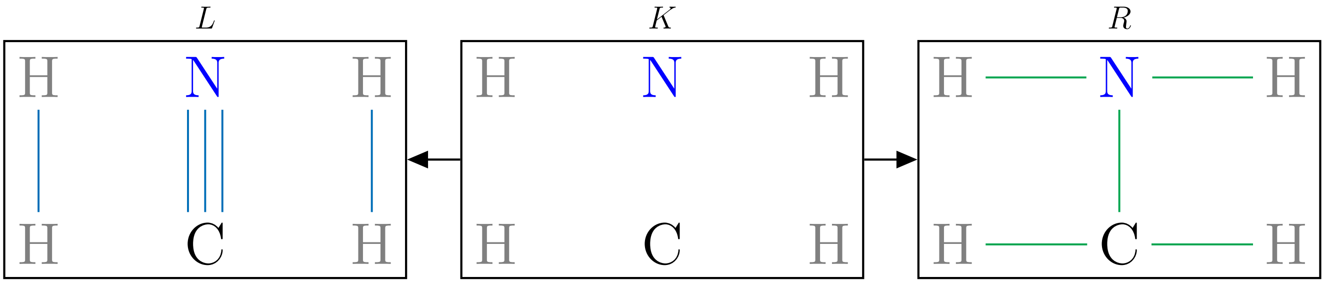

Files: out/128_r_25_11300100_{L, K, R}

0.0.27 26

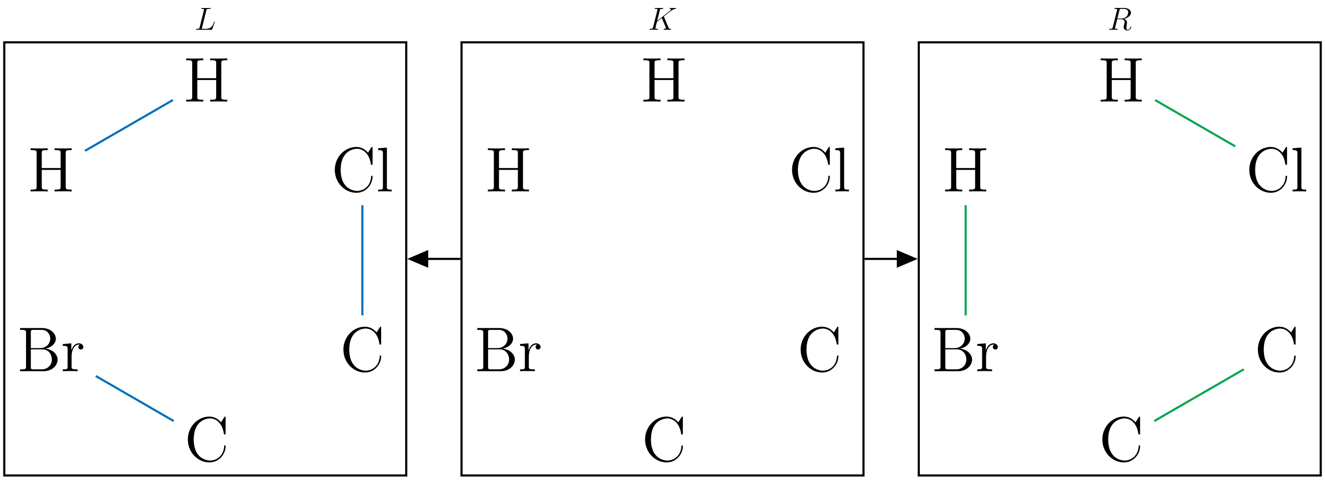

Files: out/131_r_26_10300000_{L, K, R}

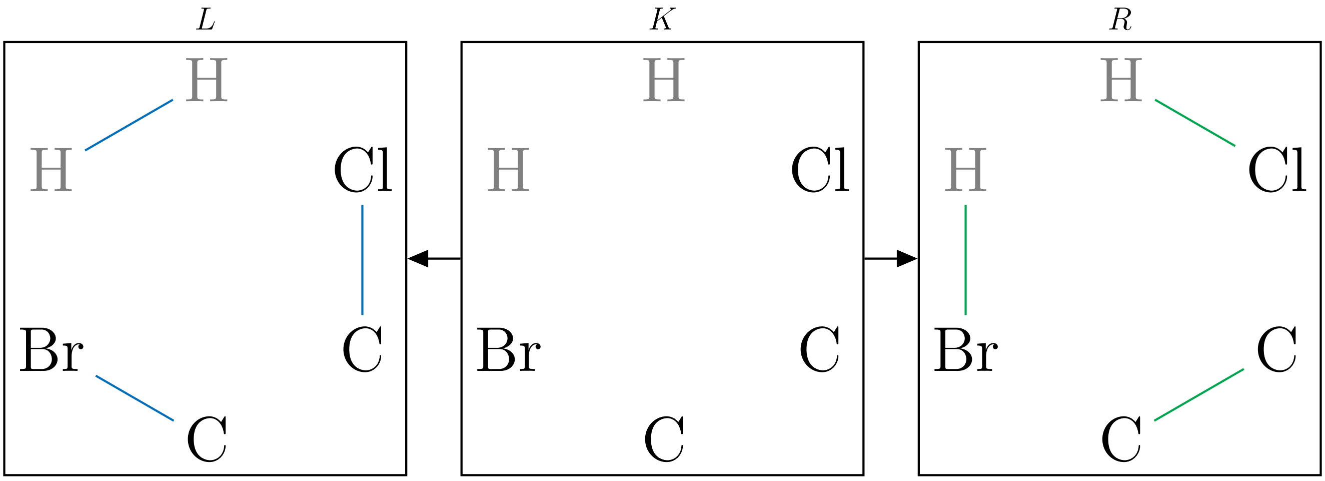

Files: out/133_r_26_11300100_{L, K, R}

0.0.28 27

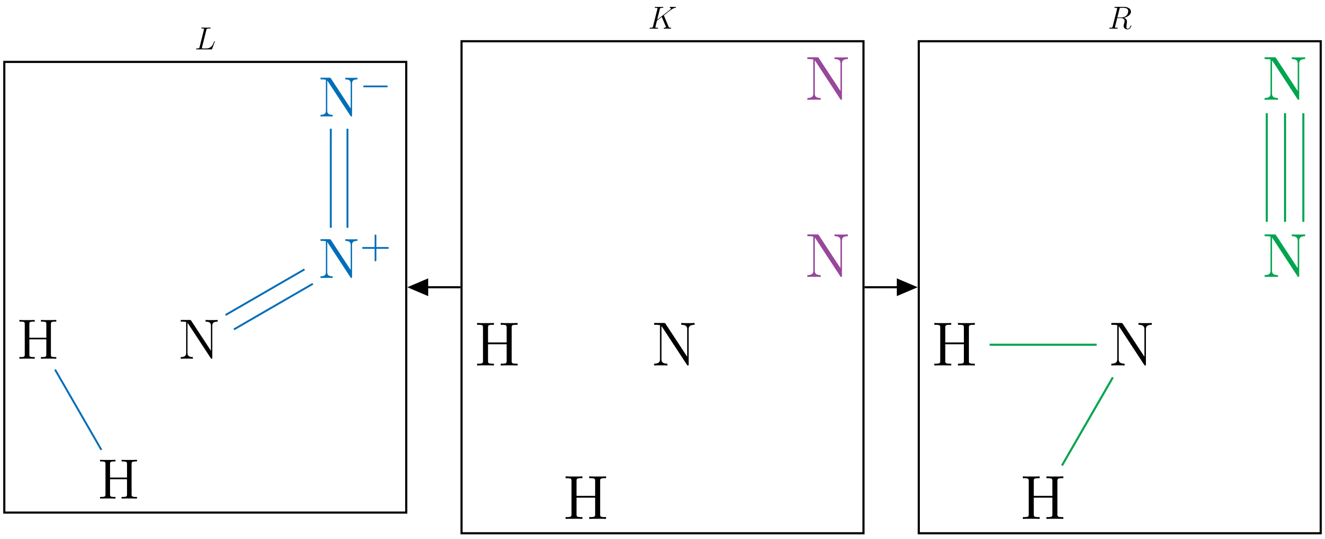

Files: out/136_r_27_10300000_{L, K, R}

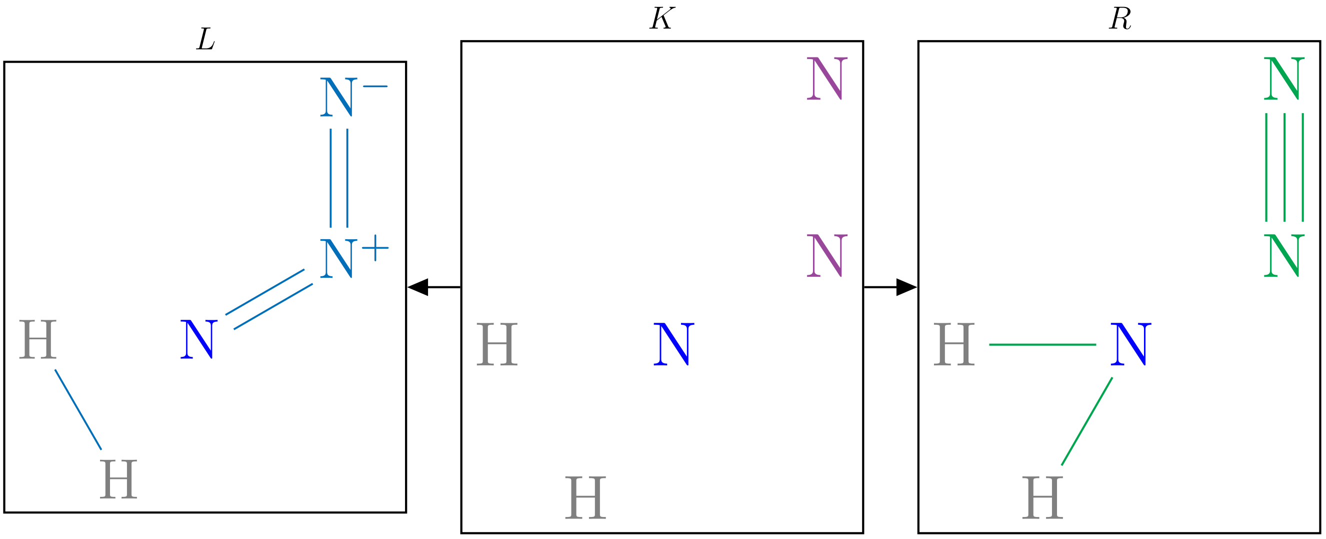

Files: out/138_r_27_11300100_{L, K, R}

0.0.29 28

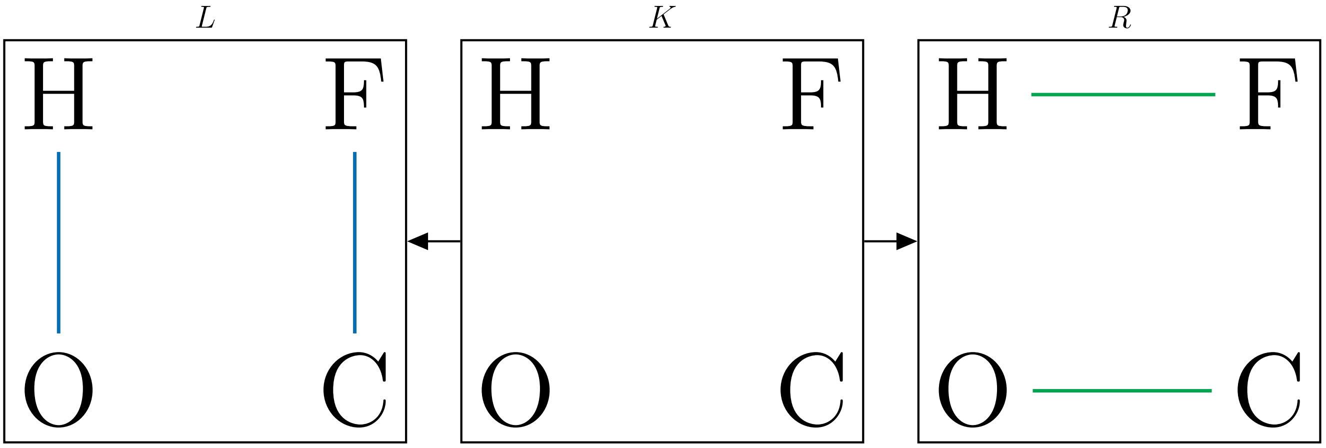

Files: out/141_r_28_10300000_{L, K, R}

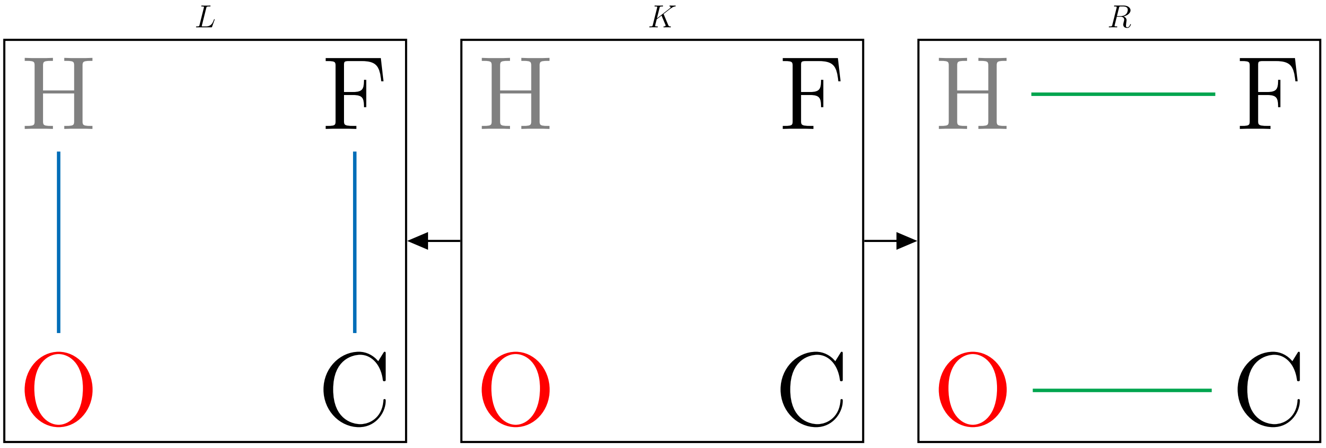

Files: out/143_r_28_11300100_{L, K, R}

0.0.30 29

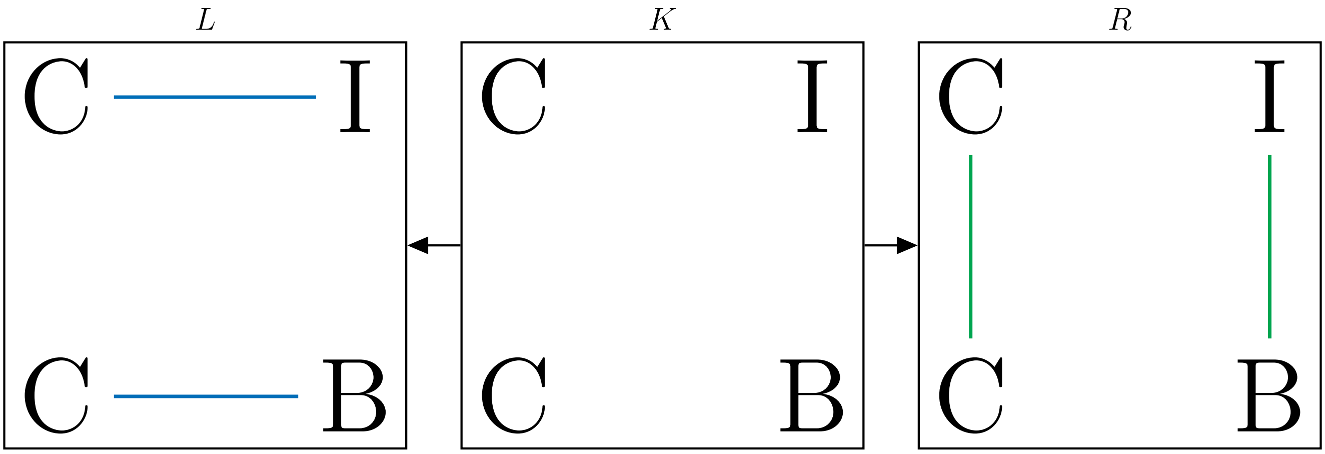

Files: out/146_r_29_10300000_{L, K, R}

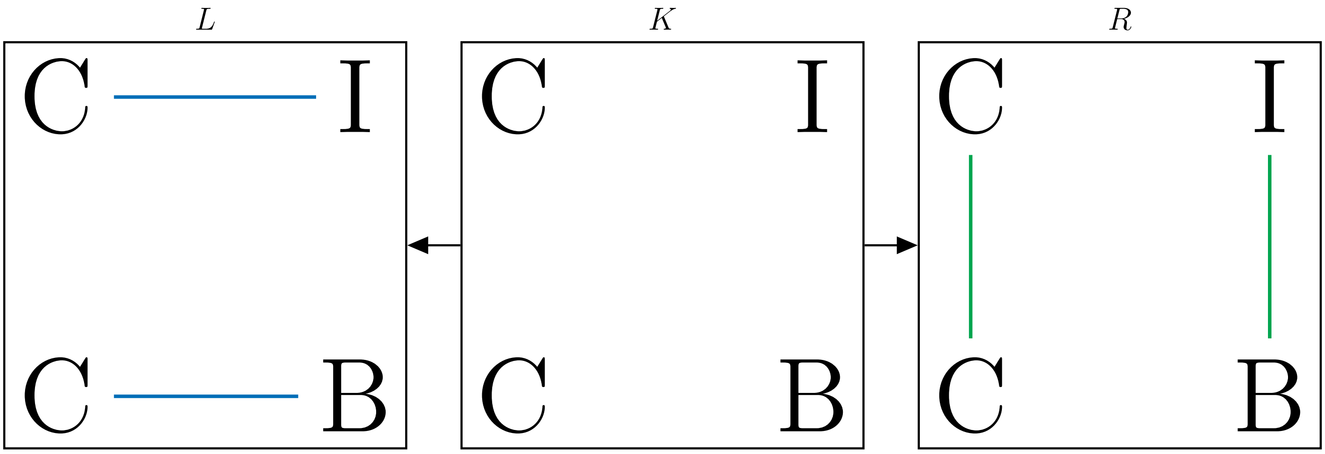

Files: out/148_r_29_11300100_{L, K, R}

0.0.31 30

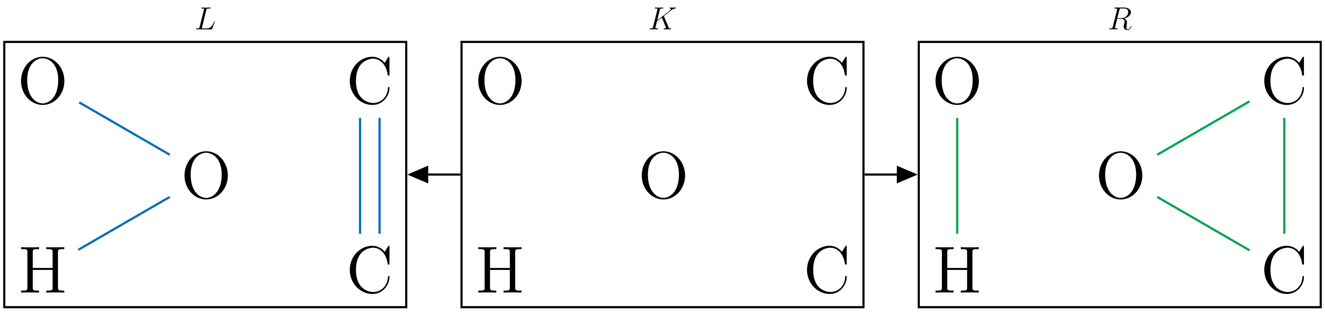

Files: out/151_r_30_10300000_{L, K, R}

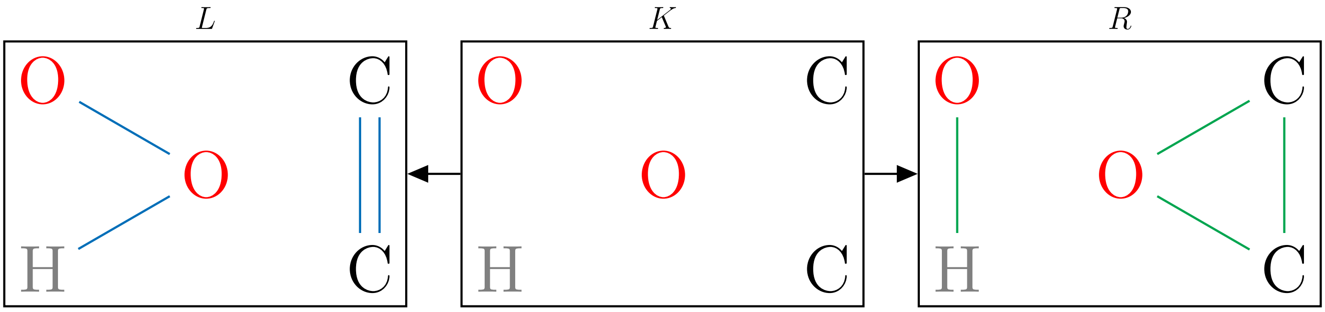

Files: out/153_r_30_11300100_{L, K, R}

0.0.32 31

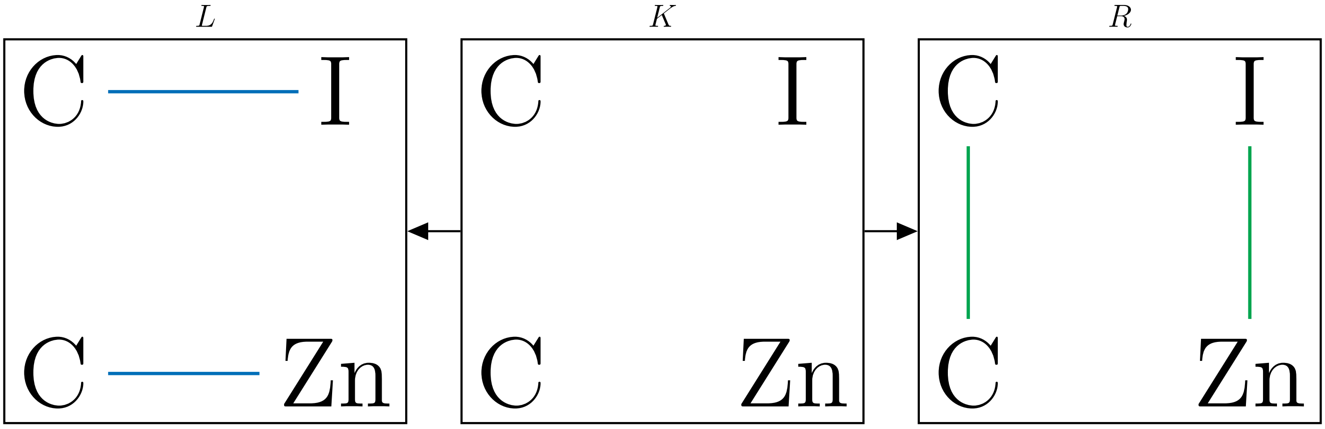

Files: out/156_r_31_10300000_{L, K, R}

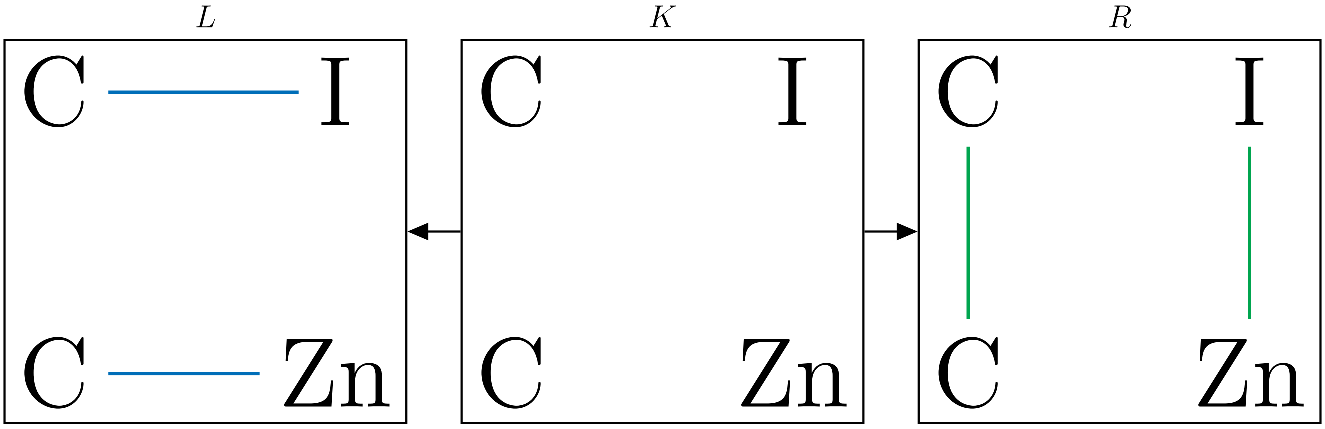

Files: out/158_r_31_11300100_{L, K, R}

0.0.33 32

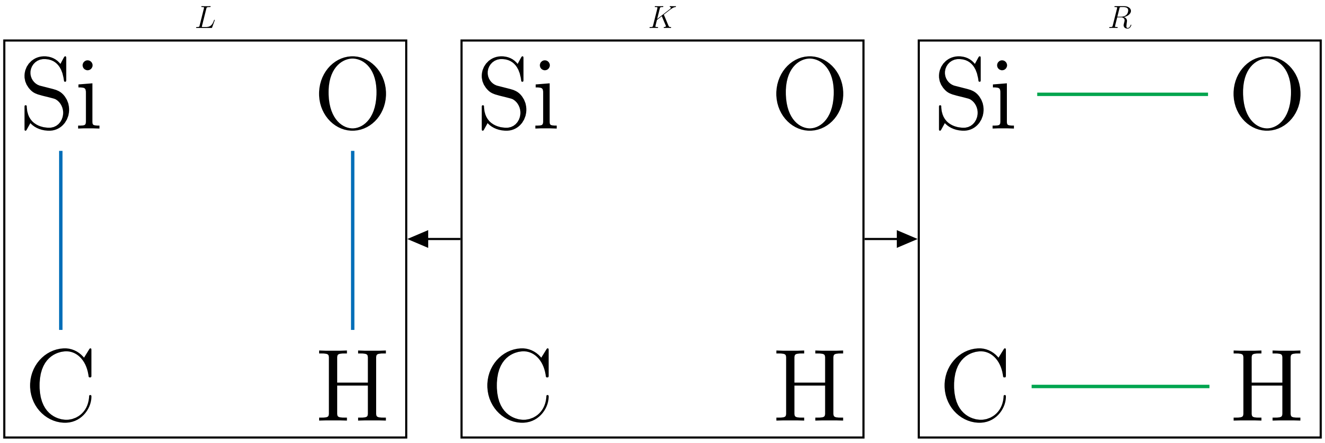

Files: out/161_r_32_10300000_{L, K, R}

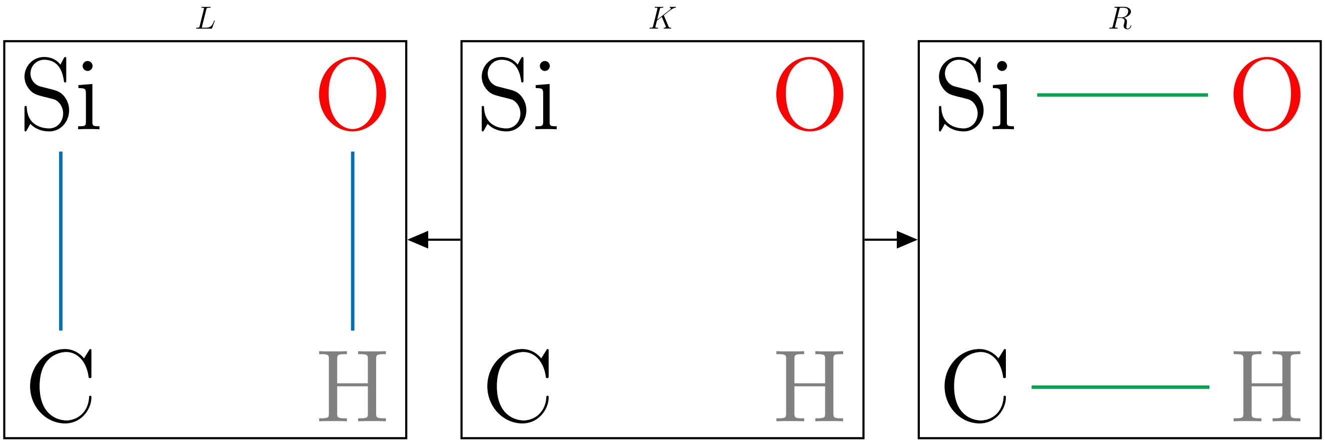

Files: out/163_r_32_11300100_{L, K, R}

0.0.34 33

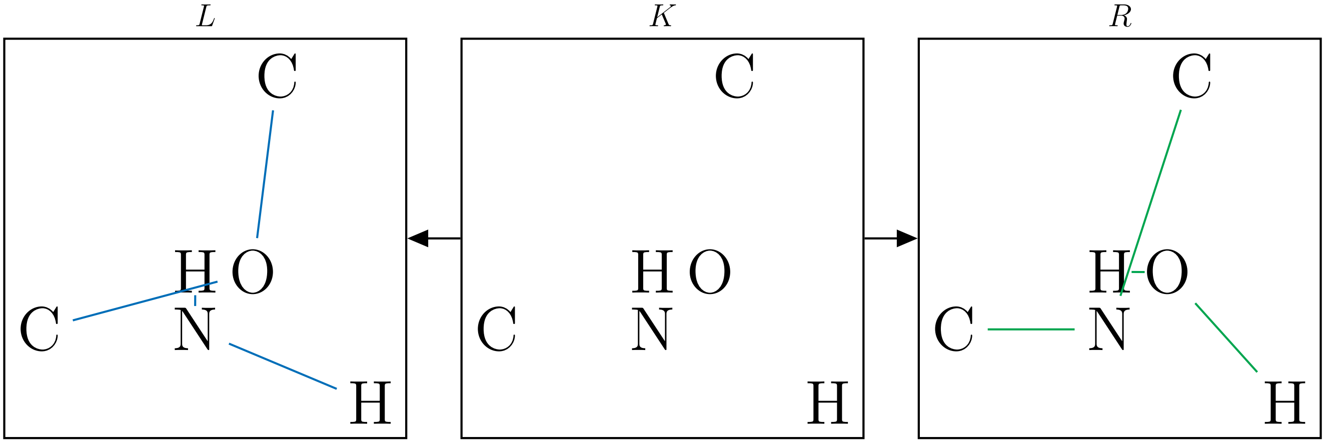

Files: out/166_r_33_10300000_{L, K, R}

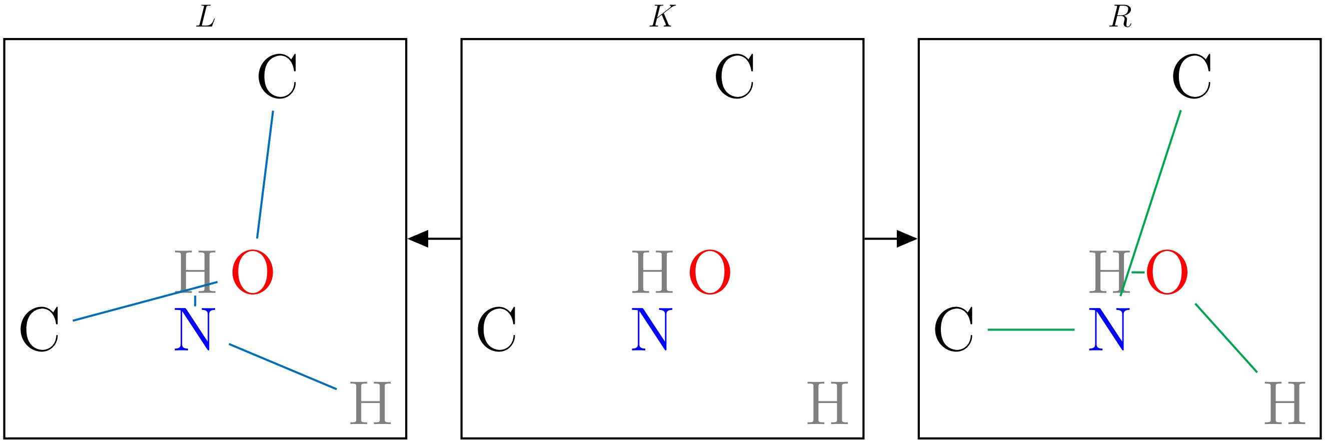

Files: out/168_r_33_11300100_{L, K, R}

0.0.35 34

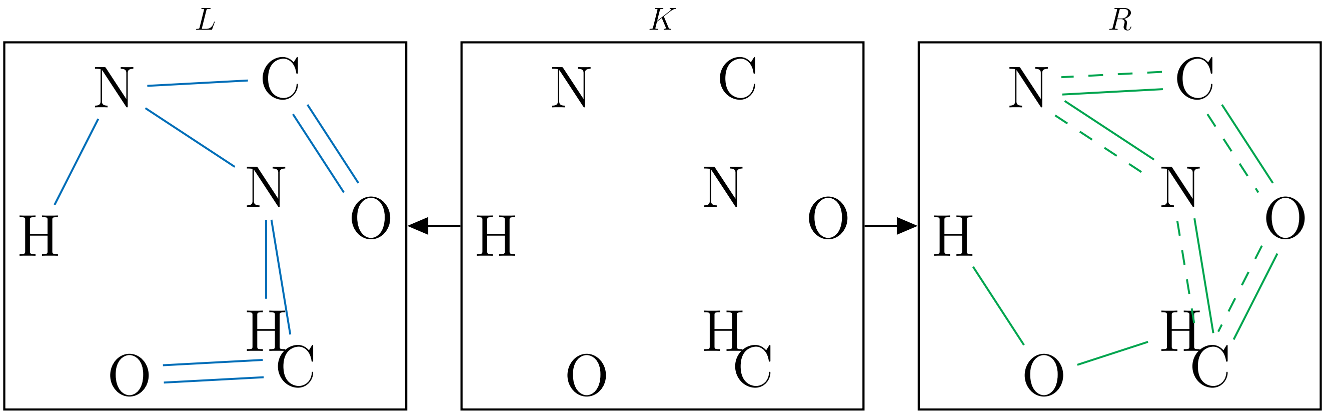

Files: out/171_r_34_10300000_{L, K, R}

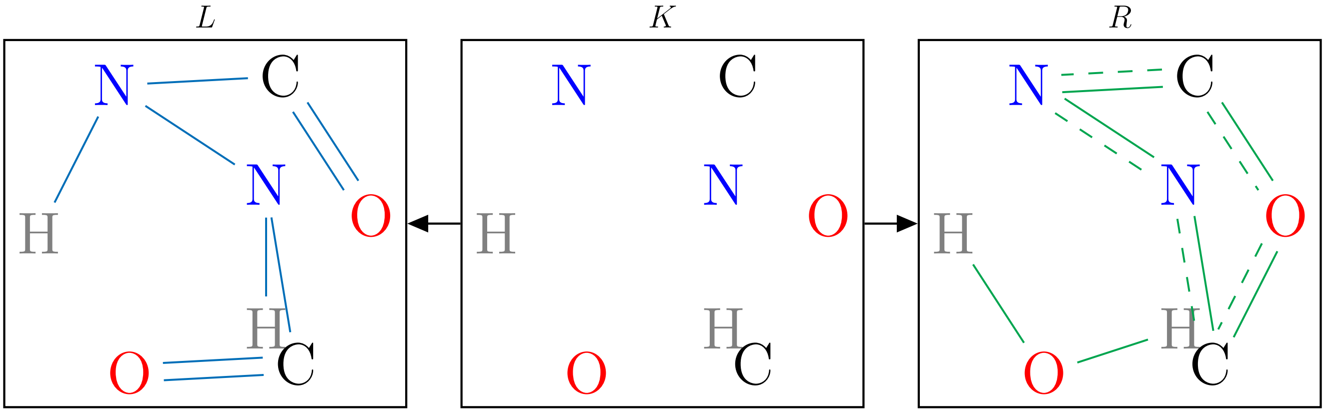

Files: out/173_r_34_11300100_{L, K, R}

0.0.36 35

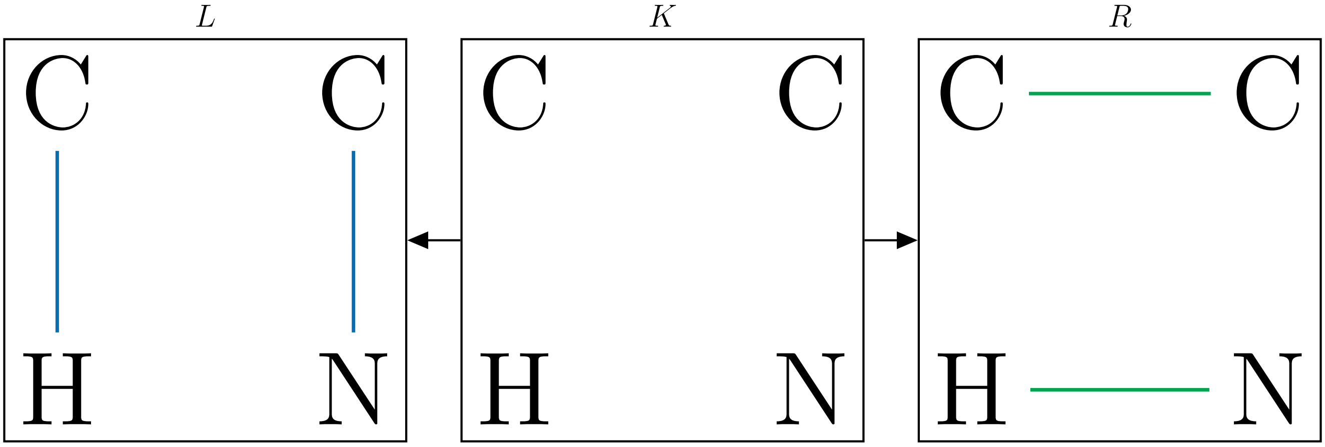

Files: out/176_r_35_10300000_{L, K, R}

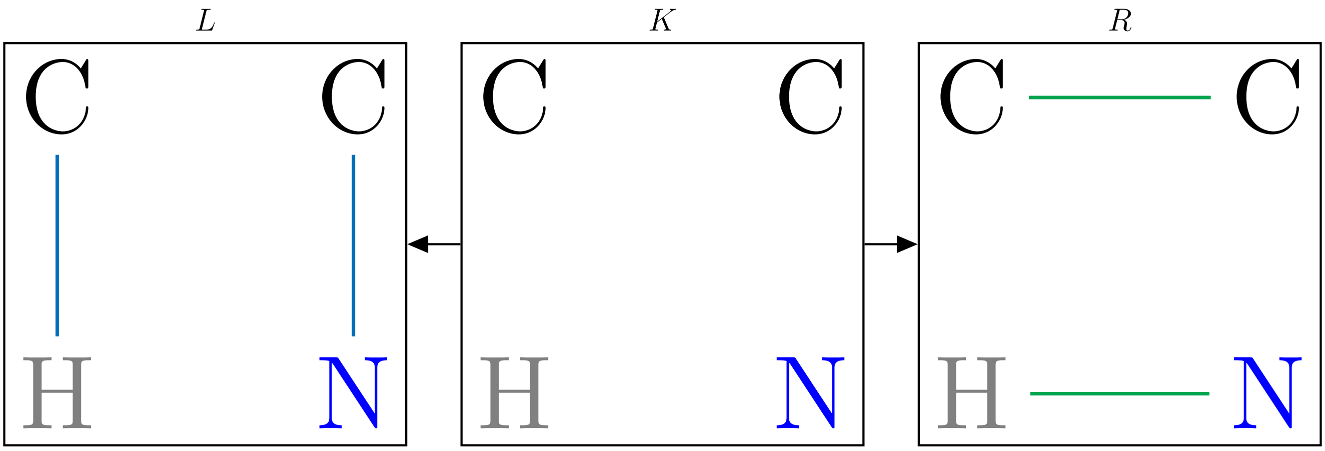

Files: out/178_r_35_11300100_{L, K, R}

0.0.37 36

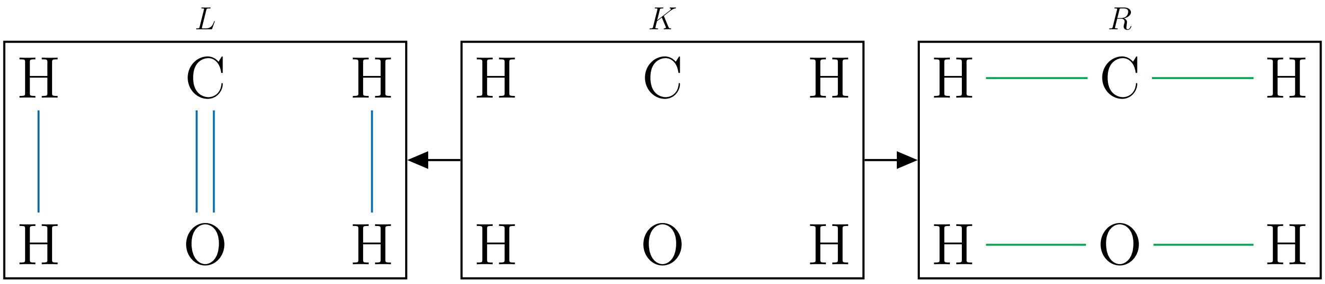

Files: out/181_r_36_10300000_{L, K, R}

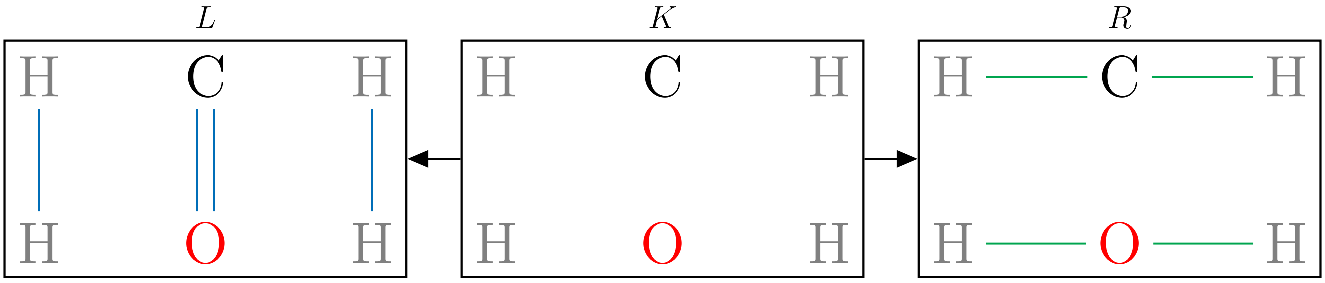

Files: out/183_r_36_11300100_{L, K, R}

0.0.38 37

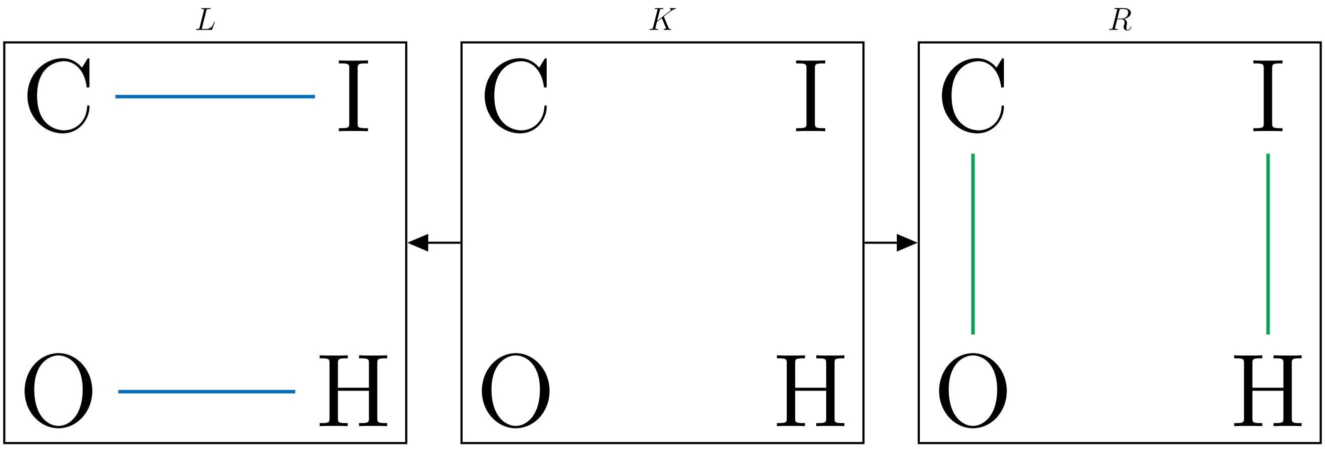

Files: out/186_r_37_10300000_{L, K, R}

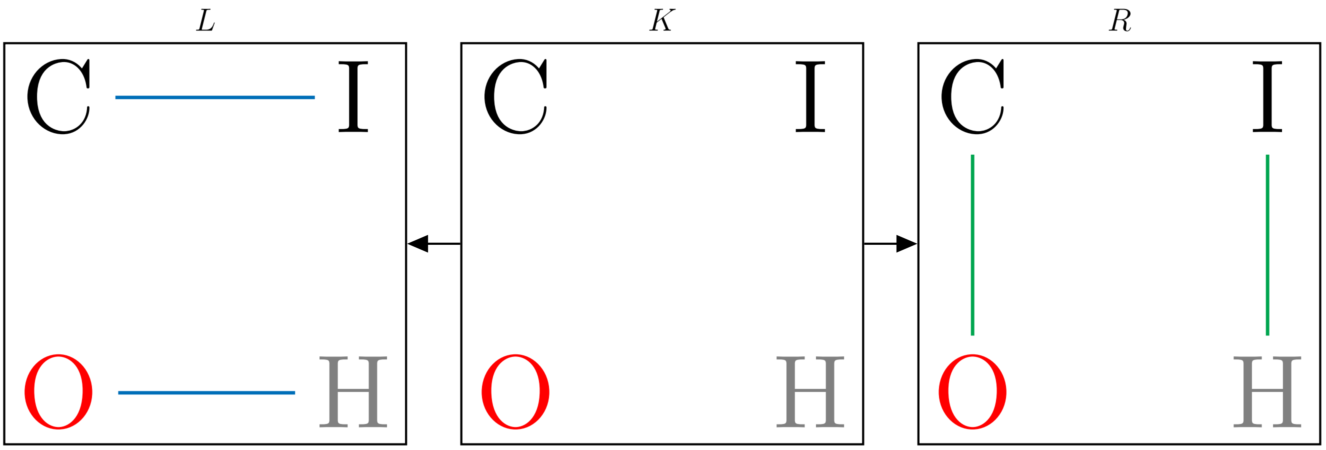

Files: out/188_r_37_11300100_{L, K, R}

0.0.39 38

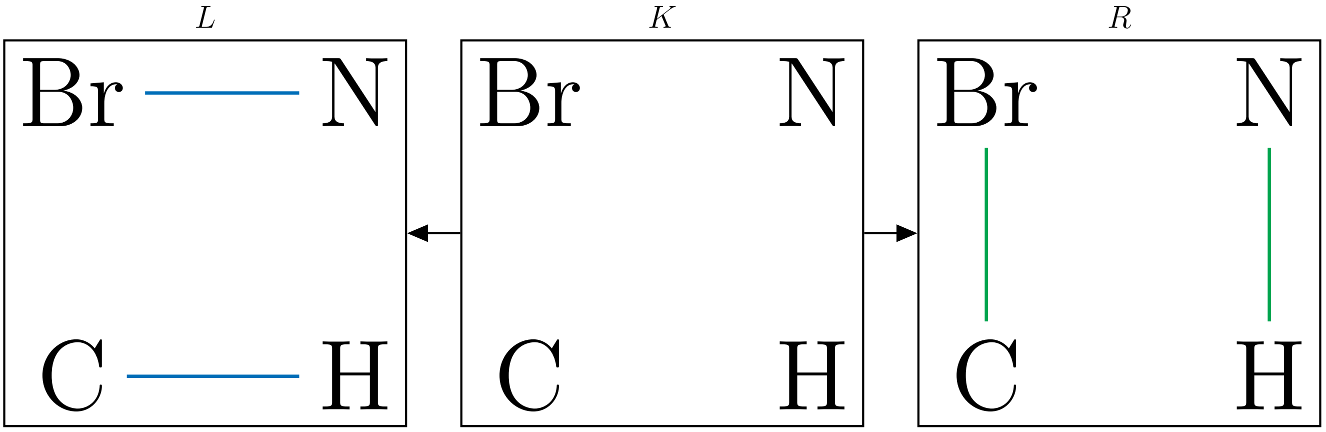

Files: out/191_r_38_10300000_{L, K, R}

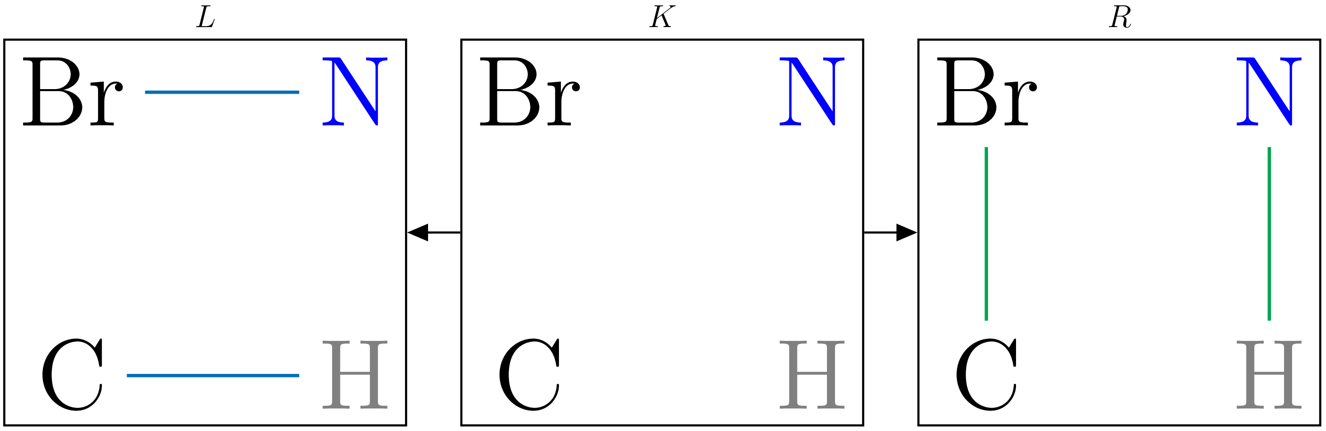

Files: out/193_r_38_11300100_{L, K, R}

0.0.40 39

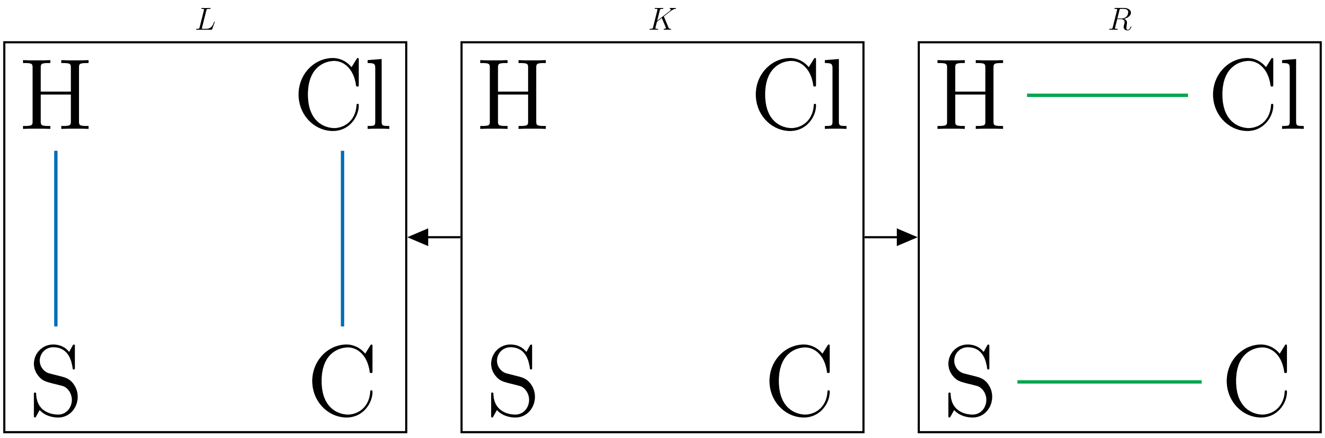

Files: out/196_r_39_10300000_{L, K, R}

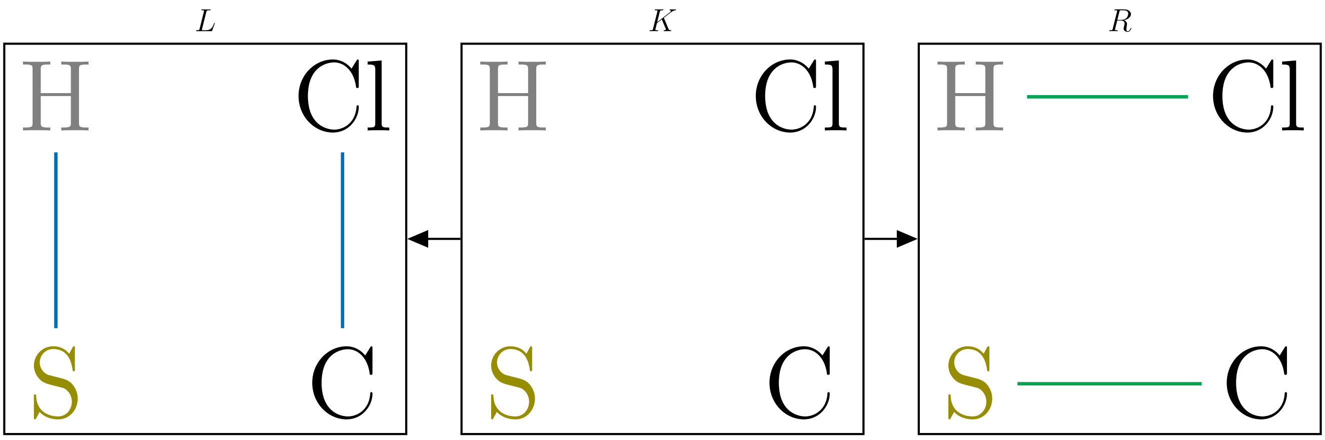

Files: out/198_r_39_11300100_{L, K, R}

0.0.41 40

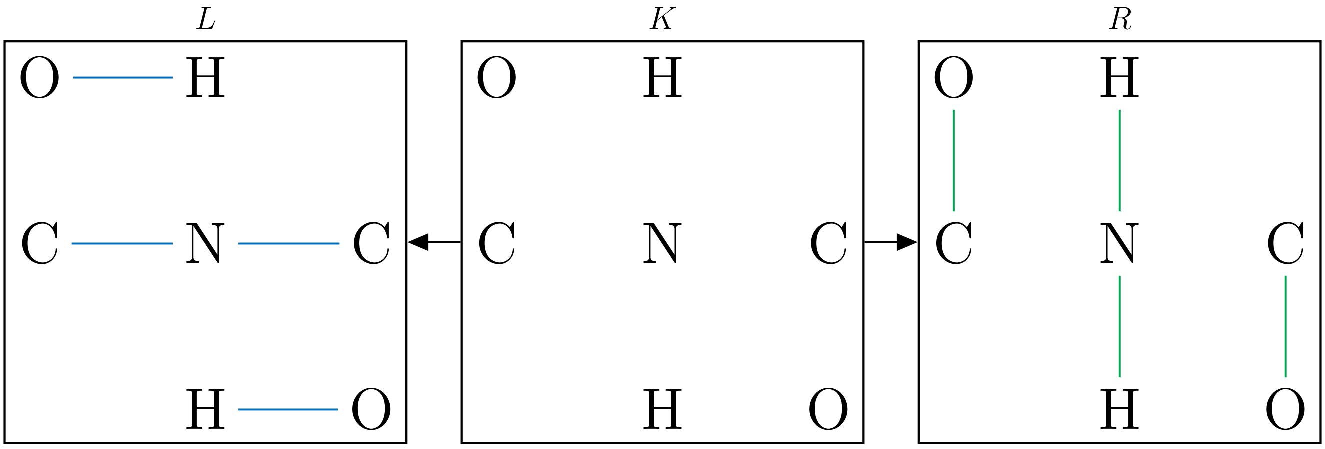

Files: out/201_r_40_10300000_{L, K, R}

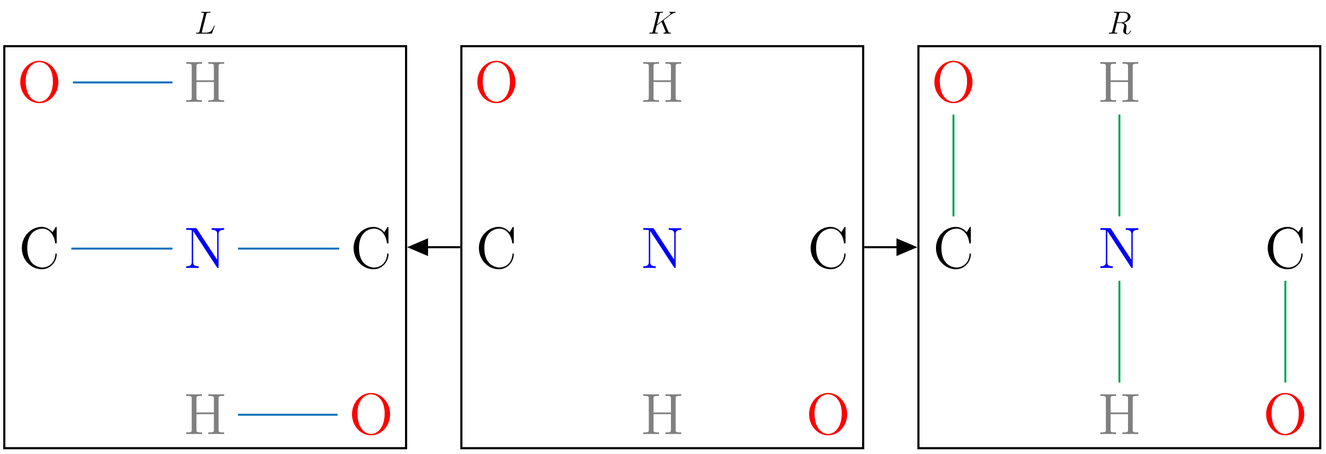

Files: out/203_r_40_11300100_{L, K, R}

0.0.42 41

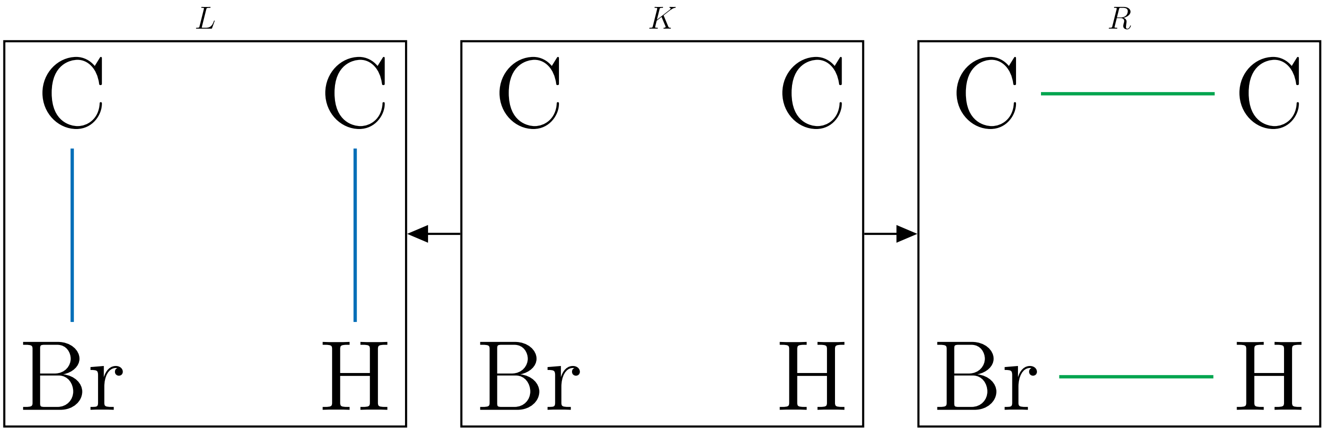

Files: out/206_r_41_10300000_{L, K, R}

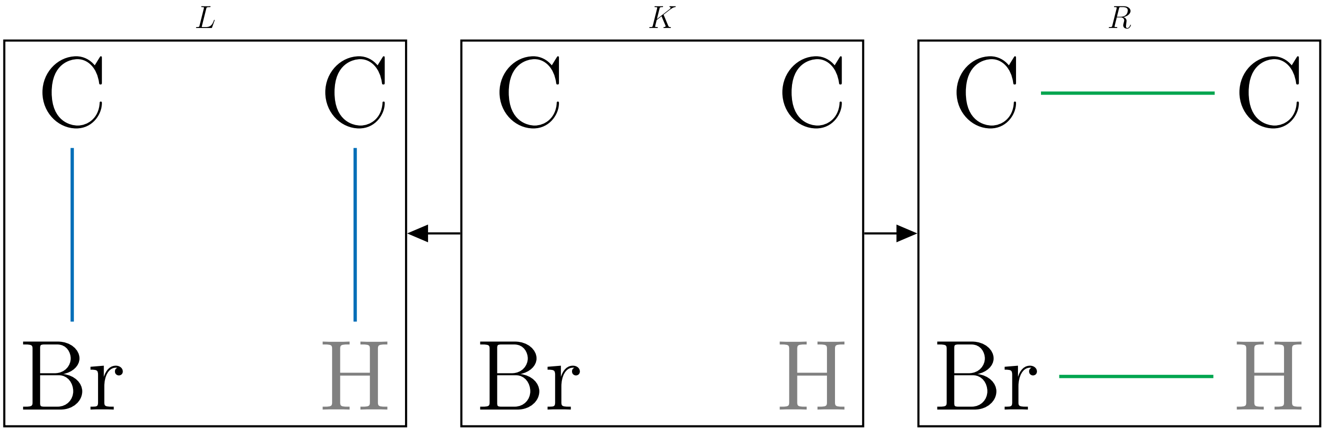

Files: out/208_r_41_11300100_{L, K, R}

0.0.43 42

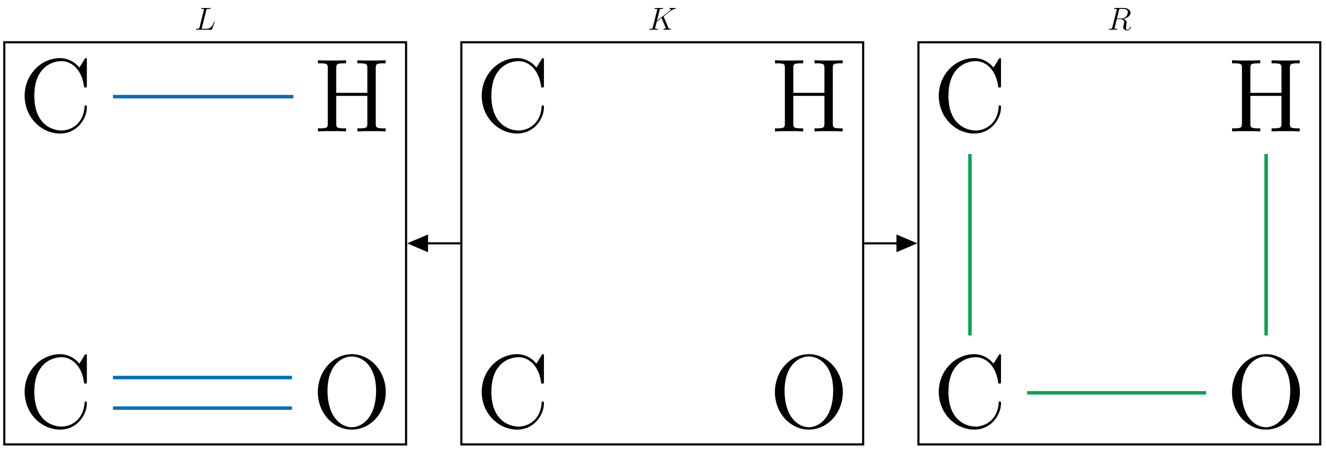

Files: out/211_r_42_10300000_{L, K, R}

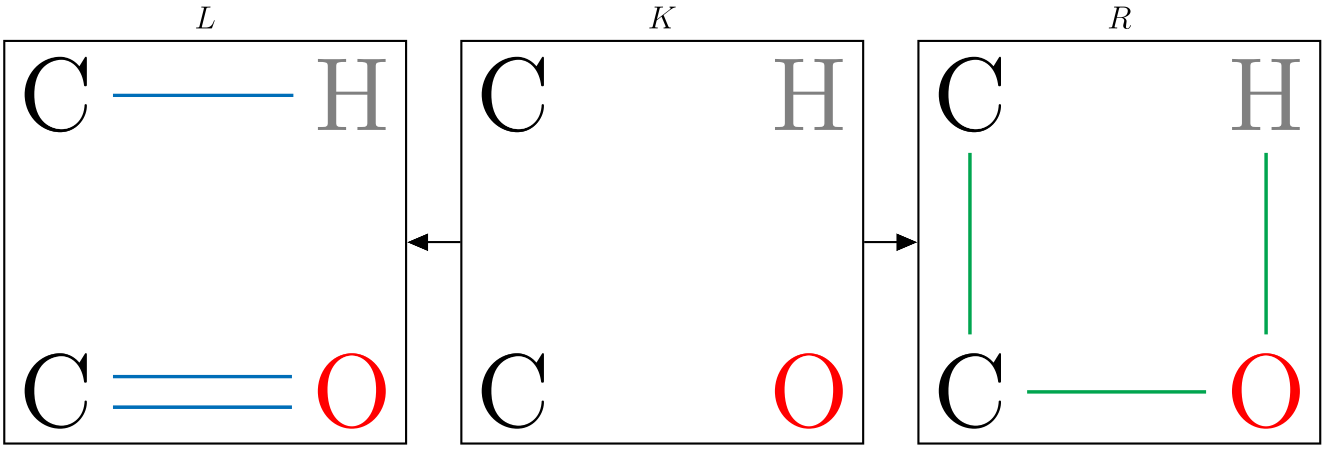

Files: out/213_r_42_11300100_{L, K, R}

0.0.44 43

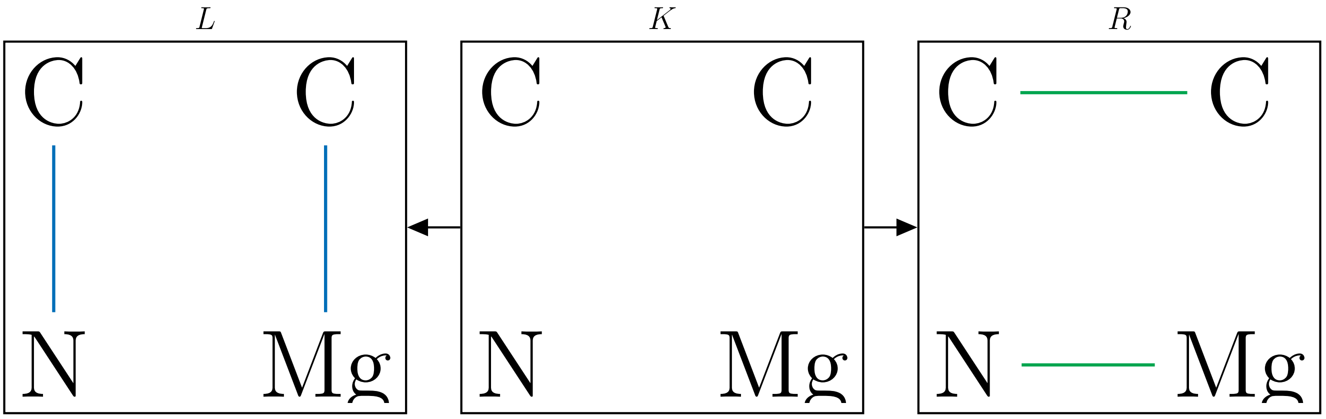

Files: out/216_r_43_10300000_{L, K, R}

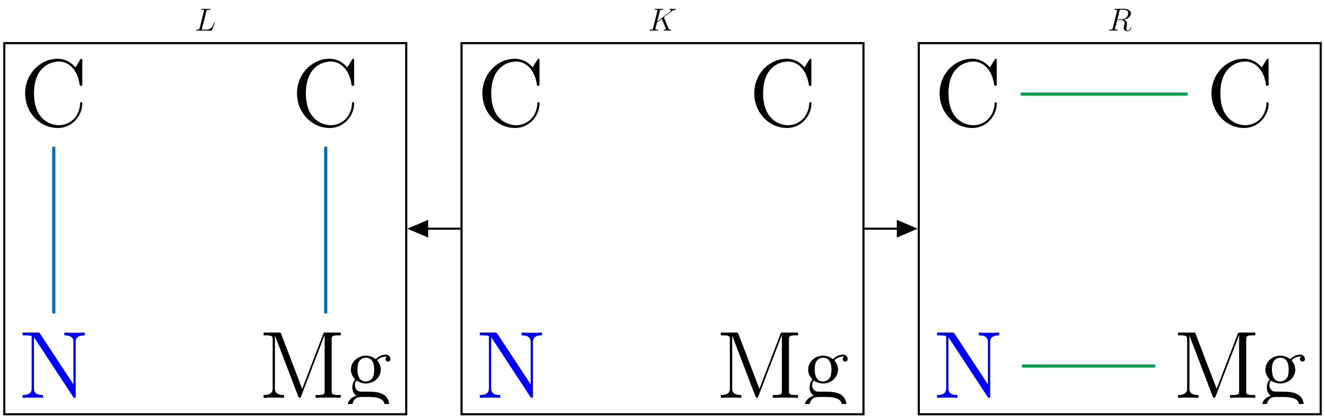

Files: out/218_r_43_11300100_{L, K, R}

0.0.45 44

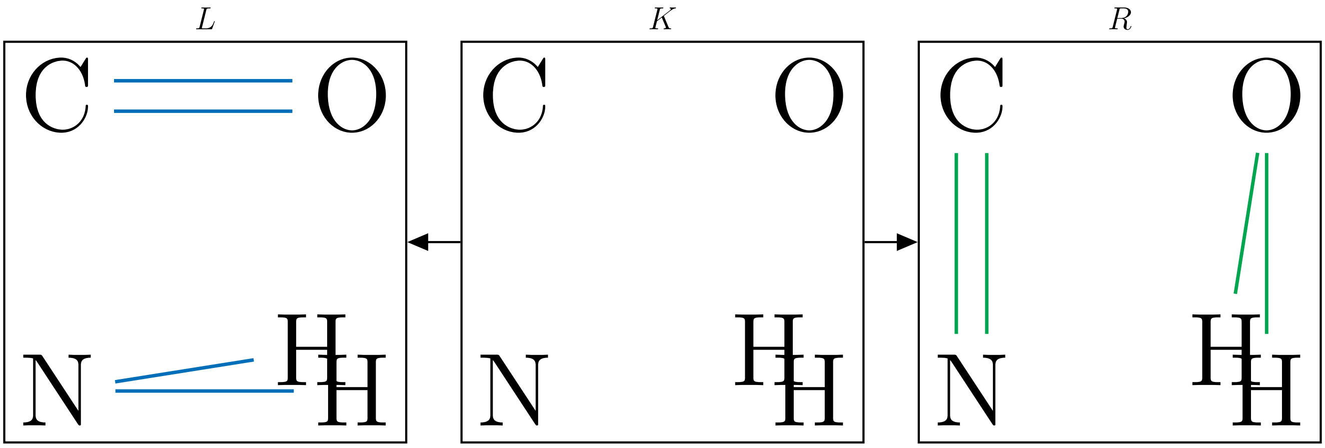

Files: out/221_r_44_10300000_{L, K, R}

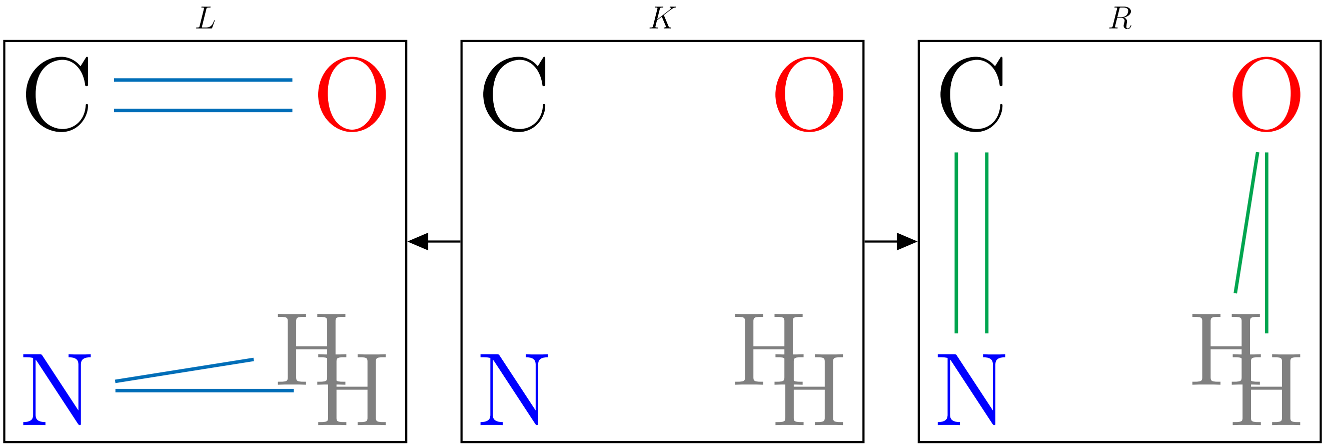

Files: out/223_r_44_11300100_{L, K, R}

0.0.46 45

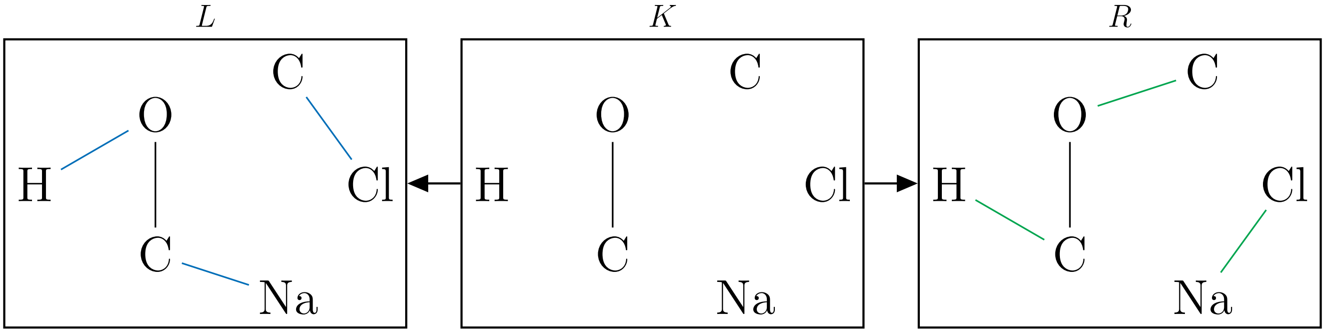

Files: out/226_r_45_10300000_{L, K, R}

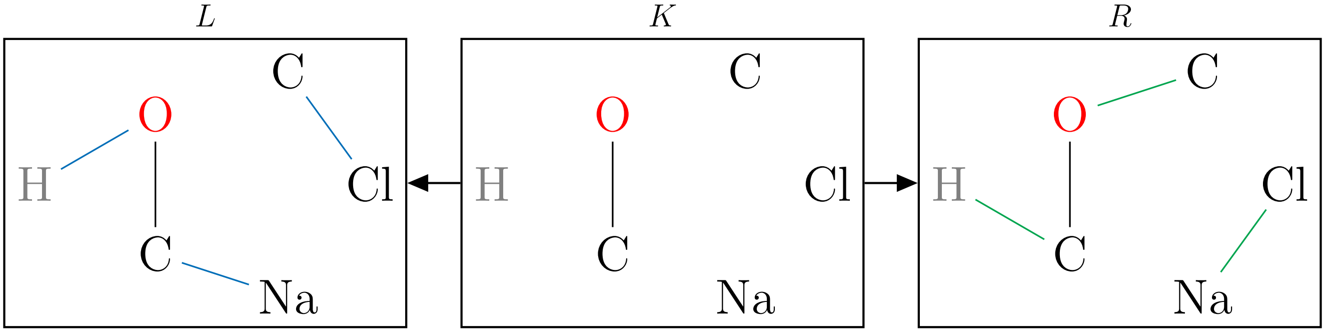

Files: out/228_r_45_11300100_{L, K, R}

0.0.47 46

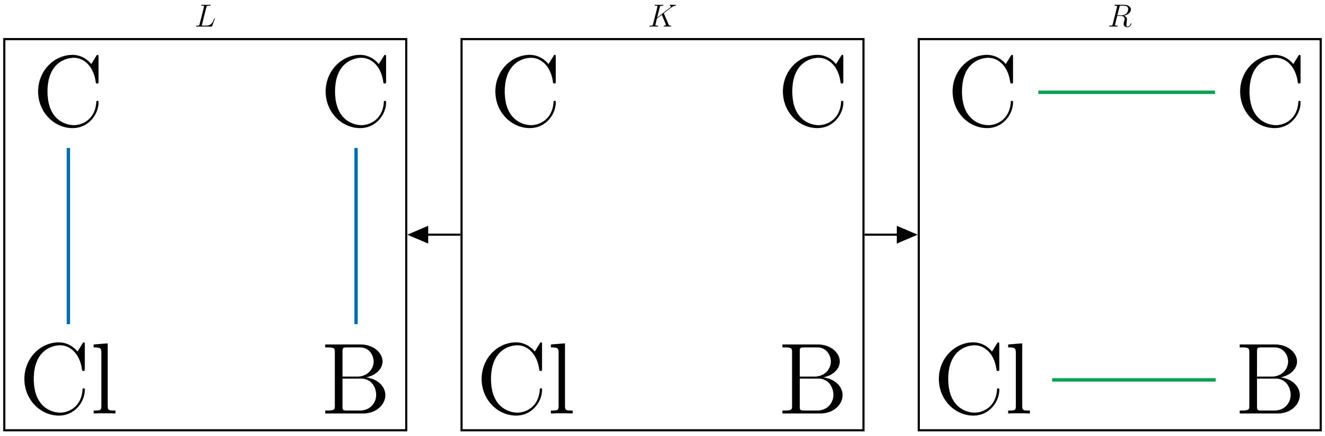

Files: out/231_r_46_10300000_{L, K, R}

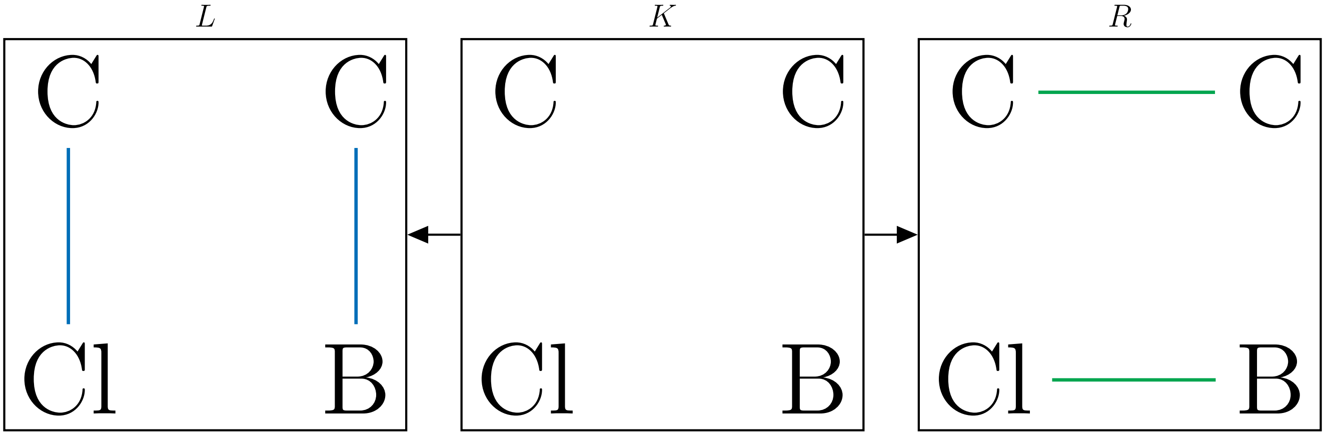

Files: out/233_r_46_11300100_{L, K, R}

0.0.48 47

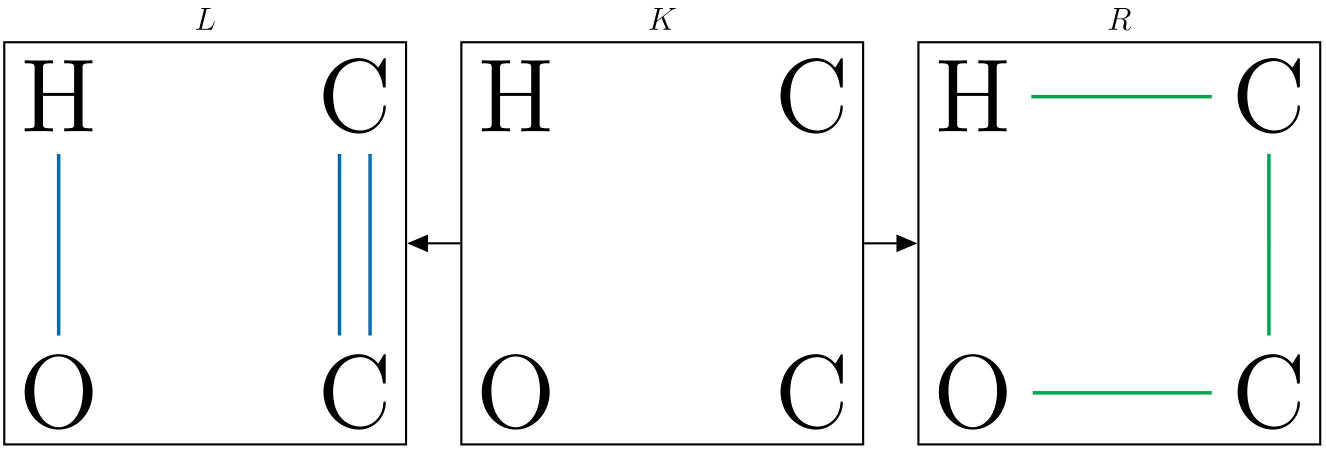

Files: out/236_r_47_10300000_{L, K, R}

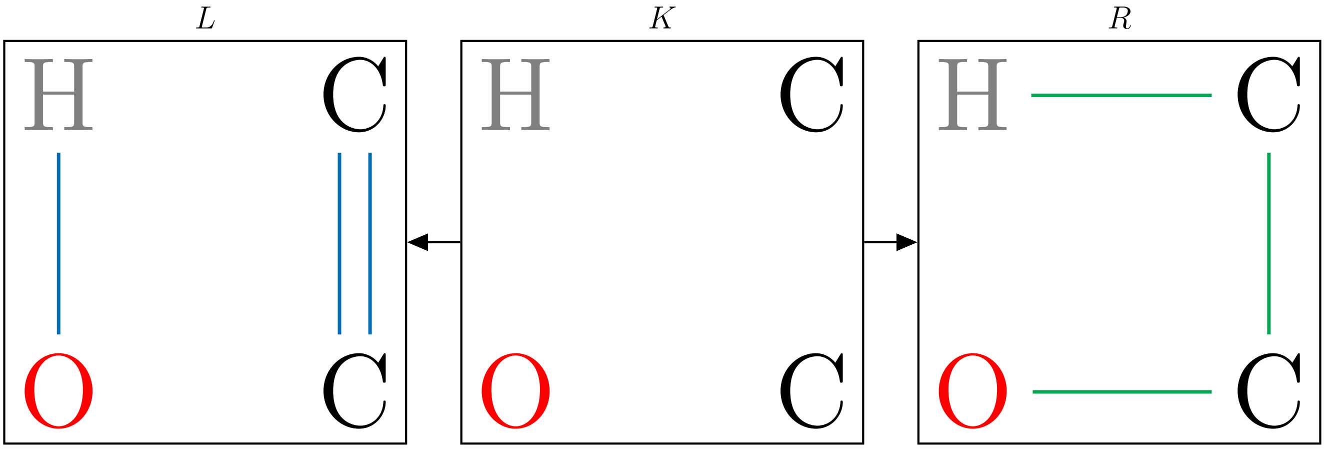

Files: out/238_r_47_11300100_{L, K, R}

0.0.49 48

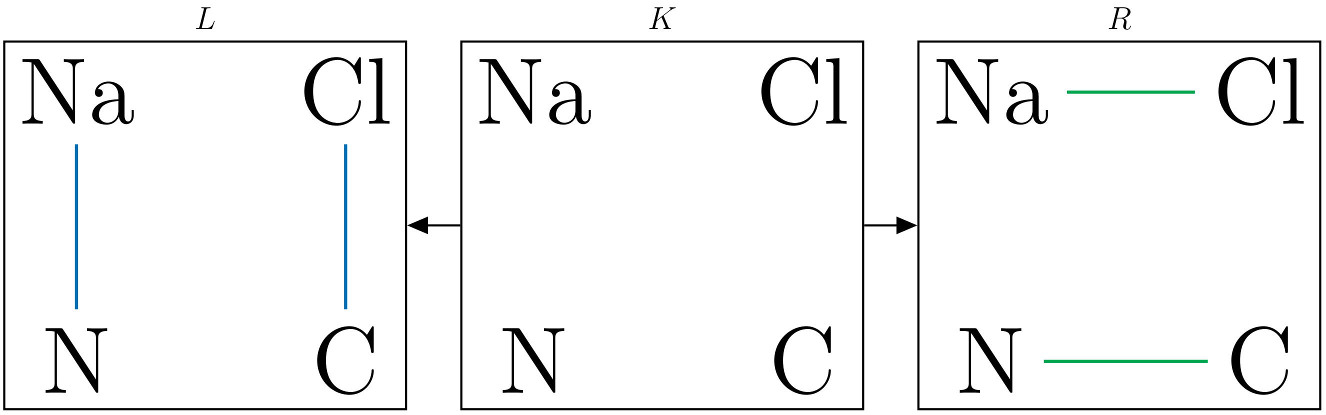

Files: out/241_r_48_10300000_{L, K, R}

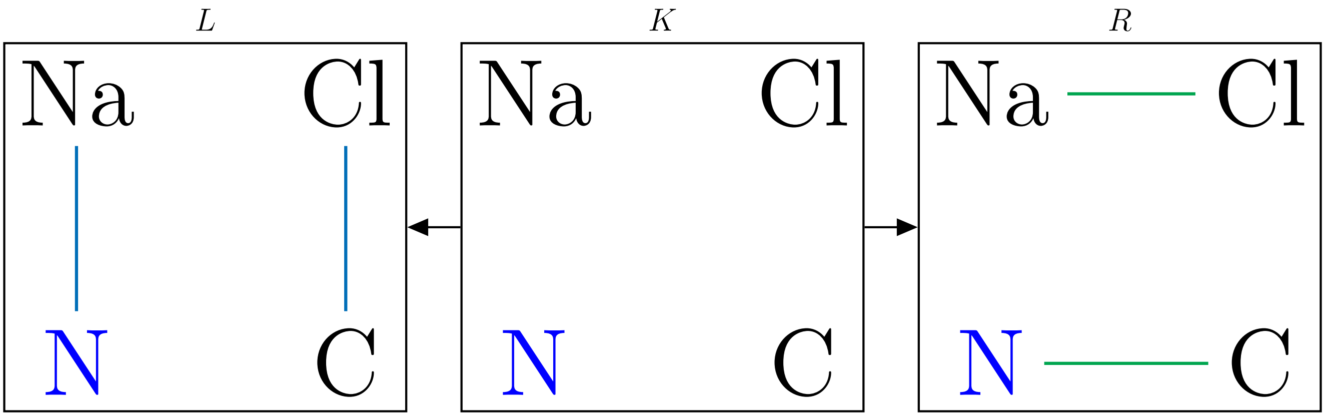

Files: out/243_r_48_11300100_{L, K, R}

0.0.50 49

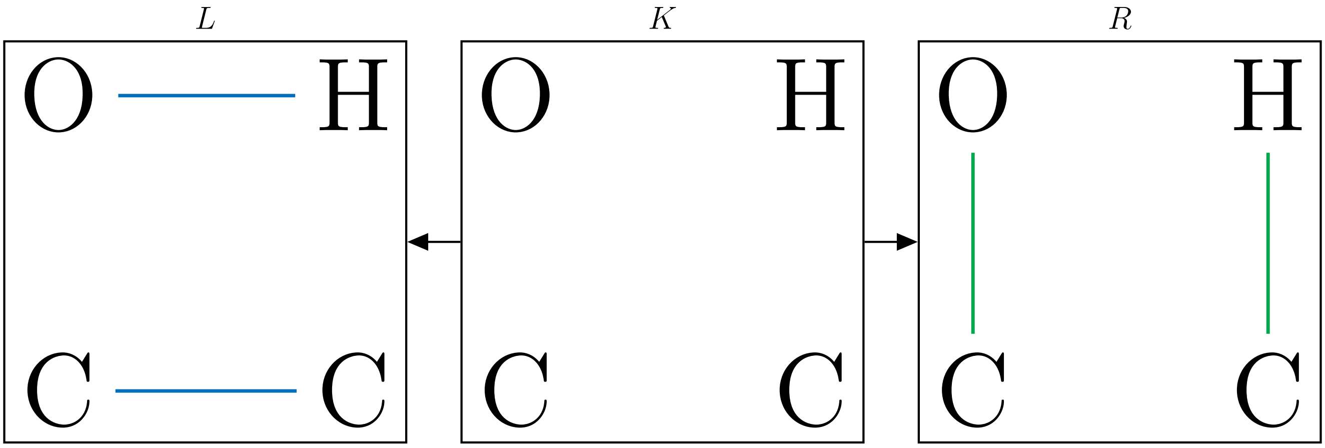

Files: out/246_r_49_10300000_{L, K, R}

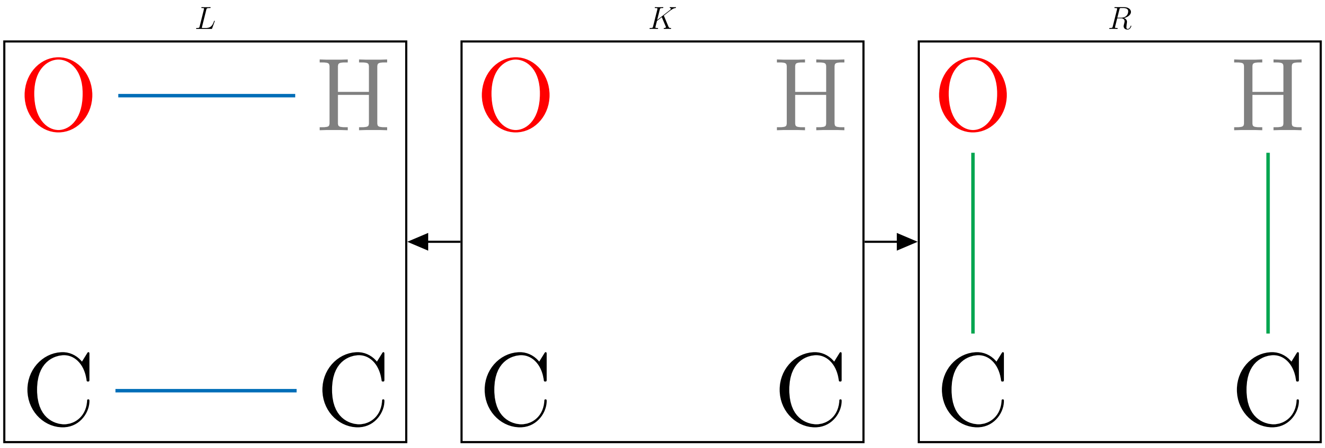

Files: out/248_r_49_11300100_{L, K, R}

0.0.51 50

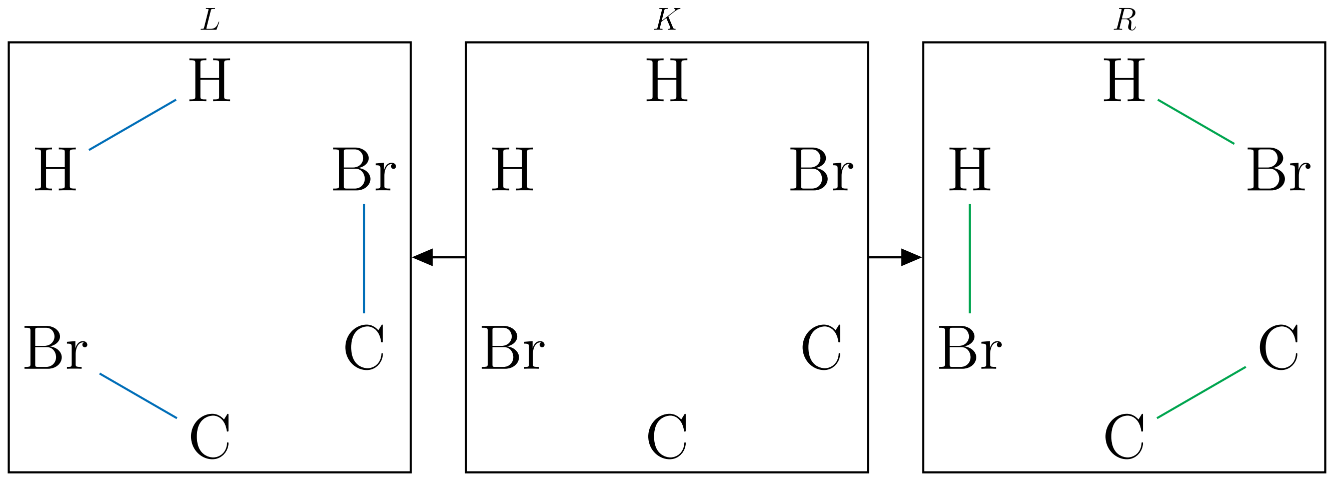

Files: out/251_r_50_10300000_{L, K, R}

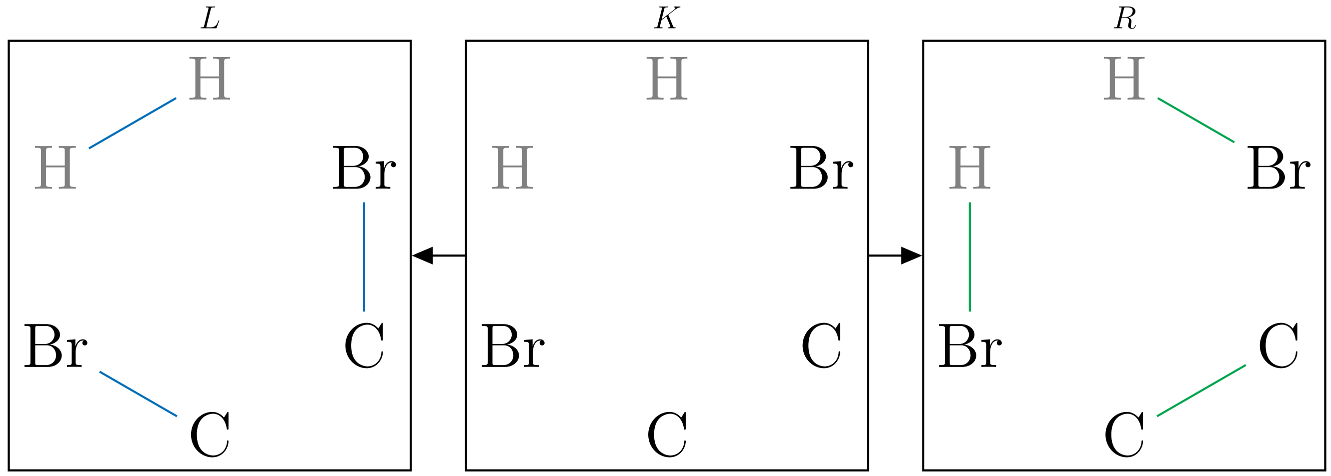

Files: out/253_r_50_11300100_{L, K, R}

0.0.52 51

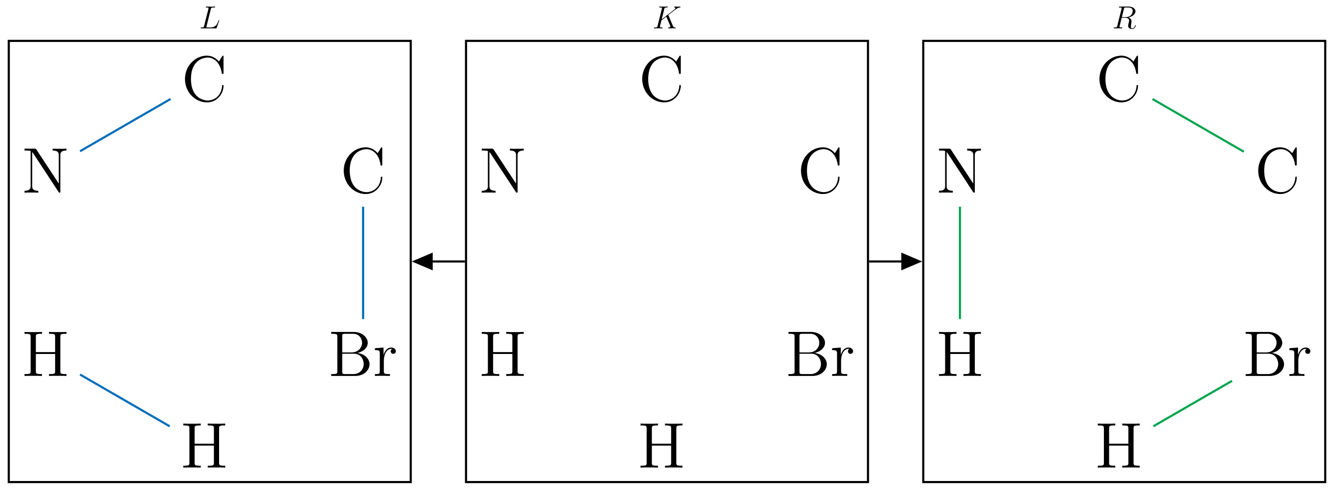

Files: out/256_r_51_10300000_{L, K, R}

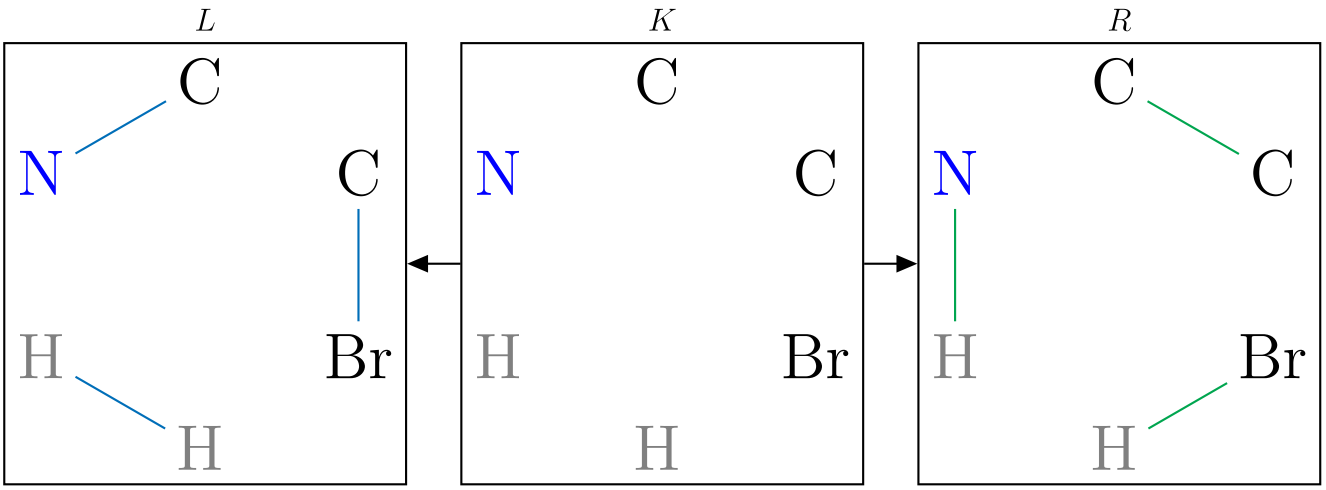

Files: out/258_r_51_11300100_{L, K, R}

0.0.53 52

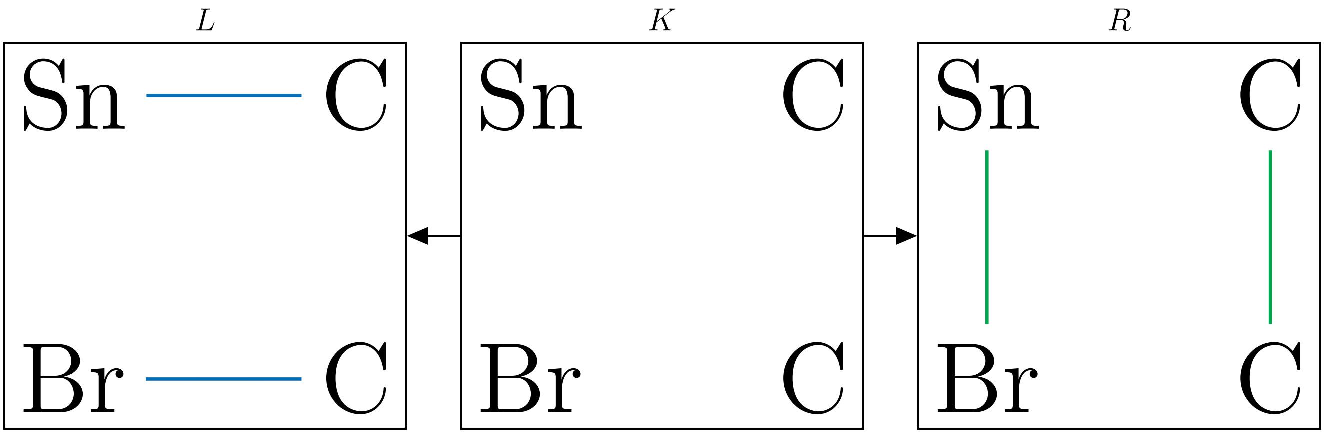

Files: out/261_r_52_10300000_{L, K, R}

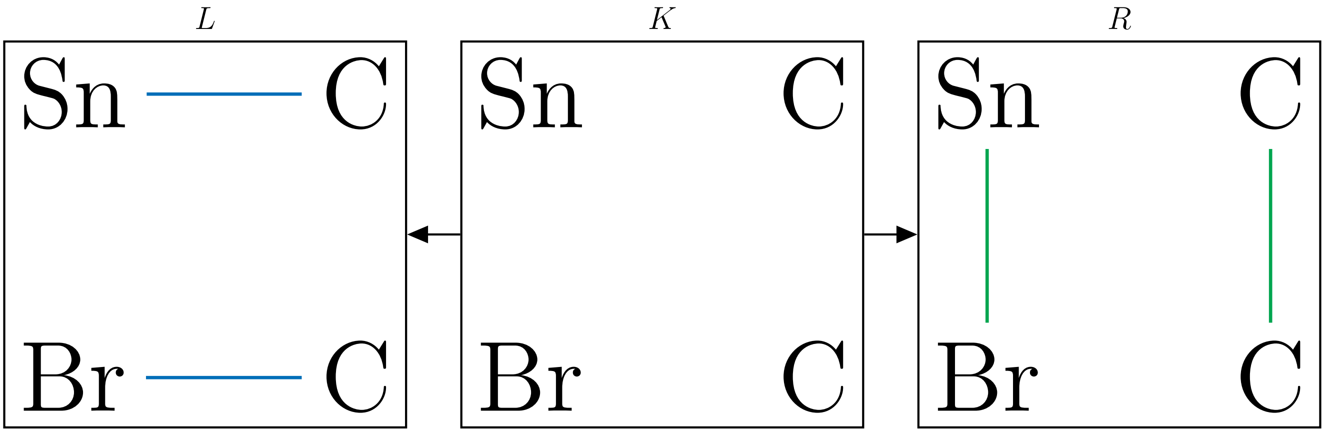

Files: out/263_r_52_11300100_{L, K, R}

0.0.54 53

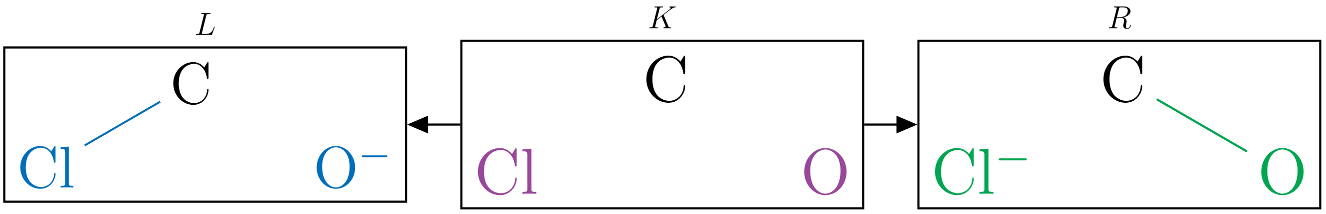

Files: out/266_r_53_10300000_{L, K, R}

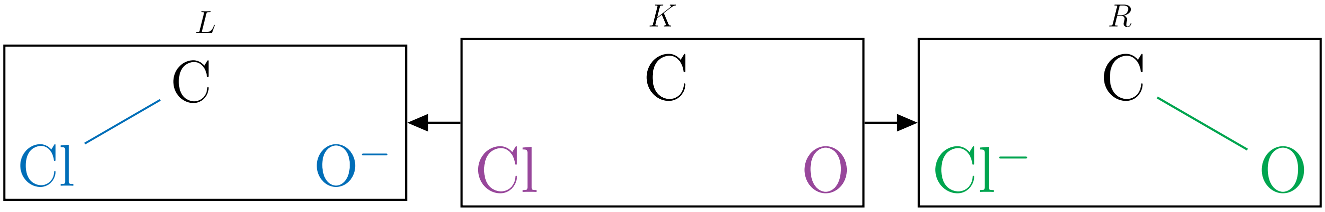

Files: out/268_r_53_11300100_{L, K, R}

0.0.55 54

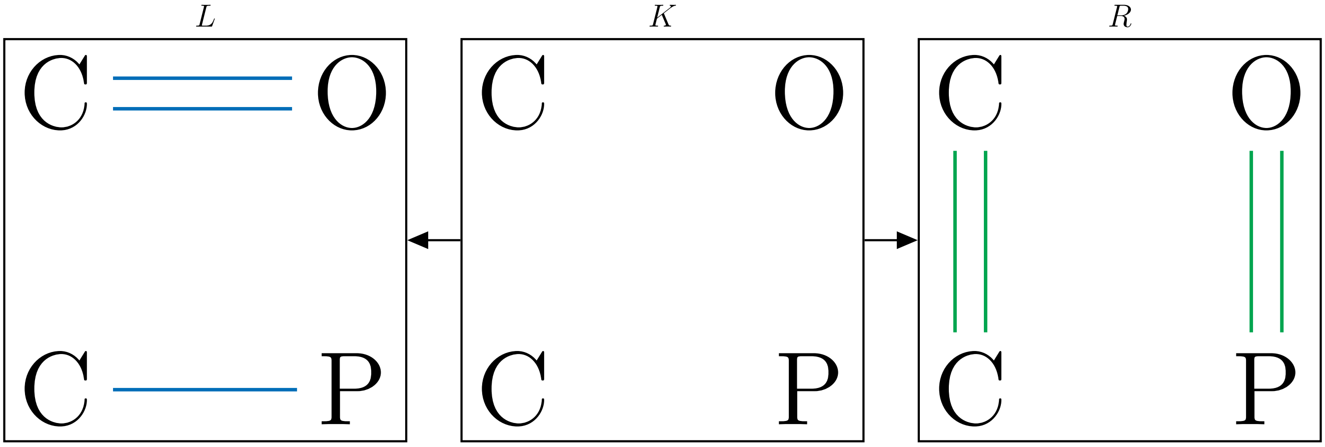

Files: out/271_r_54_10300000_{L, K, R}

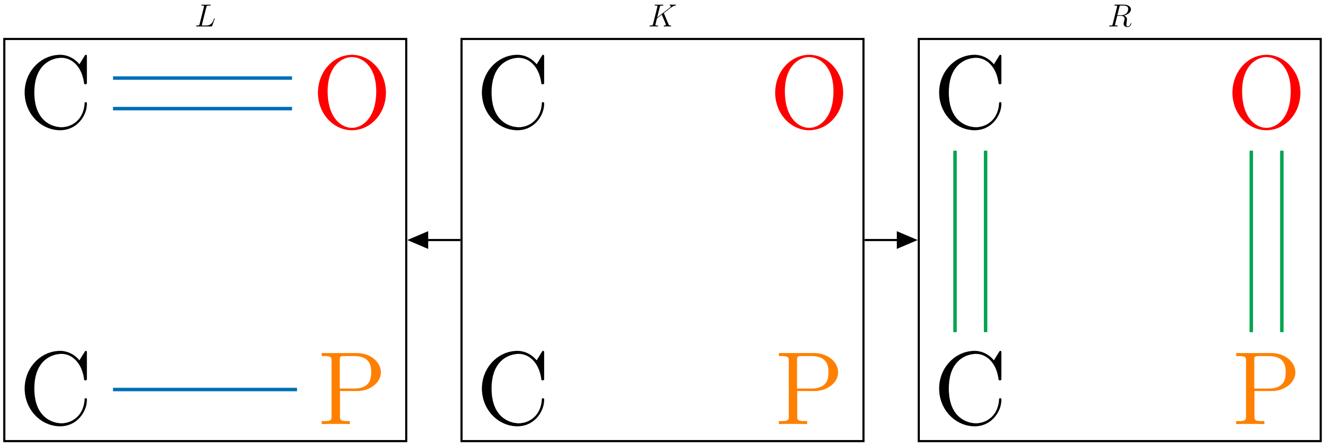

Files: out/273_r_54_11300100_{L, K, R}

0.0.56 55

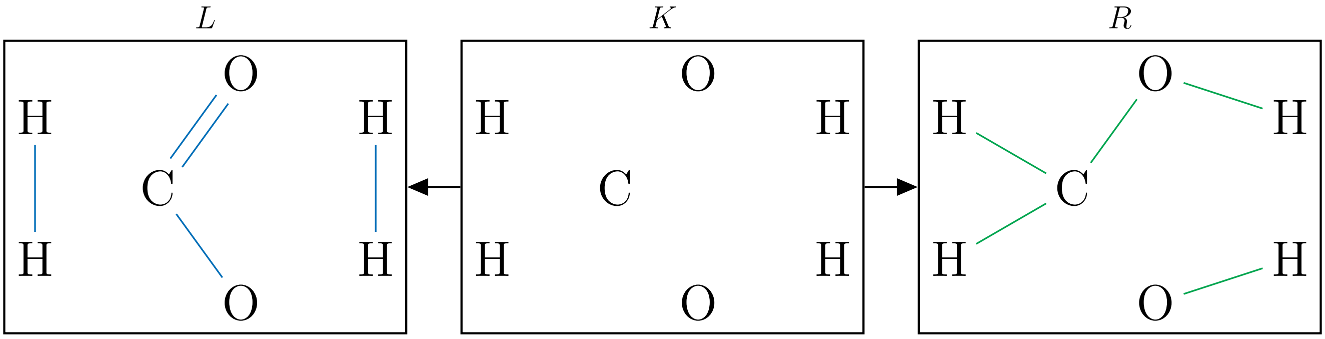

Files: out/276_r_55_10300000_{L, K, R}

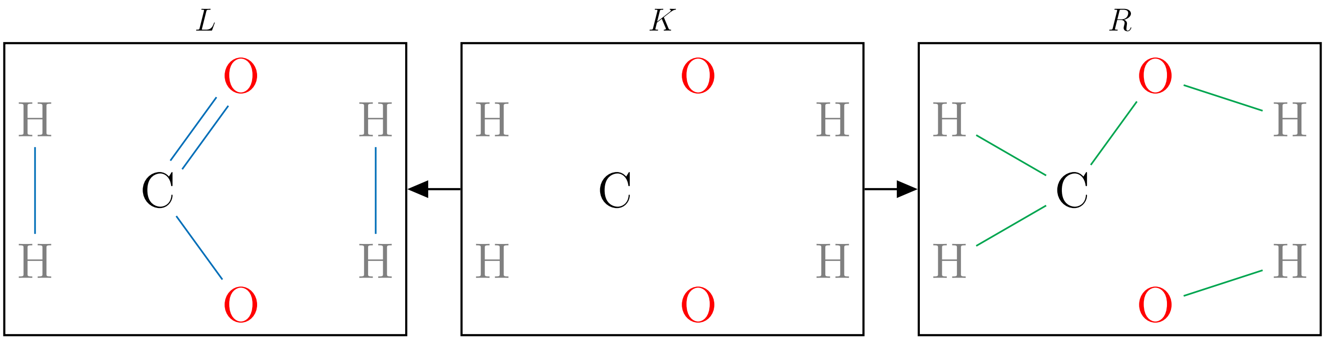

Files: out/278_r_55_11300100_{L, K, R}

0.0.57 56

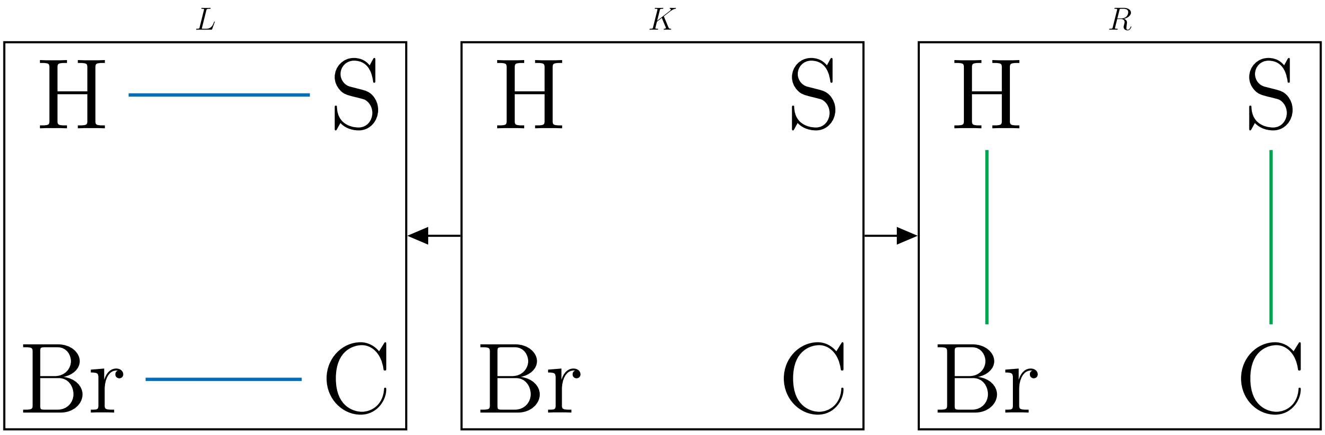

Files: out/281_r_56_10300000_{L, K, R}

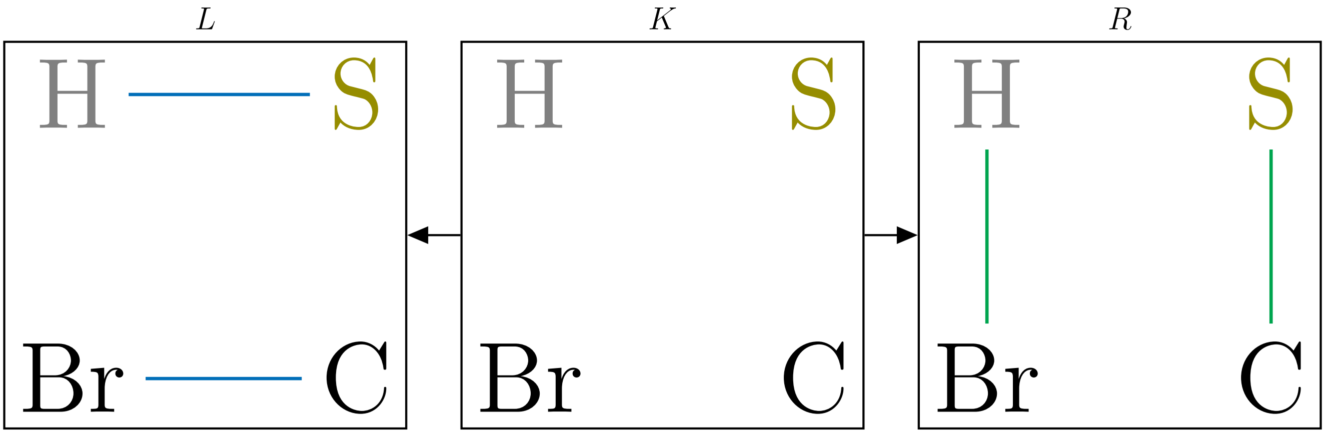

Files: out/283_r_56_11300100_{L, K, R}

0.0.58 57

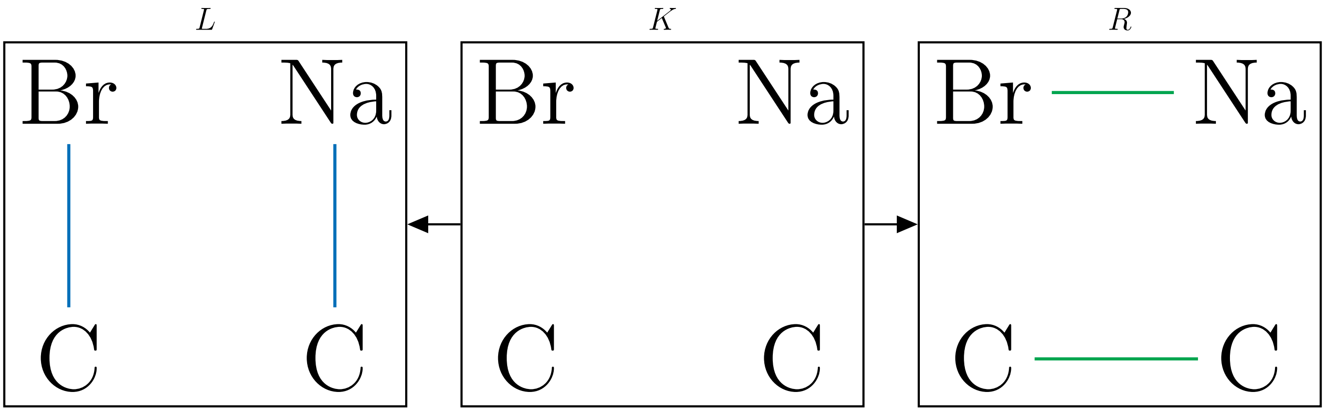

Files: out/286_r_57_10300000_{L, K, R}

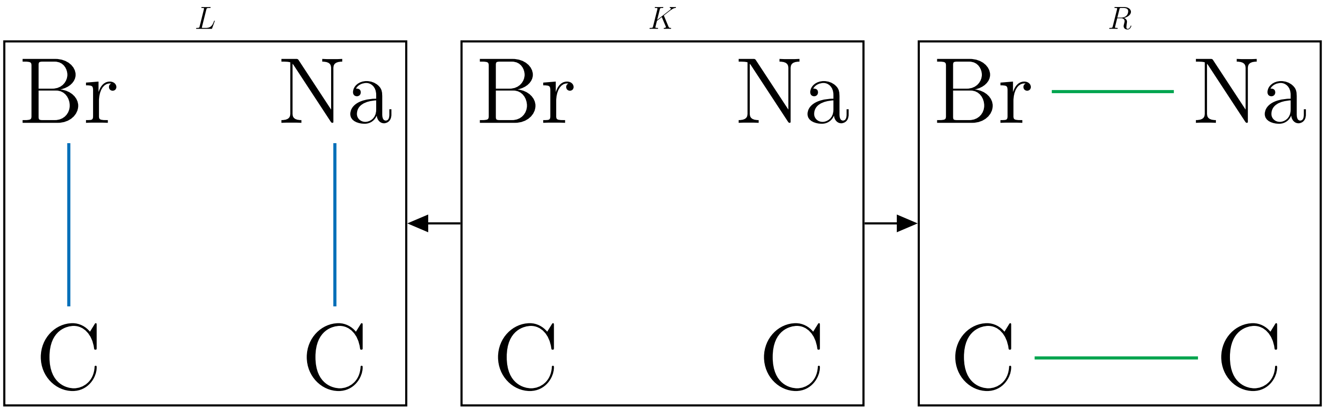

Files: out/288_r_57_11300100_{L, K, R}

0.0.59 58

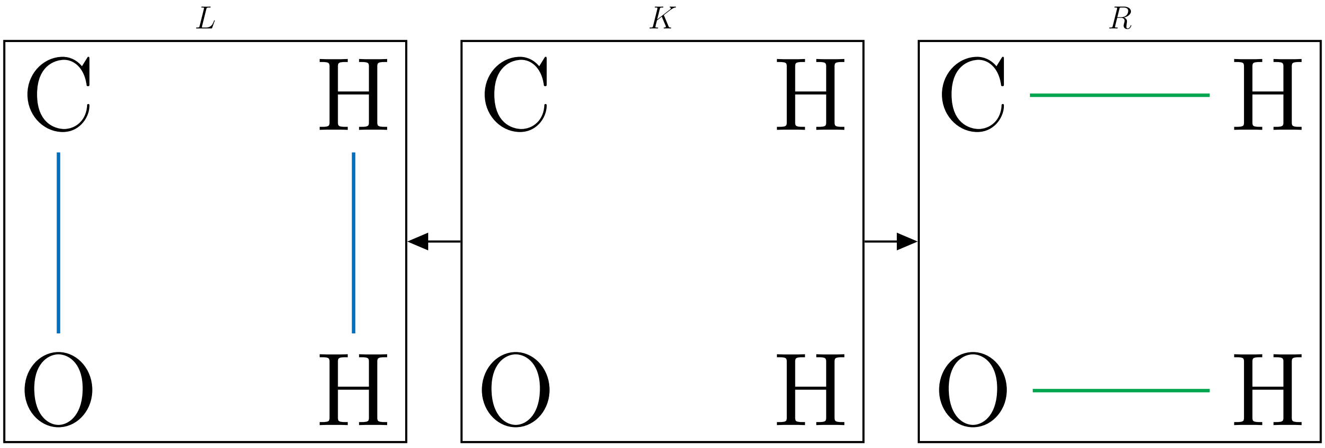

Files: out/291_r_58_10300000_{L, K, R}

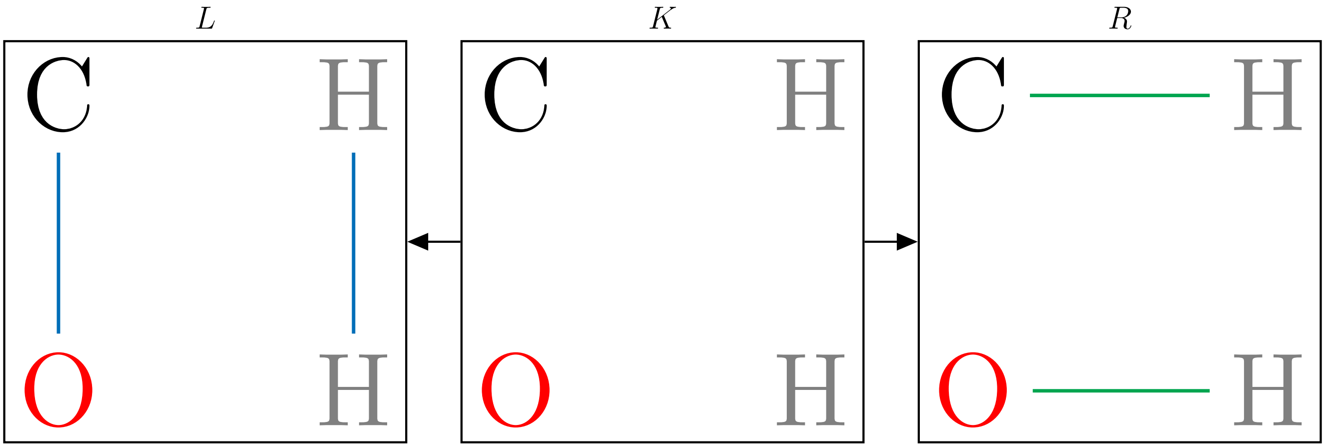

Files: out/293_r_58_11300100_{L, K, R}

0.0.60 59

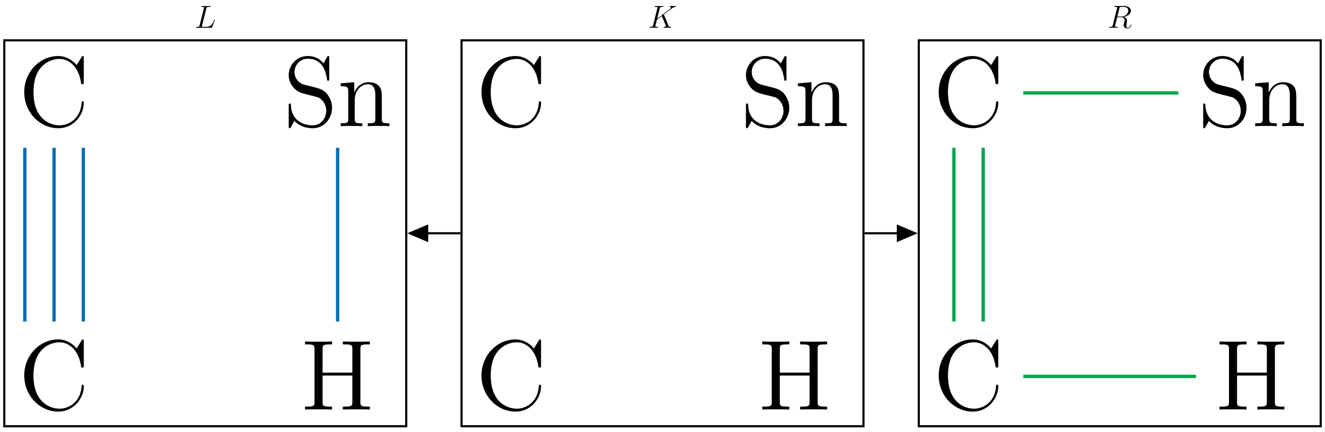

Files: out/296_r_59_10300000_{L, K, R}

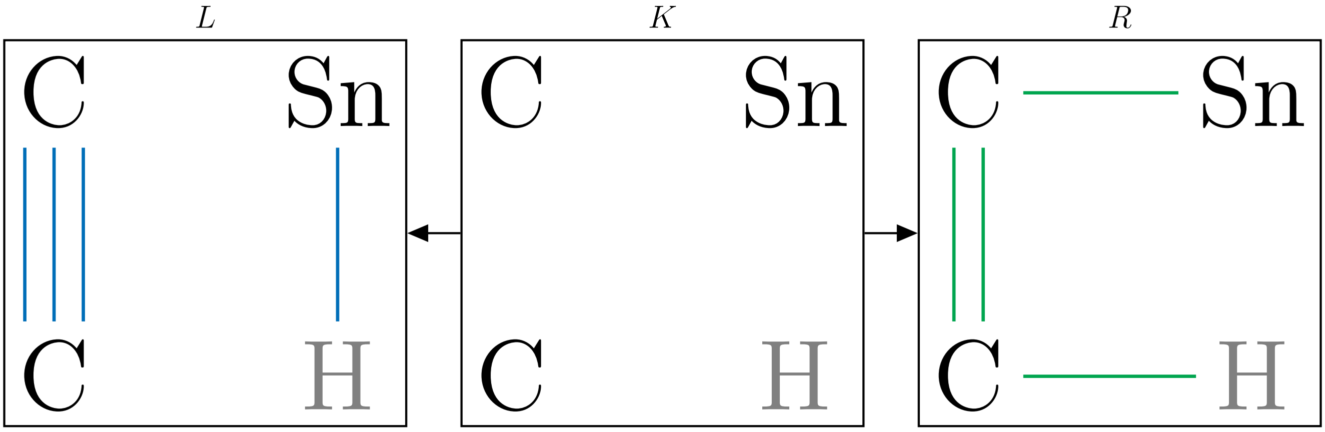

Files: out/298_r_59_11300100_{L, K, R}

0.0.61 60

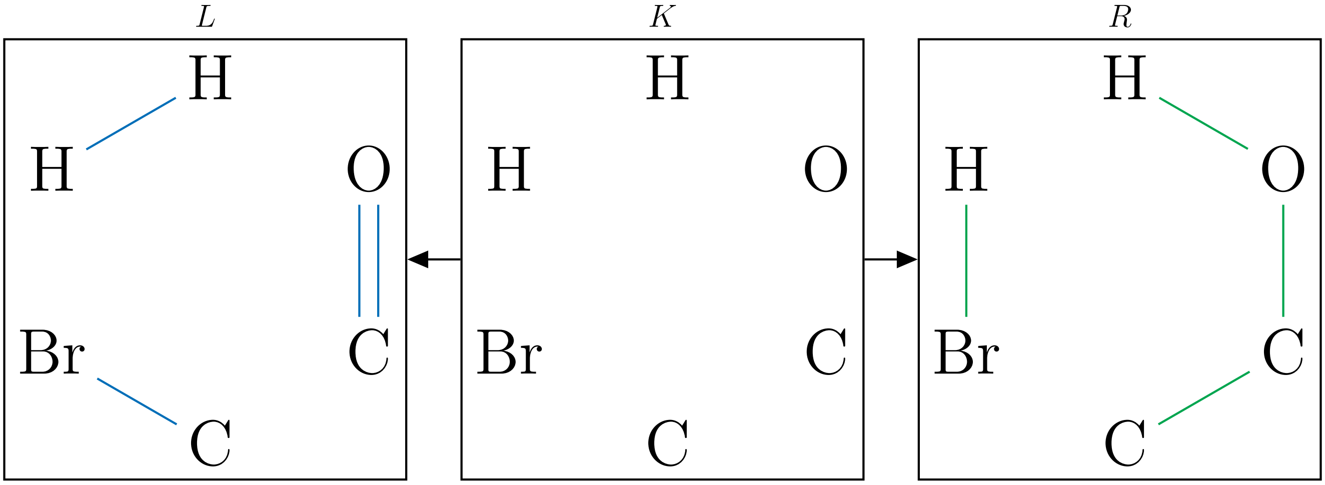

Files: out/301_r_60_10300000_{L, K, R}

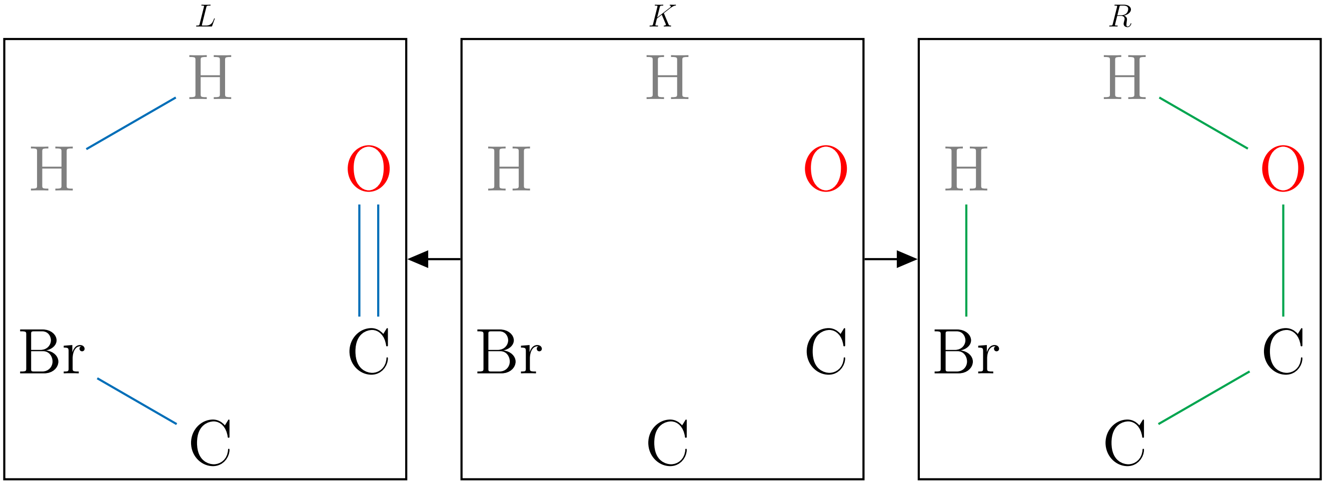

Files: out/303_r_60_11300100_{L, K, R}

0.0.62 61

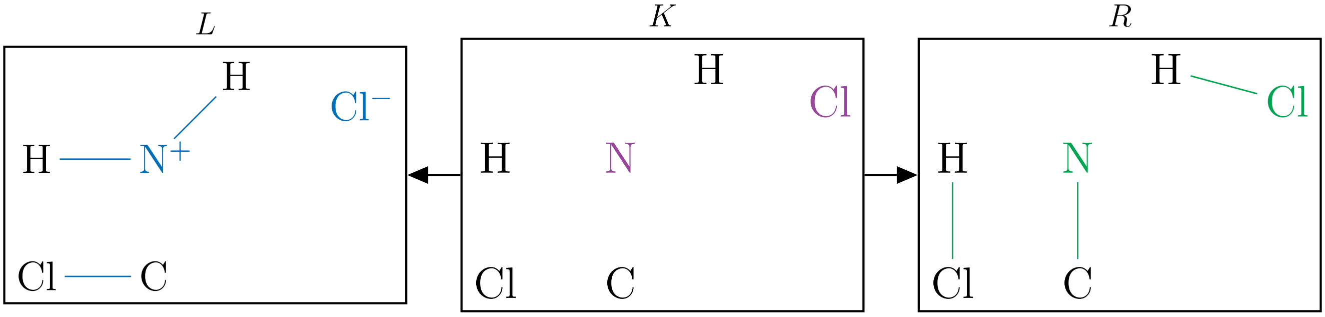

Files: out/306_r_61_10300000_{L, K, R}

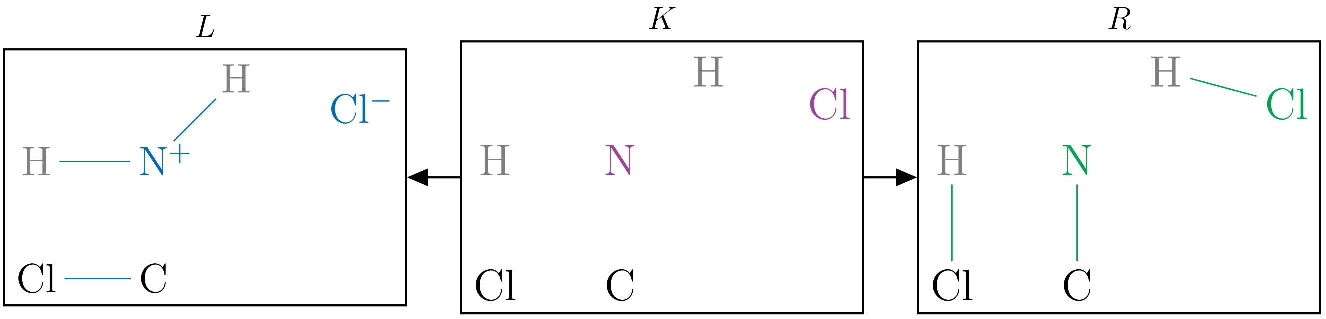

Files: out/308_r_61_11300100_{L, K, R}

0.0.63 62

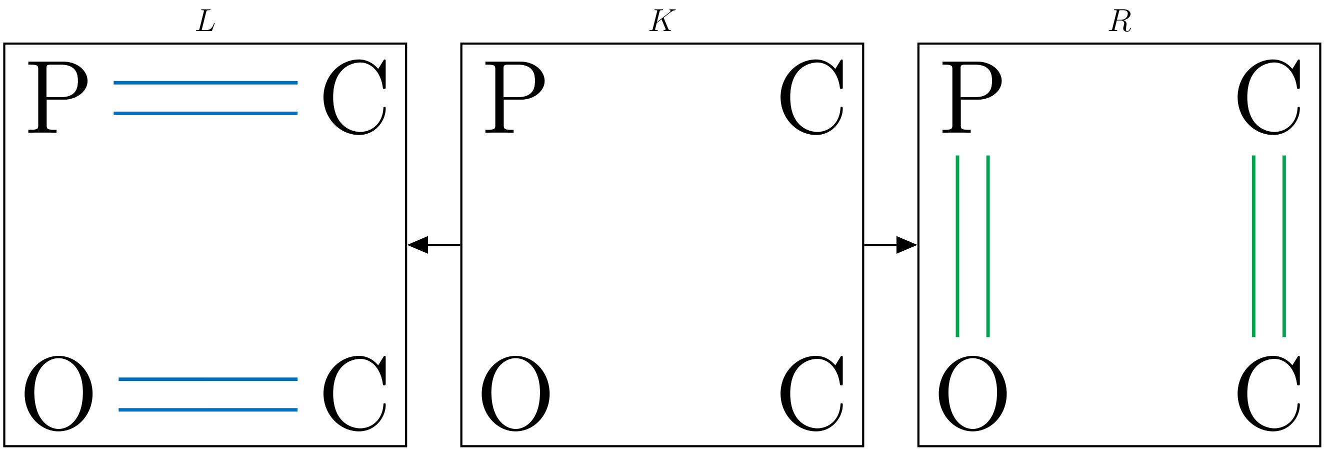

Files: out/311_r_62_10300000_{L, K, R}

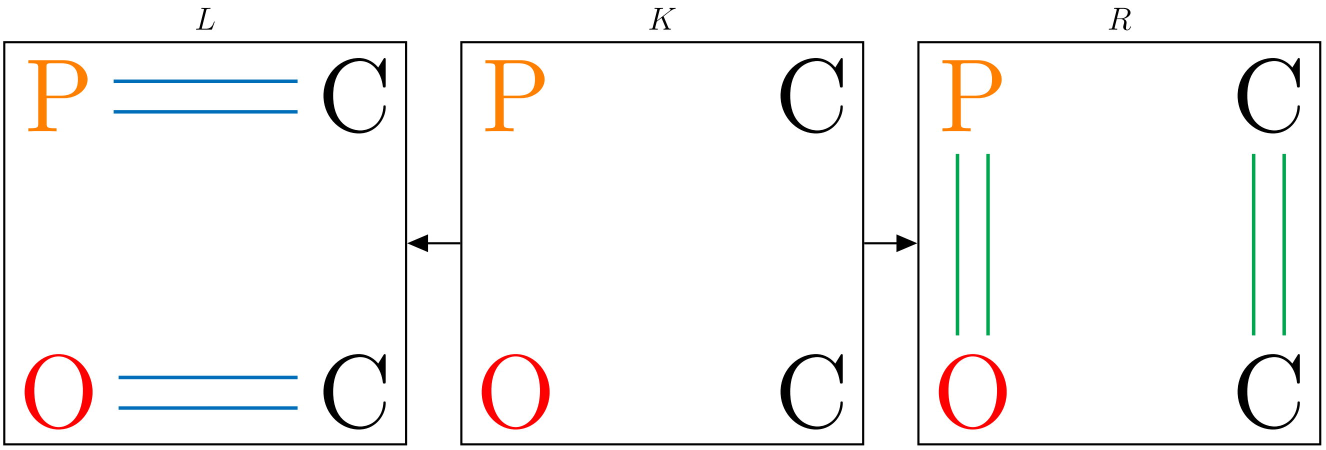

Files: out/313_r_62_11300100_{L, K, R}

0.0.64 63

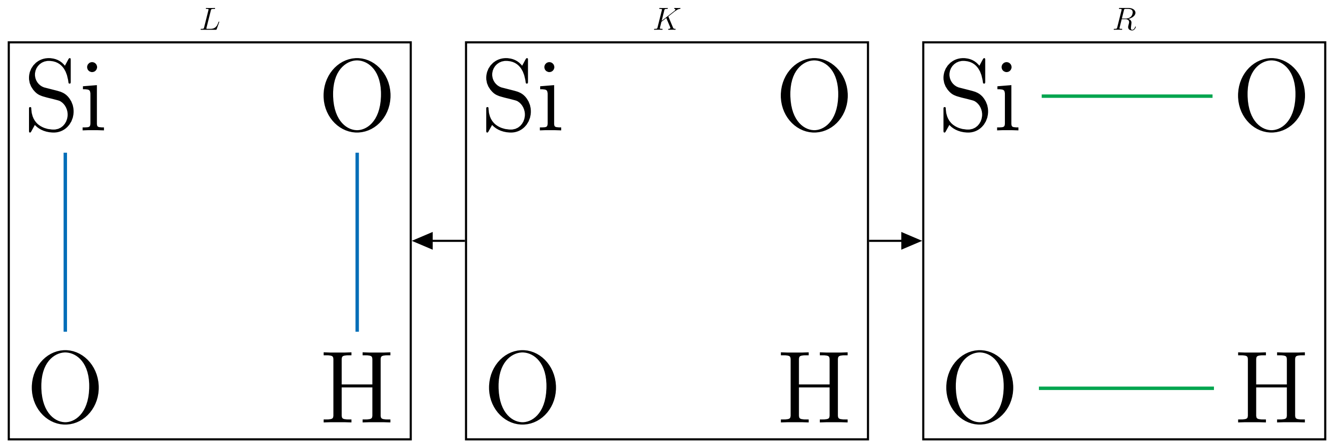

Files: out/316_r_63_10300000_{L, K, R}

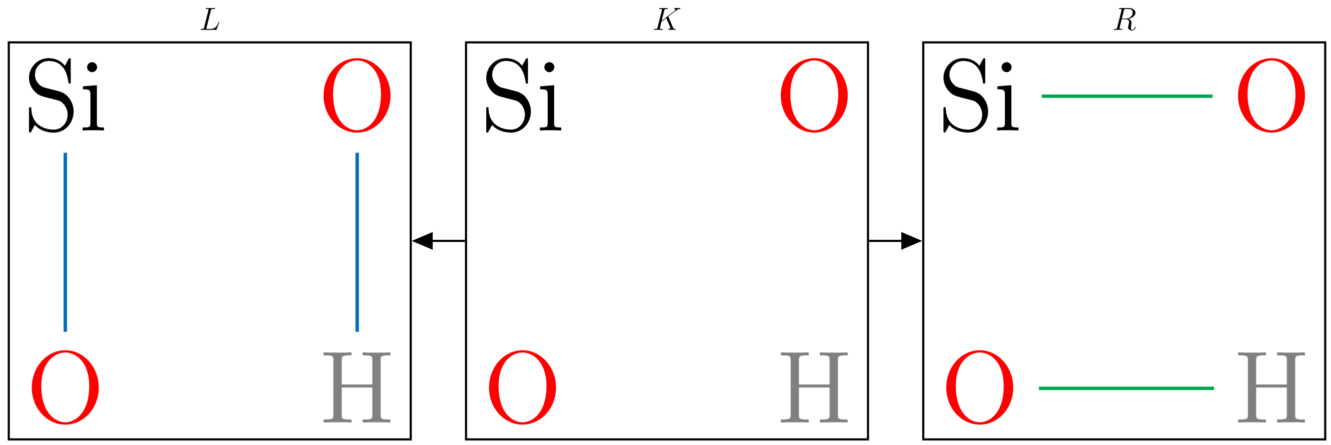

Files: out/318_r_63_11300100_{L, K, R}

0.0.65 64

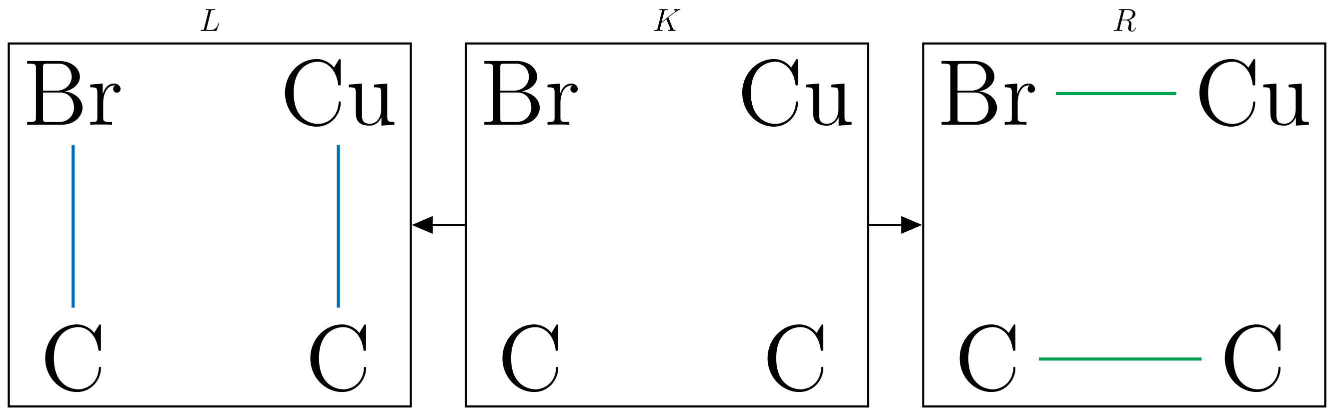

Files: out/321_r_64_10300000_{L, K, R}

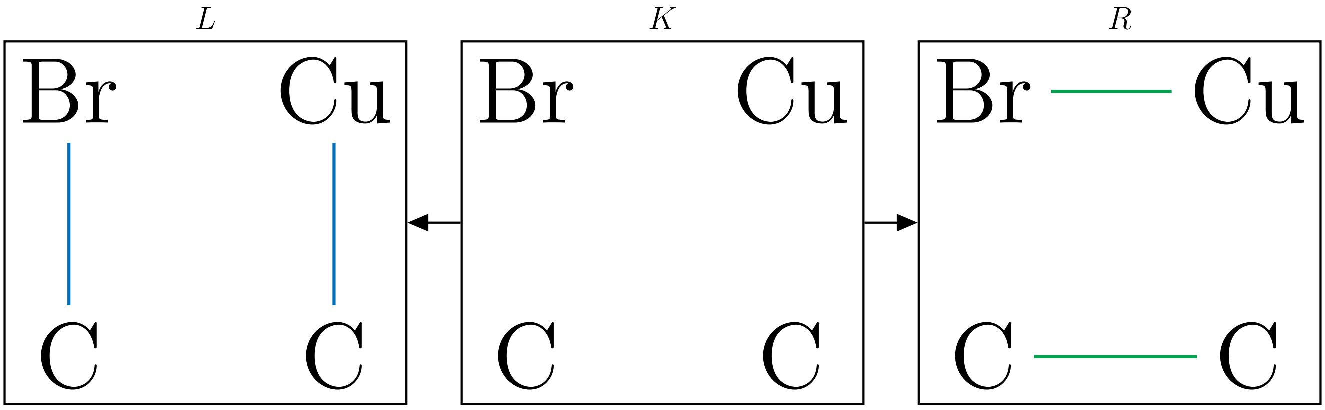

Files: out/323_r_64_11300100_{L, K, R}

0.0.66 65

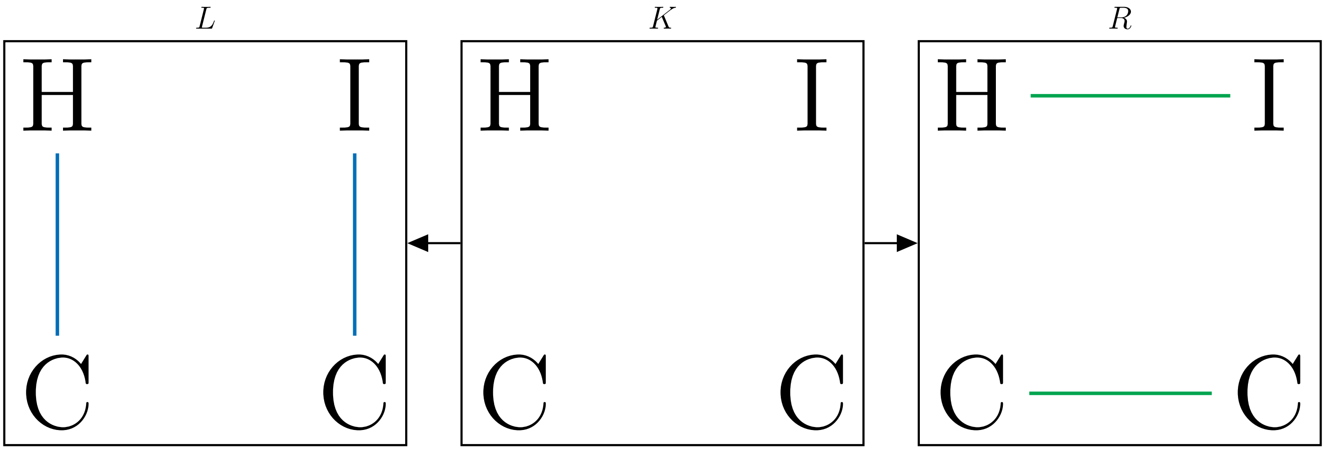

Files: out/326_r_65_10300000_{L, K, R}

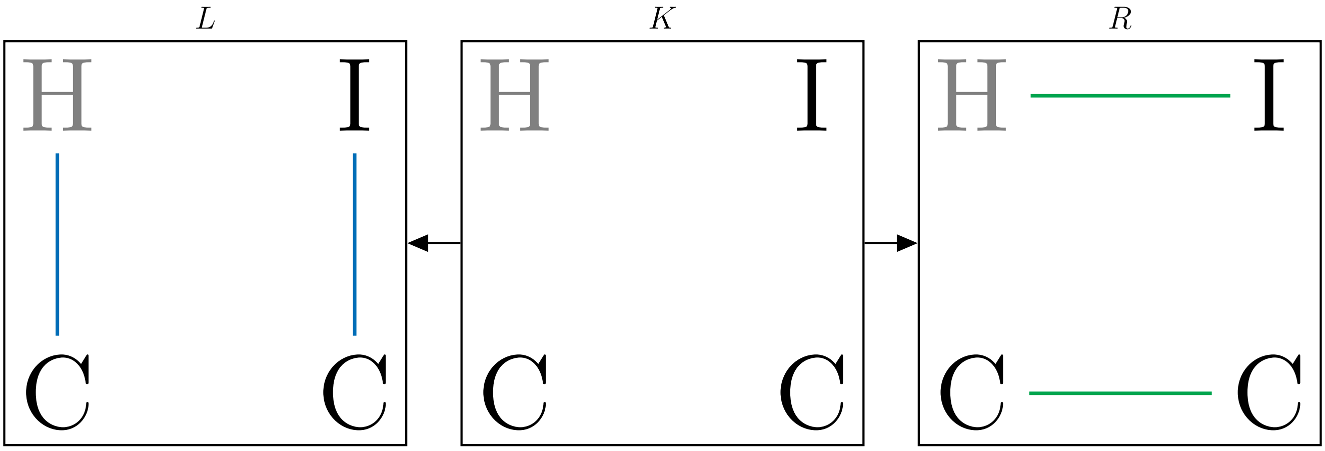

Files: out/328_r_65_11300100_{L, K, R}

0.0.67 66

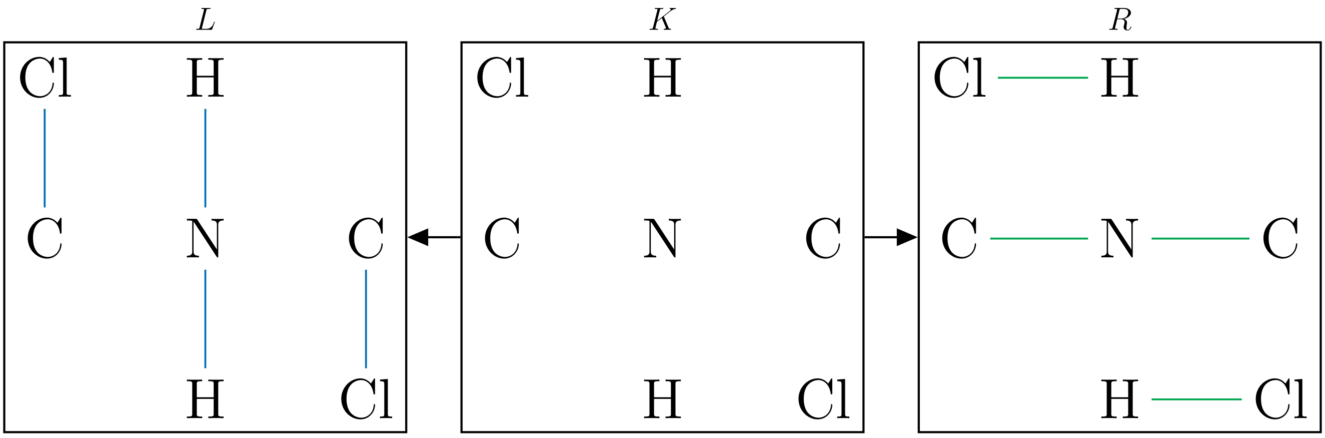

Files: out/331_r_66_10300000_{L, K, R}

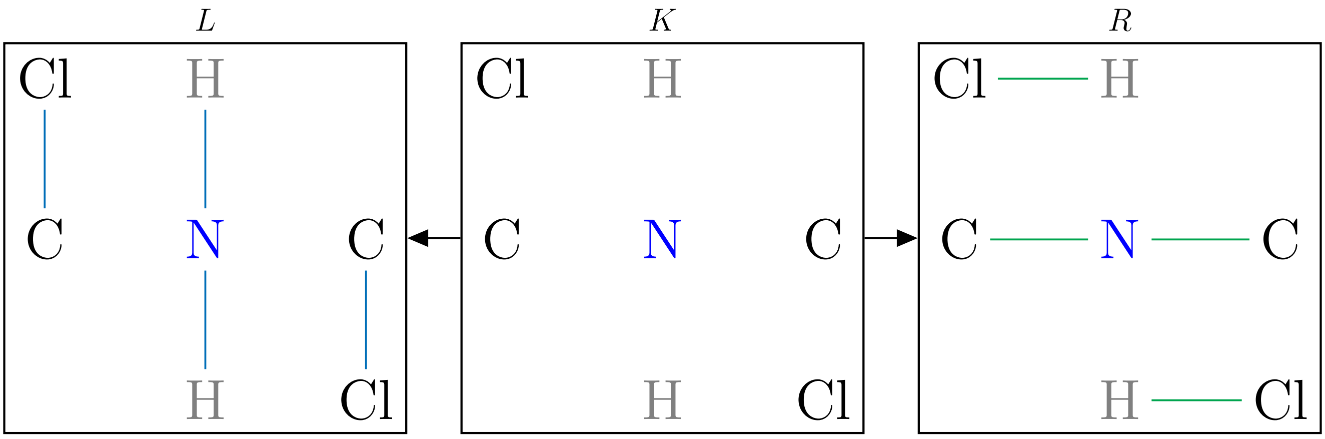

Files: out/333_r_66_11300100_{L, K, R}

0.0.68 67

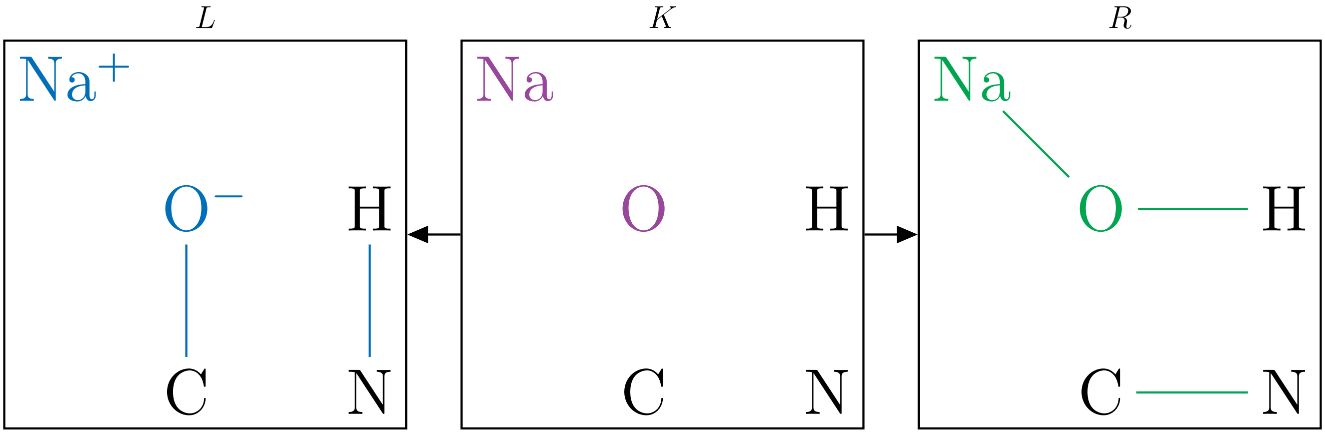

Files: out/336_r_67_10300000_{L, K, R}

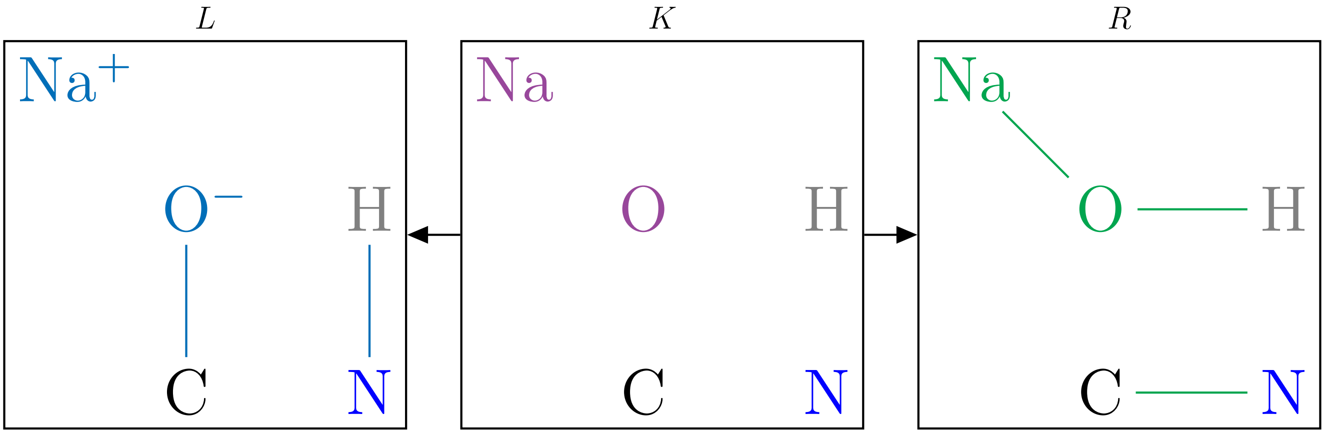

Files: out/338_r_67_11300100_{L, K, R}

0.0.69 68

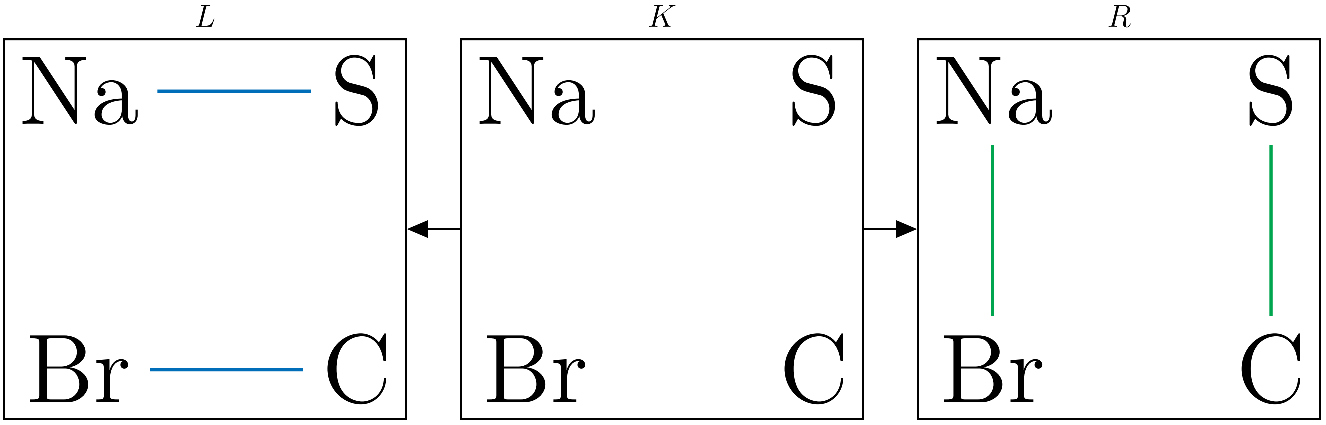

Files: out/341_r_68_10300000_{L, K, R}

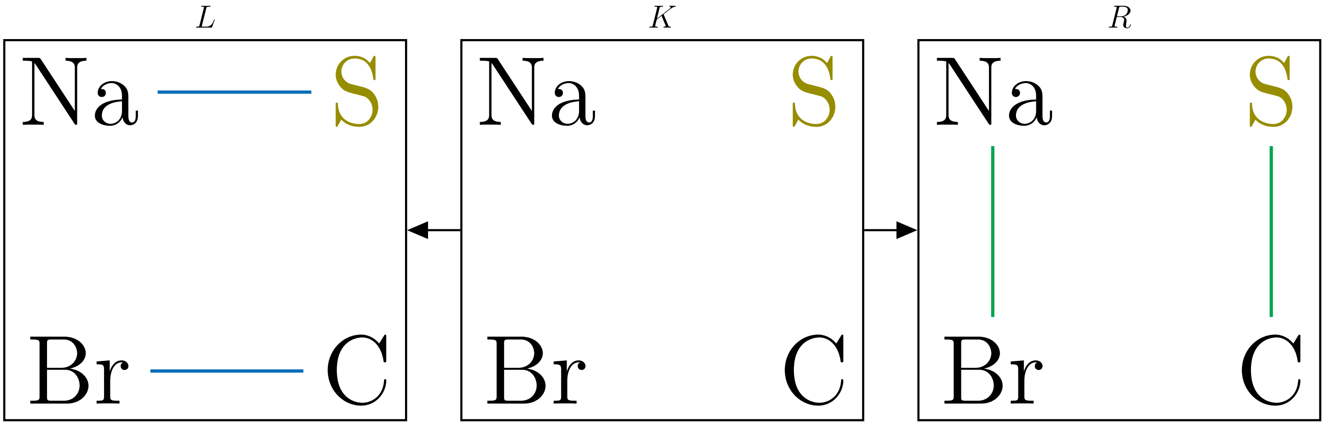

Files: out/343_r_68_11300100_{L, K, R}

0.0.70 69

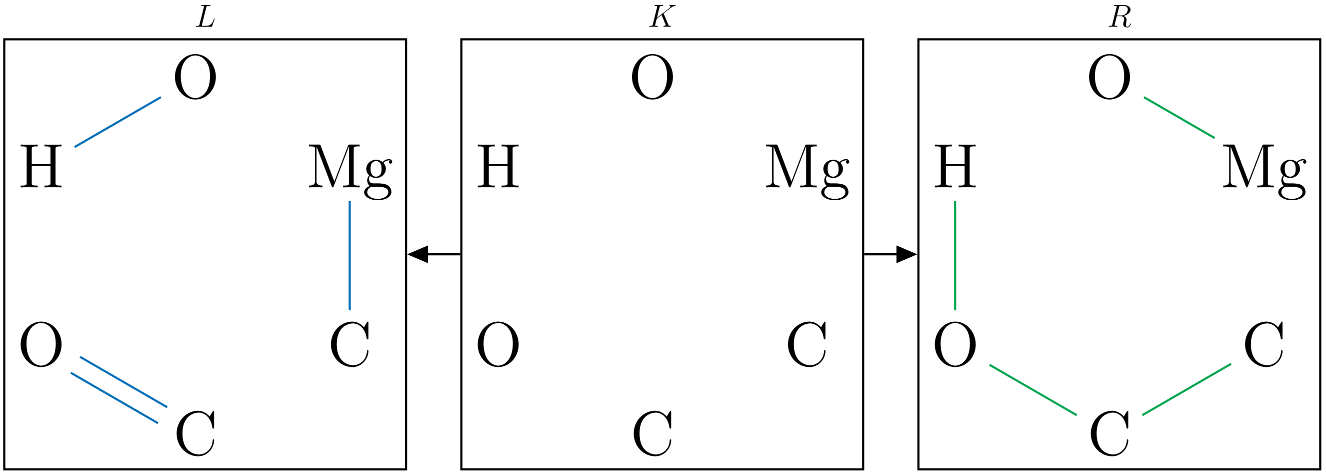

Files: out/346_r_69_10300000_{L, K, R}

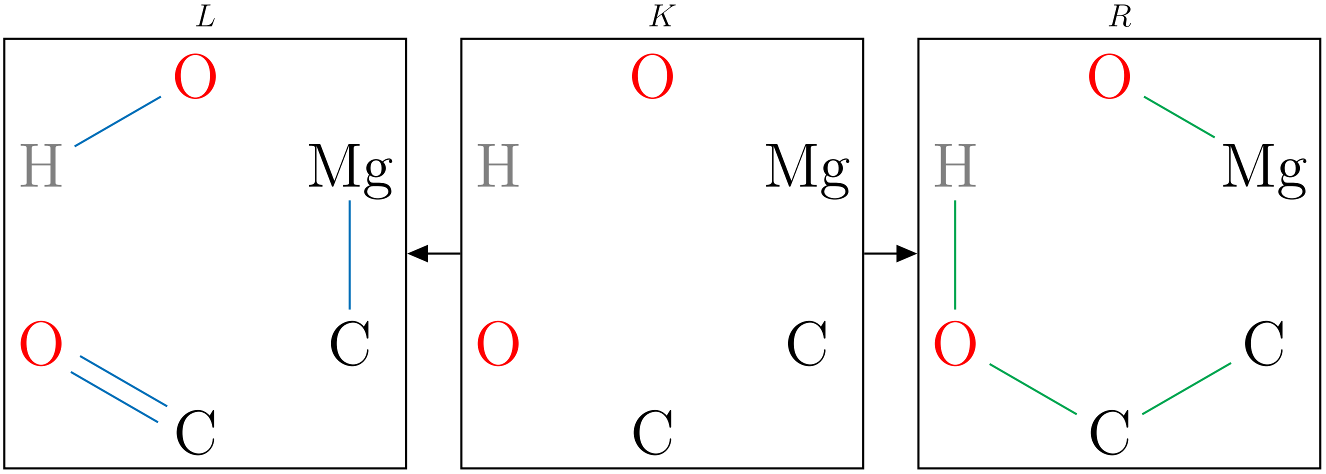

Files: out/348_r_69_11300100_{L, K, R}

0.0.71 70

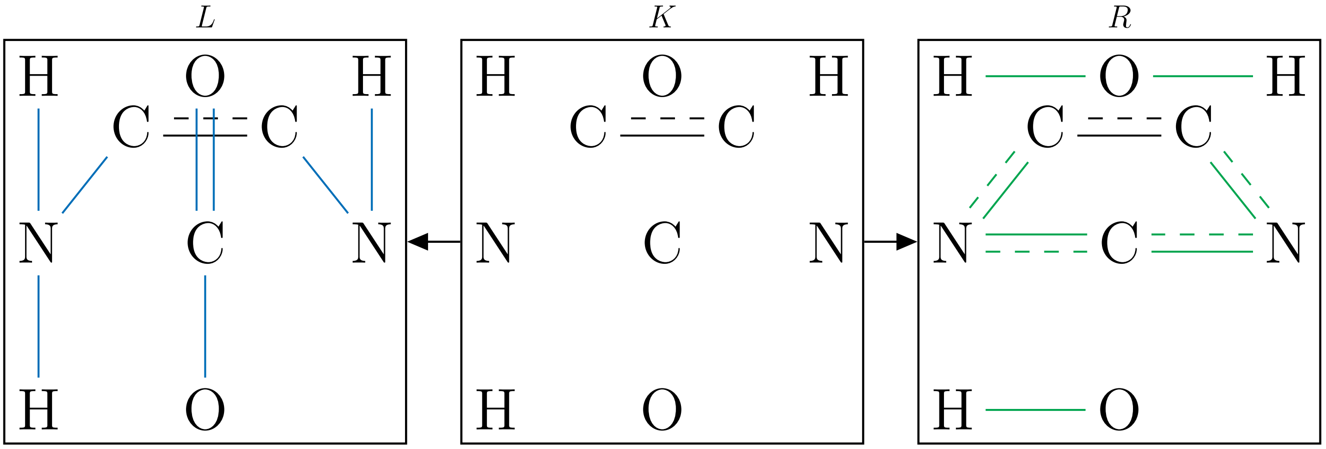

Files: out/351_r_70_10300000_{L, K, R}

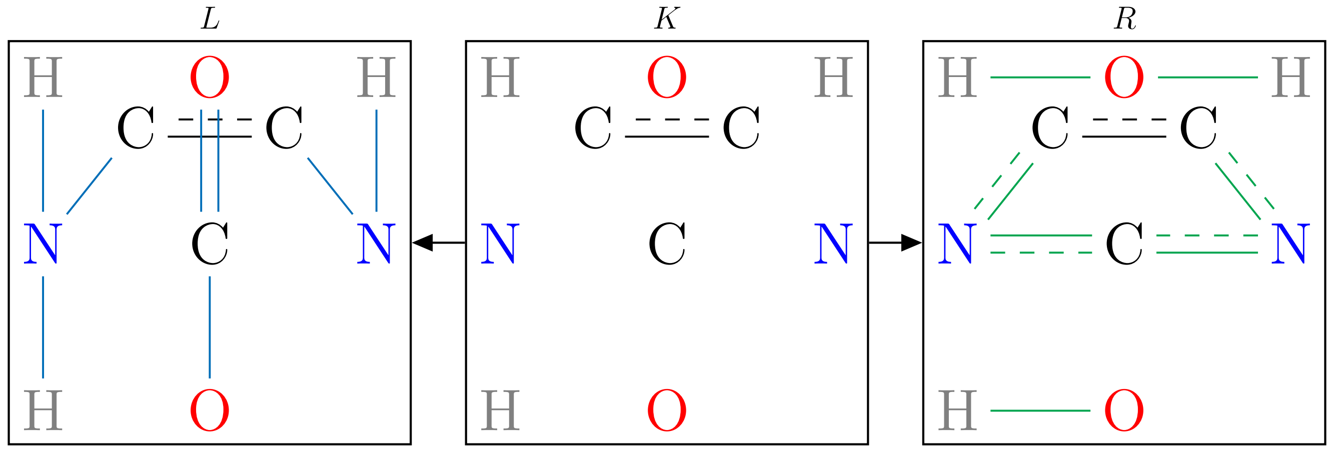

Files: out/353_r_70_11300100_{L, K, R}

0.0.72 71

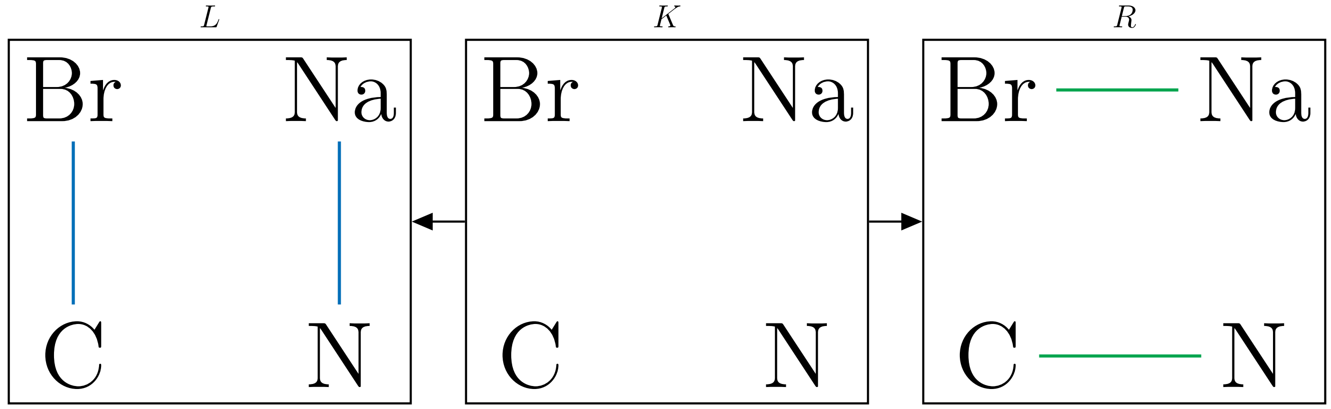

Files: out/356_r_71_10300000_{L, K, R}

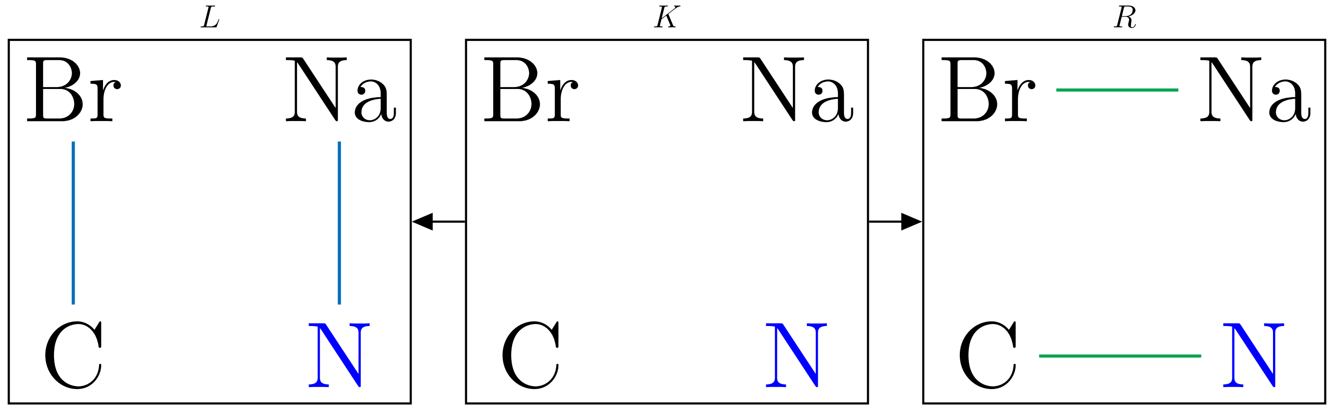

Files: out/358_r_71_11300100_{L, K, R}

0.0.73 72

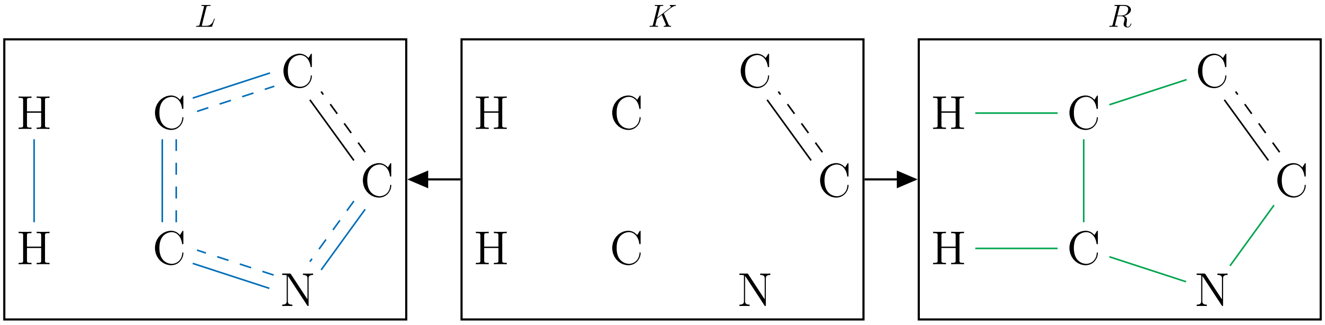

Files: out/361_r_72_10300000_{L, K, R}

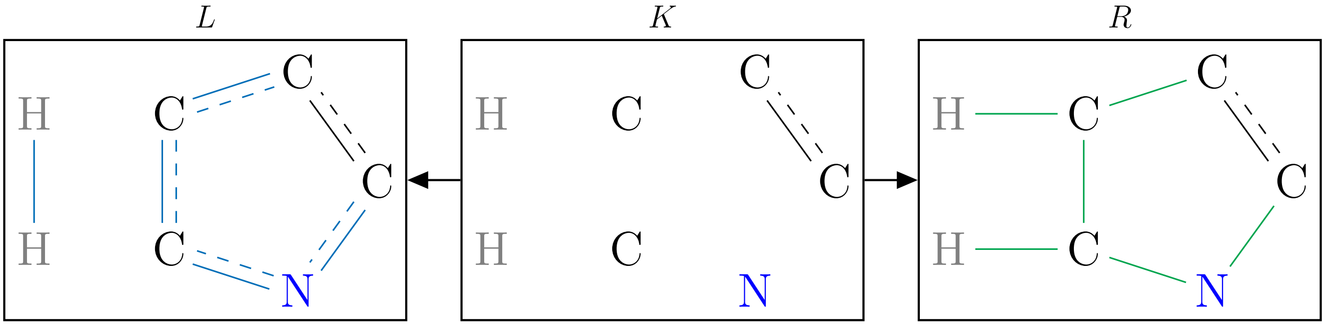

Files: out/363_r_72_11300100_{L, K, R}

0.0.74 73

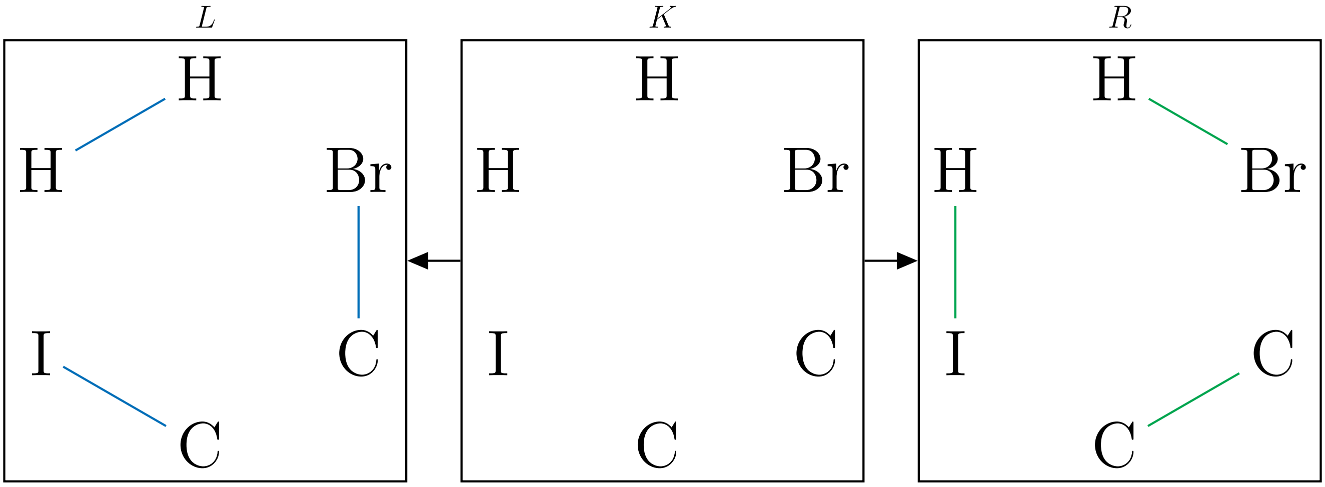

Files: out/366_r_73_10300000_{L, K, R}

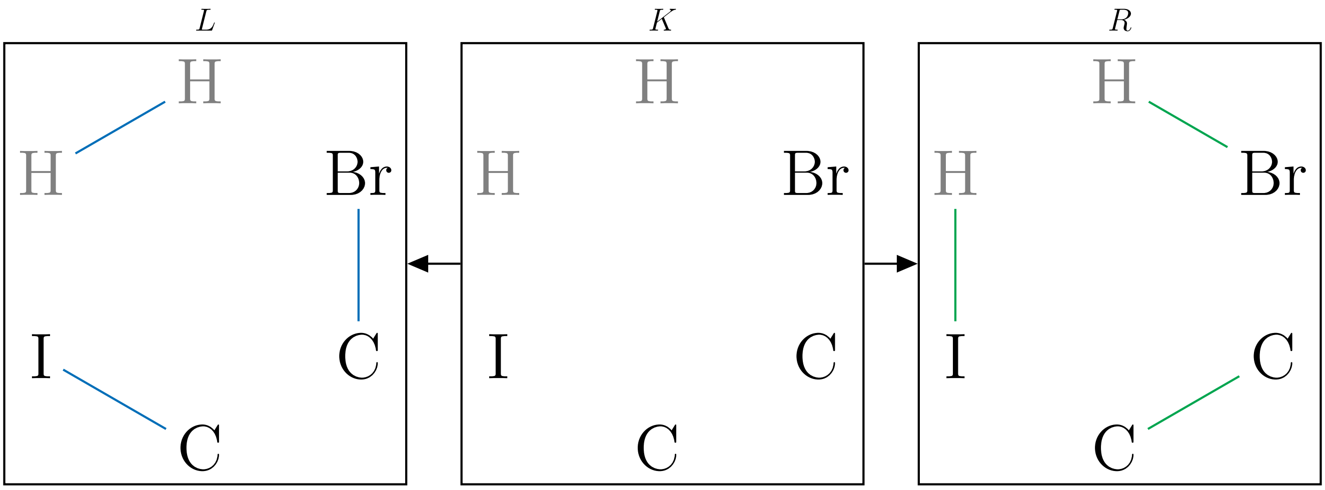

Files: out/368_r_73_11300100_{L, K, R}

0.0.75 74

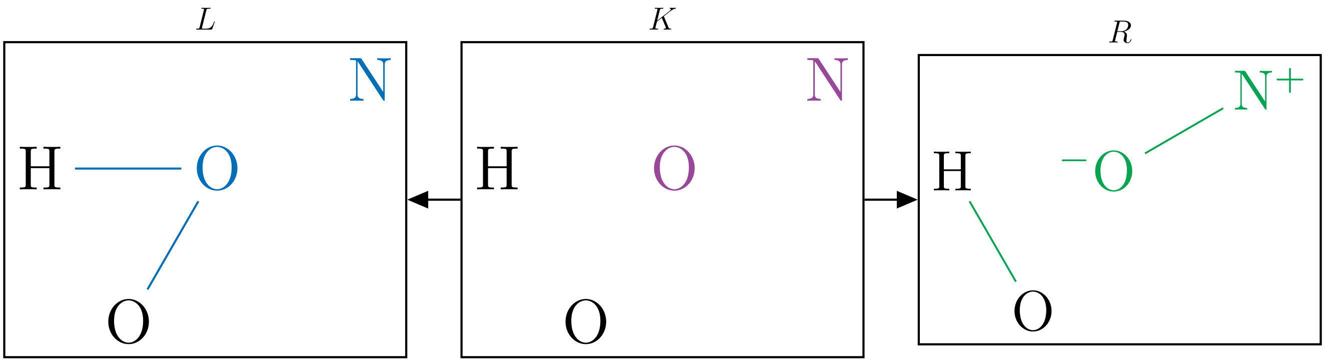

Files: out/371_r_74_10300000_{L, K, R}

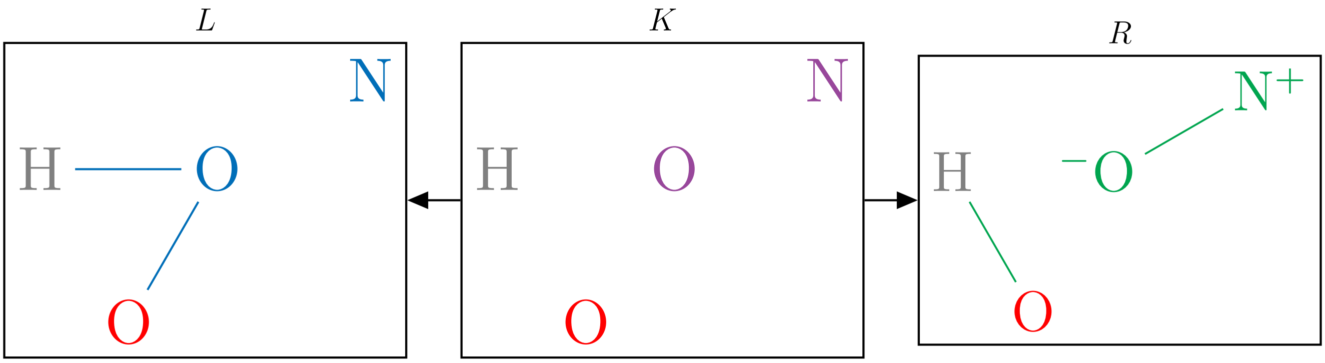

Files: out/373_r_74_11300100_{L, K, R}

0.0.76 75

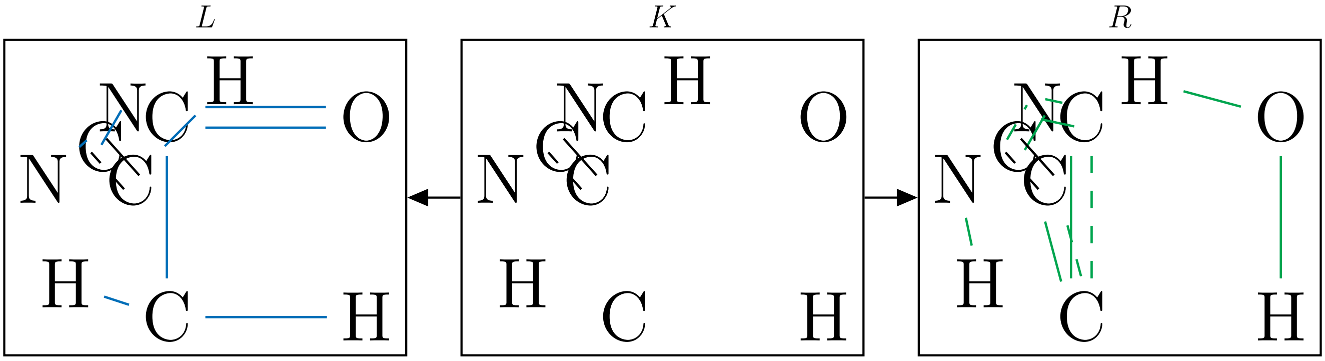

Files: out/376_r_75_10300000_{L, K, R}

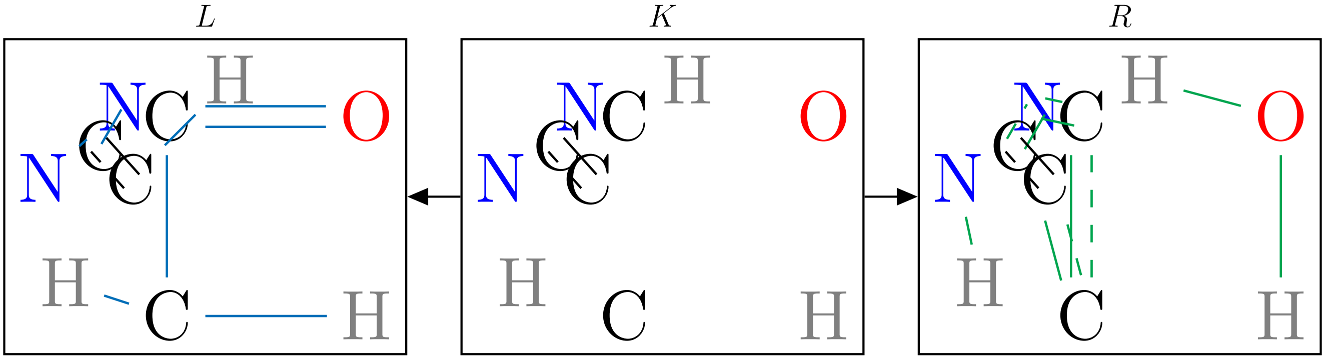

Files: out/378_r_75_11300100_{L, K, R}

0.0.77 76

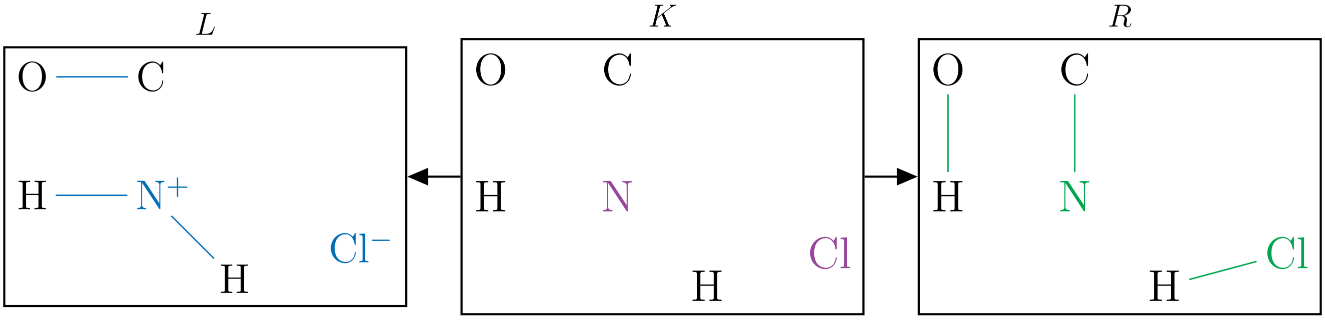

Files: out/381_r_76_10300000_{L, K, R}

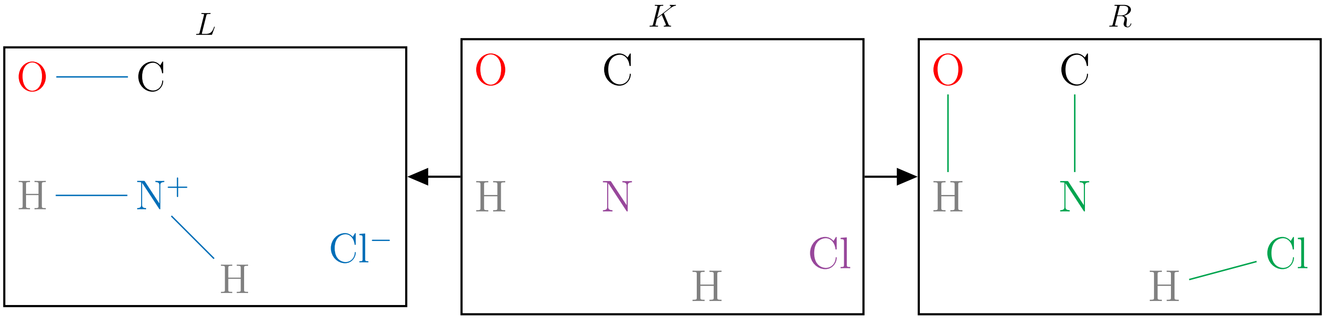

Files: out/383_r_76_11300100_{L, K, R}

0.0.78 77

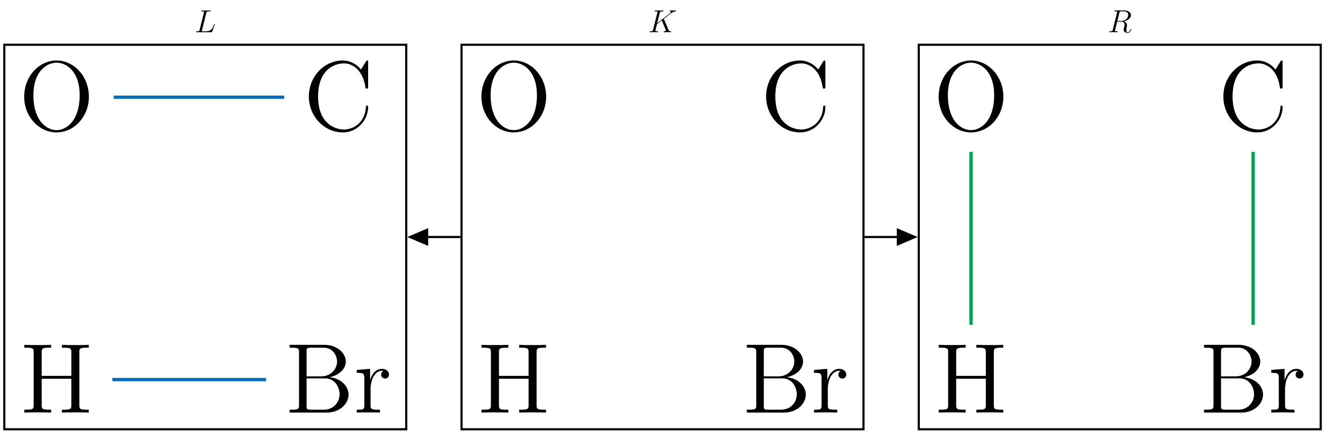

Files: out/386_r_77_10300000_{L, K, R}

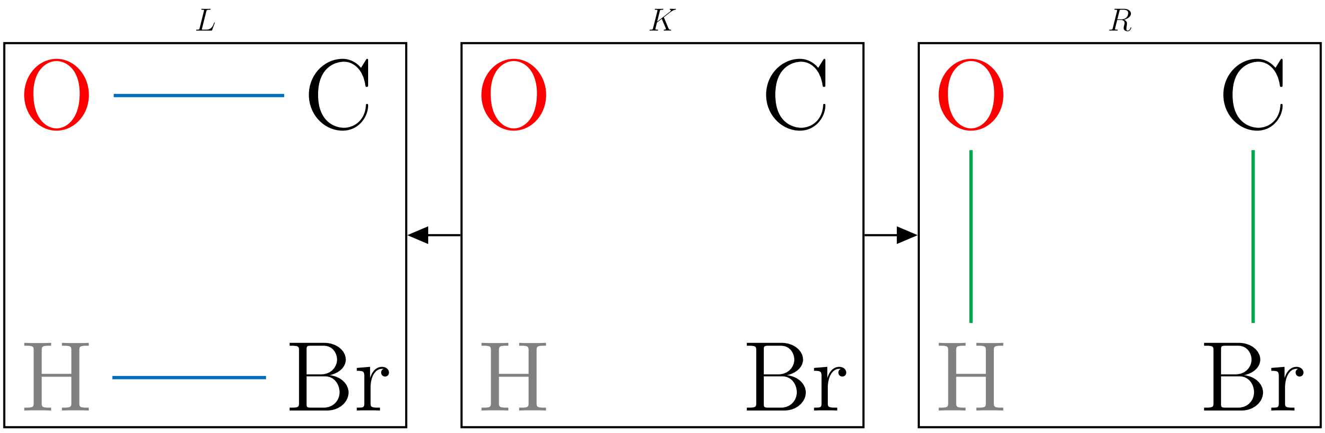

Files: out/388_r_77_11300100_{L, K, R}

0.0.79 78

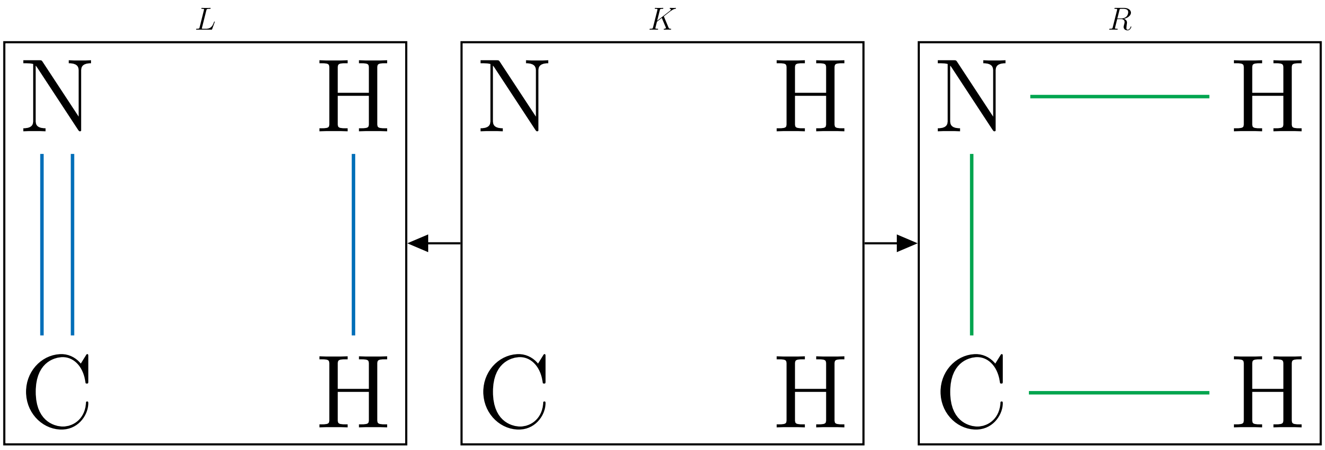

Files: out/391_r_78_10300000_{L, K, R}

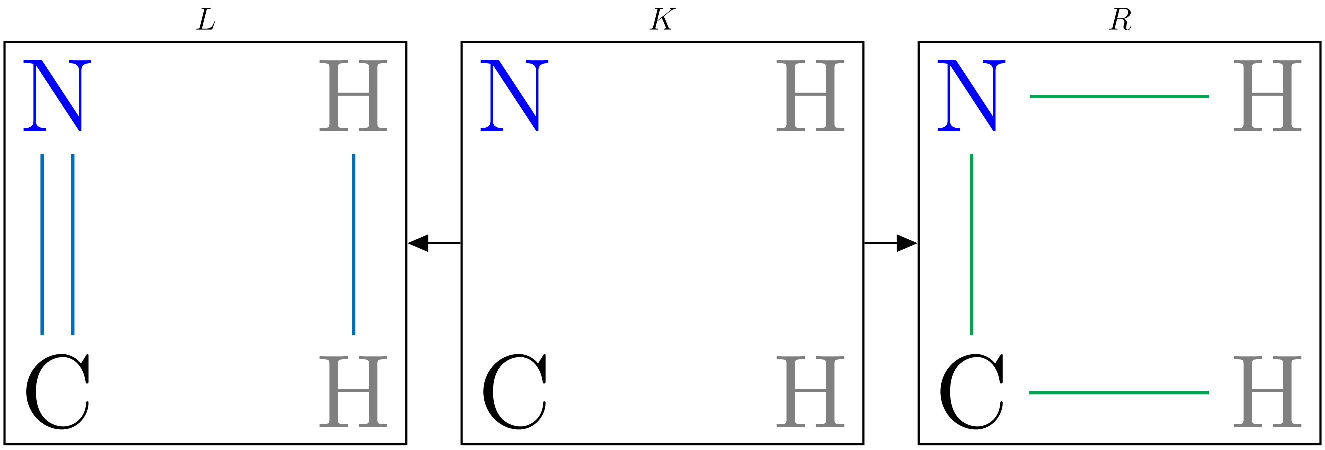

Files: out/393_r_78_11300100_{L, K, R}

0.0.80 79

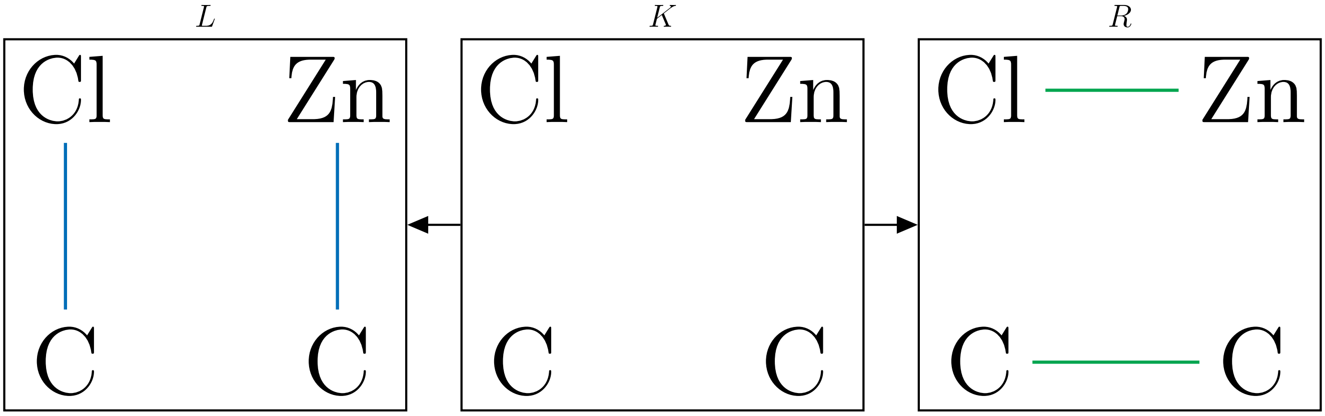

Files: out/396_r_79_10300000_{L, K, R}

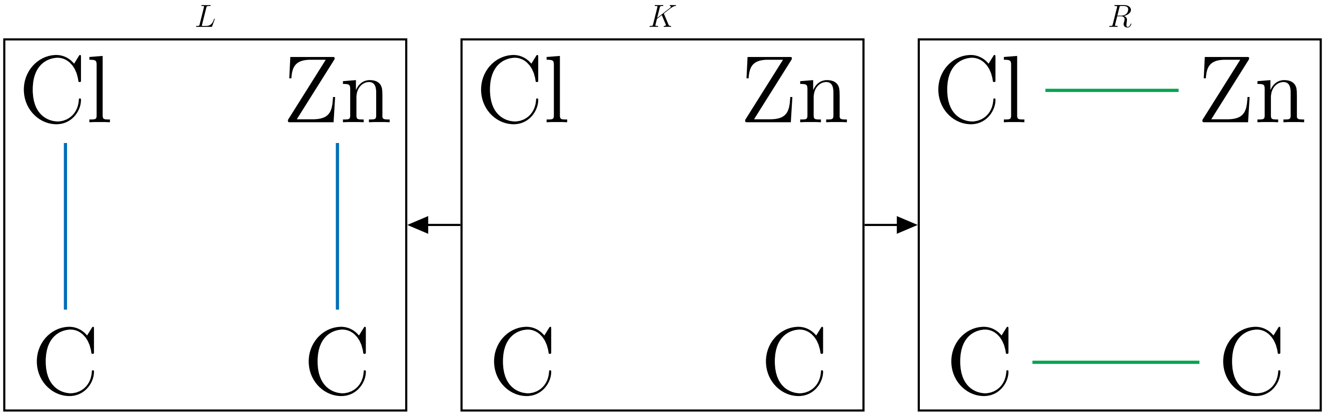

Files: out/398_r_79_11300100_{L, K, R}

0.0.81 80

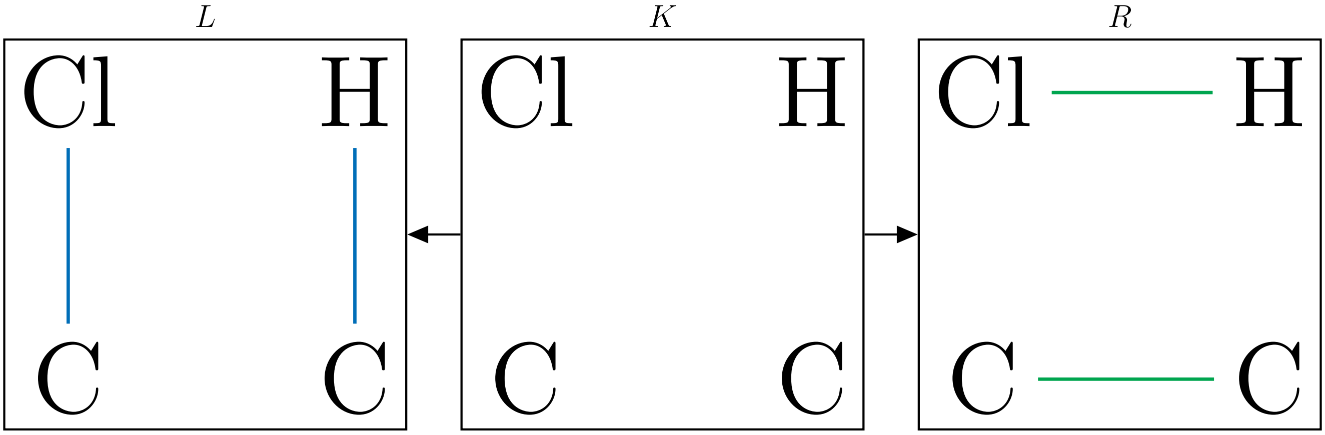

Files: out/401_r_80_10300000_{L, K, R}

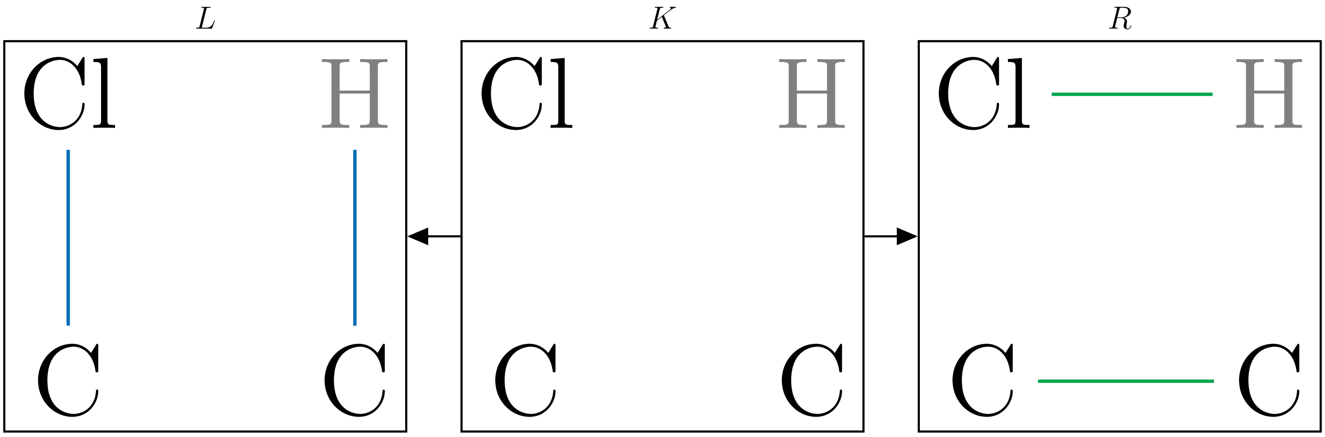

Files: out/403_r_80_11300100_{L, K, R}

0.0.82 81

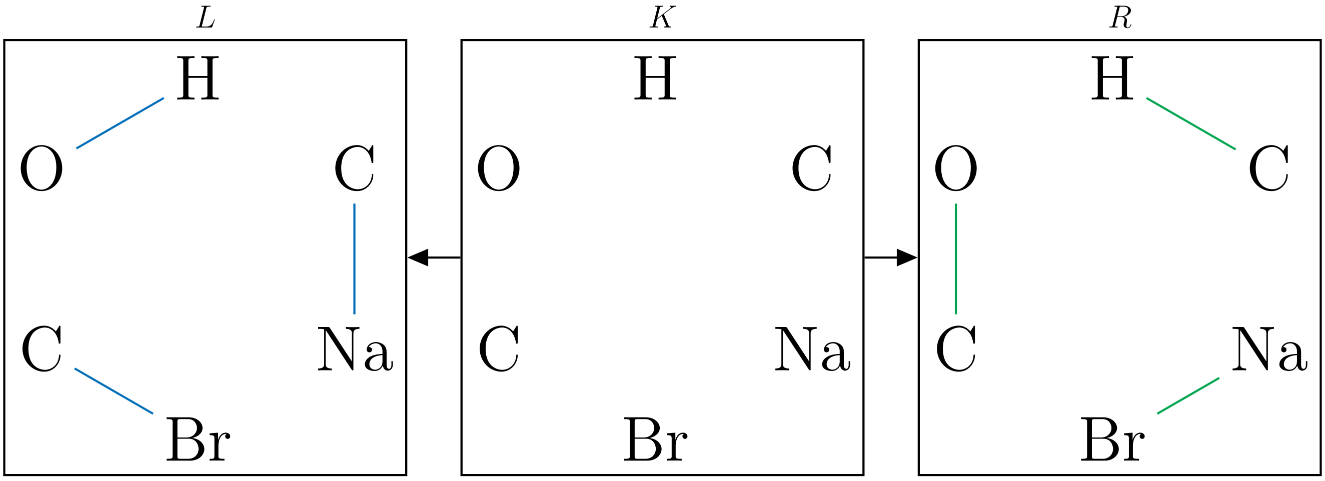

Files: out/406_r_81_10300000_{L, K, R}

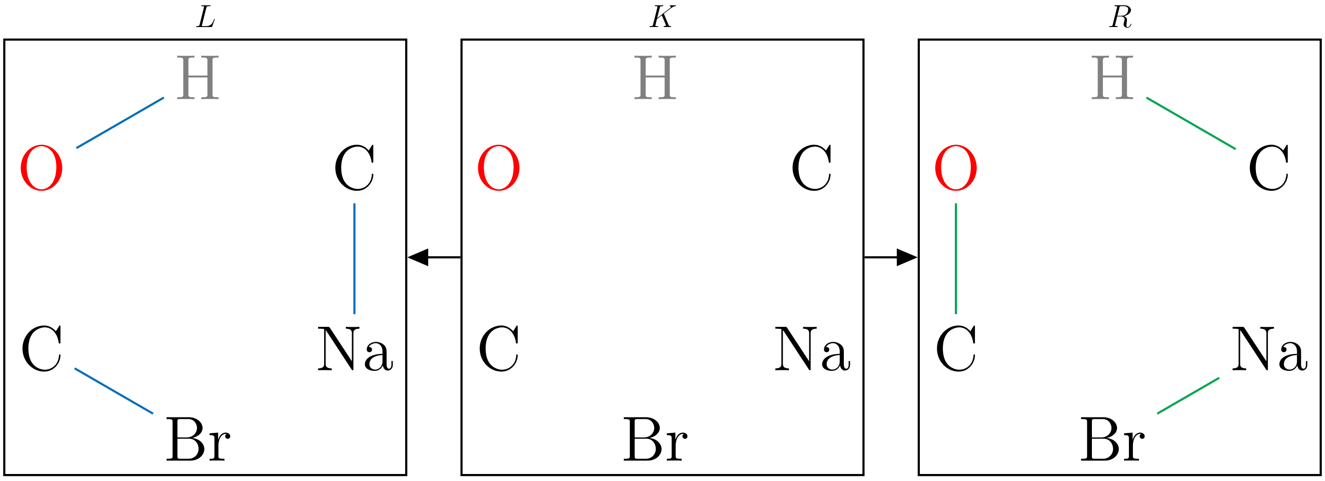

Files: out/408_r_81_11300100_{L, K, R}

0.0.83 82

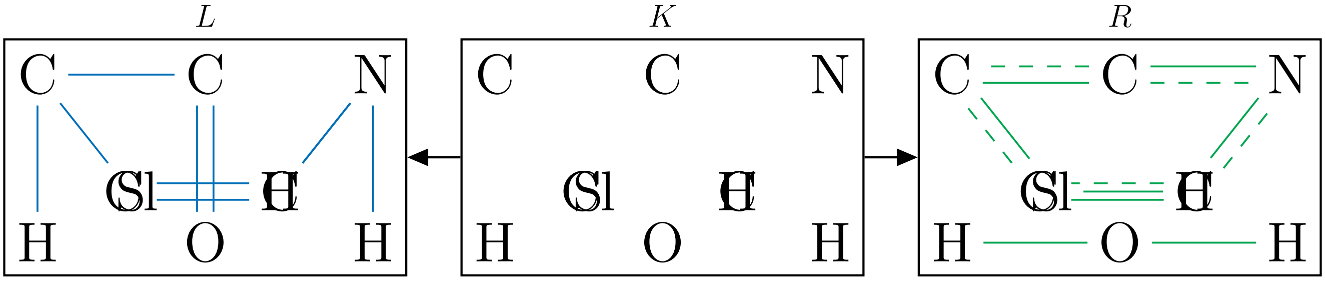

Files: out/411_r_82_10300000_{L, K, R}

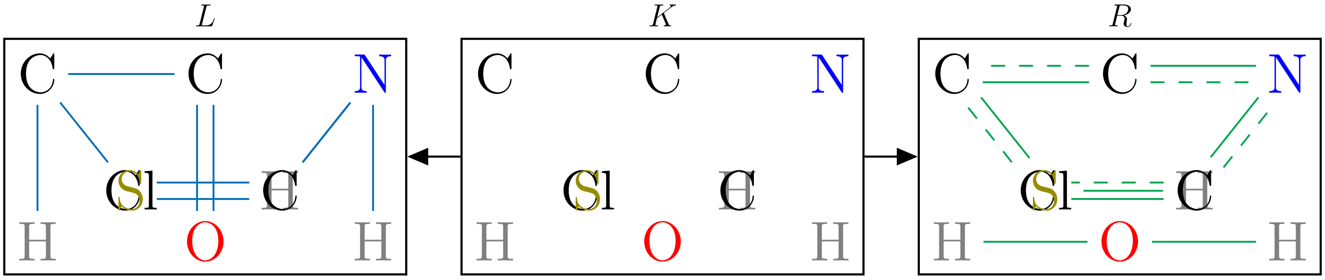

Files: out/413_r_82_11300100_{L, K, R}

0.0.84 83

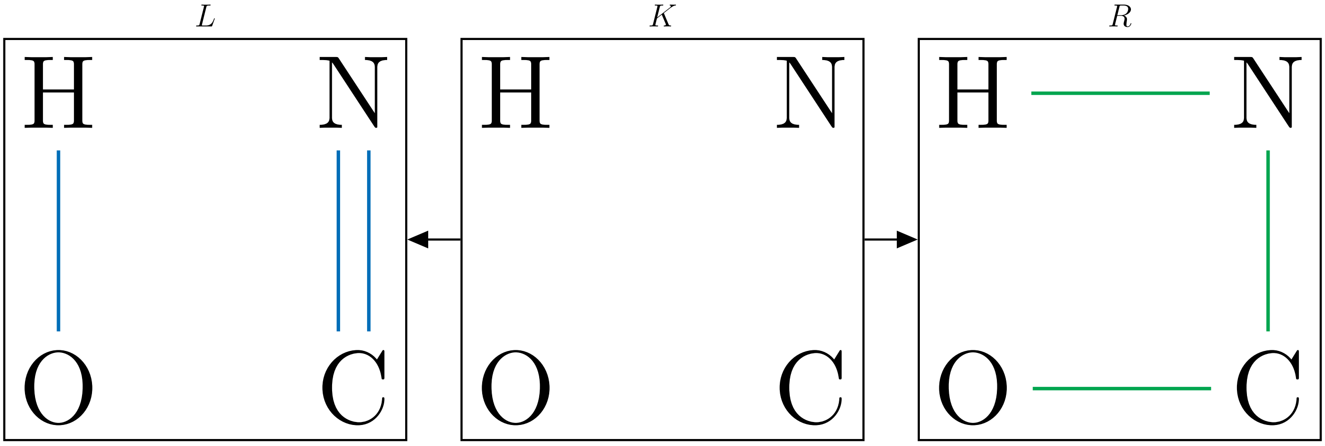

Files: out/416_r_83_10300000_{L, K, R}

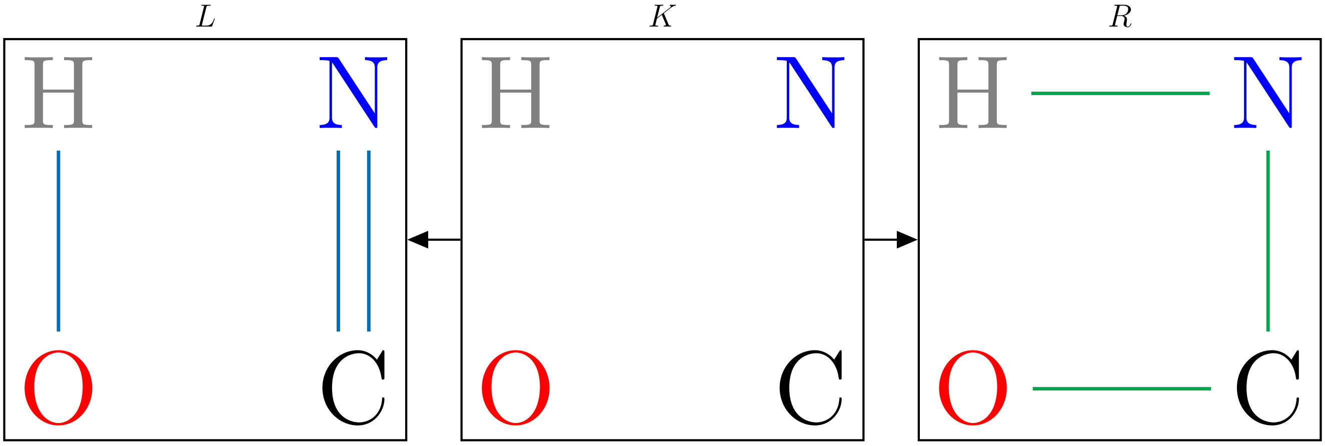

Files: out/418_r_83_11300100_{L, K, R}

0.0.85 84

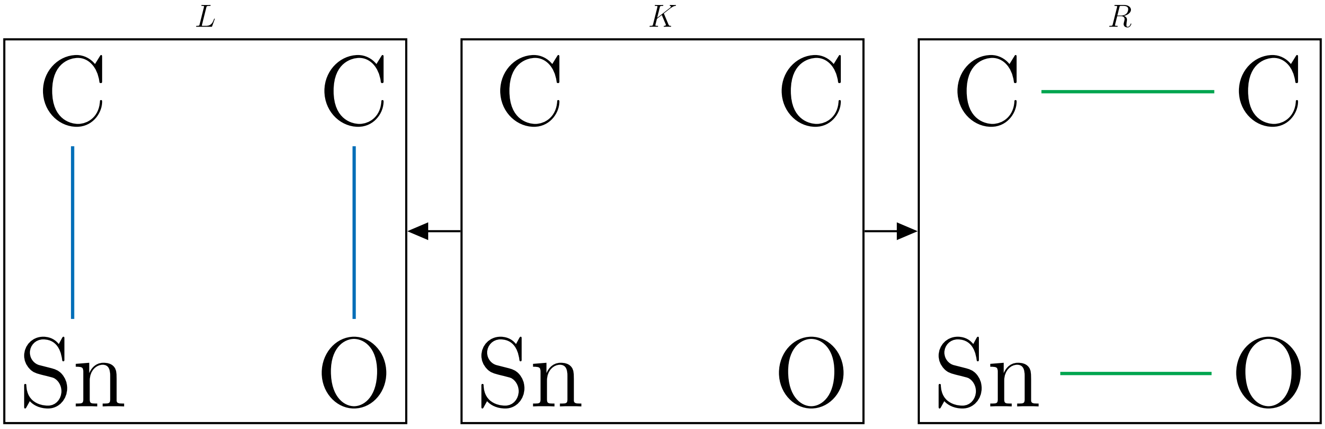

Files: out/421_r_84_10300000_{L, K, R}

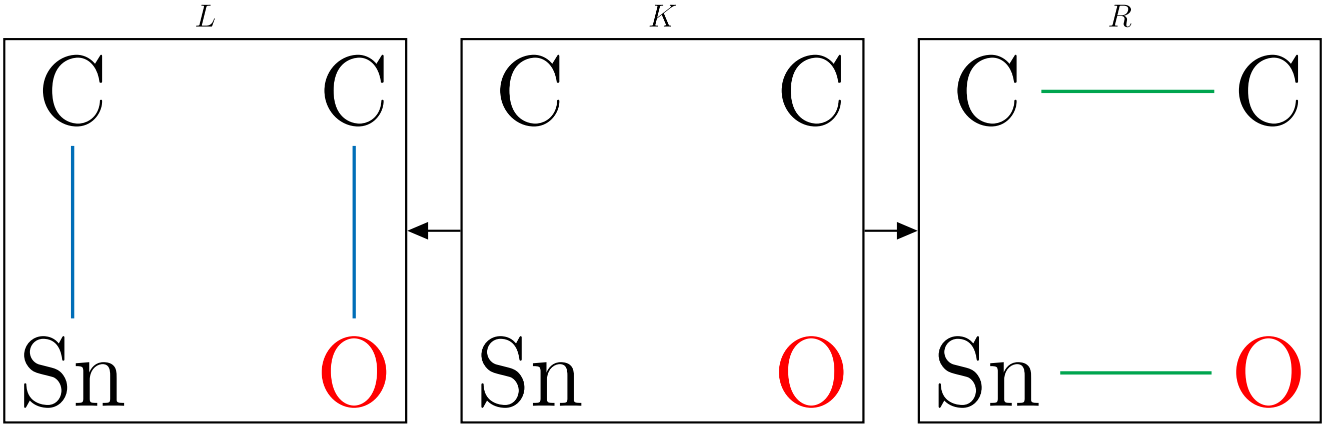

Files: out/423_r_84_11300100_{L, K, R}

0.0.86 85

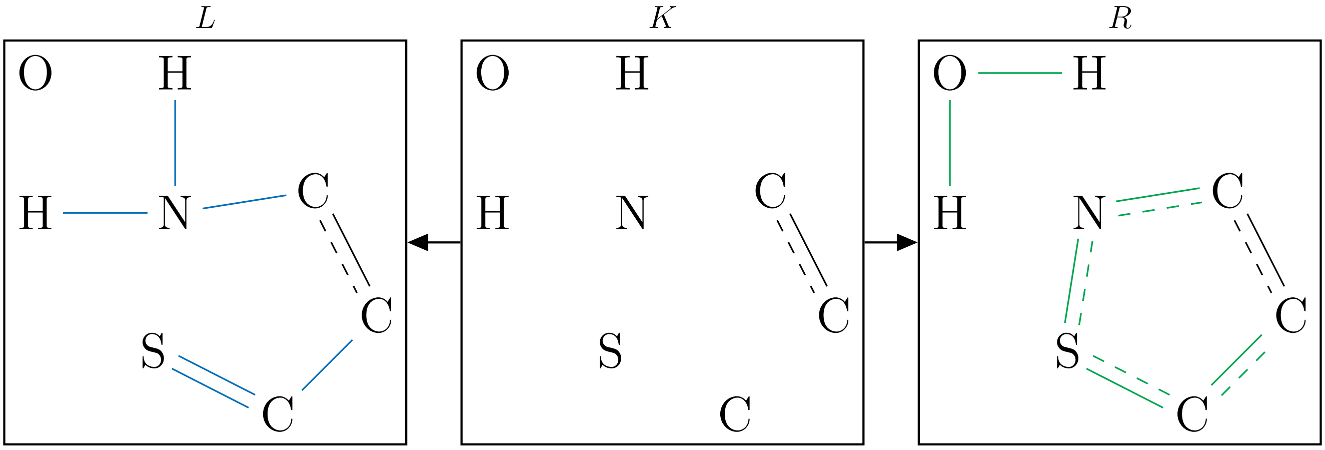

Files: out/426_r_85_10300000_{L, K, R}

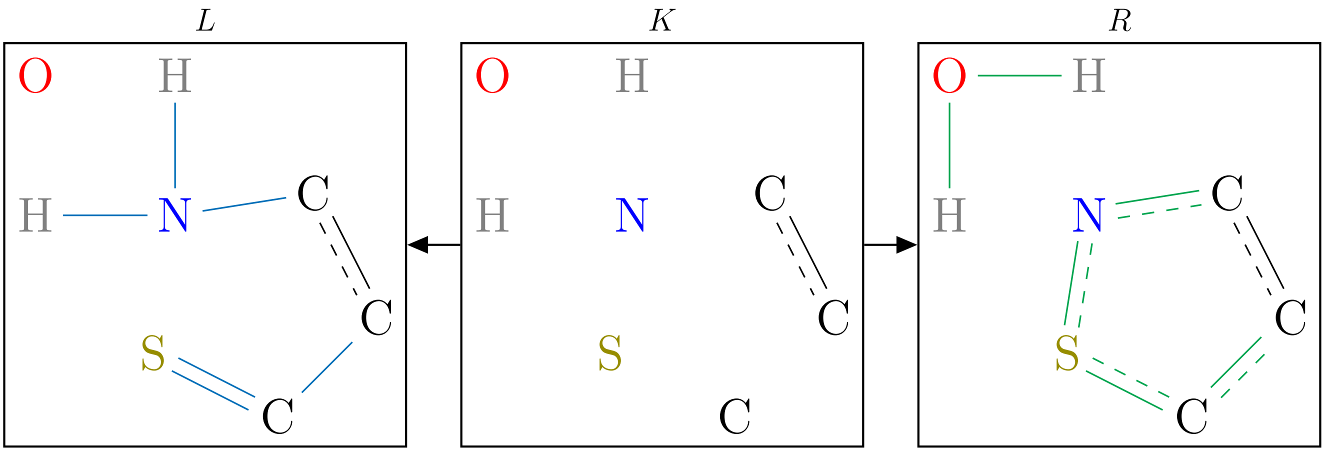

Files: out/428_r_85_11300100_{L, K, R}

0.0.87 86

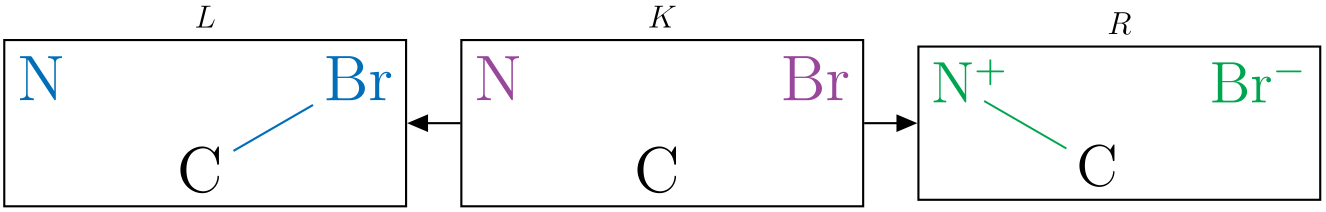

Files: out/431_r_86_10300000_{L, K, R}

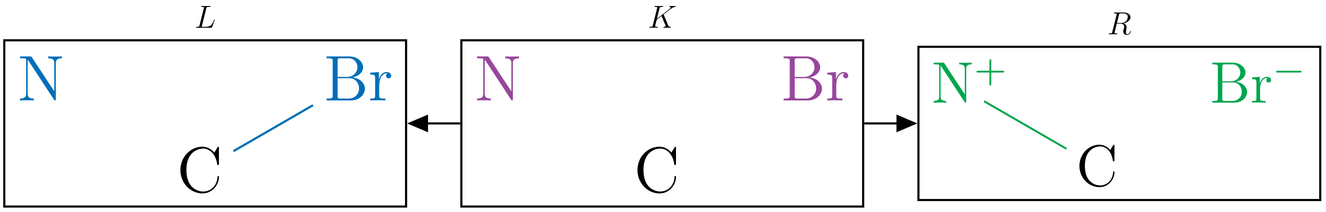

Files: out/433_r_86_11300100_{L, K, R}

0.0.88 87

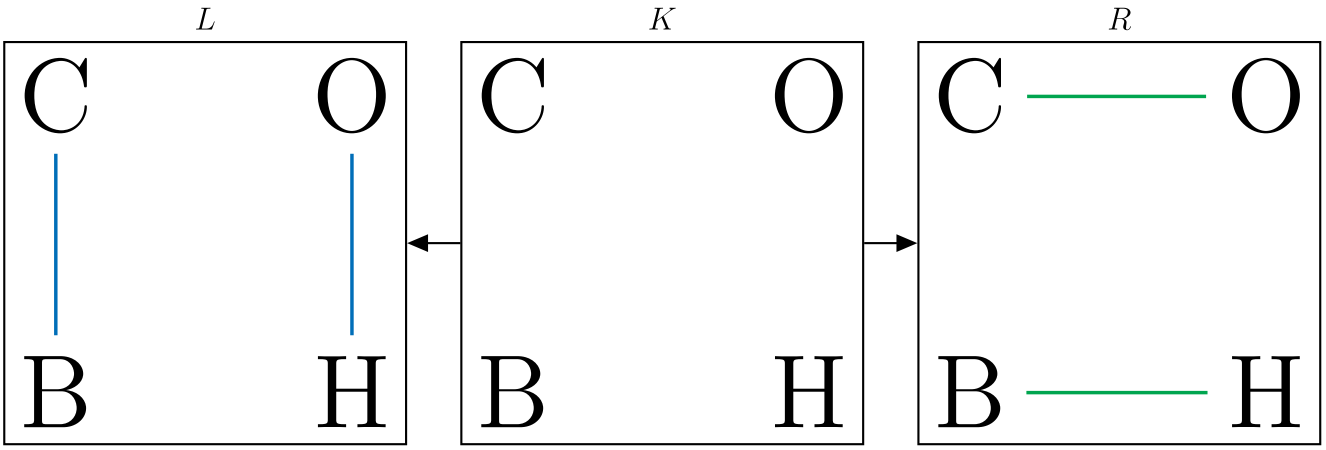

Files: out/436_r_87_10300000_{L, K, R}

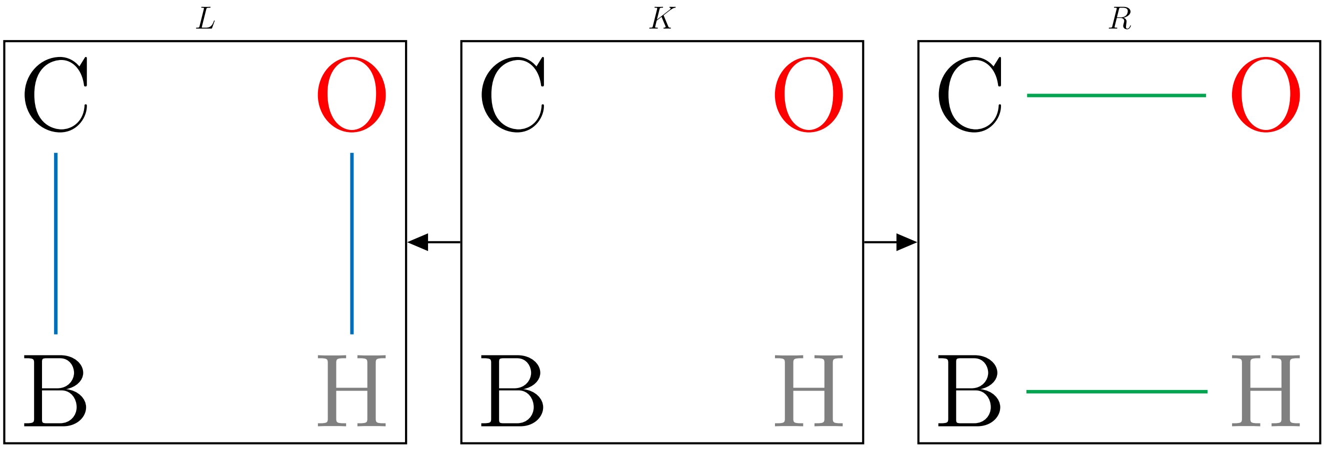

Files: out/438_r_87_11300100_{L, K, R}

0.0.89 88

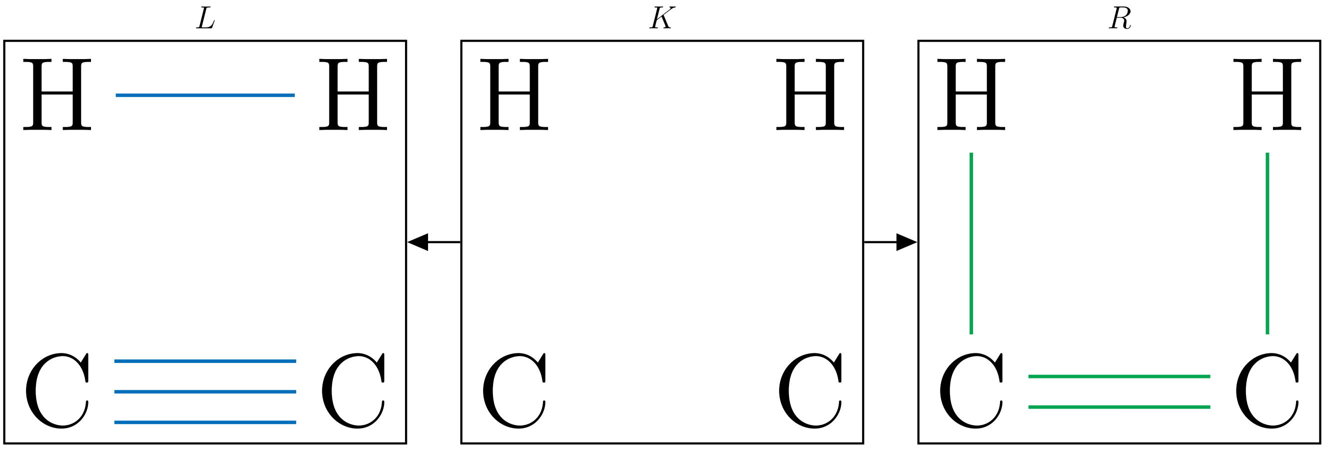

Files: out/441_r_88_10300000_{L, K, R}

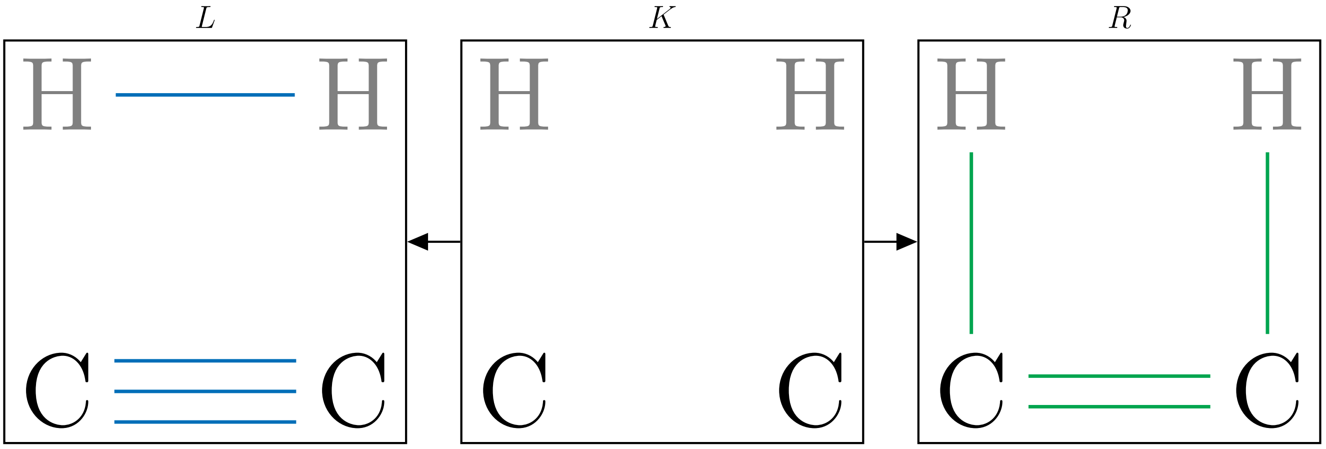

Files: out/443_r_88_11300100_{L, K, R}

0.0.90 89

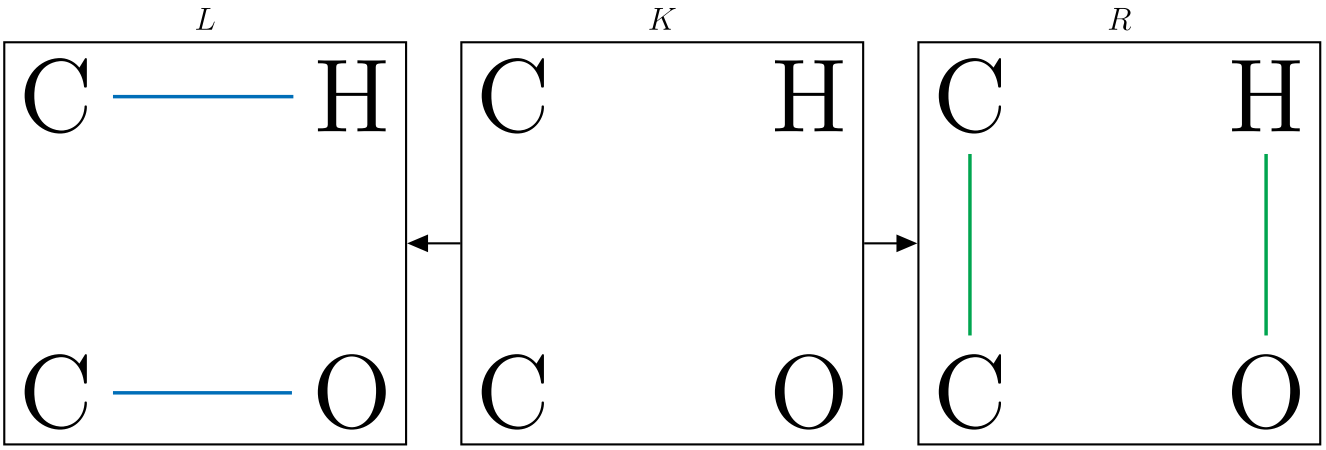

Files: out/446_r_89_10300000_{L, K, R}

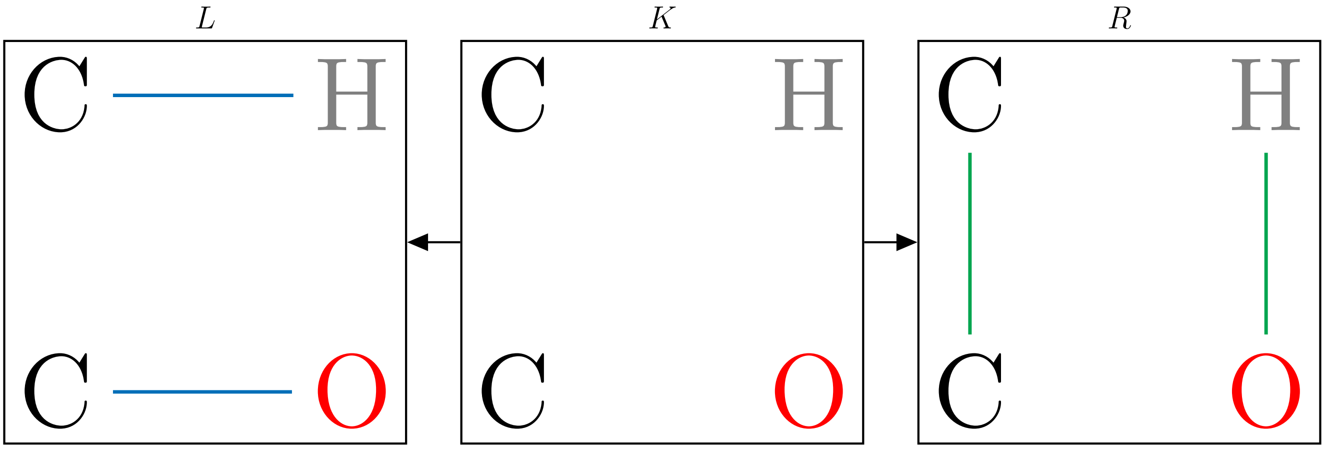

Files: out/448_r_89_11300100_{L, K, R}

0.0.91 90

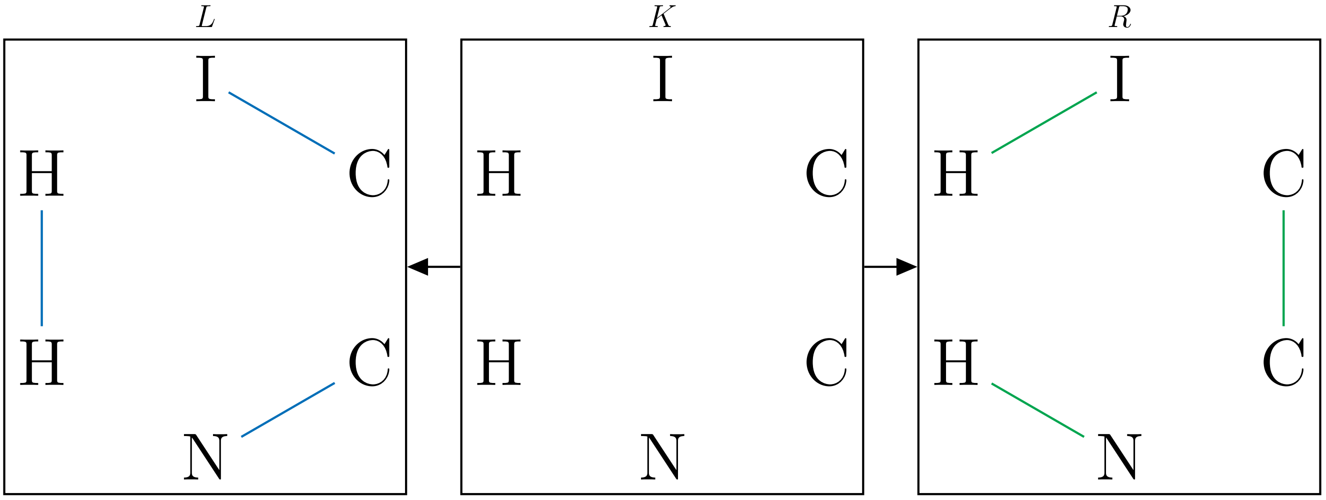

Files: out/451_r_90_10300000_{L, K, R}

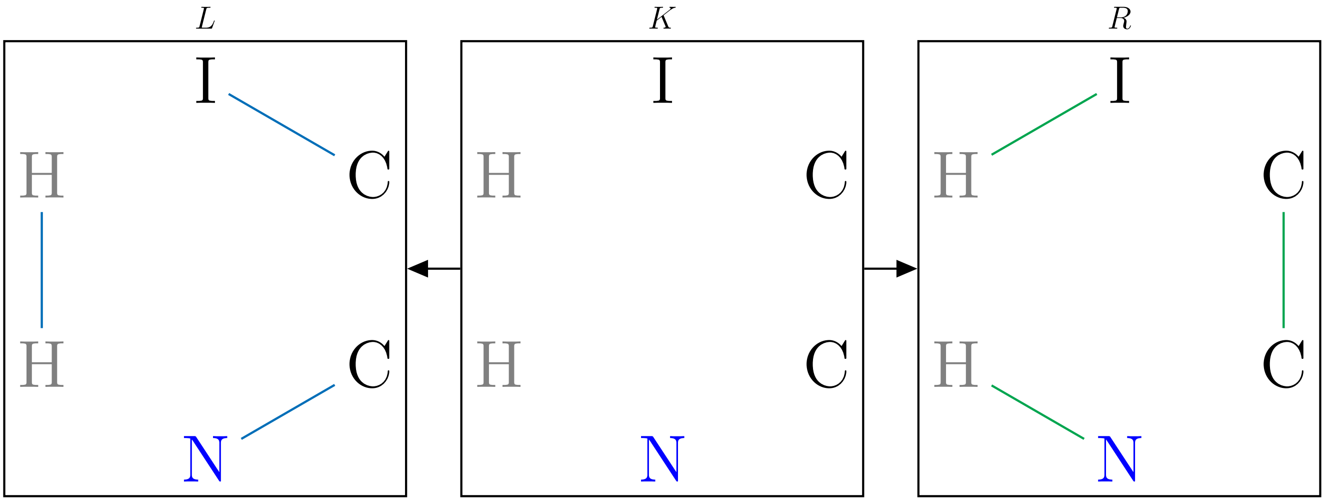

Files: out/453_r_90_11300100_{L, K, R}

0.0.92 91

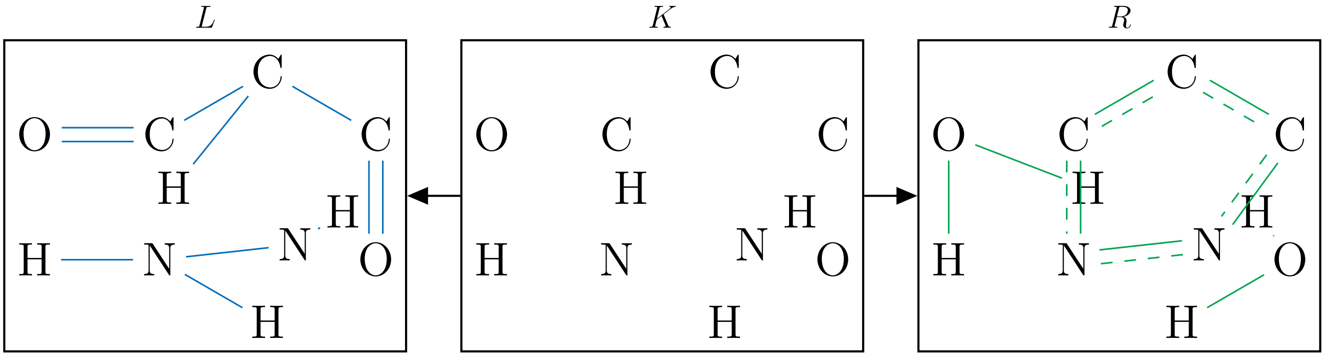

Files: out/456_r_91_10300000_{L, K, R}

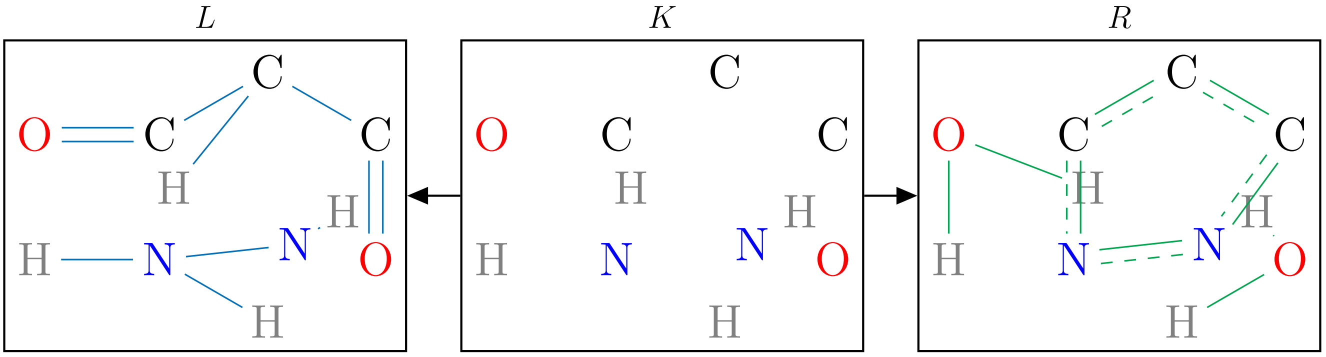

Files: out/458_r_91_11300100_{L, K, R}

0.0.93 92

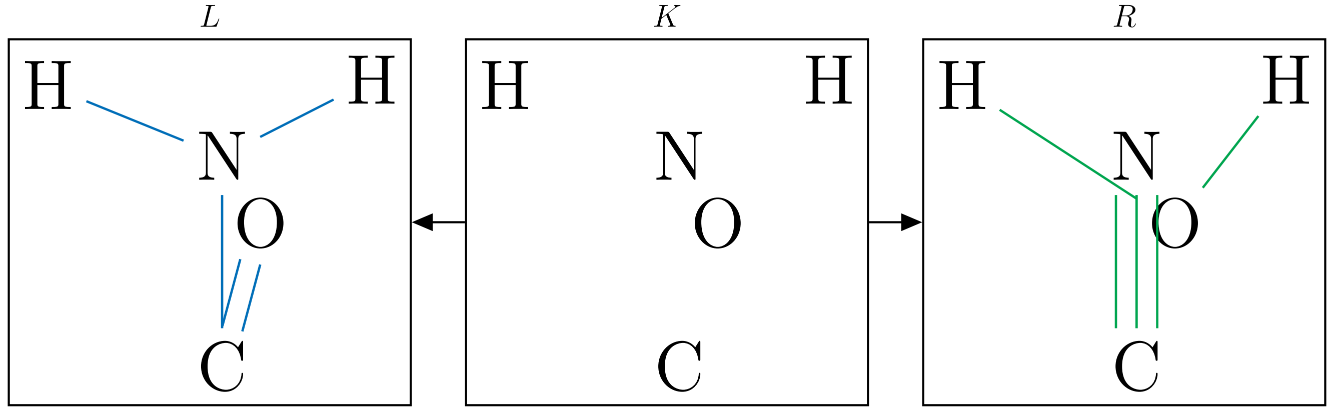

Files: out/461_r_92_10300000_{L, K, R}

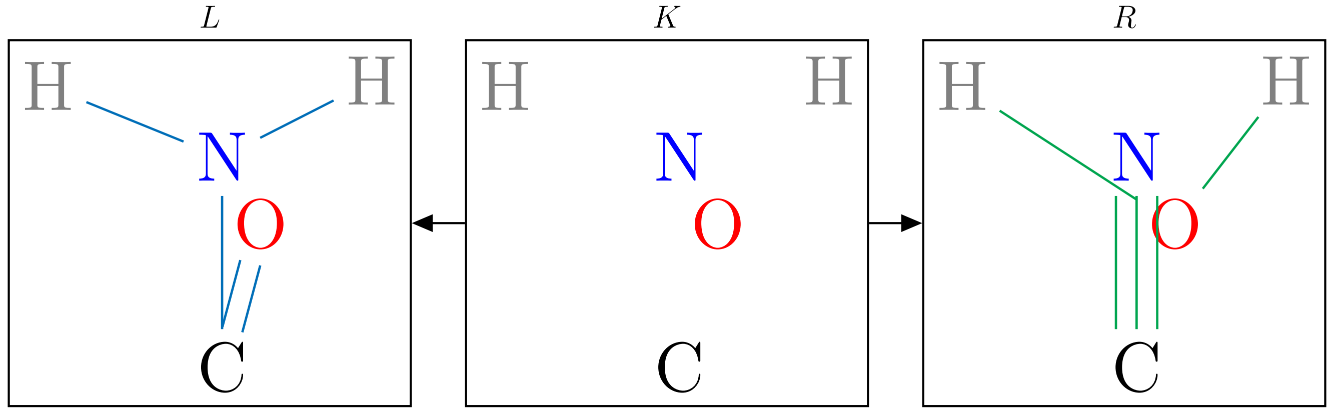

Files: out/463_r_92_11300100_{L, K, R}

0.0.94 93

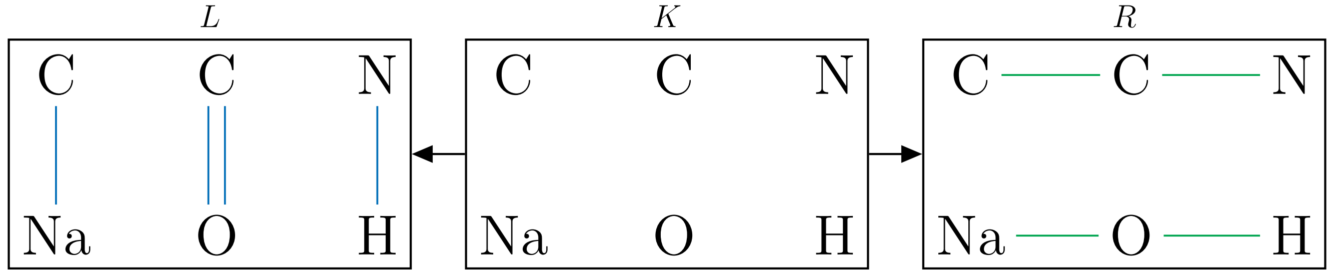

Files: out/466_r_93_10300000_{L, K, R}

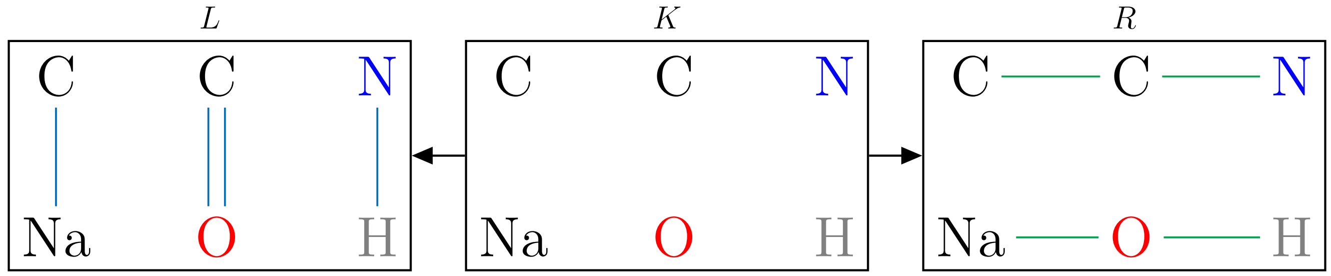

Files: out/468_r_93_11300100_{L, K, R}

0.0.95 94

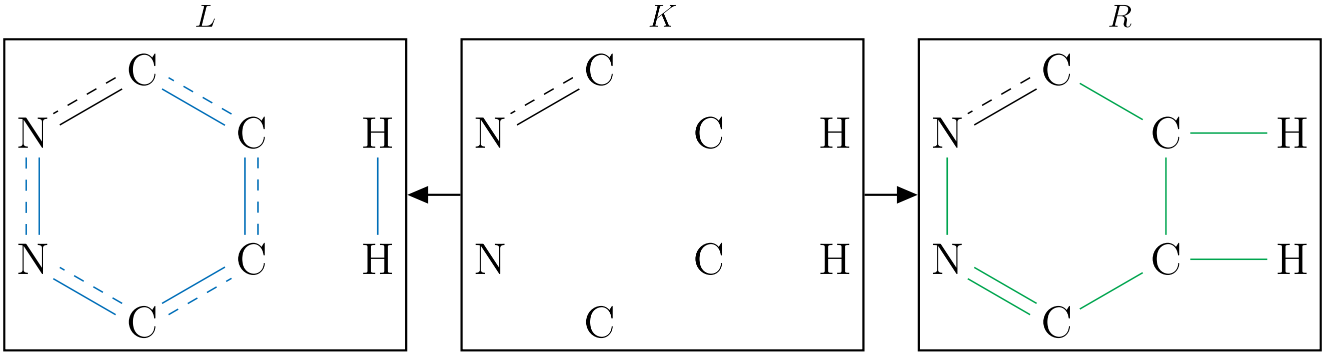

Files: out/471_r_94_10300000_{L, K, R}

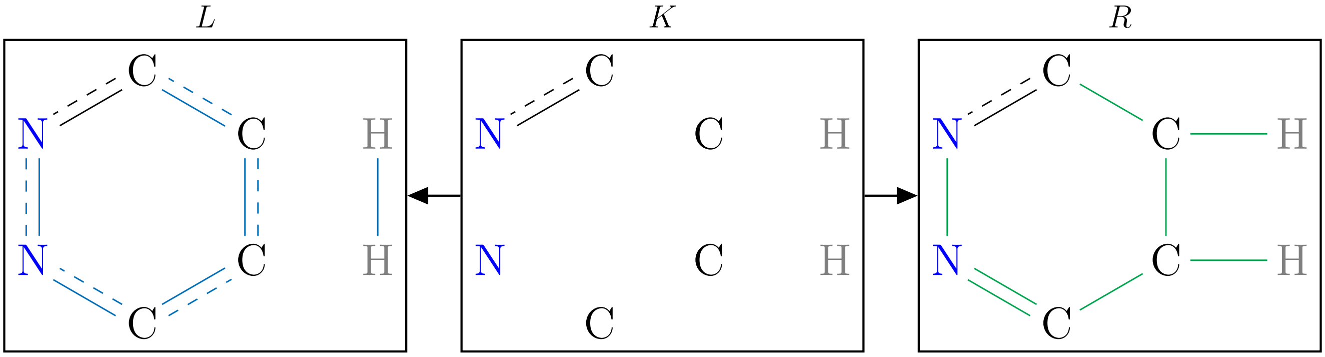

Files: out/473_r_94_11300100_{L, K, R}

0.0.96 95

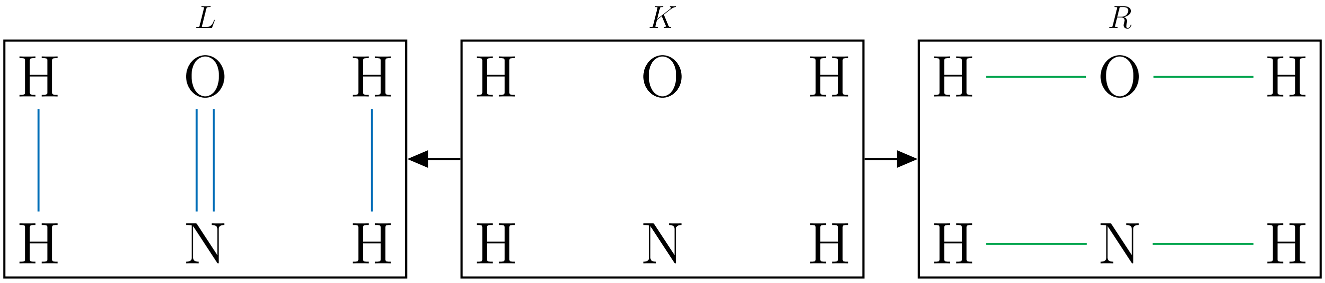

Files: out/476_r_95_10300000_{L, K, R}

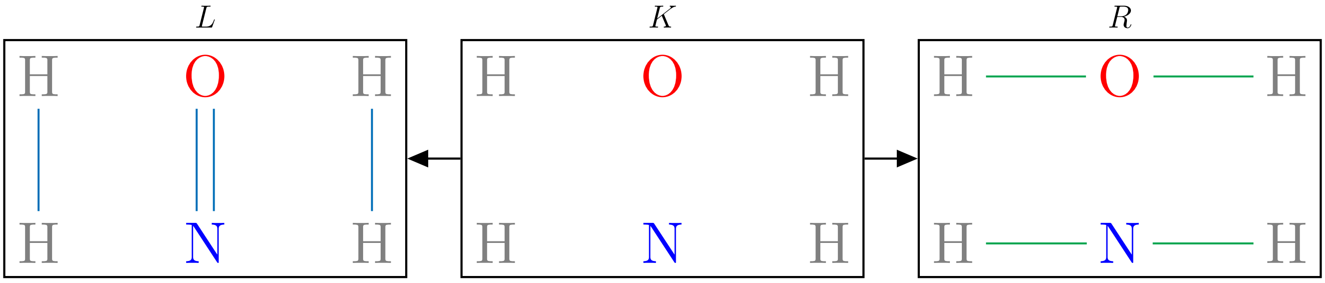

Files: out/478_r_95_11300100_{L, K, R}

0.0.97 96

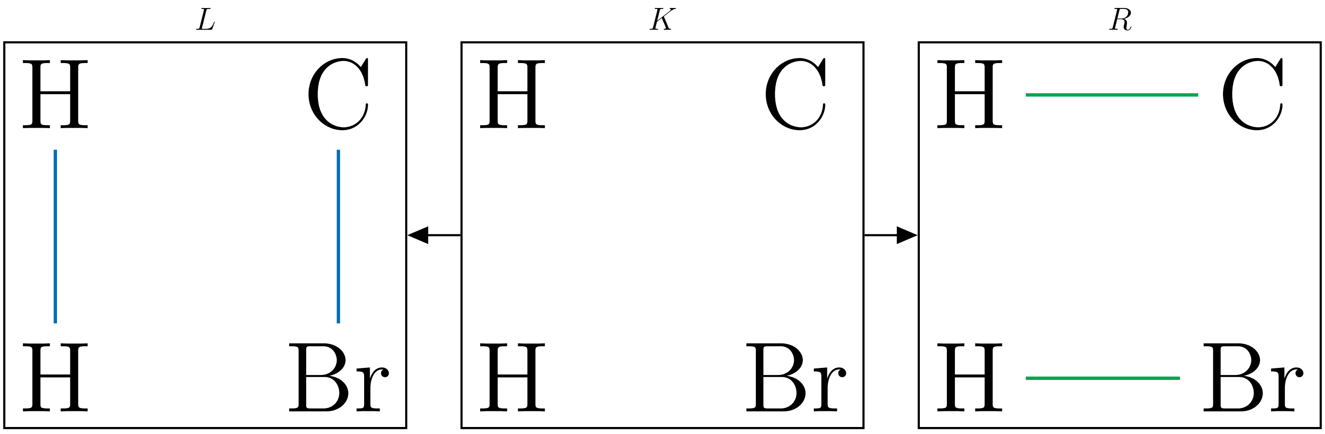

Files: out/481_r_96_10300000_{L, K, R}

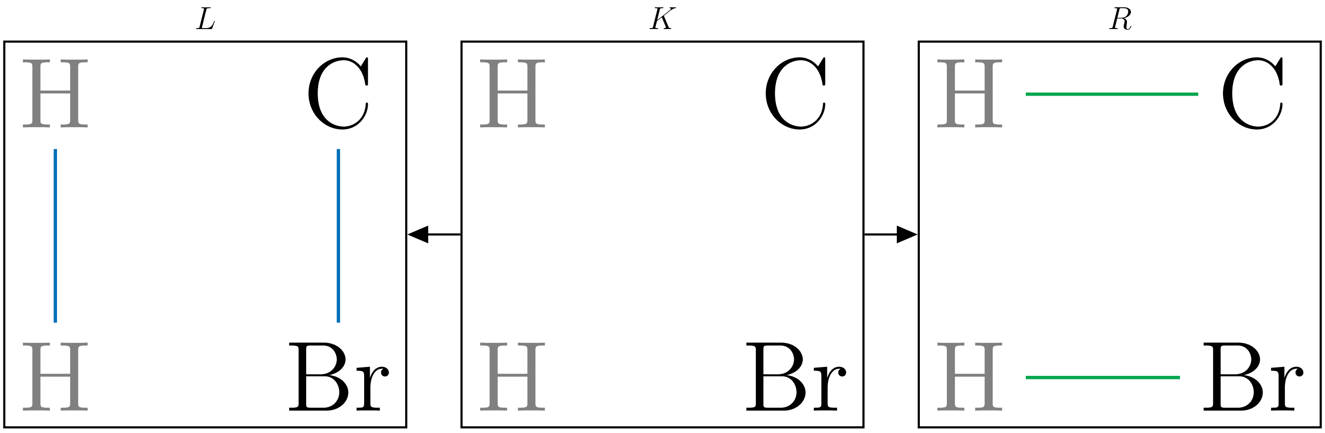

Files: out/483_r_96_11300100_{L, K, R}

0.0.98 97

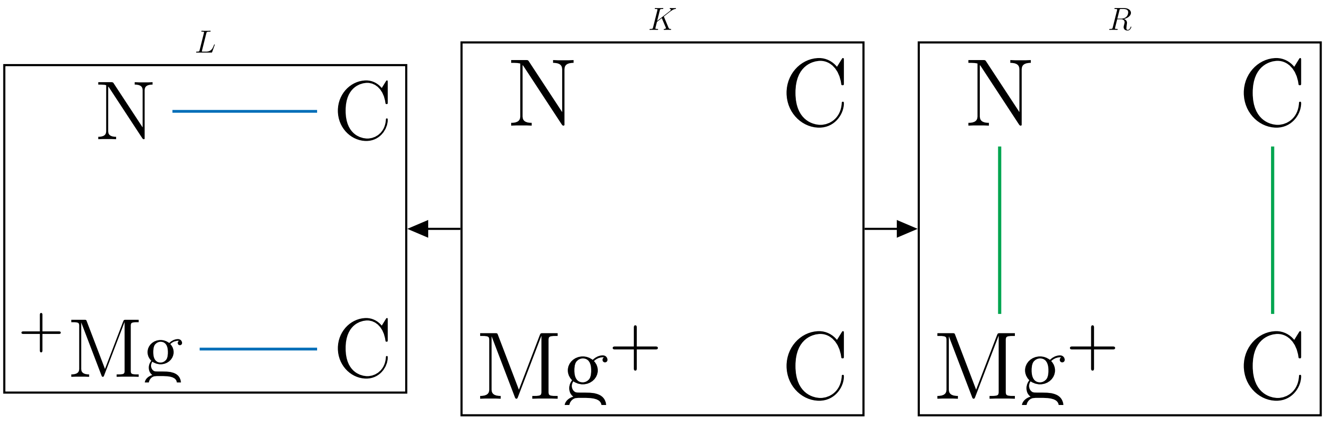

Files: out/486_r_97_10300000_{L, K, R}

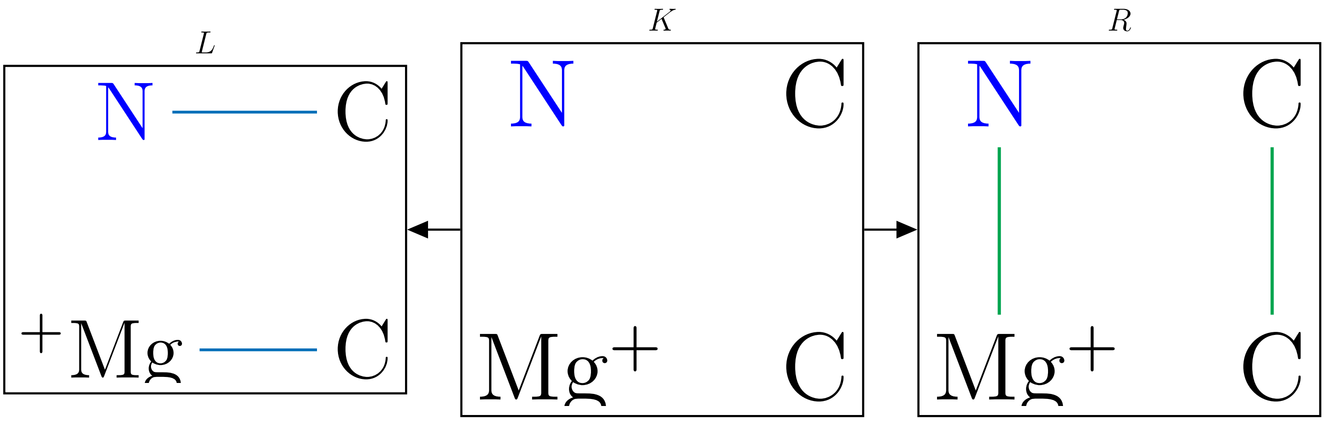

Files: out/488_r_97_11300100_{L, K, R}

0.0.99 98

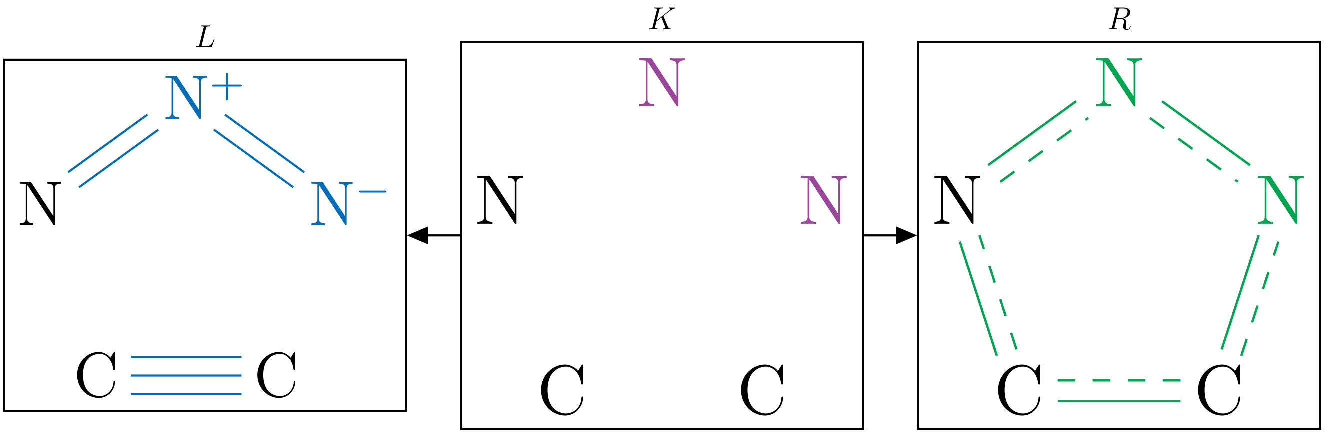

Files: out/491_r_98_10300000_{L, K, R}

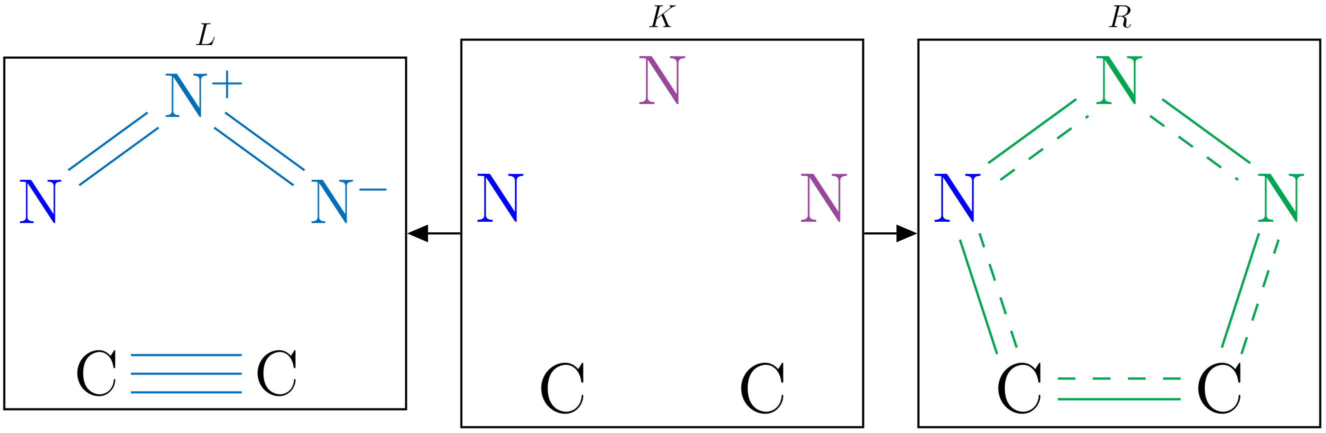

Files: out/493_r_98_11300100_{L, K, R}

0.0.100 99

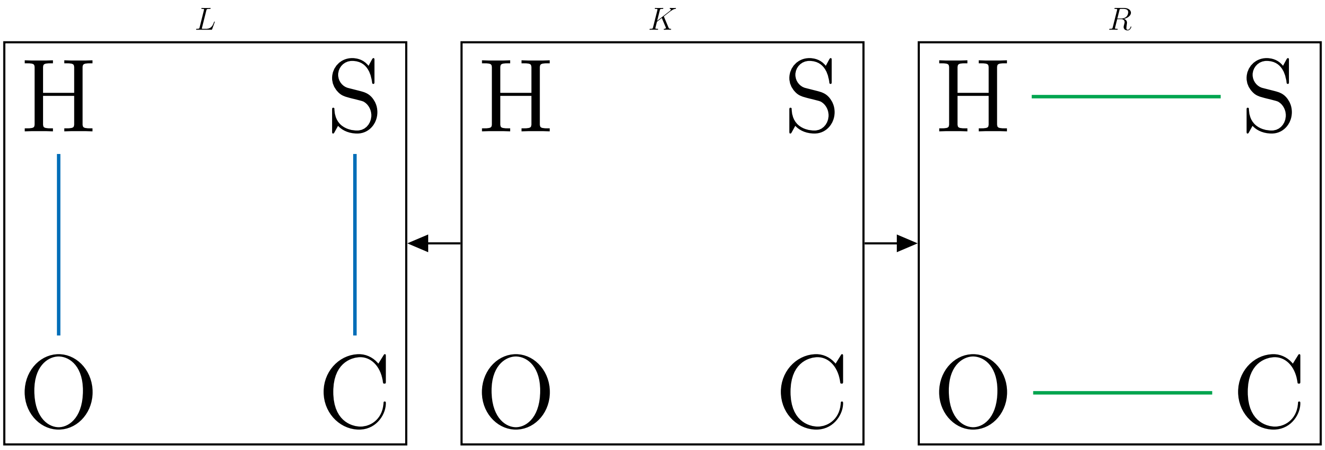

Files: out/496_r_99_10300000_{L, K, R}

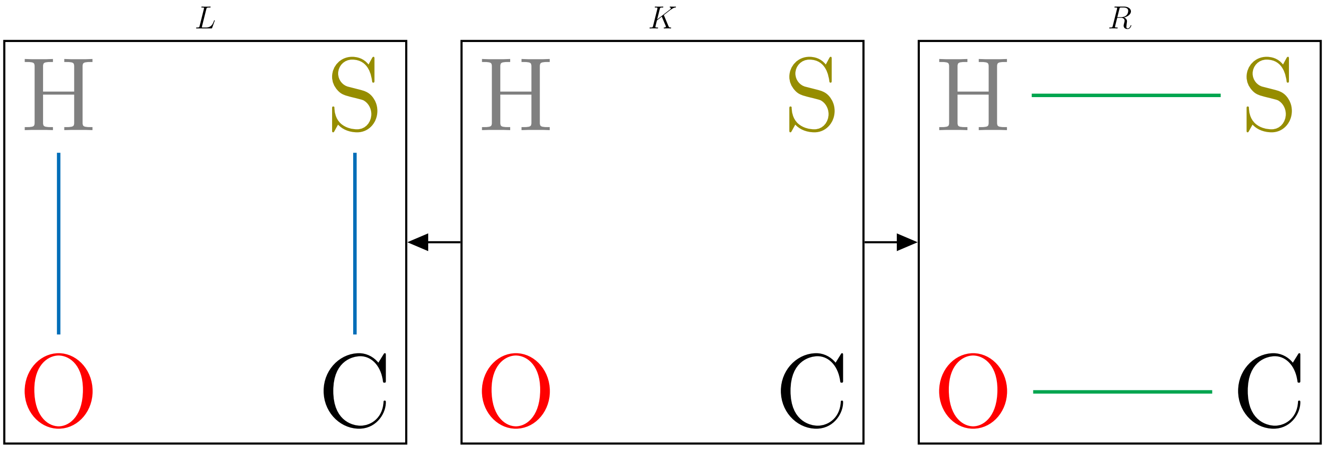

Files: out/498_r_99_11300100_{L, K, R}

0.0.101 100

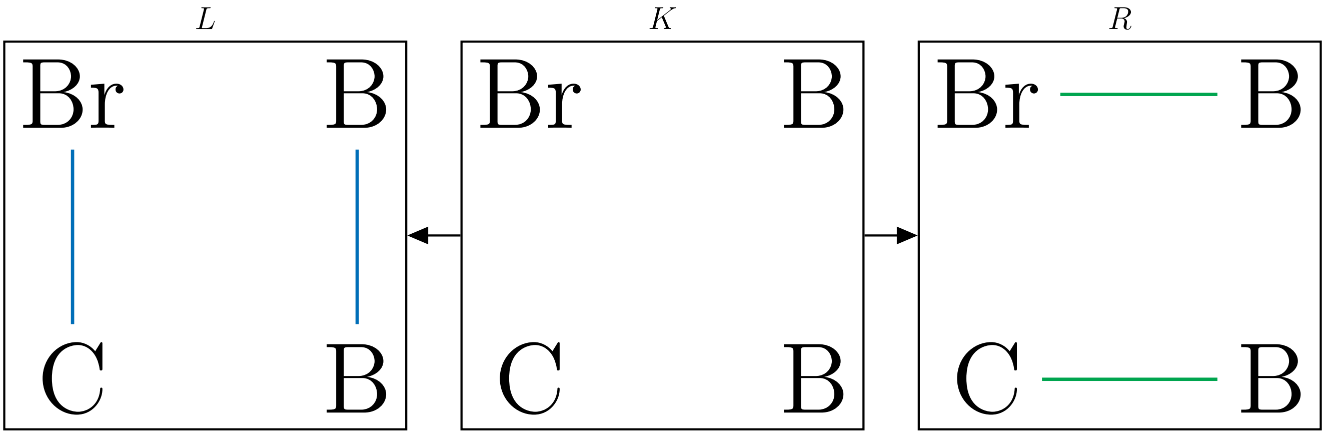

Files: out/501_r_100_10300000_{L, K, R}

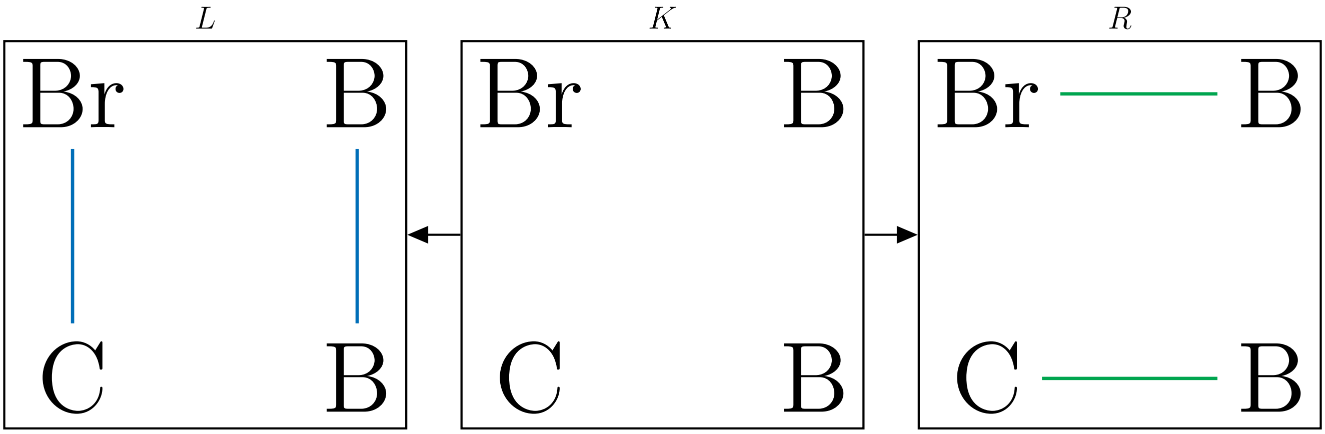

Files: out/503_r_100_11300100_{L, K, R}

0.0.102 101

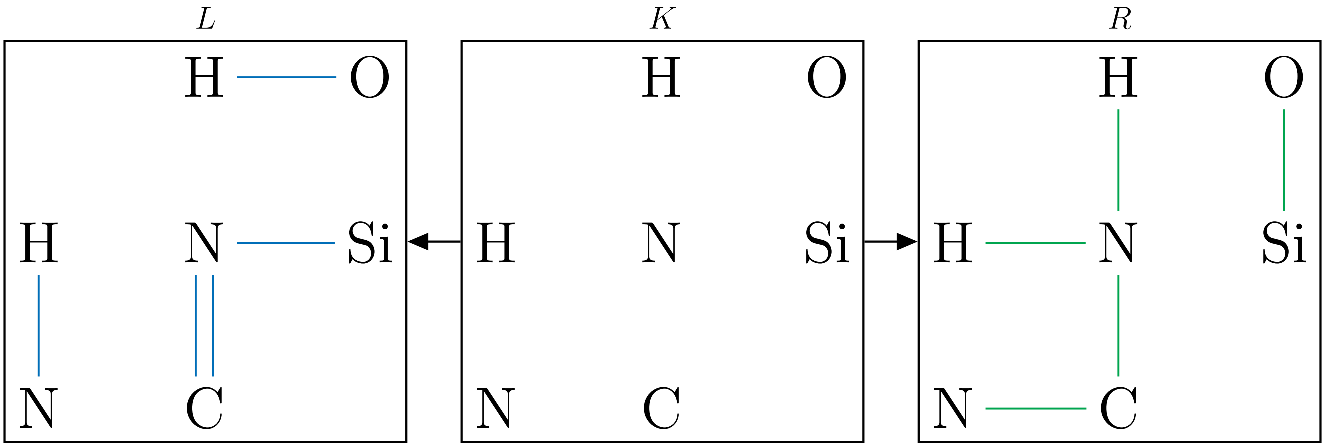

Files: out/506_r_101_10300000_{L, K, R}

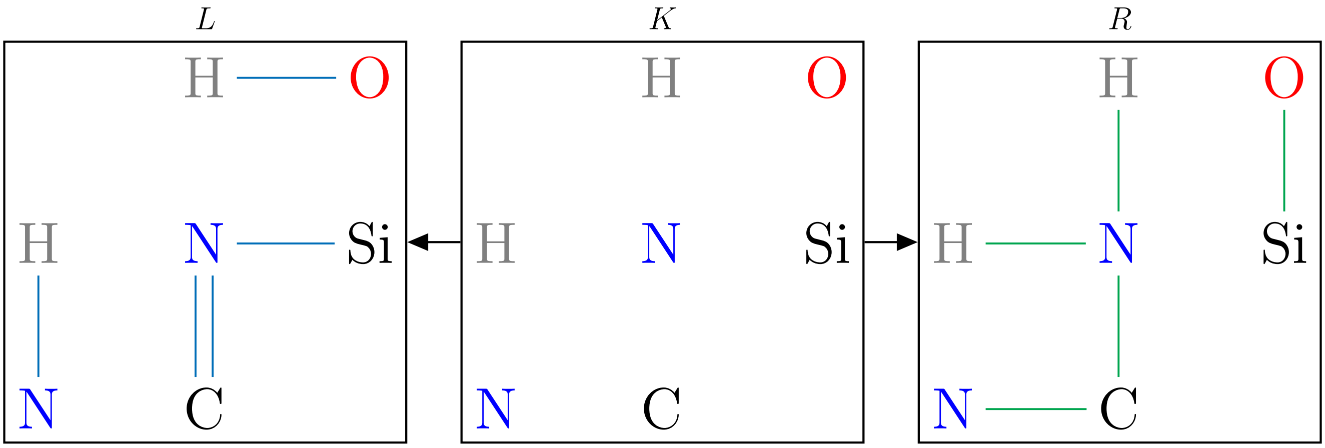

Files: out/508_r_101_11300100_{L, K, R}

0.0.103 102

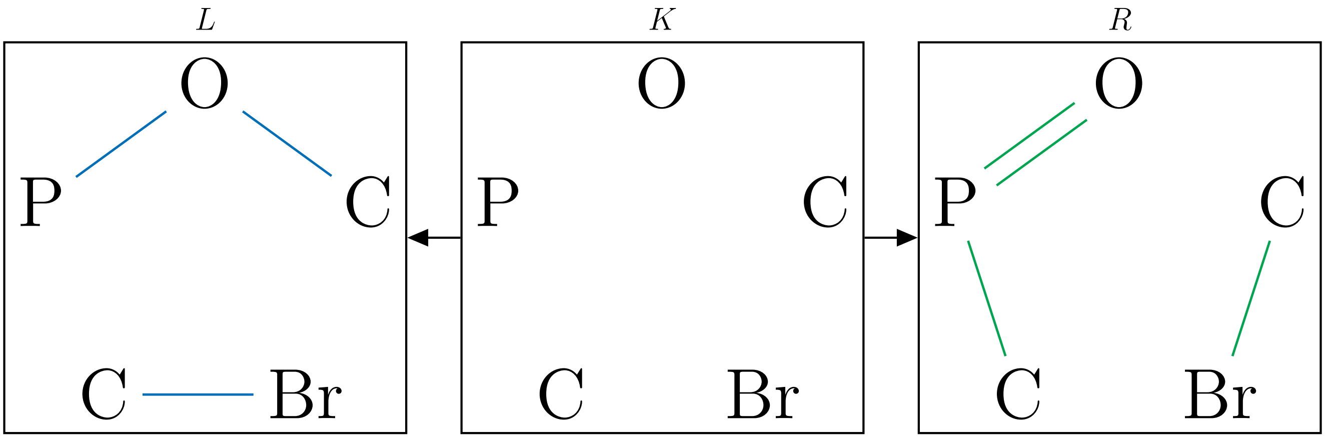

Files: out/511_r_102_10300000_{L, K, R}

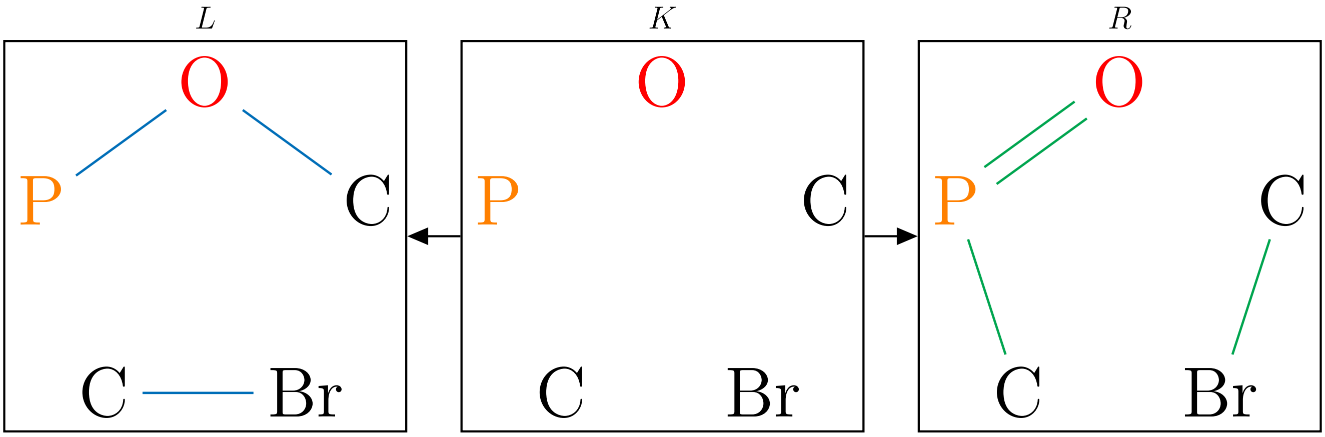

Files: out/513_r_102_11300100_{L, K, R}

0.0.104 103

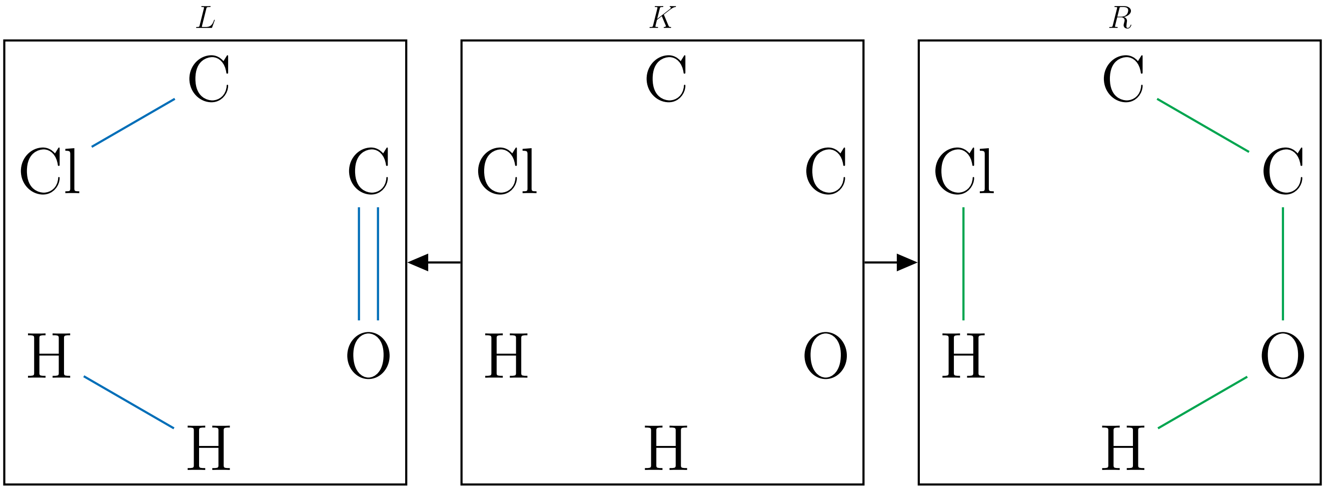

Files: out/516_r_103_10300000_{L, K, R}

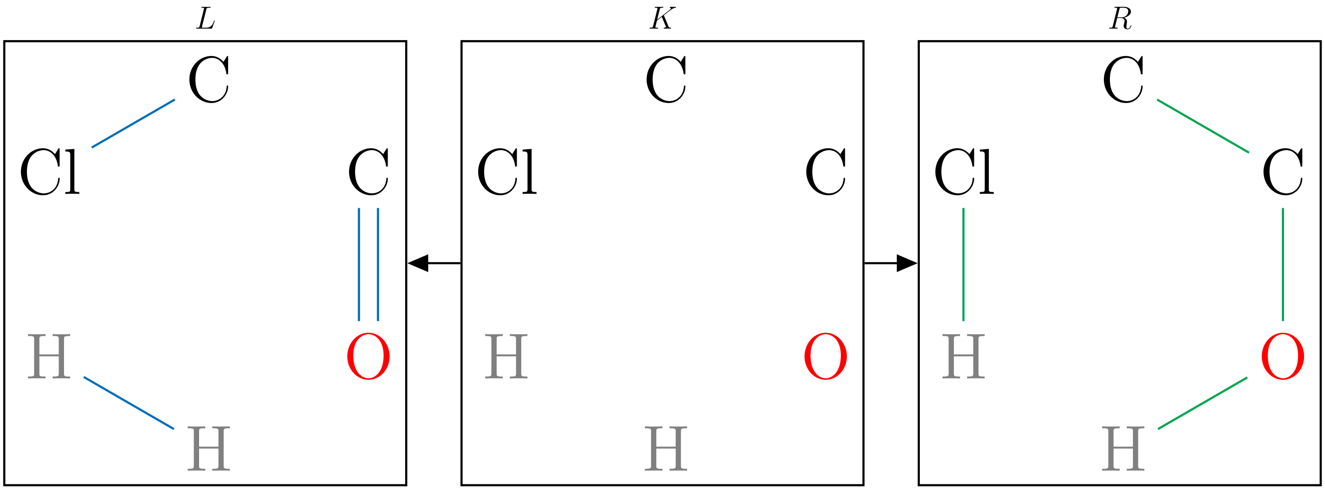

Files: out/518_r_103_11300100_{L, K, R}

0.0.105 104

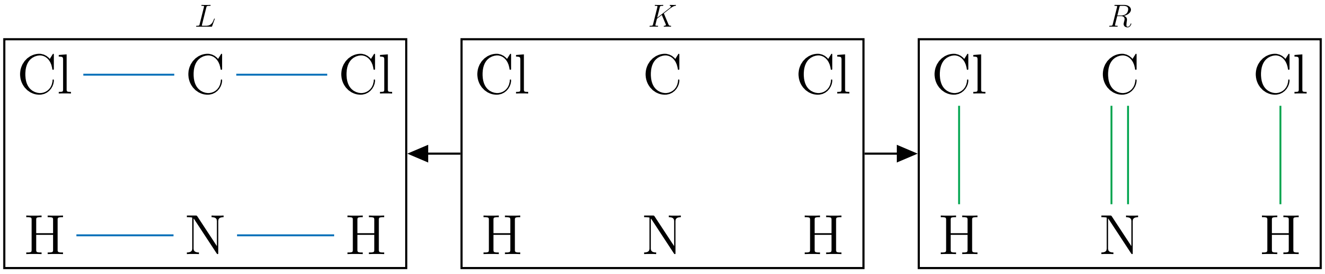

Files: out/521_r_104_10300000_{L, K, R}

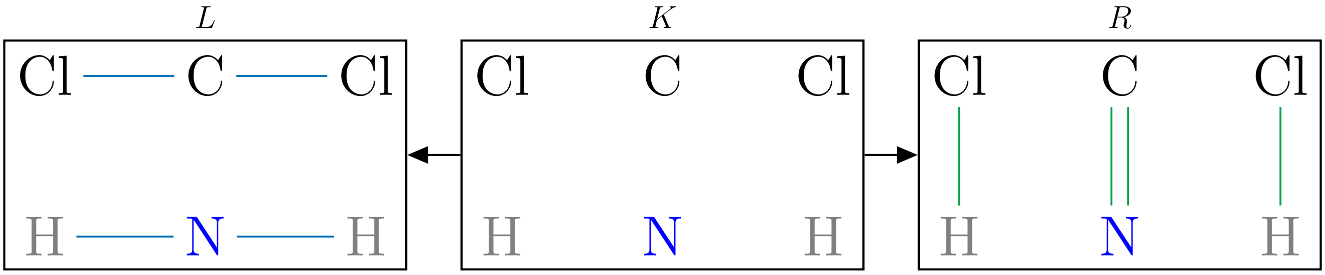

Files: out/523_r_104_11300100_{L, K, R}

0.0.106 105

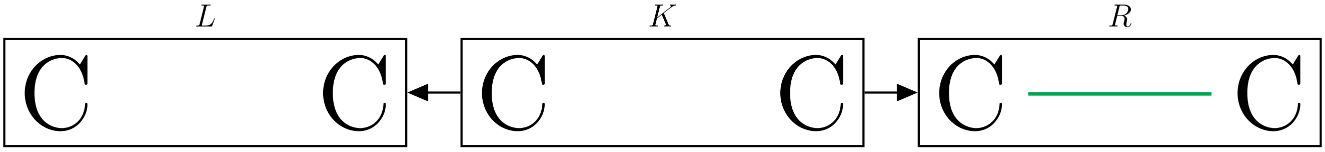

Files: out/526_r_105_10300000_{L, K, R}

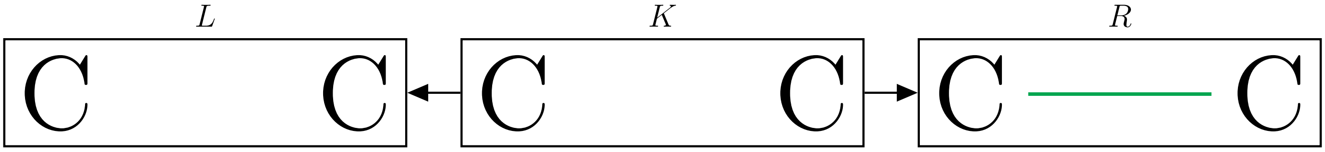

Files: out/528_r_105_11300100_{L, K, R}

0.0.107 106

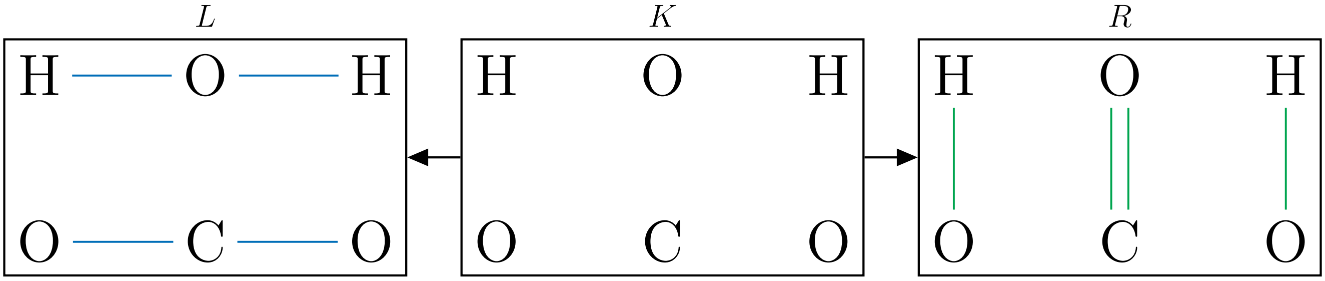

Files: out/531_r_106_10300000_{L, K, R}

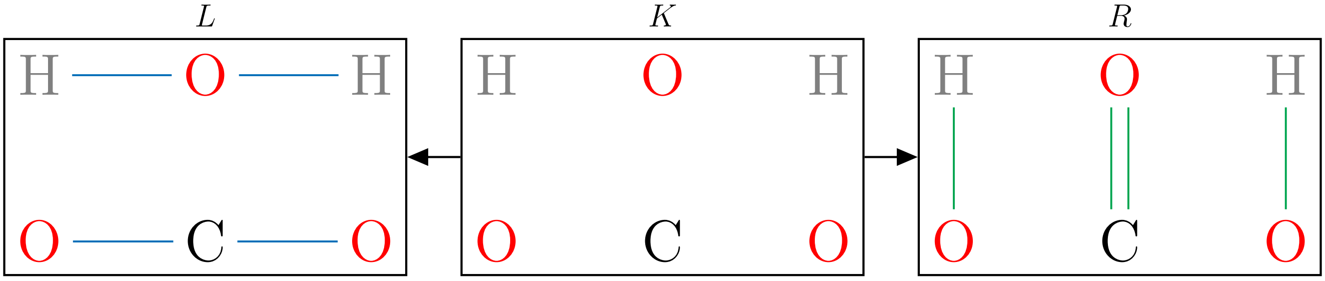

Files: out/533_r_106_11300100_{L, K, R}

0.0.108 107

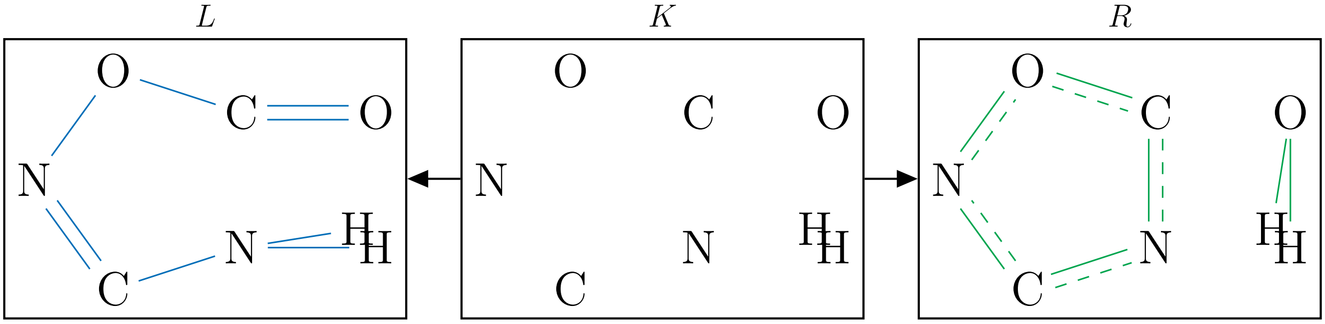

Files: out/536_r_107_10300000_{L, K, R}

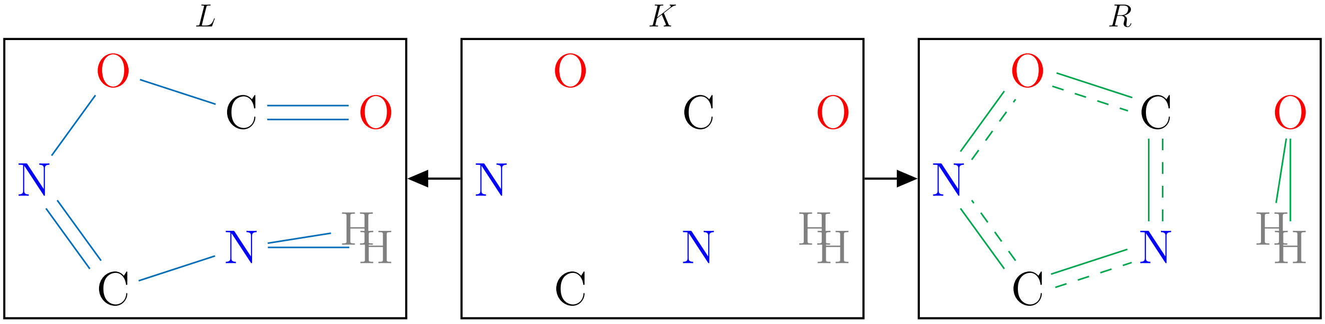

Files: out/538_r_107_11300100_{L, K, R}

0.0.109 108

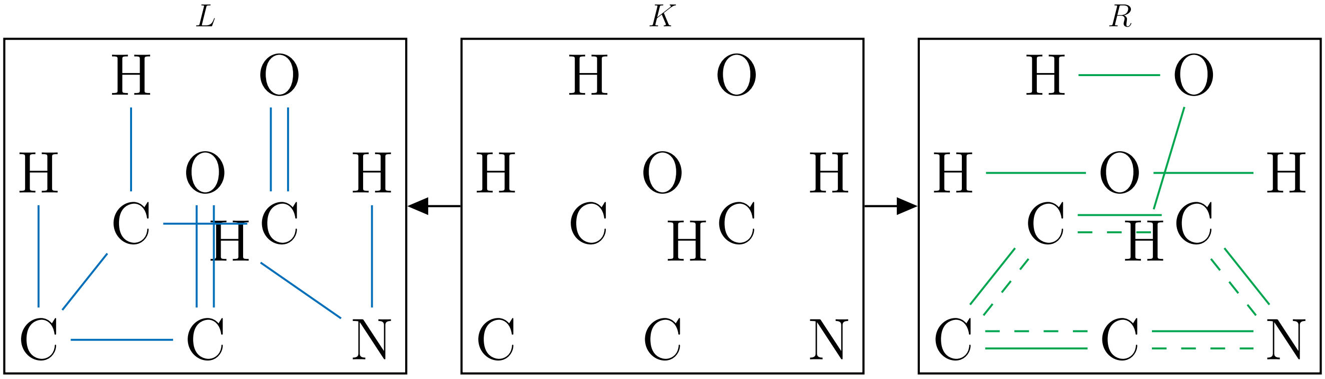

Files: out/541_r_108_10300000_{L, K, R}

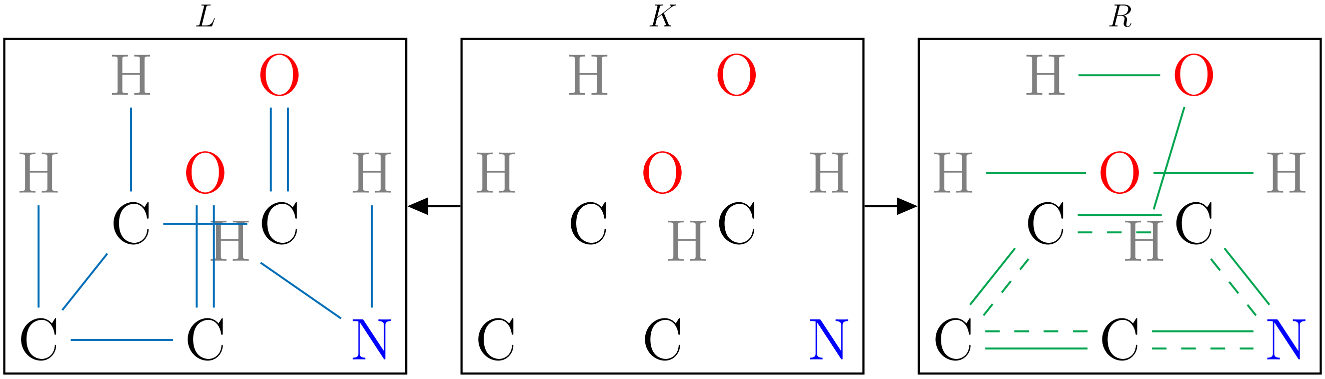

Files: out/543_r_108_11300100_{L, K, R}

0.0.110 109

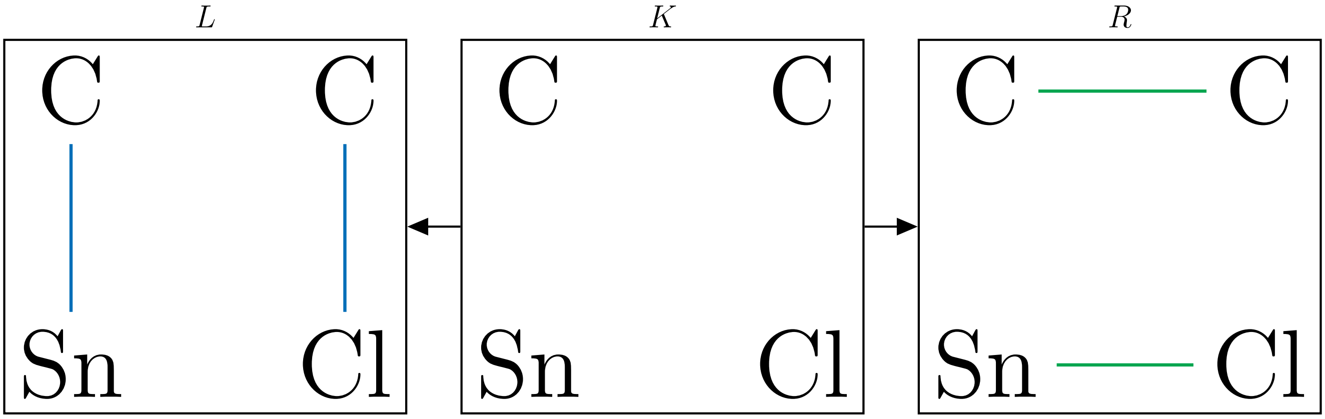

Files: out/546_r_109_10300000_{L, K, R}

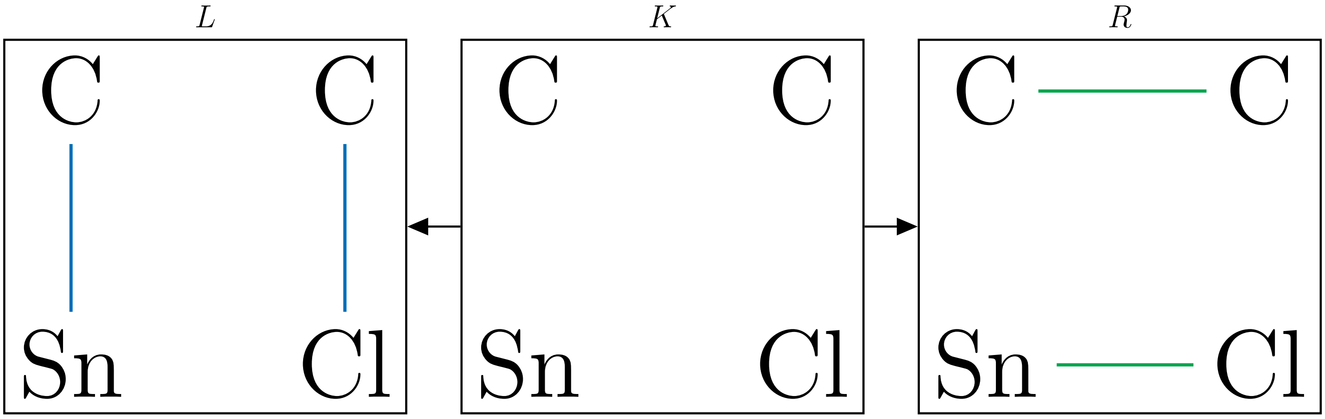

Files: out/548_r_109_11300100_{L, K, R}

0.0.111 110

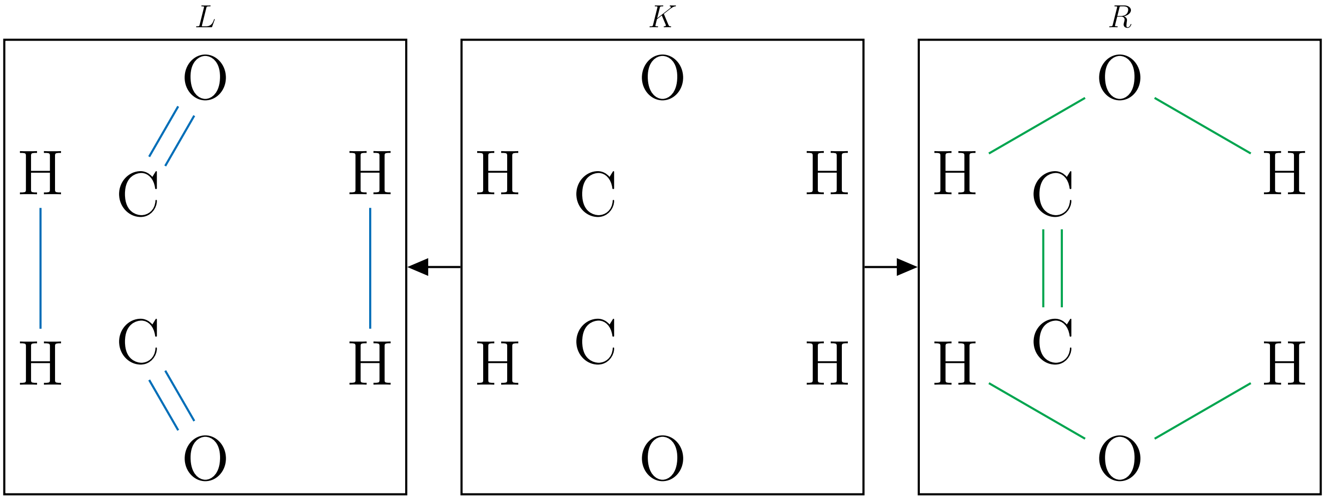

Files: out/551_r_110_10300000_{L, K, R}

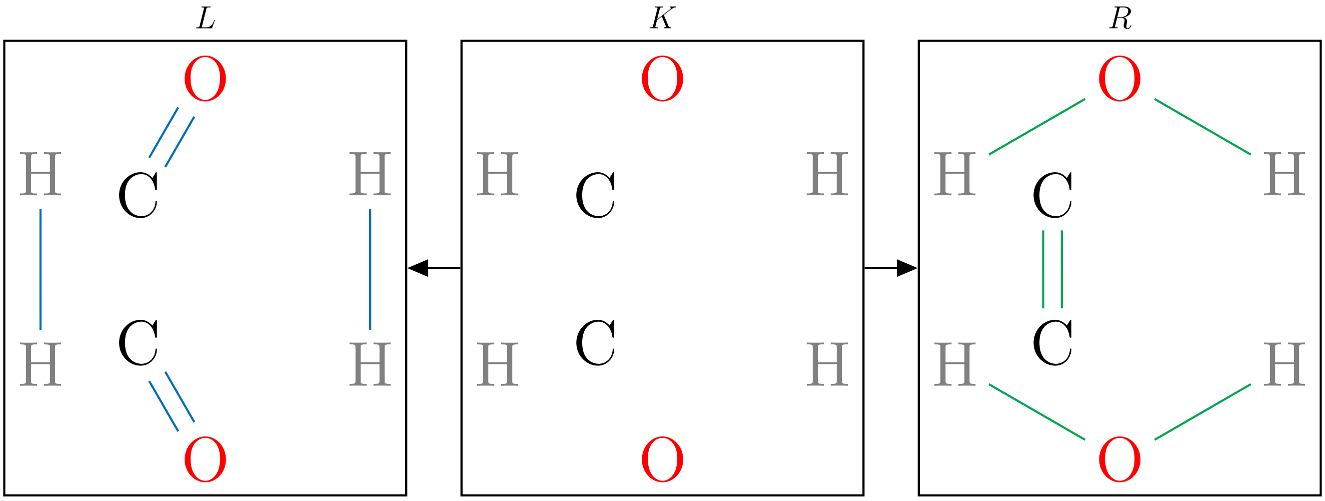

Files: out/553_r_110_11300100_{L, K, R}

0.0.112 111

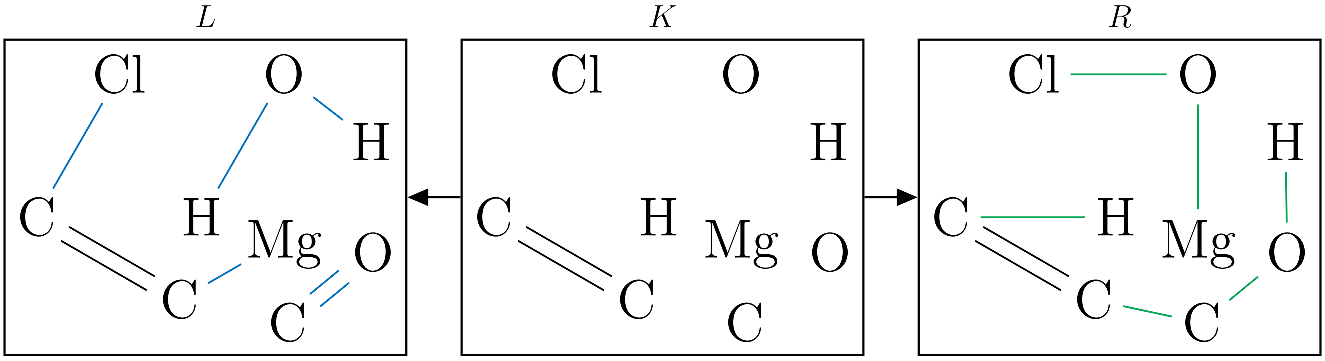

Files: out/556_r_111_10300000_{L, K, R}

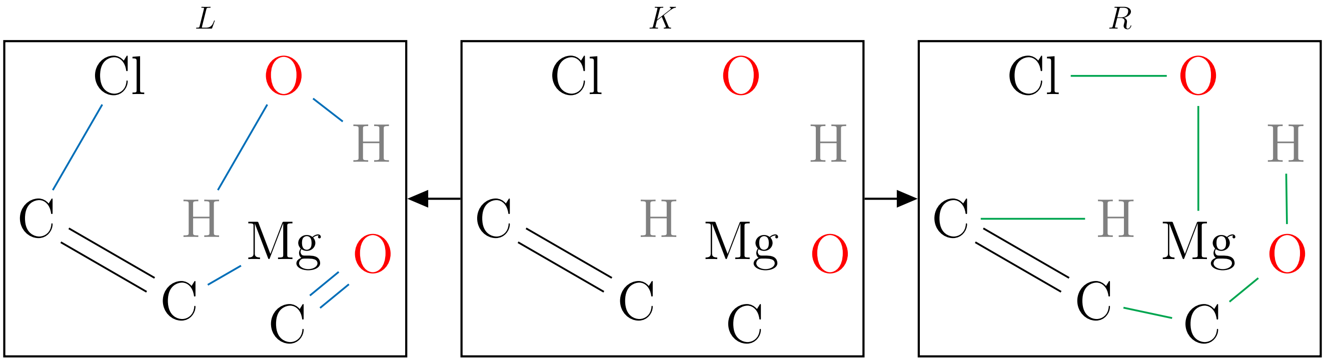

Files: out/558_r_111_11300100_{L, K, R}

0.0.113 112

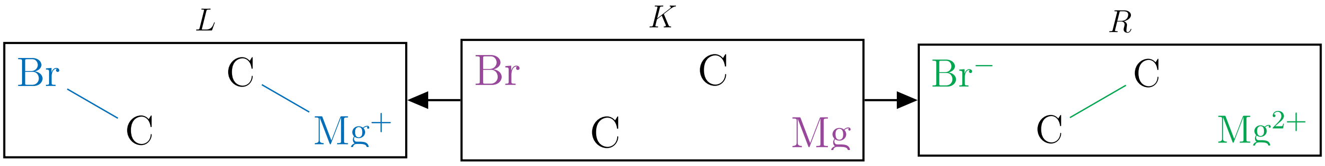

Files: out/561_r_112_10300000_{L, K, R}

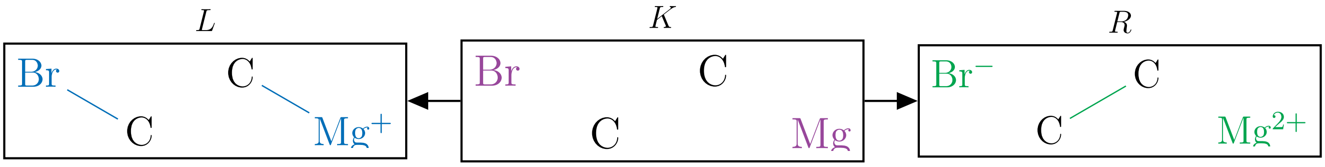

Files: out/563_r_112_11300100_{L, K, R}

0.0.114 113

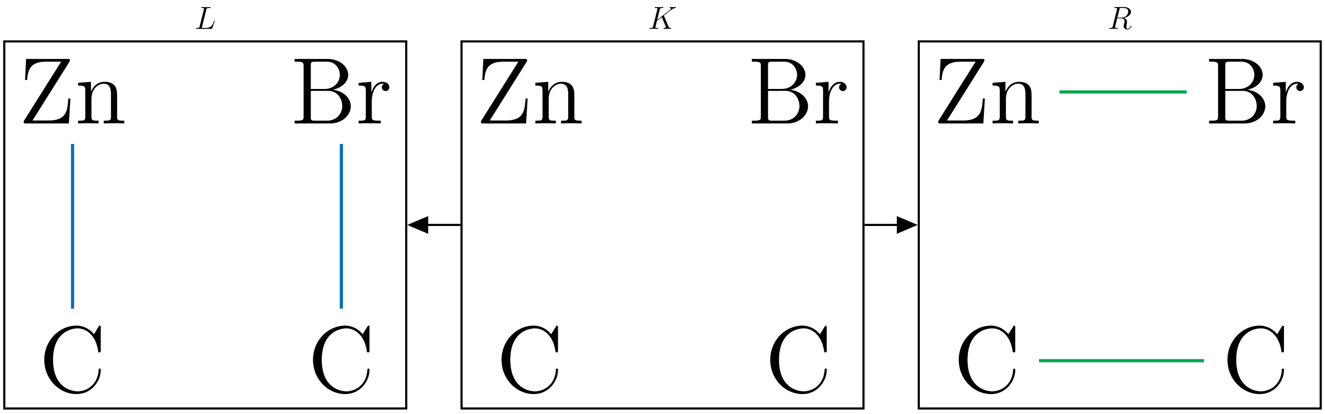

Files: out/566_r_113_10300000_{L, K, R}

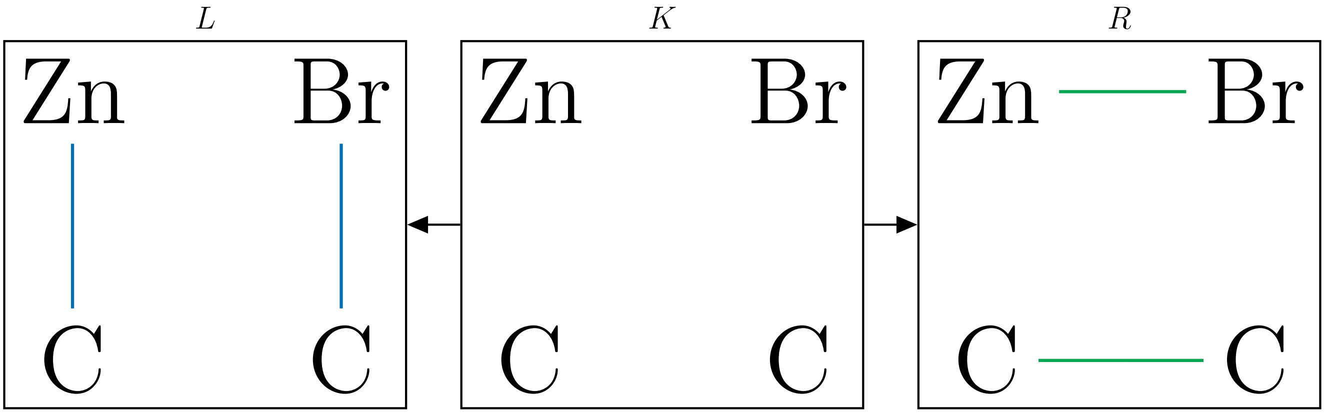

Files: out/568_r_113_11300100_{L, K, R}

0.0.115 114

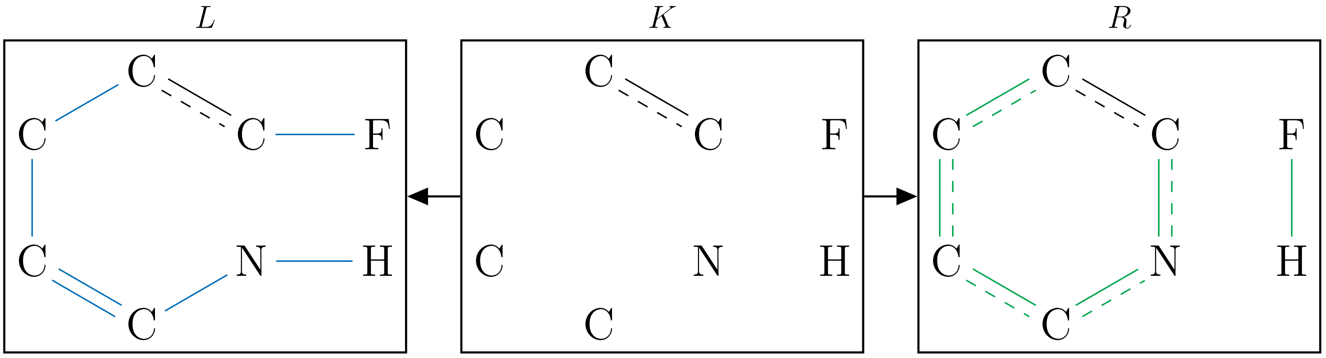

Files: out/571_r_114_10300000_{L, K, R}

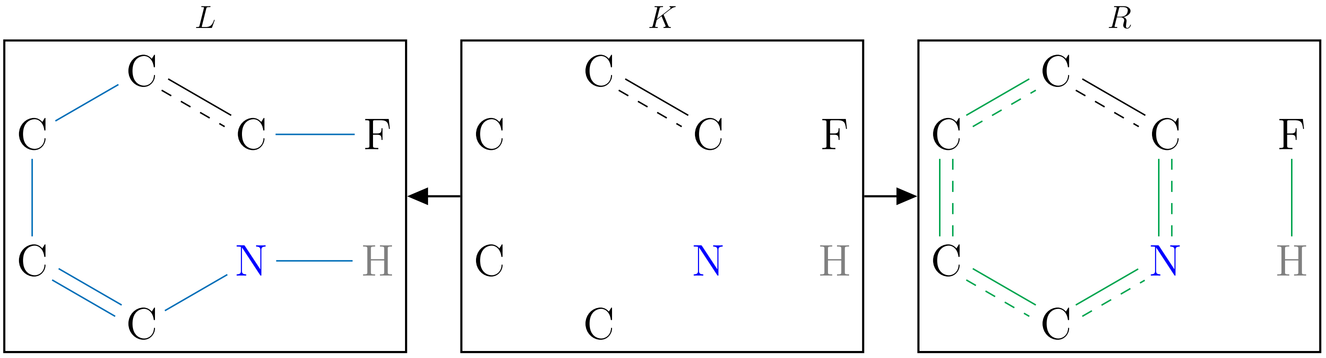

Files: out/573_r_114_11300100_{L, K, R}

0.0.116 115

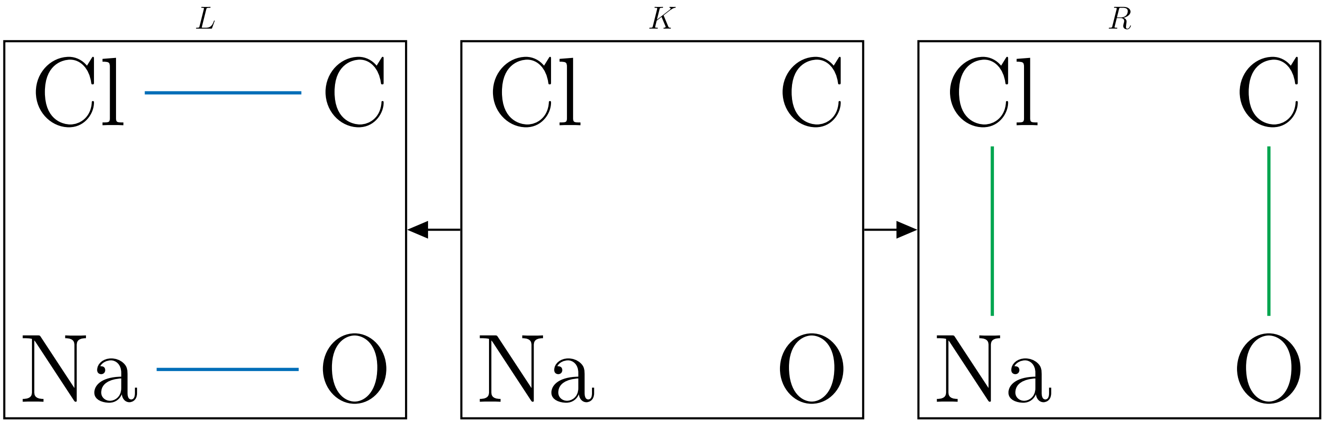

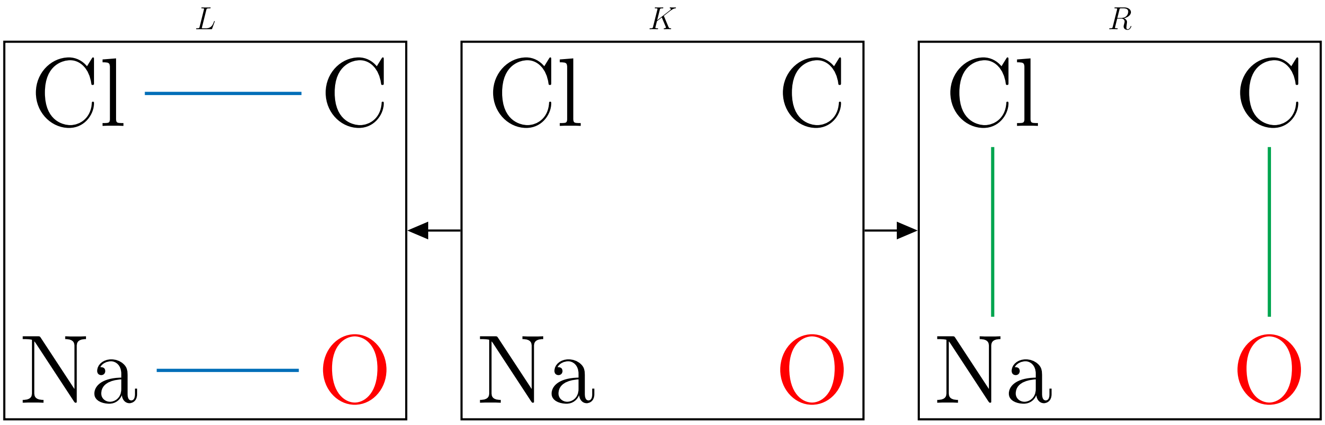

Files: out/578_r_115_11300100_{L, K, R}

0.0.117 116

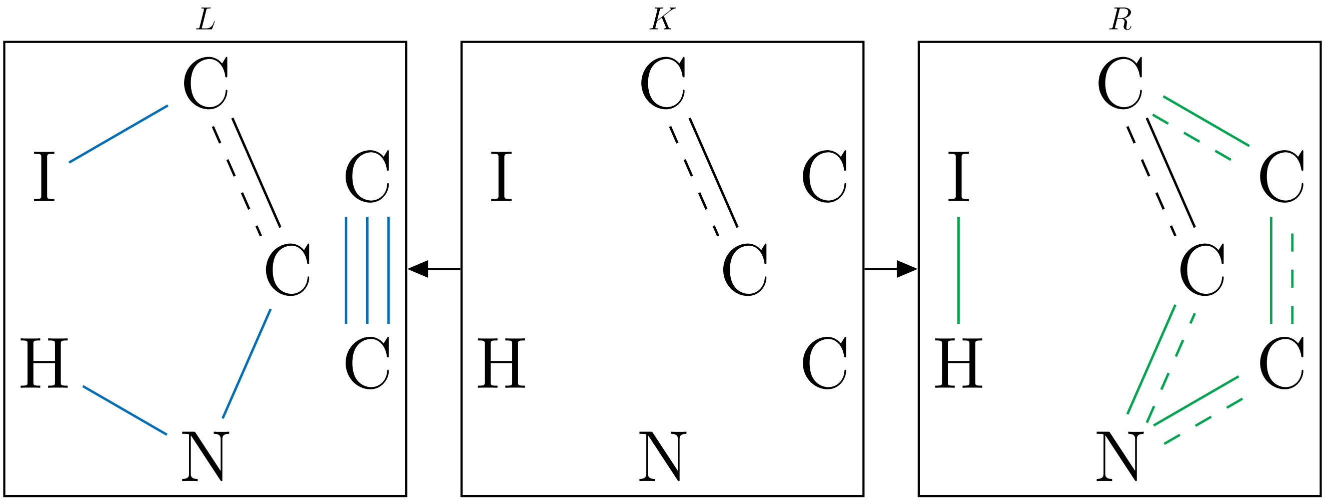

Files: out/581_r_116_10300000_{L, K, R}

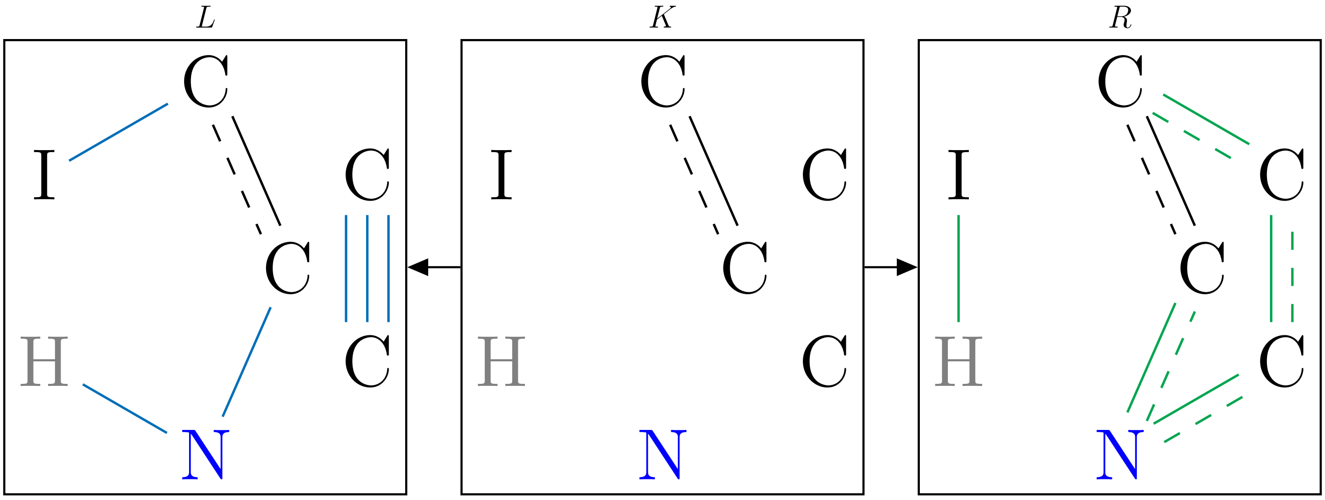

Files: out/583_r_116_11300100_{L, K, R}

0.0.118 117

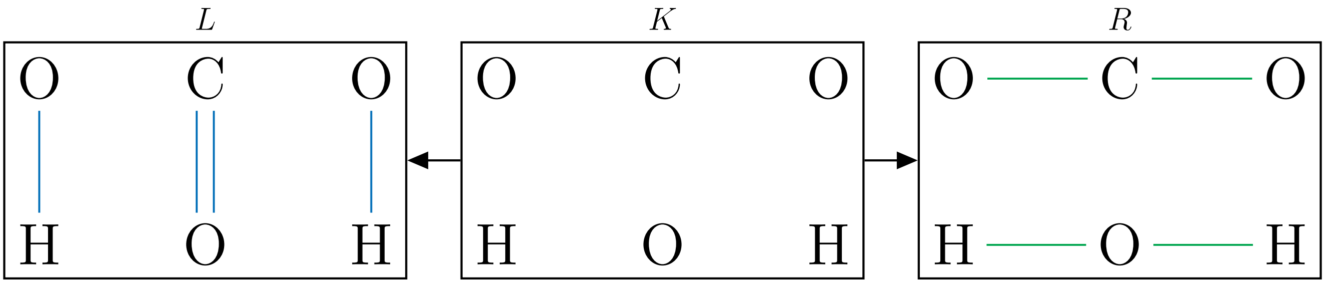

Files: out/586_r_117_10300000_{L, K, R}

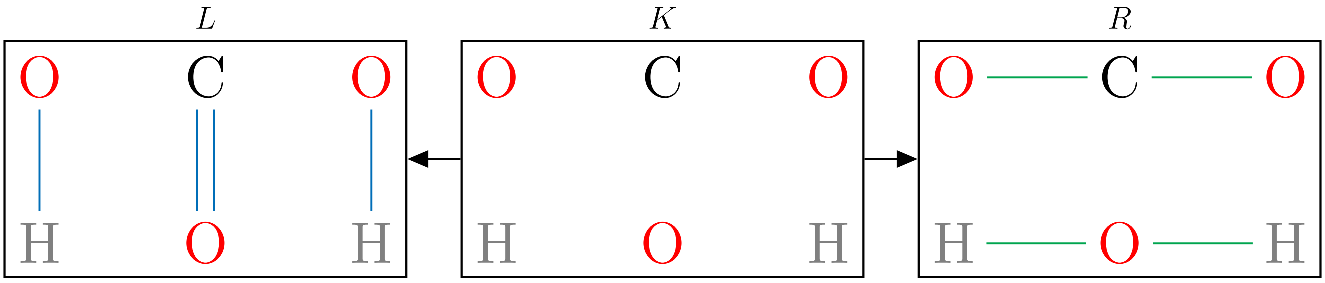

Files: out/588_r_117_11300100_{L, K, R}

0.0.119 118

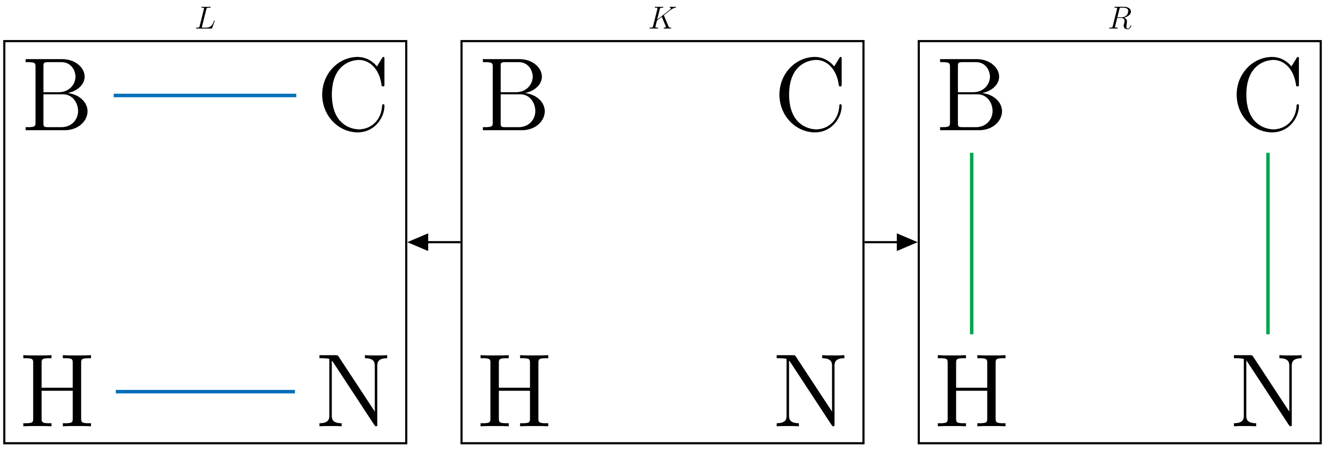

Files: out/591_r_118_10300000_{L, K, R}

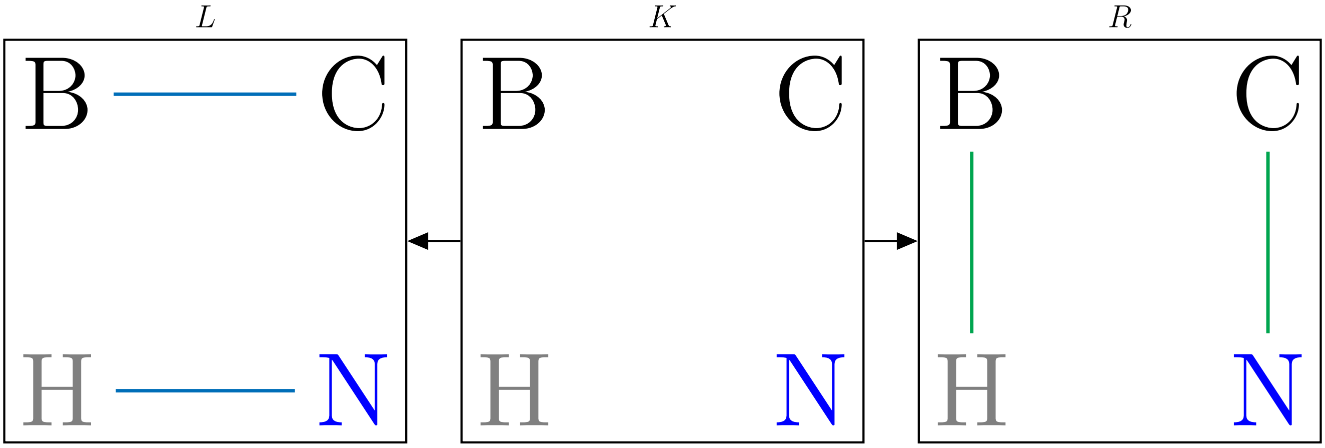

Files: out/593_r_118_11300100_{L, K, R}

0.0.120 119

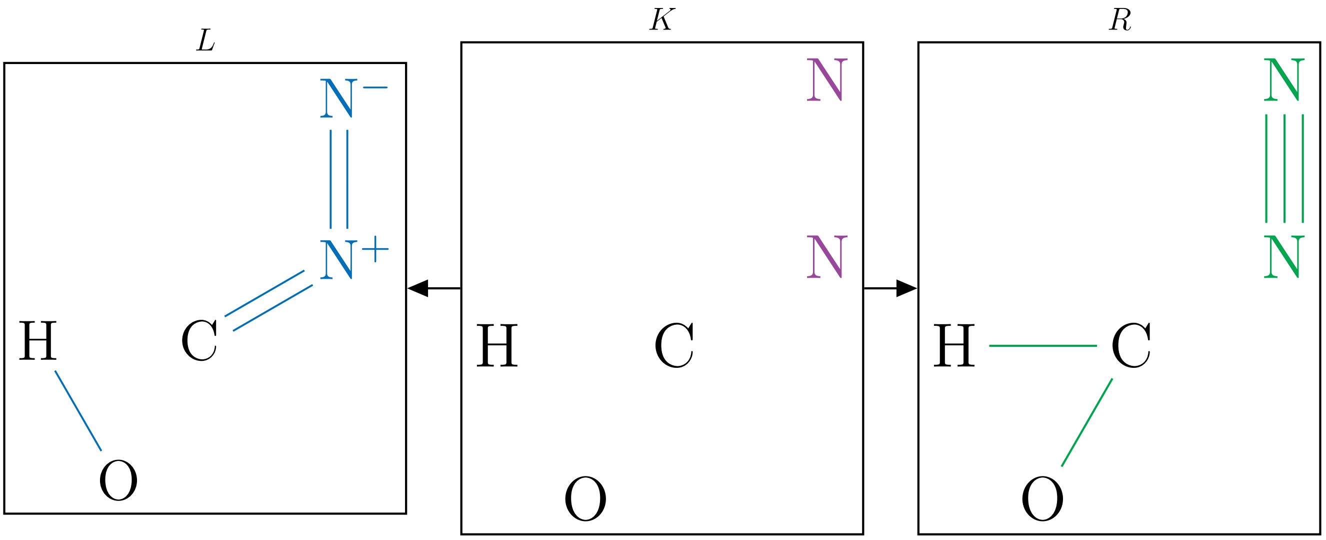

Files: out/596_r_119_10300000_{L, K, R}

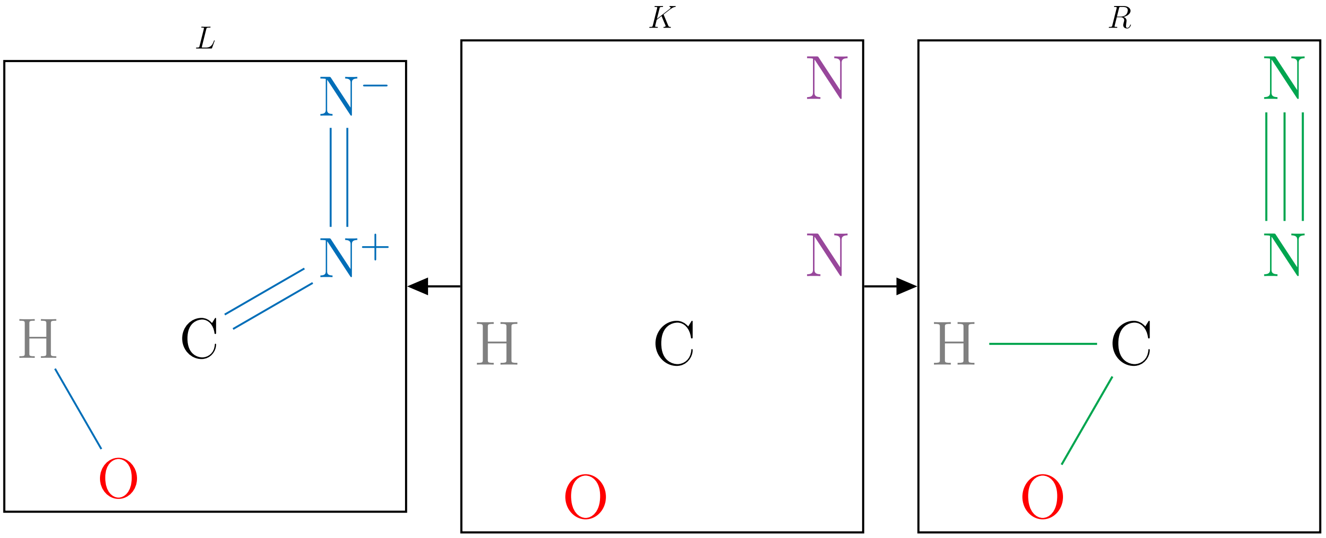

Files: out/598_r_119_11300100_{L, K, R}

0.0.121 120

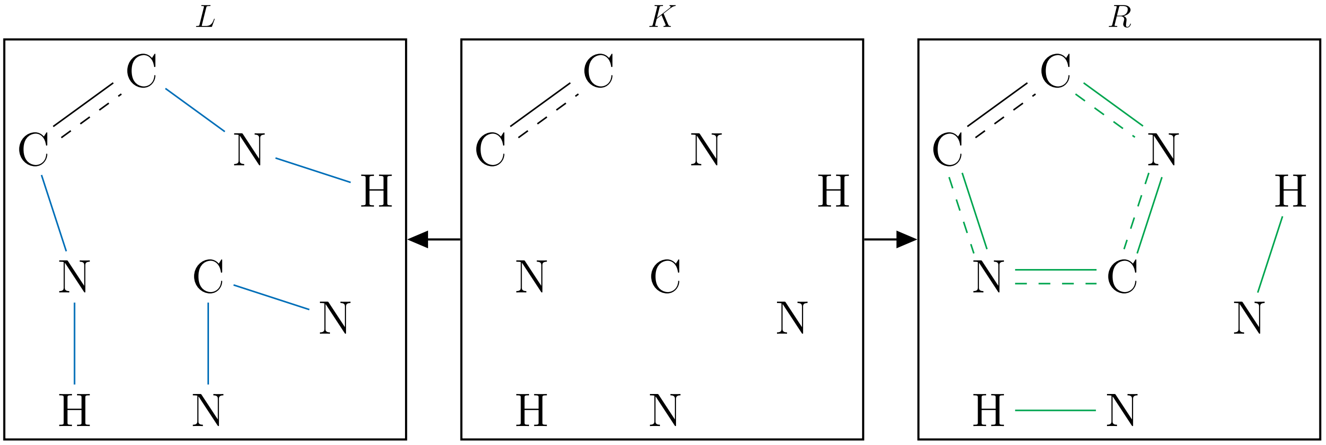

Files: out/601_r_120_10300000_{L, K, R}

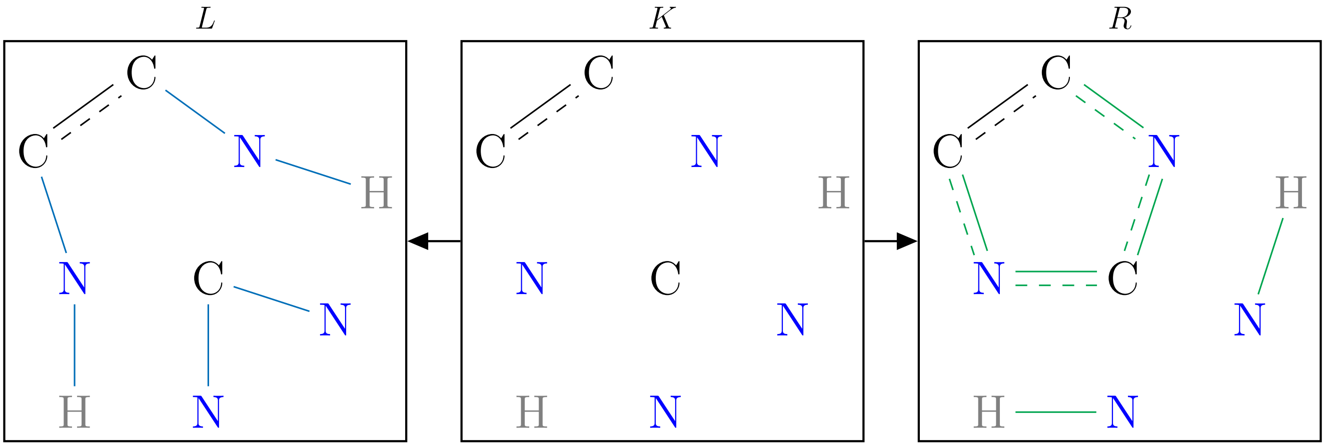

Files: out/603_r_120_11300100_{L, K, R}

0.0.122 121

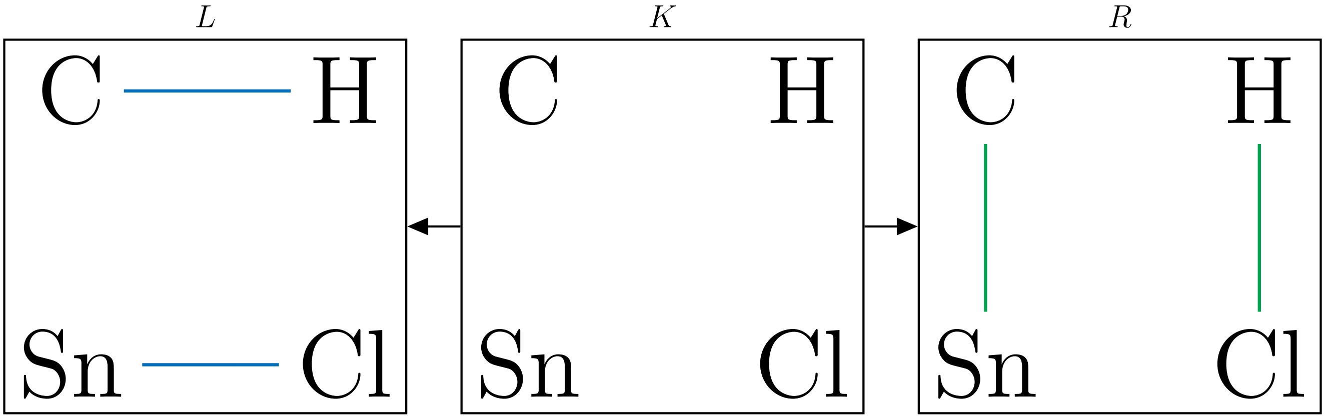

Files: out/606_r_121_10300000_{L, K, R}

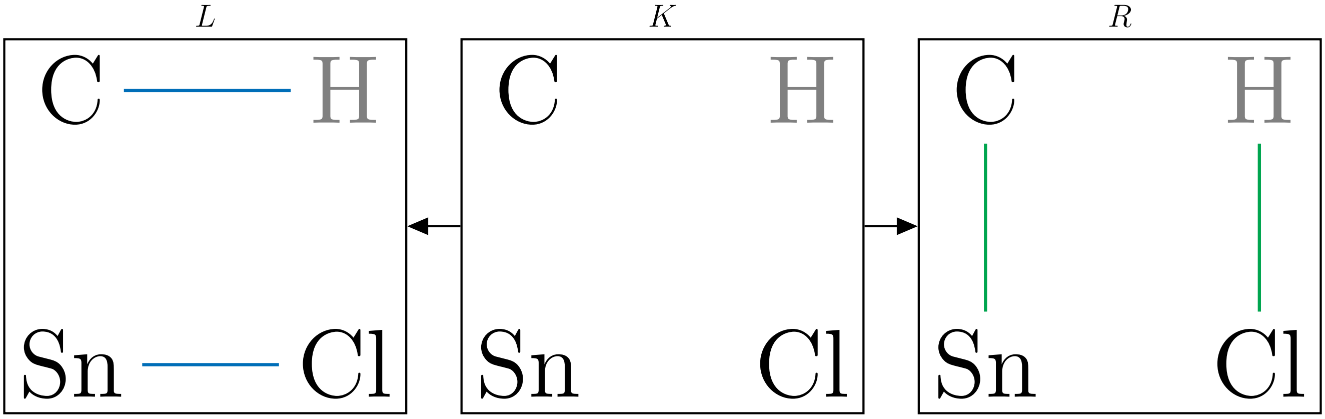

Files: out/608_r_121_11300100_{L, K, R}

0.0.123 122

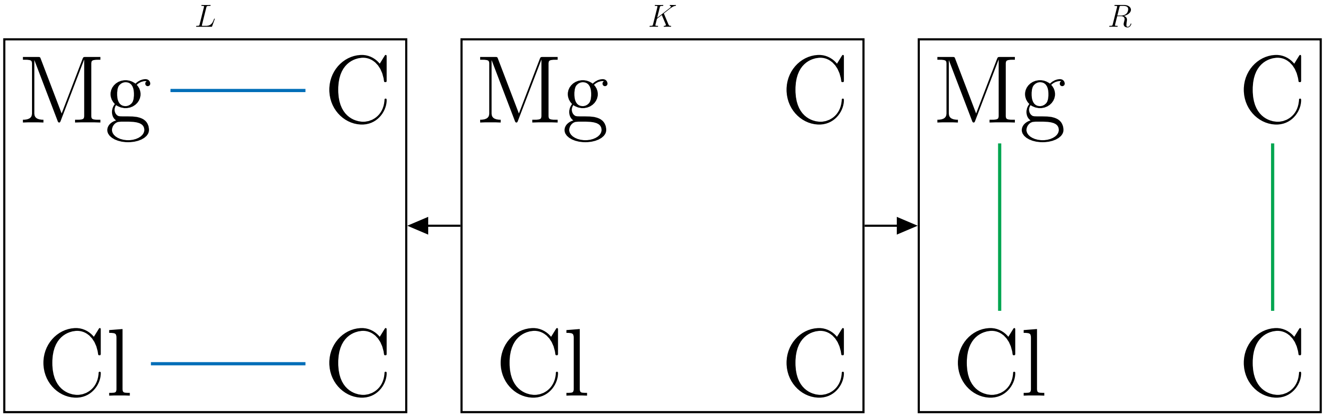

Files: out/611_r_122_10300000_{L, K, R}

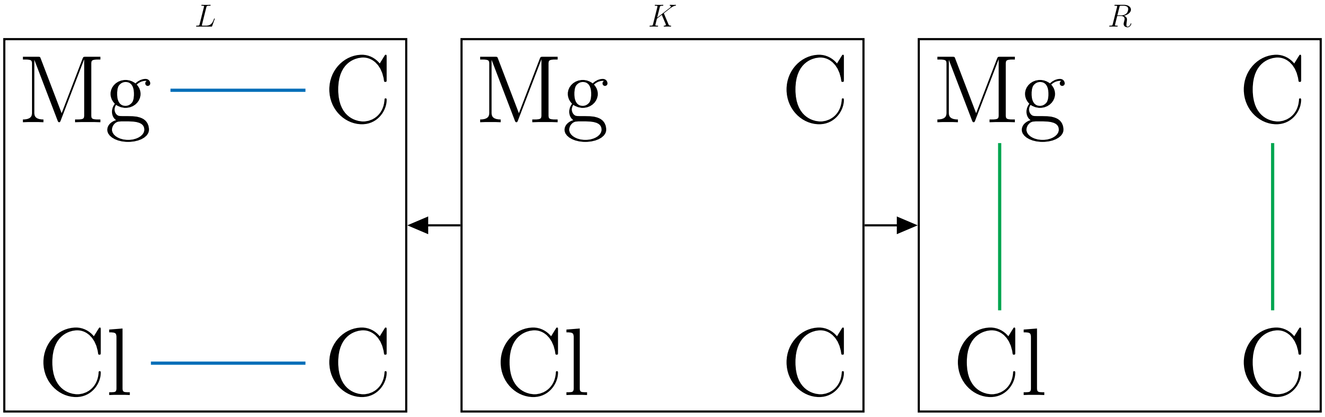

Files: out/613_r_122_11300100_{L, K, R}

0.0.124 123

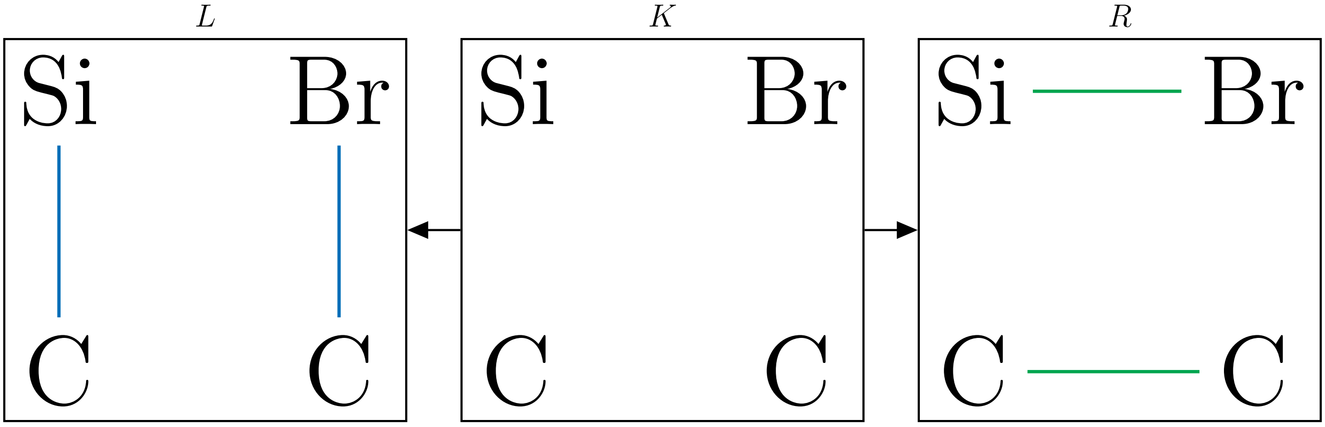

Files: out/616_r_123_10300000_{L, K, R}

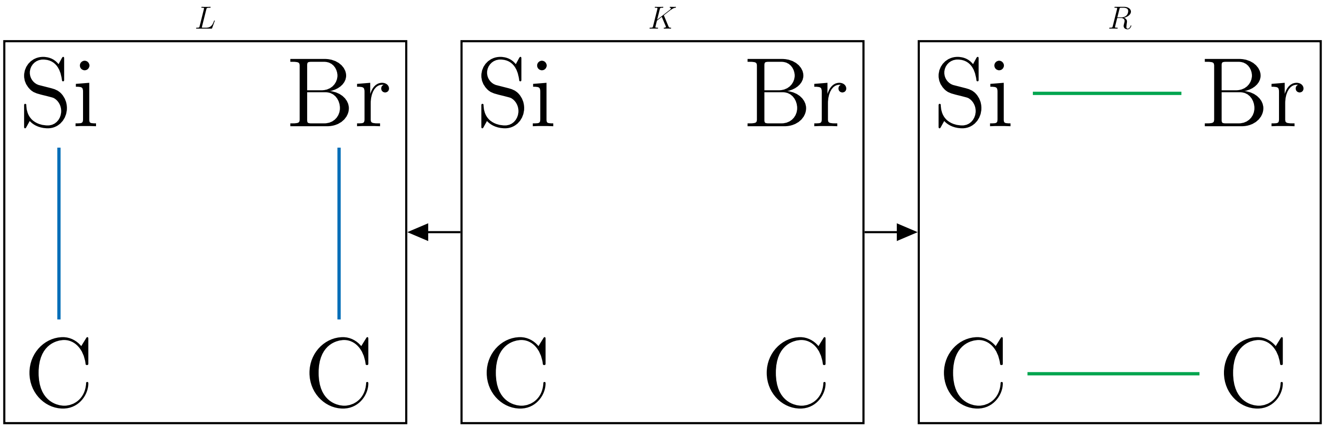

Files: out/618_r_123_11300100_{L, K, R}

0.0.125 124

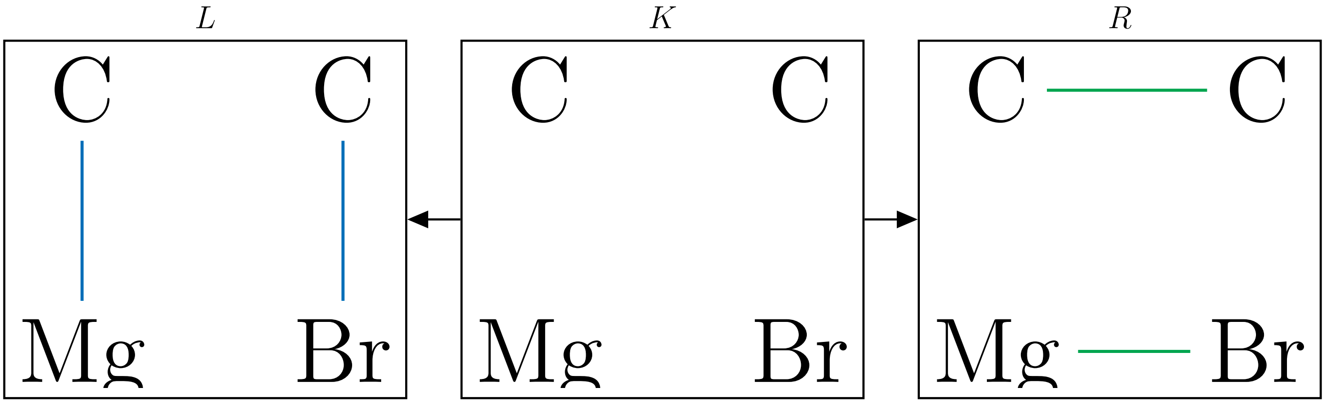

Files: out/621_r_124_10300000_{L, K, R}

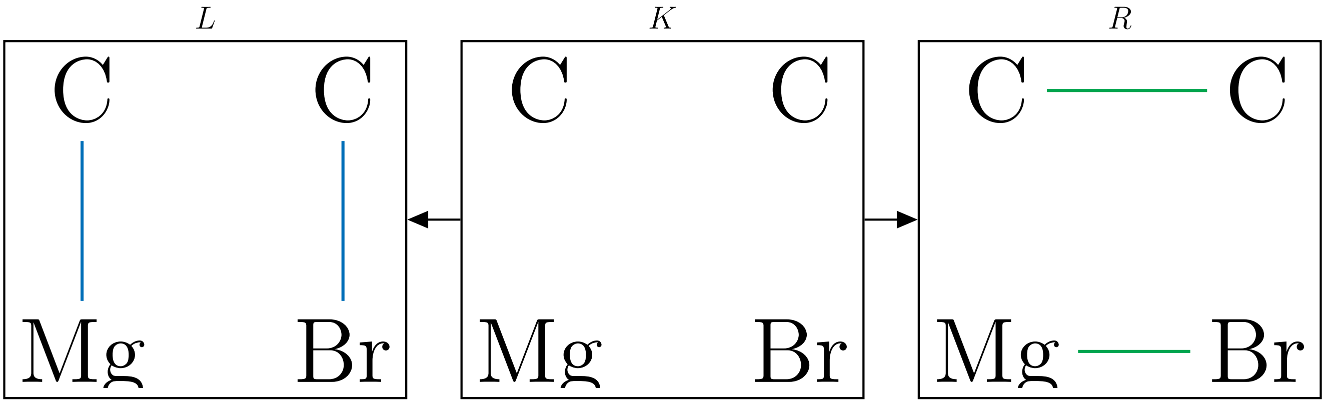

Files: out/623_r_124_11300100_{L, K, R}

0.0.126 125

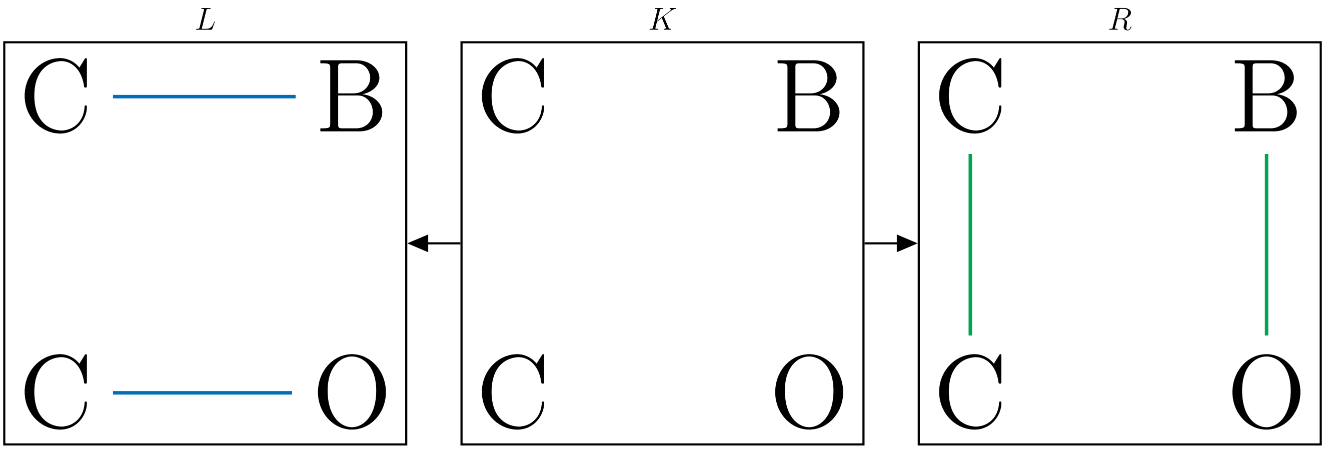

Files: out/626_r_125_10300000_{L, K, R}

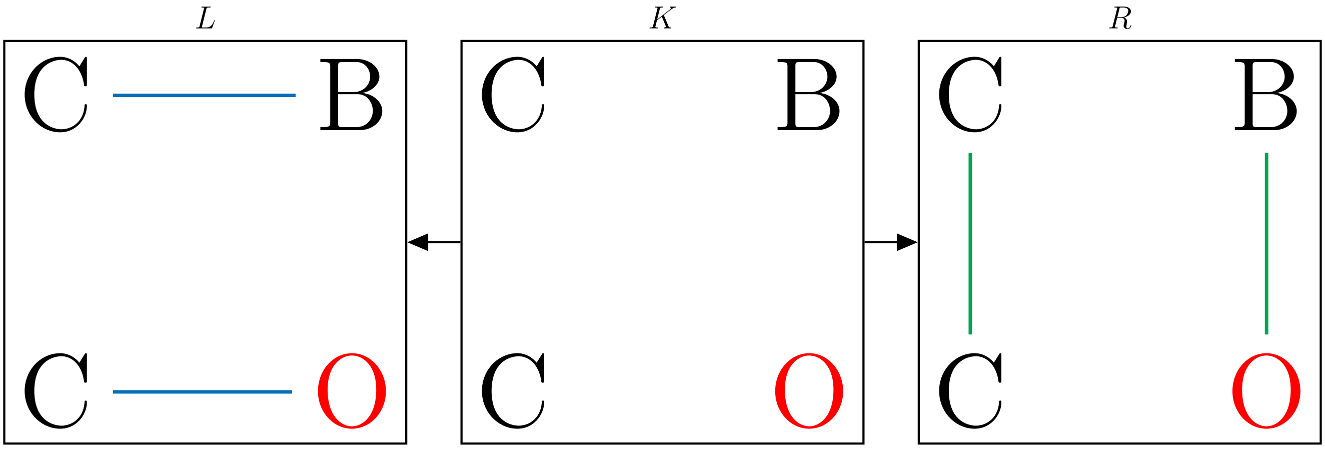

Files: out/628_r_125_11300100_{L, K, R}

0.0.127 126

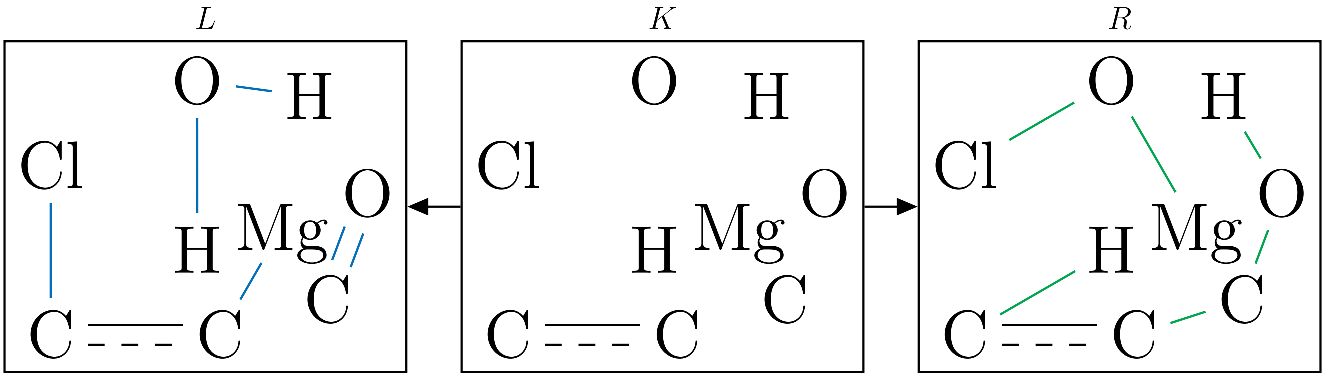

Files: out/631_r_126_10300000_{L, K, R}

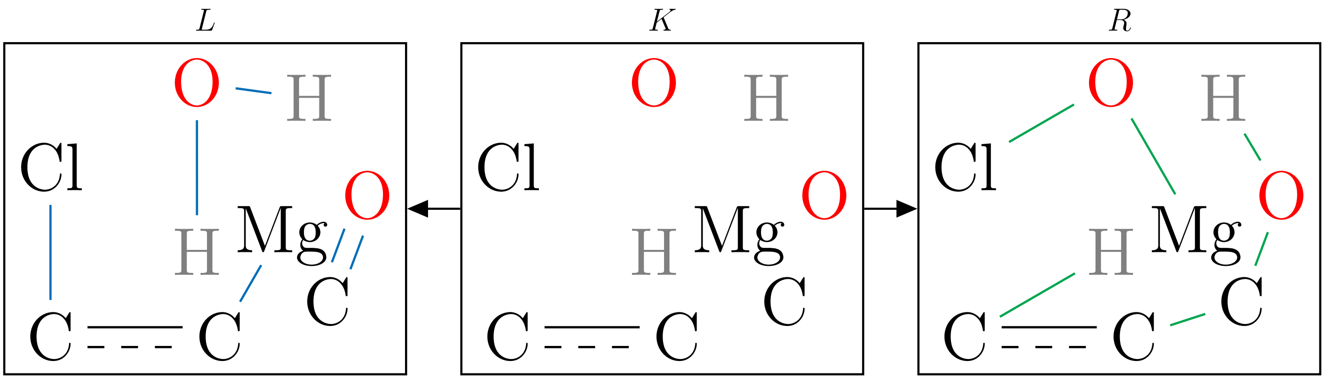

Files: out/633_r_126_11300100_{L, K, R}

0.0.128 127

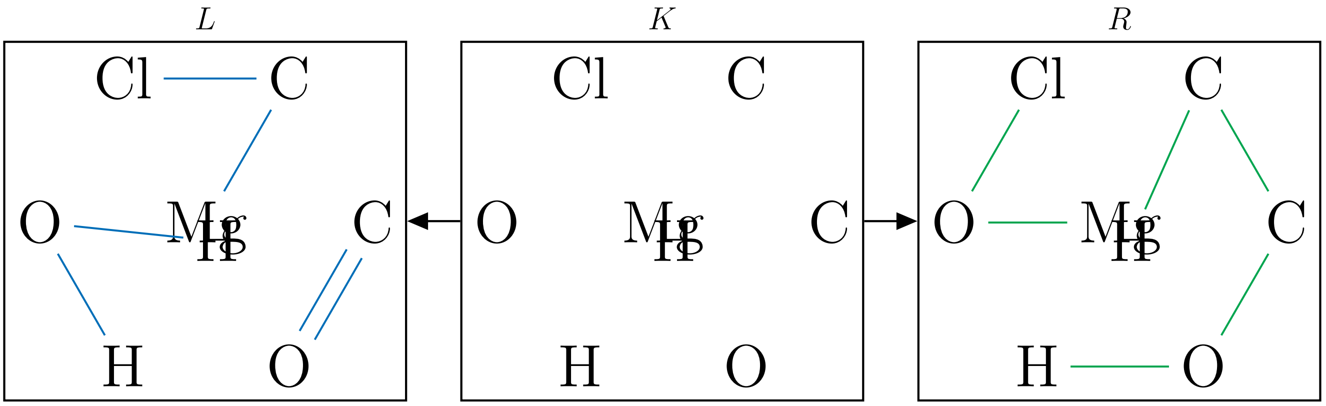

Files: out/636_r_127_10300000_{L, K, R}

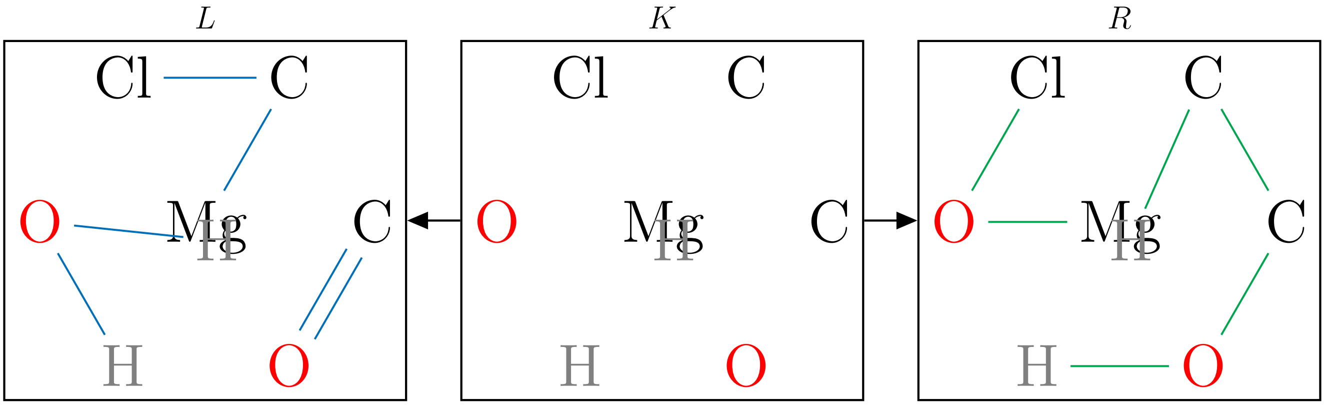

Files: out/638_r_127_11300100_{L, K, R}

0.0.129 128

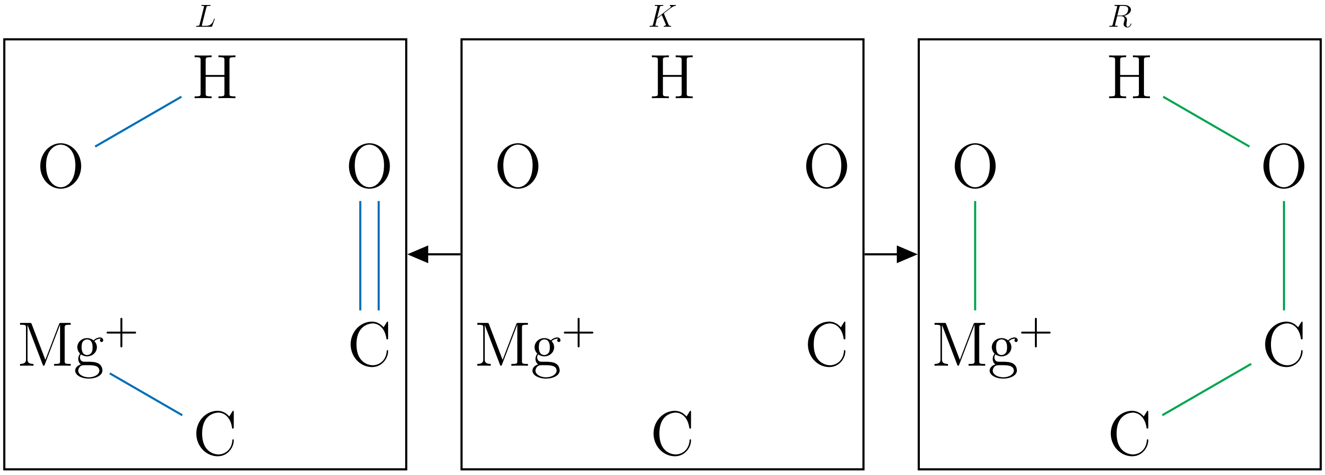

Files: out/641_r_128_10300000_{L, K, R}

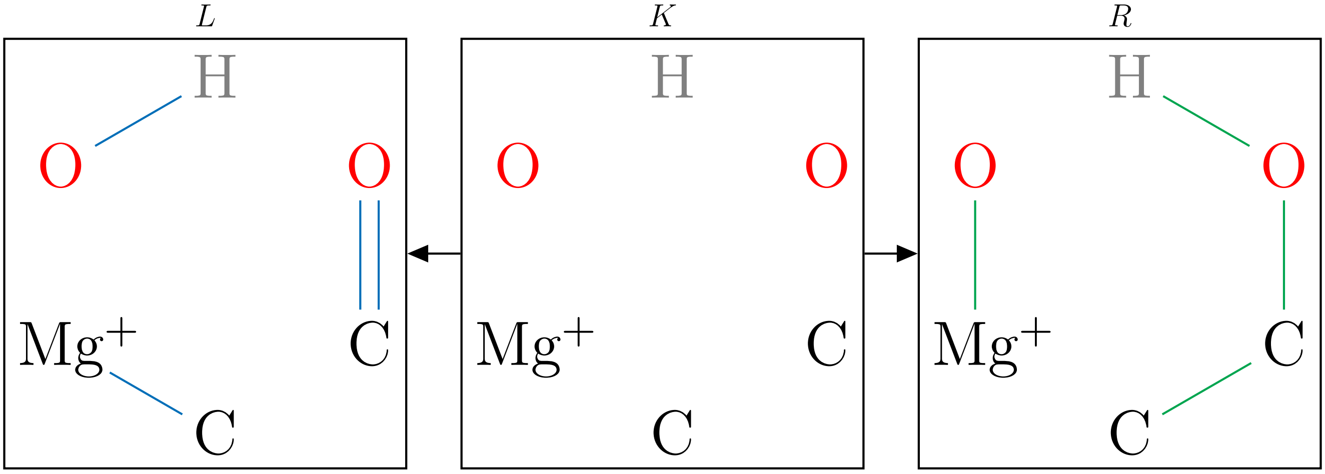

Files: out/643_r_128_11300100_{L, K, R}

0.0.130 129

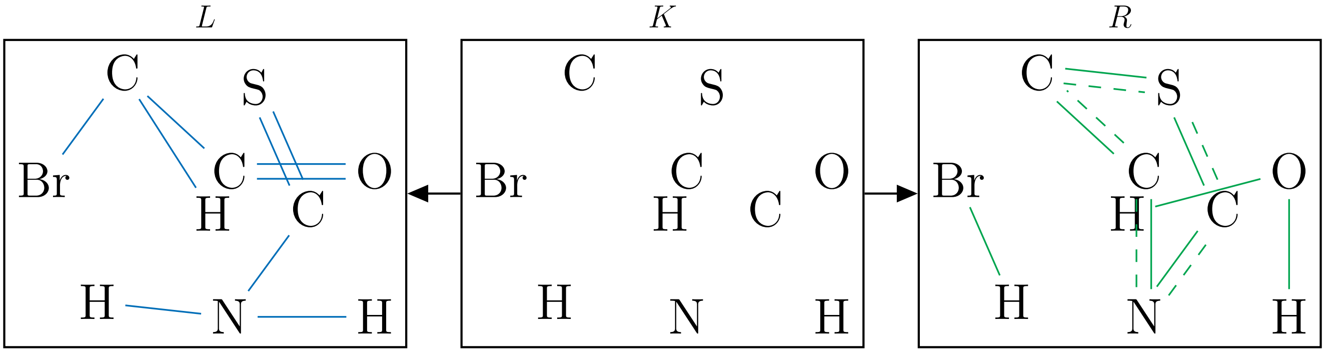

Files: out/646_r_129_10300000_{L, K, R}

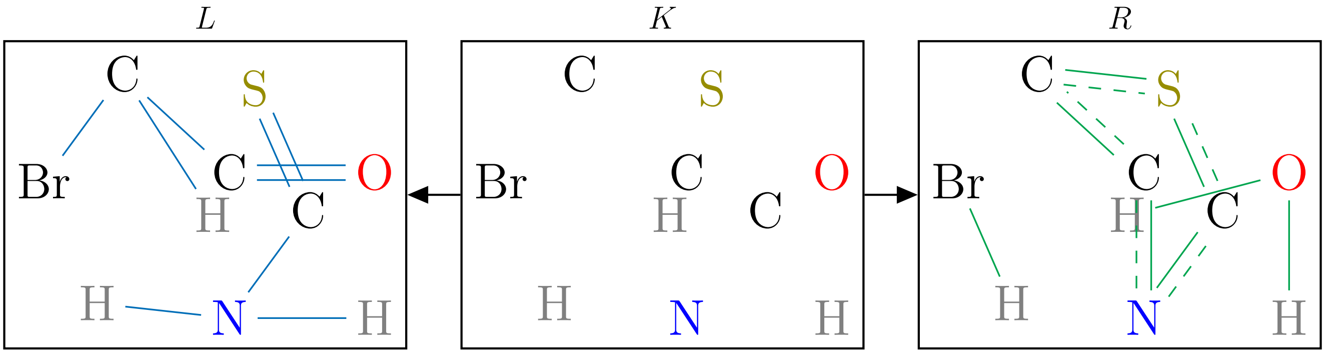

Files: out/648_r_129_11300100_{L, K, R}

0.0.131 130

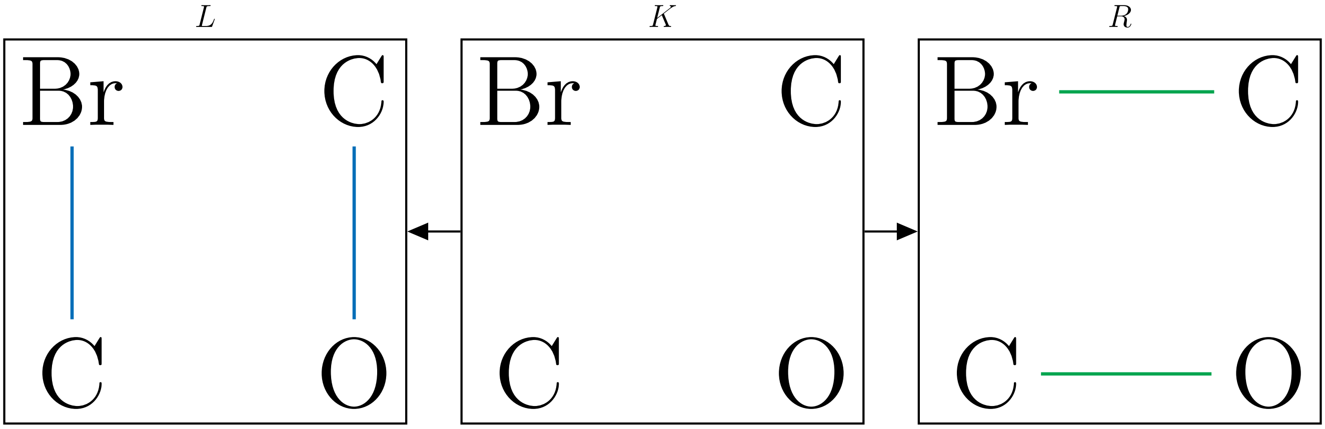

Files: out/651_r_130_10300000_{L, K, R}

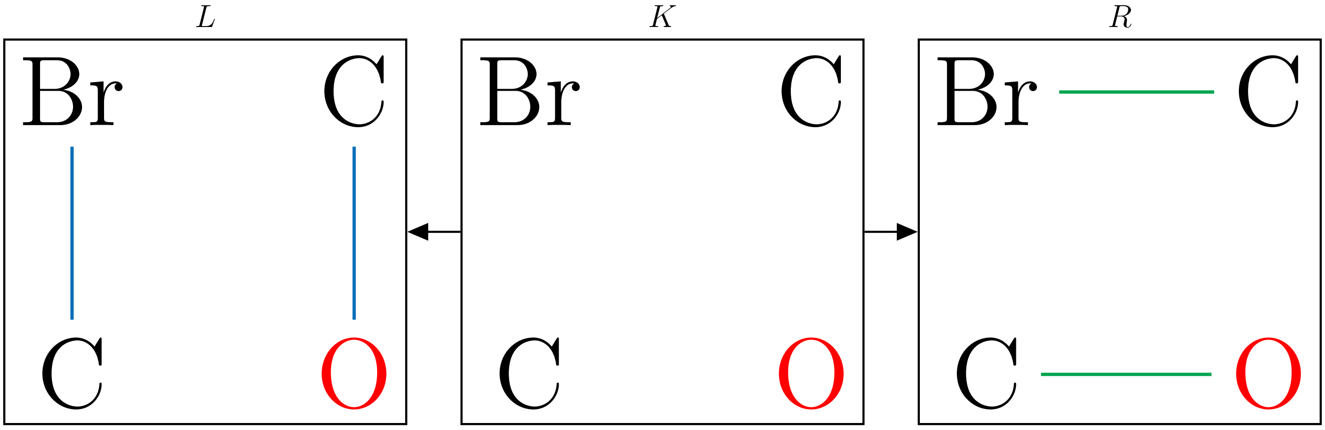

Files: out/653_r_130_11300100_{L, K, R}

0.0.132 131

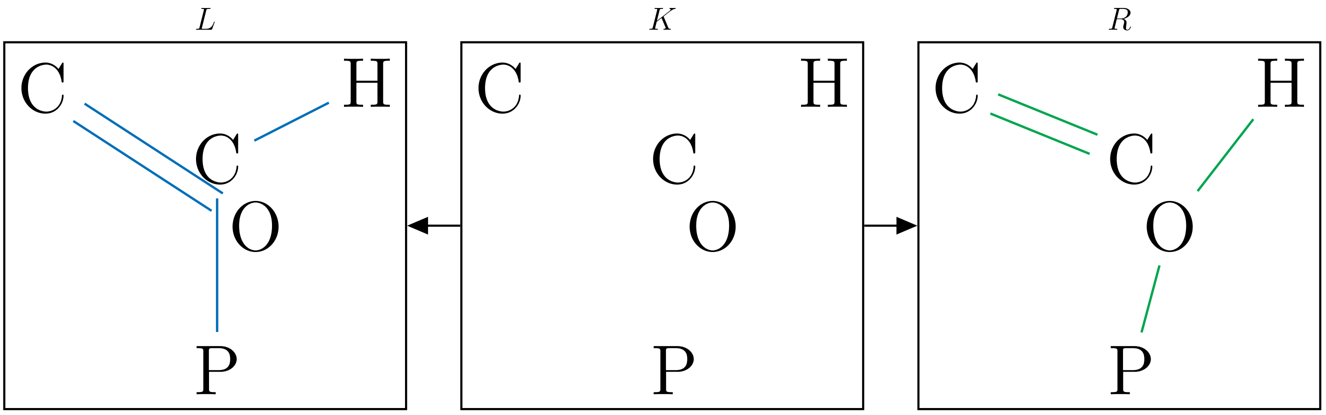

Files: out/656_r_131_10300000_{L, K, R}

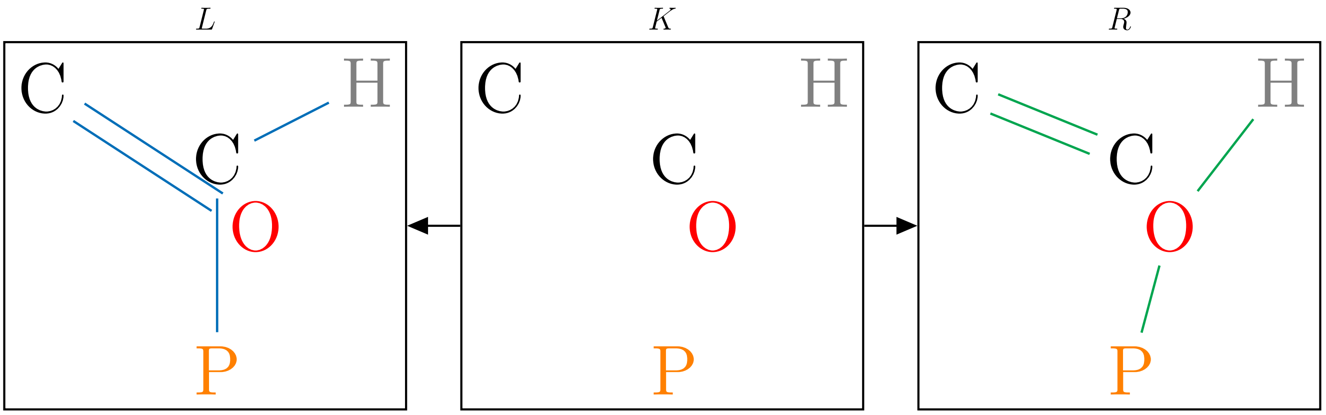

Files: out/658_r_131_11300100_{L, K, R}

0.0.133 132

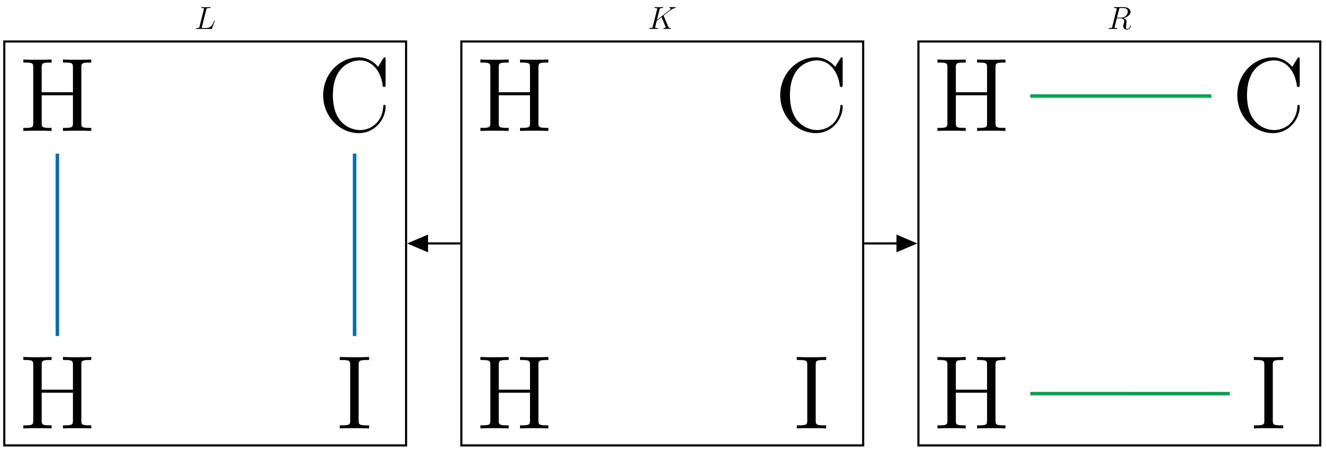

Files: out/661_r_132_10300000_{L, K, R}

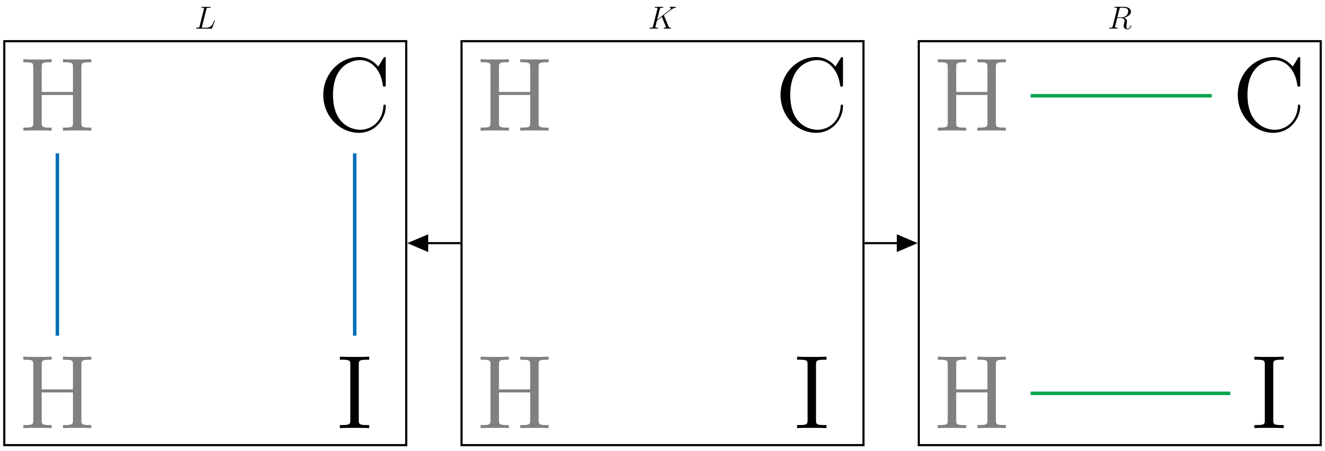

Files: out/663_r_132_11300100_{L, K, R}

0.0.134 133

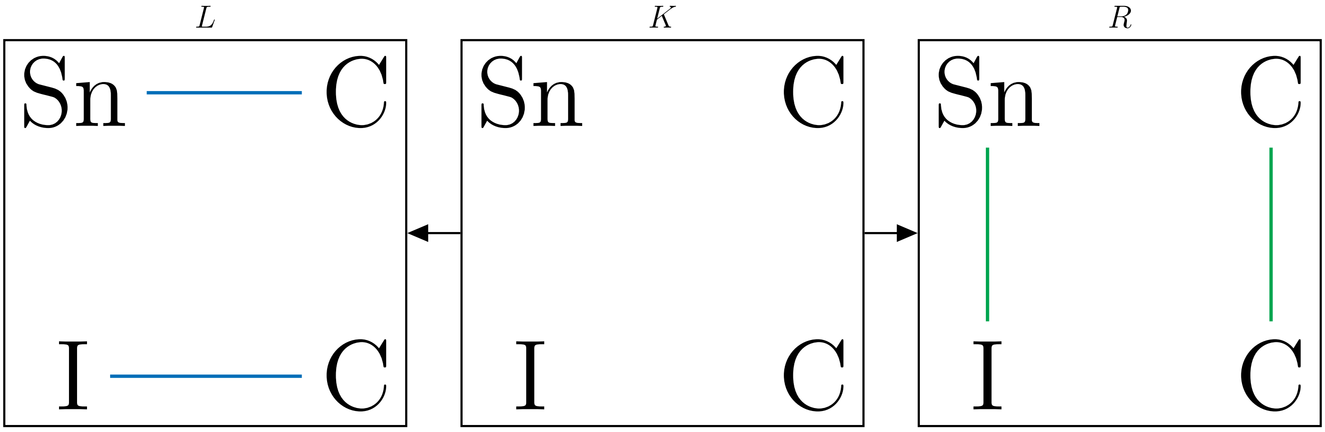

Files: out/666_r_133_10300000_{L, K, R}

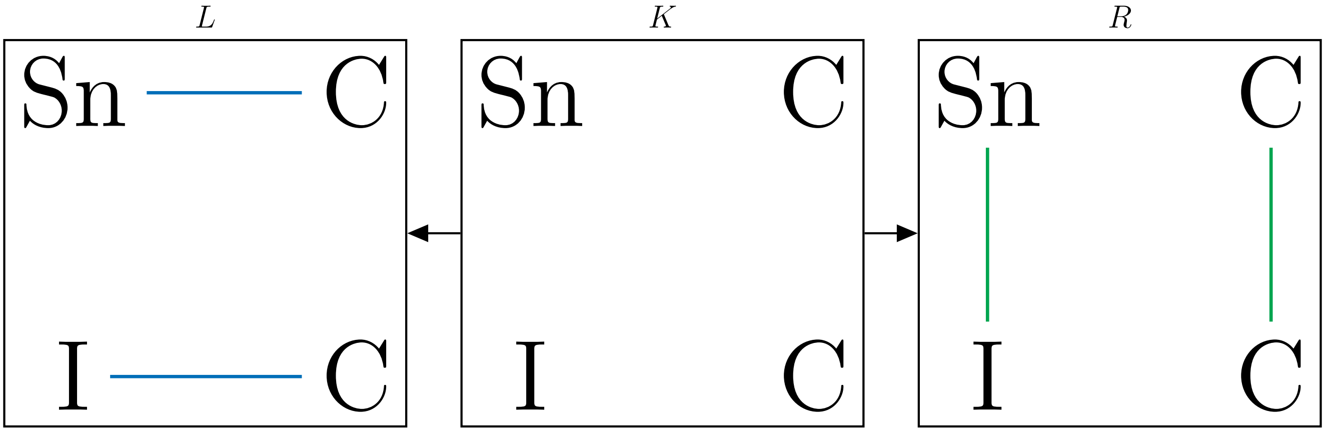

Files: out/668_r_133_11300100_{L, K, R}

0.0.135 134

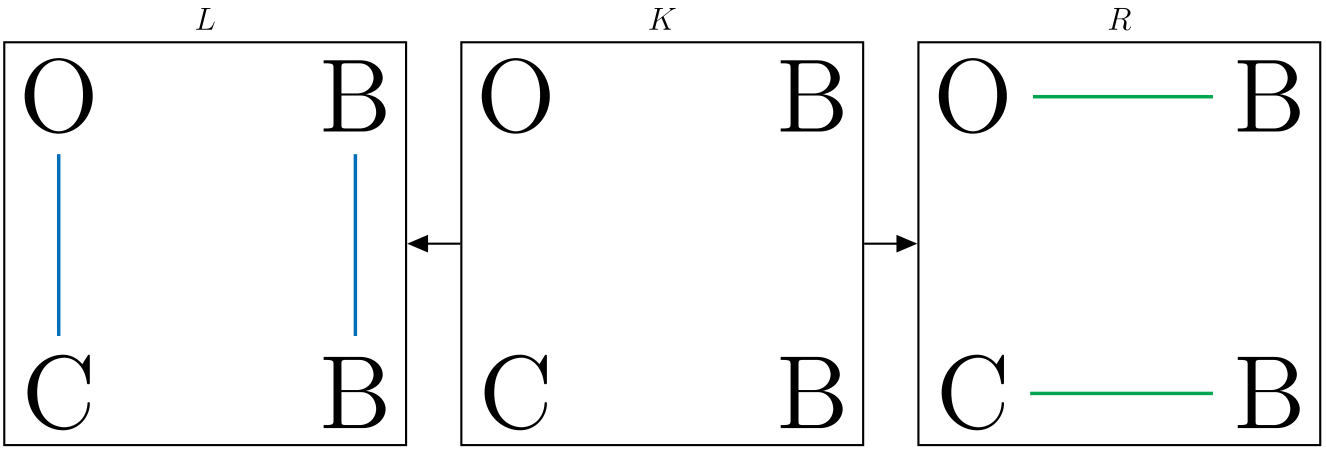

Files: out/671_r_134_10300000_{L, K, R}

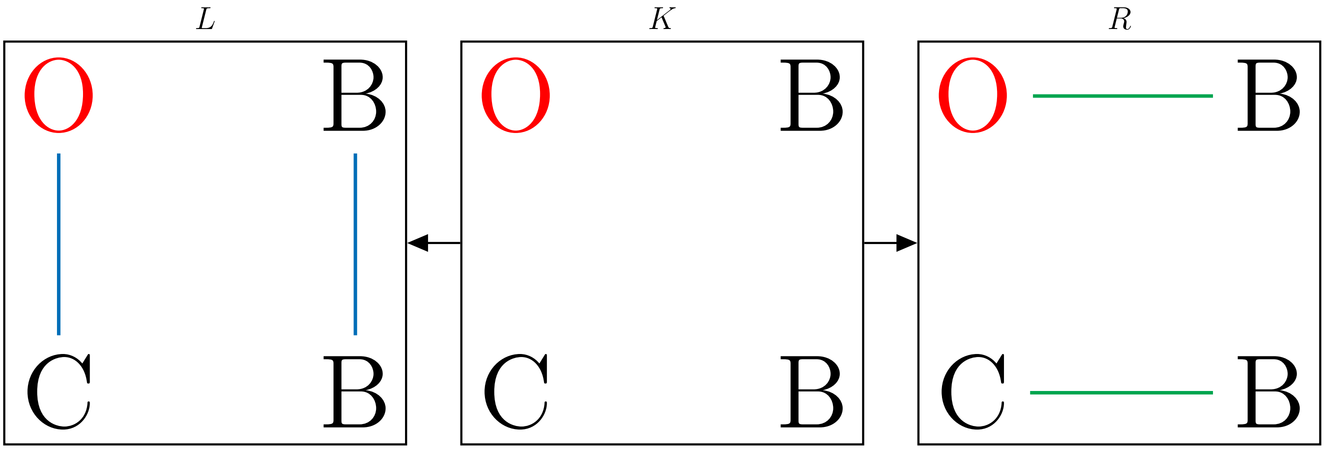

Files: out/673_r_134_11300100_{L, K, R}

0.0.136 135

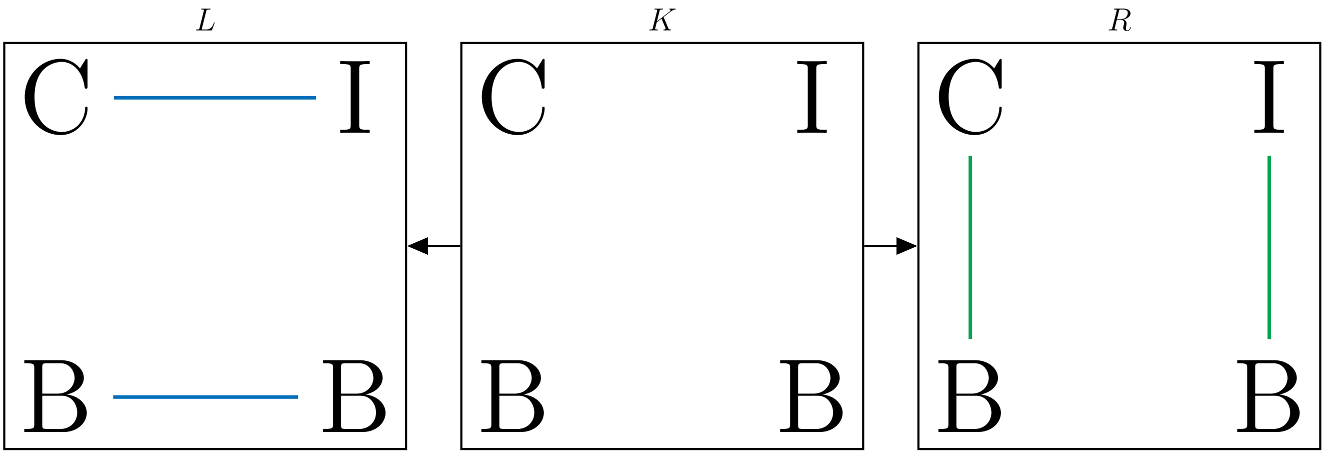

Files: out/676_r_135_10300000_{L, K, R}

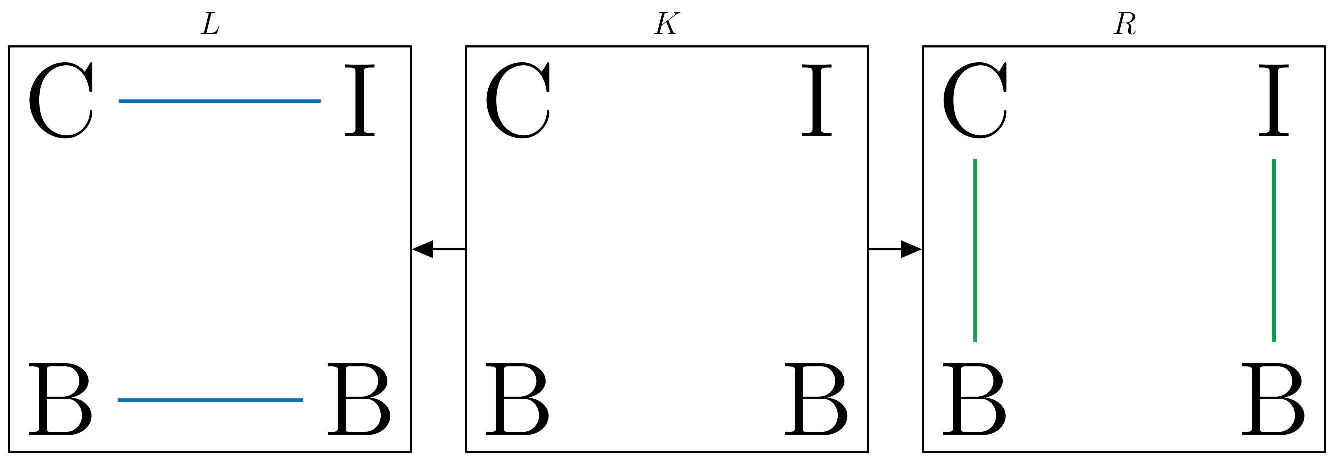

Files: out/678_r_135_11300100_{L, K, R}

0.0.137 136

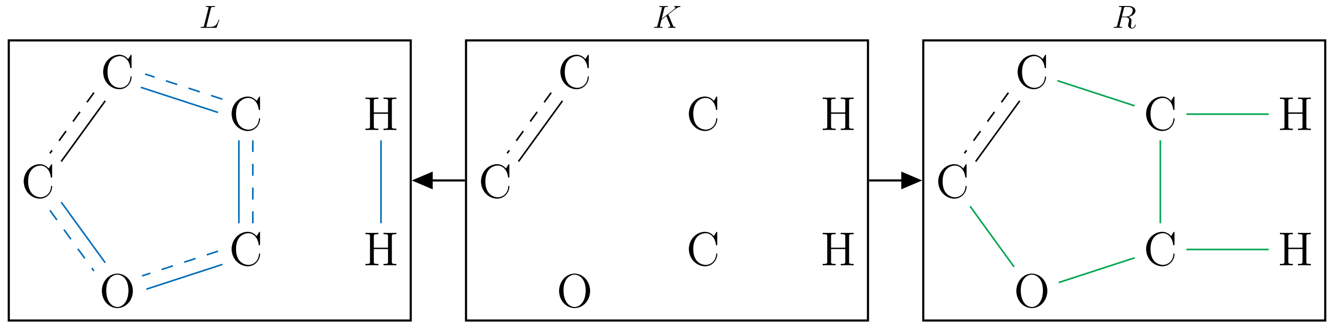

Files: out/681_r_136_10300000_{L, K, R}

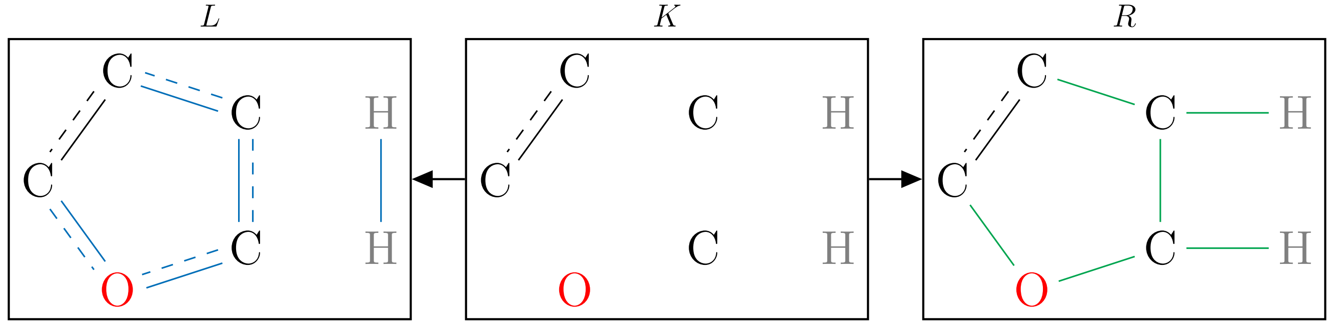

Files: out/683_r_136_11300100_{L, K, R}

0.0.138 137

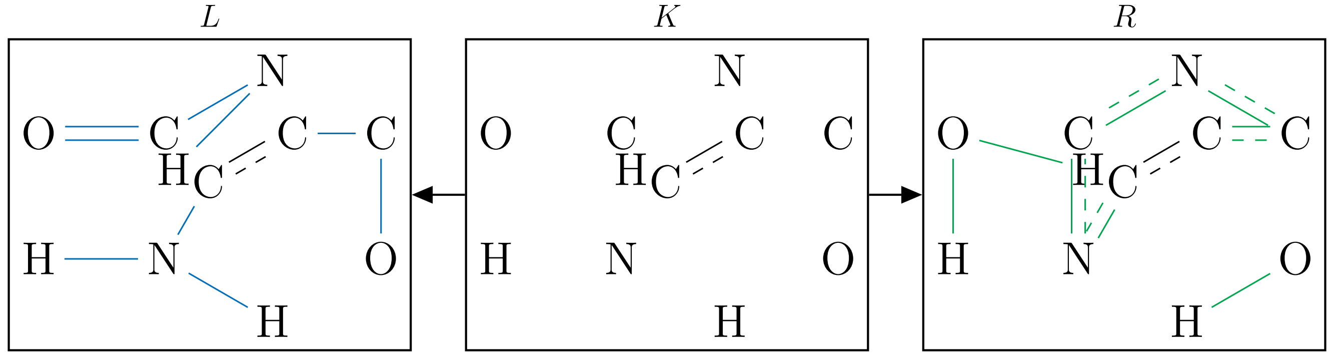

Files: out/686_r_137_10300000_{L, K, R}

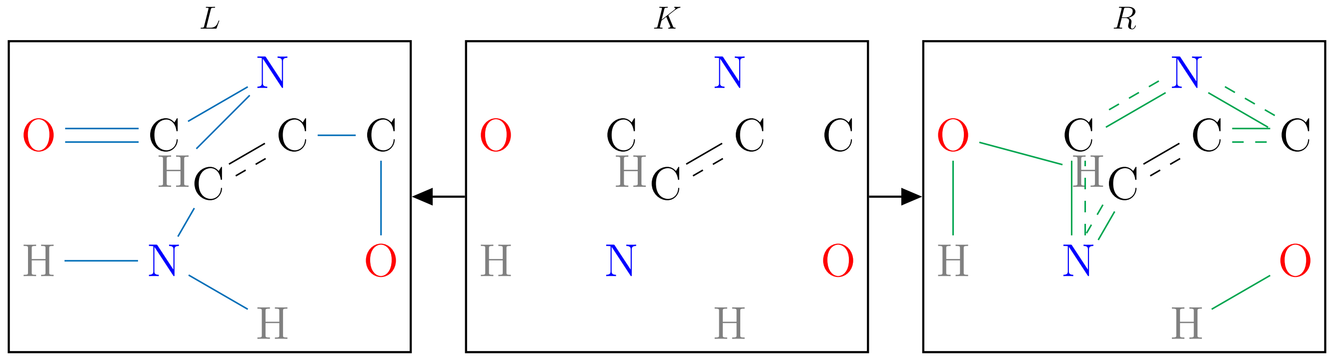

Files: out/688_r_137_11300100_{L, K, R}

0.0.139 138

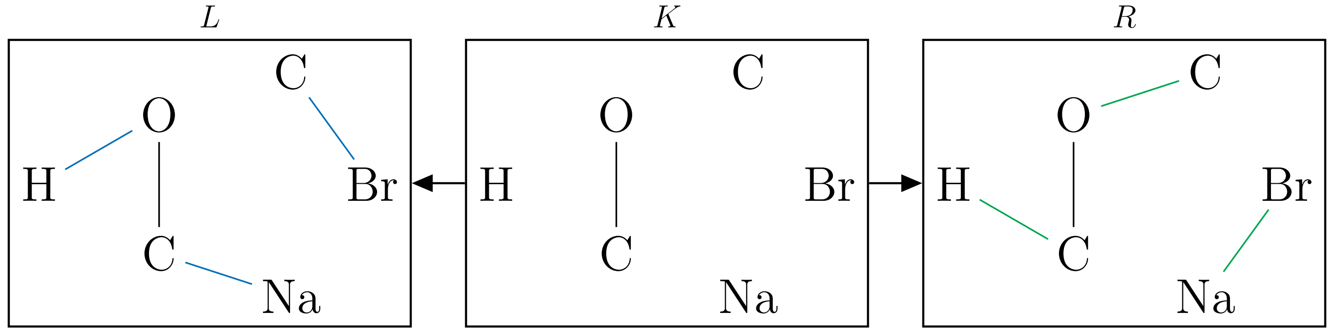

Files: out/691_r_138_10300000_{L, K, R}

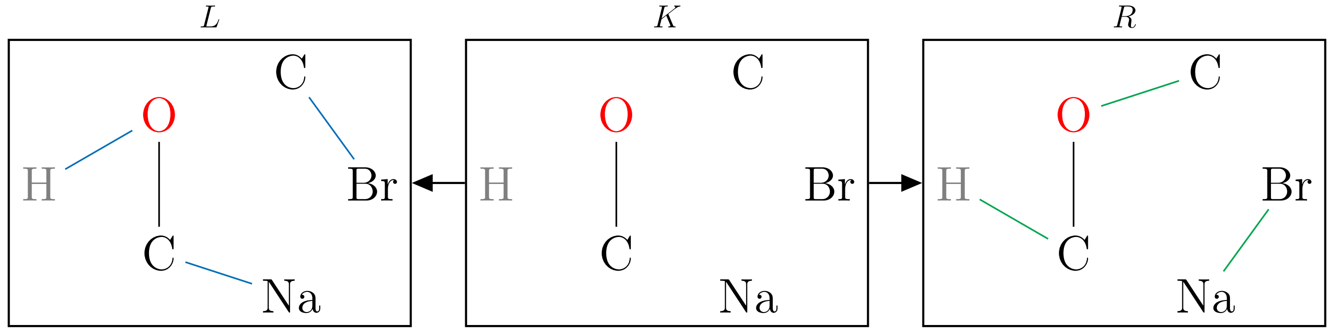

Files: out/693_r_138_11300100_{L, K, R}

0.0.140 139

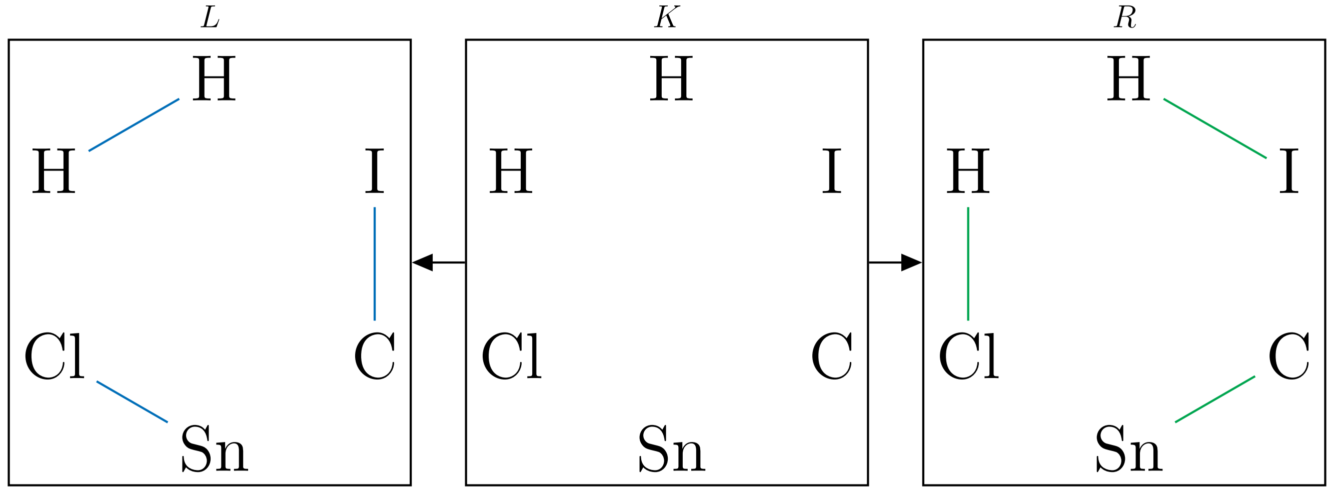

Files: out/696_r_139_10300000_{L, K, R}

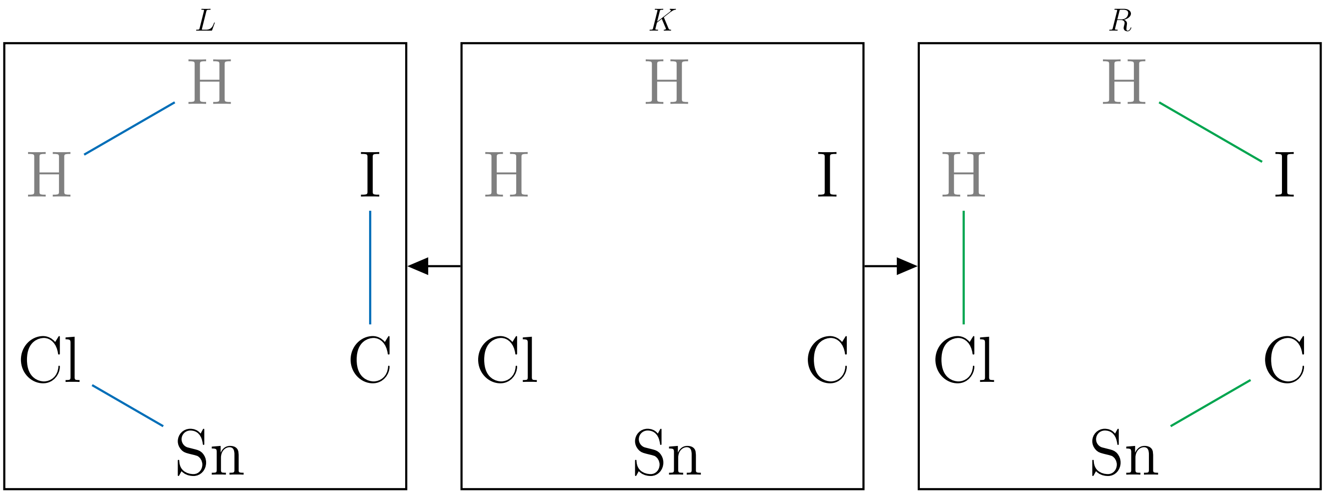

Files: out/698_r_139_11300100_{L, K, R}

0.0.141 140

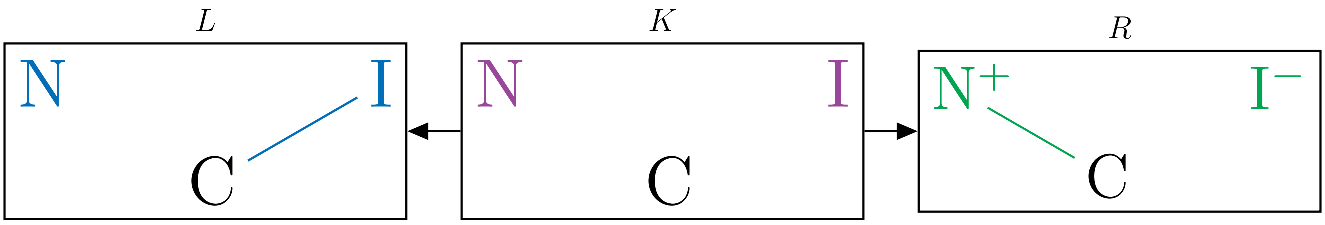

Files: out/701_r_140_10300000_{L, K, R}

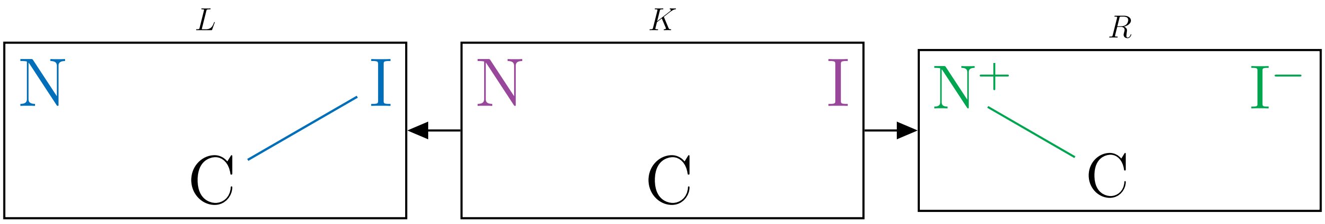

Files: out/703_r_140_11300100_{L, K, R}

0.0.142 141

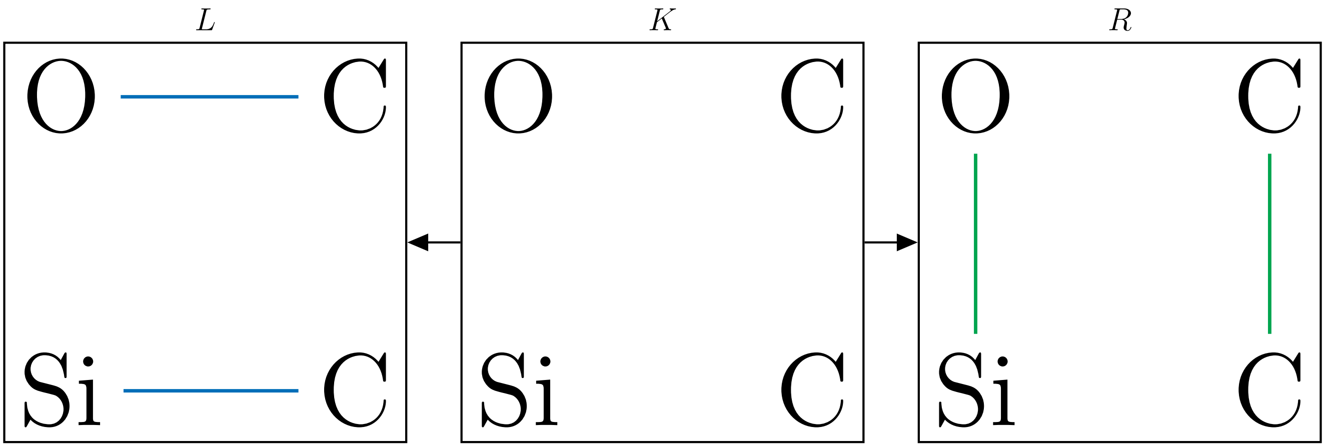

Files: out/706_r_141_10300000_{L, K, R}

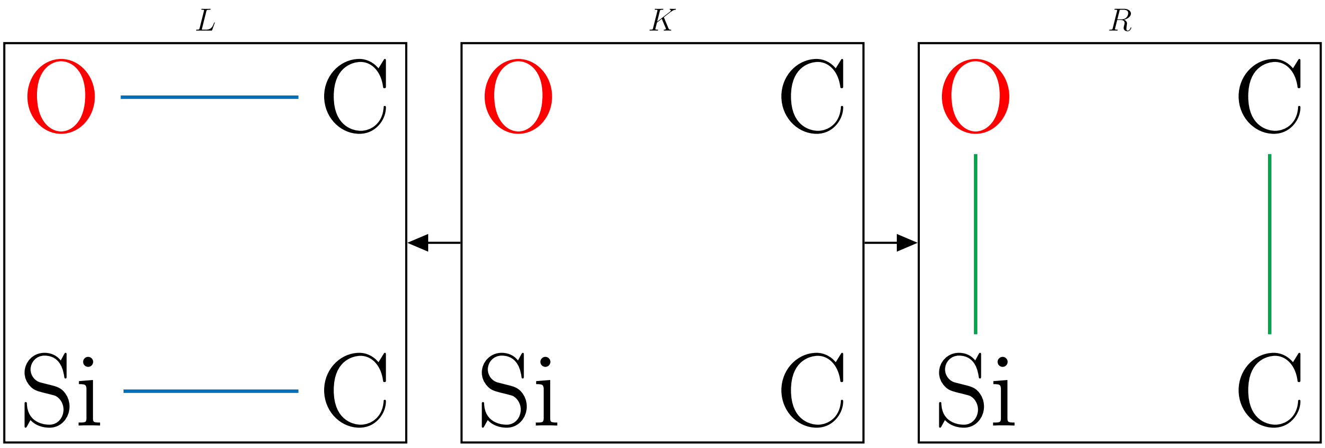

Files: out/708_r_141_11300100_{L, K, R}

0.0.143 142

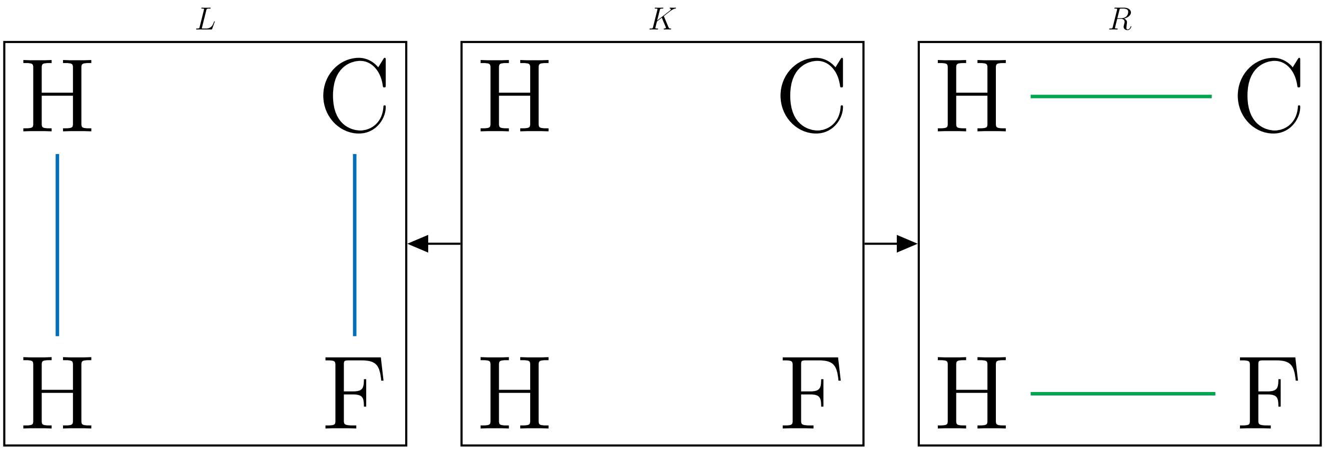

Files: out/711_r_142_10300000_{L, K, R}

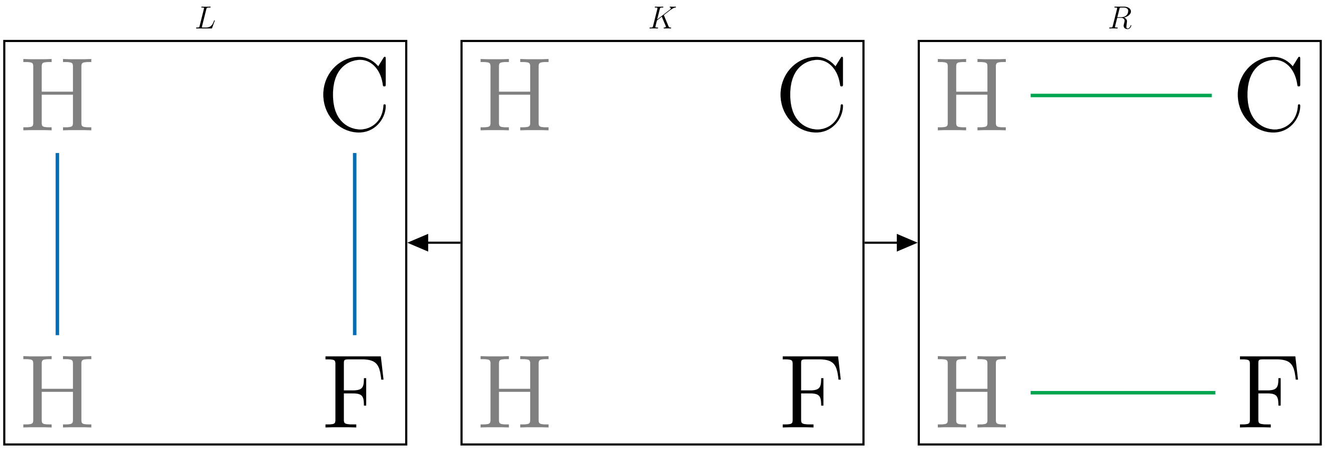

Files: out/713_r_142_11300100_{L, K, R}

0.0.144 143

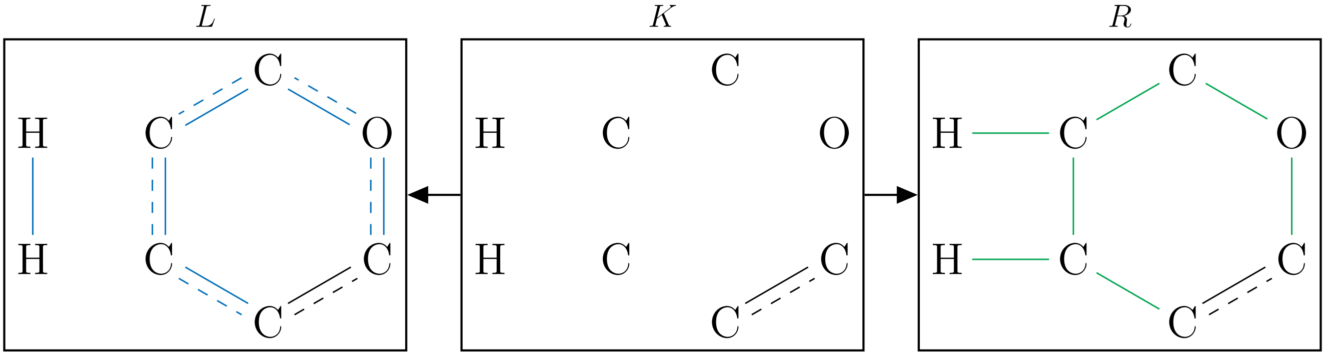

Files: out/716_r_143_10300000_{L, K, R}

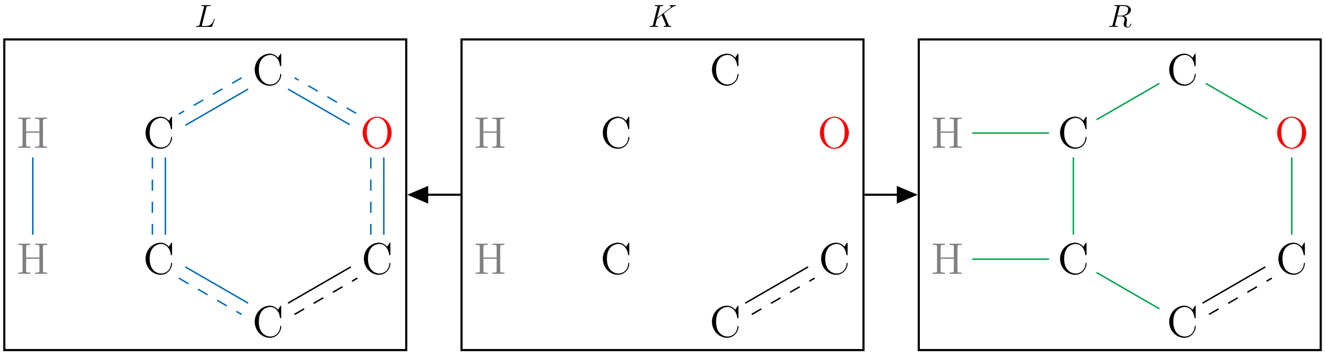

Files: out/718_r_143_11300100_{L, K, R}

0.0.145 144

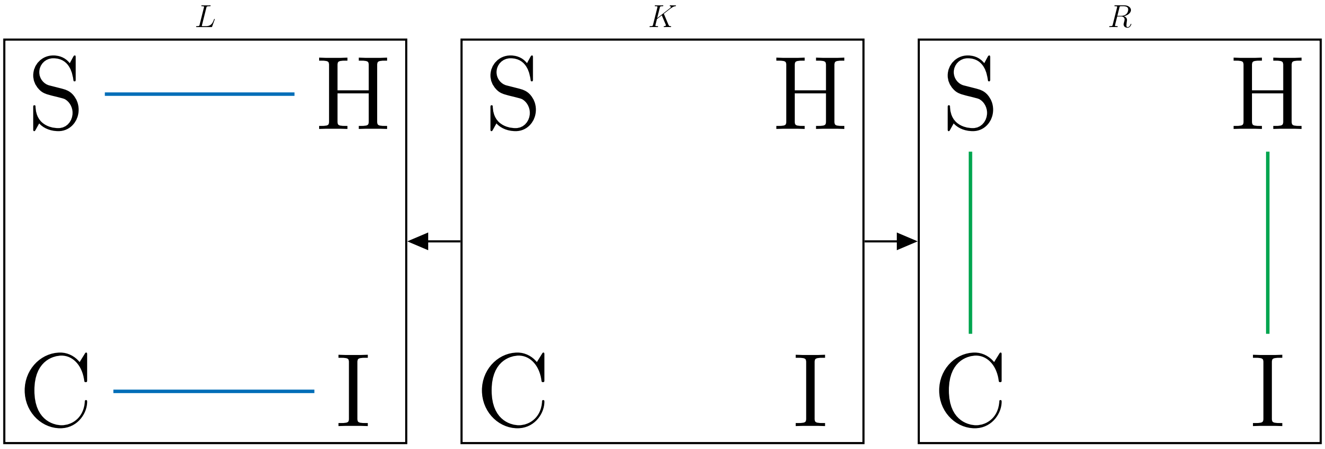

Files: out/721_r_144_10300000_{L, K, R}

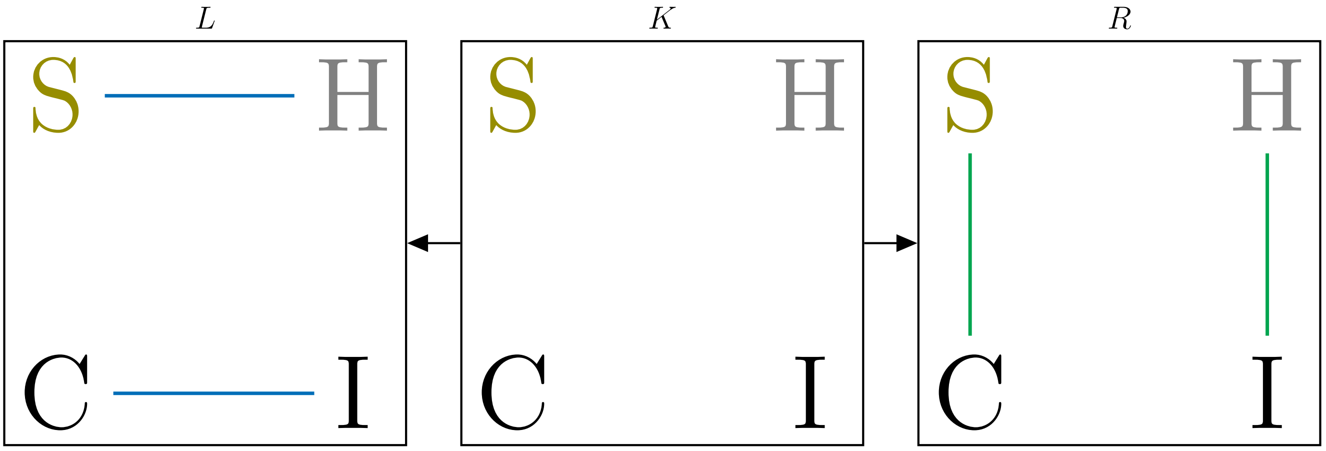

Files: out/723_r_144_11300100_{L, K, R}

0.0.146 145

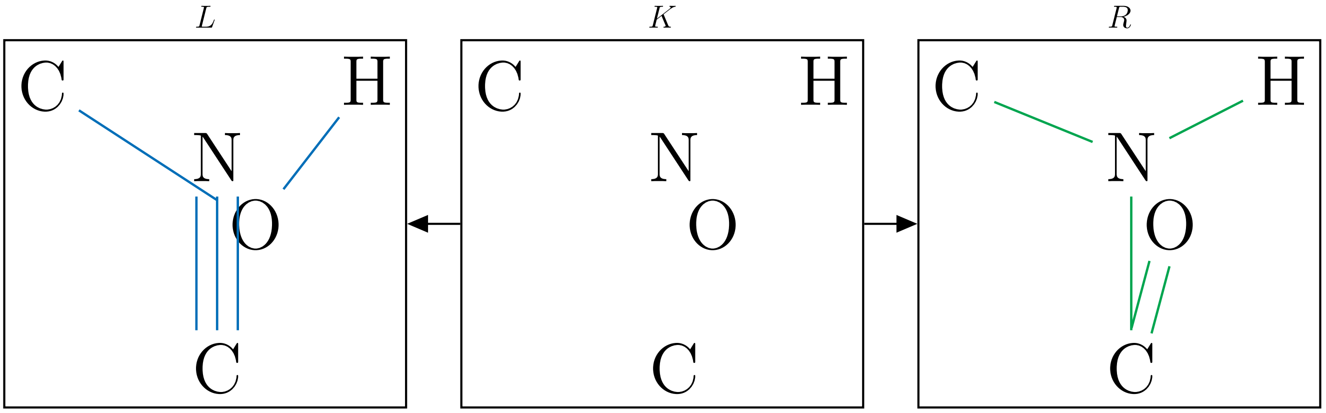

Files: out/726_r_145_10300000_{L, K, R}

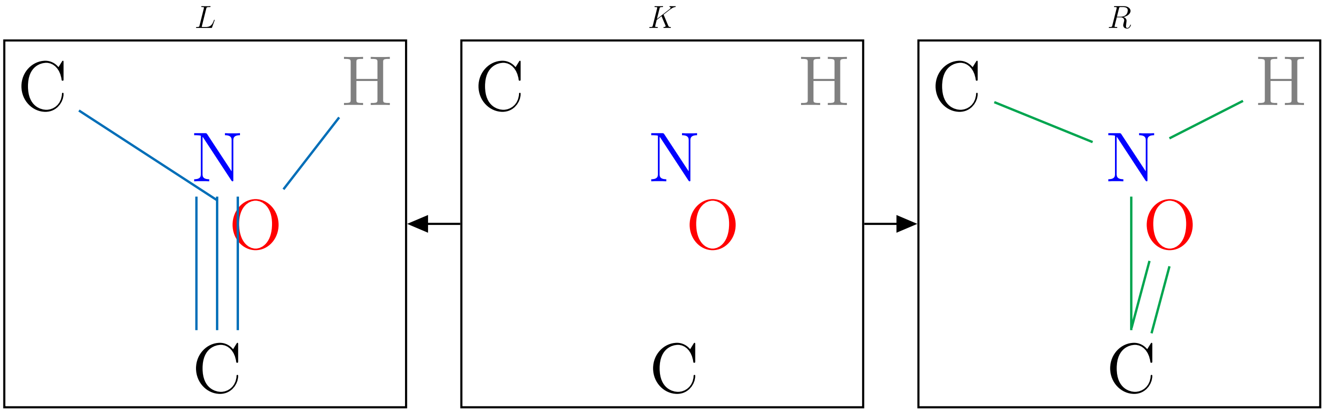

Files: out/728_r_145_11300100_{L, K, R}

0.0.147 146

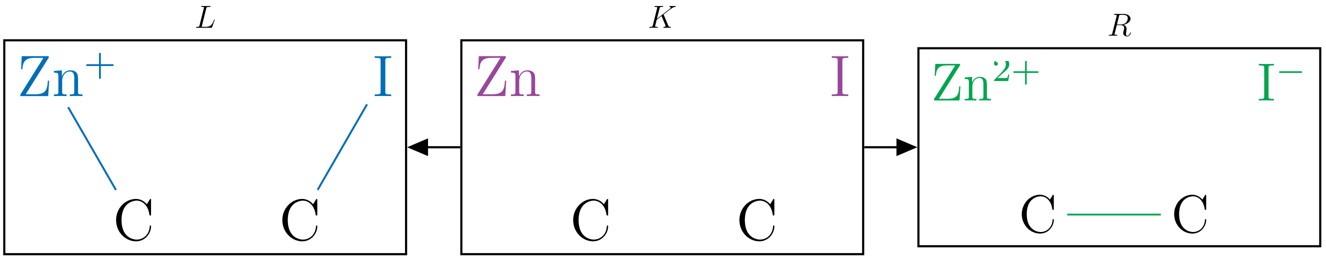

Files: out/731_r_146_10300000_{L, K, R}

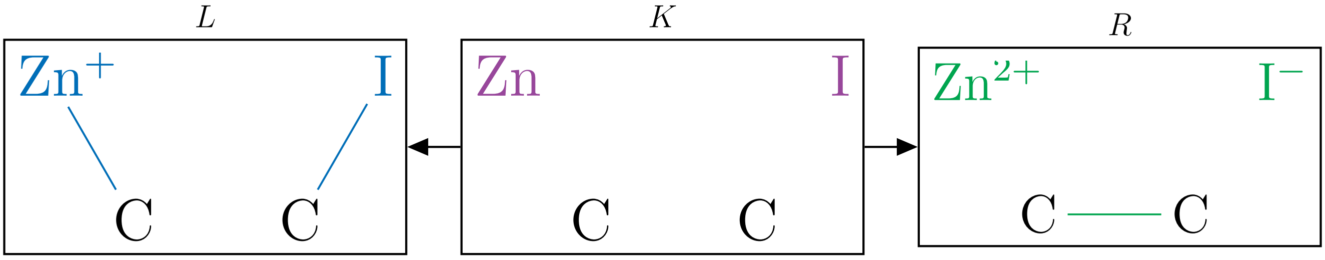

Files: out/733_r_146_11300100_{L, K, R}

0.0.148 147

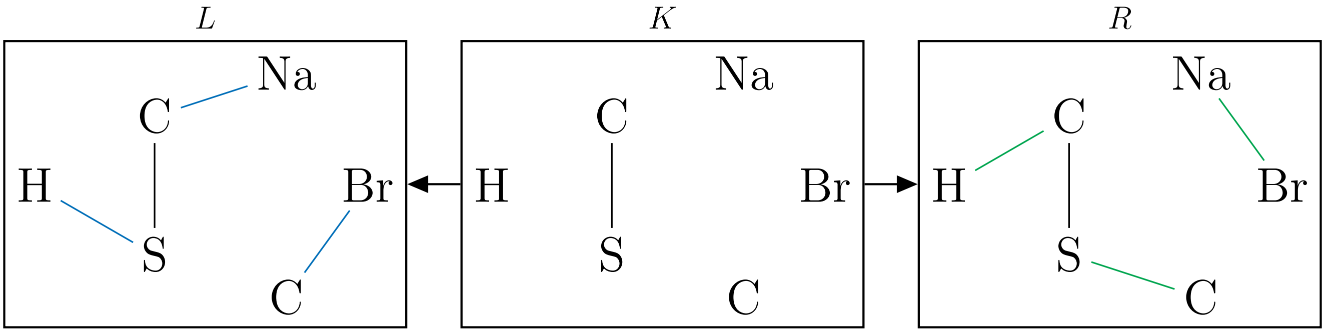

Files: out/736_r_147_10300000_{L, K, R}

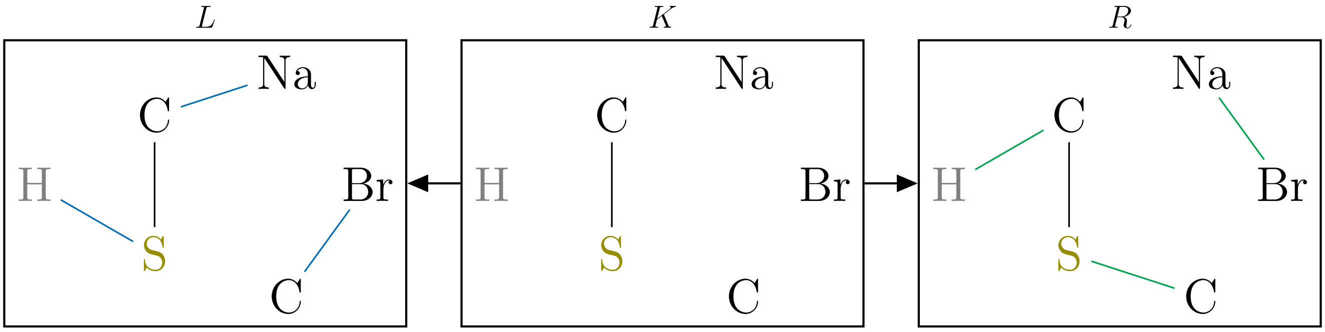

Files: out/738_r_147_11300100_{L, K, R}

0.0.149 148

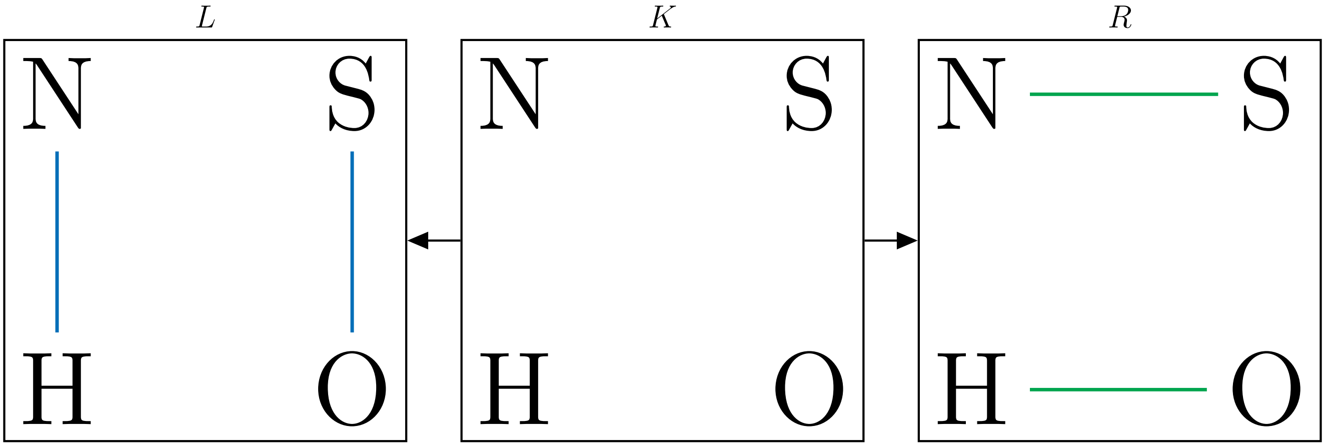

Files: out/741_r_148_10300000_{L, K, R}

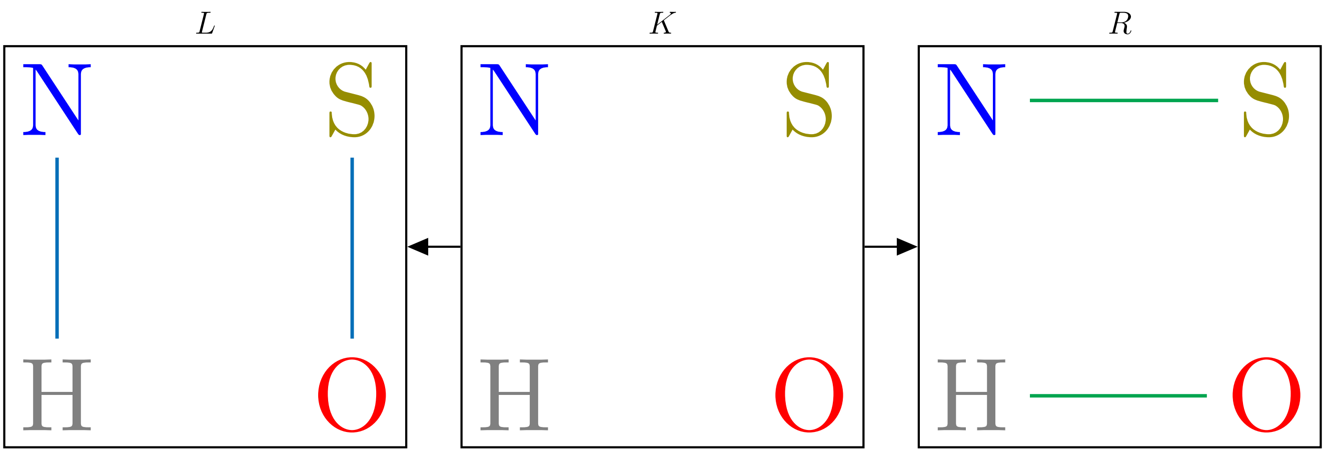

Files: out/743_r_148_11300100_{L, K, R}

0.0.150 149

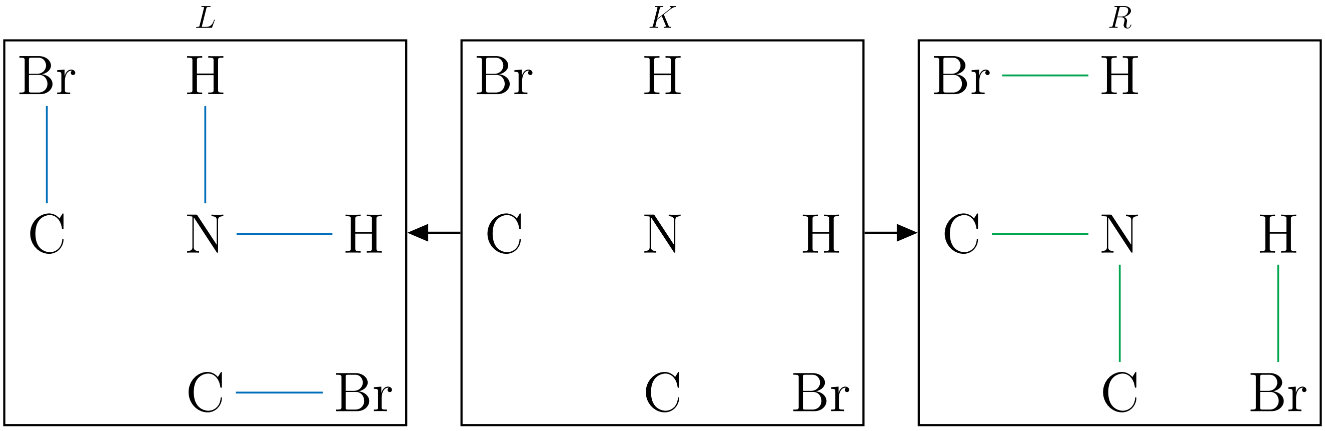

Files: out/746_r_149_10300000_{L, K, R}

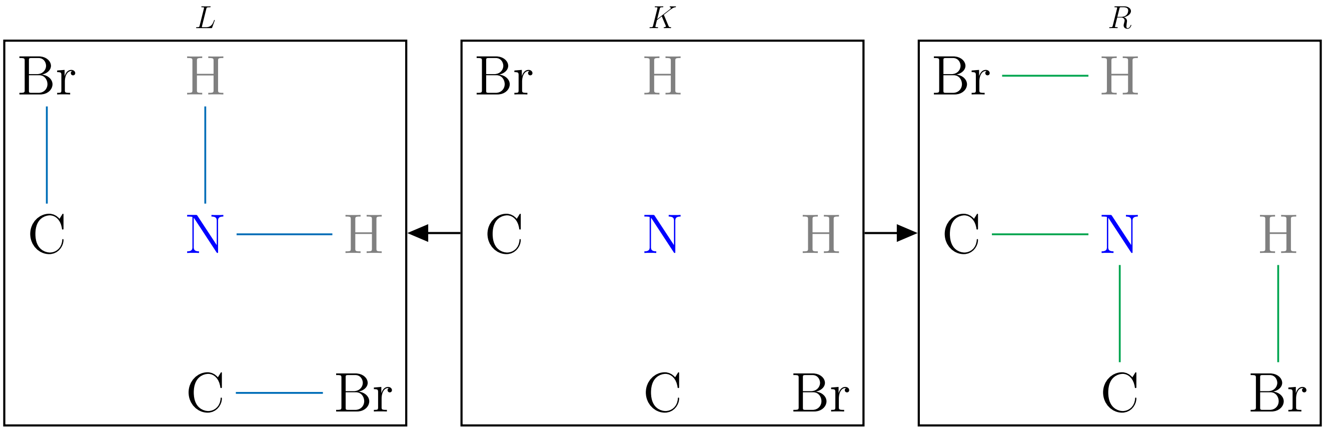

Files: out/748_r_149_11300100_{L, K, R}

0.0.151 150

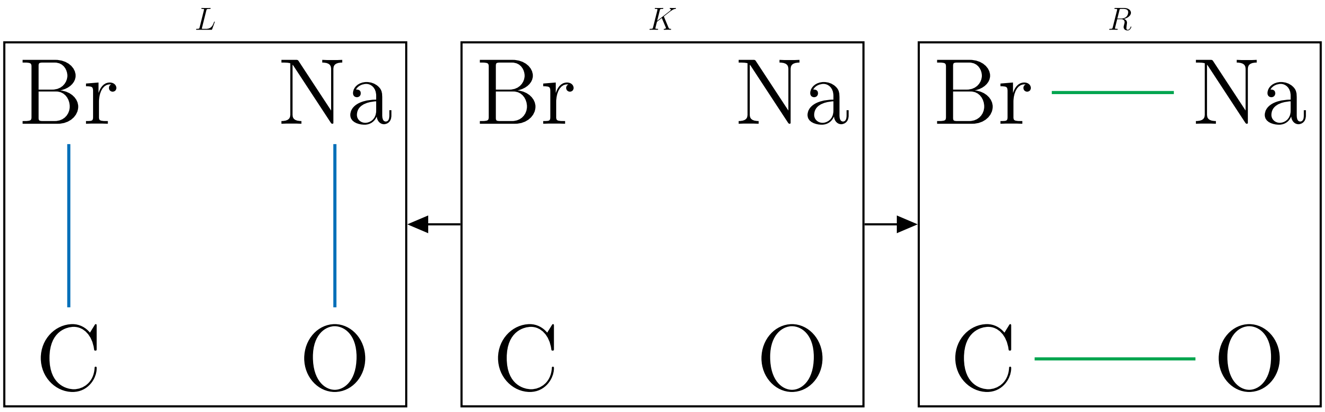

Files: out/751_r_150_10300000_{L, K, R}

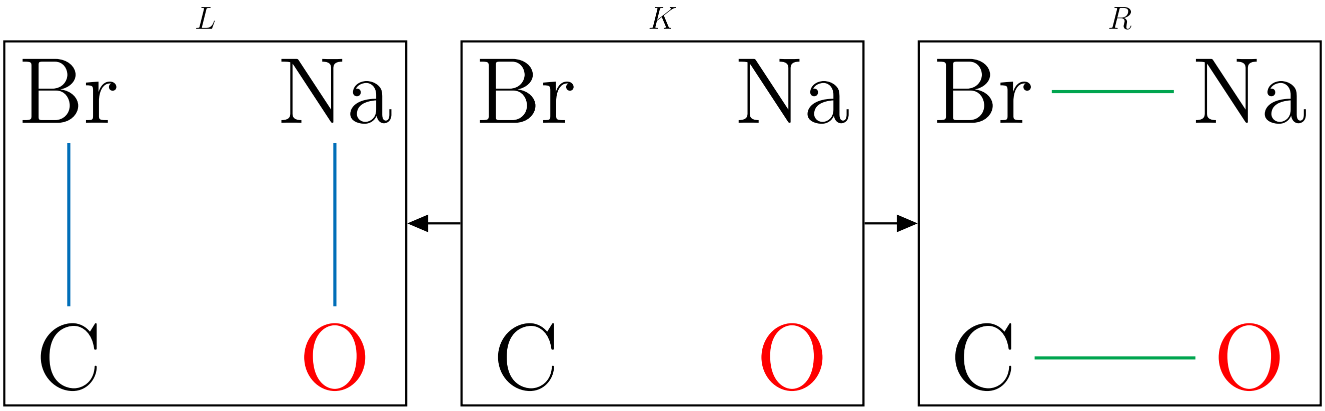

Files: out/753_r_150_11300100_{L, K, R}

0.0.152 151

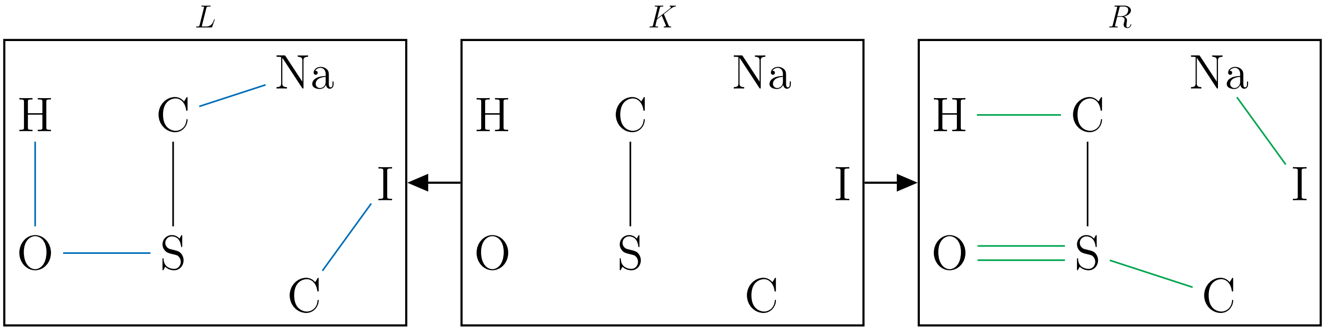

Files: out/756_r_151_10300000_{L, K, R}

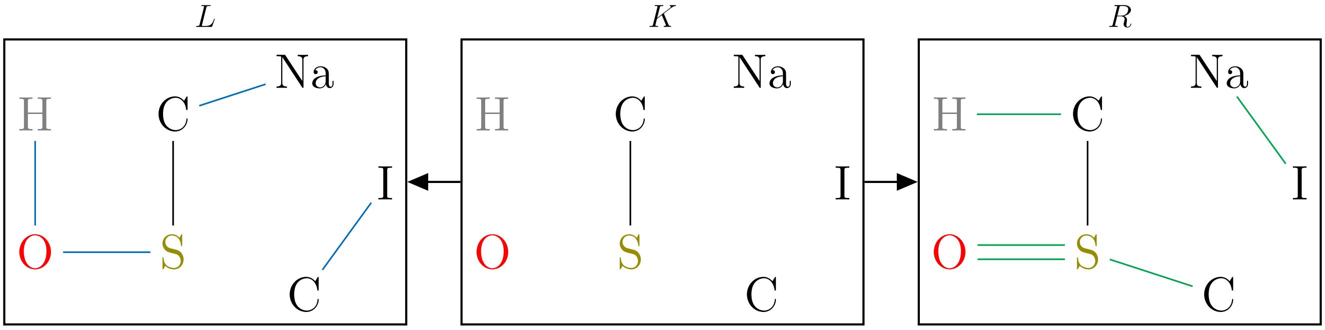

Files: out/758_r_151_11300100_{L, K, R}

0.0.153 152

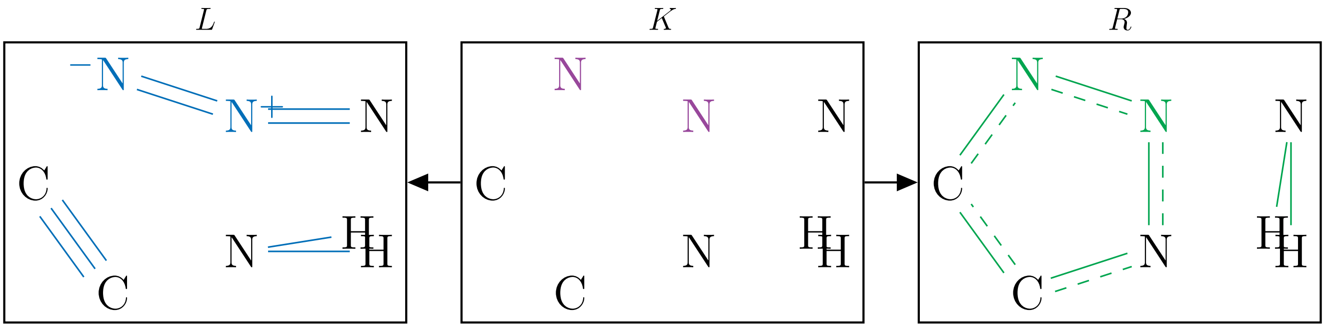

Files: out/761_r_152_10300000_{L, K, R}

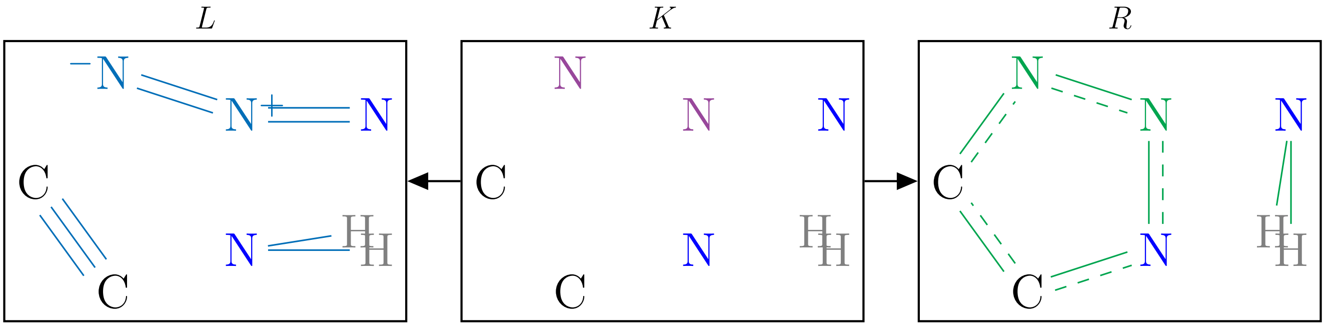

Files: out/763_r_152_11300100_{L, K, R}

0.0.154 153

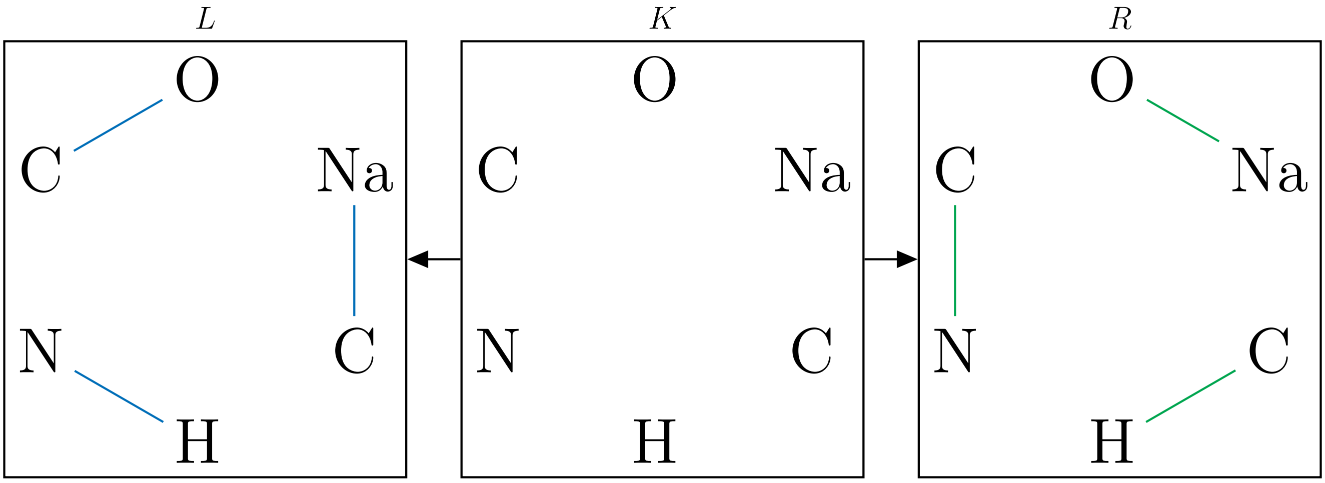

Files: out/766_r_153_10300000_{L, K, R}

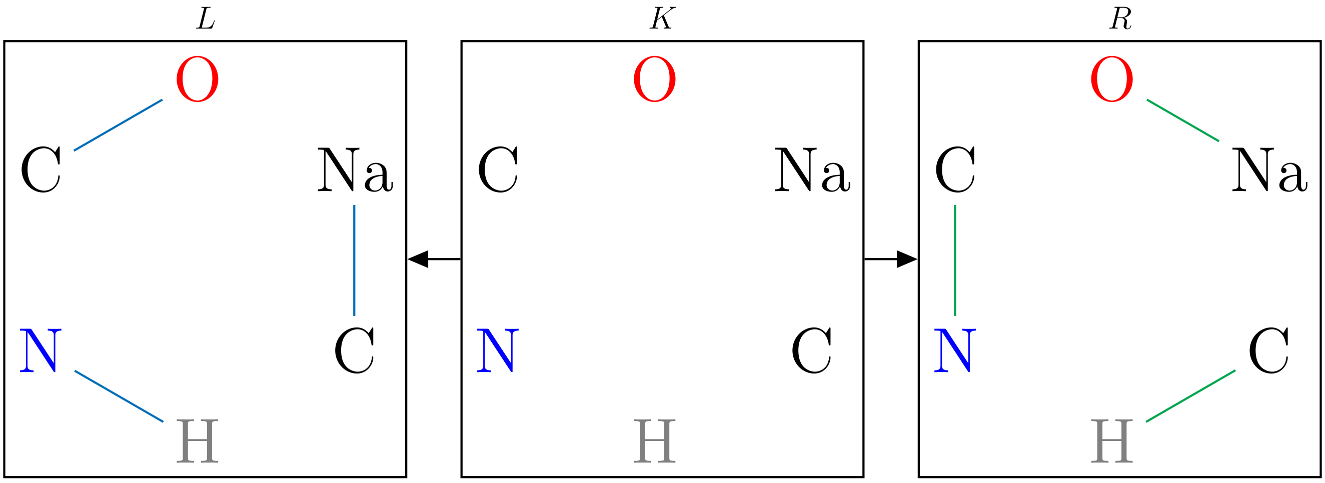

Files: out/768_r_153_11300100_{L, K, R}

0.0.155 154

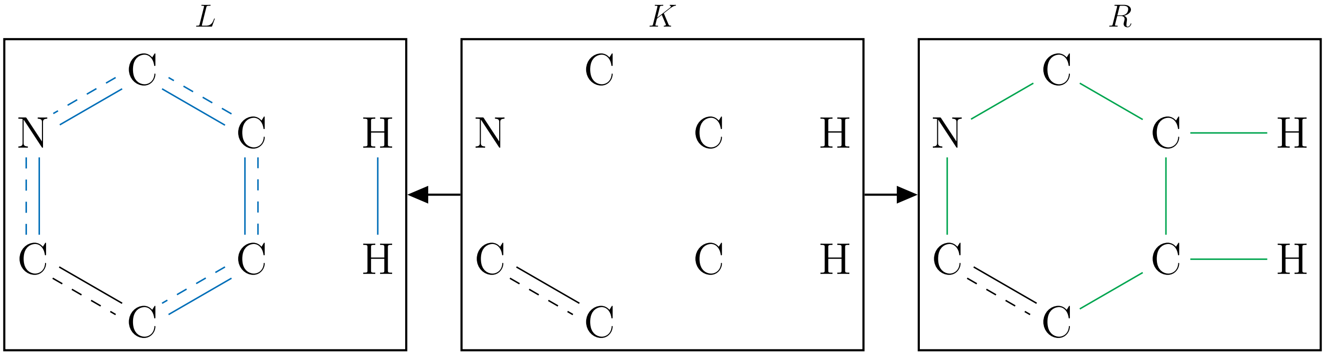

Files: out/771_r_154_10300000_{L, K, R}

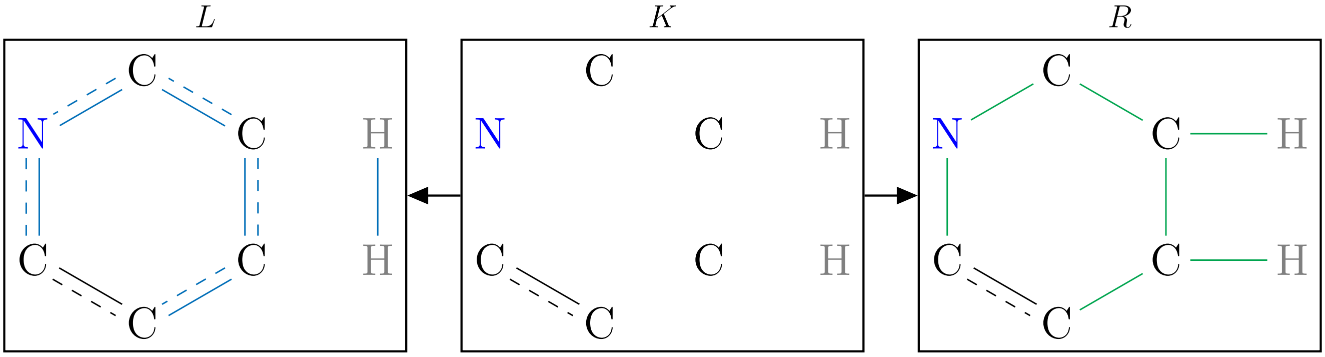

Files: out/773_r_154_11300100_{L, K, R}

0.0.156 155

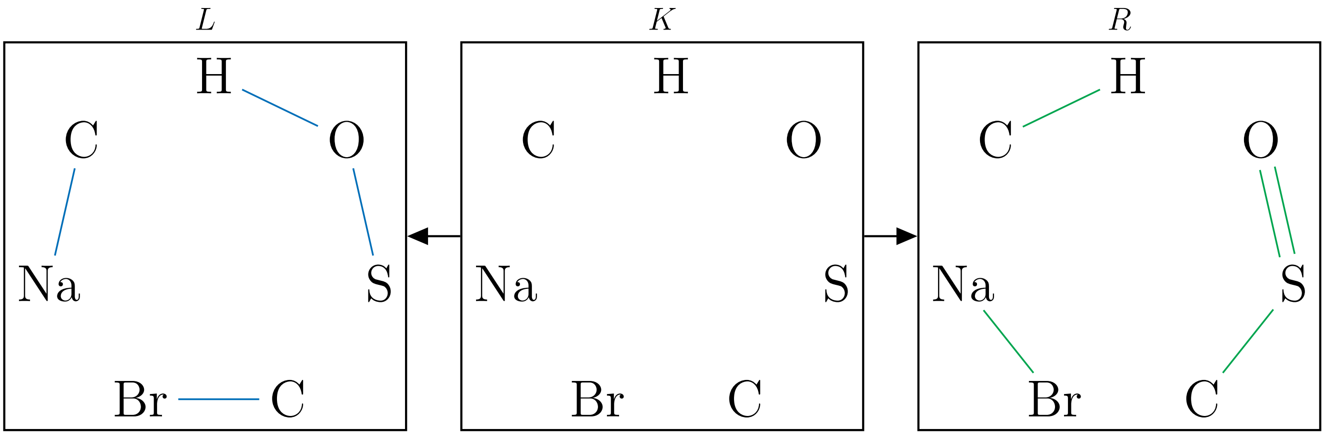

Files: out/776_r_155_10300000_{L, K, R}

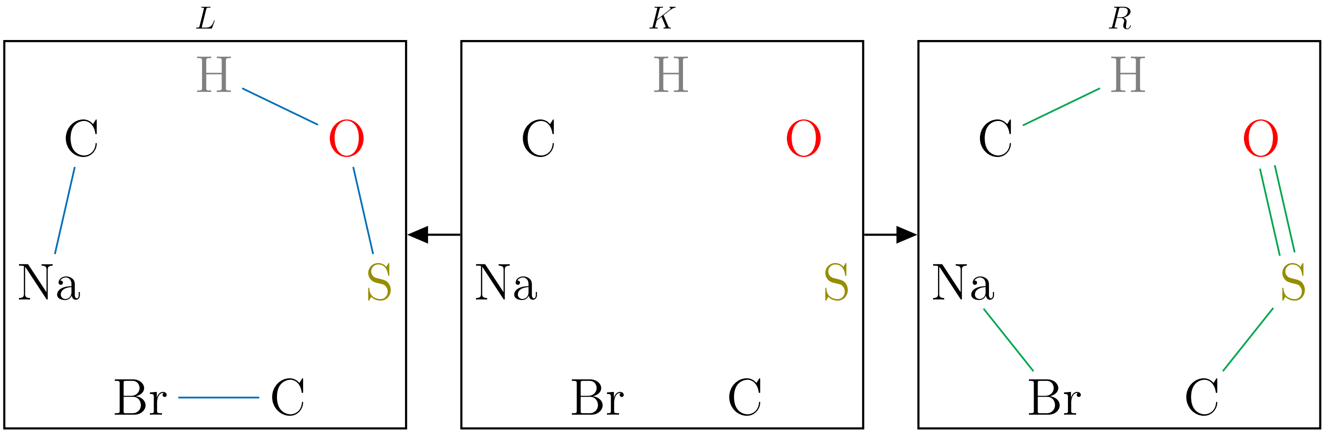

Files: out/778_r_155_11300100_{L, K, R}

0.0.157 156

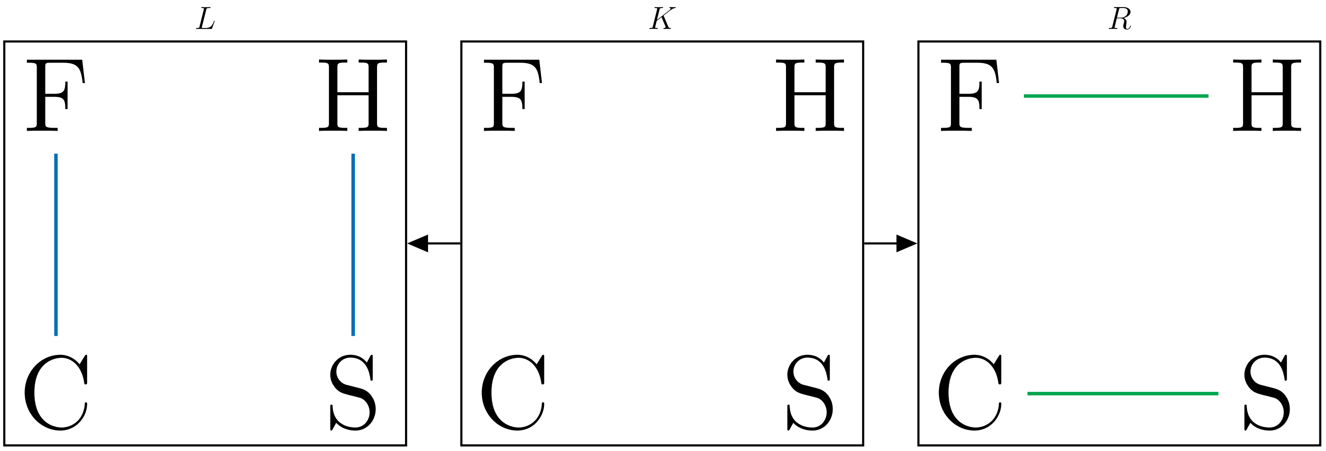

Files: out/781_r_156_10300000_{L, K, R}

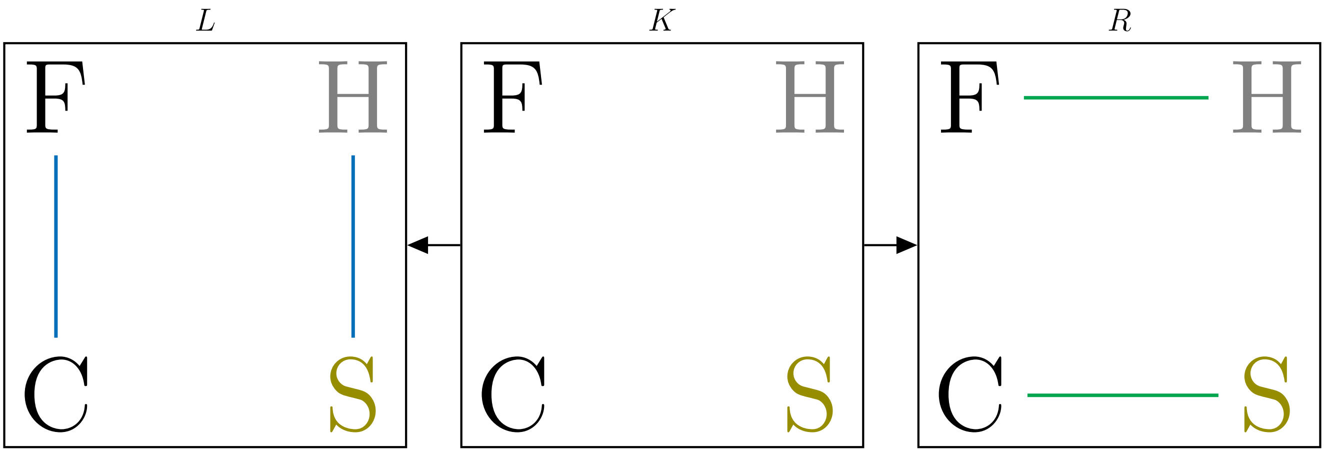

Files: out/783_r_156_11300100_{L, K, R}

0.0.158 157

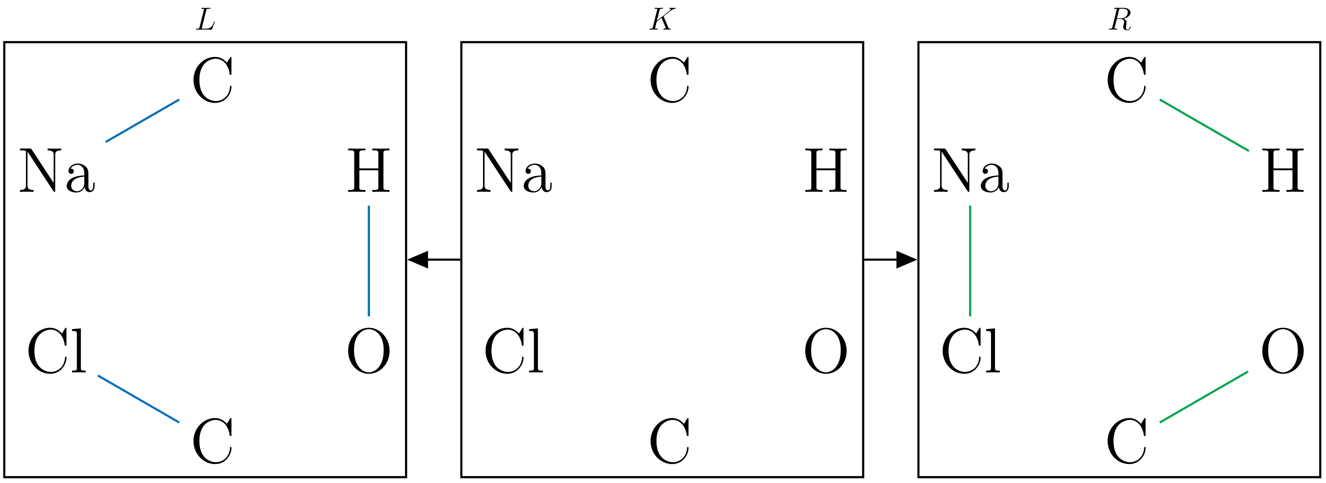

Files: out/786_r_157_10300000_{L, K, R}

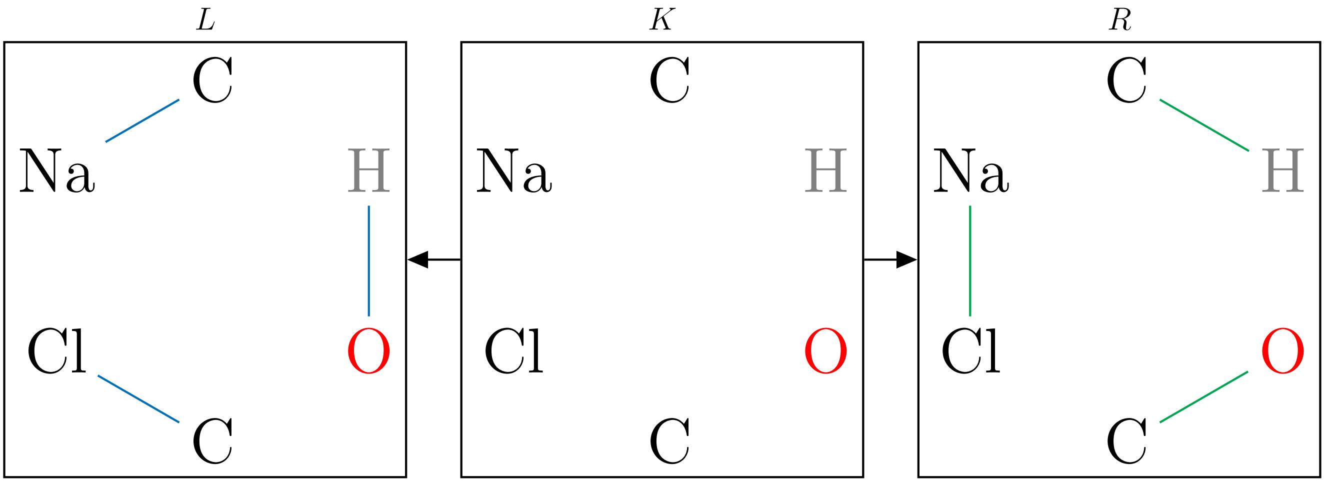

Files: out/788_r_157_11300100_{L, K, R}

0.0.159 158

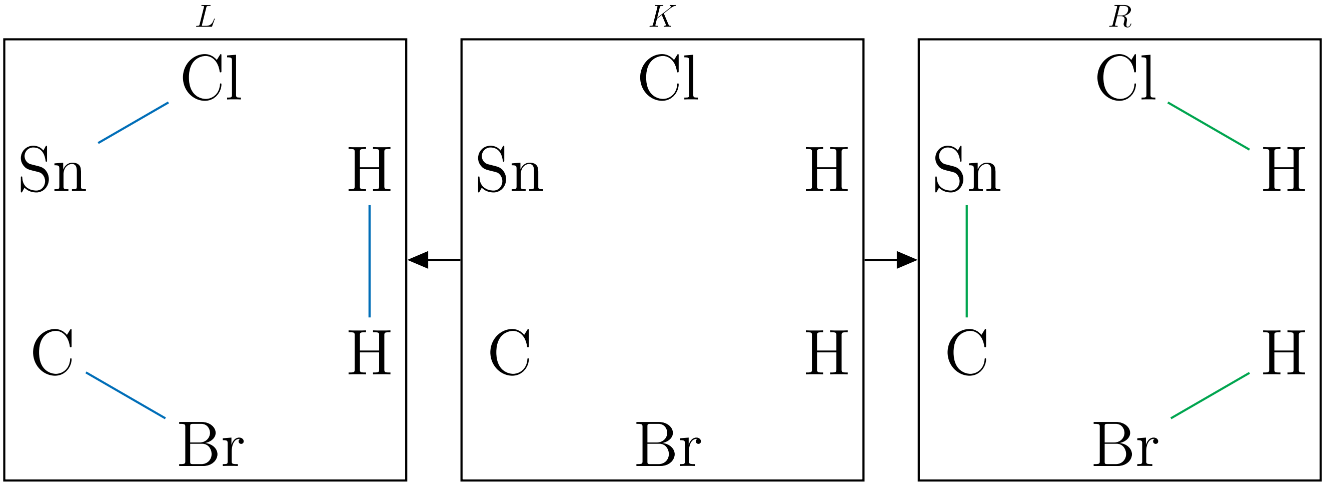

Files: out/791_r_158_10300000_{L, K, R}

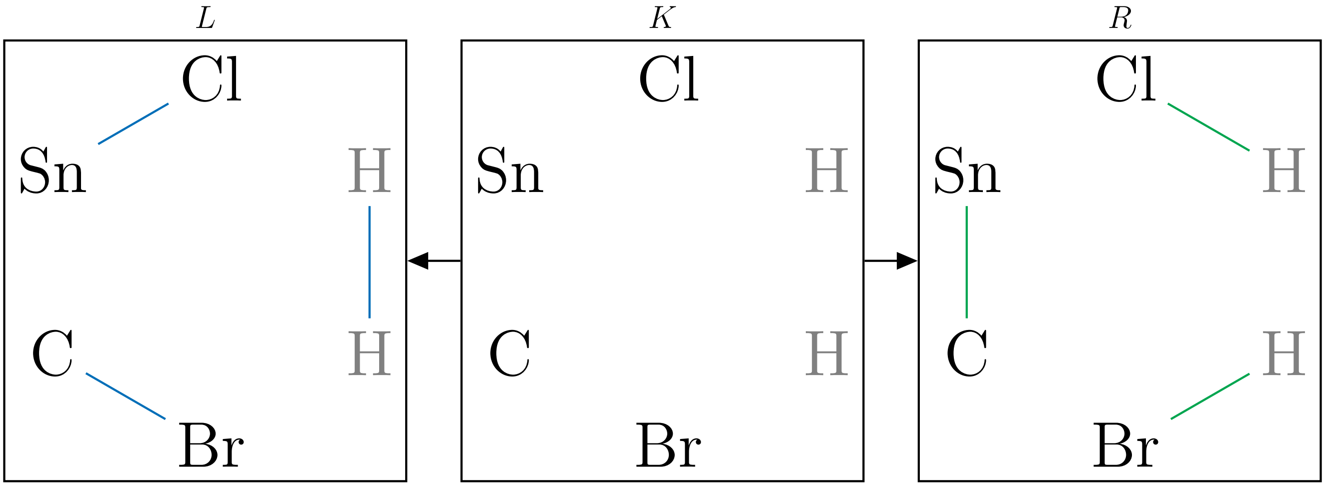

Files: out/793_r_158_11300100_{L, K, R}

0.0.160 159

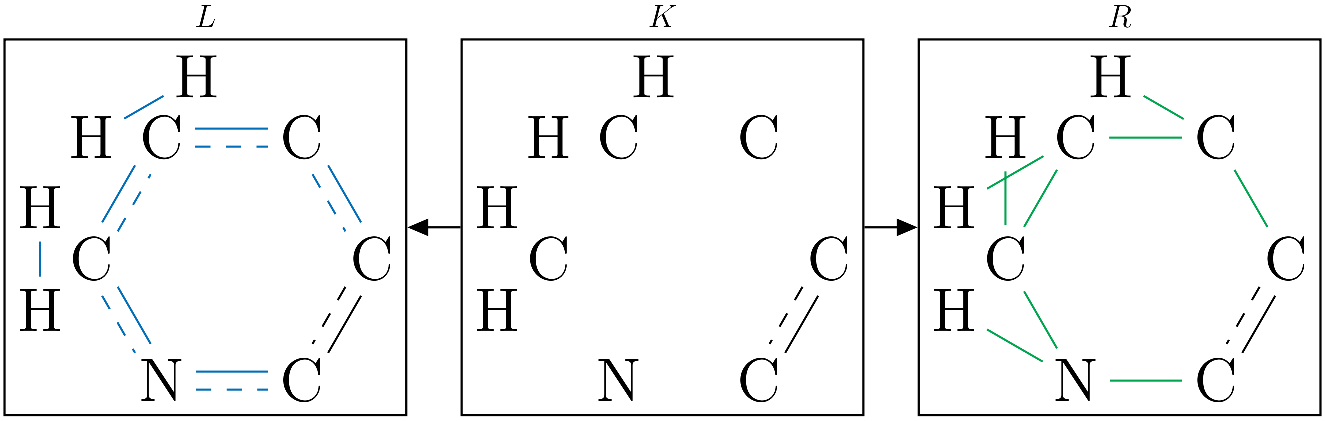

Files: out/796_r_159_10300000_{L, K, R}

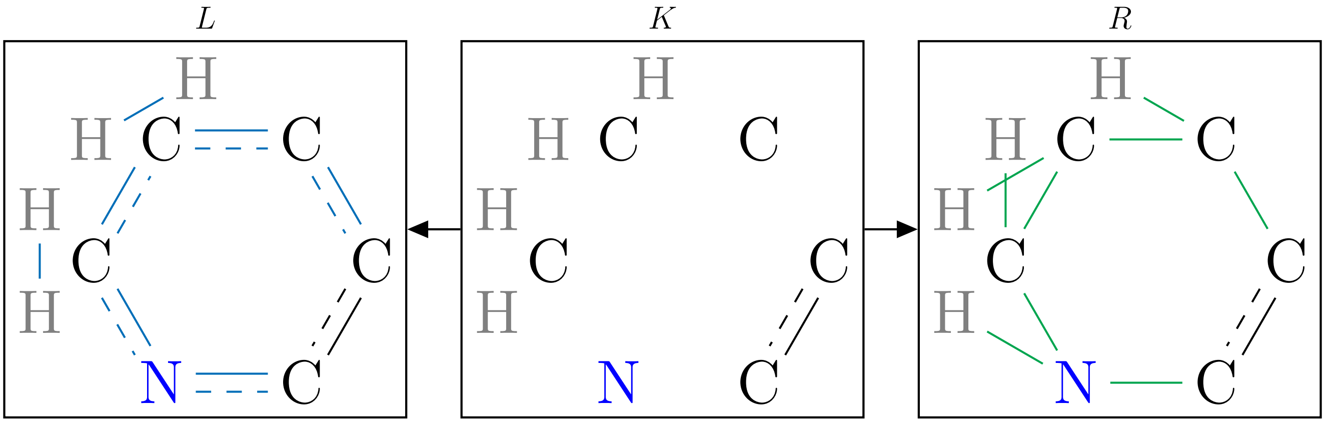

Files: out/798_r_159_11300100_{L, K, R}

0.0.161 160

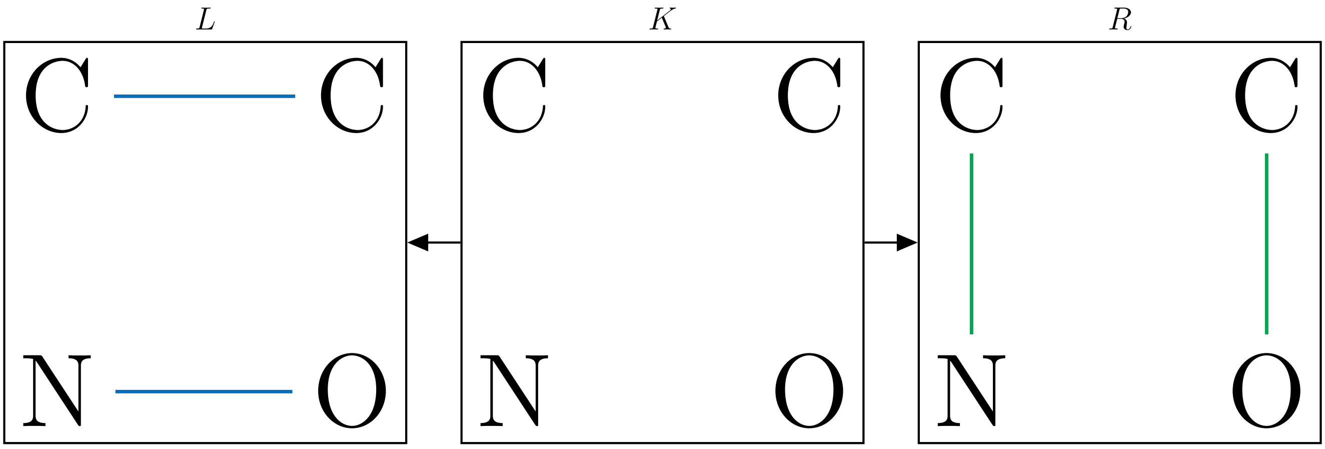

Files: out/801_r_160_10300000_{L, K, R}

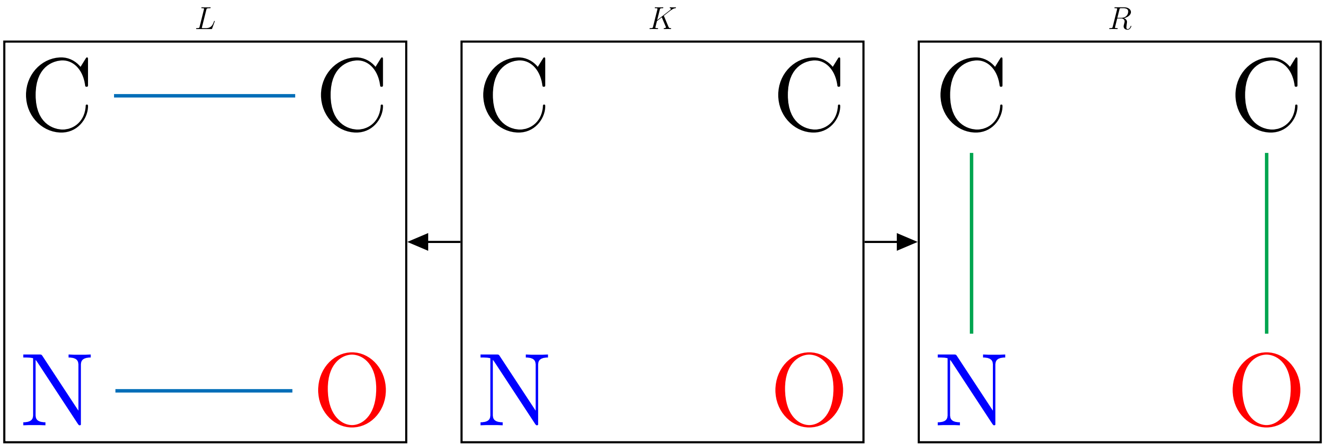

Files: out/803_r_160_11300100_{L, K, R}

0.0.162 161

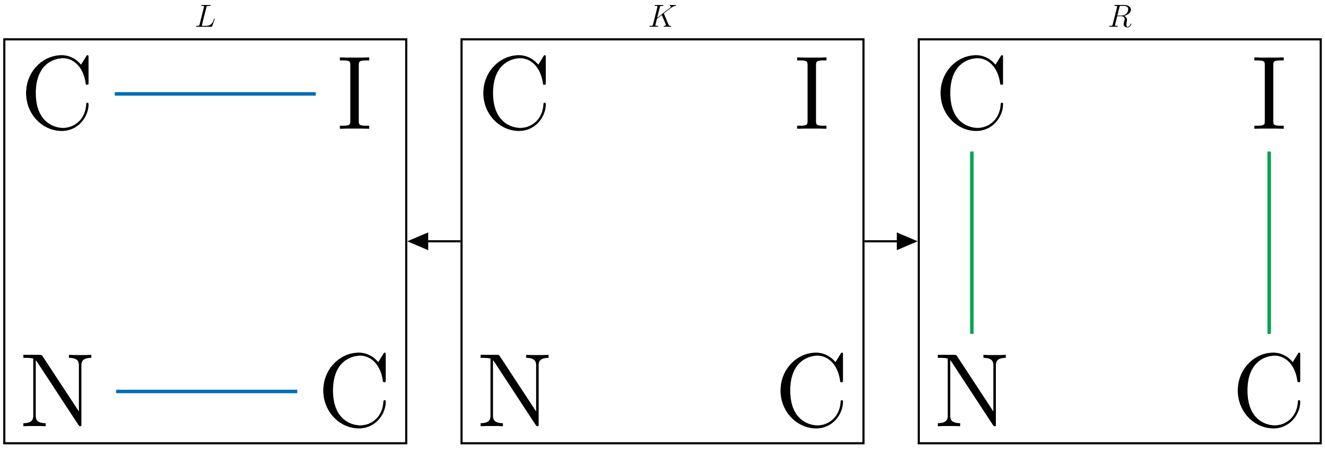

Files: out/806_r_161_10300000_{L, K, R}

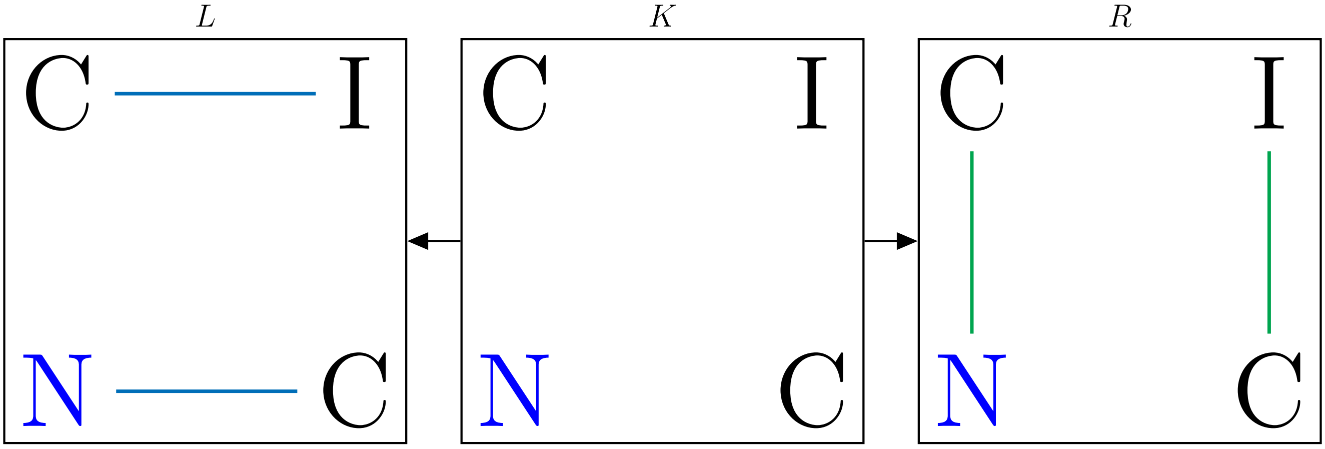

Files: out/808_r_161_11300100_{L, K, R}

0.0.163 162

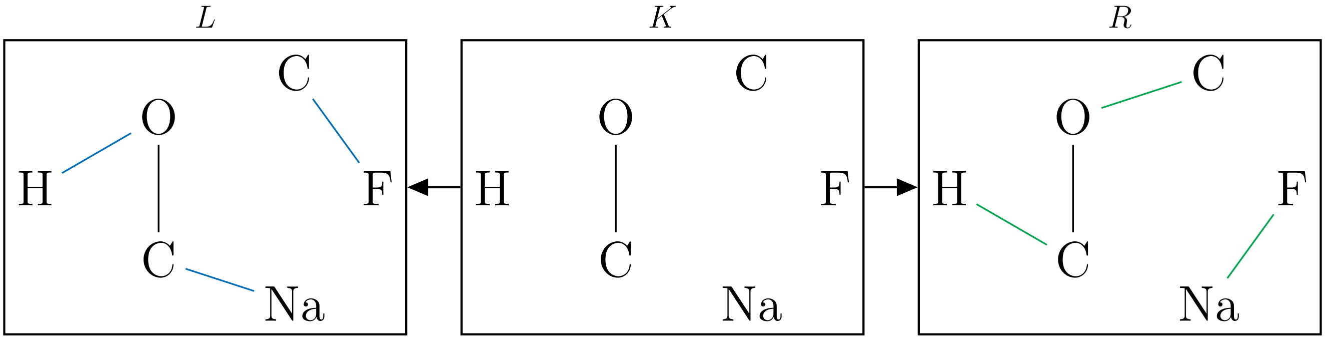

Files: out/811_r_162_10300000_{L, K, R}

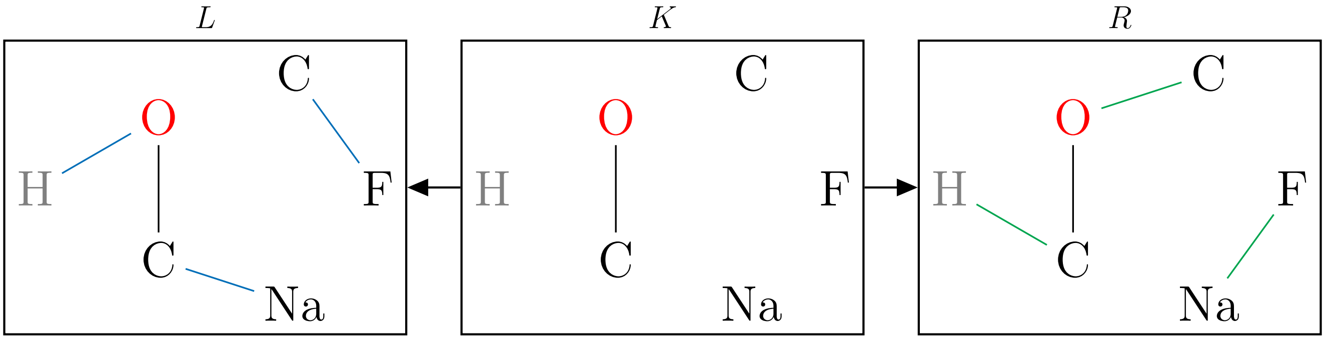

Files: out/813_r_162_11300100_{L, K, R}

0.0.164 163

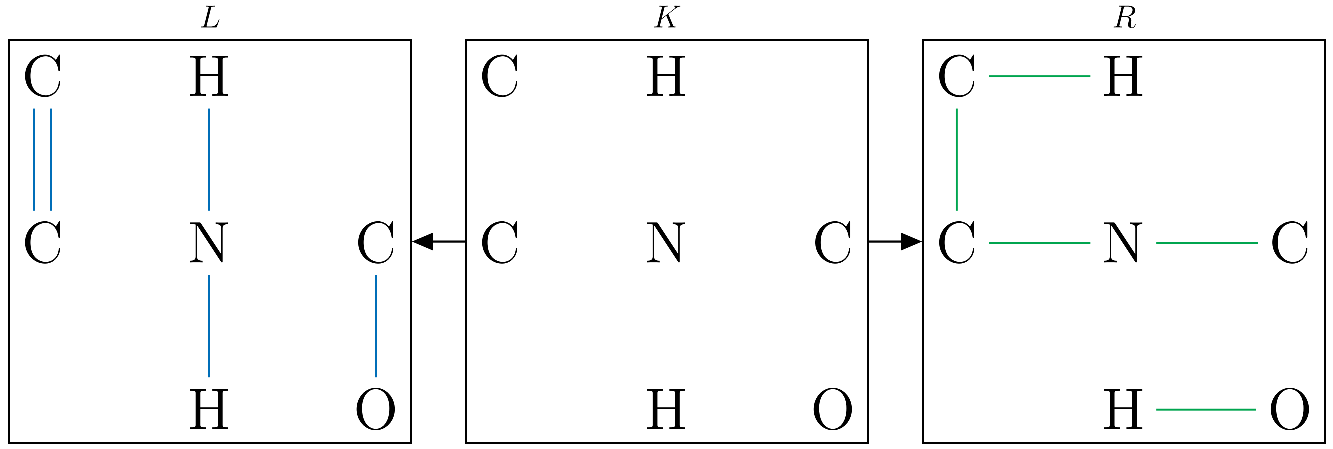

Files: out/816_r_163_10300000_{L, K, R}

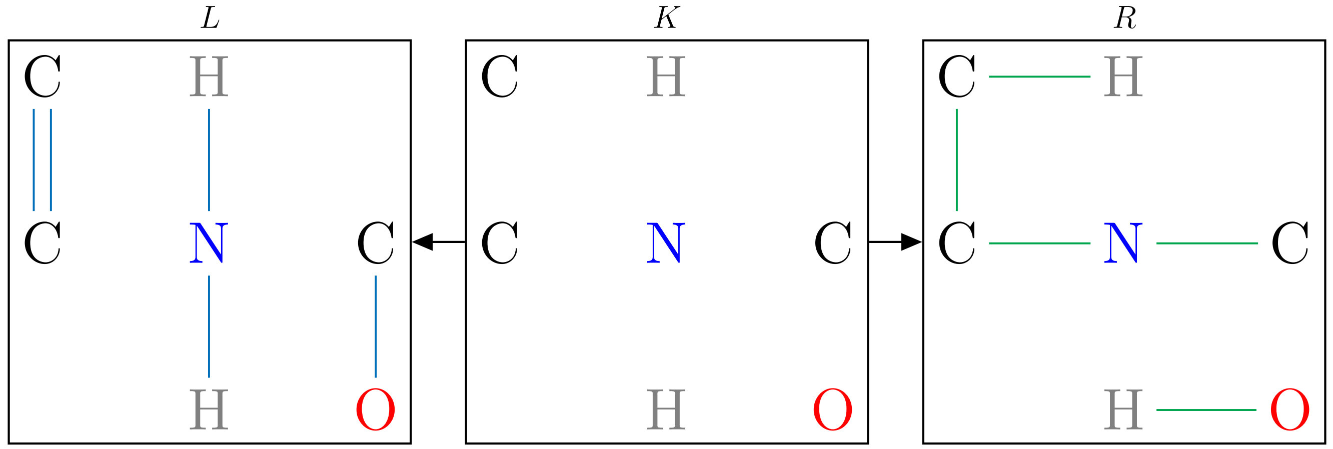

Files: out/818_r_163_11300100_{L, K, R}

0.0.165 164

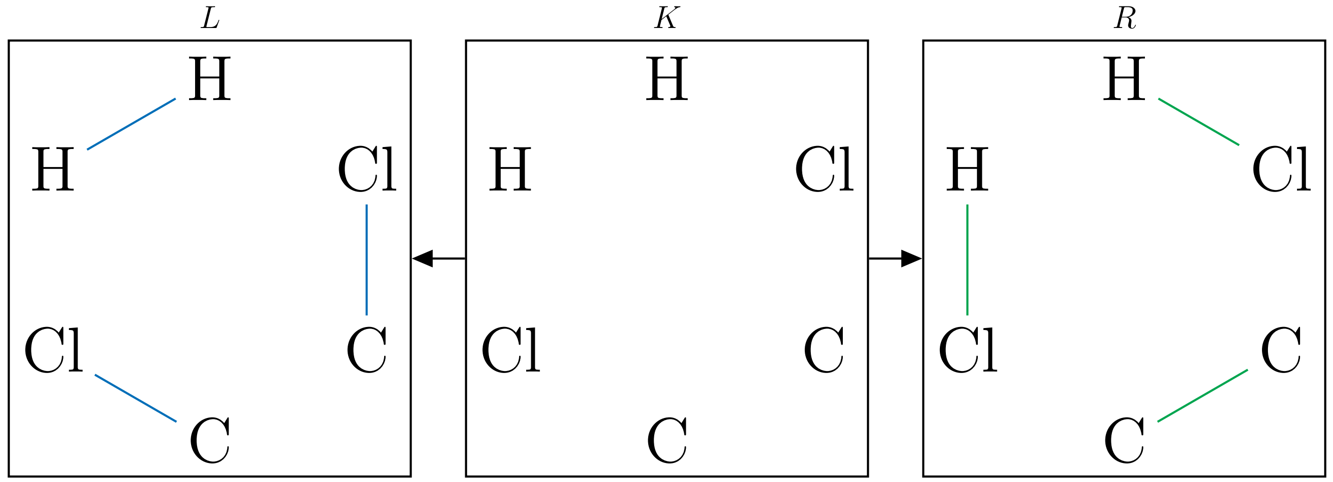

Files: out/821_r_164_10300000_{L, K, R}

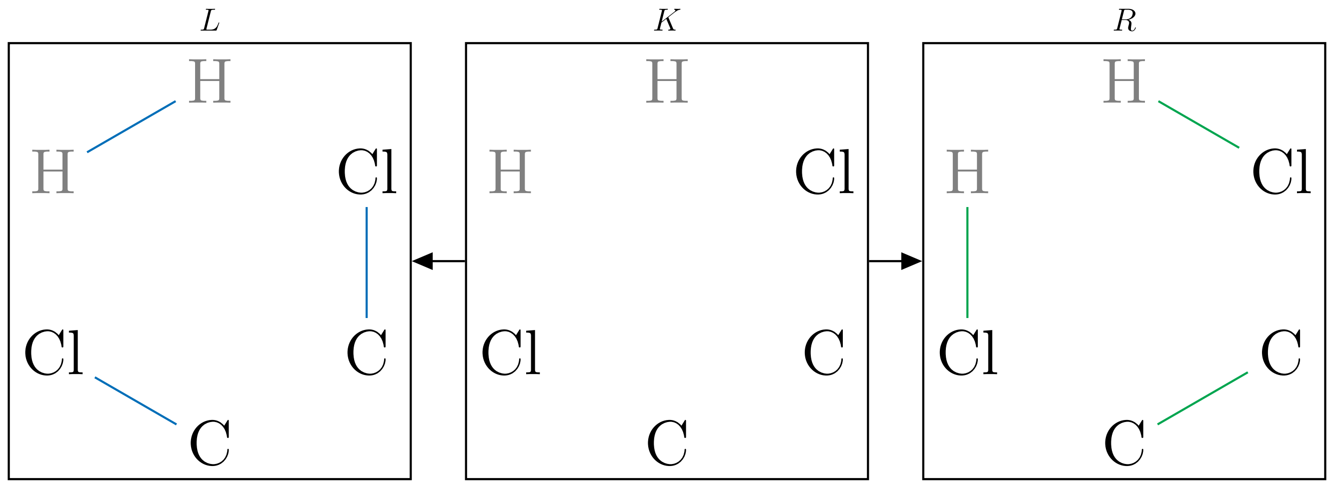

Files: out/823_r_164_11300100_{L, K, R}

0.0.166 165

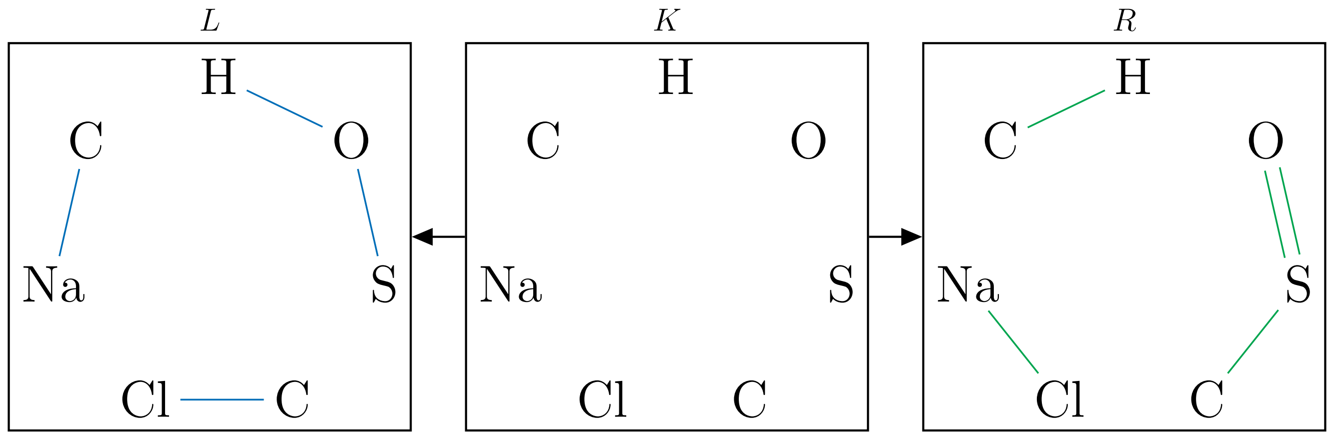

Files: out/826_r_165_10300000_{L, K, R}

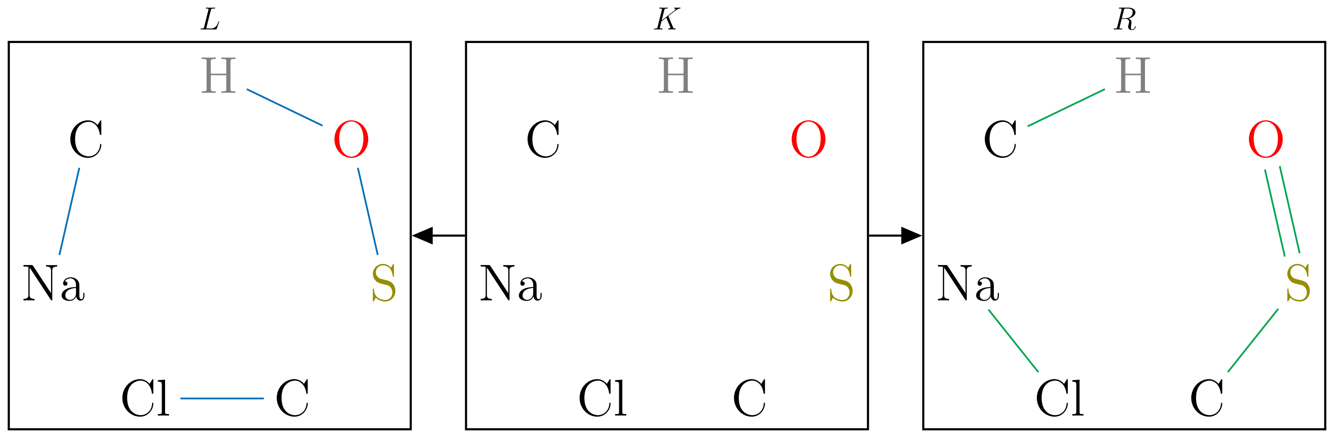

Files: out/828_r_165_11300100_{L, K, R}

0.0.167 166

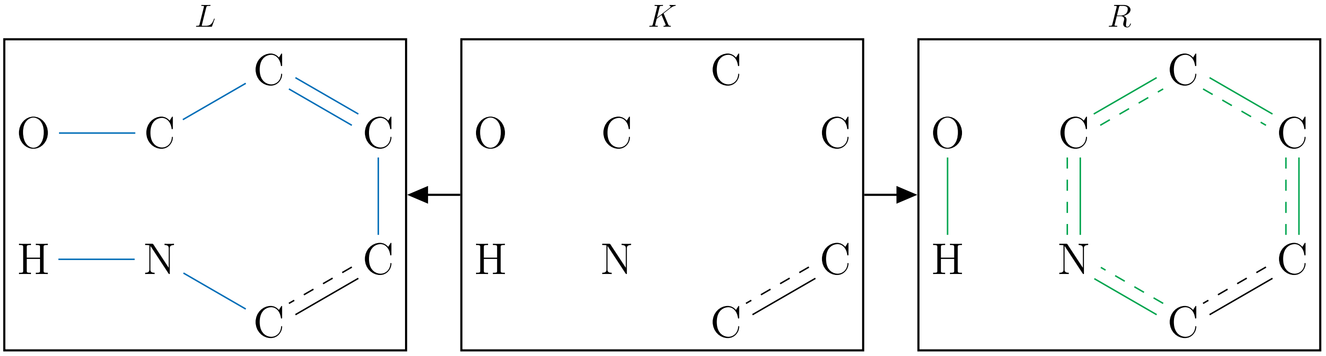

Files: out/831_r_166_10300000_{L, K, R}

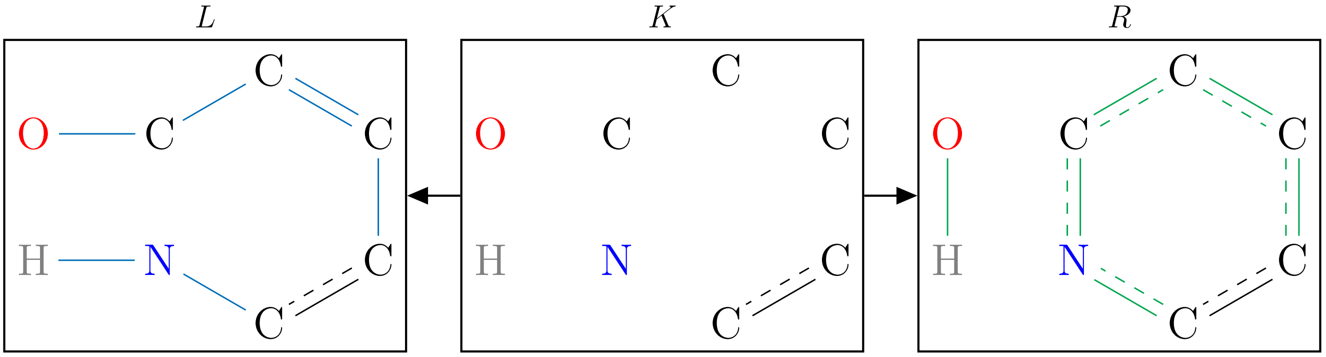

Files: out/833_r_166_11300100_{L, K, R}

0.0.168 167

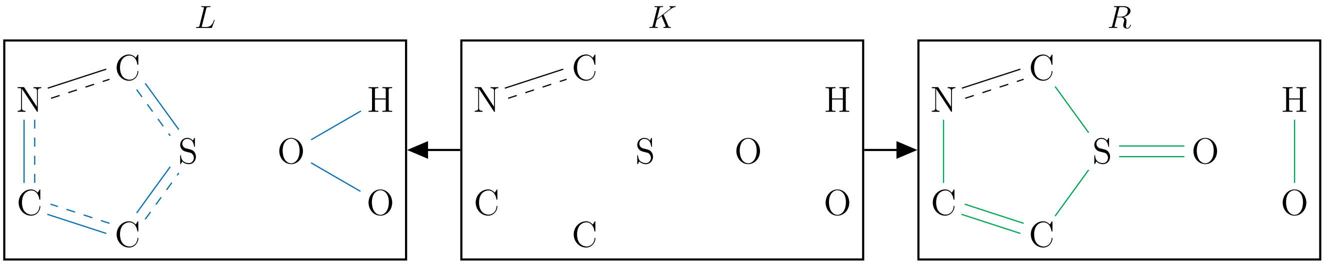

Files: out/836_r_167_10300000_{L, K, R}

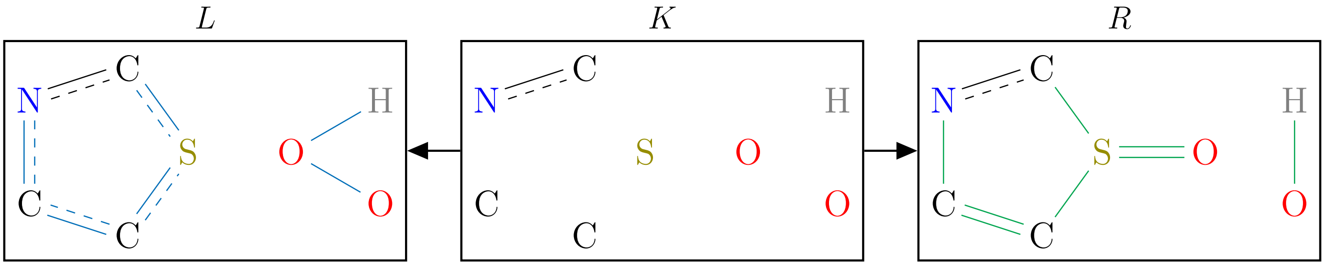

Files: out/838_r_167_11300100_{L, K, R}

0.0.169 168

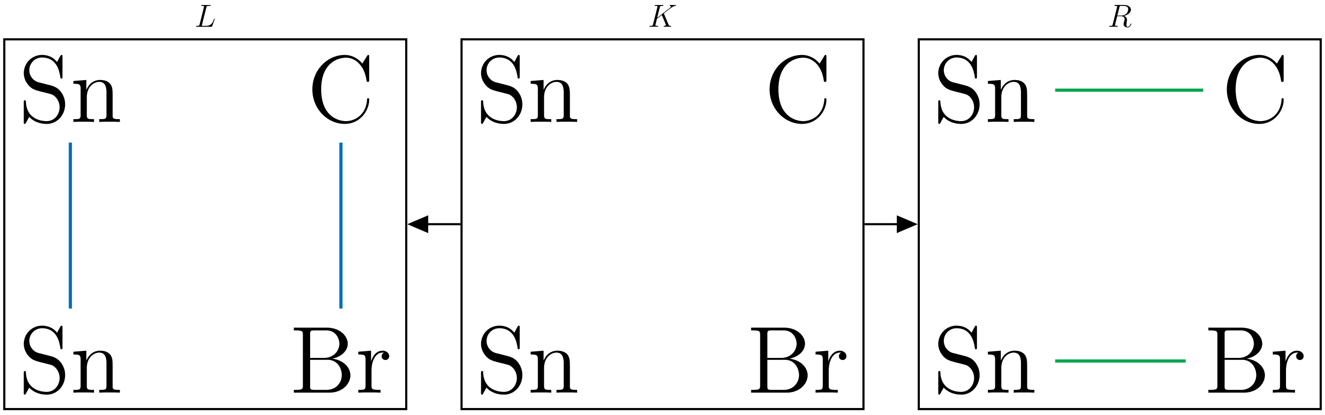

Files: out/841_r_168_10300000_{L, K, R}

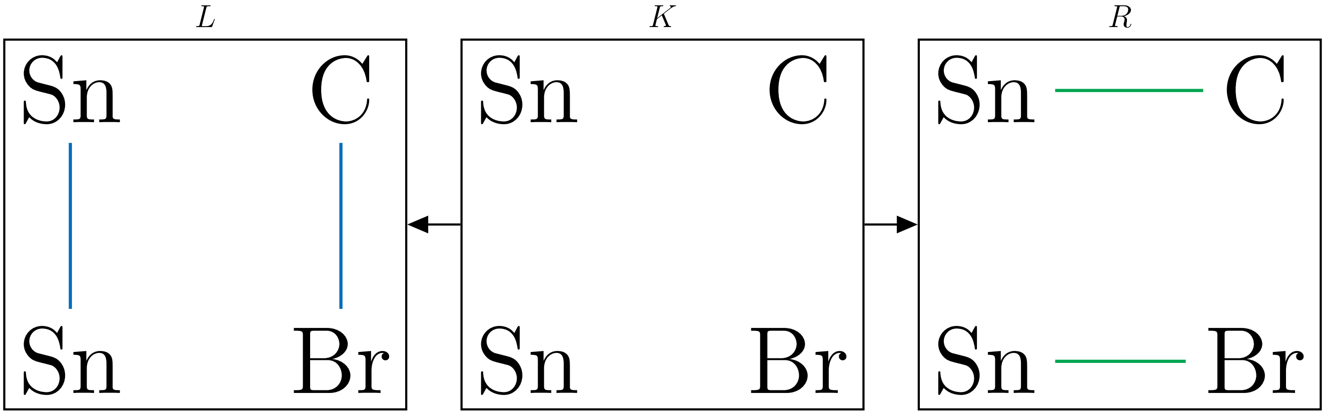

Files: out/843_r_168_11300100_{L, K, R}

0.0.170 169

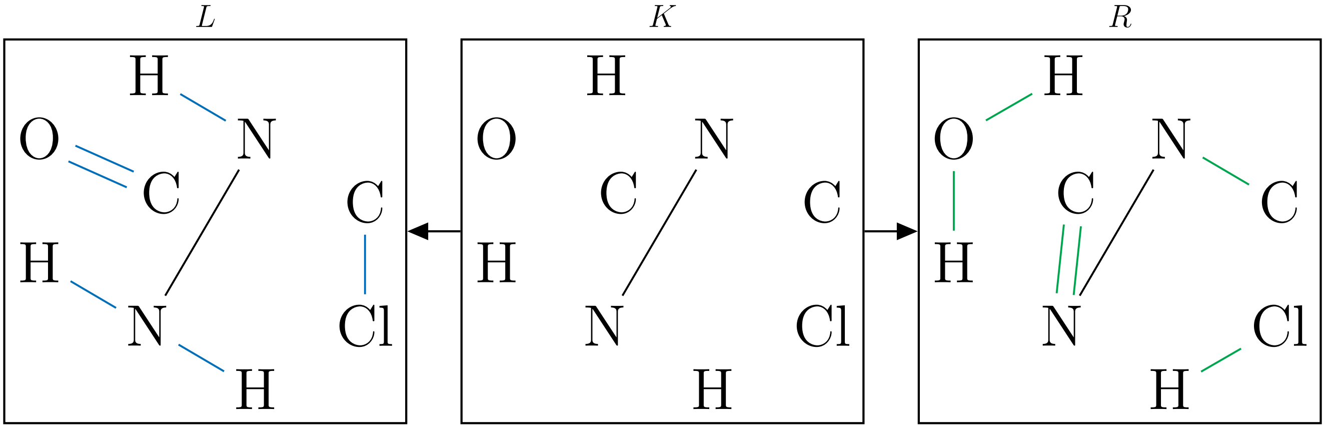

Files: out/846_r_169_10300000_{L, K, R}

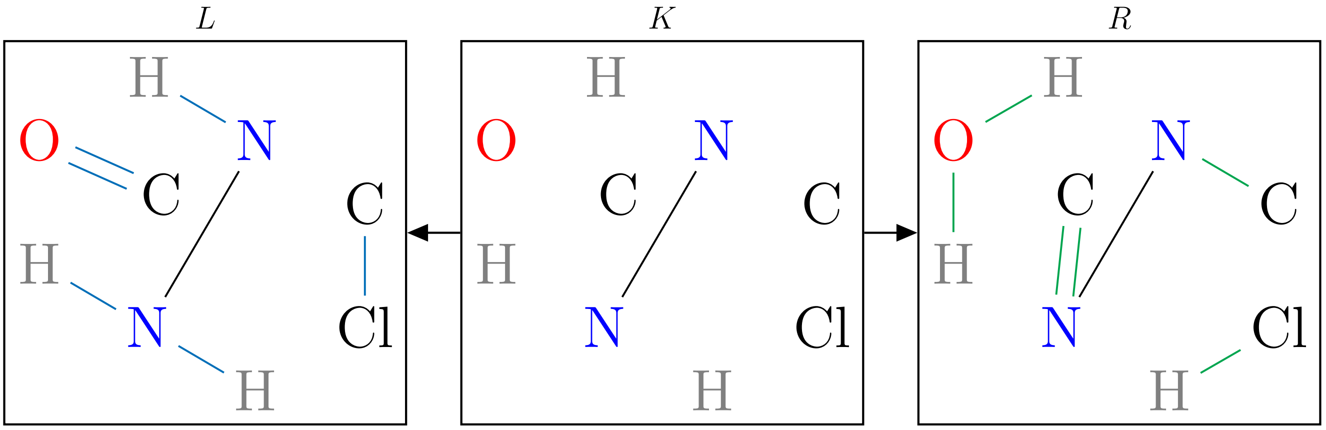

Files: out/848_r_169_11300100_{L, K, R}

0.0.171 170

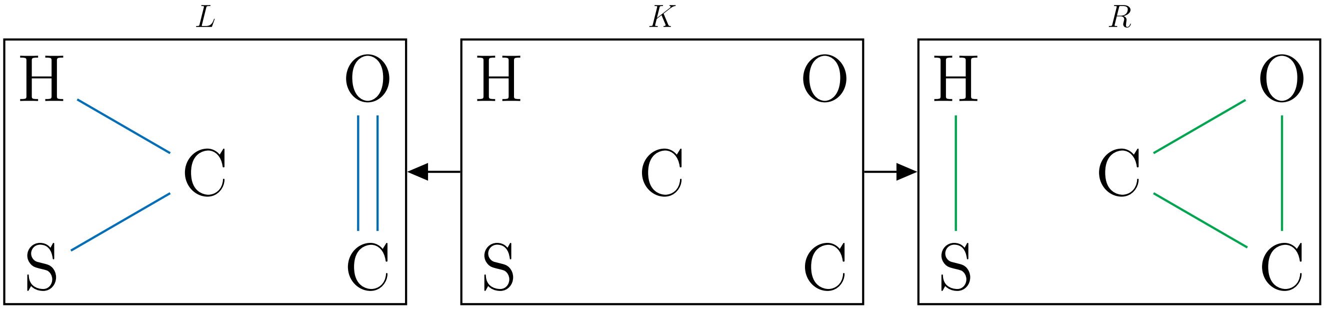

Files: out/851_r_170_10300000_{L, K, R}

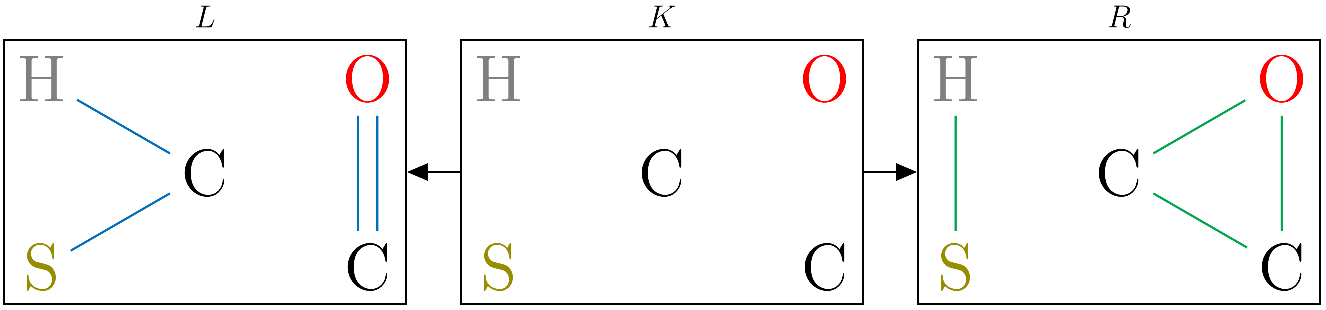

Files: out/853_r_170_11300100_{L, K, R}

0.0.172 171

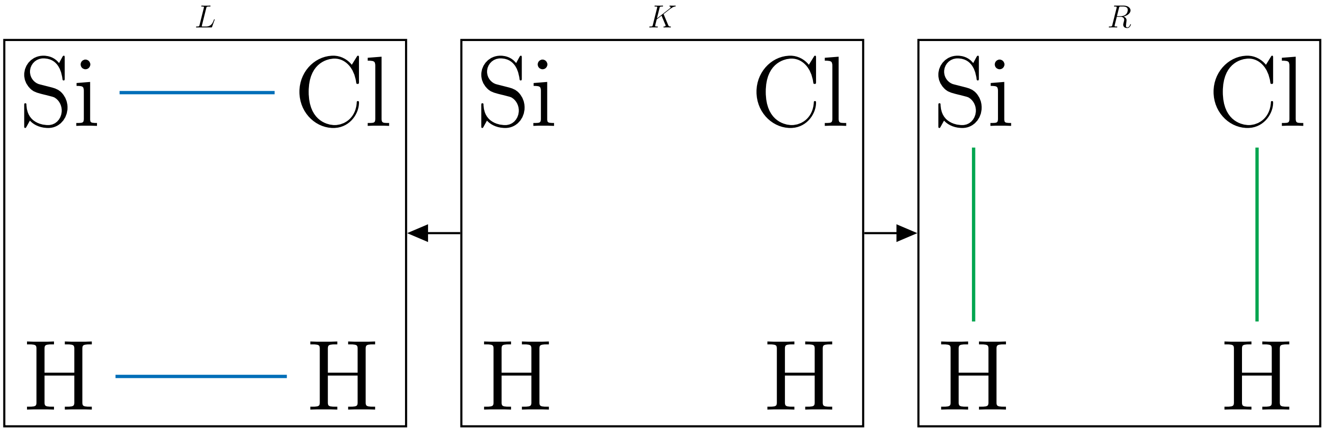

Files: out/856_r_171_10300000_{L, K, R}

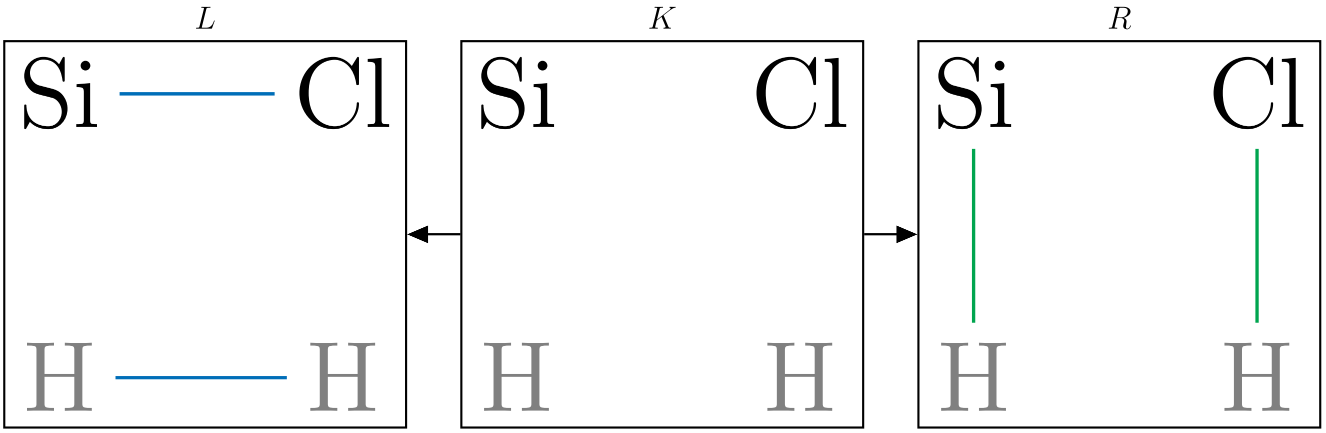

Files: out/858_r_171_11300100_{L, K, R}

0.0.173 172

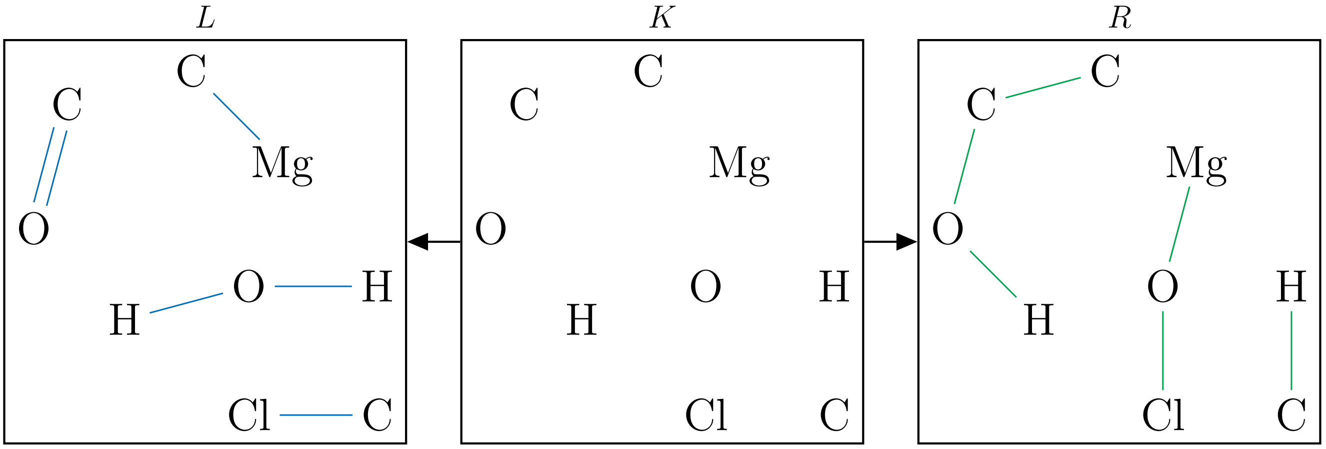

Files: out/861_r_172_10300000_{L, K, R}

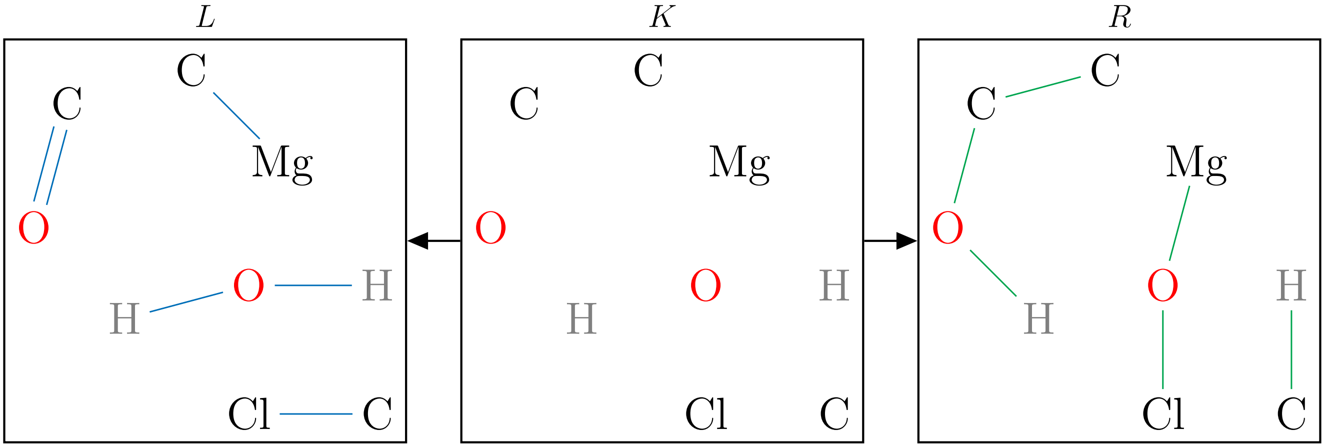

Files: out/863_r_172_11300100_{L, K, R}

0.0.174 173

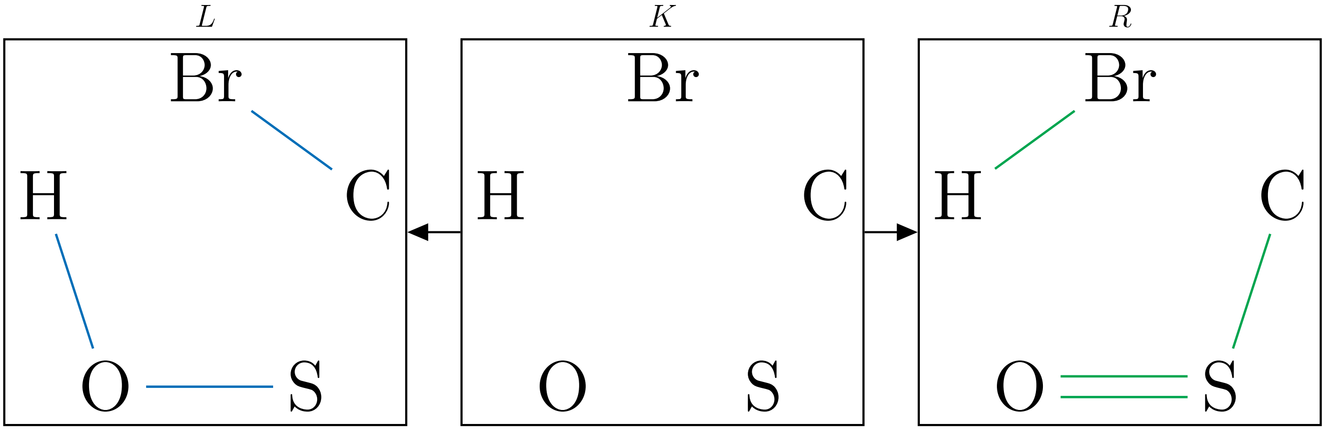

Files: out/866_r_173_10300000_{L, K, R}

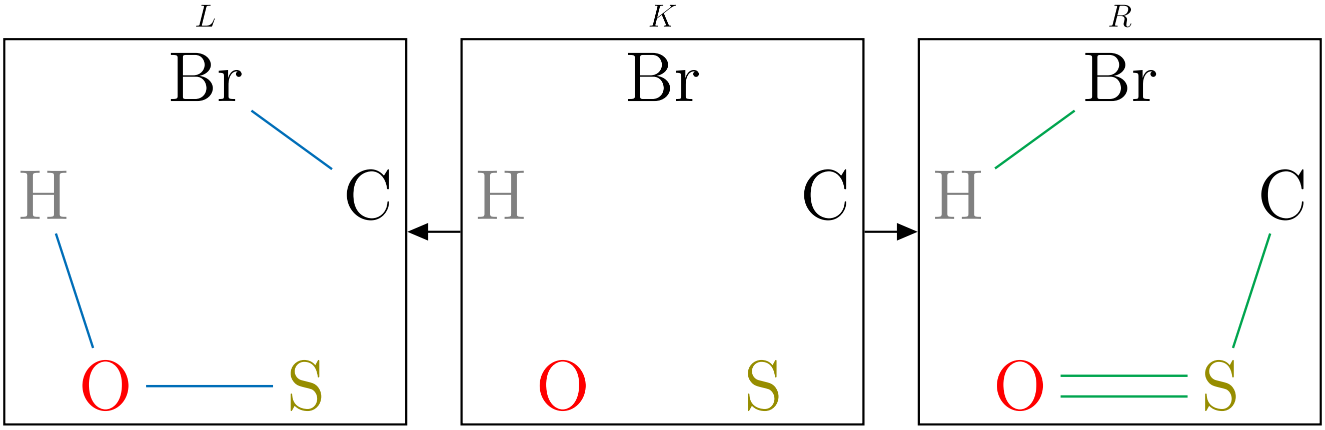

Files: out/868_r_173_11300100_{L, K, R}

0.0.175 174

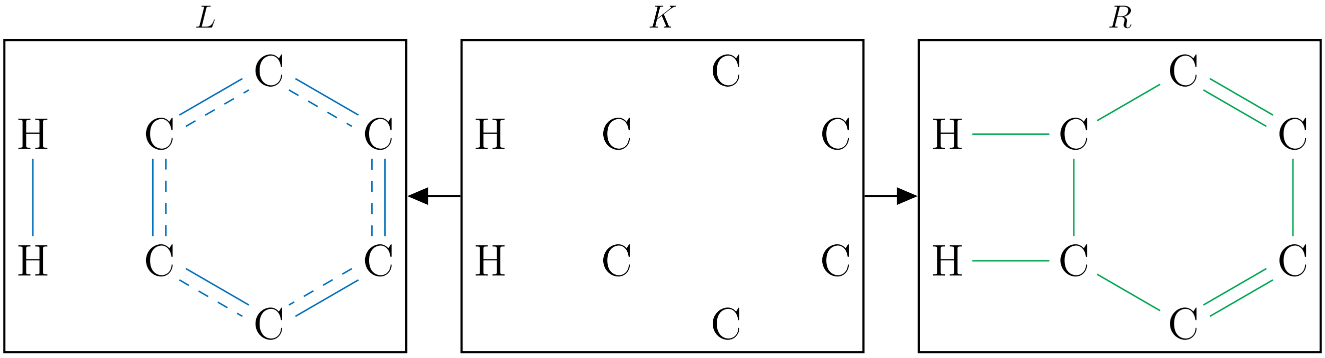

Files: out/871_r_174_10300000_{L, K, R}

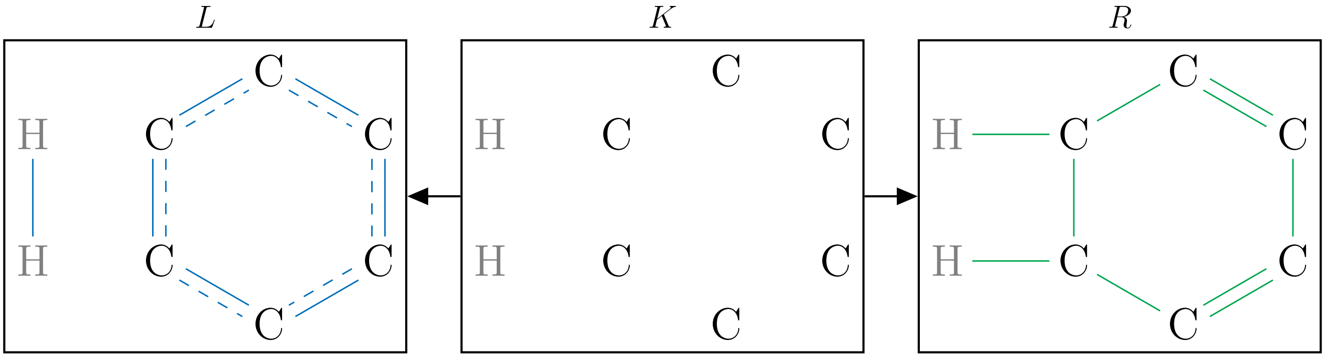

Files: out/873_r_174_11300100_{L, K, R}

0.0.176 175

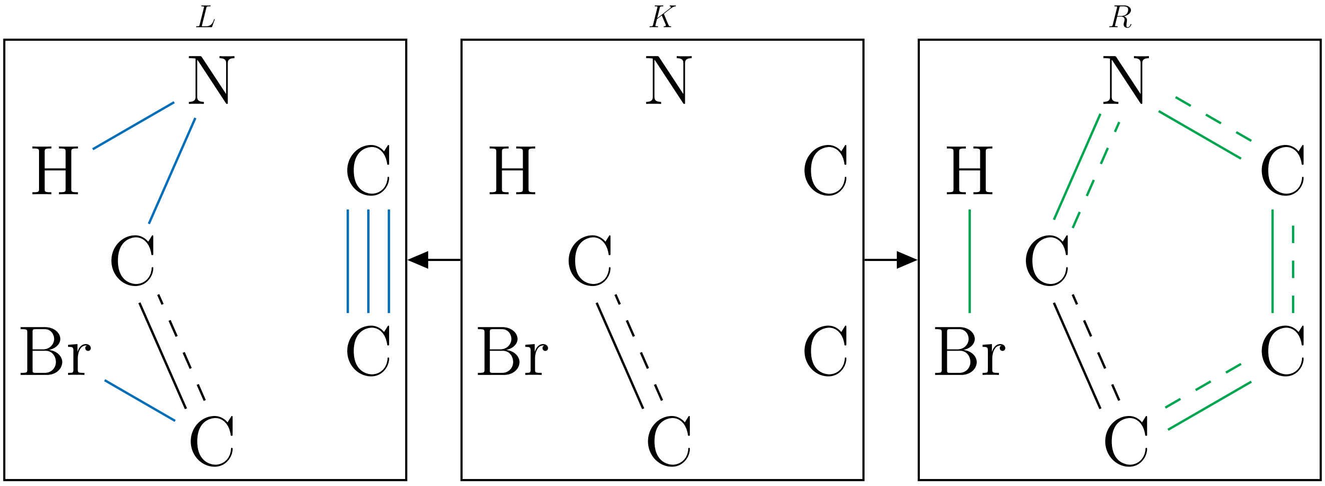

Files: out/876_r_175_10300000_{L, K, R}

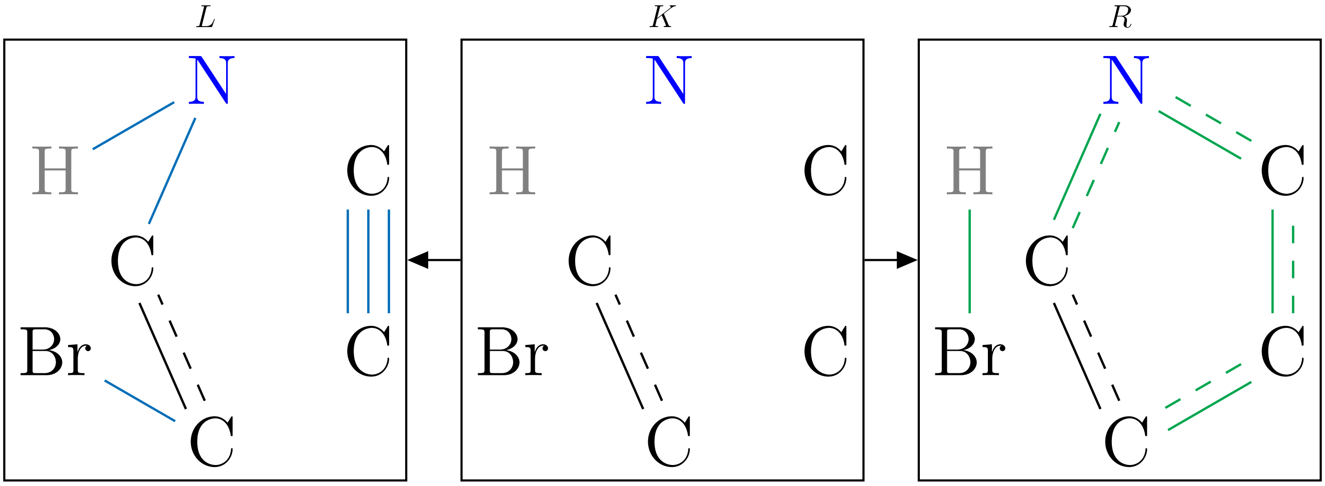

Files: out/878_r_175_11300100_{L, K, R}

0.0.177 176

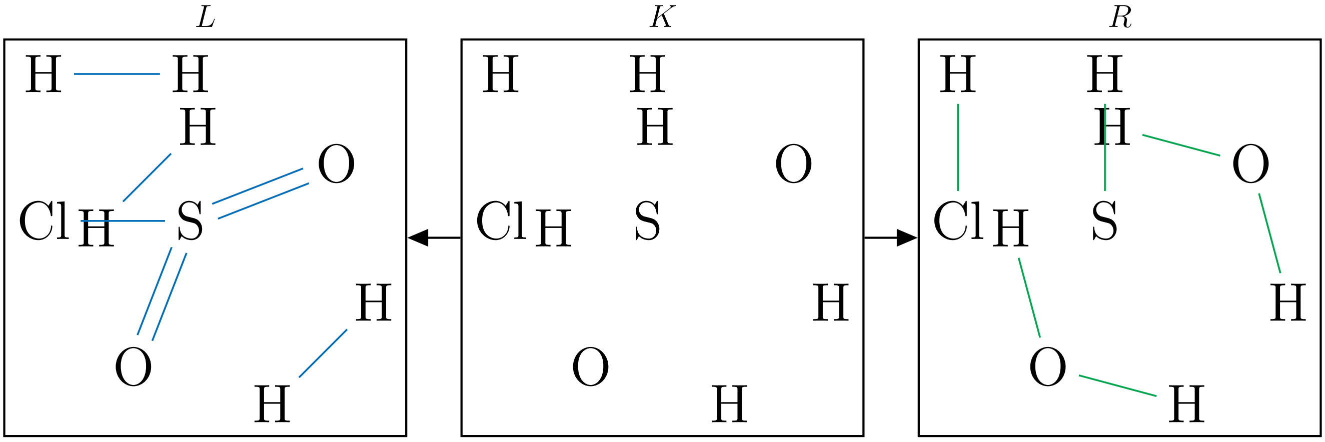

Files: out/881_r_176_10300000_{L, K, R}

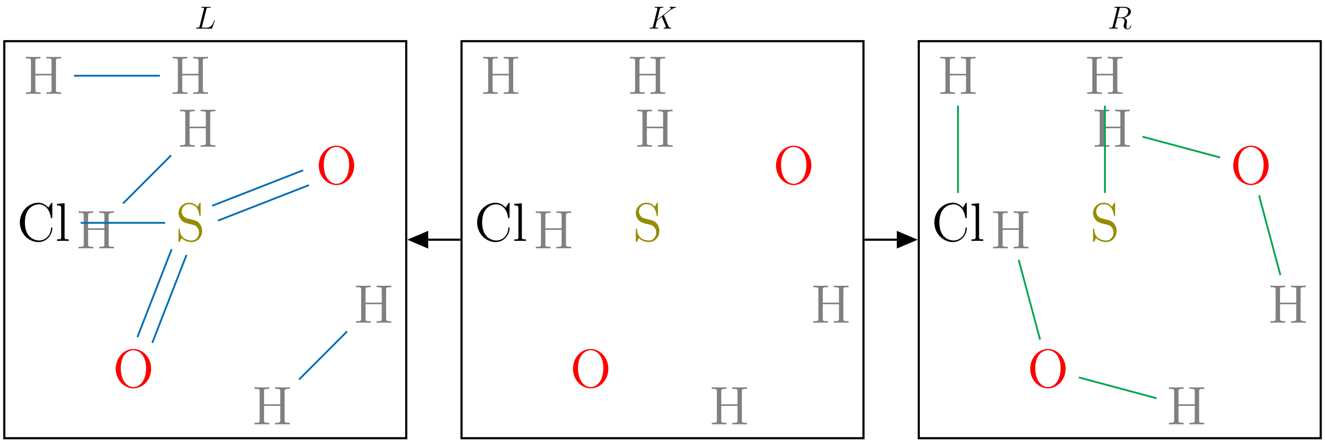

Files: out/883_r_176_11300100_{L, K, R}

0.0.178 177

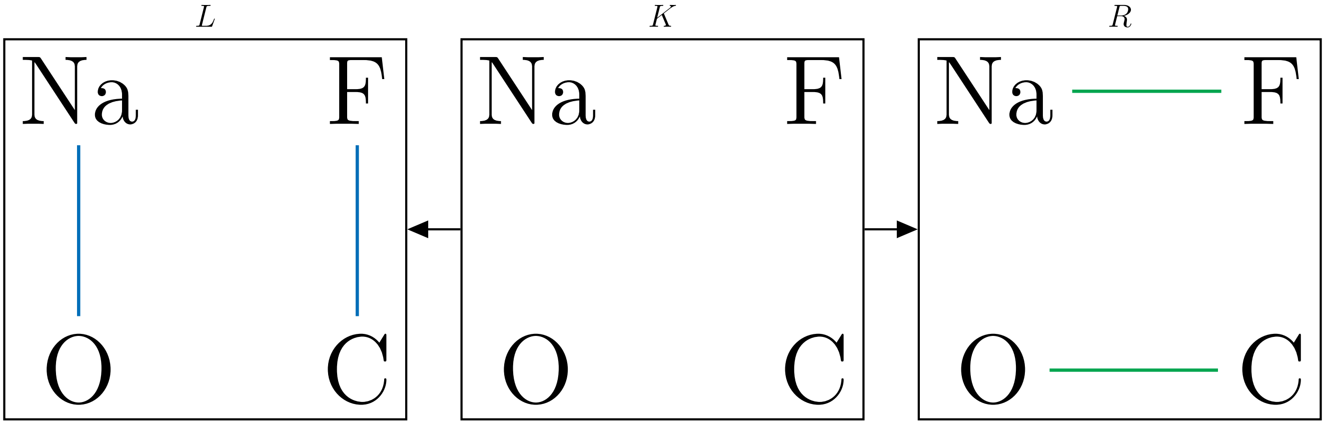

Files: out/886_r_177_10300000_{L, K, R}

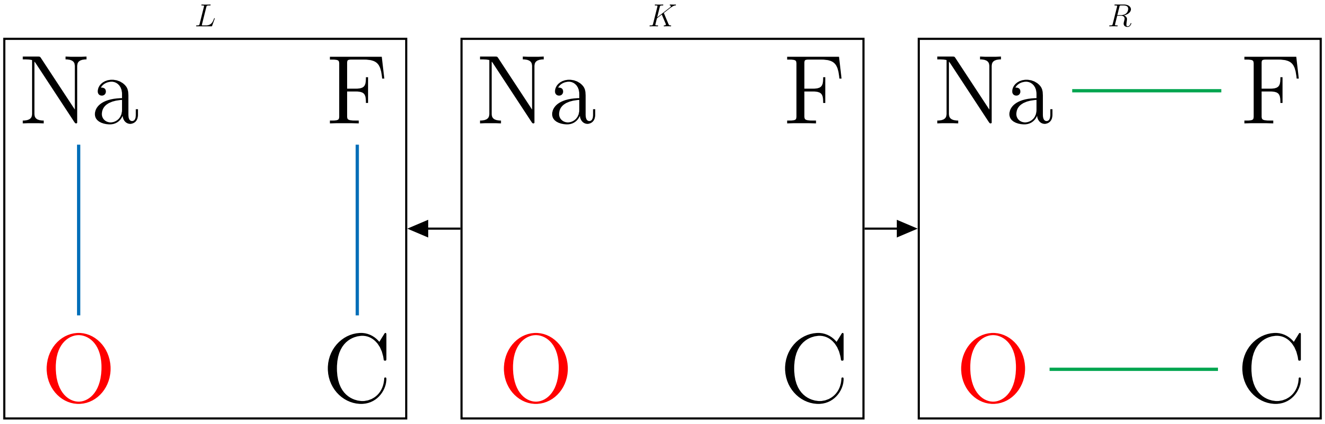

Files: out/888_r_177_11300100_{L, K, R}

0.0.179 178

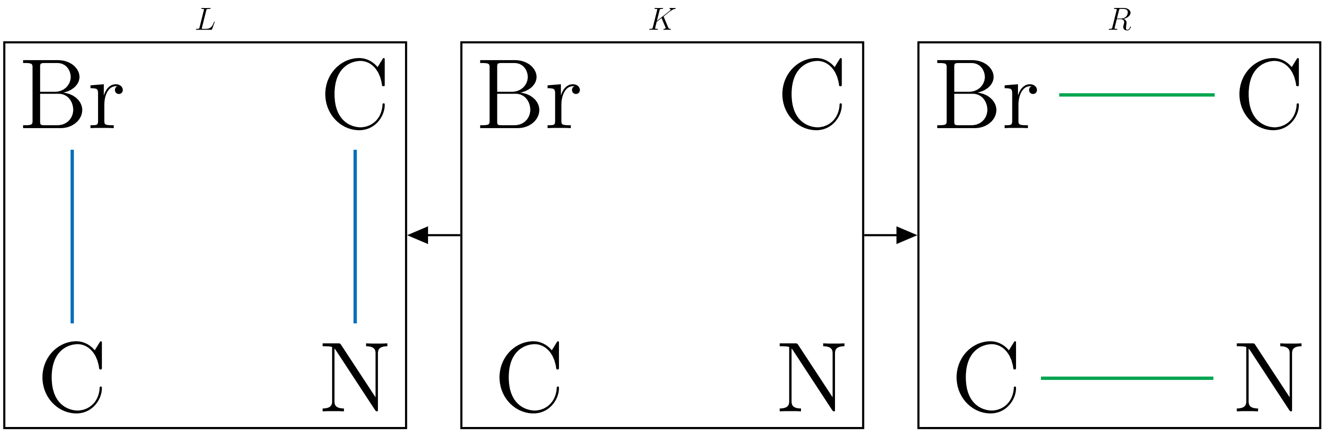

Files: out/891_r_178_10300000_{L, K, R}

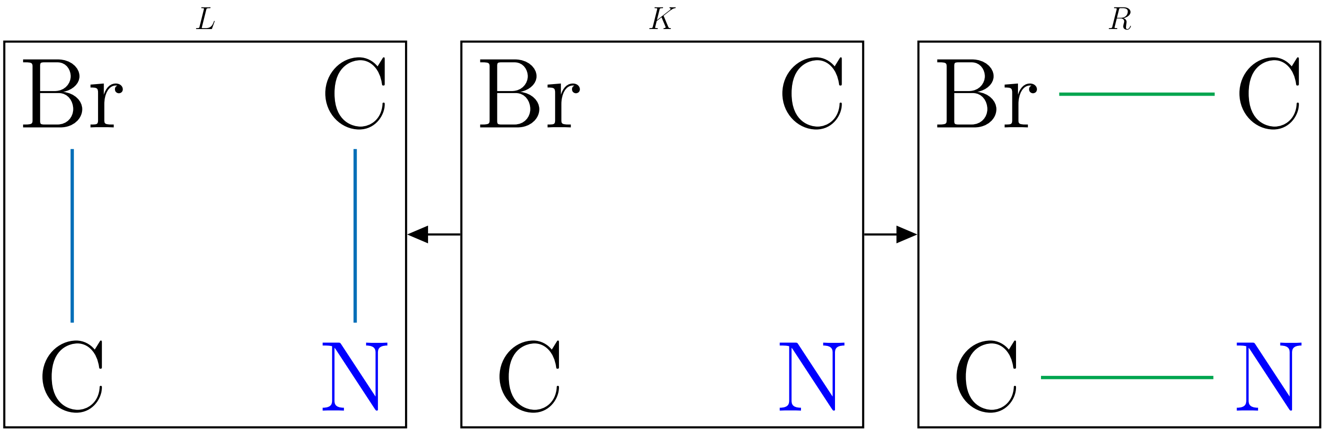

Files: out/893_r_178_11300100_{L, K, R}

0.0.180 179

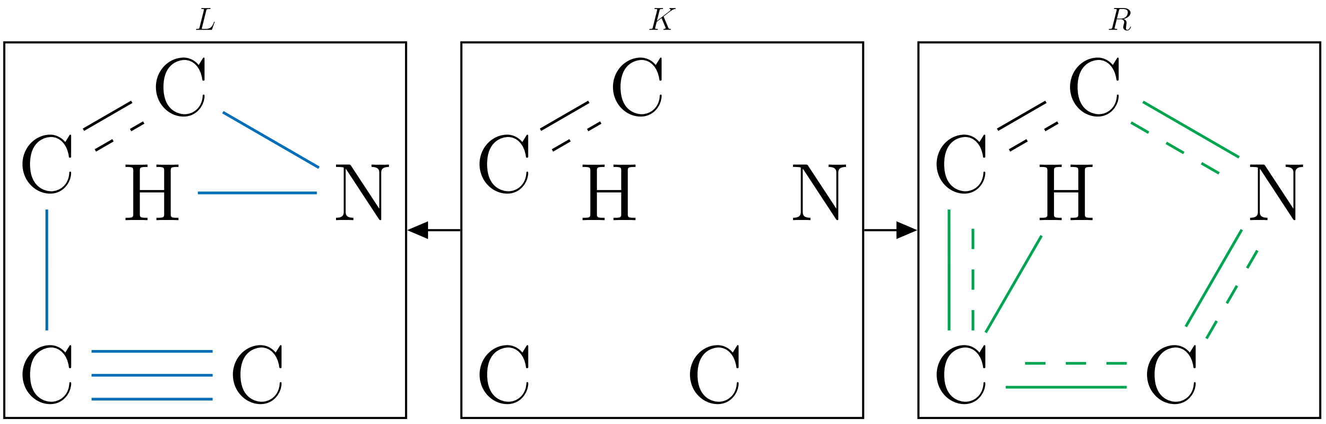

Files: out/896_r_179_10300000_{L, K, R}

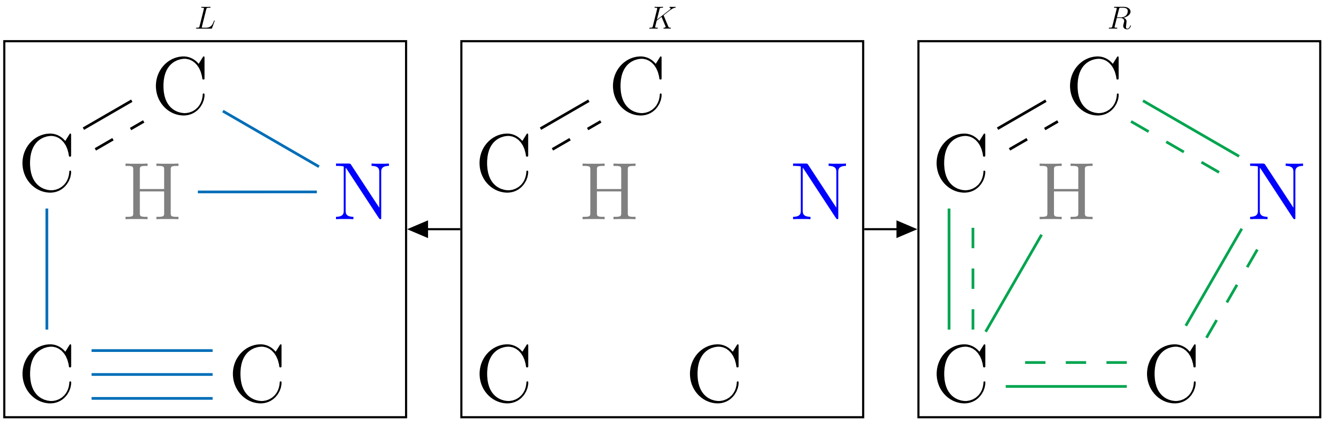

Files: out/898_r_179_11300100_{L, K, R}

0.0.181 180

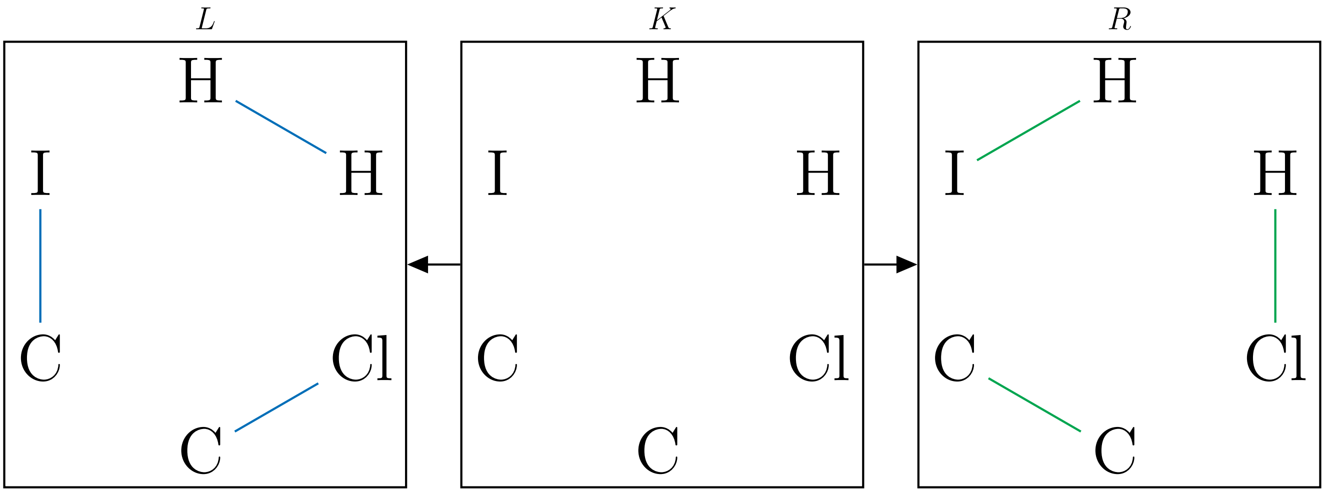

Files: out/901_r_180_10300000_{L, K, R}

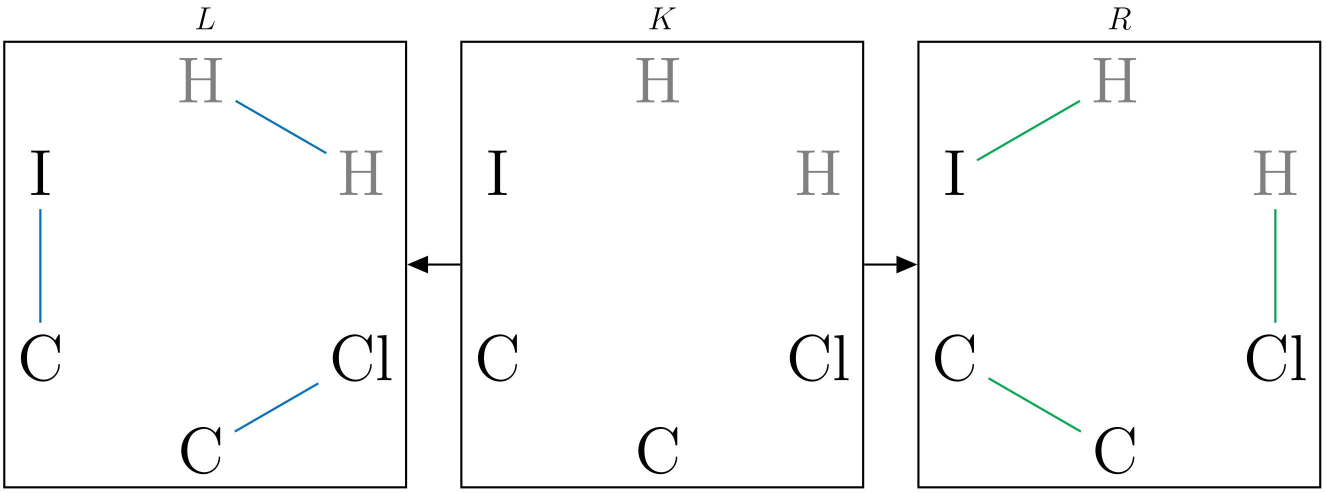

Files: out/903_r_180_11300100_{L, K, R}

0.0.182 181

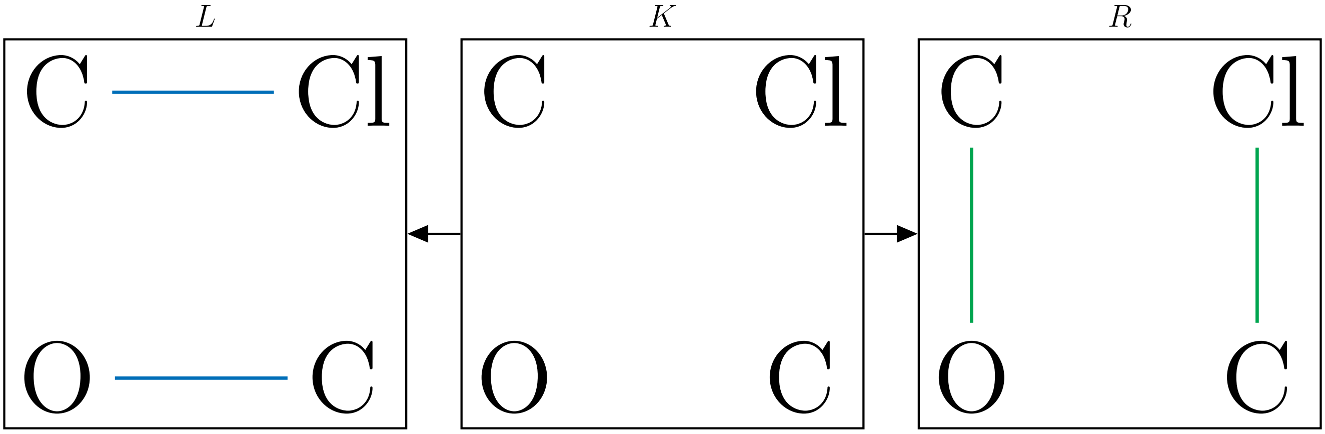

Files: out/906_r_181_10300000_{L, K, R}

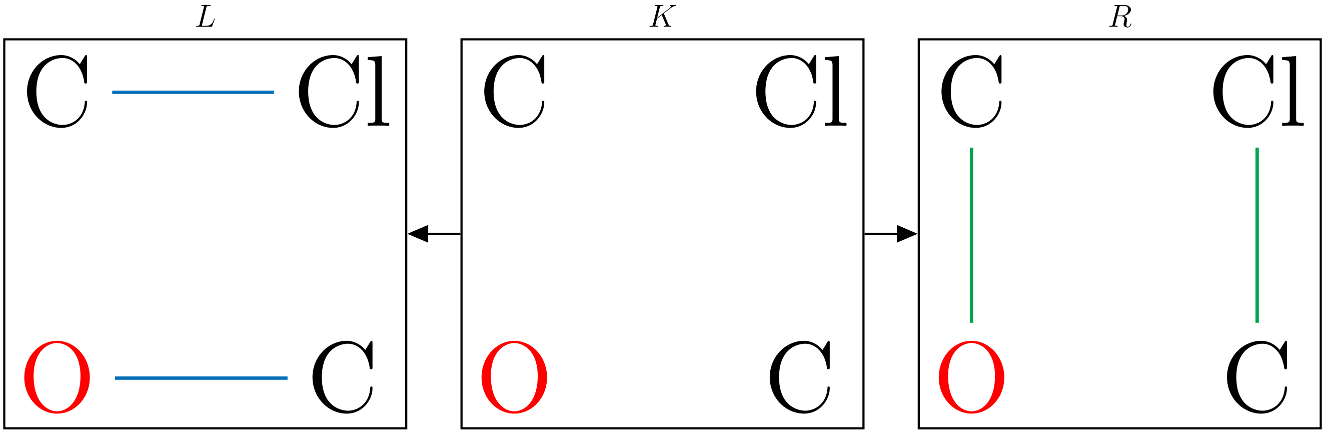

Files: out/908_r_181_11300100_{L, K, R}

0.0.183 182

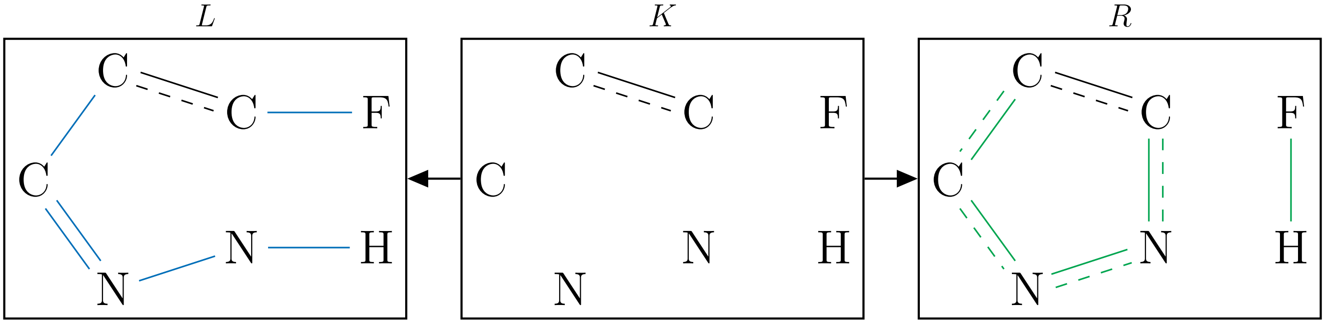

Files: out/911_r_182_10300000_{L, K, R}

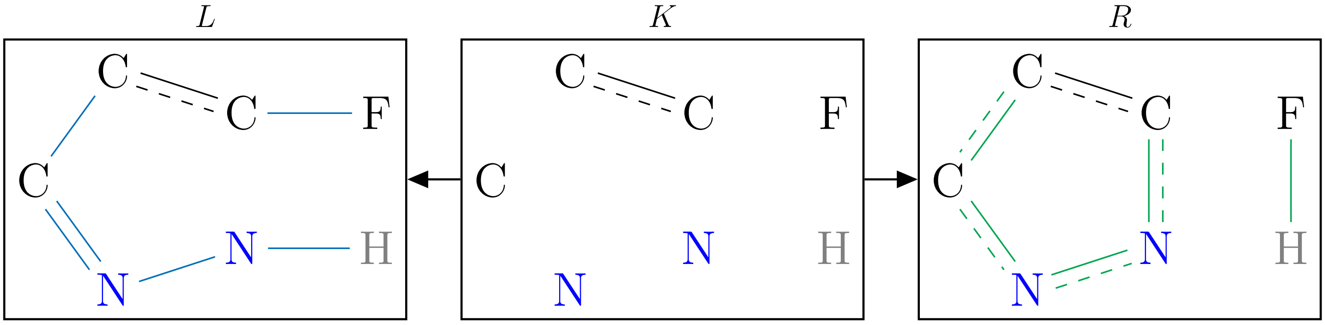

Files: out/913_r_182_11300100_{L, K, R}

0.0.184 183

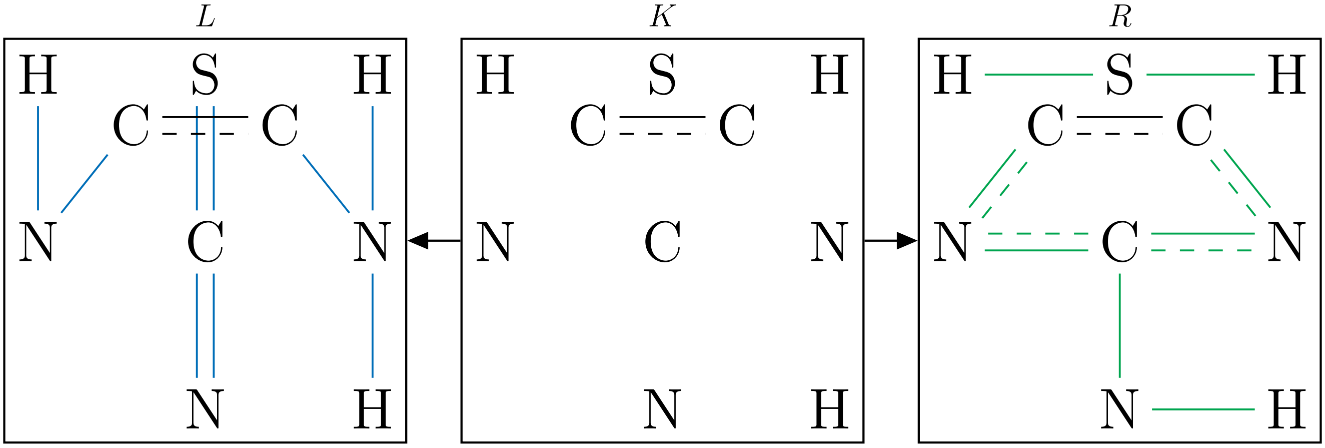

Files: out/916_r_183_10300000_{L, K, R}

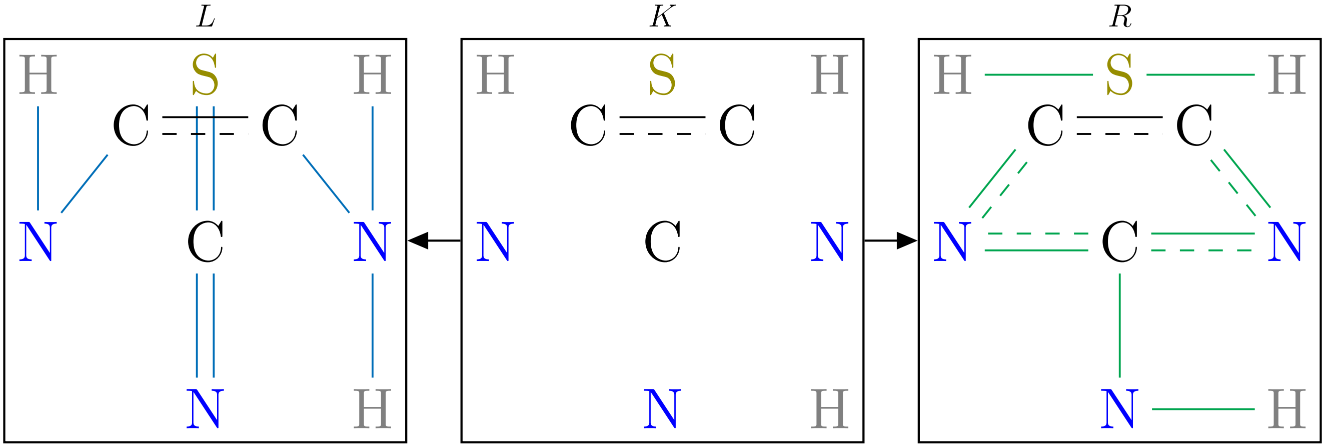

Files: out/918_r_183_11300100_{L, K, R}

0.0.185 184

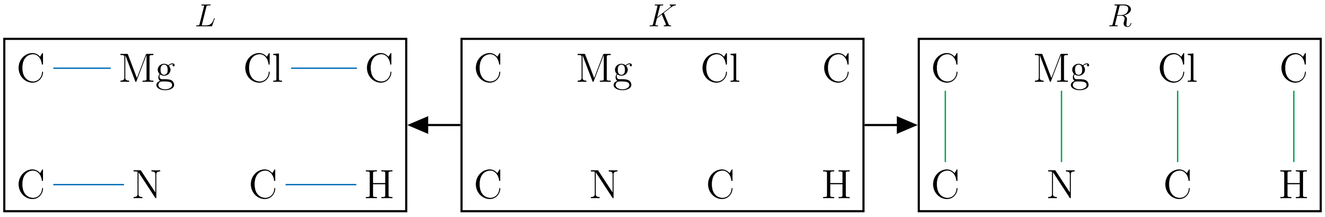

Files: out/921_r_184_10300000_{L, K, R}

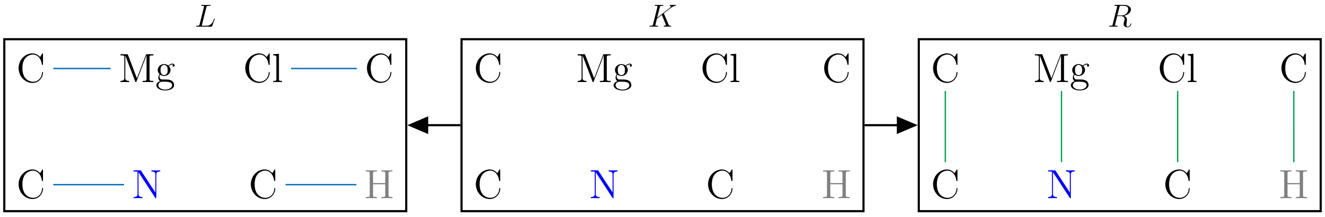

Files: out/923_r_184_11300100_{L, K, R}

0.0.186 185

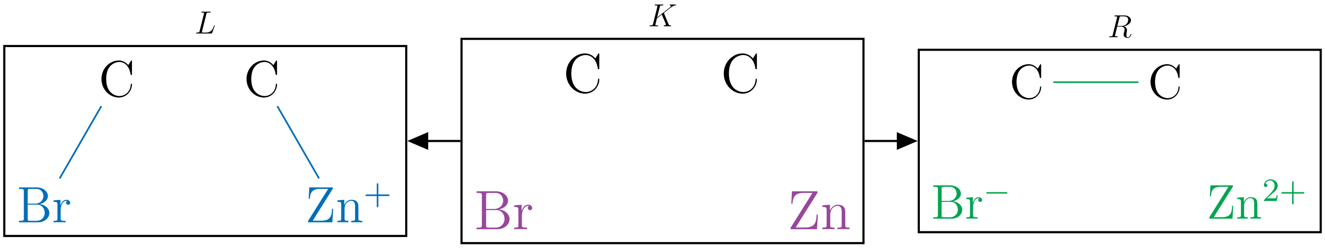

Files: out/926_r_185_10300000_{L, K, R}

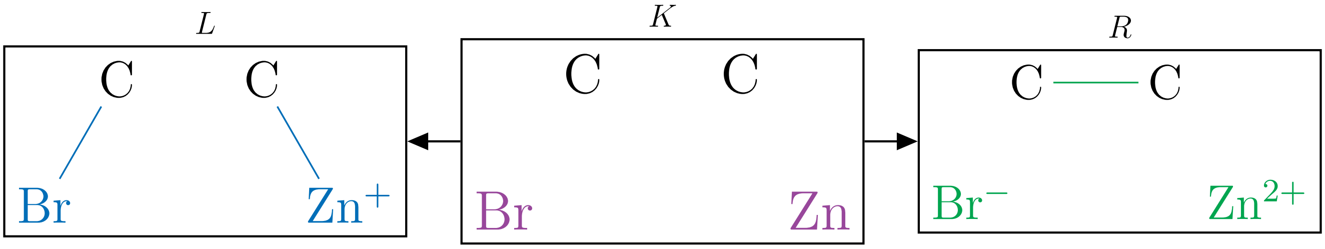

Files: out/928_r_185_11300100_{L, K, R}

0.0.187 186

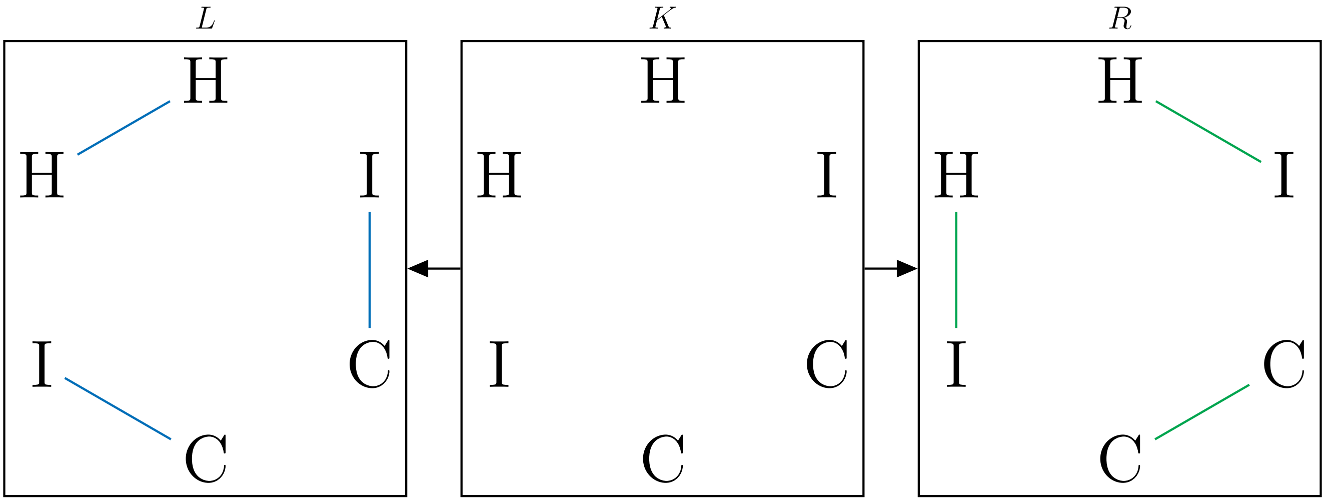

Files: out/931_r_186_10300000_{L, K, R}

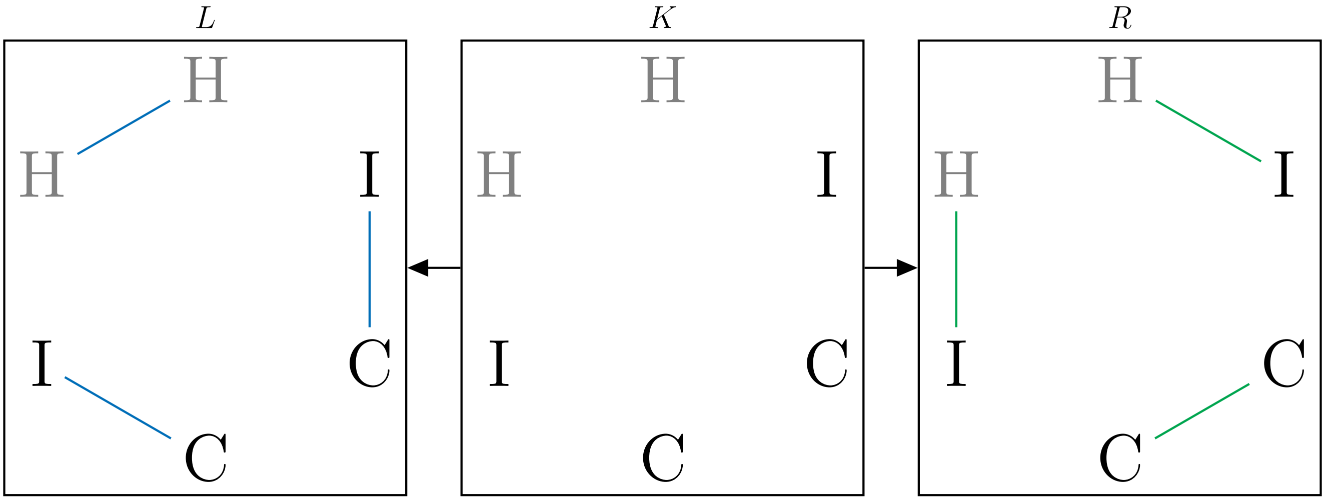

Files: out/933_r_186_11300100_{L, K, R}

0.0.188 187

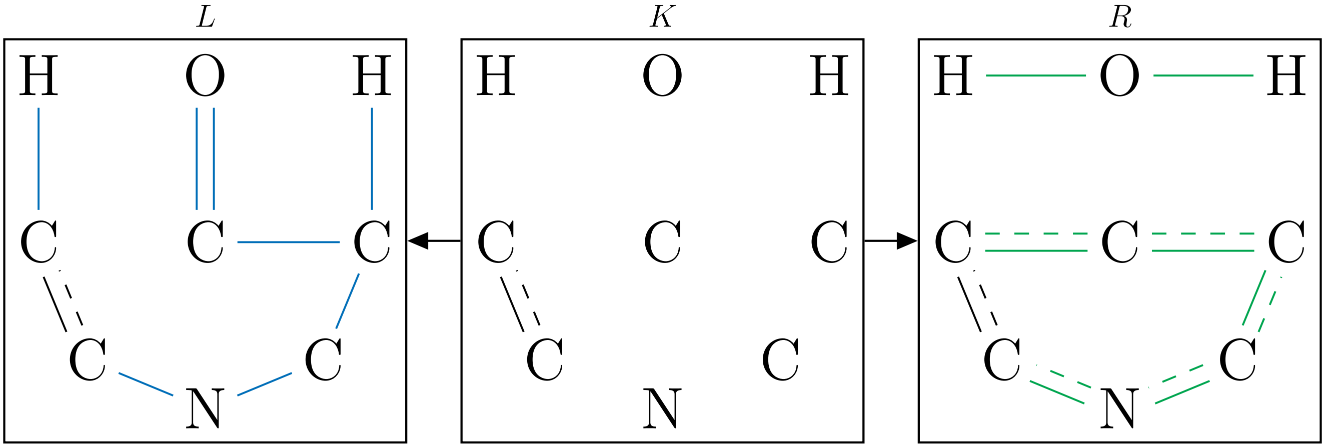

Files: out/936_r_187_10300000_{L, K, R}

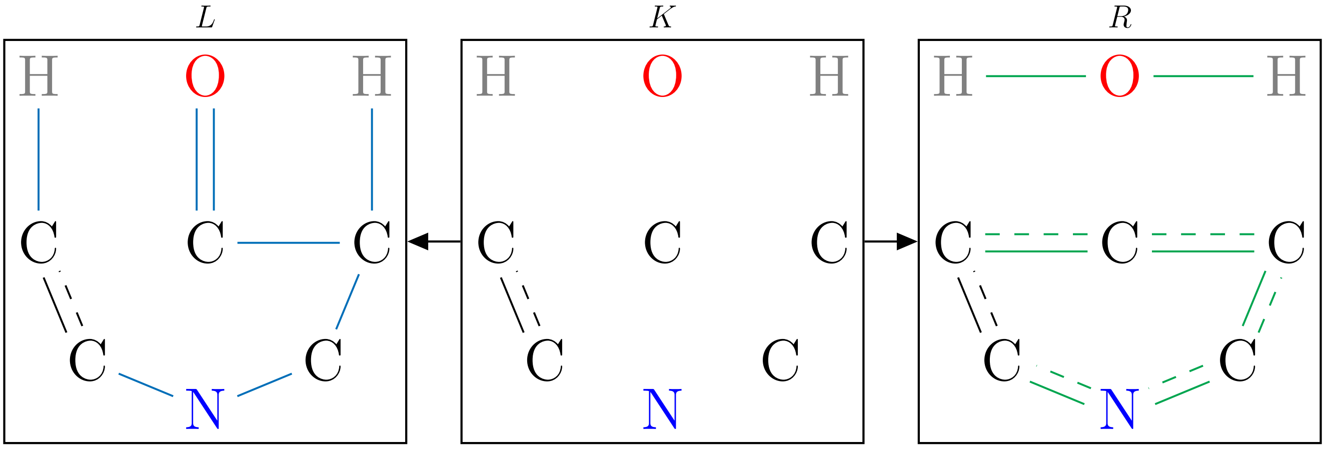

Files: out/938_r_187_11300100_{L, K, R}

0.0.189 188

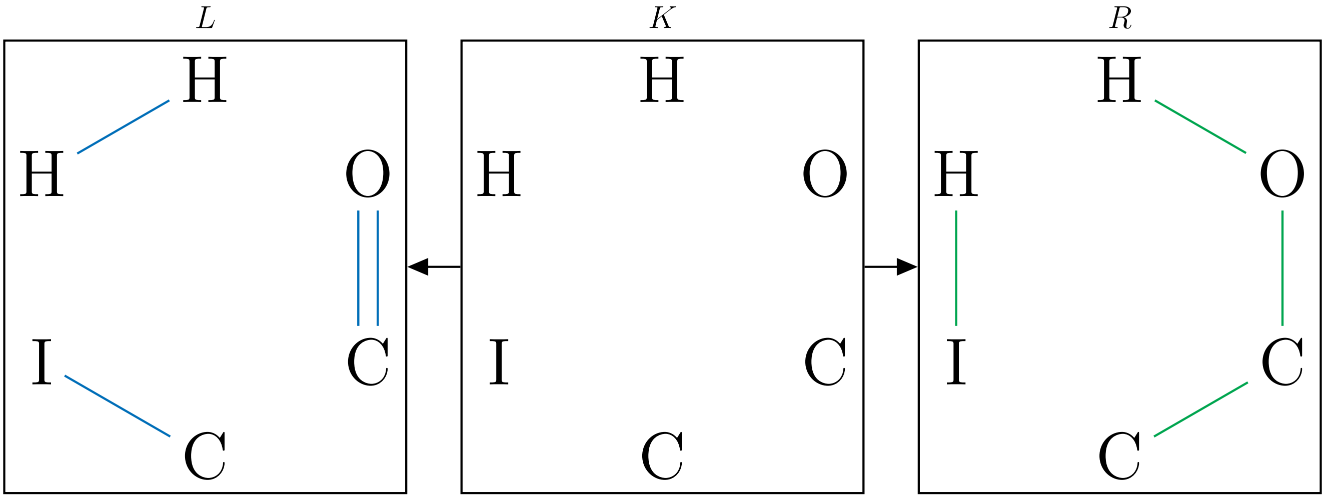

Files: out/941_r_188_10300000_{L, K, R}

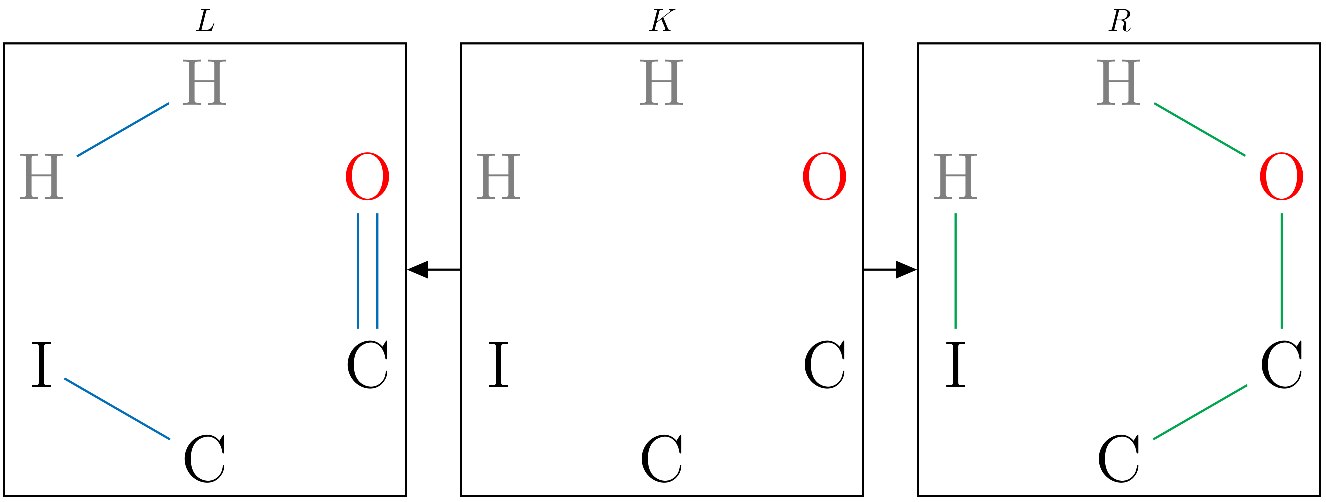

Files: out/943_r_188_11300100_{L, K, R}

0.0.190 189

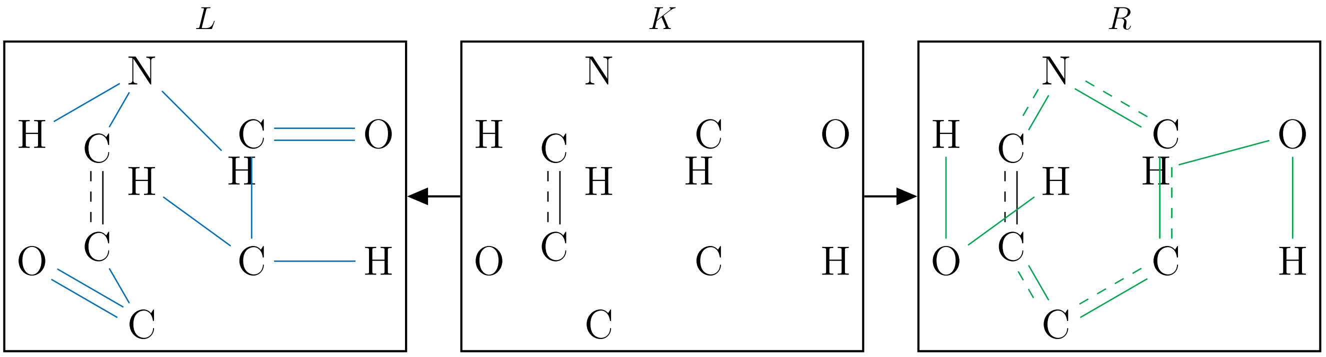

Files: out/946_r_189_10300000_{L, K, R}

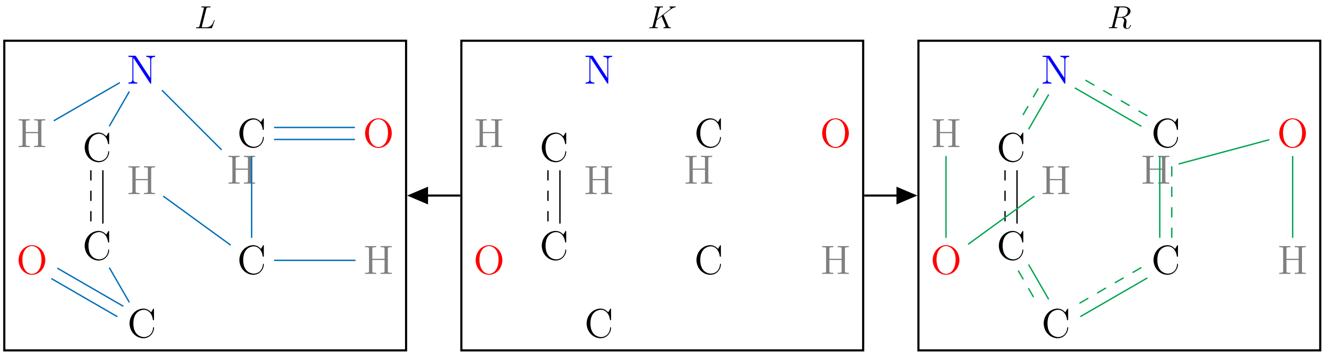

Files: out/948_r_189_11300100_{L, K, R}

0.0.191 190

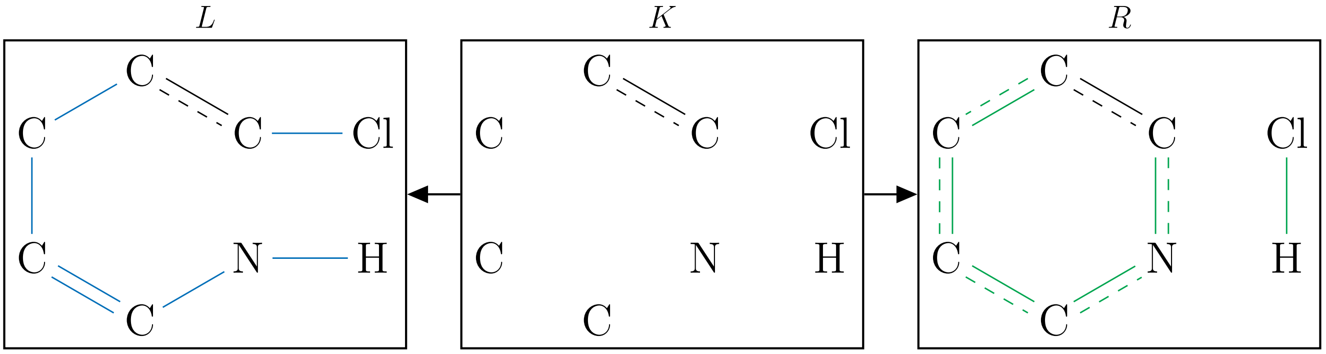

Files: out/951_r_190_10300000_{L, K, R}

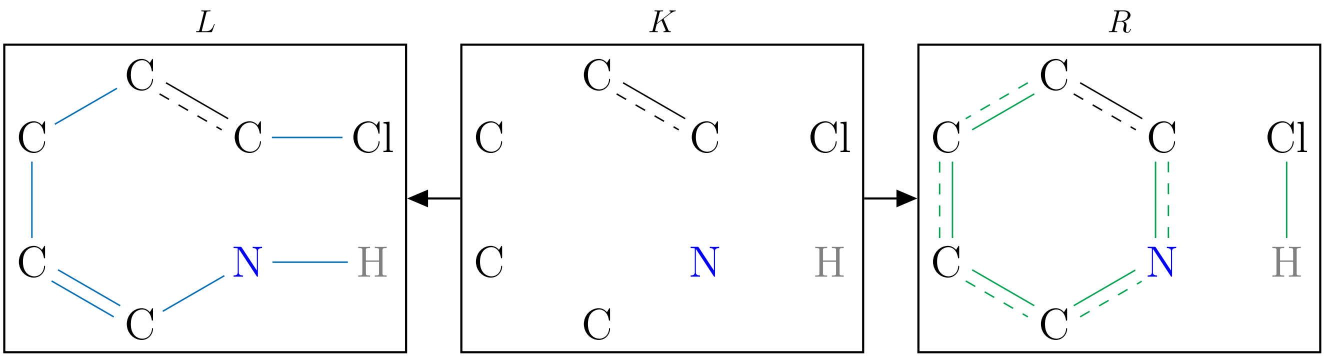

Files: out/953_r_190_11300100_{L, K, R}

0.0.192 191

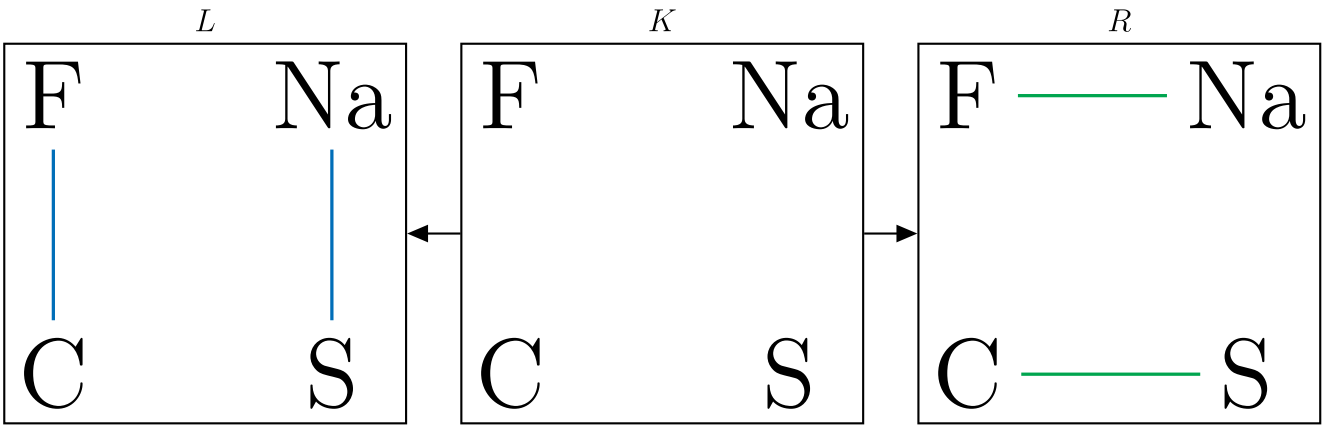

Files: out/956_r_191_10300000_{L, K, R}

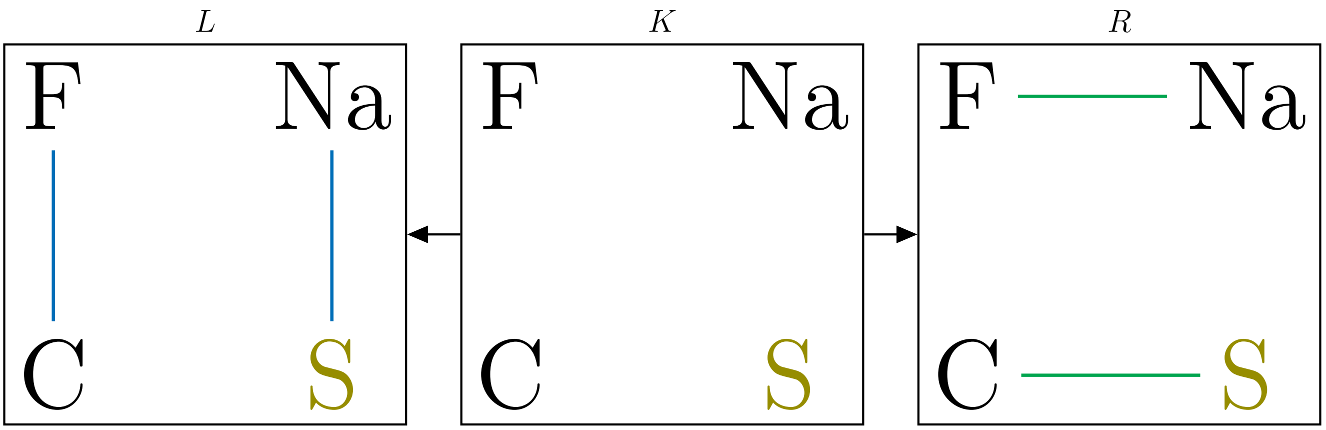

Files: out/958_r_191_11300100_{L, K, R}

0.0.193 192

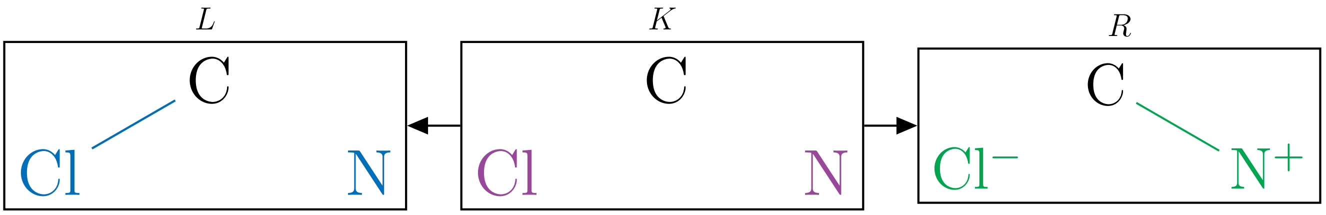

Files: out/961_r_192_10300000_{L, K, R}

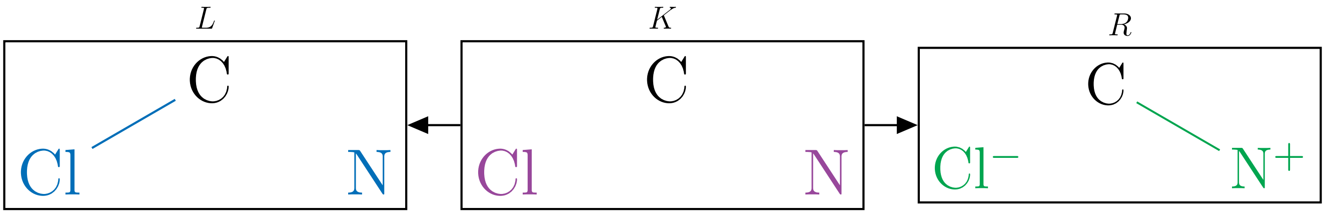

Files: out/963_r_192_11300100_{L, K, R}

0.0.194 193

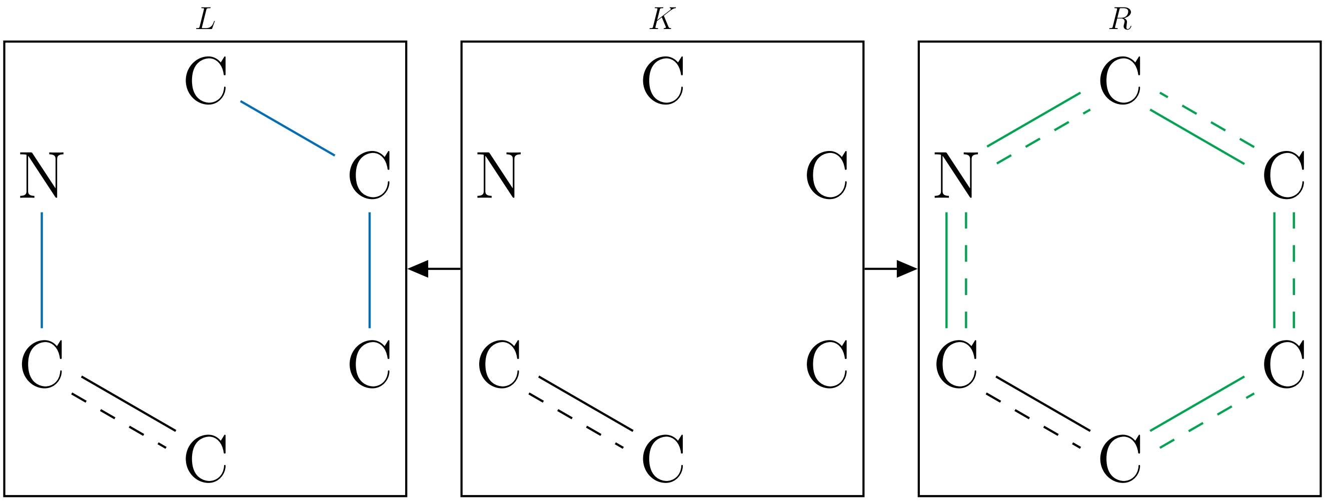

Files: out/966_r_193_10300000_{L, K, R}

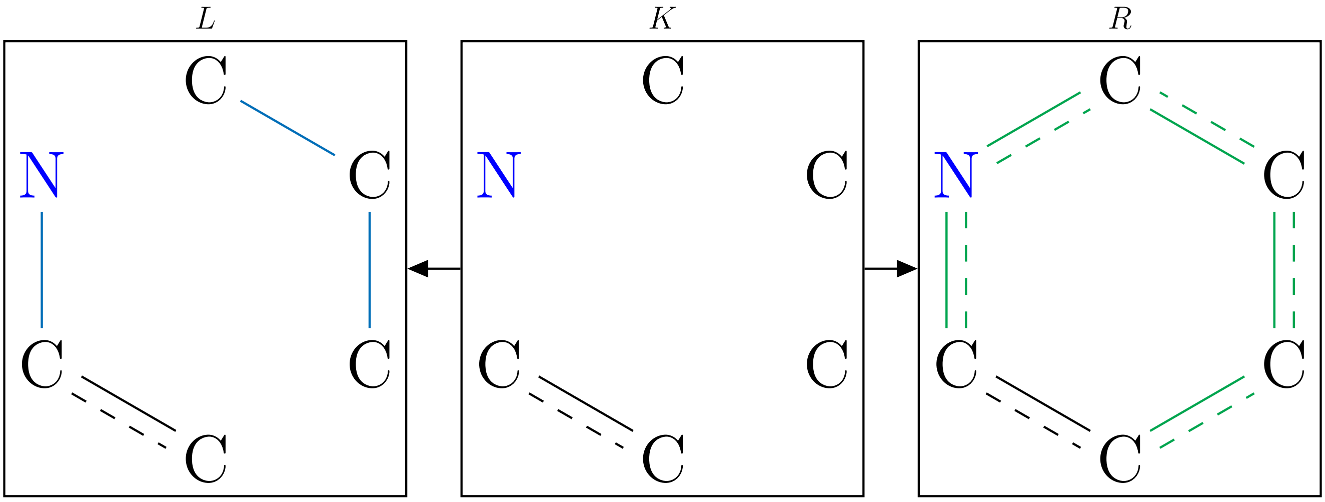

Files: out/968_r_193_11300100_{L, K, R}

0.0.195 194

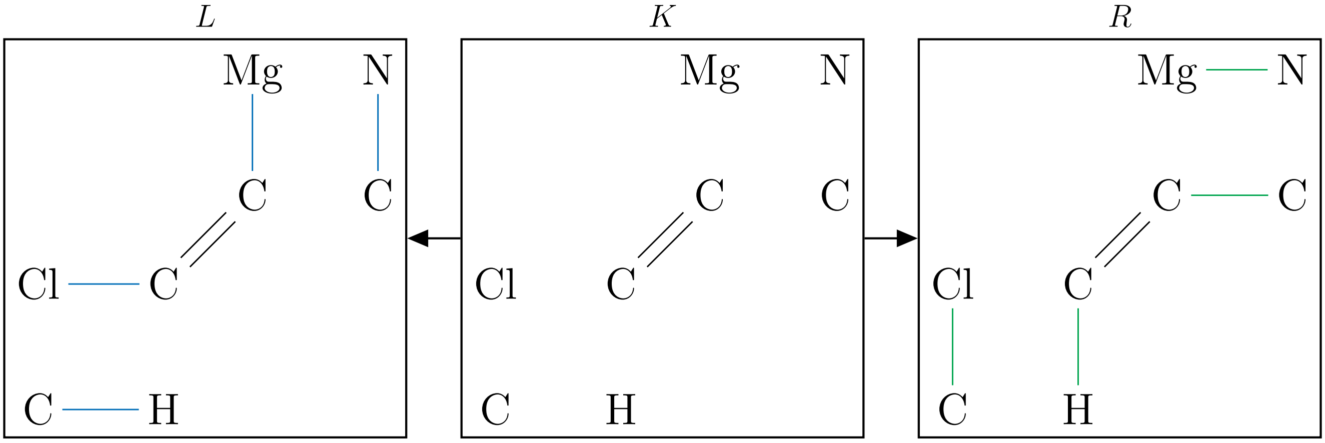

Files: out/971_r_194_10300000_{L, K, R}

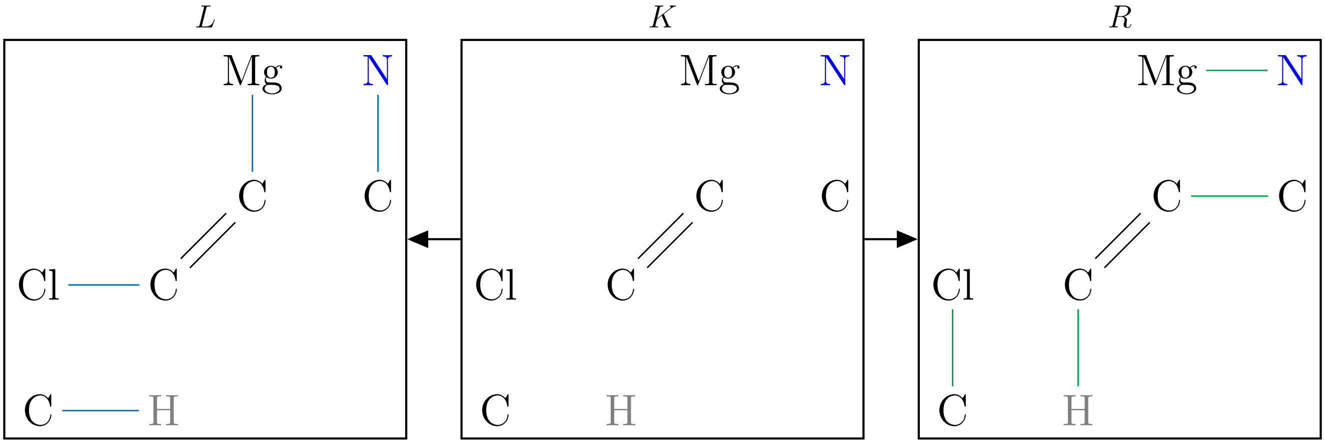

Files: out/973_r_194_11300100_{L, K, R}

0.0.196 195

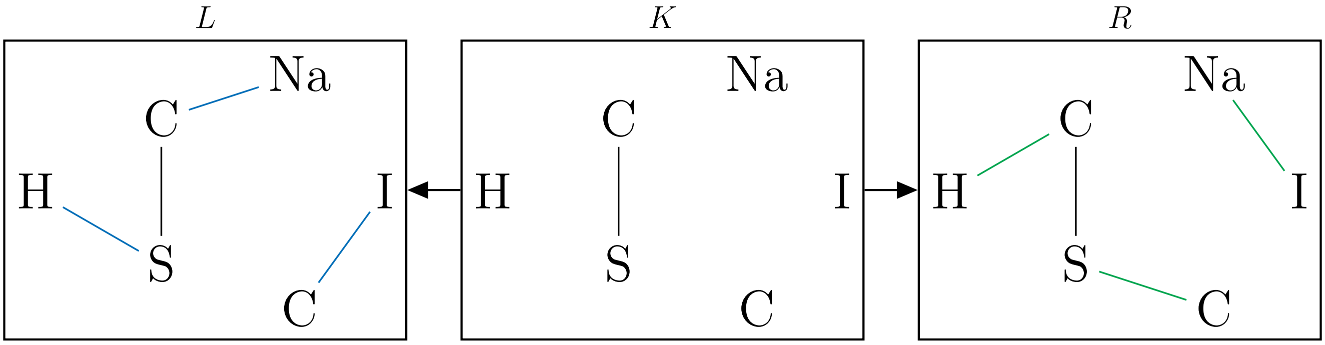

Files: out/976_r_195_10300000_{L, K, R}

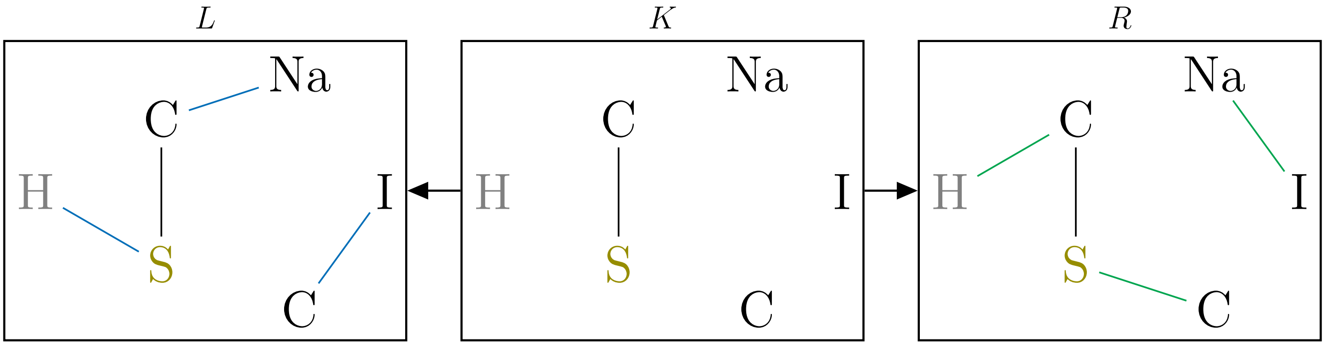

Files: out/978_r_195_11300100_{L, K, R}

0.0.197 196

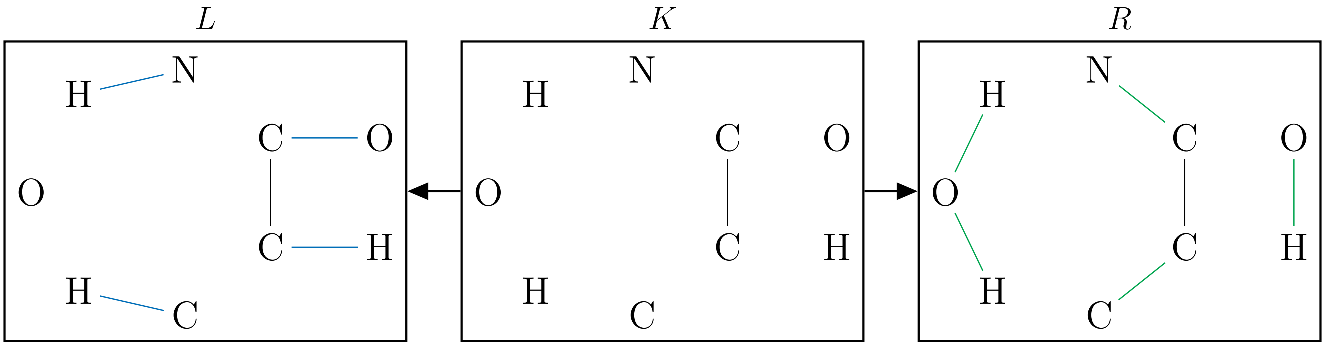

Files: out/981_r_196_10300000_{L, K, R}

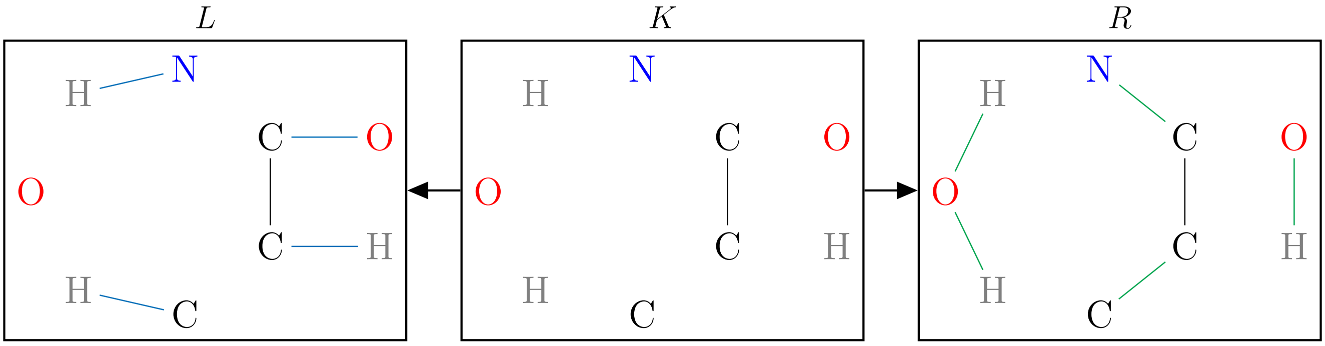

Files: out/983_r_196_11300100_{L, K, R}

0.0.198 197

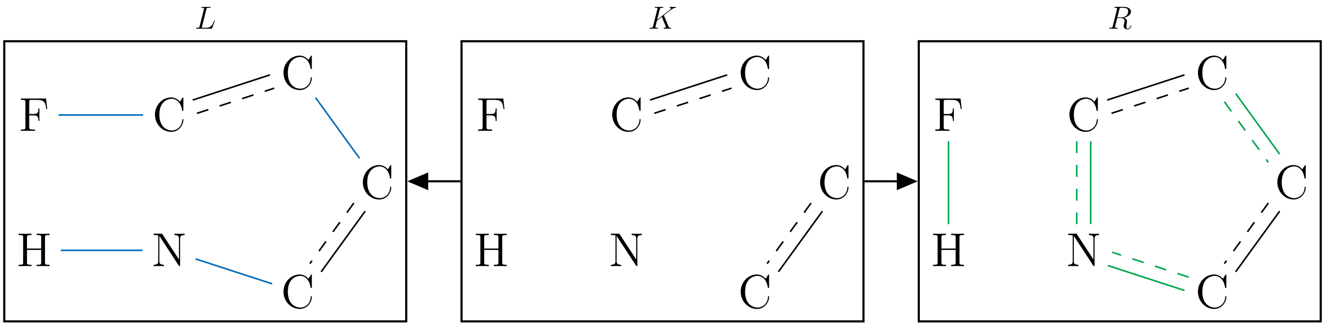

Files: out/986_r_197_10300000_{L, K, R}

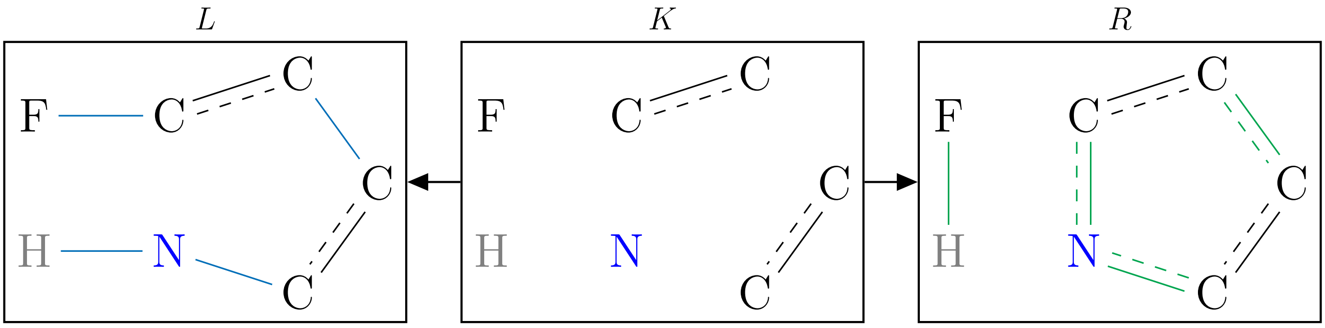

Files: out/988_r_197_11300100_{L, K, R}

0.0.199 198

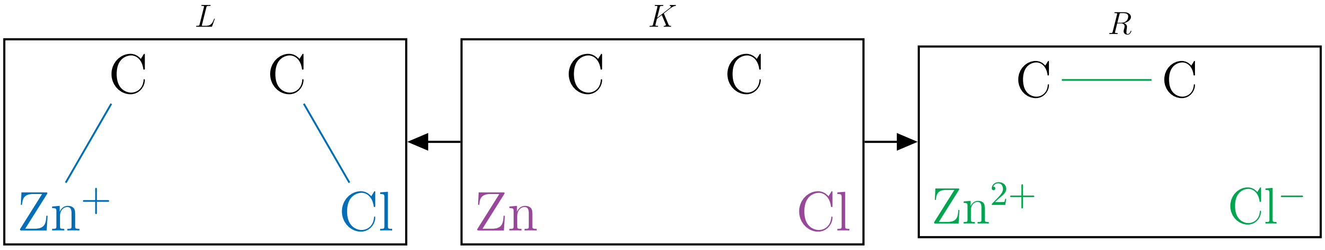

Files: out/991_r_198_10300000_{L, K, R}

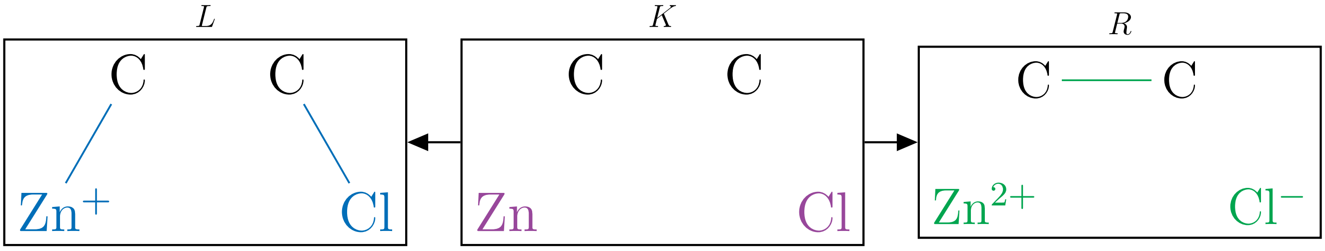

Files: out/993_r_198_11300100_{L, K, R}

0.0.200 199

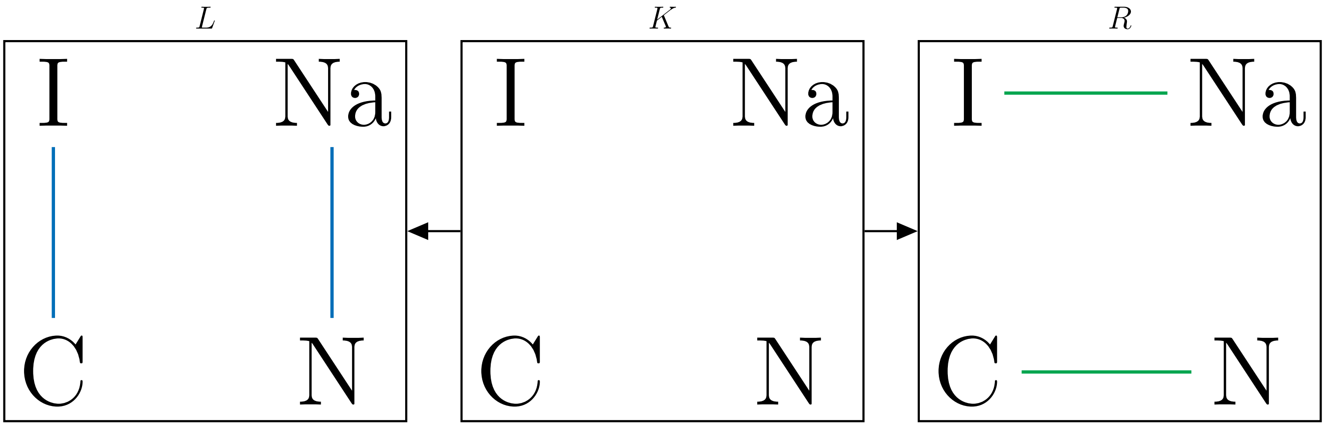

Files: out/996_r_199_10300000_{L, K, R}

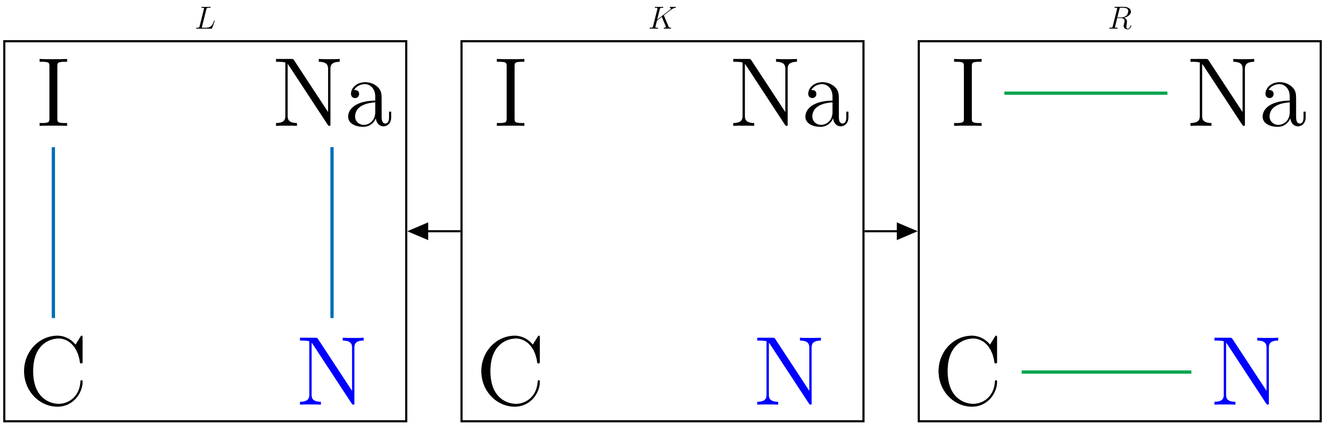

Files: out/998_r_199_11300100_{L, K, R}

0.0.201 200

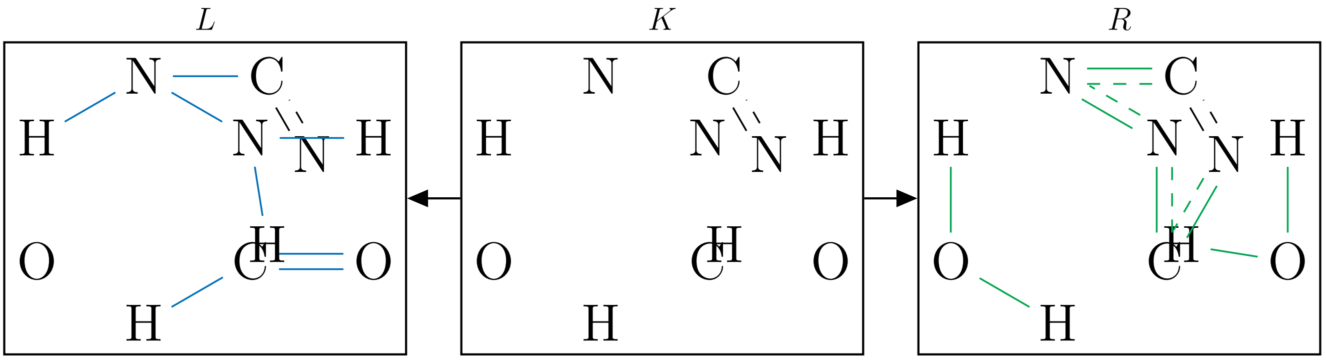

Files: out/1001_r_200_10300000_{L, K, R}

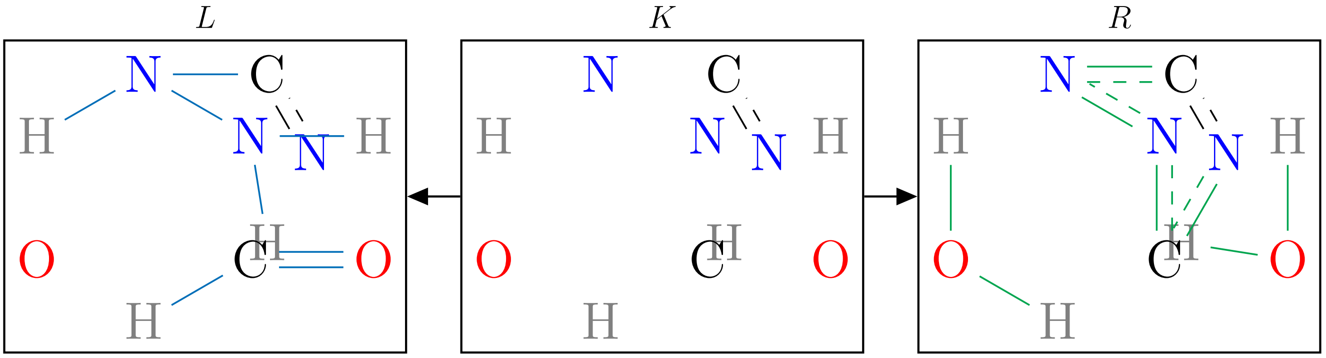

Files: out/1003_r_200_11300100_{L, K, R}

0.0.202 201

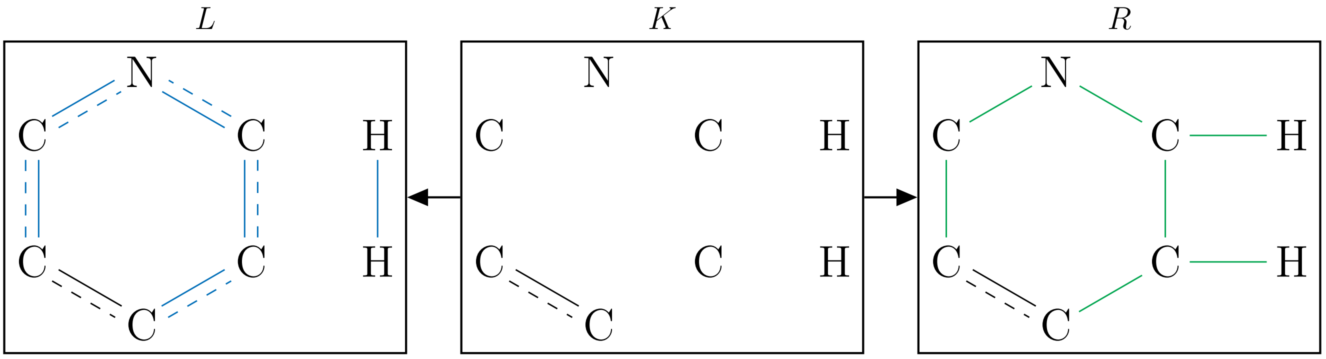

Files: out/1006_r_201_10300000_{L, K, R}

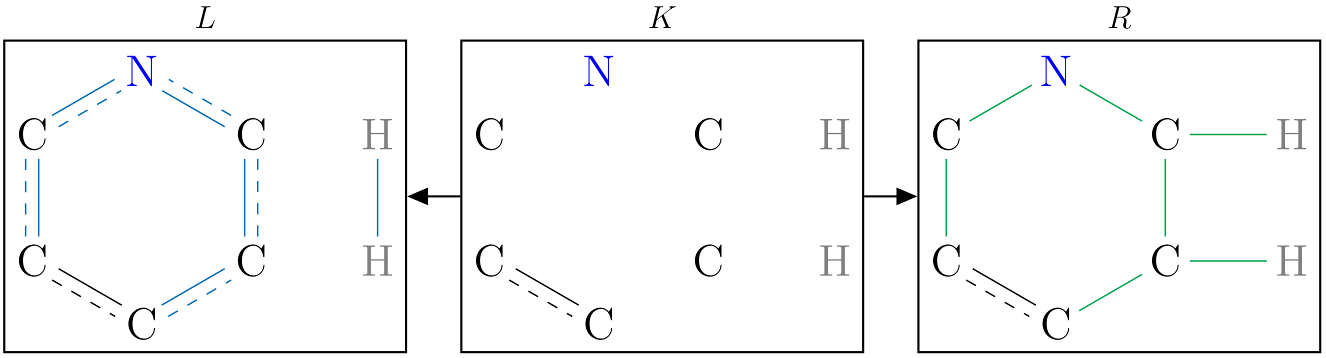

Files: out/1008_r_201_11300100_{L, K, R}

0.0.203 202

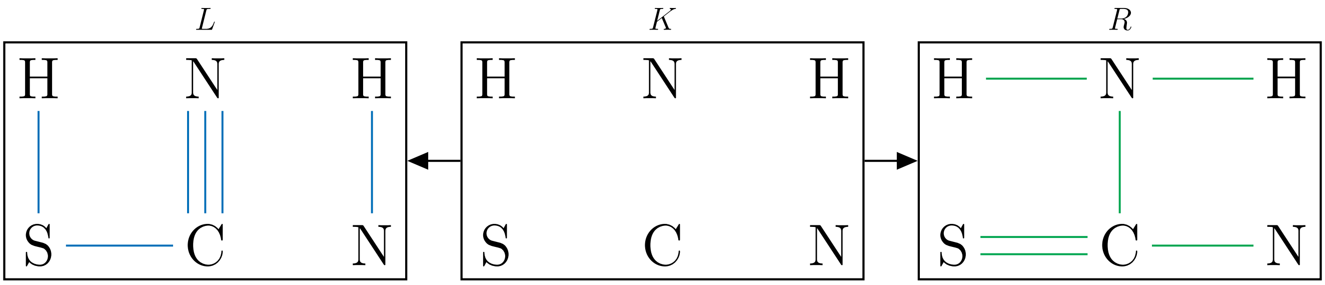

Files: out/1011_r_202_10300000_{L, K, R}

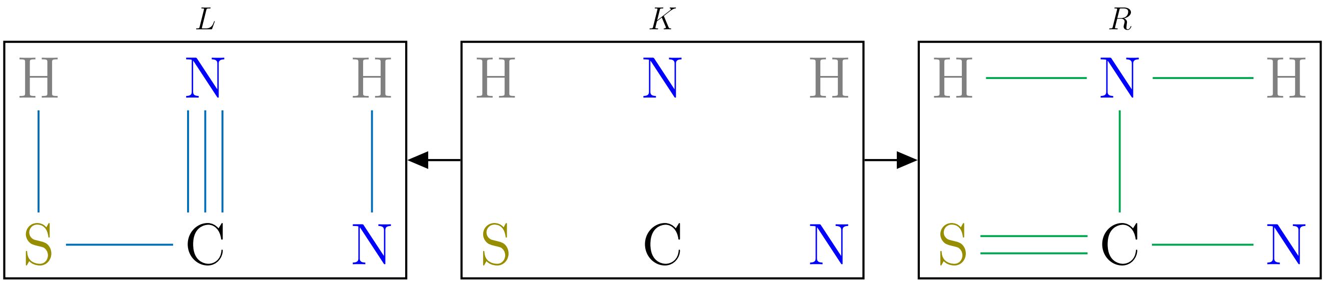

Files: out/1013_r_202_11300100_{L, K, R}

0.0.204 203

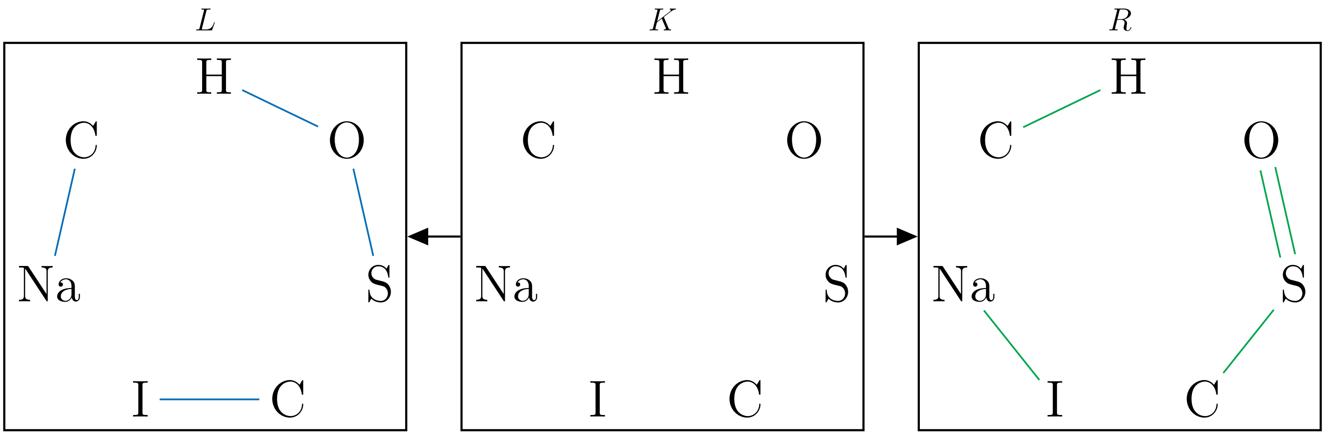

Files: out/1016_r_203_10300000_{L, K, R}

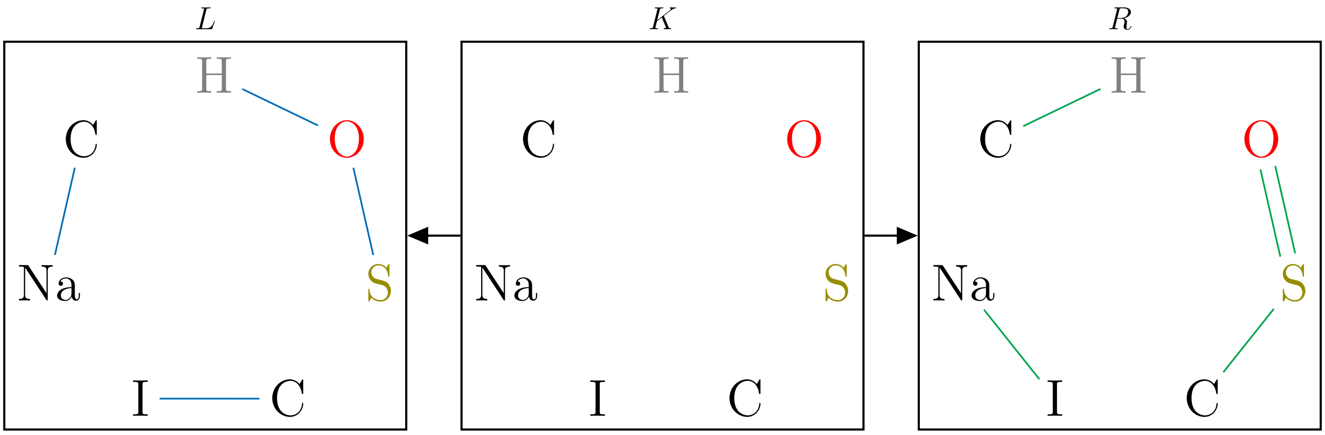

Files: out/1018_r_203_11300100_{L, K, R}

0.0.205 204

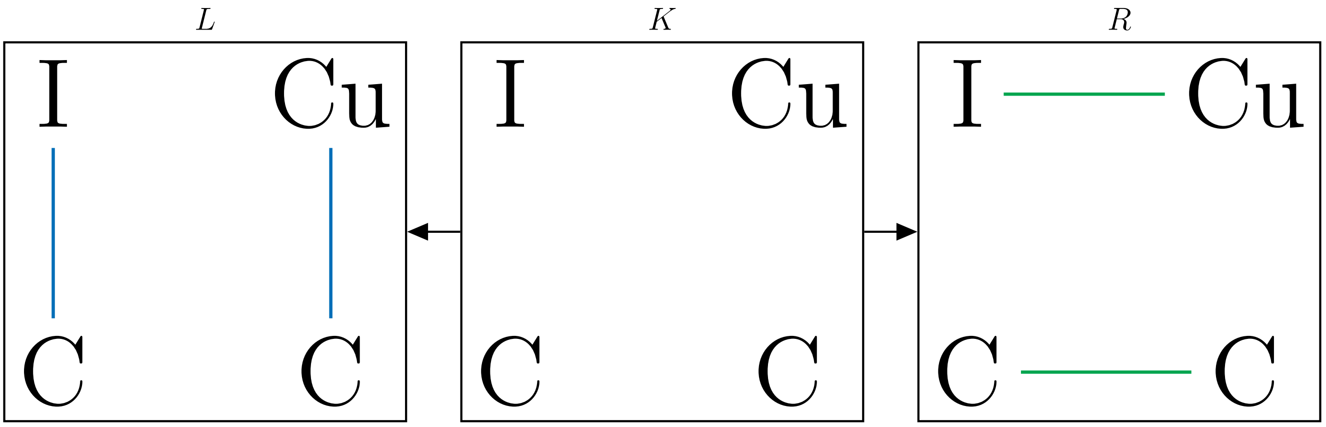

Files: out/1021_r_204_10300000_{L, K, R}

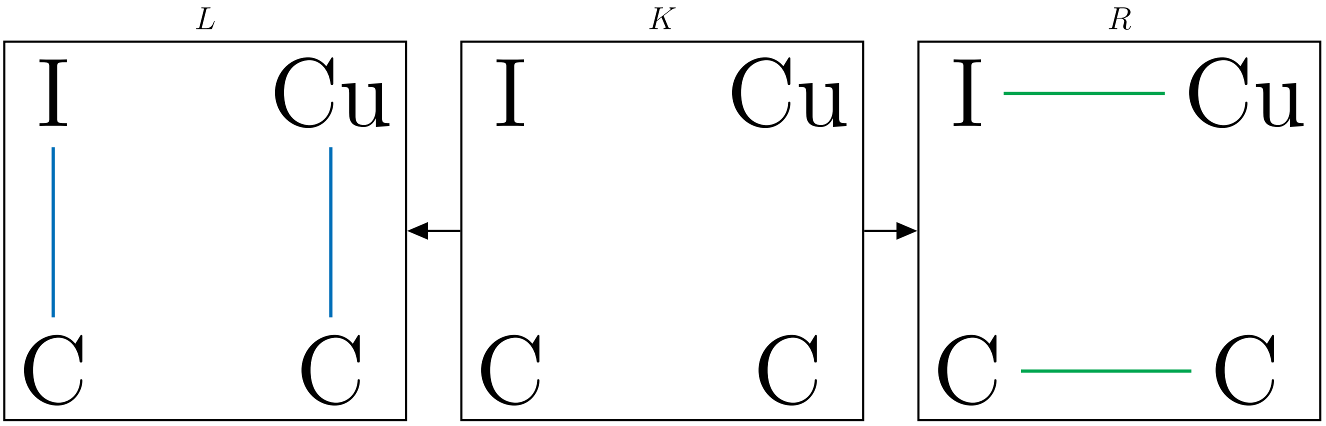

Files: out/1023_r_204_11300100_{L, K, R}

0.0.206 205

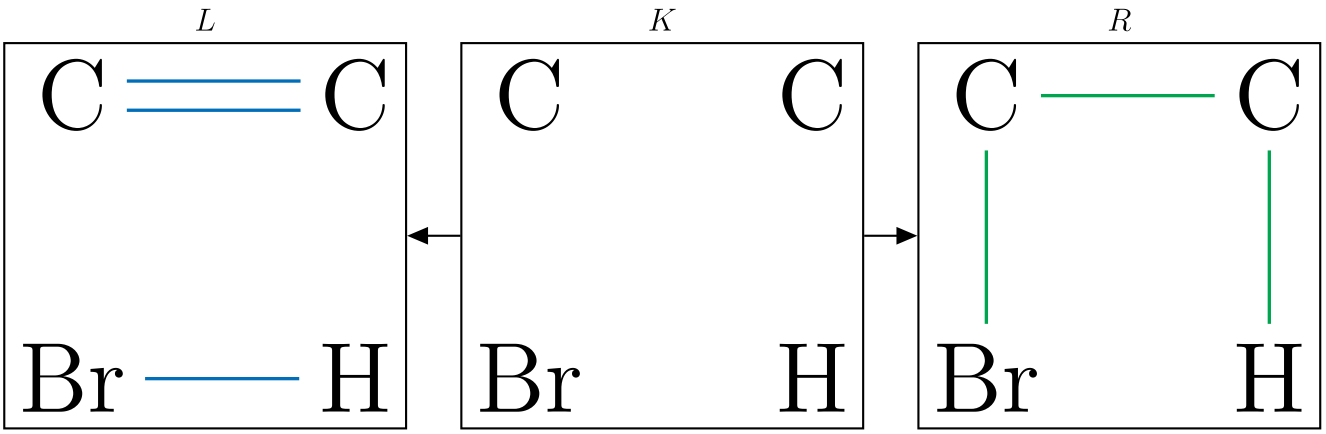

Files: out/1026_r_205_10300000_{L, K, R}

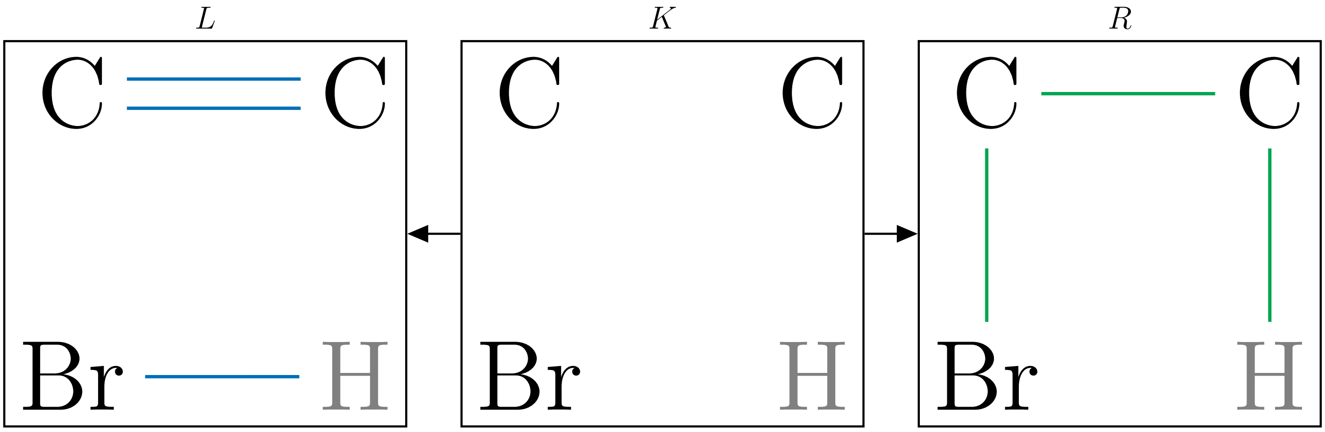

Files: out/1028_r_205_11300100_{L, K, R}

0.0.207 206

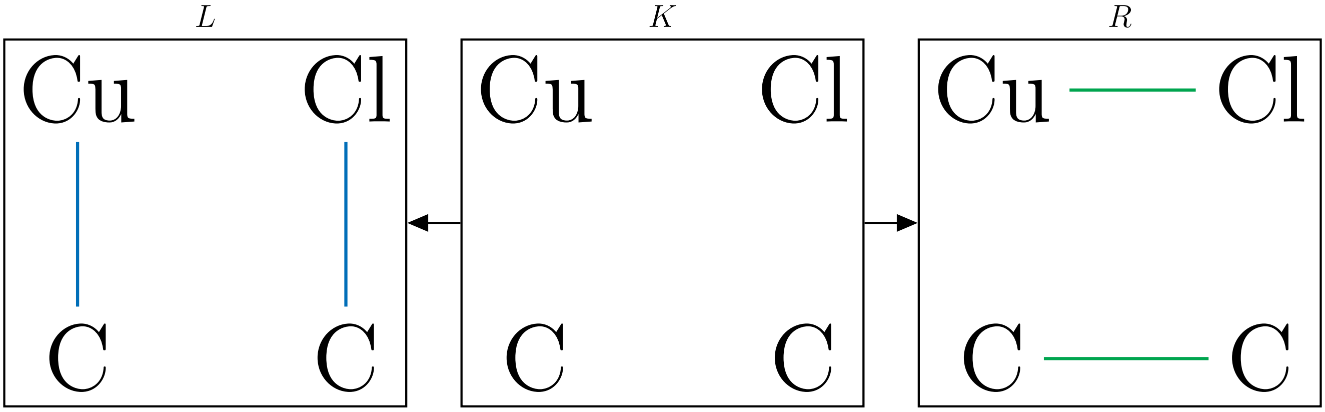

Files: out/1031_r_206_10300000_{L, K, R}

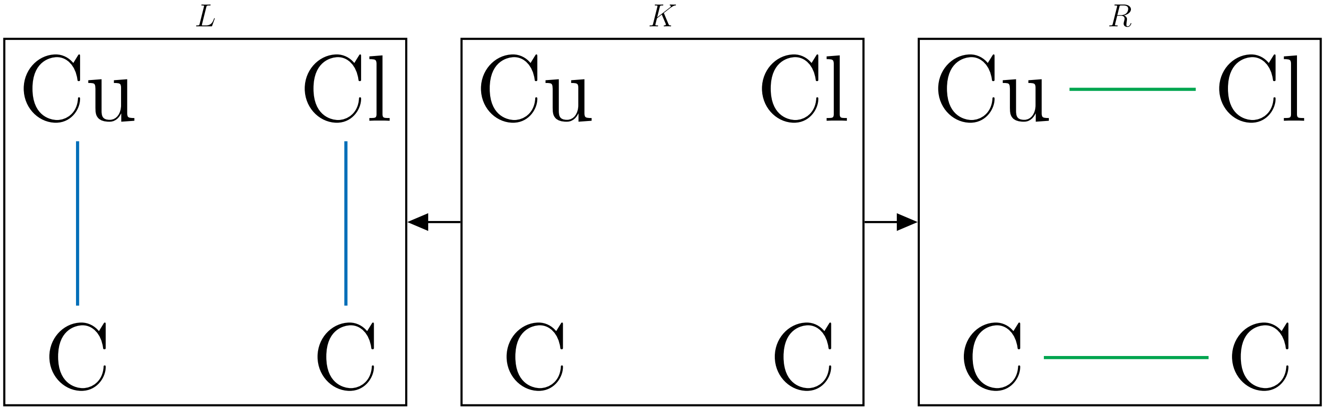

Files: out/1033_r_206_11300100_{L, K, R}

0.0.208 207

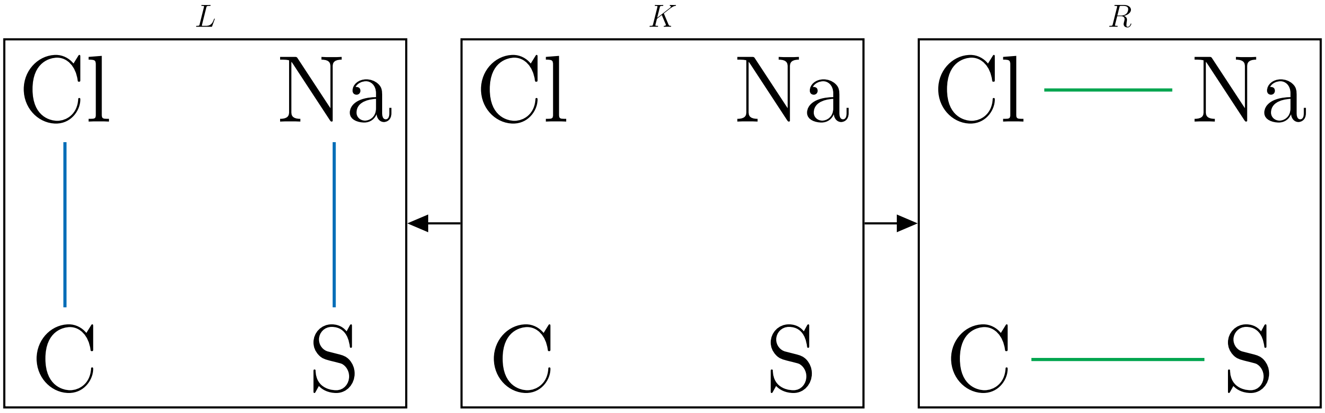

Files: out/1036_r_207_10300000_{L, K, R}

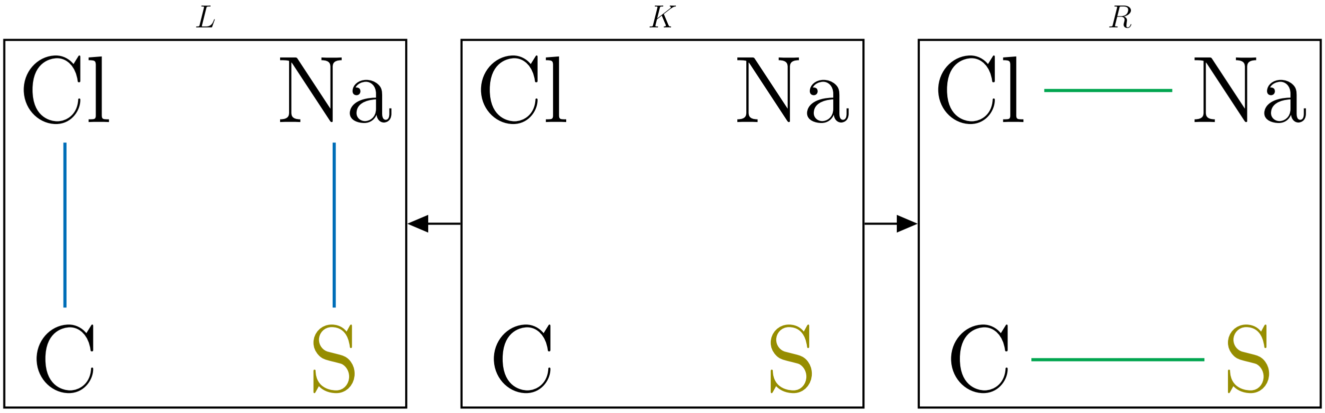

Files: out/1038_r_207_11300100_{L, K, R}

0.0.209 208

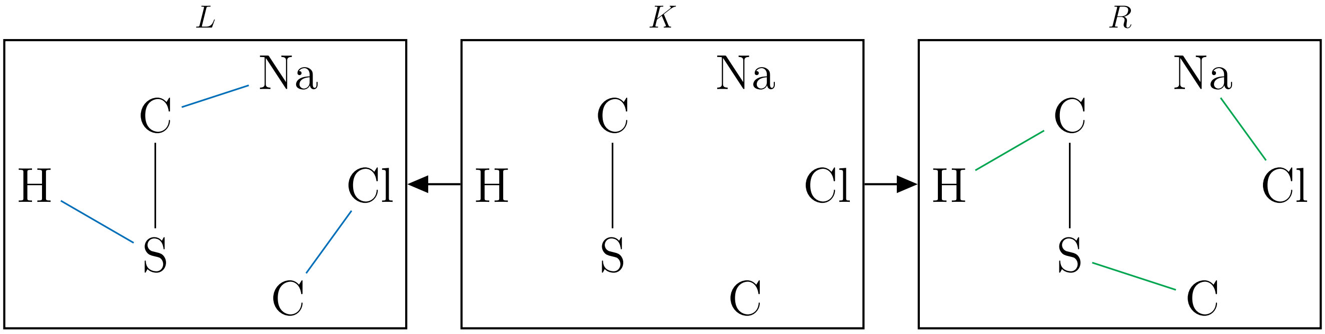

Files: out/1041_r_208_10300000_{L, K, R}

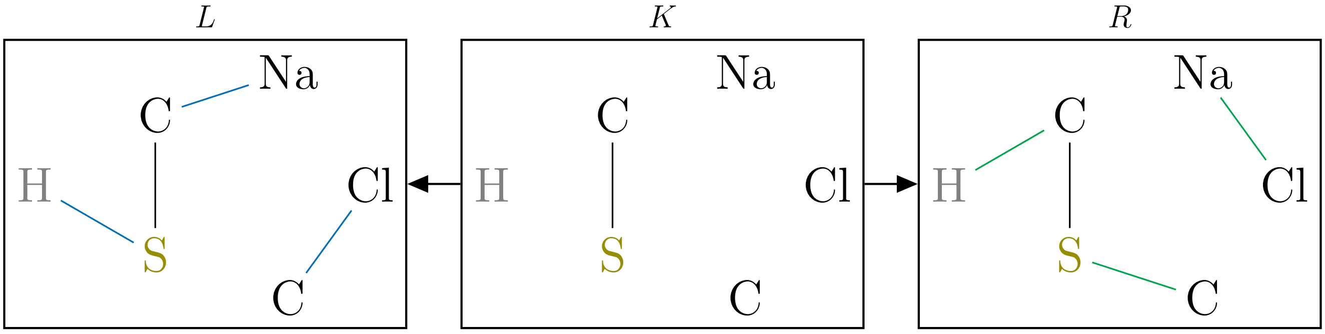

Files: out/1043_r_208_11300100_{L, K, R}

0.0.210 209

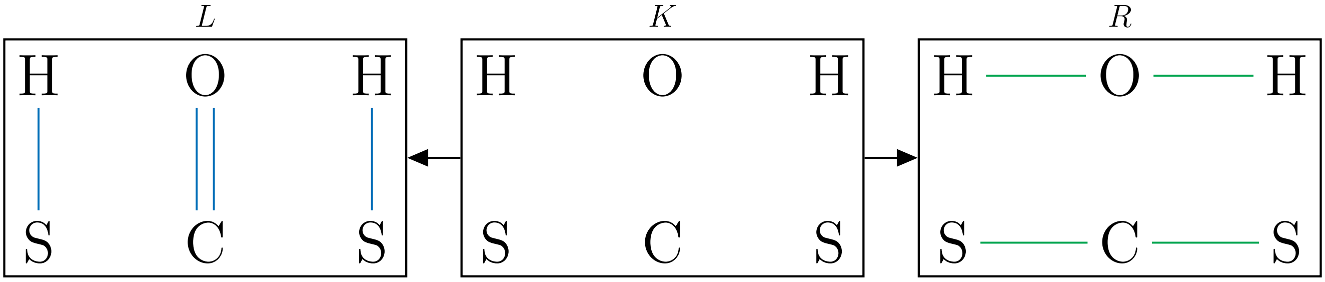

Files: out/1046_r_209_10300000_{L, K, R}

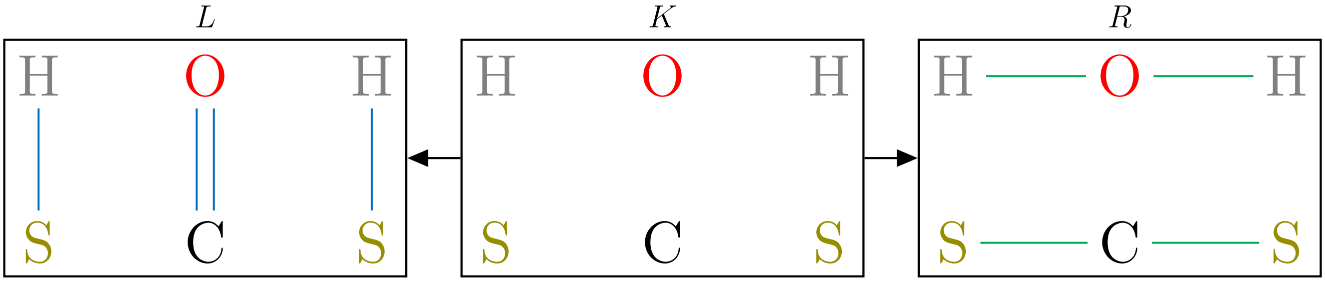

Files: out/1048_r_209_11300100_{L, K, R}

0.0.211 210

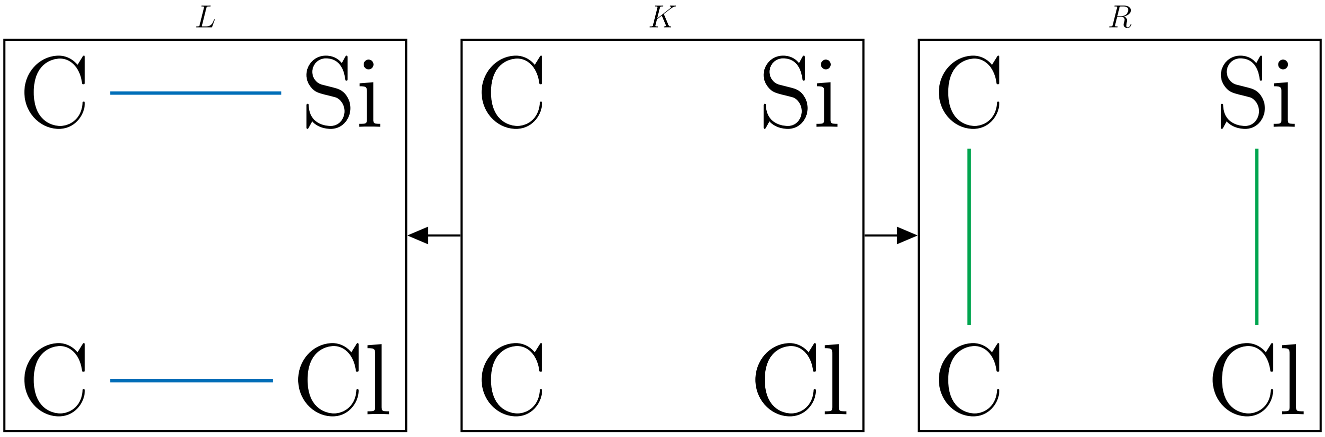

Files: out/1051_r_210_10300000_{L, K, R}

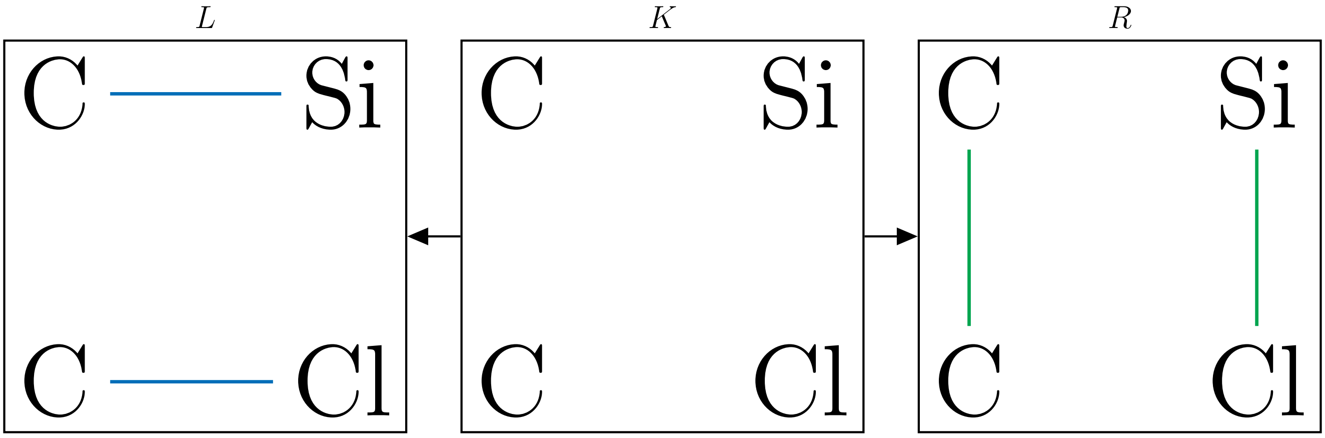

Files: out/1053_r_210_11300100_{L, K, R}

0.0.212 211

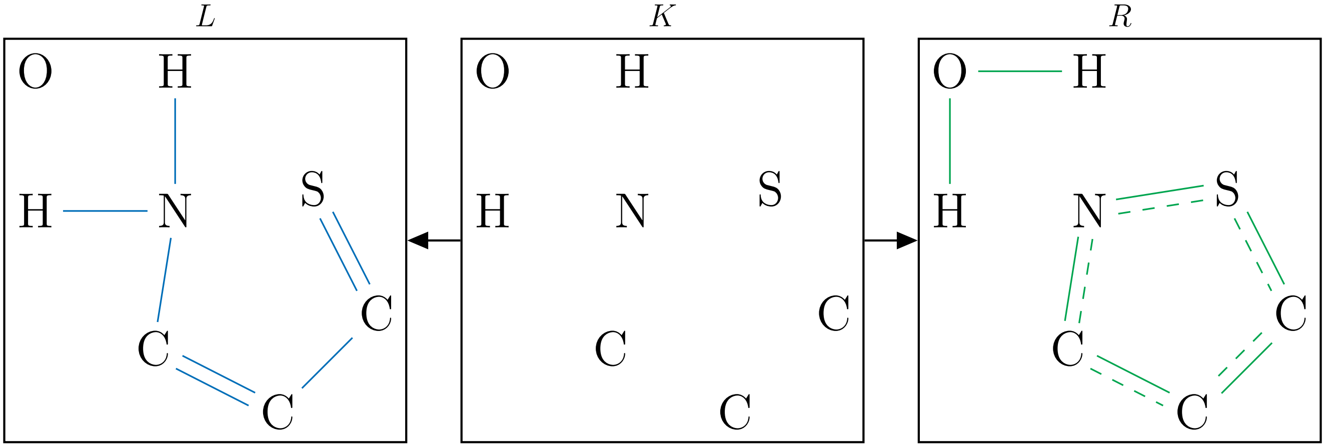

Files: out/1056_r_211_10300000_{L, K, R}

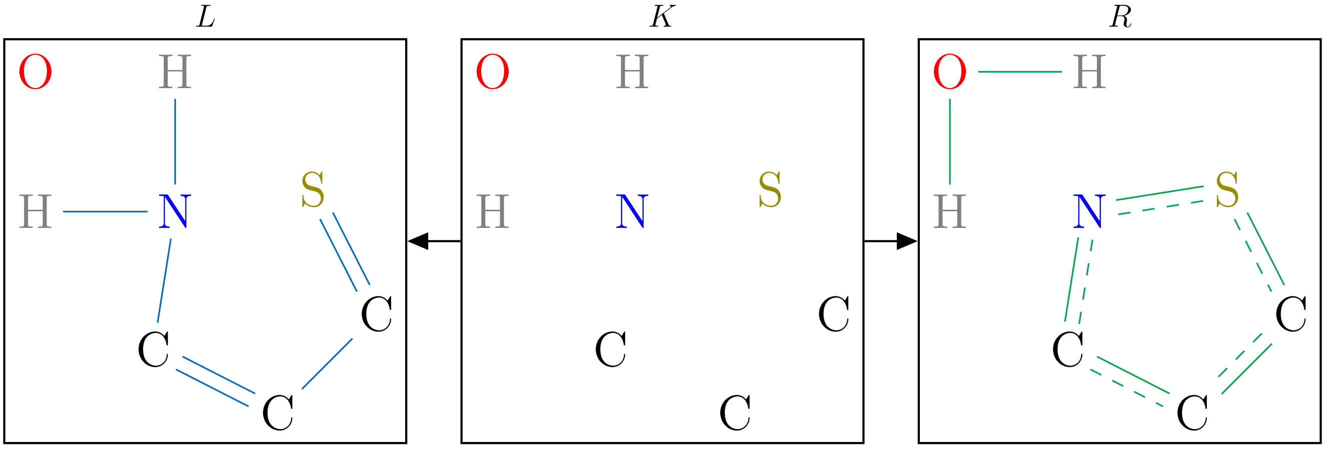

Files: out/1058_r_211_11300100_{L, K, R}

0.0.213 212

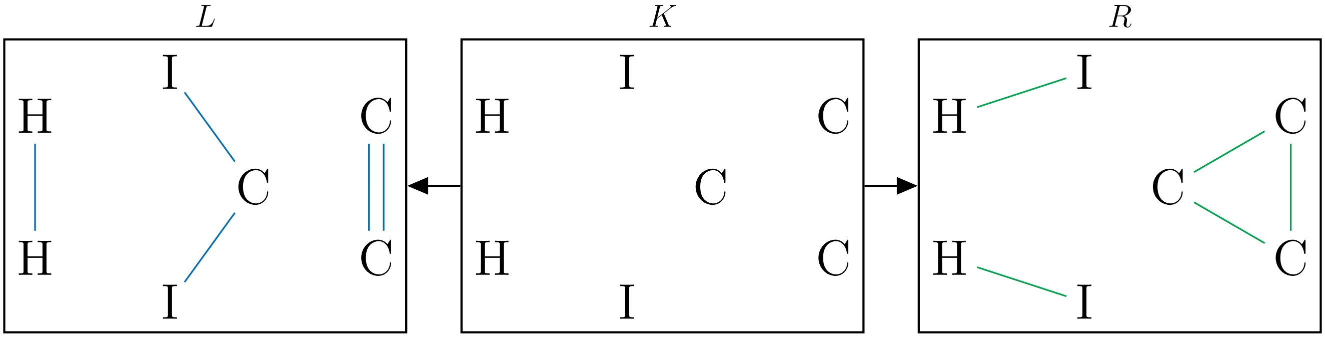

Files: out/1061_r_212_10300000_{L, K, R}

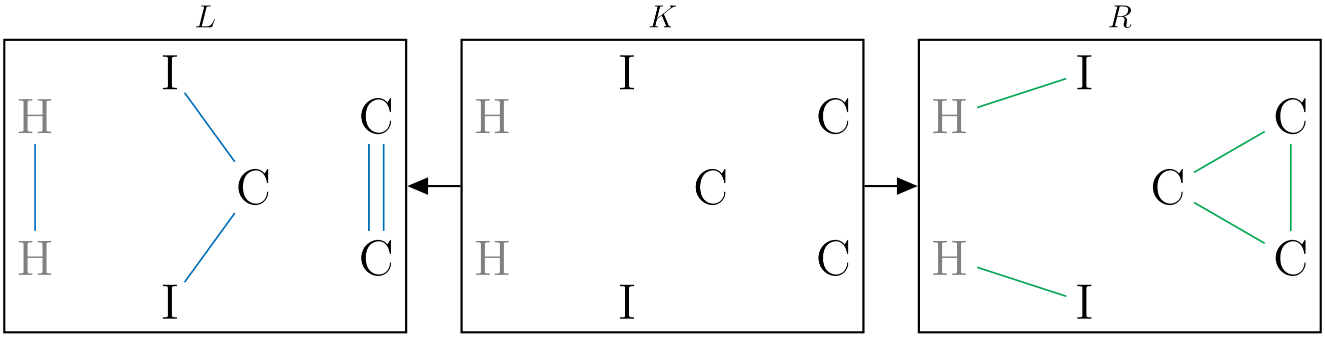

0.0.214 213

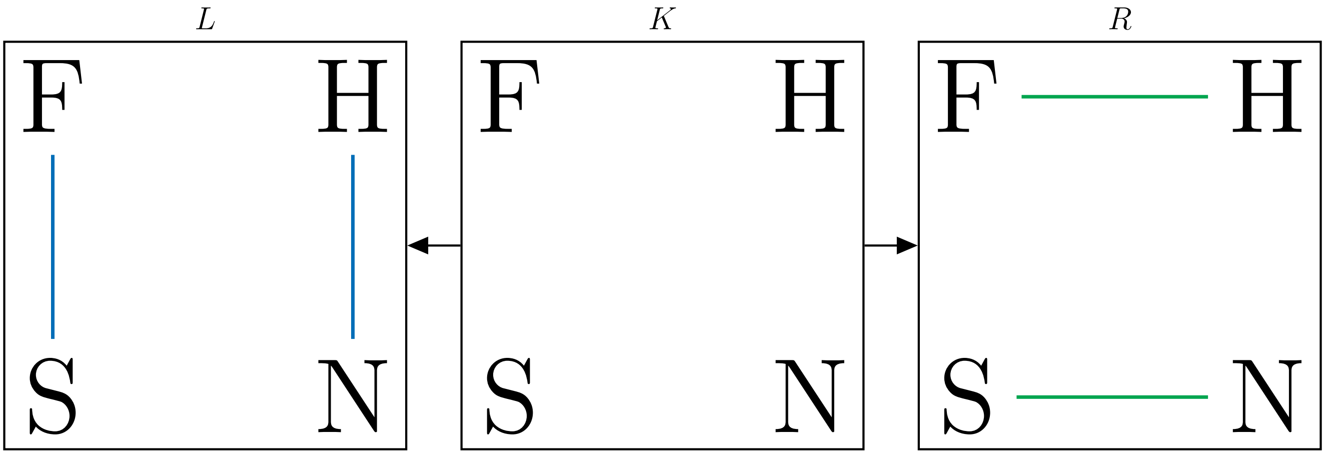

Files: out/1066_r_213_10300000_{L, K, R}

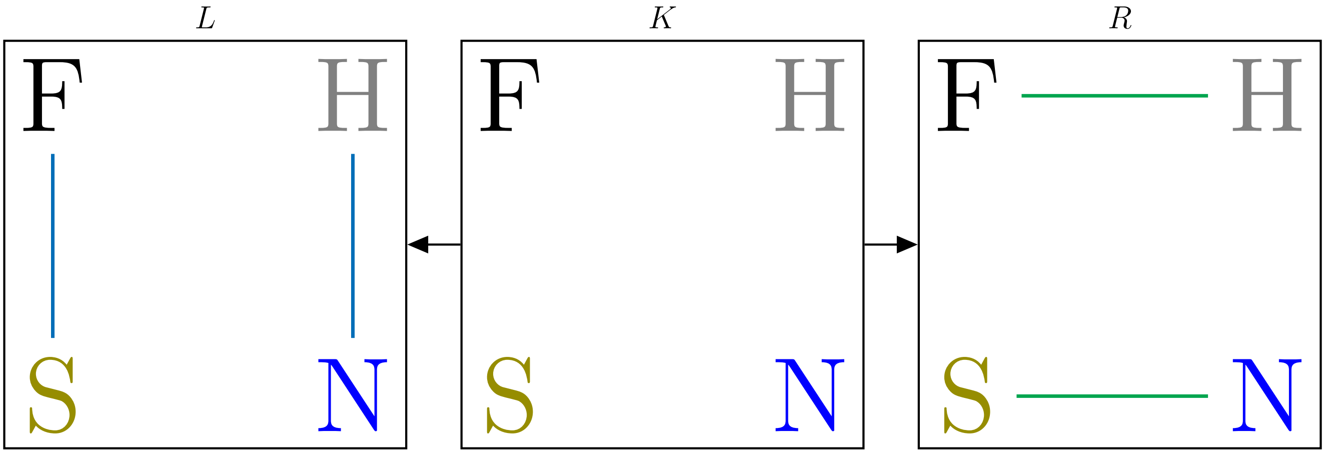

Files: out/1068_r_213_11300100_{L, K, R}

0.0.215 214

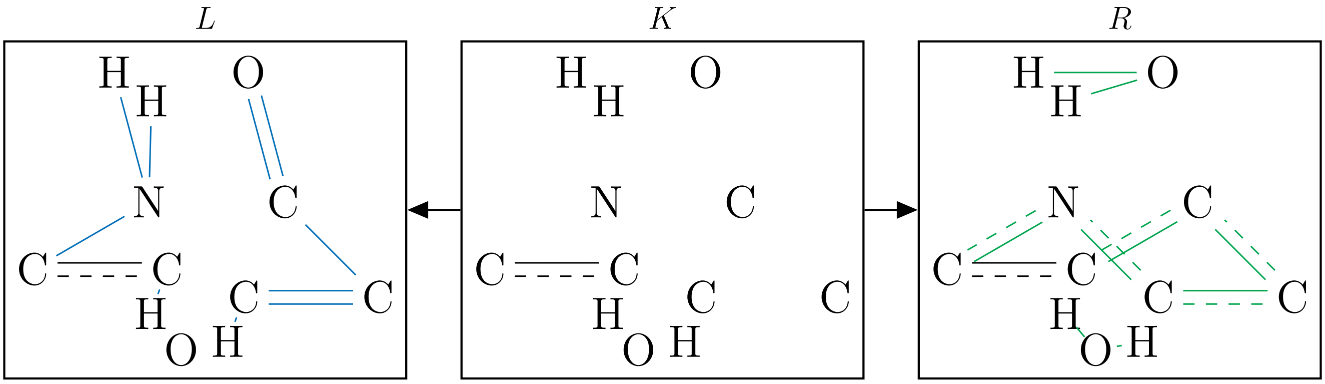

Files: out/1071_r_214_10300000_{L, K, R}

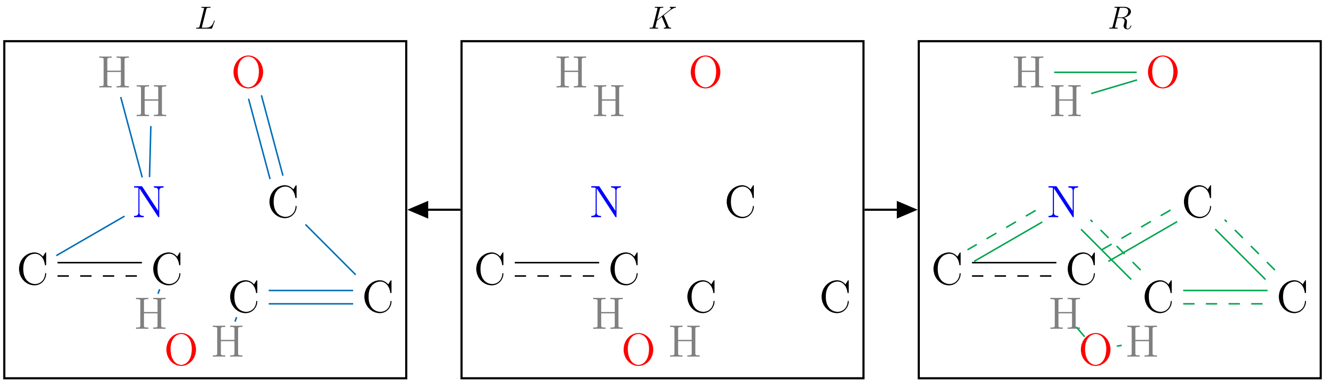

Files: out/1073_r_214_11300100_{L, K, R}

0.0.216 215

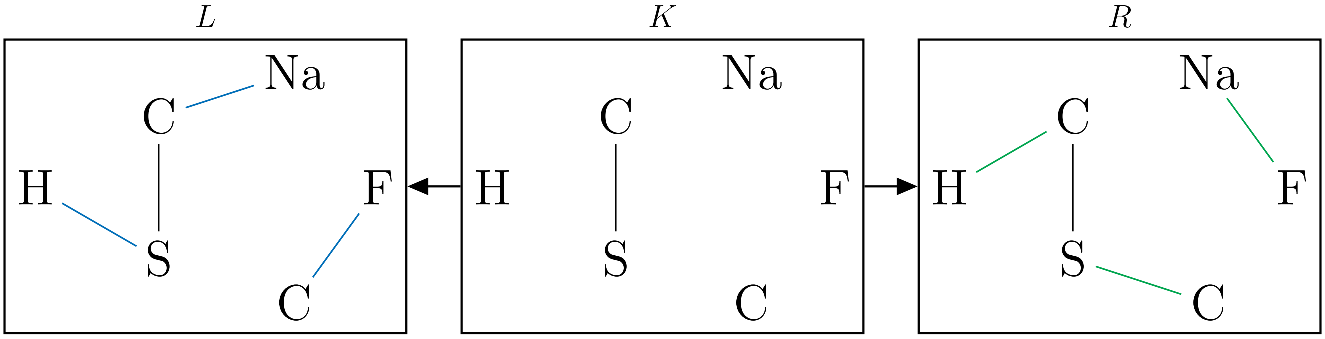

Files: out/1076_r_215_10300000_{L, K, R}

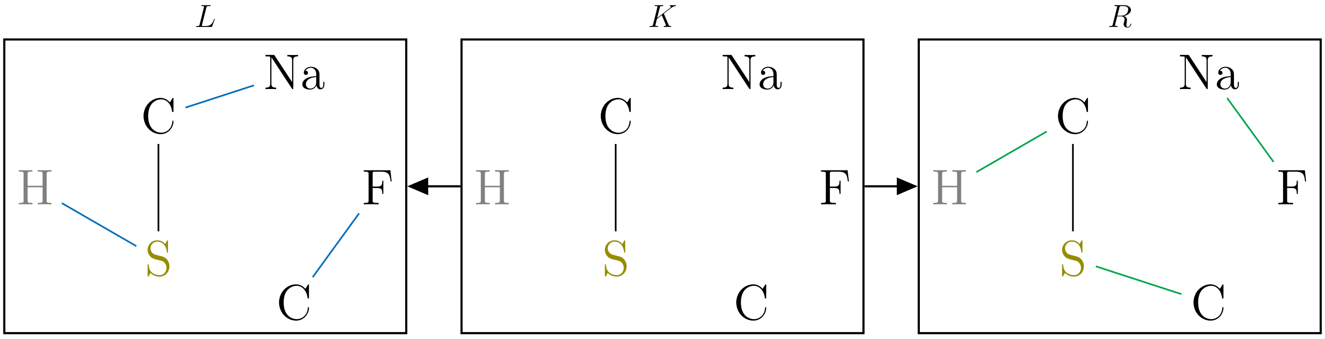

Files: out/1078_r_215_11300100_{L, K, R}

0.0.217 216

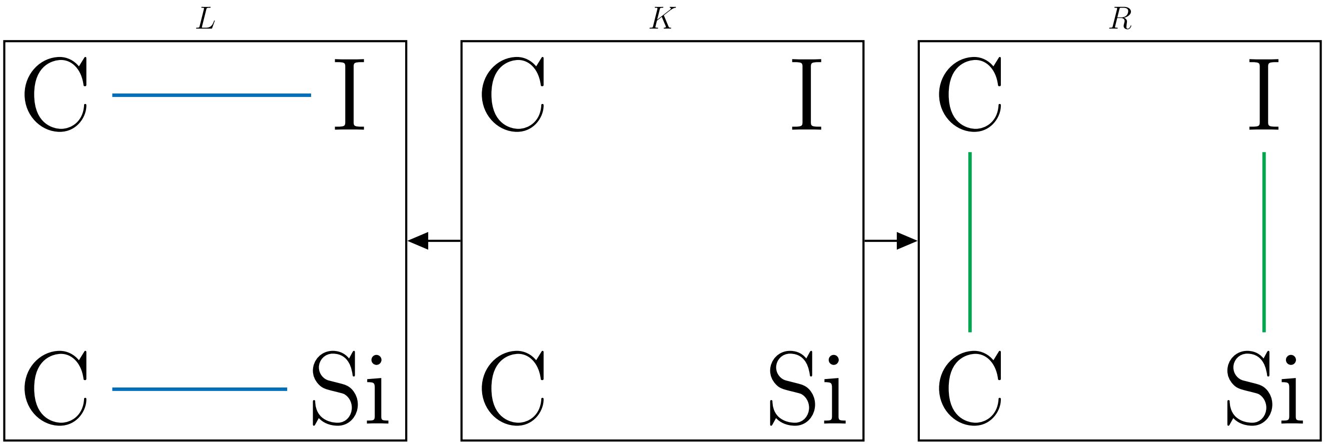

Files: out/1081_r_216_10300000_{L, K, R}

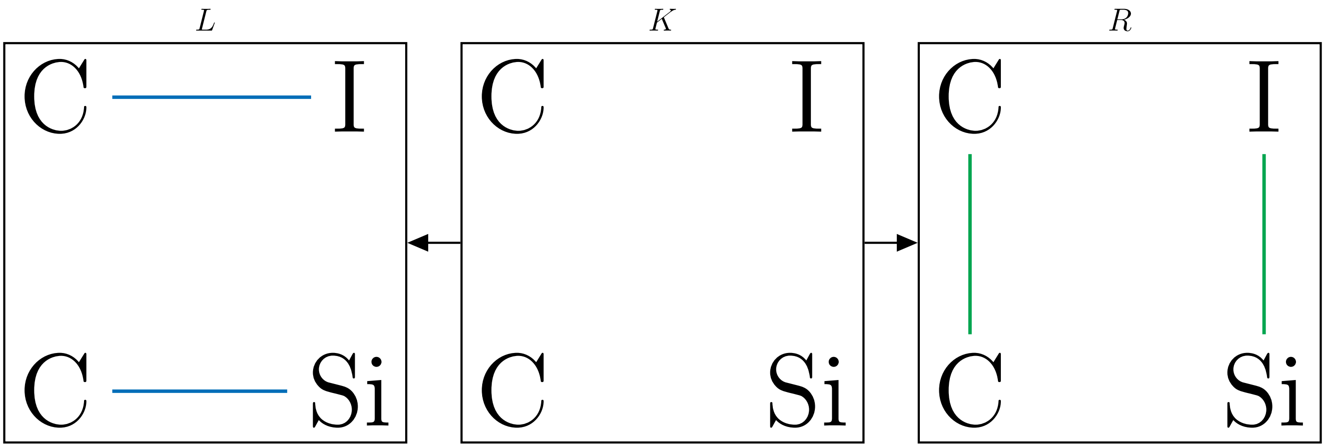

Files: out/1083_r_216_11300100_{L, K, R}

0.0.218 217

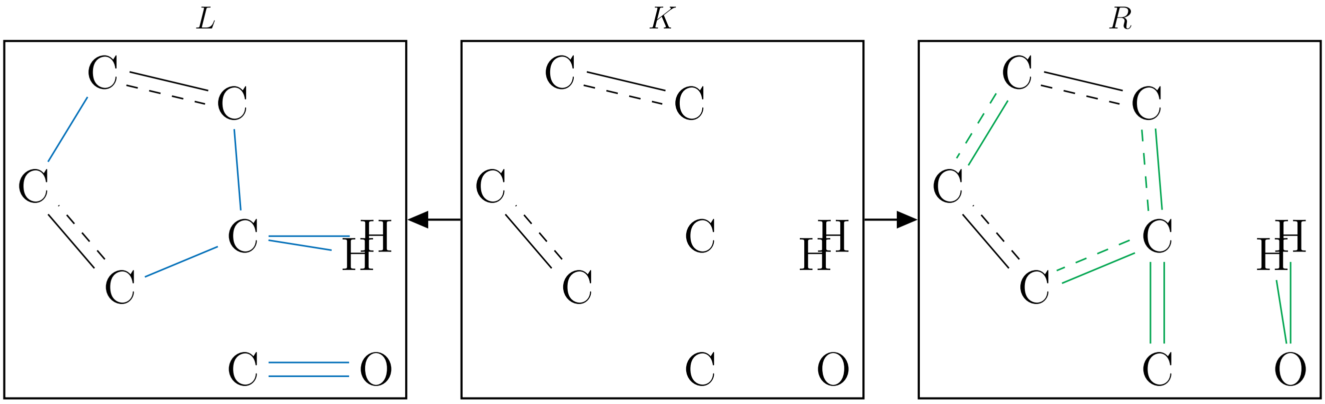

Files: out/1086_r_217_10300000_{L, K, R}

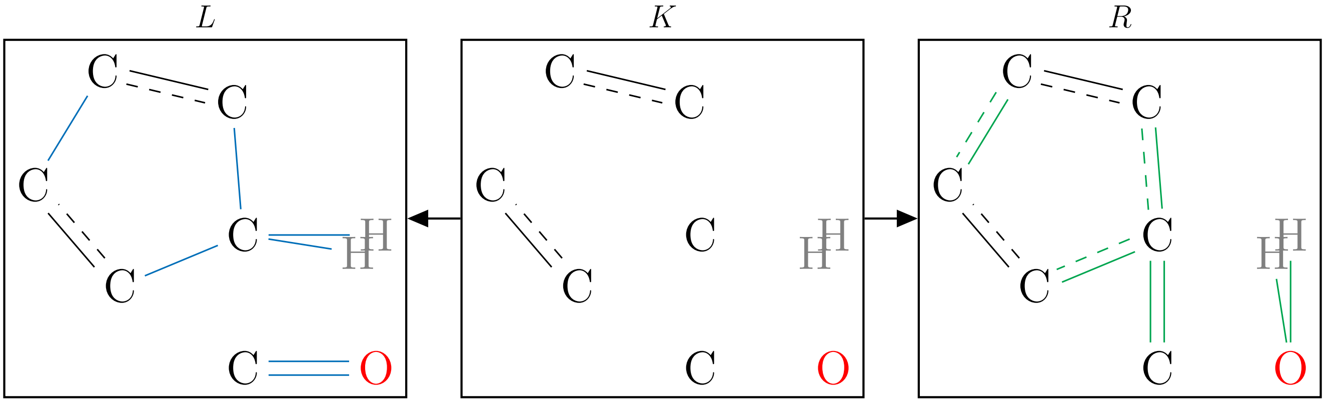

Files: out/1088_r_217_11300100_{L, K, R}

0.0.219 218

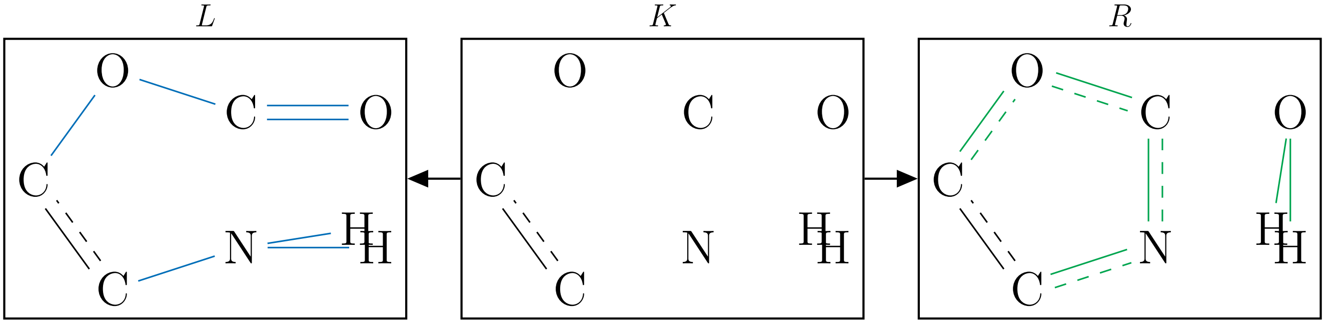

Files: out/1091_r_218_10300000_{L, K, R}

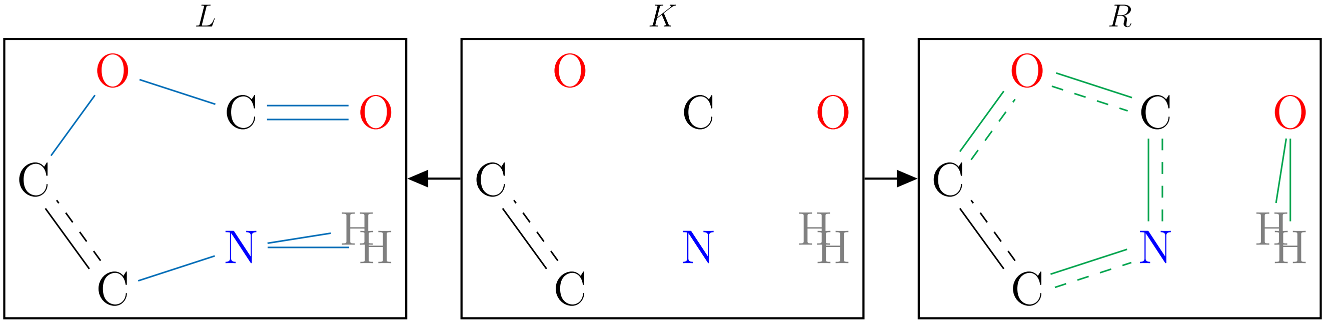

Files: out/1093_r_218_11300100_{L, K, R}

0.0.220 219

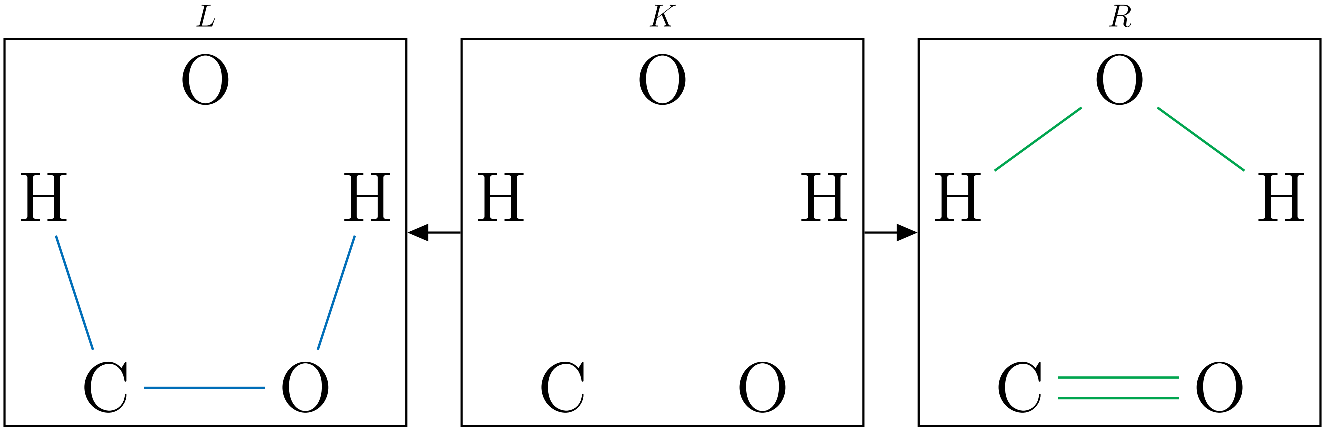

Files: out/1096_r_219_10300000_{L, K, R}

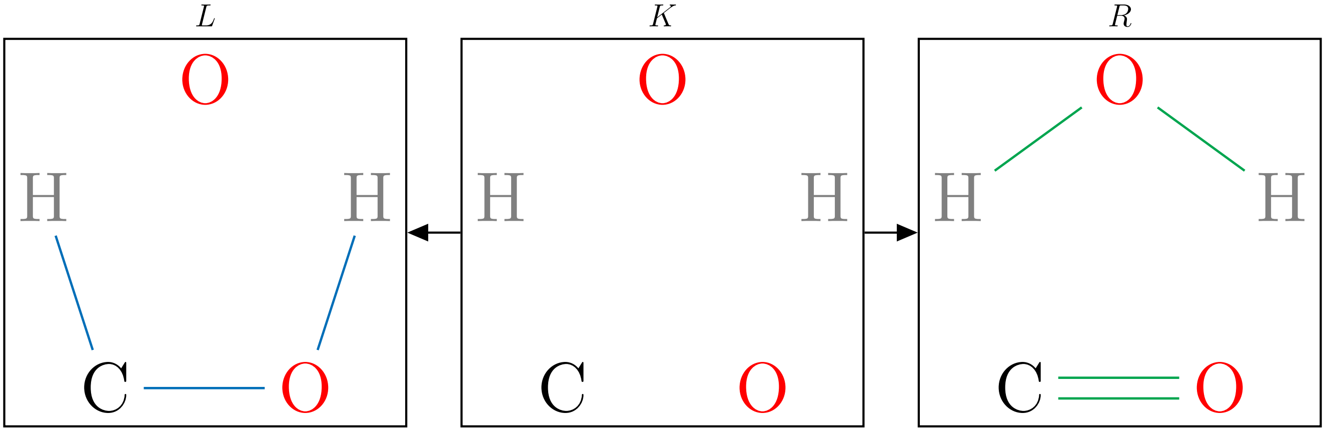

Files: out/1098_r_219_11300100_{L, K, R}

0.0.221 220

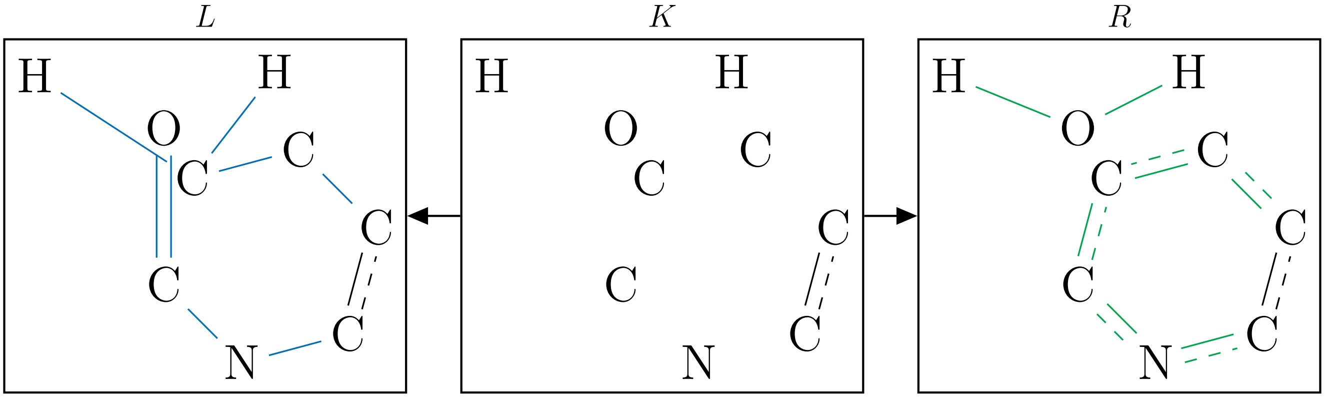

Files: out/1101_r_220_10300000_{L, K, R}

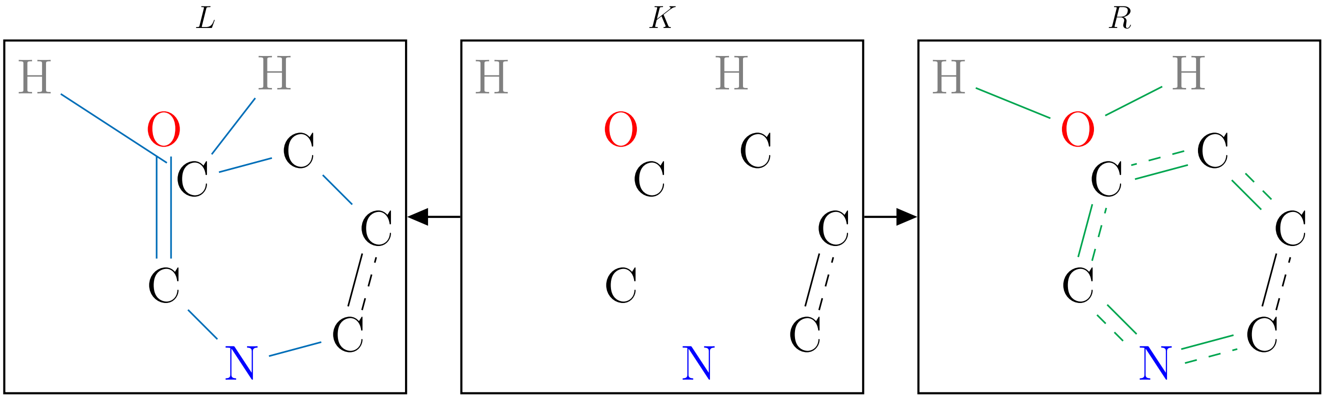

Files: out/1103_r_220_11300100_{L, K, R}

0.0.222 221

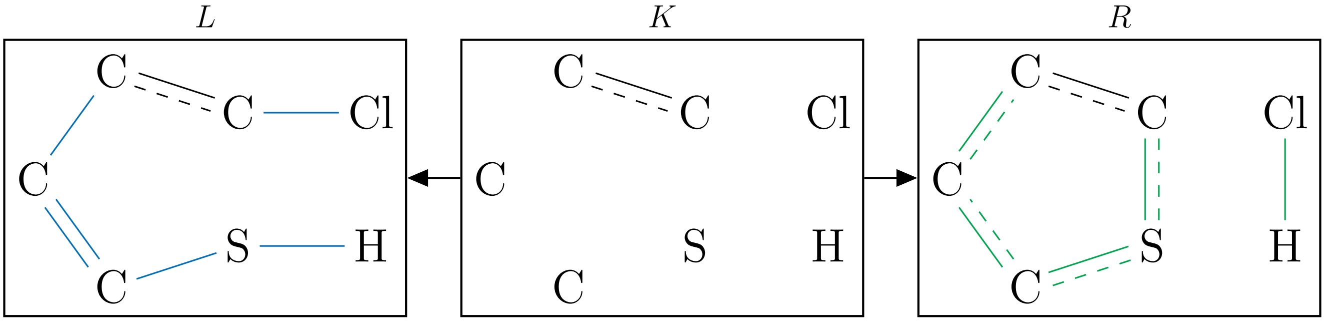

Files: out/1106_r_221_10300000_{L, K, R}

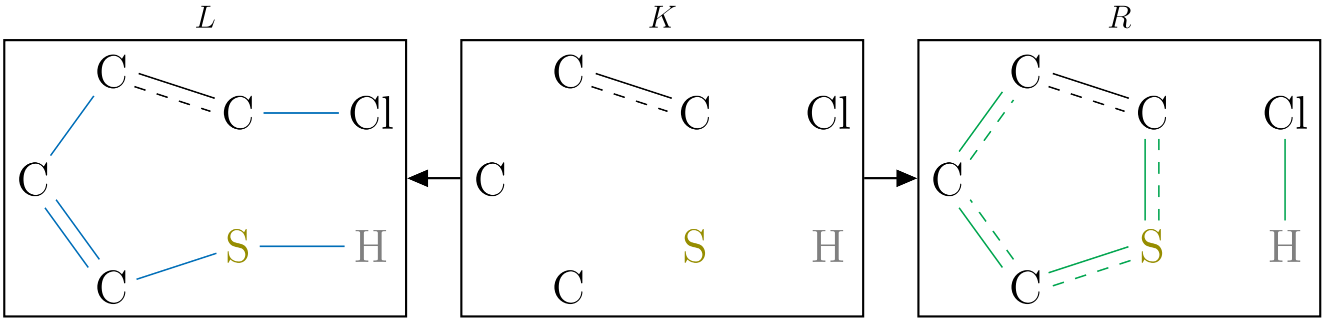

Files: out/1108_r_221_11300100_{L, K, R}

0.0.223 222

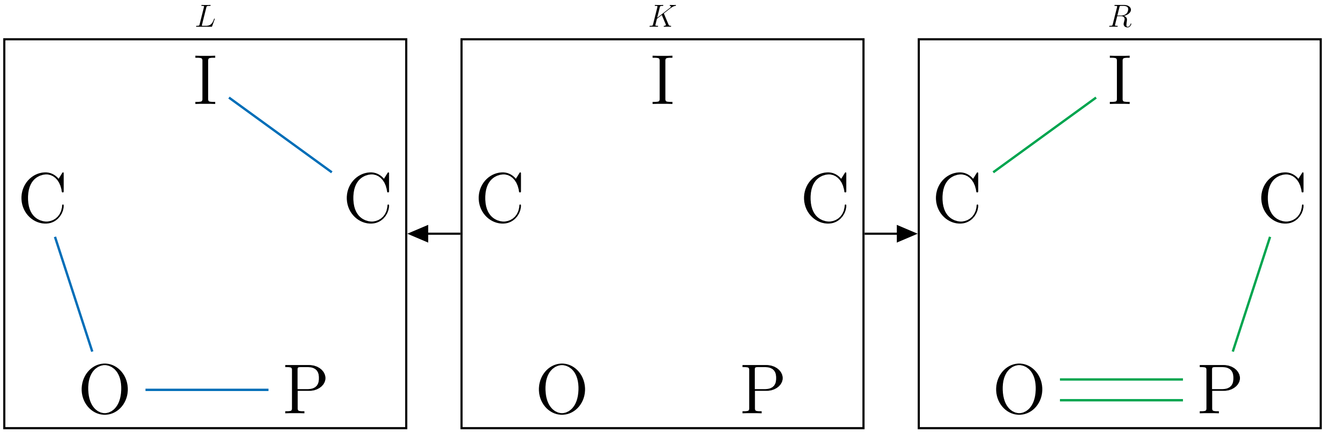

Files: out/1111_r_222_10300000_{L, K, R}

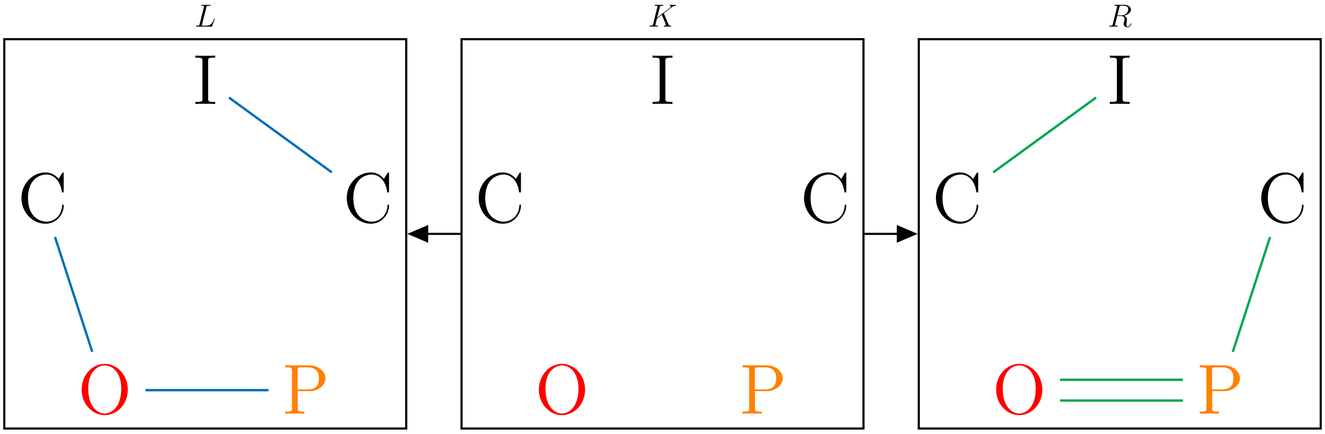

Files: out/1113_r_222_11300100_{L, K, R}

0.0.224 223

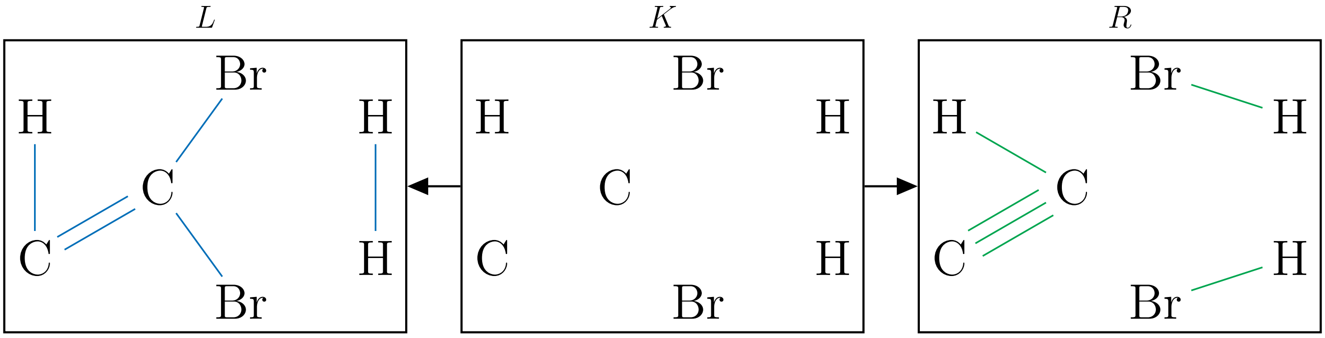

Files: out/1116_r_223_10300000_{L, K, R}

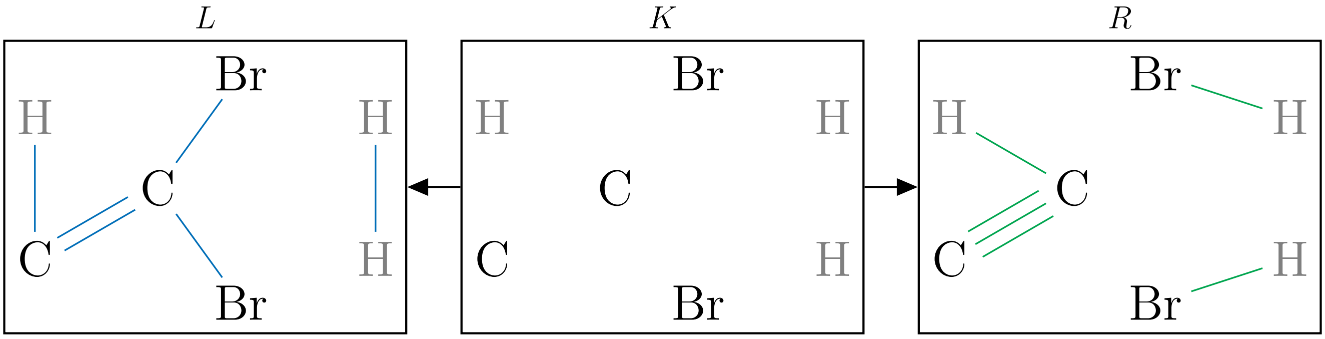

Files: out/1118_r_223_11300100_{L, K, R}

0.0.225 224

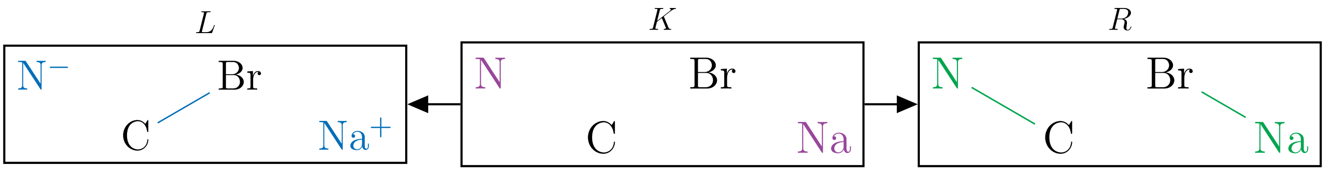

Files: out/1121_r_224_10300000_{L, K, R}

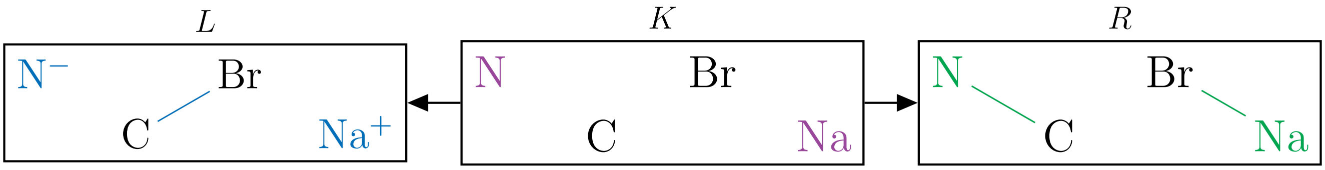

Files: out/1123_r_224_11300100_{L, K, R}

0.0.226 225

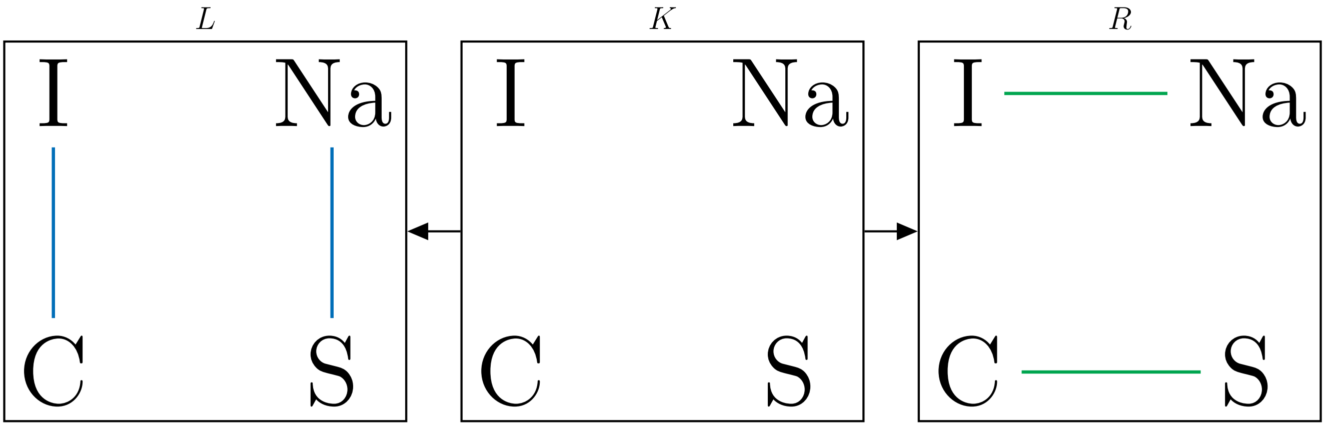

Files: out/1126_r_225_10300000_{L, K, R}

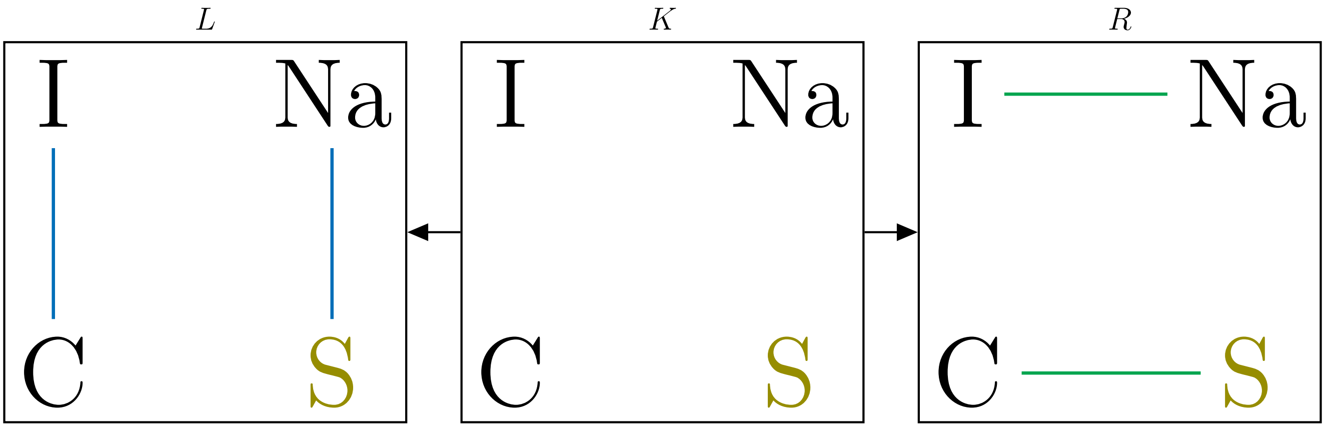

Files: out/1128_r_225_11300100_{L, K, R}

0.0.227 226

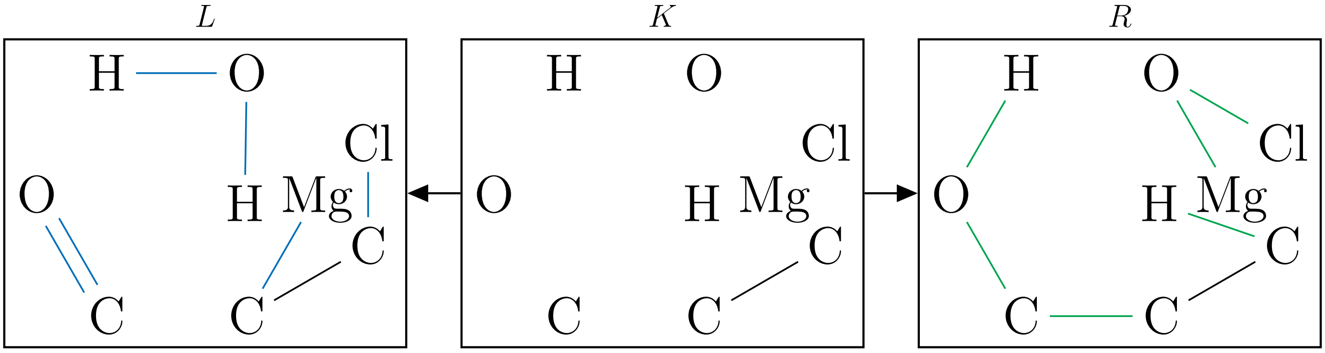

Files: out/1131_r_226_10300000_{L, K, R}

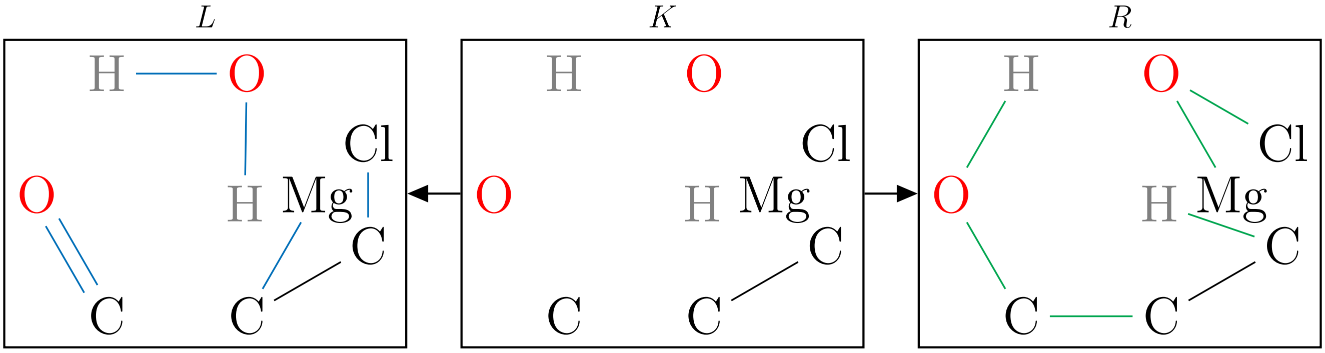

Files: out/1133_r_226_11300100_{L, K, R}

0.0.228 227

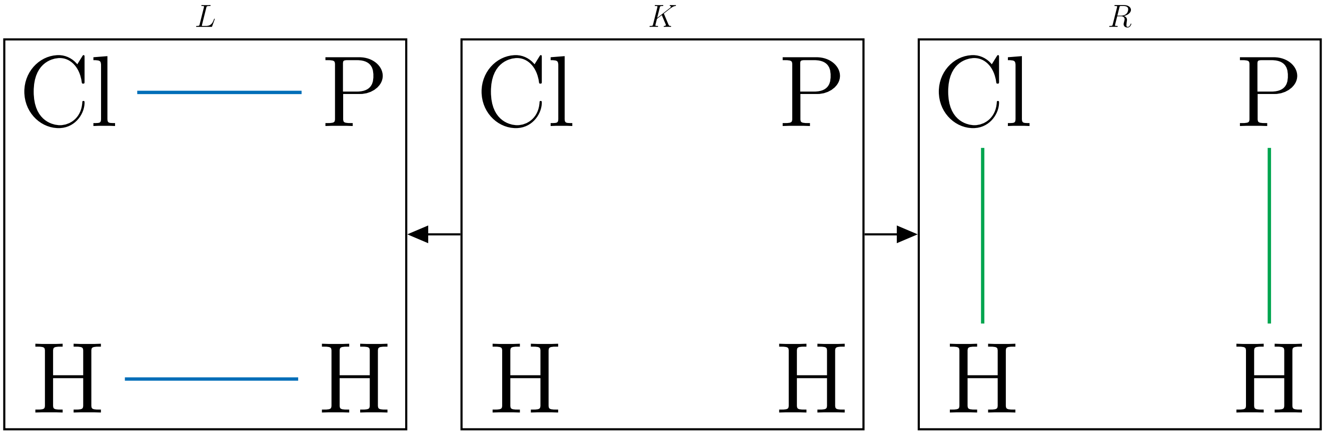

Files: out/1136_r_227_10300000_{L, K, R}

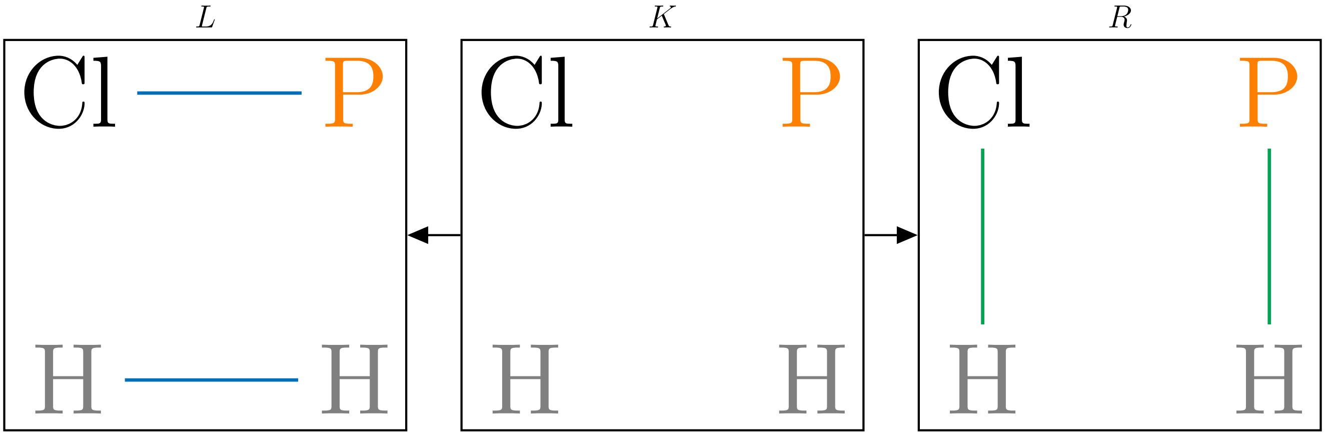

Files: out/1138_r_227_11300100_{L, K, R}

0.0.229 228

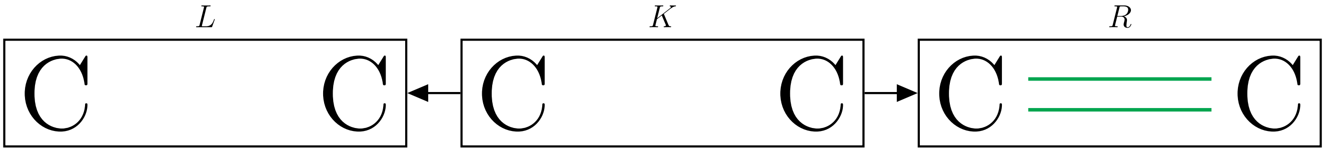

Files: out/1141_r_228_10300000_{L, K, R}

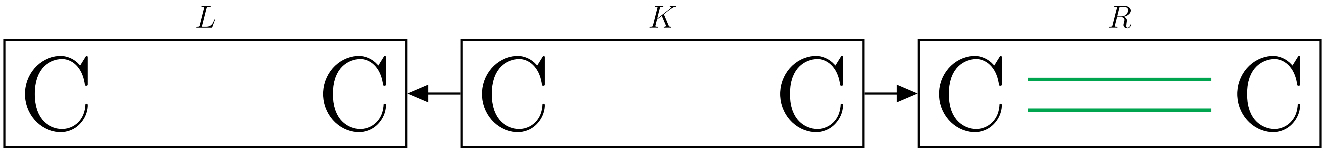

Files: out/1143_r_228_11300100_{L, K, R}

0.0.230 229

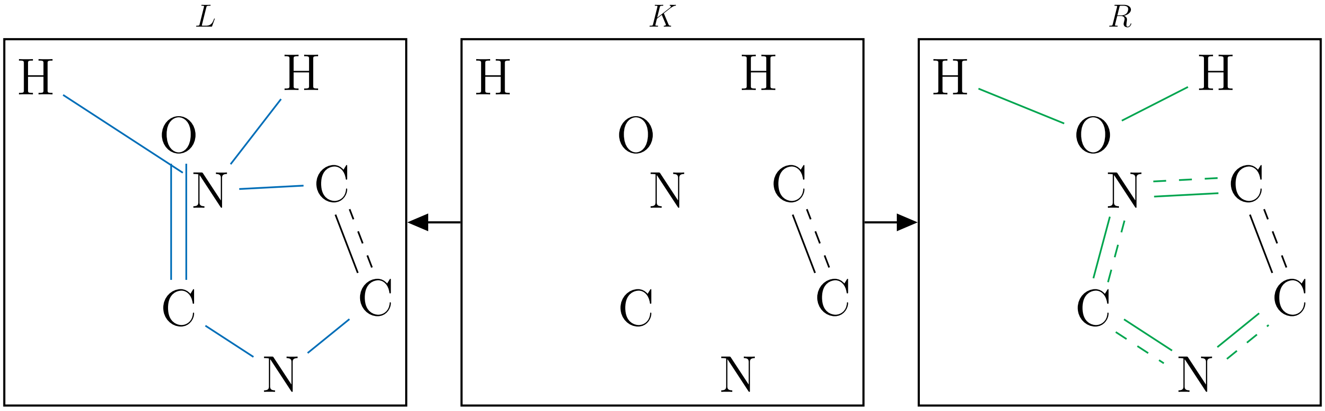

Files: out/1146_r_229_10300000_{L, K, R}

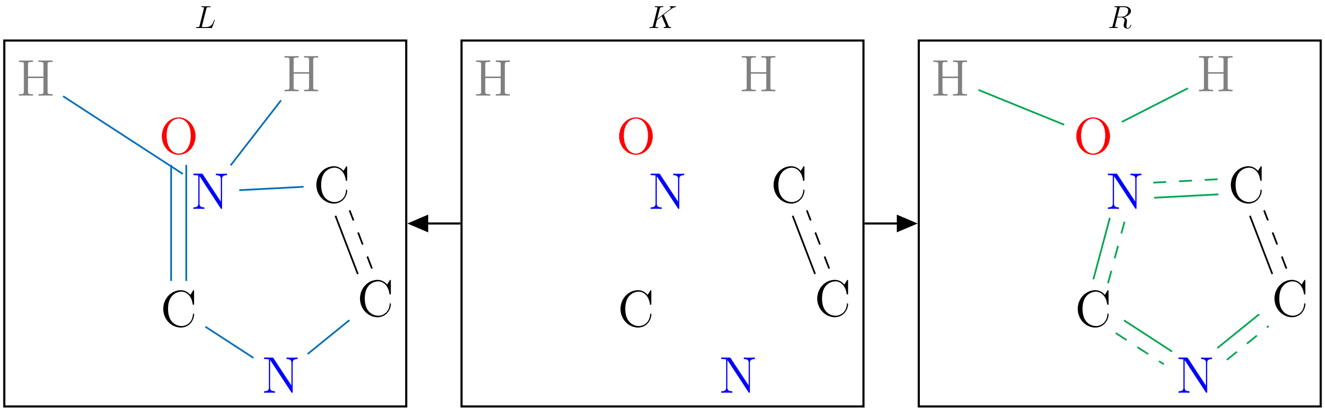

Files: out/1148_r_229_11300100_{L, K, R}

0.0.231 230

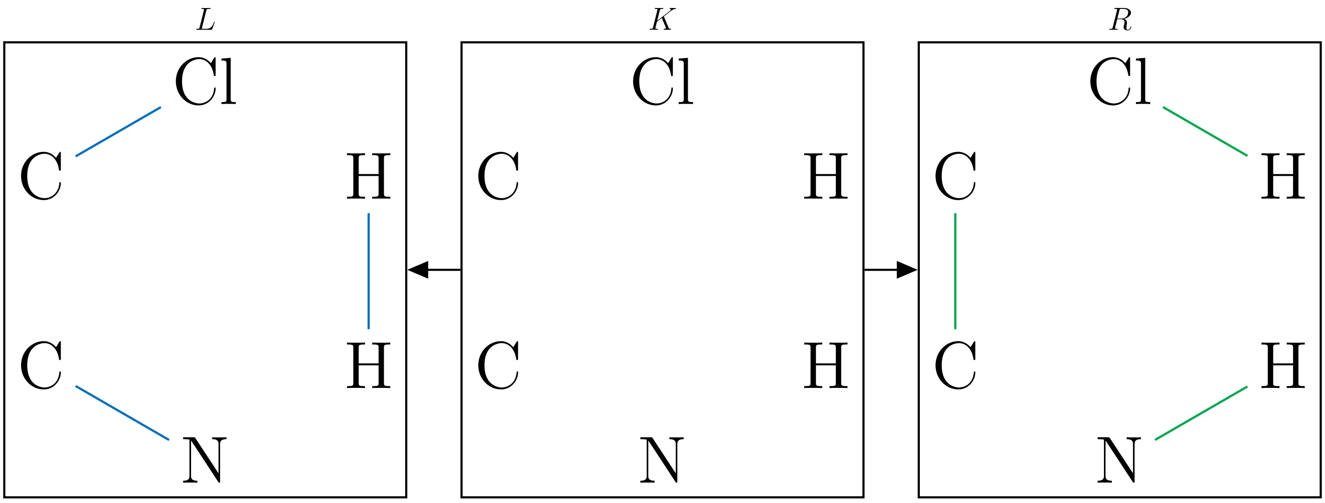

Files: out/1151_r_230_10300000_{L, K, R}

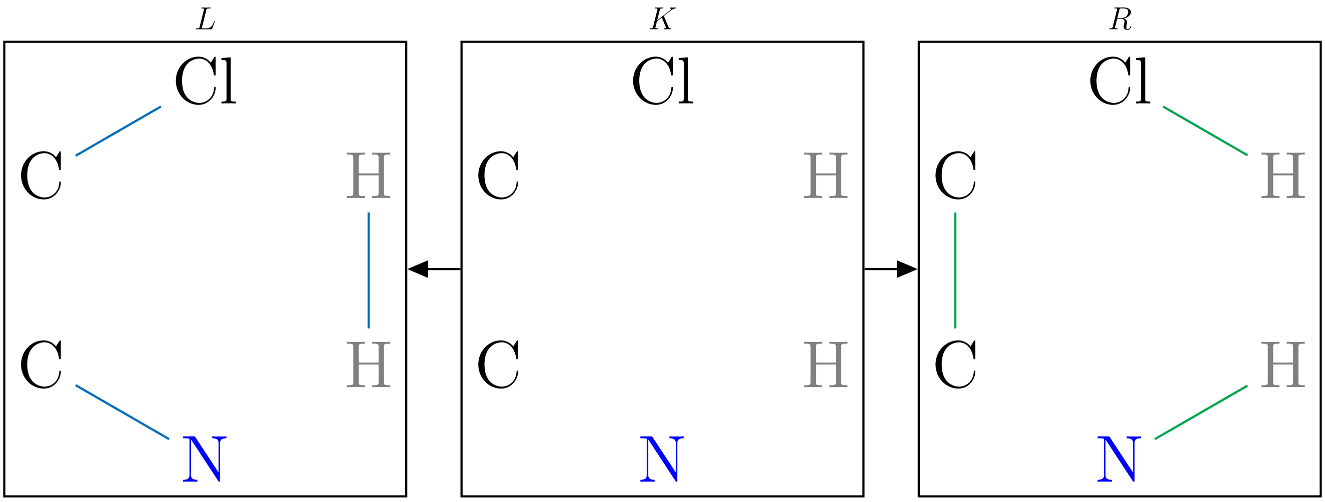

Files: out/1153_r_230_11300100_{L, K, R}

0.0.232 231

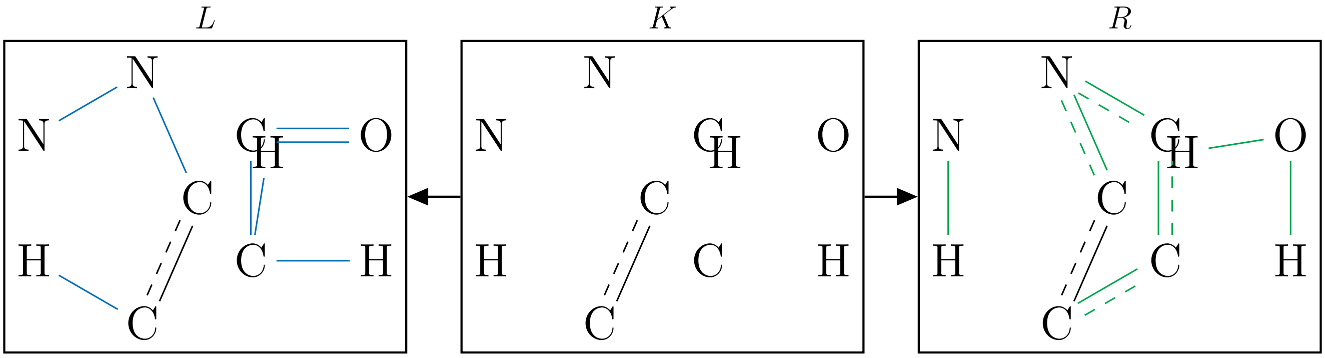

Files: out/1156_r_231_10300000_{L, K, R}

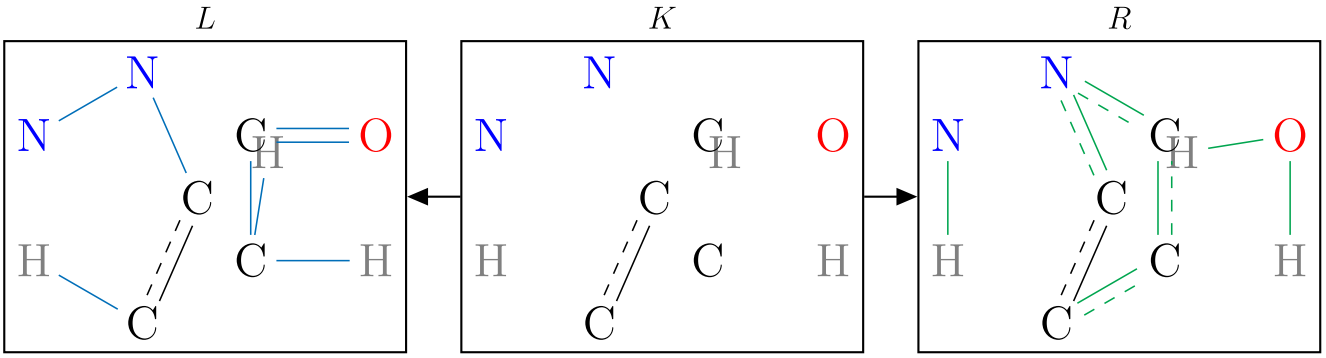

Files: out/1158_r_231_11300100_{L, K, R}

0.0.233 232

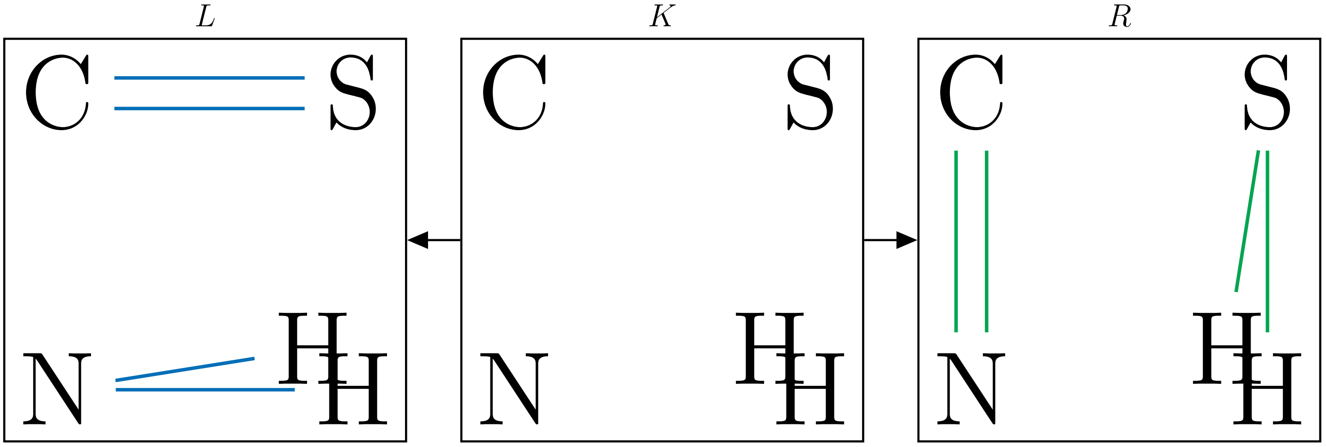

Files: out/1161_r_232_10300000_{L, K, R}

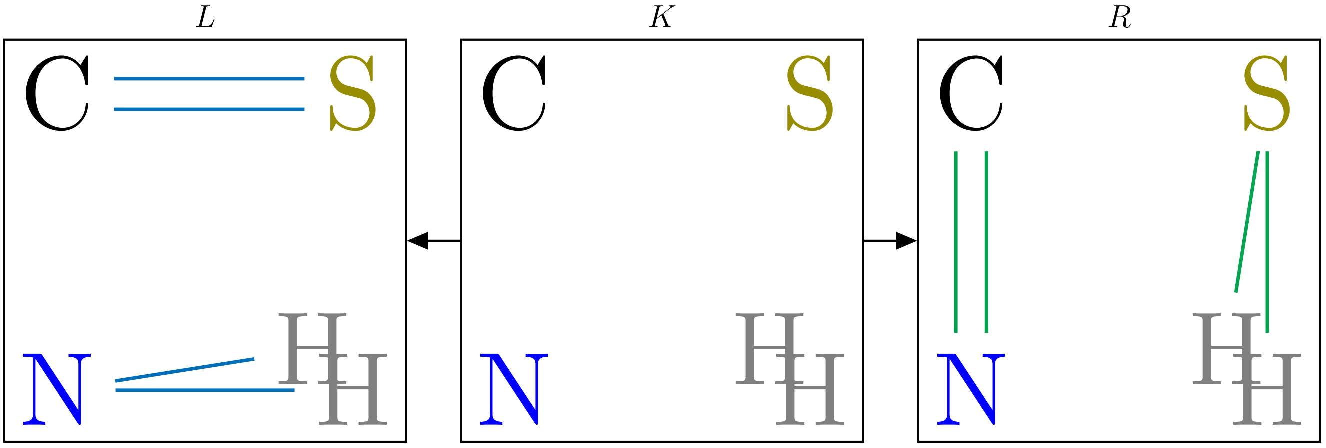

Files: out/1163_r_232_11300100_{L, K, R}

0.0.234 233

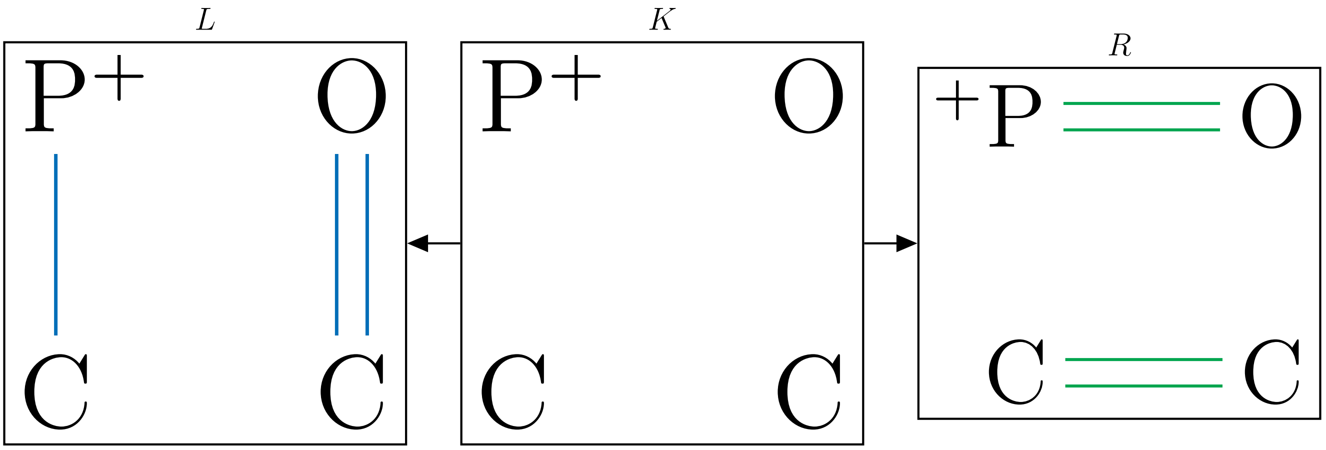

Files: out/1166_r_233_10300000_{L, K, R}

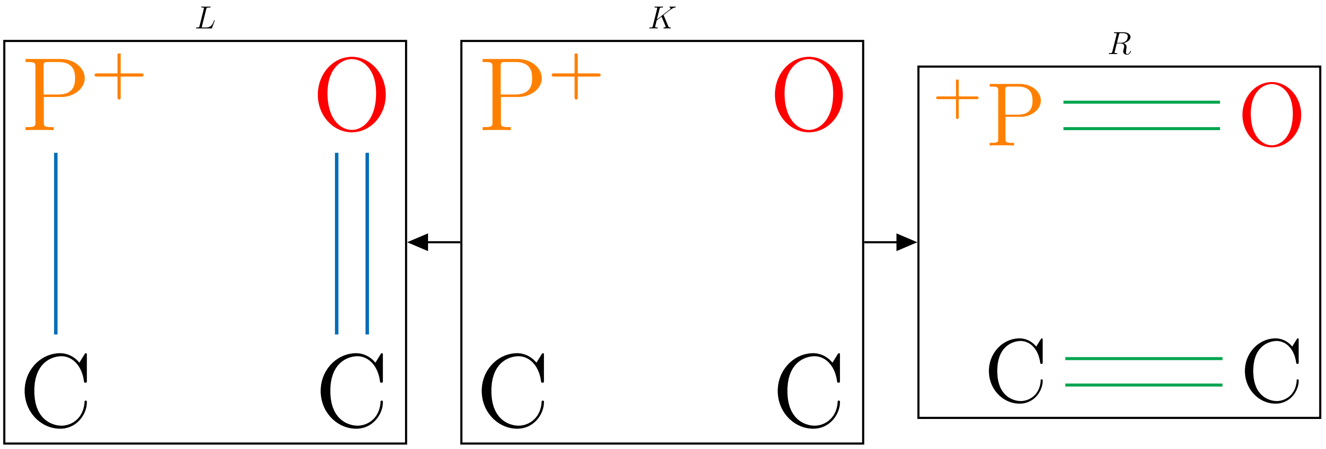

Files: out/1168_r_233_11300100_{L, K, R}

0.0.235 234

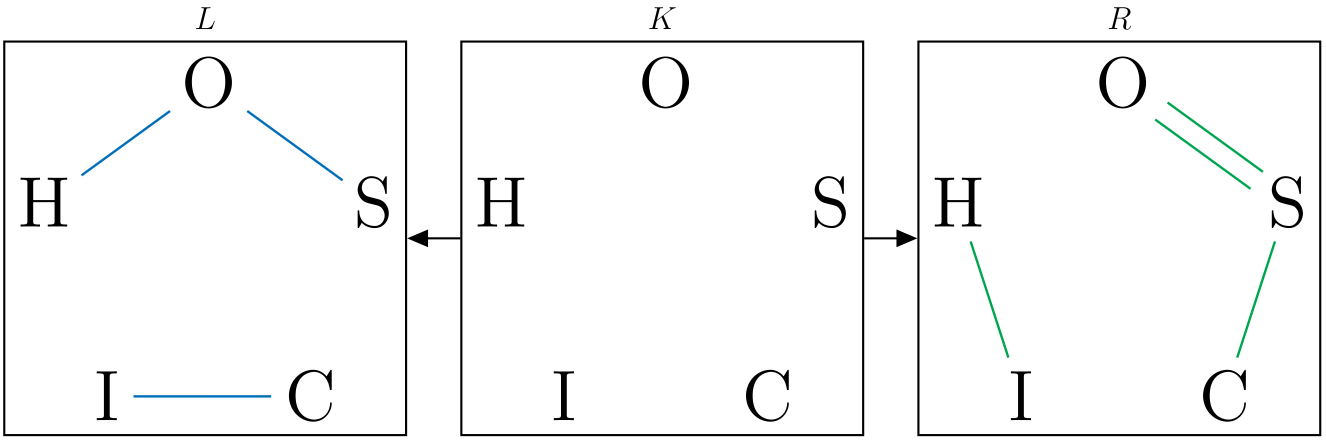

Files: out/1171_r_234_10300000_{L, K, R}

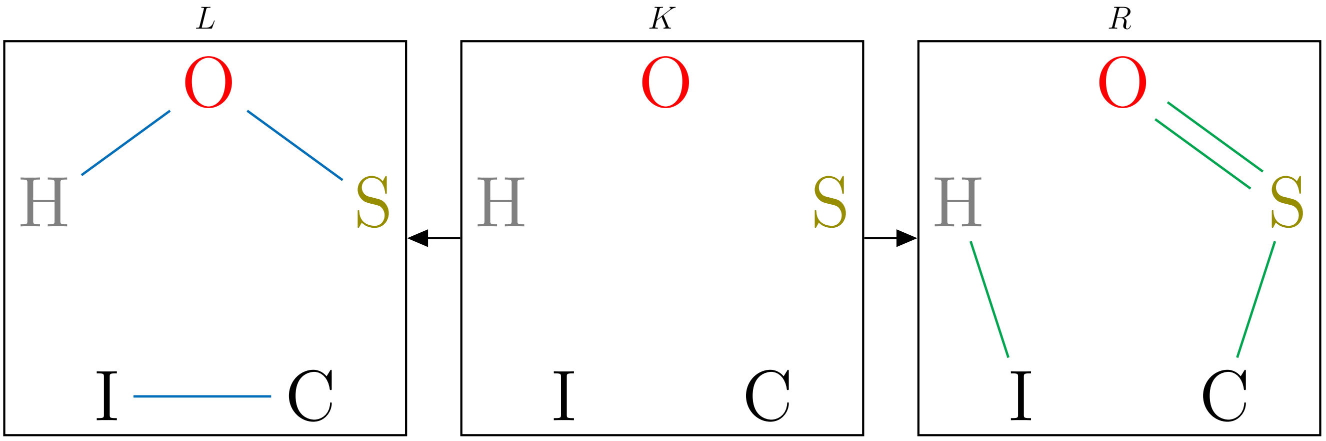

Files: out/1173_r_234_11300100_{L, K, R}

0.0.236 235

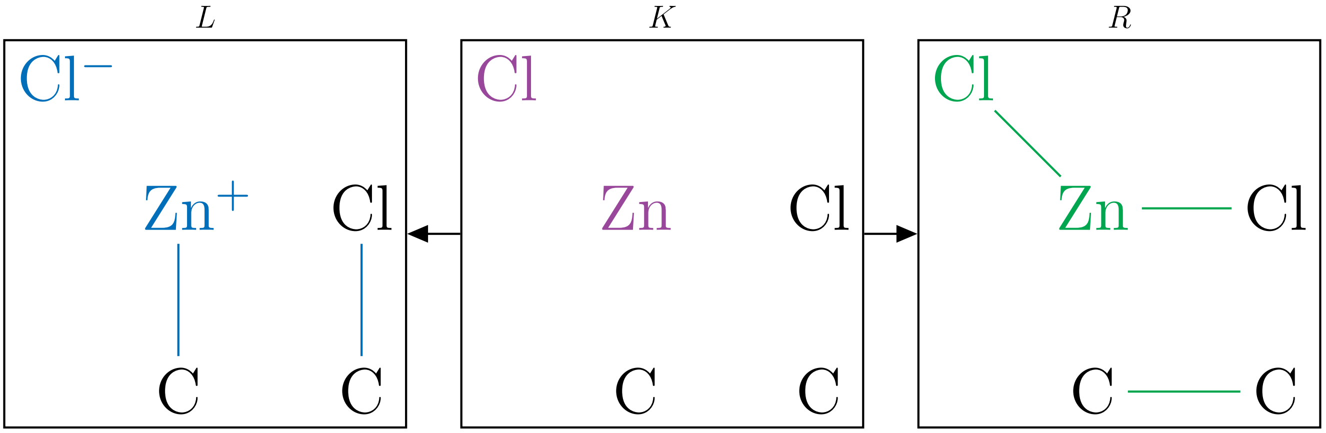

Files: out/1176_r_235_10300000_{L, K, R}

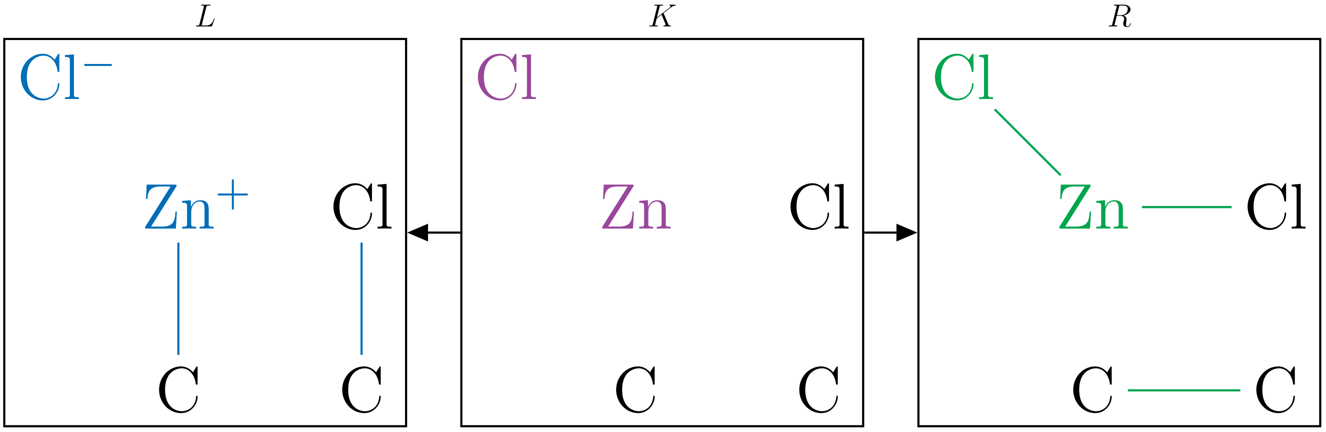

Files: out/1178_r_235_11300100_{L, K, R}

0.0.237 236

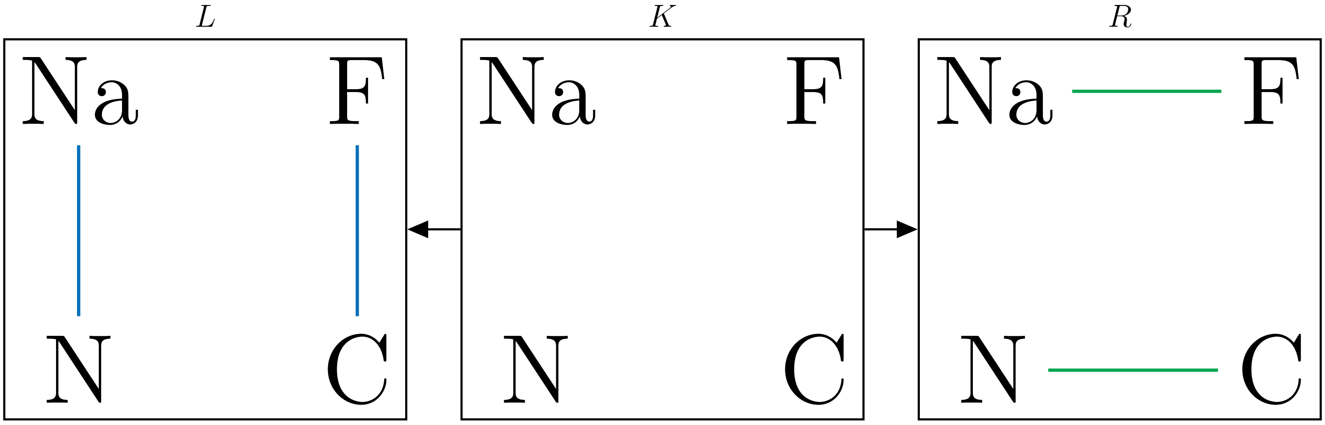

Files: out/1181_r_236_10300000_{L, K, R}

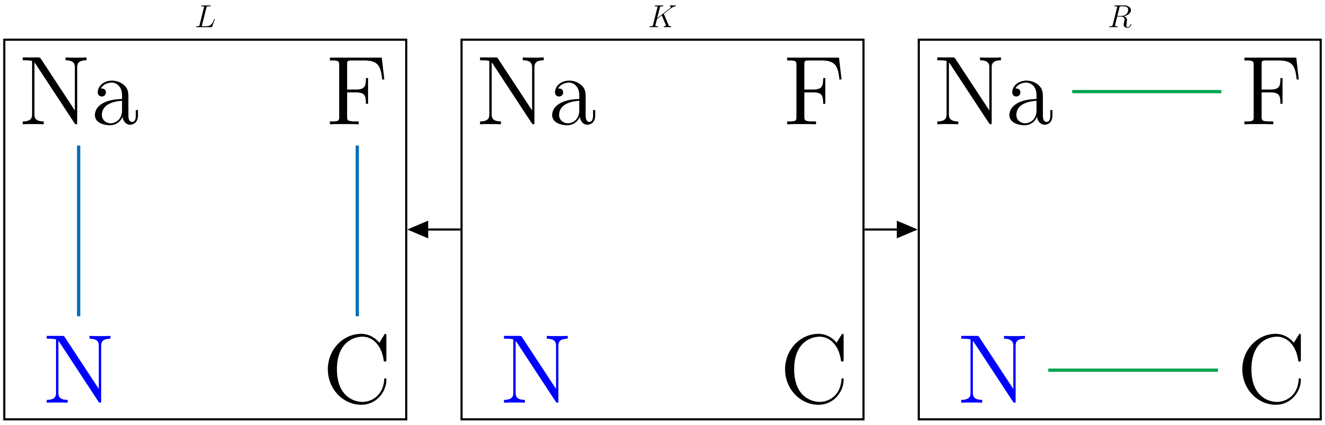

Files: out/1183_r_236_11300100_{L, K, R}

0.0.238 237

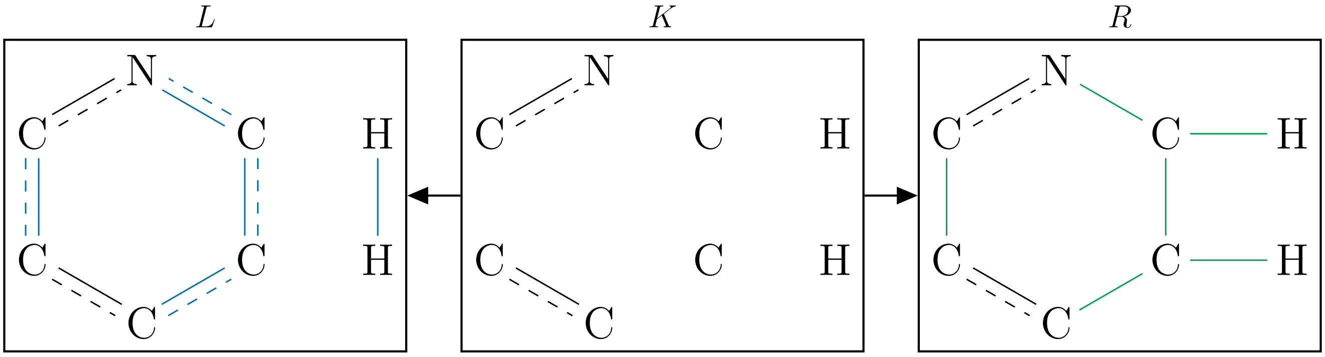

Files: out/1186_r_237_10300000_{L, K, R}

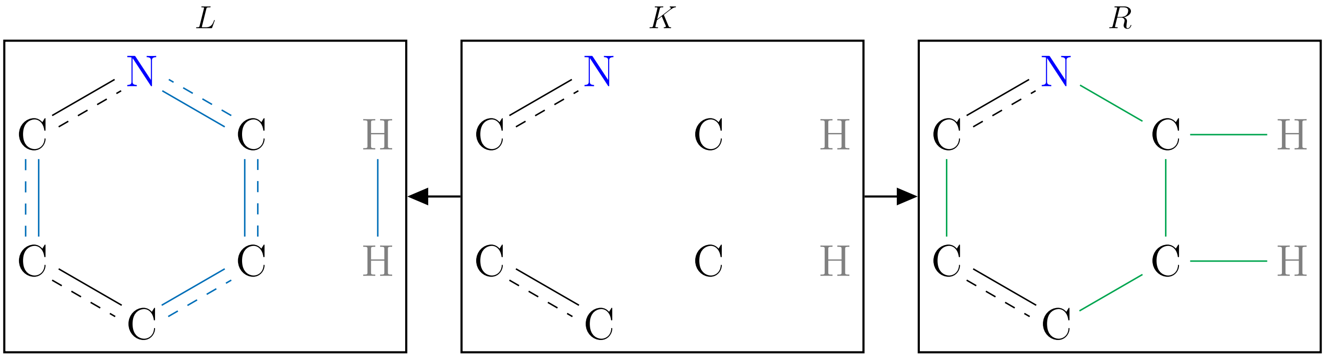

Files: out/1188_r_237_11300100_{L, K, R}

0.0.239 238

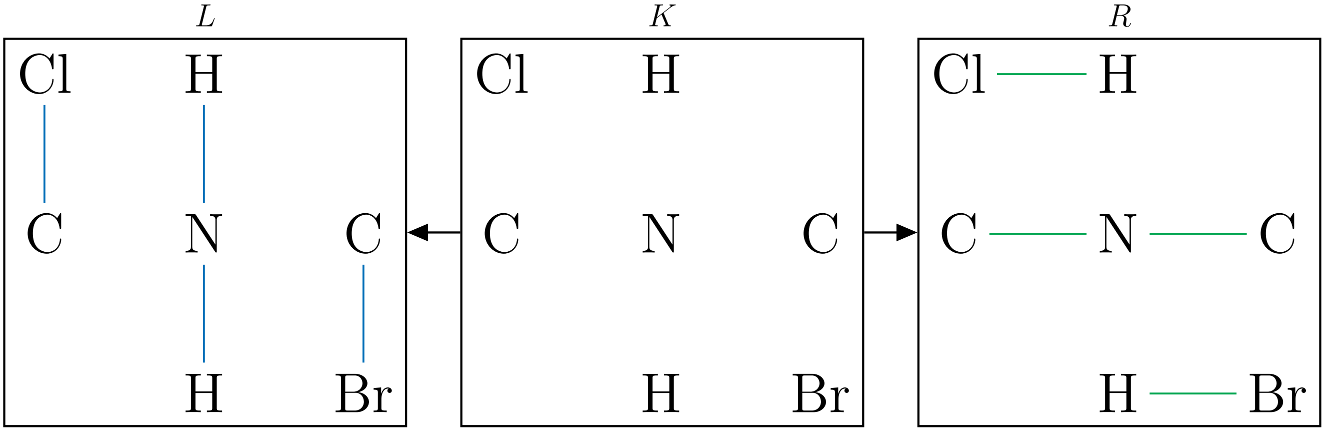

Files: out/1191_r_238_10300000_{L, K, R}

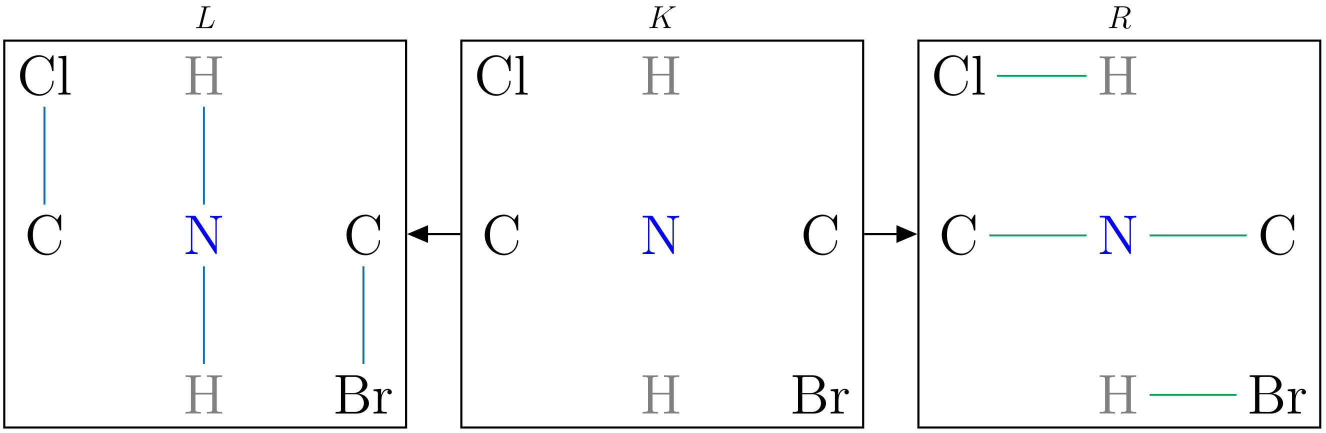

Files: out/1193_r_238_11300100_{L, K, R}

0.0.240 239

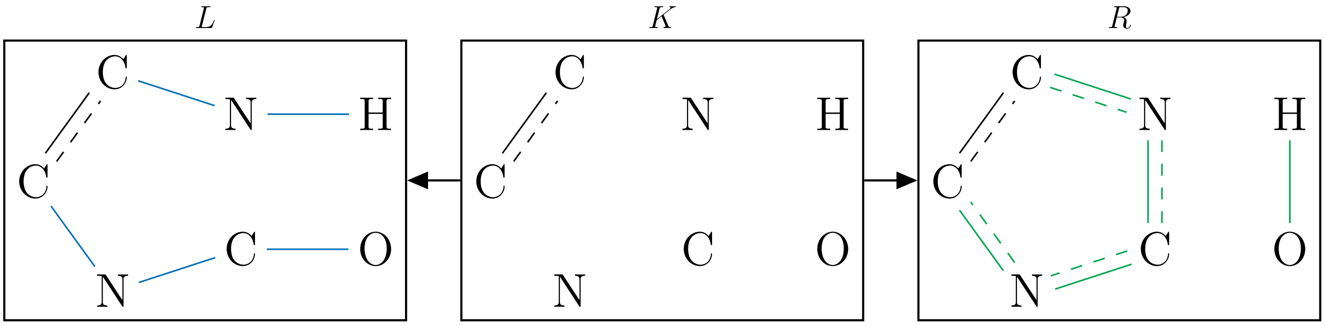

Files: out/1196_r_239_10300000_{L, K, R}

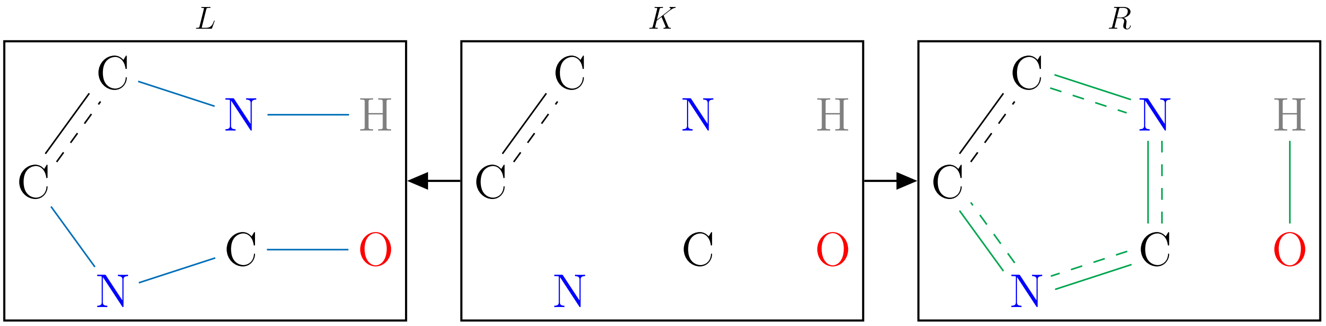

Files: out/1198_r_239_11300100_{L, K, R}

0.0.241 240

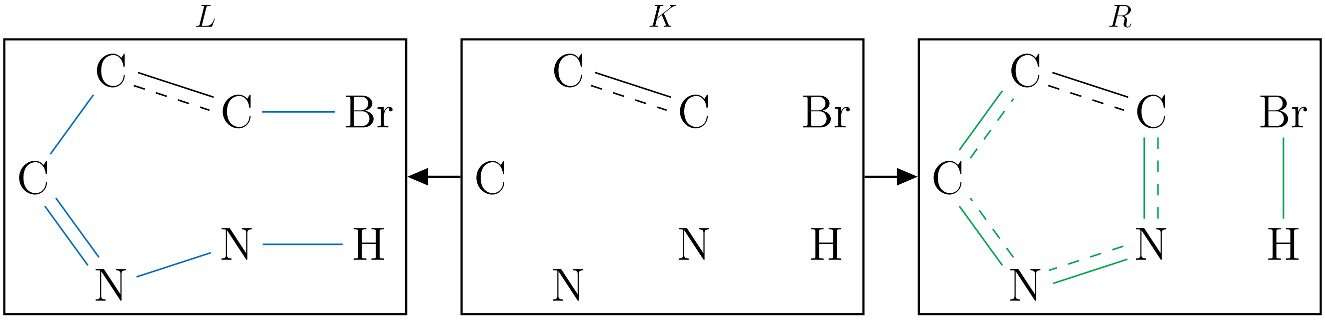

Files: out/1201_r_240_10300000_{L, K, R}

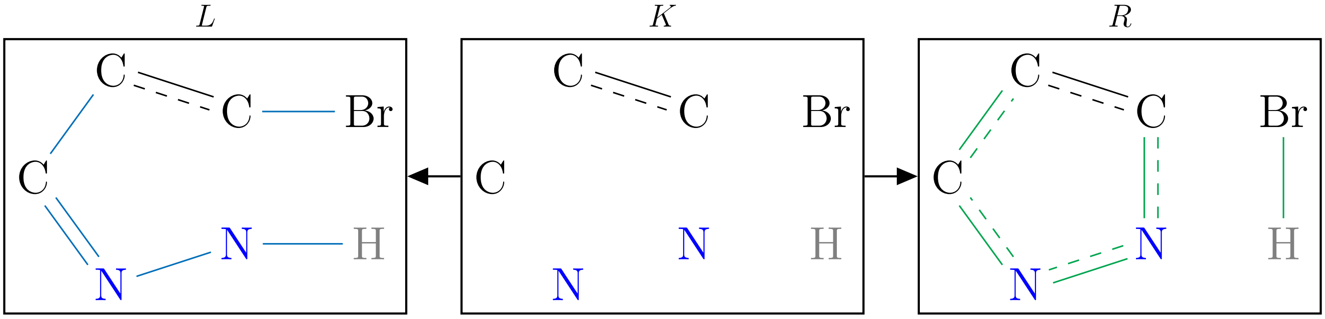

Files: out/1203_r_240_11300100_{L, K, R}

0.0.242 241

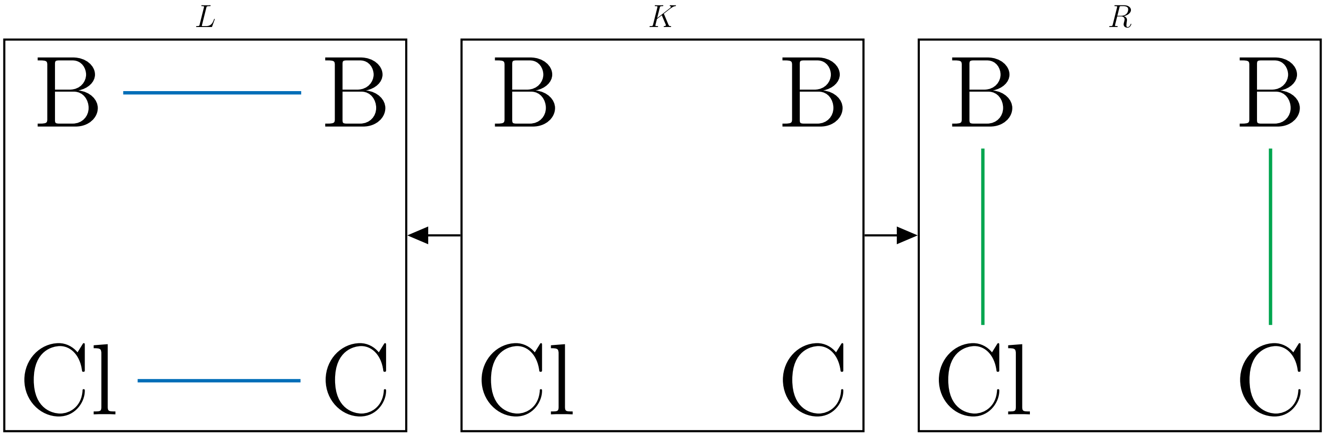

Files: out/1206_r_241_10300000_{L, K, R}

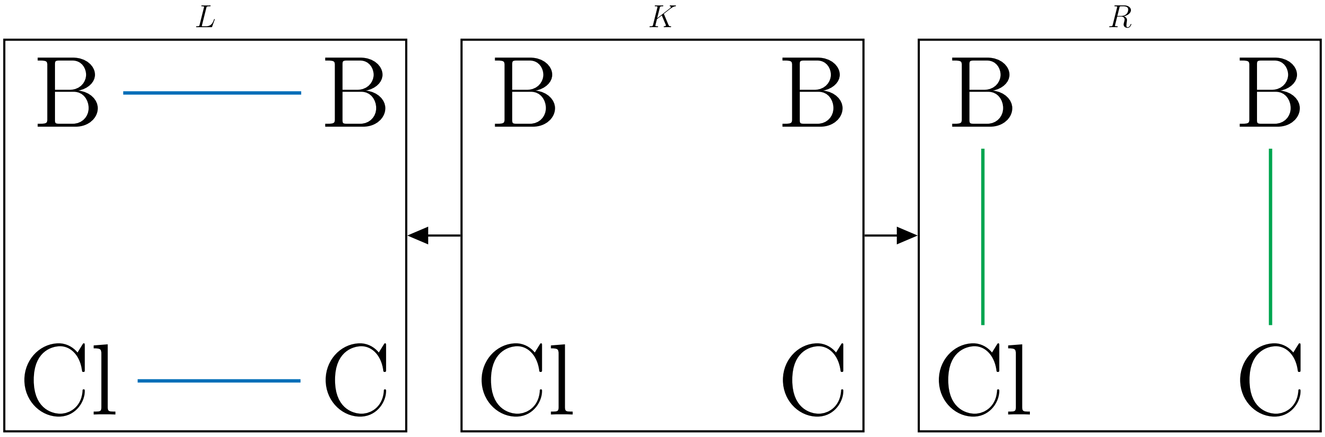

Files: out/1208_r_241_11300100_{L, K, R}

0.0.243 242

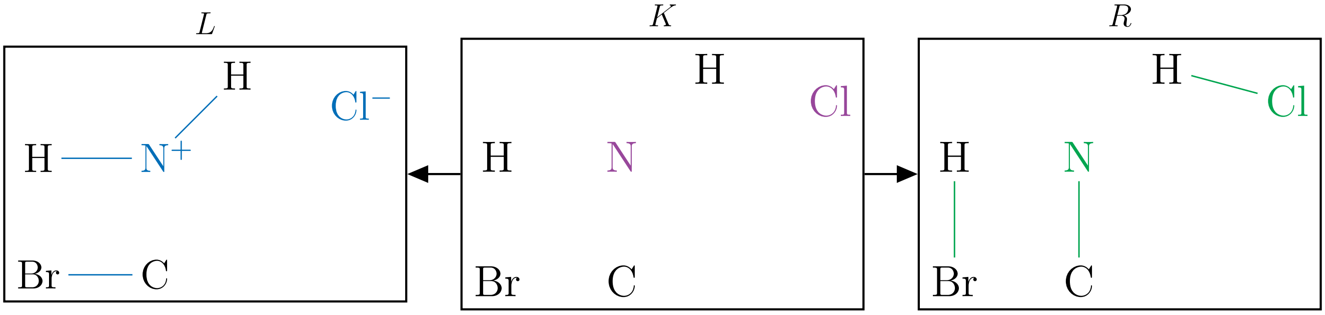

Files: out/1211_r_242_10300000_{L, K, R}

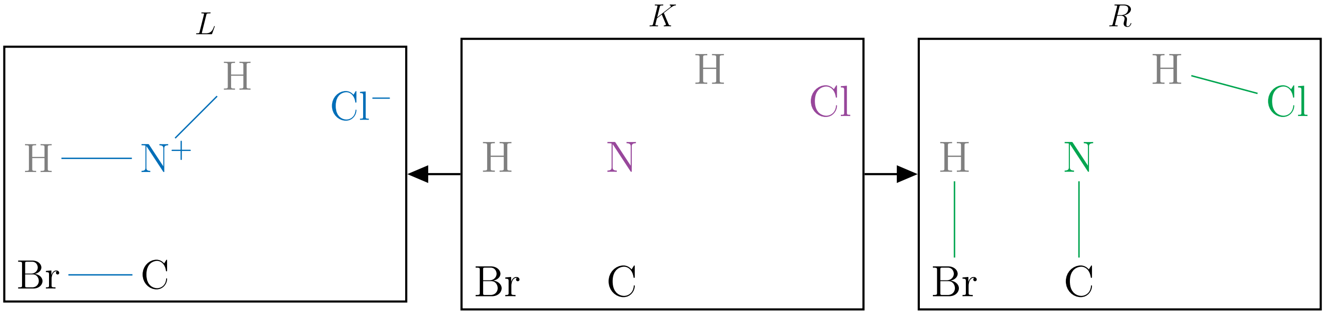

Files: out/1213_r_242_11300100_{L, K, R}

0.0.244 243

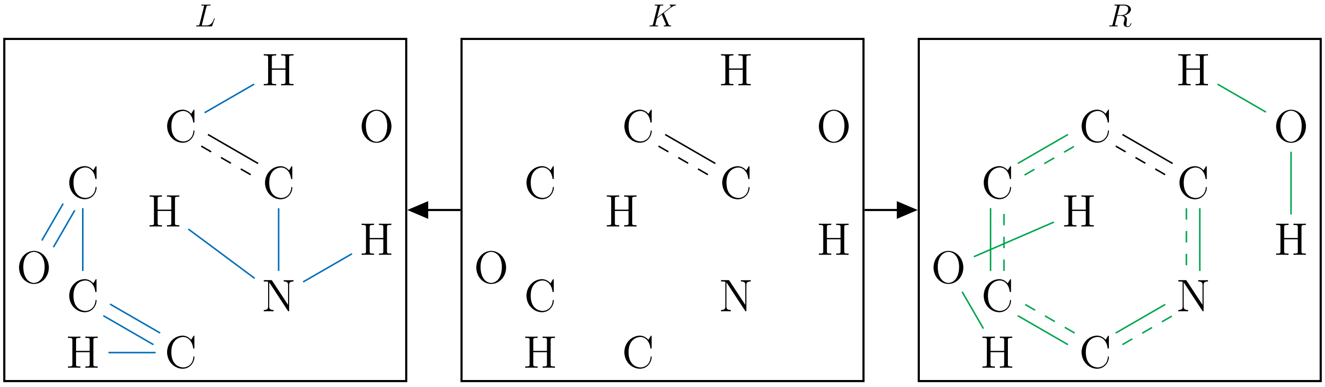

Files: out/1216_r_243_10300000_{L, K, R}

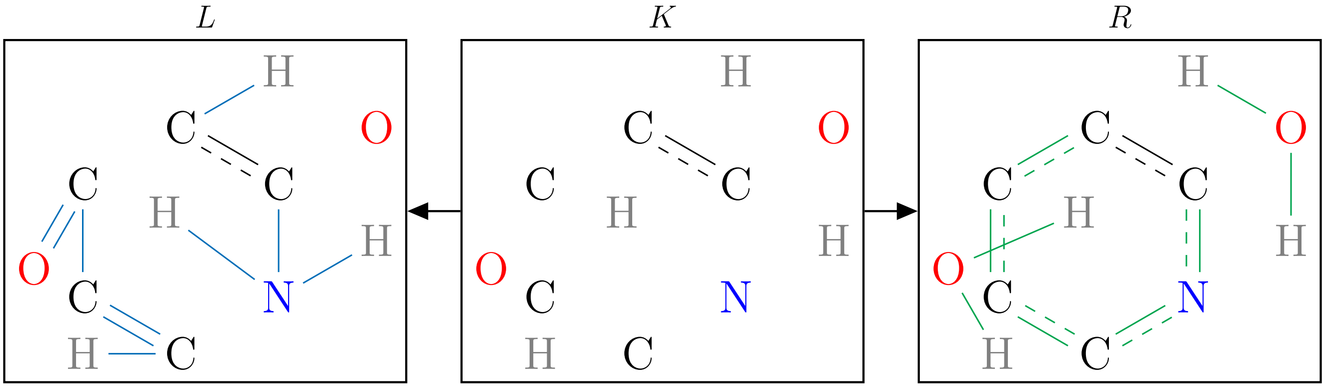

Files: out/1218_r_243_11300100_{L, K, R}

0.0.245 244

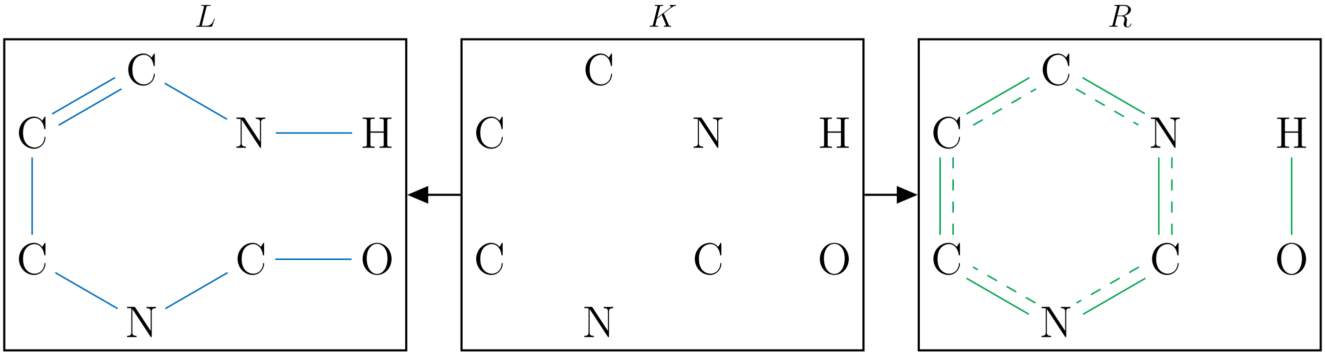

Files: out/1221_r_244_10300000_{L, K, R}

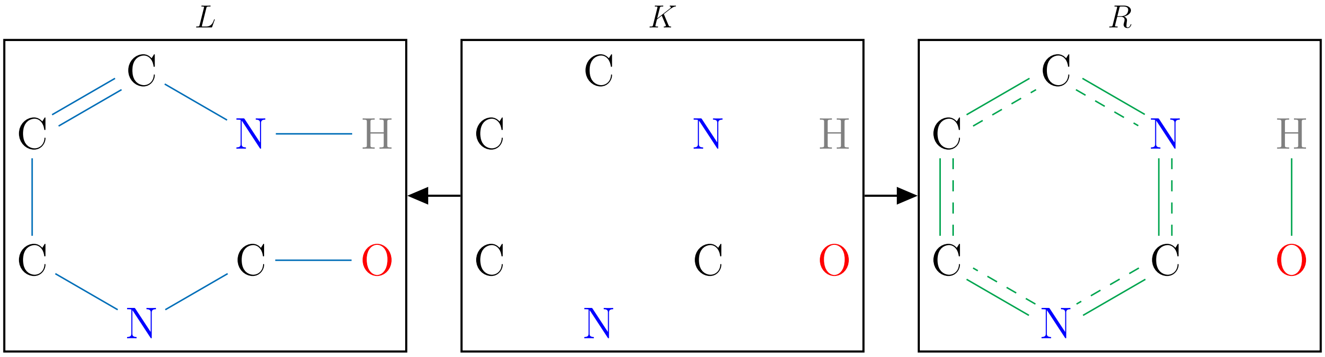

Files: out/1223_r_244_11300100_{L, K, R}

0.0.246 245

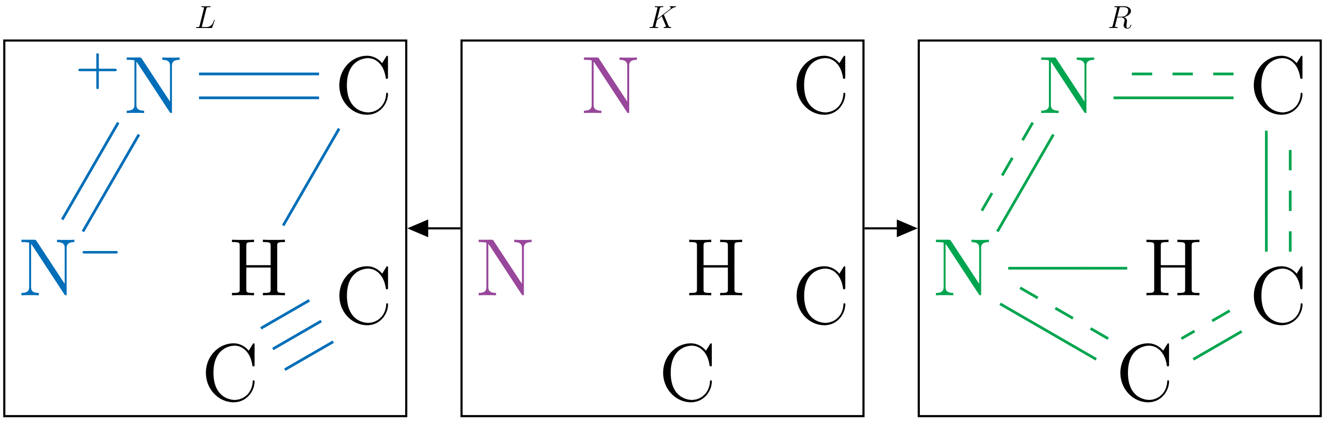

Files: out/1226_r_245_10300000_{L, K, R}

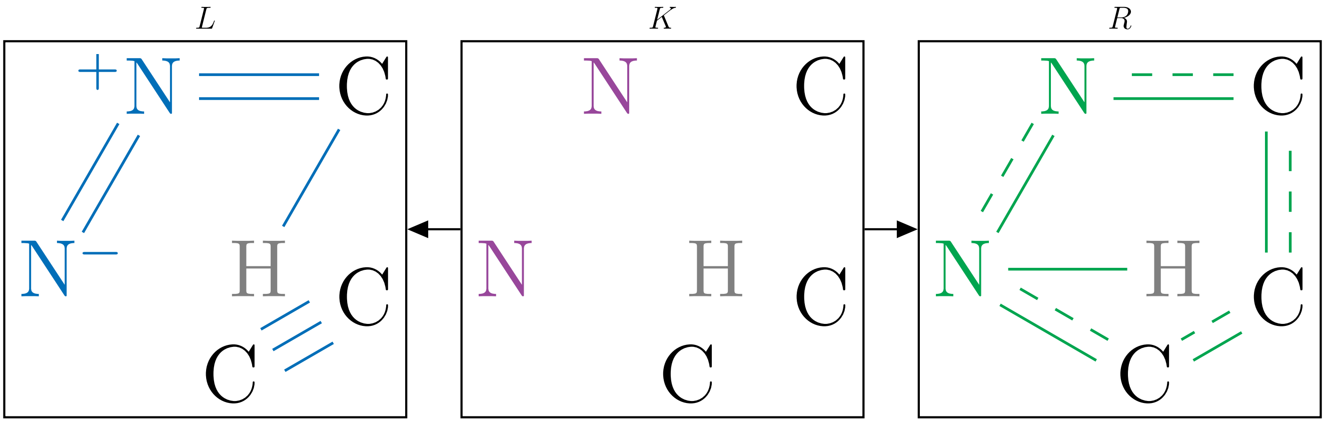

Files: out/1228_r_245_11300100_{L, K, R}

0.0.247 246

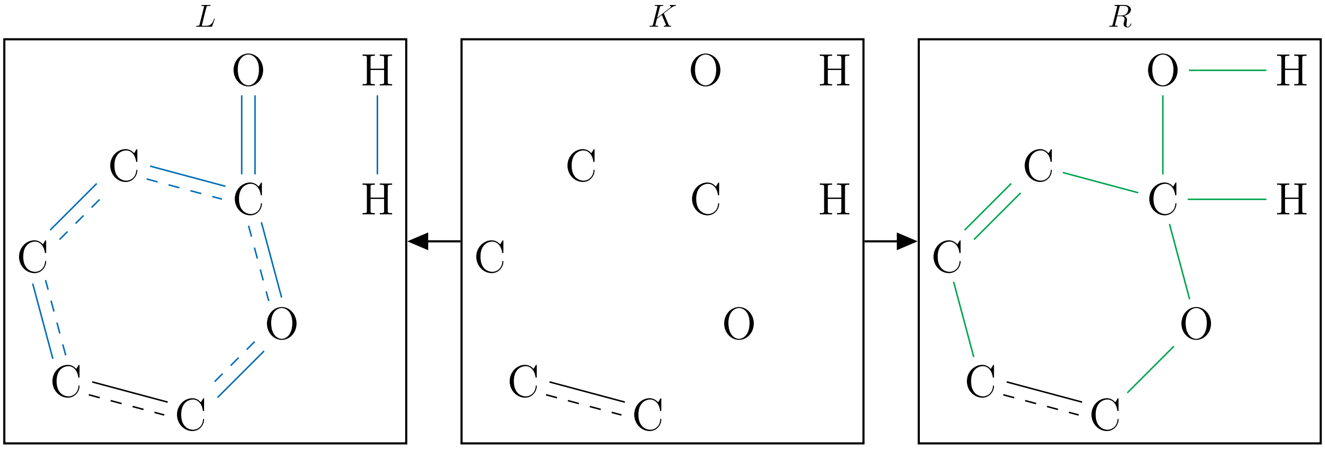

Files: out/1231_r_246_10300000_{L, K, R}

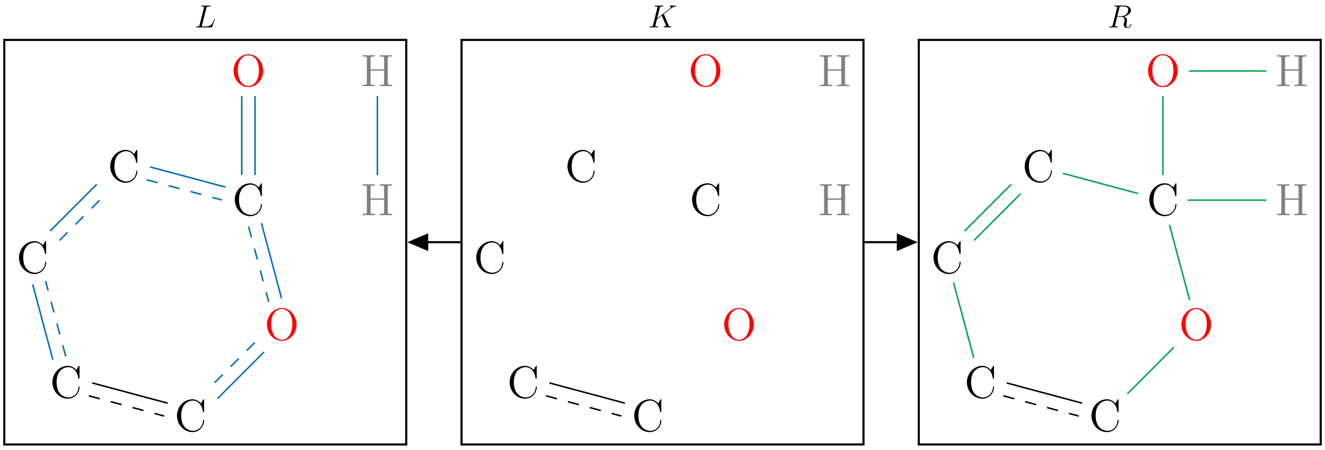

Files: out/1233_r_246_11300100_{L, K, R}

0.0.248 247

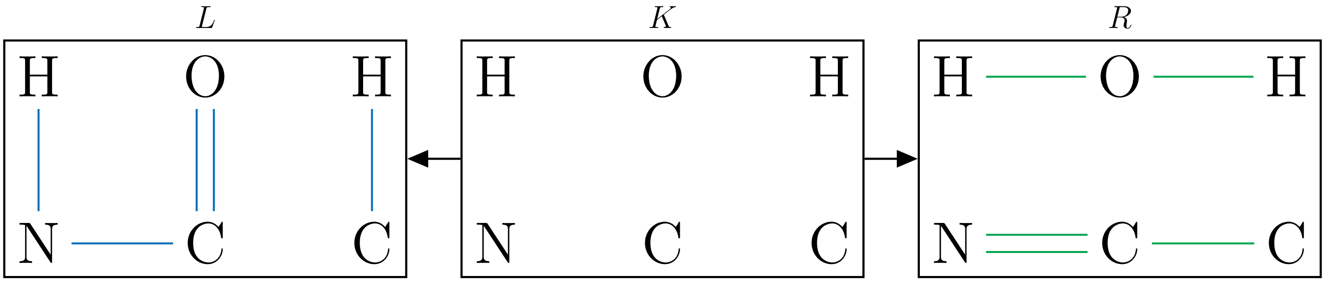

Files: out/1236_r_247_10300000_{L, K, R}

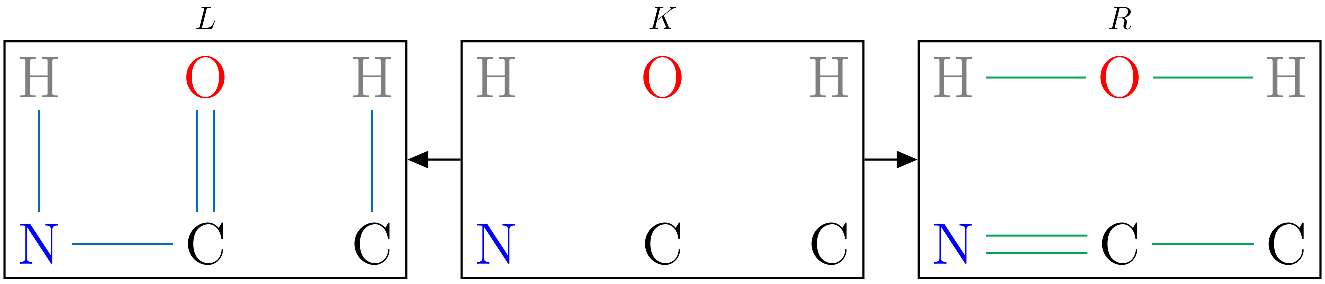

Files: out/1238_r_247_11300100_{L, K, R}

0.0.249 248

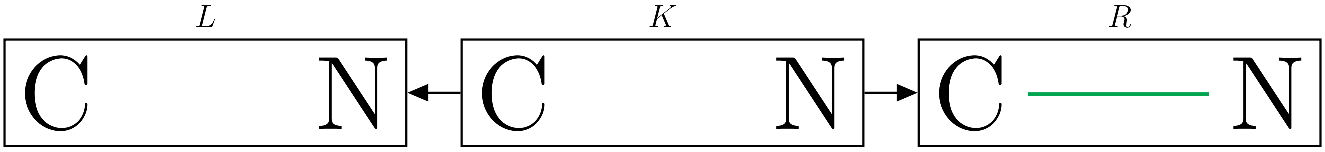

Files: out/1241_r_248_10300000_{L, K, R}

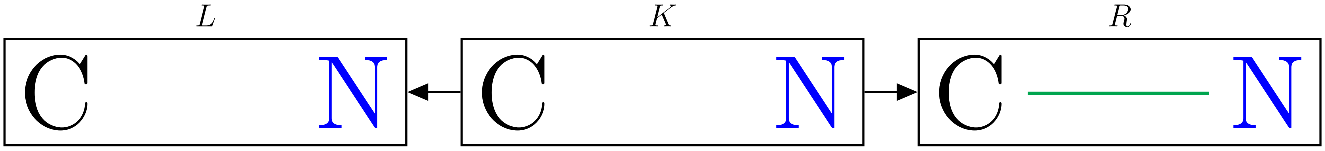

Files: out/1243_r_248_11300100_{L, K, R}

0.0.250 249

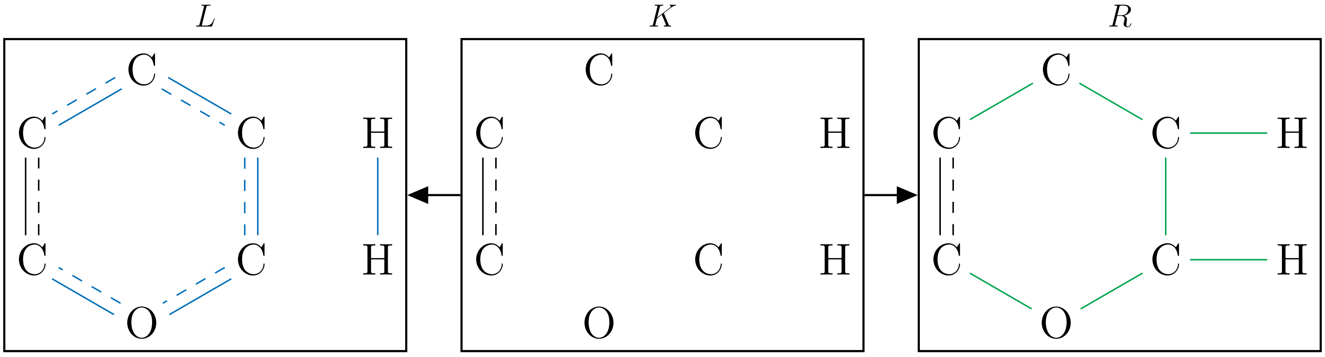

Files: out/1246_r_249_10300000_{L, K, R}

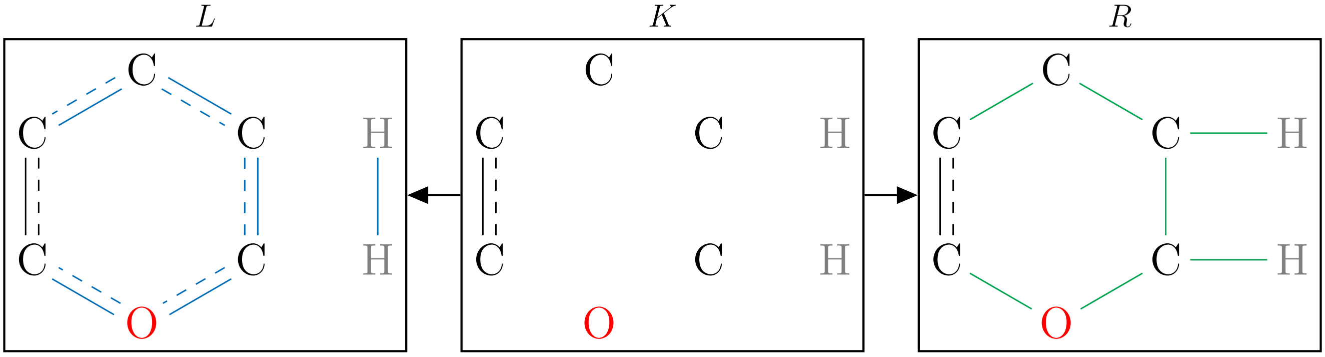

Files: out/1248_r_249_11300100_{L, K, R}

0.0.251 250

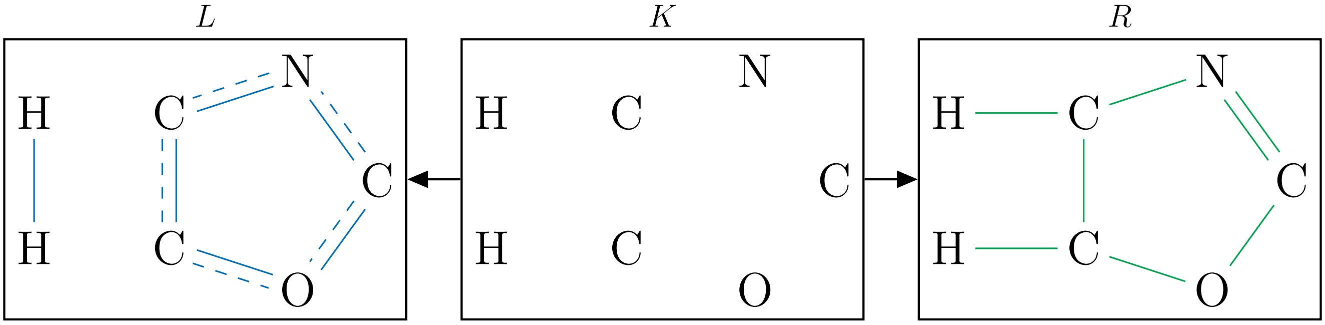

Files: out/1251_r_250_10300000_{L, K, R}

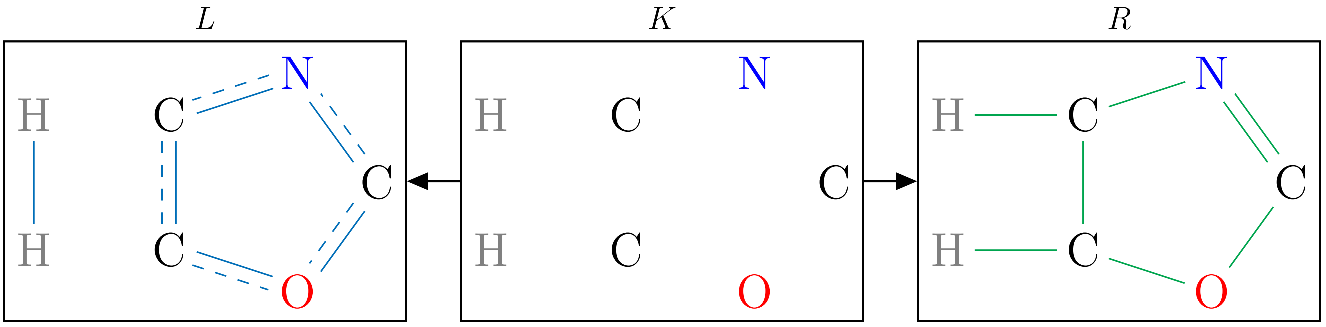

Files: out/1253_r_250_11300100_{L, K, R}

0.0.252 251

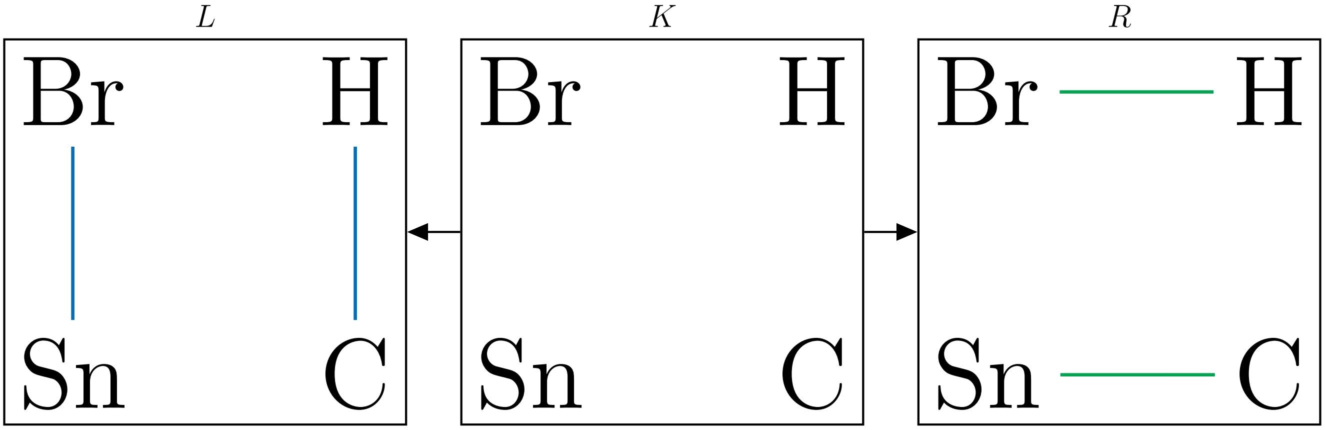

Files: out/1256_r_251_10300000_{L, K, R}

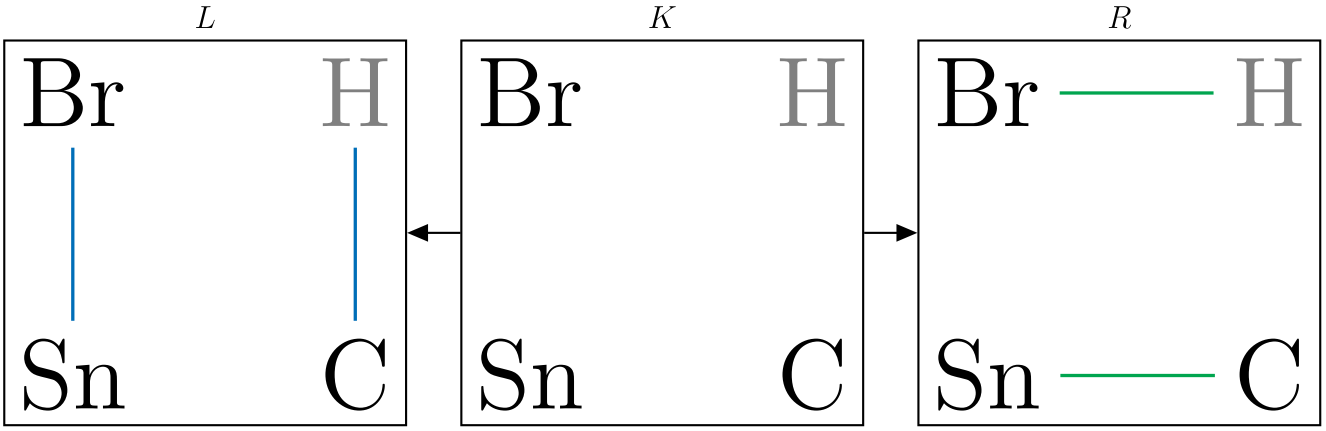

Files: out/1258_r_251_11300100_{L, K, R}

0.0.253 252

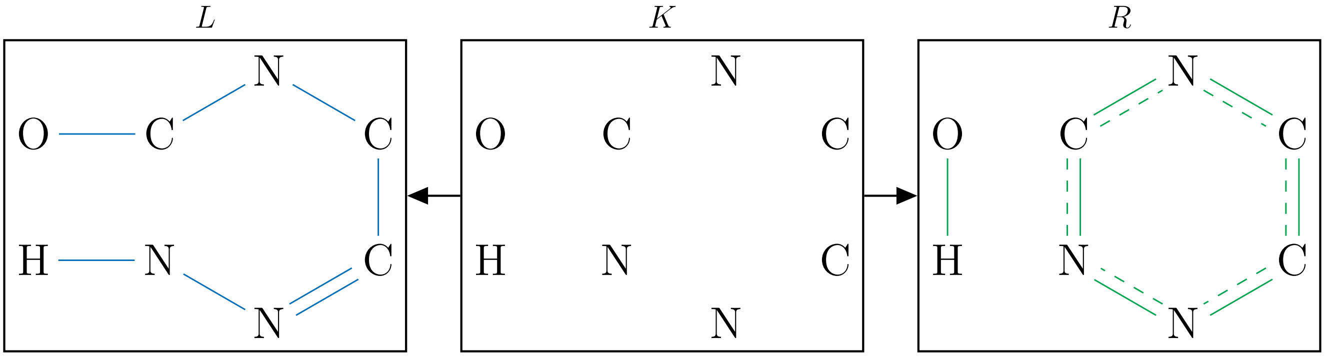

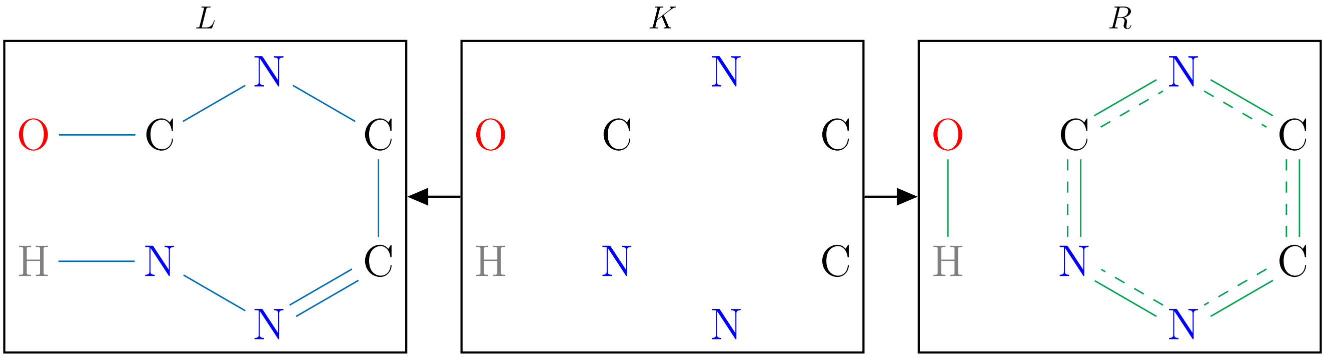

Files: out/1263_r_252_11300100_{L, K, R}

0.0.254 253

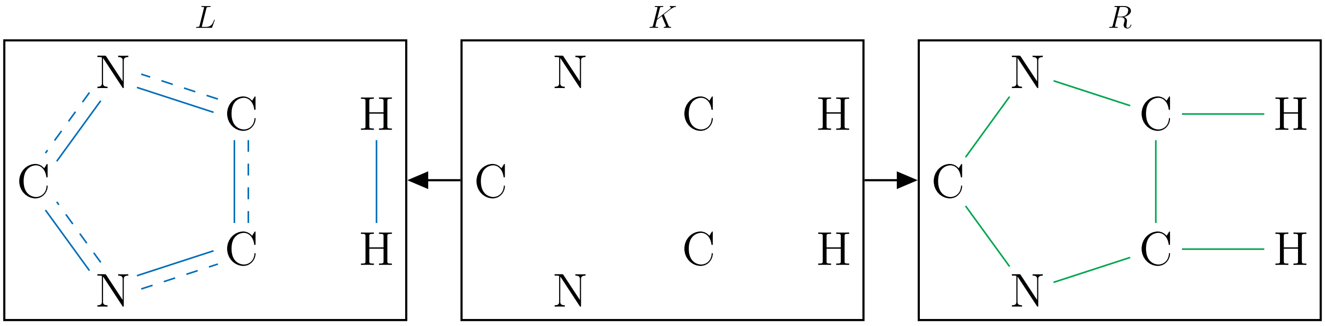

Files: out/1266_r_253_10300000_{L, K, R}

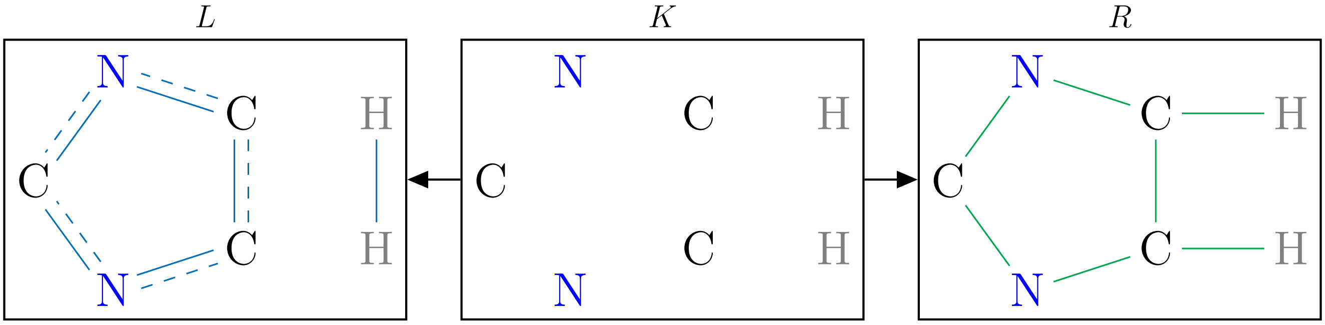

Files: out/1268_r_253_11300100_{L, K, R}

0.0.255 254

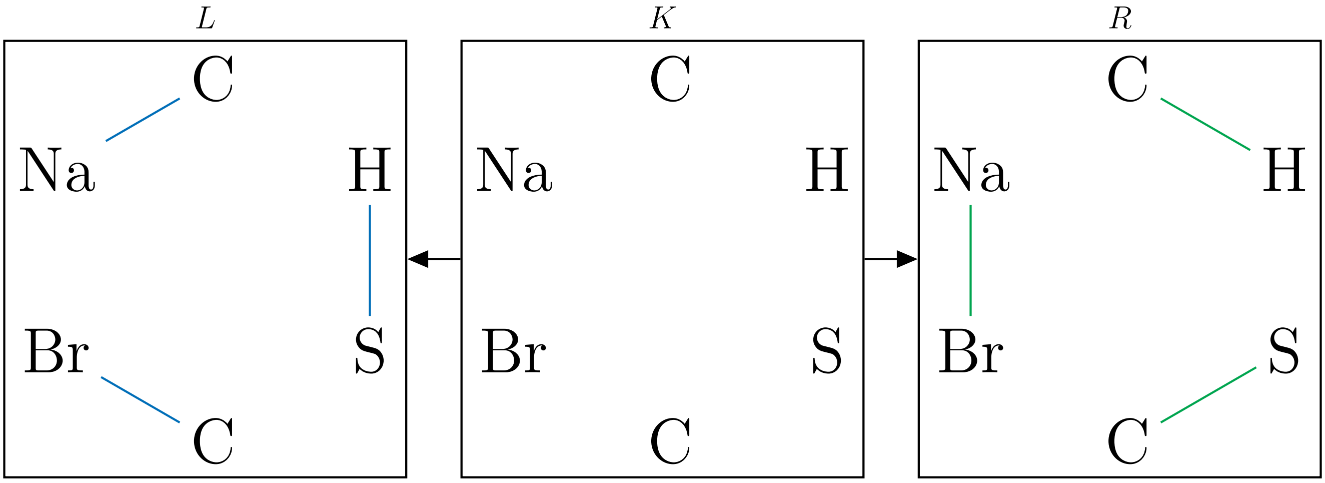

Files: out/1271_r_254_10300000_{L, K, R}

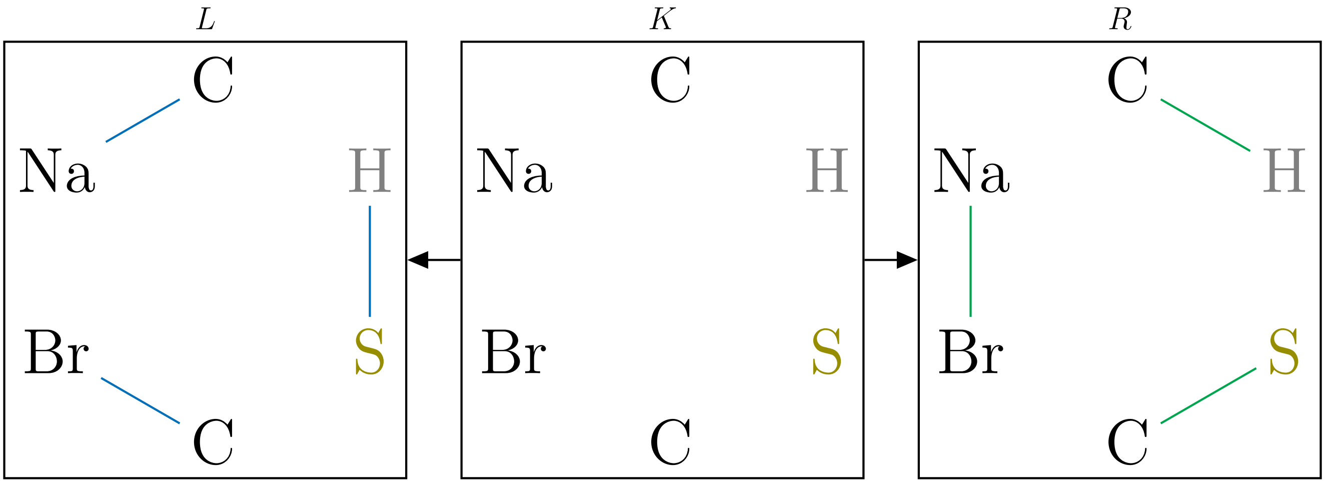

Files: out/1273_r_254_11300100_{L, K, R}

0.0.256 255

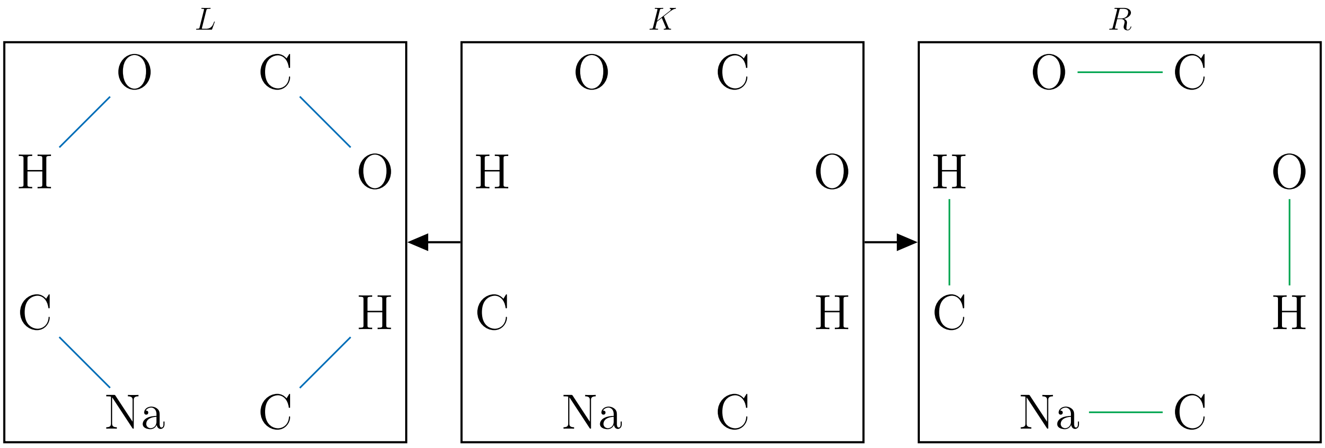

Files: out/1276_r_255_10300000_{L, K, R}

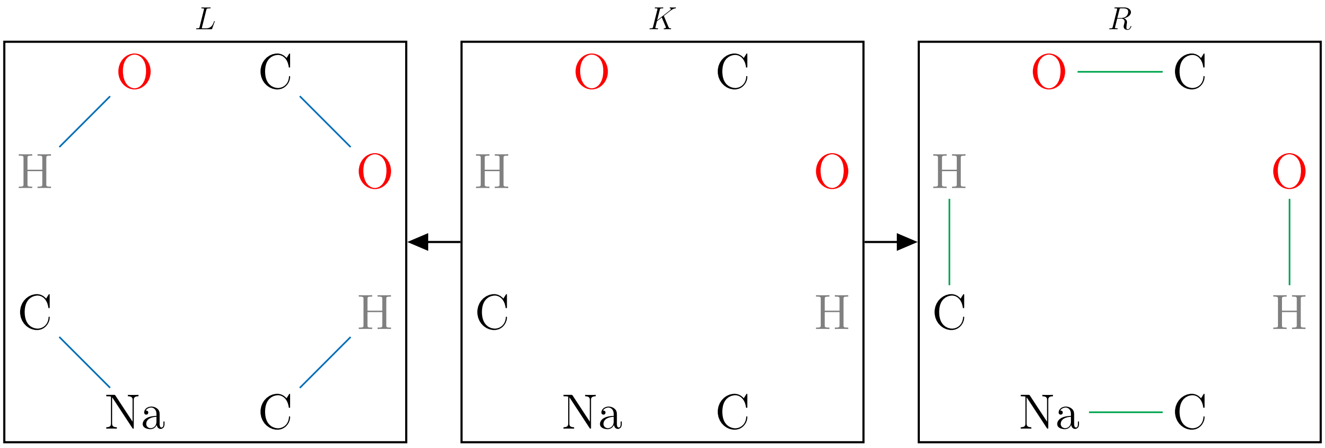

Files: out/1278_r_255_11300100_{L, K, R}

0.0.257 256

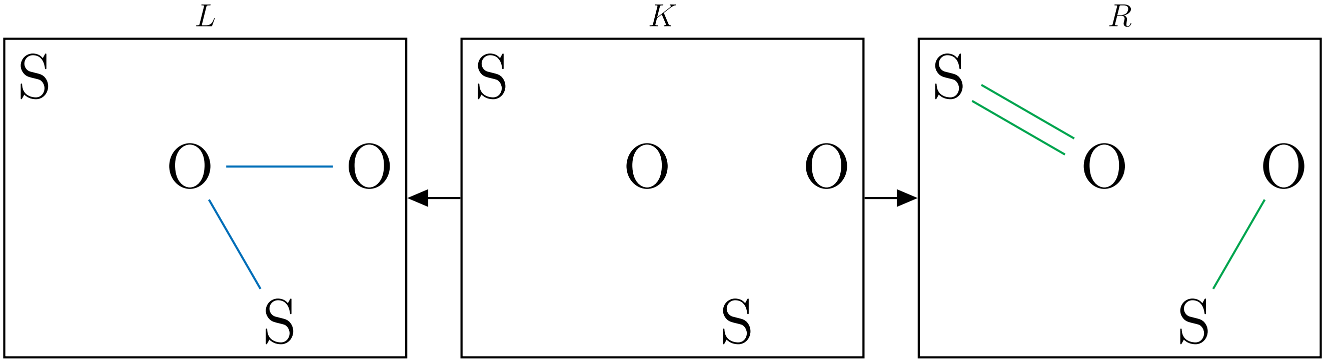

Files: out/1281_r_256_10300000_{L, K, R}

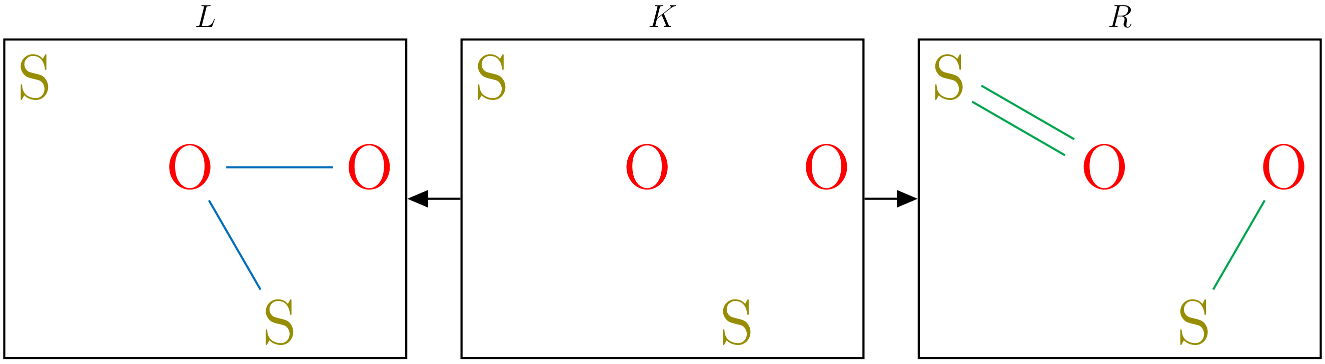

Files: out/1283_r_256_11300100_{L, K, R}

0.0.258 257

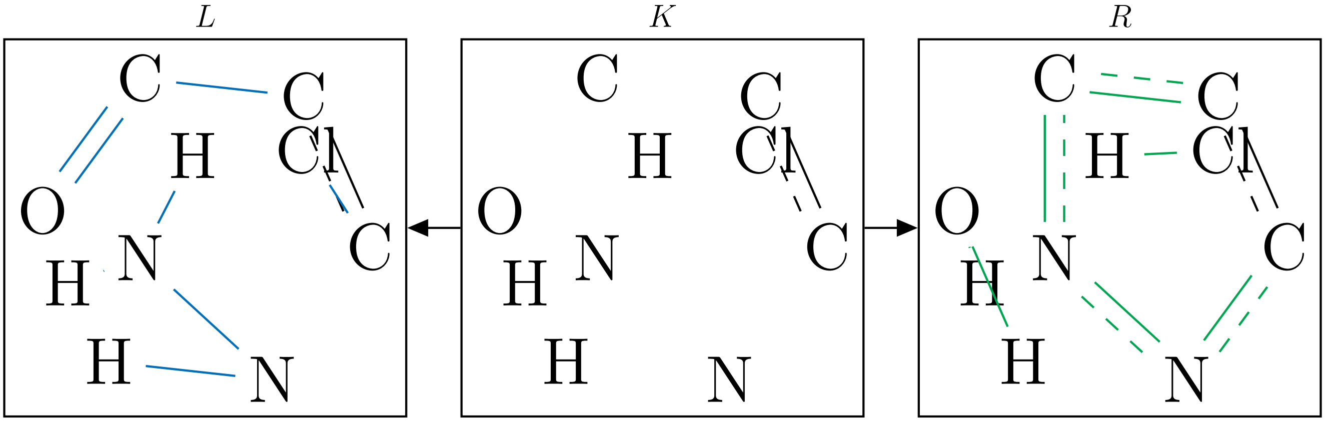

Files: out/1286_r_257_10300000_{L, K, R}

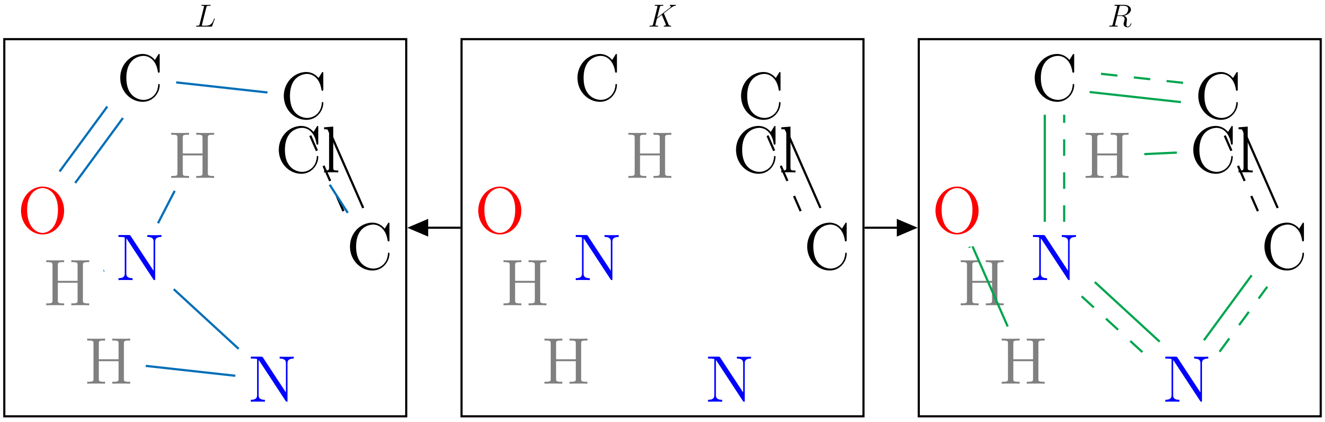

Files: out/1288_r_257_11300100_{L, K, R}

0.0.259 258

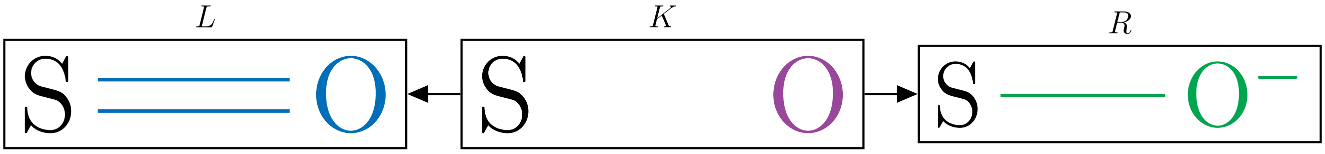

Files: out/1291_r_258_10300000_{L, K, R}

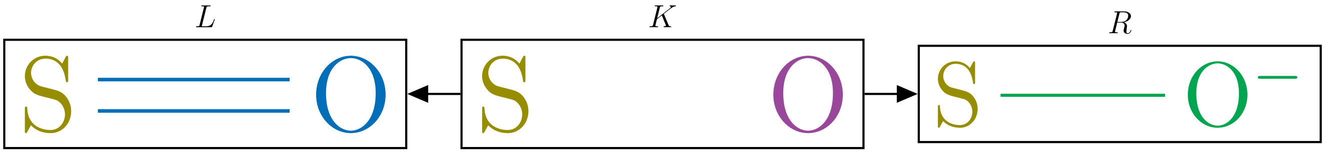

Files: out/1293_r_258_11300100_{L, K, R}

0.0.260 259

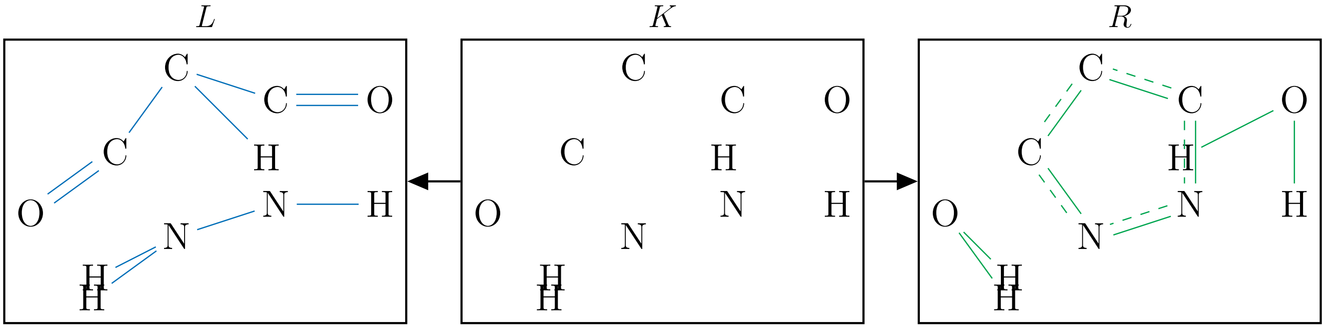

Files: out/1296_r_259_10300000_{L, K, R}

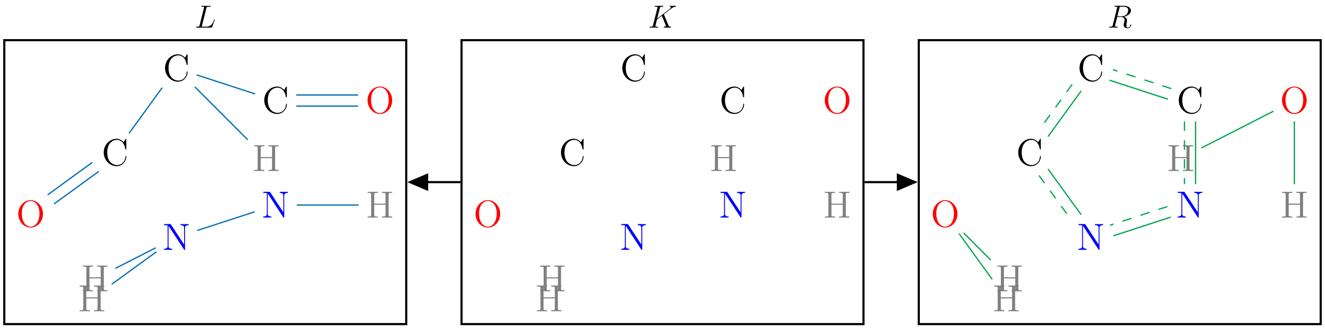

Files: out/1298_r_259_11300100_{L, K, R}

0.0.261 260

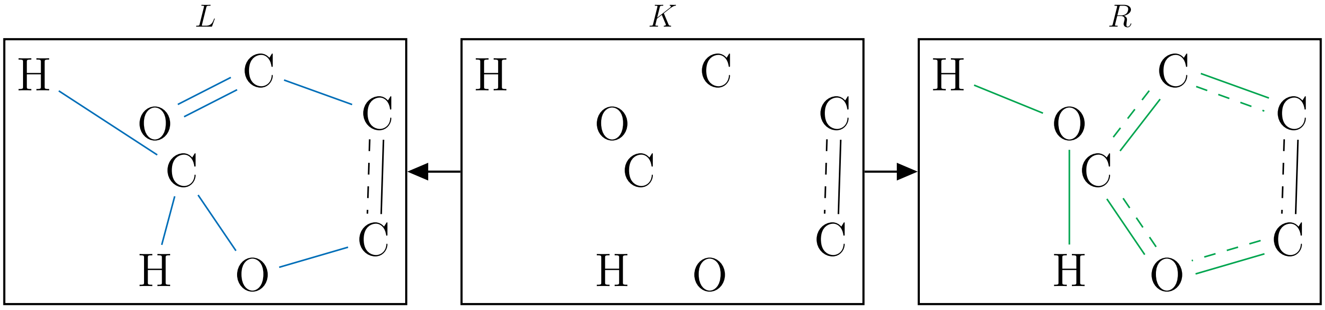

Files: out/1301_r_260_10300000_{L, K, R}

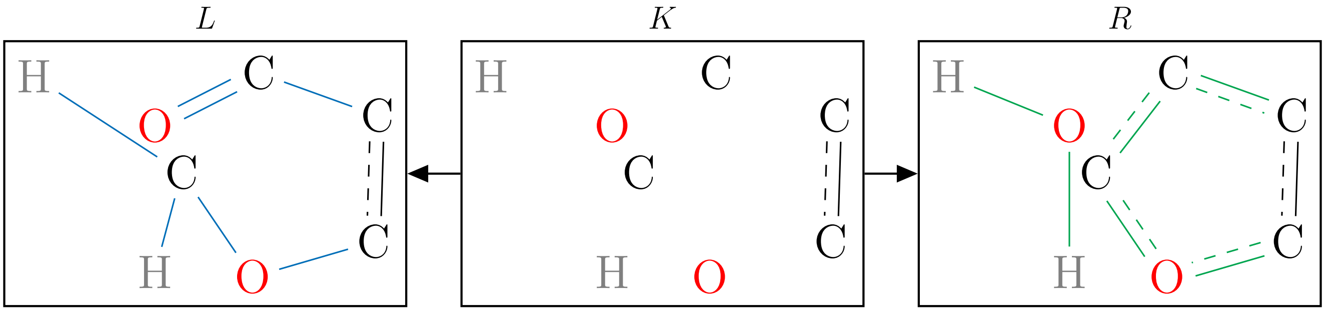

Files: out/1303_r_260_11300100_{L, K, R}

0.0.262 261

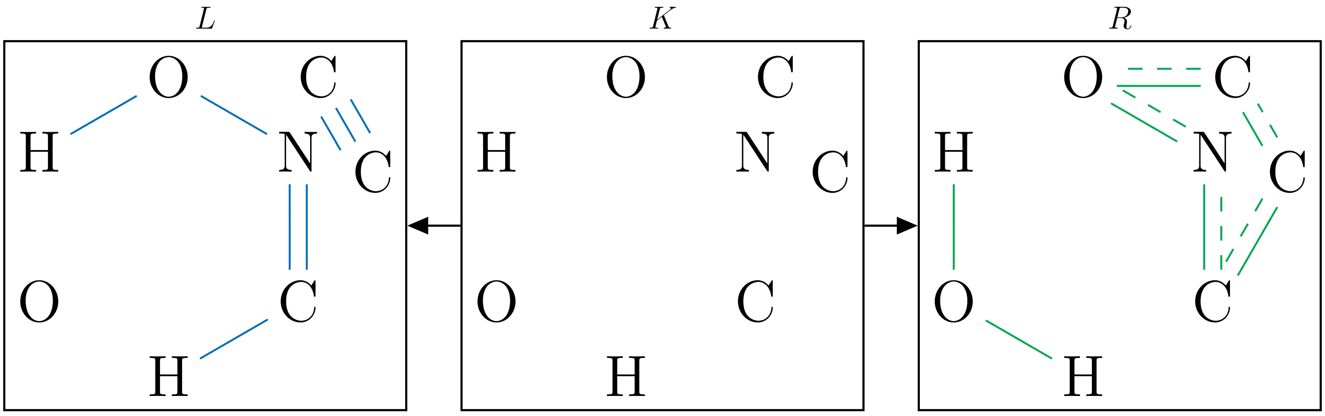

Files: out/1306_r_261_10300000_{L, K, R}

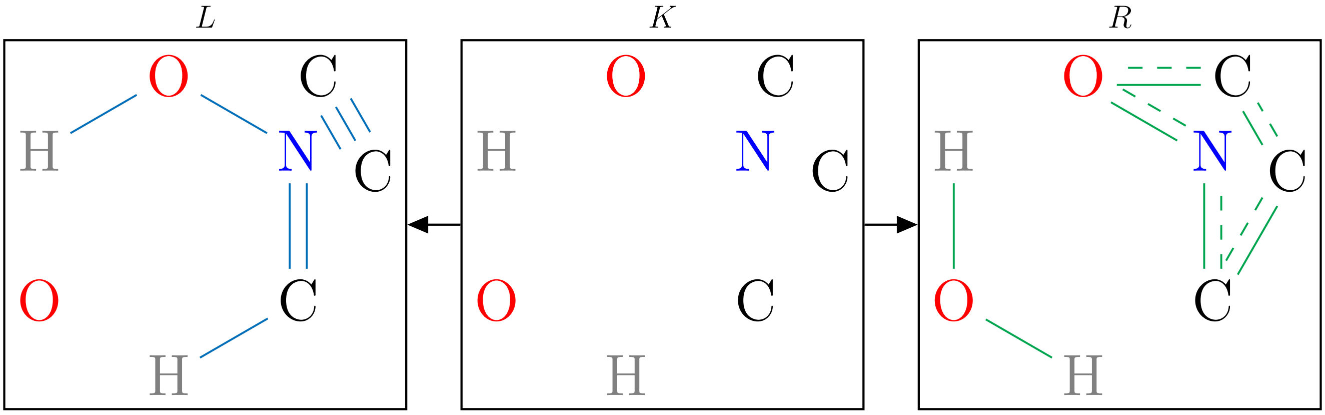

Files: out/1308_r_261_11300100_{L, K, R}

0.0.263 262

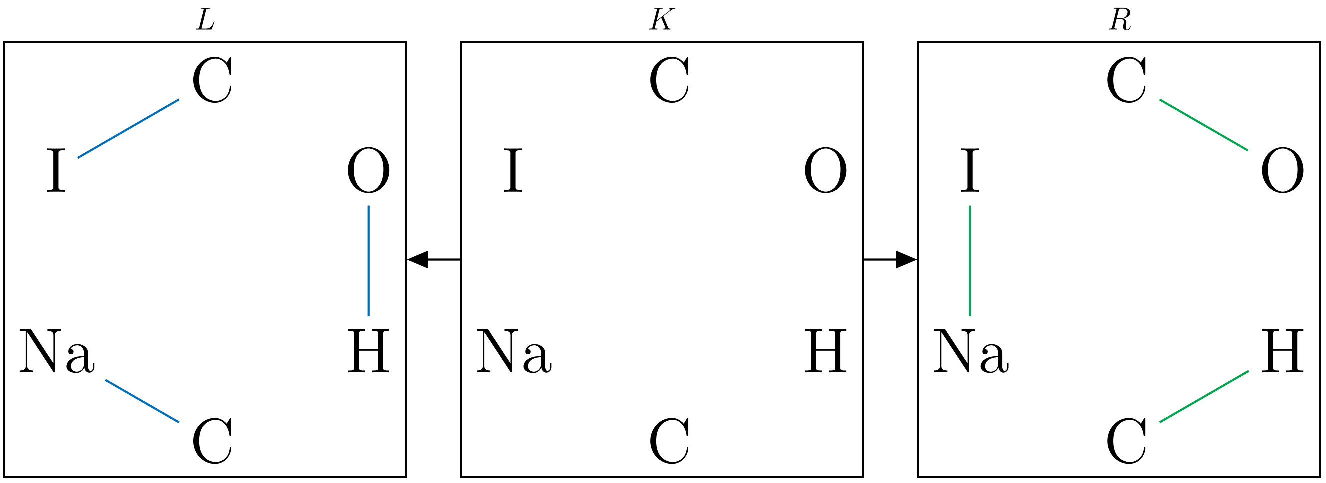

Files: out/1311_r_262_10300000_{L, K, R}

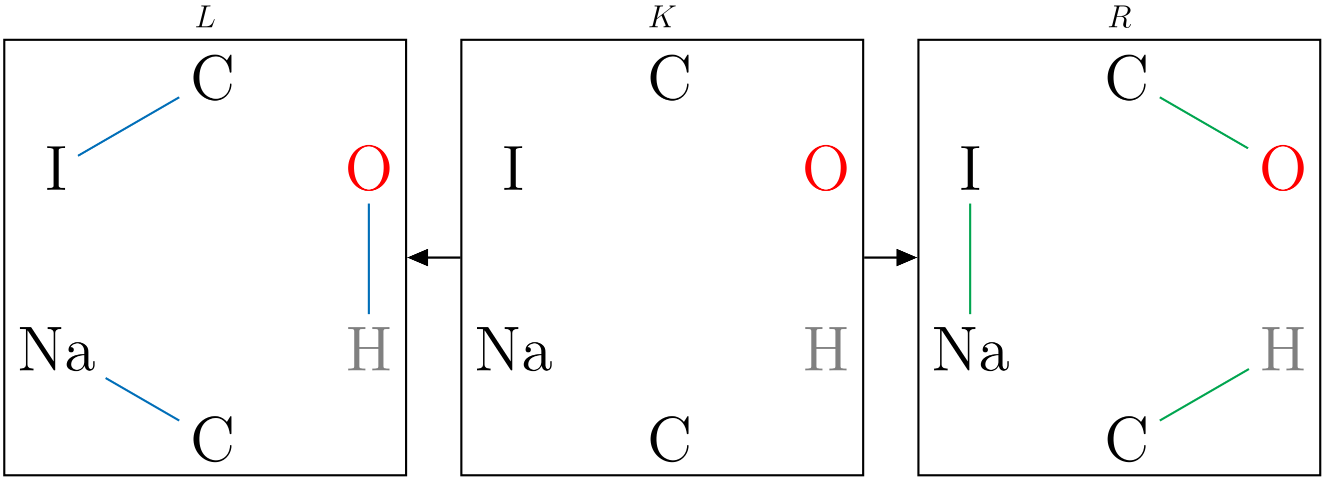

Files: out/1313_r_262_11300100_{L, K, R}

0.0.264 263

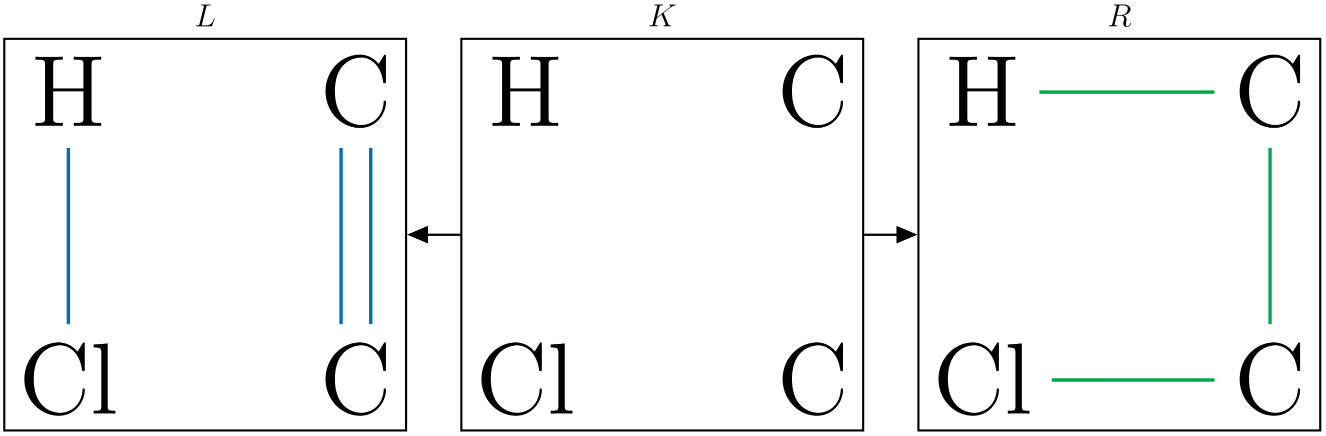

Files: out/1316_r_263_10300000_{L, K, R}

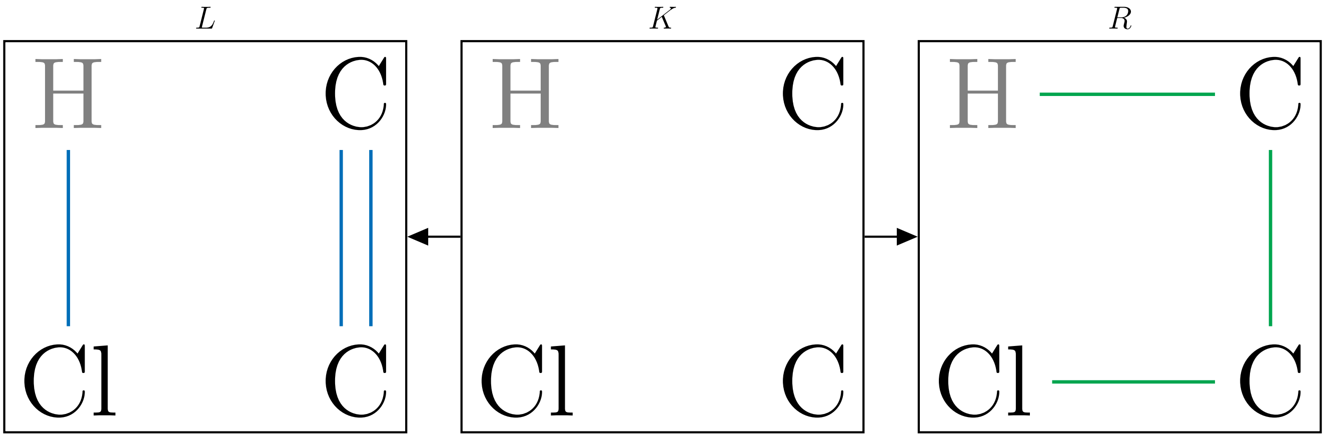

Files: out/1318_r_263_11300100_{L, K, R}

0.0.265 264

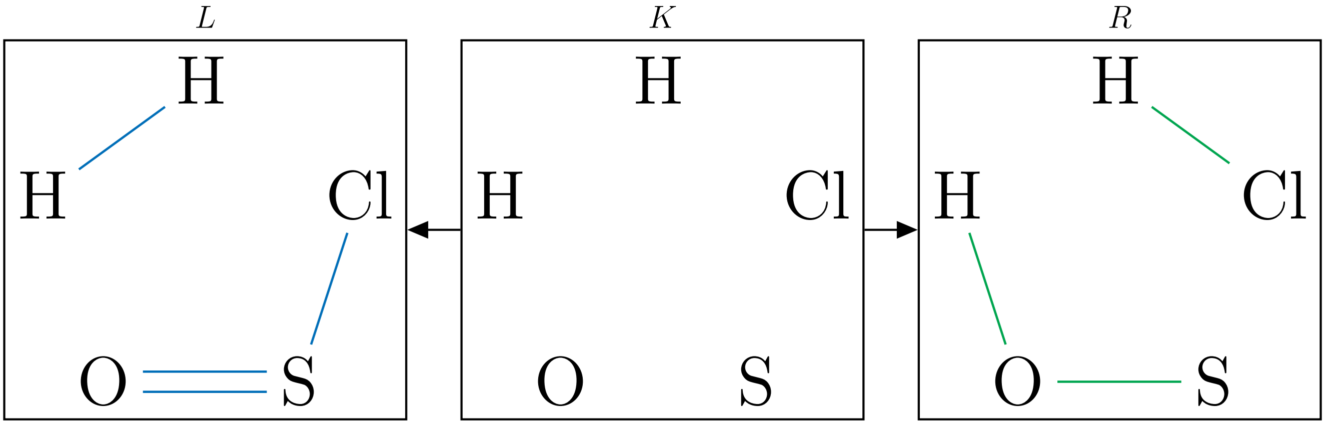

Files: out/1321_r_264_10300000_{L, K, R}

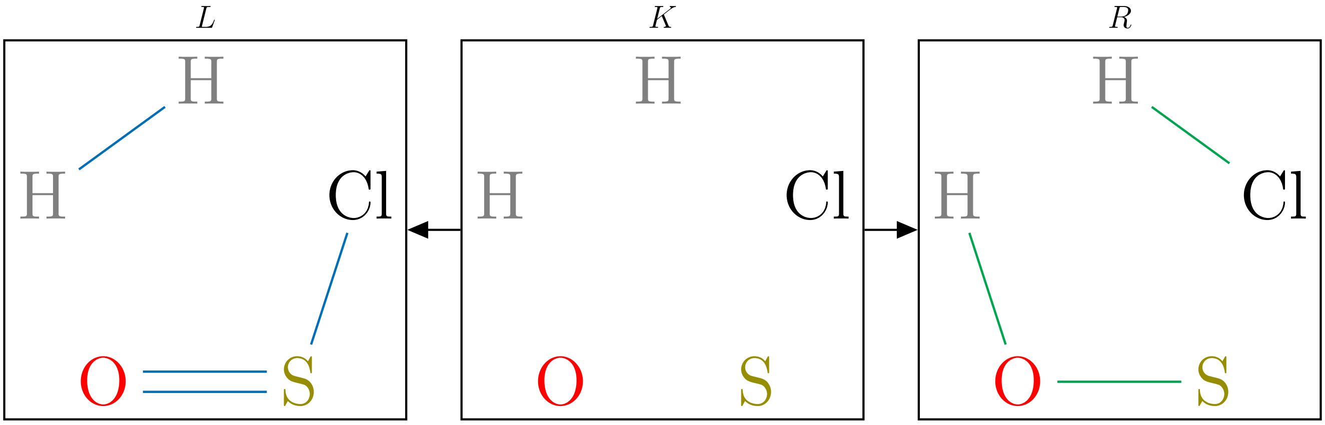

Files: out/1323_r_264_11300100_{L, K, R}

0.0.266 265

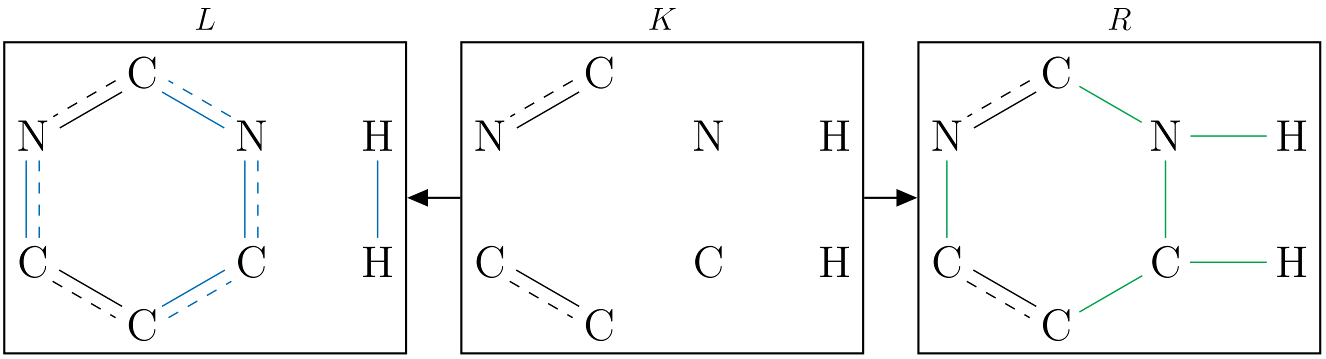

Files: out/1326_r_265_10300000_{L, K, R}

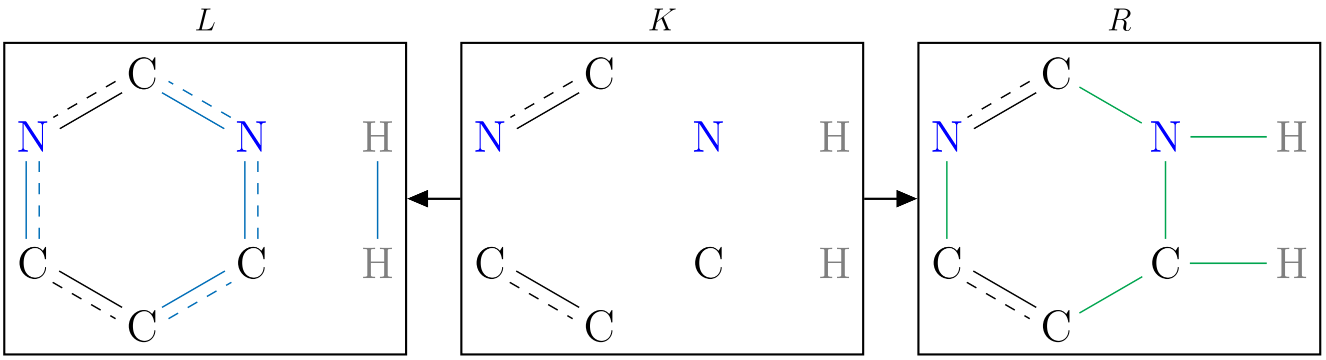

Files: out/1328_r_265_11300100_{L, K, R}

0.0.267 266

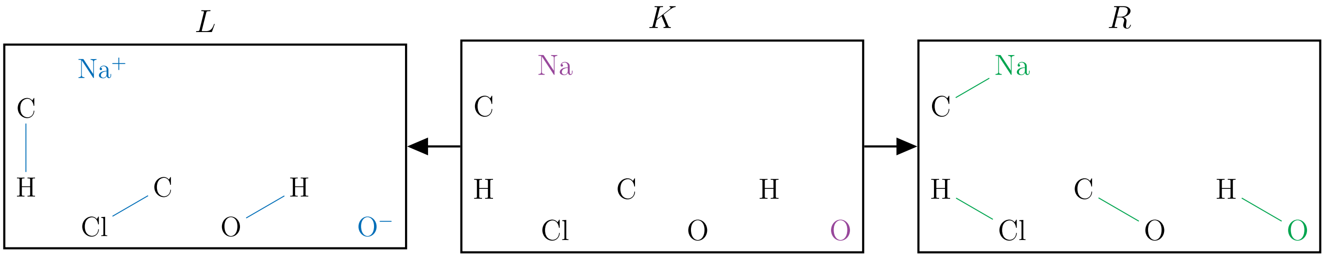

Files: out/1331_r_266_10300000_{L, K, R}

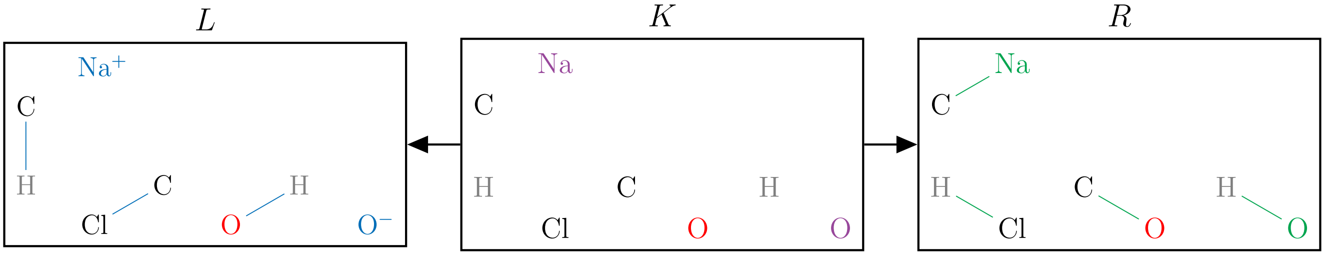

Files: out/1333_r_266_11300100_{L, K, R}

0.0.268 267

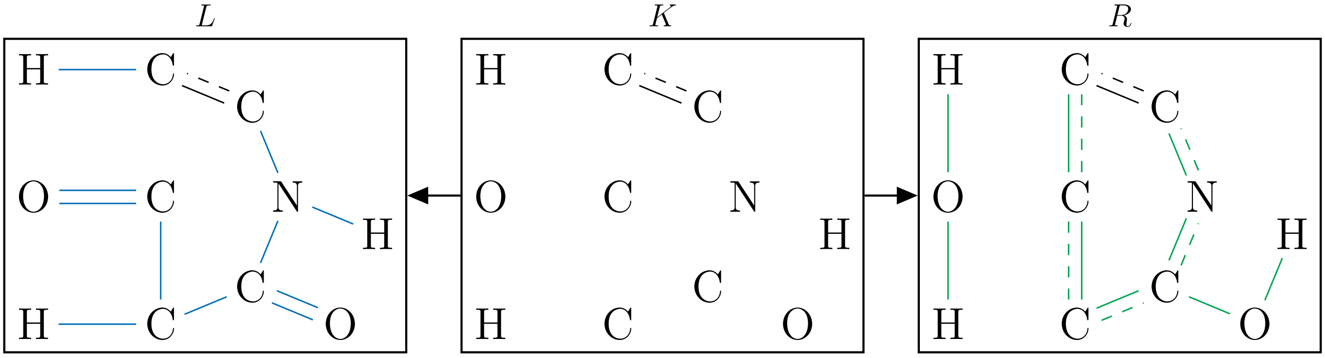

Files: out/1336_r_267_10300000_{L, K, R}

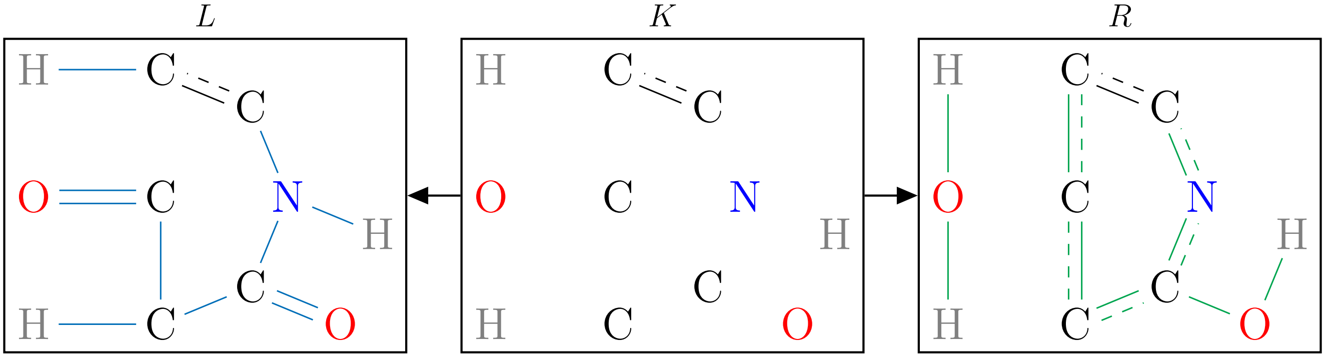

Files: out/1338_r_267_11300100_{L, K, R}

0.0.269 268

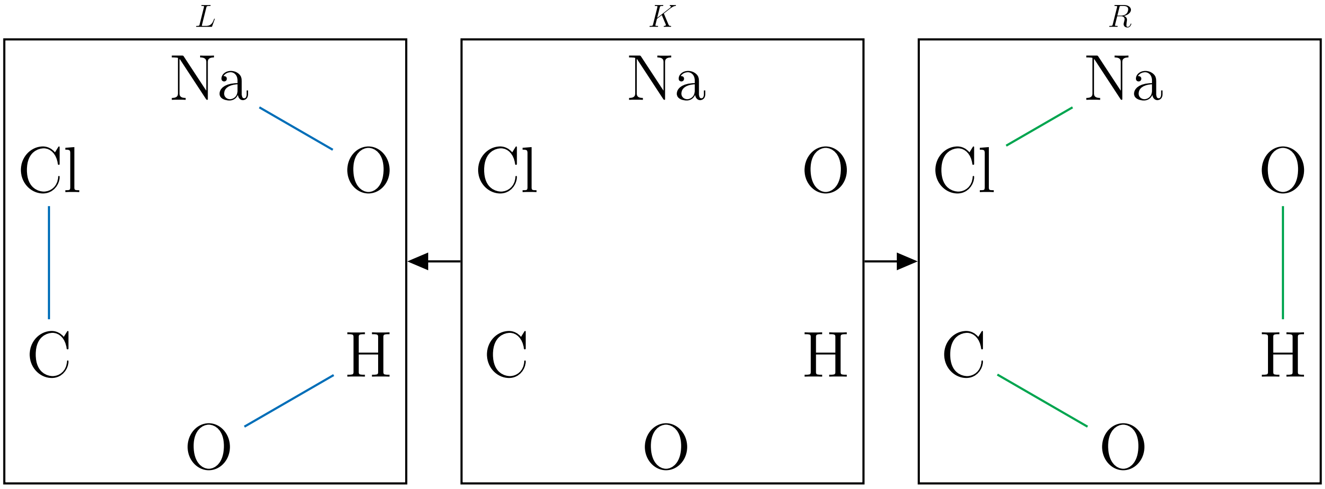

Files: out/1341_r_268_10300000_{L, K, R}

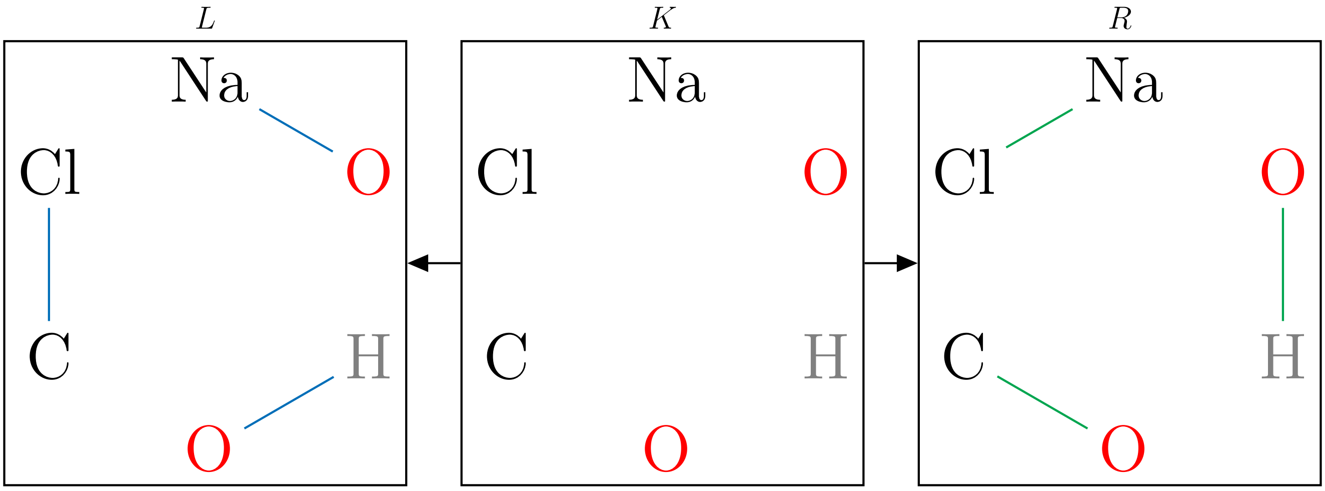

Files: out/1343_r_268_11300100_{L, K, R}

0.0.270 269

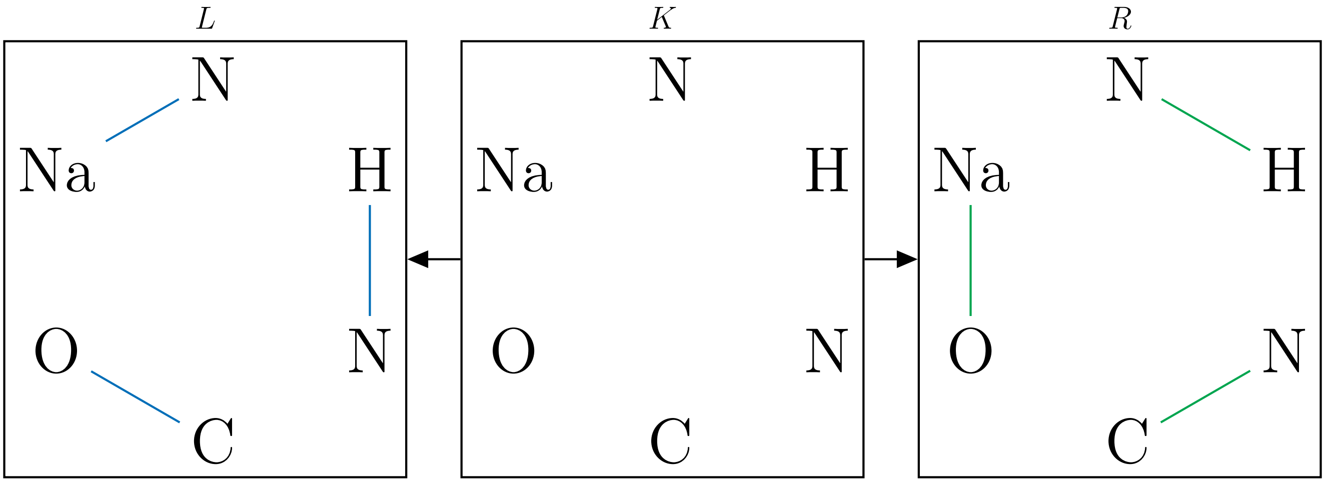

Files: out/1346_r_269_10300000_{L, K, R}

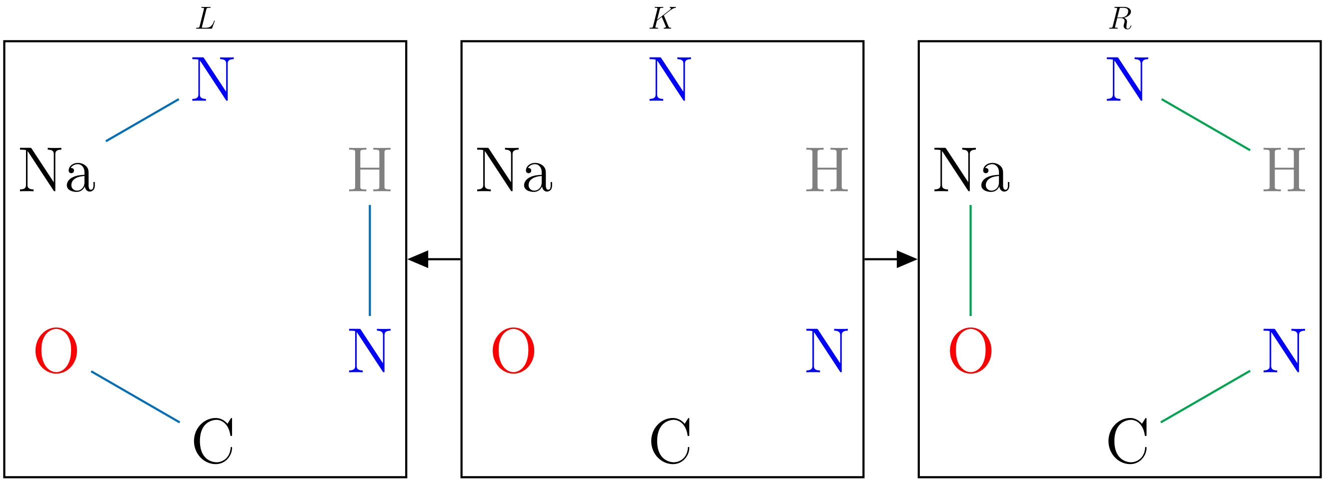

Files: out/1348_r_269_11300100_{L, K, R}

0.0.271 270

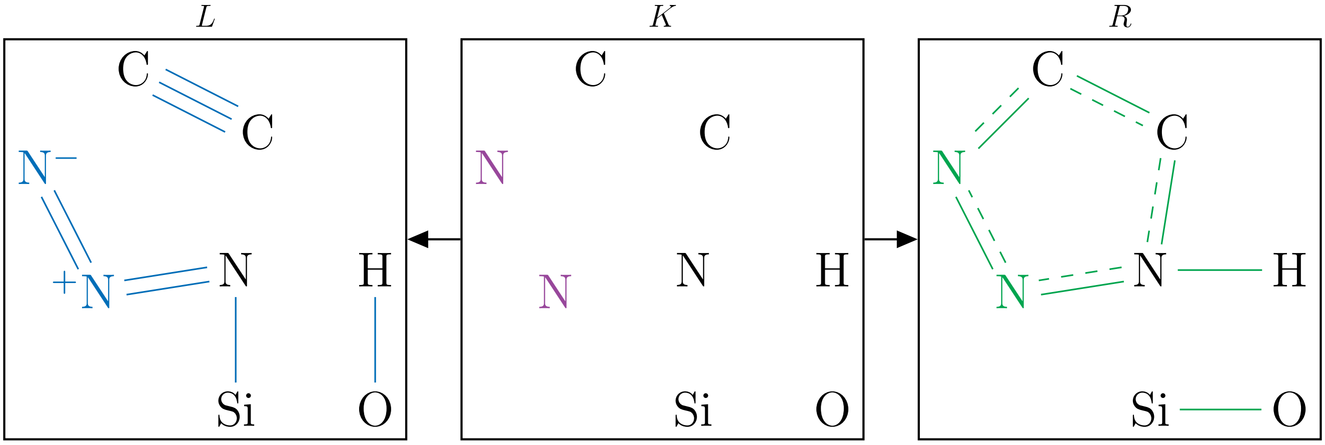

Files: out/1351_r_270_10300000_{L, K, R}

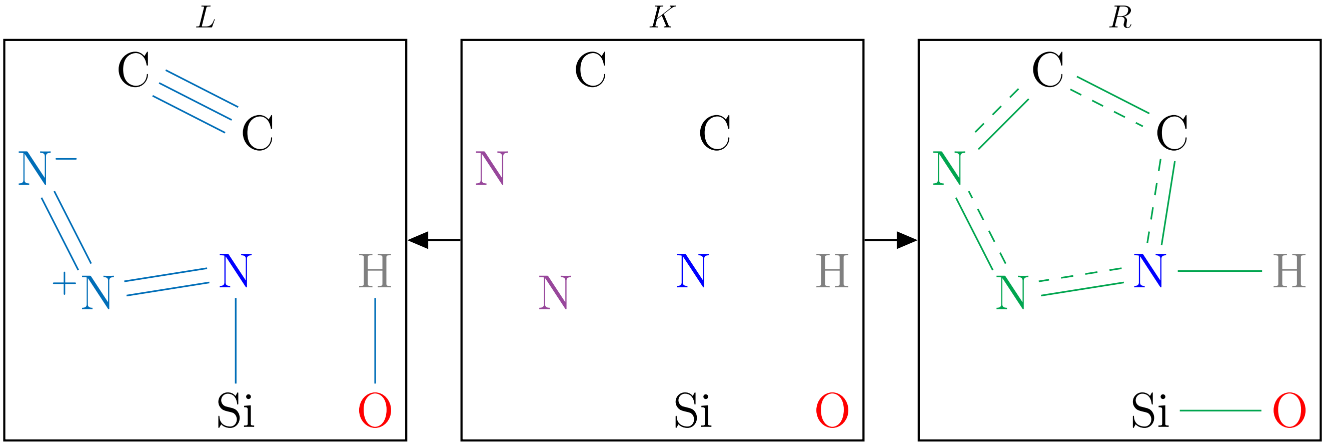

Files: out/1353_r_270_11300100_{L, K, R}

0.0.272 271

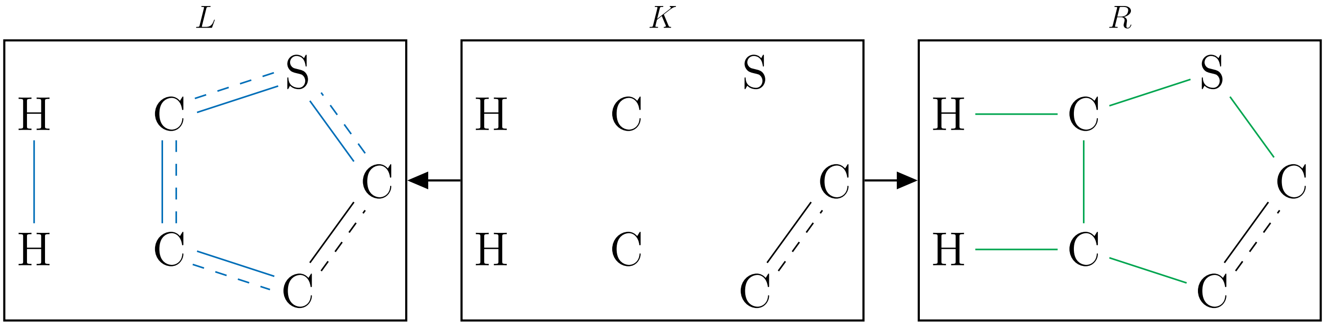

Files: out/1356_r_271_10300000_{L, K, R}

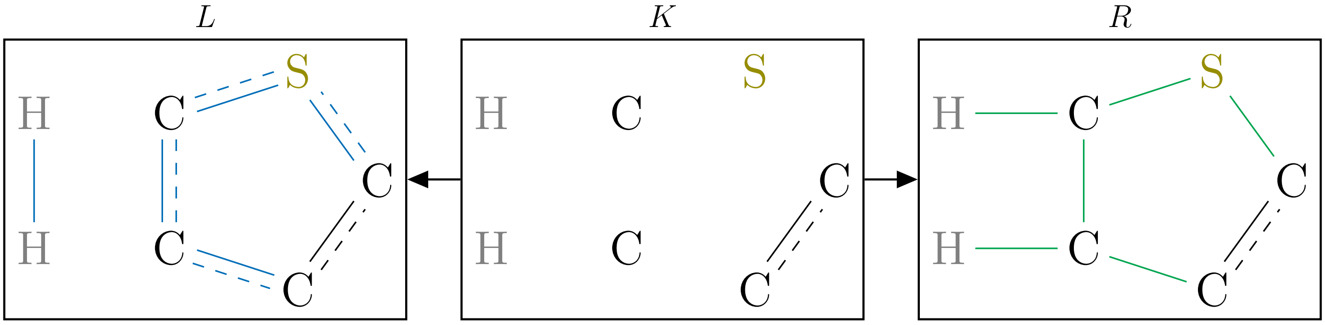

Files: out/1358_r_271_11300100_{L, K, R}

0.0.273 272

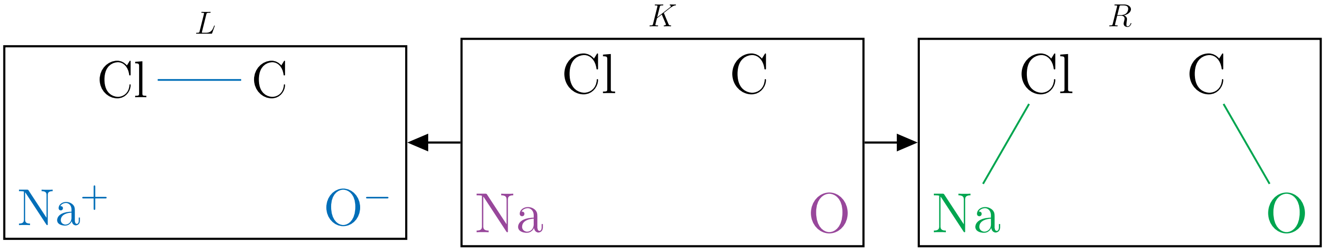

Files: out/1361_r_272_10300000_{L, K, R}

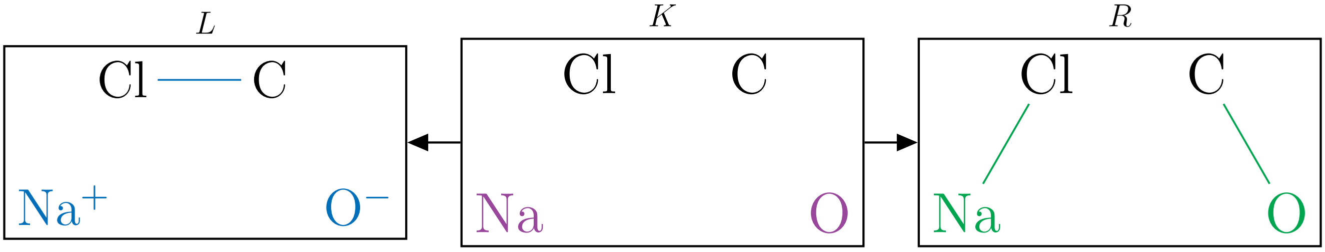

Files: out/1363_r_272_11300100_{L, K, R}

0.0.274 273

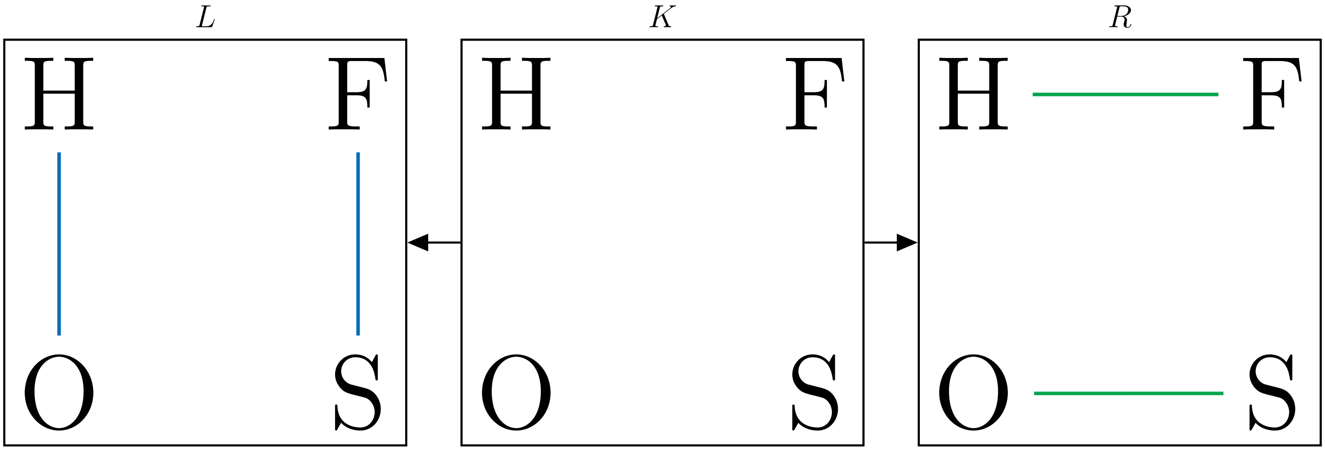

Files: out/1366_r_273_10300000_{L, K, R}

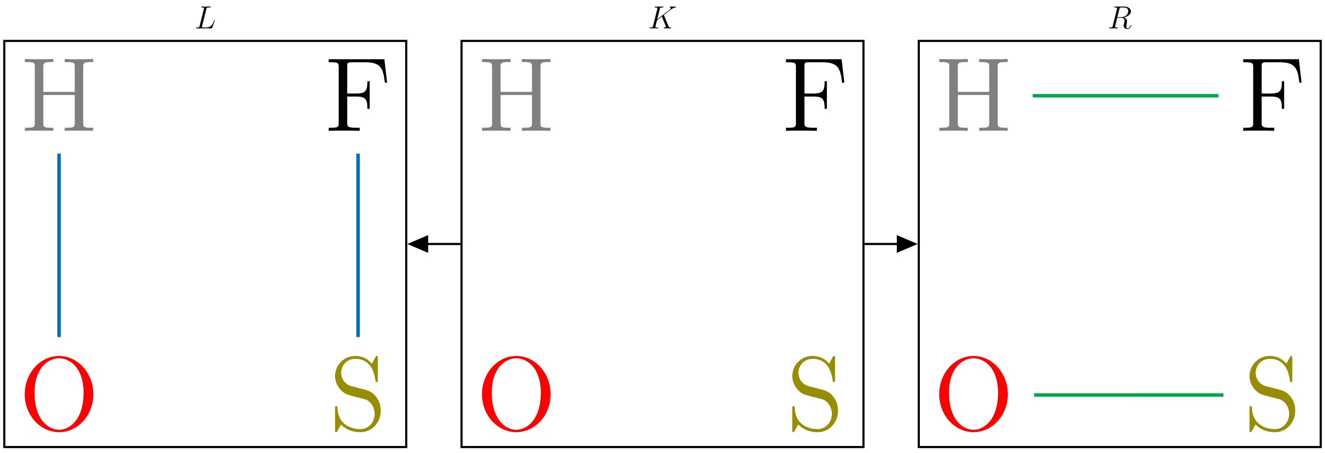

Files: out/1368_r_273_11300100_{L, K, R}

0.0.275 274

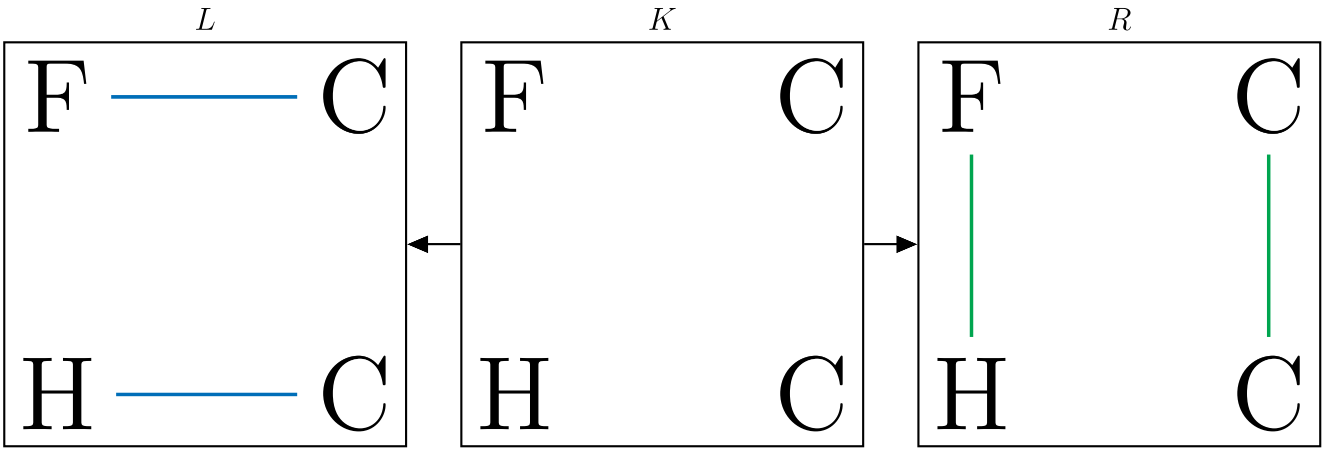

Files: out/1371_r_274_10300000_{L, K, R}

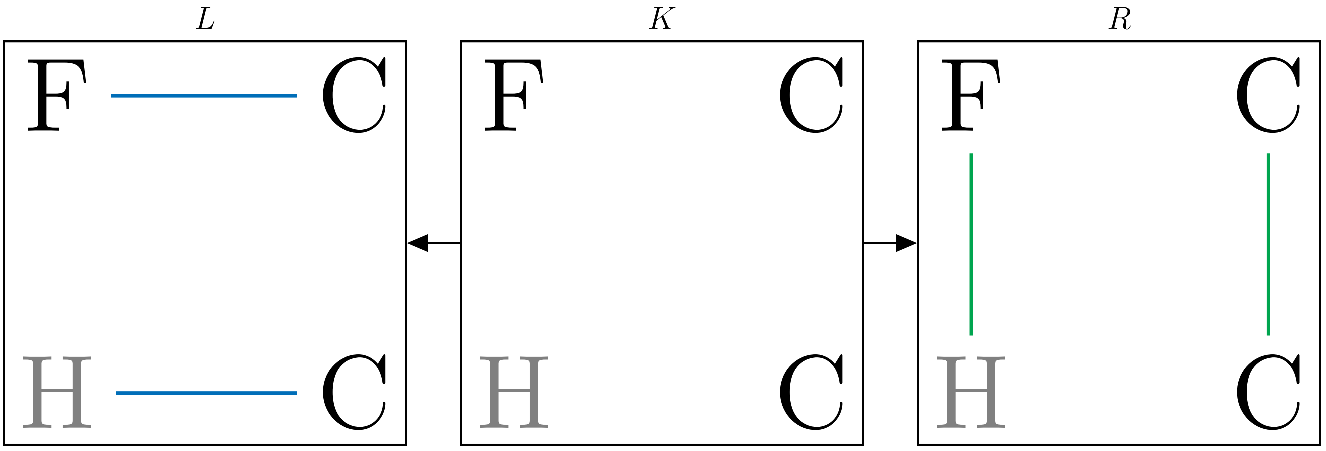

Files: out/1373_r_274_11300100_{L, K, R}

0.0.276 275

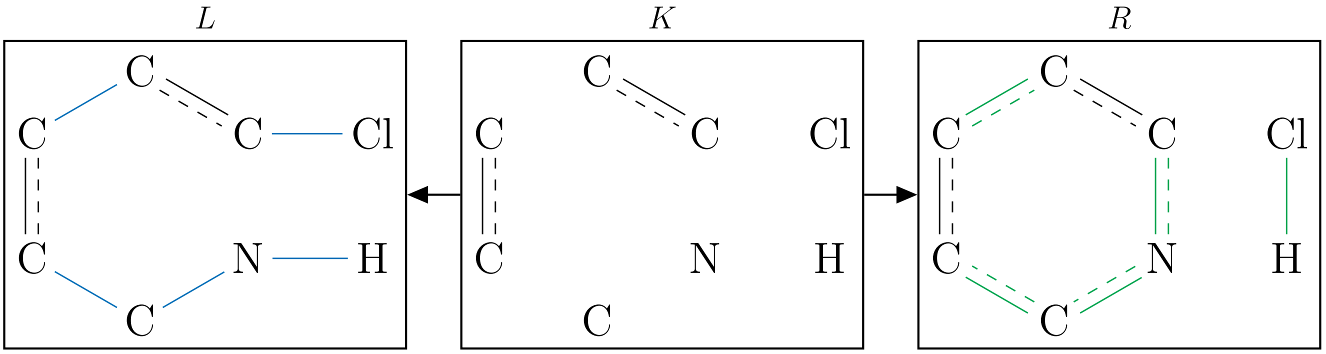

Files: out/1376_r_275_10300000_{L, K, R}

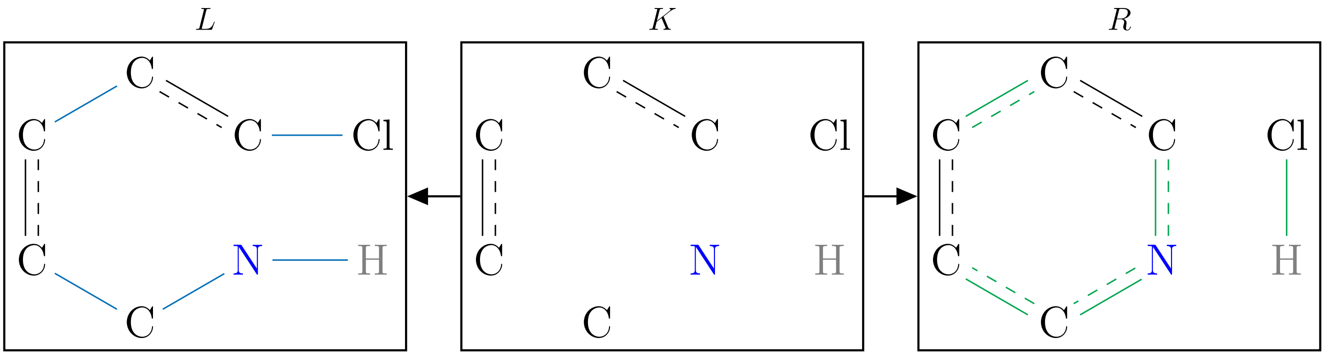

Files: out/1378_r_275_11300100_{L, K, R}

0.0.277 276

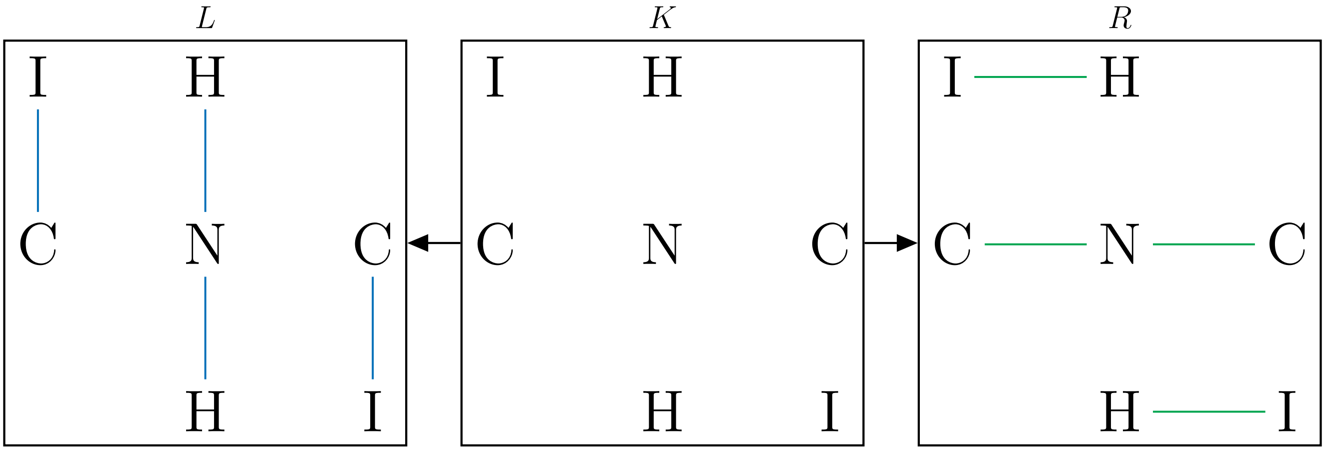

Files: out/1381_r_276_10300000_{L, K, R}

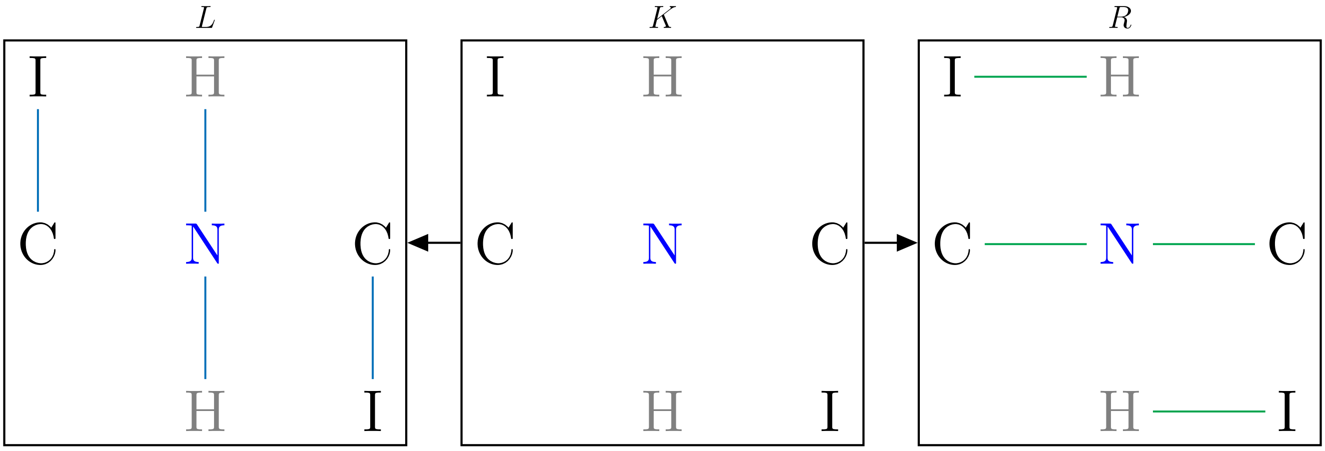

Files: out/1383_r_276_11300100_{L, K, R}

0.0.278 277

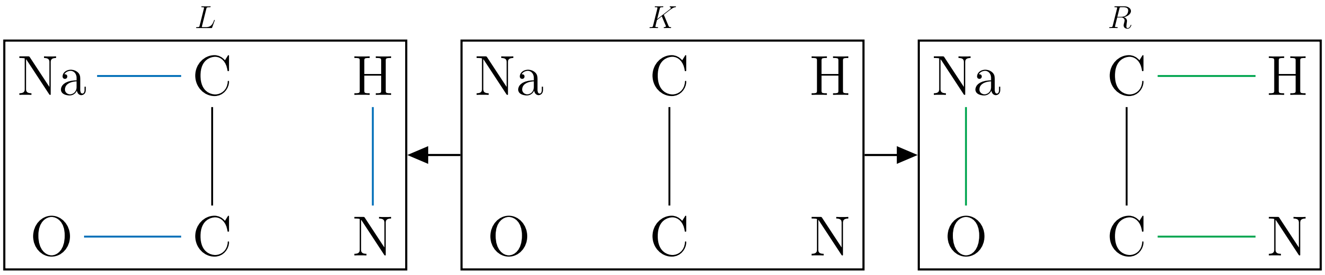

Files: out/1386_r_277_10300000_{L, K, R}

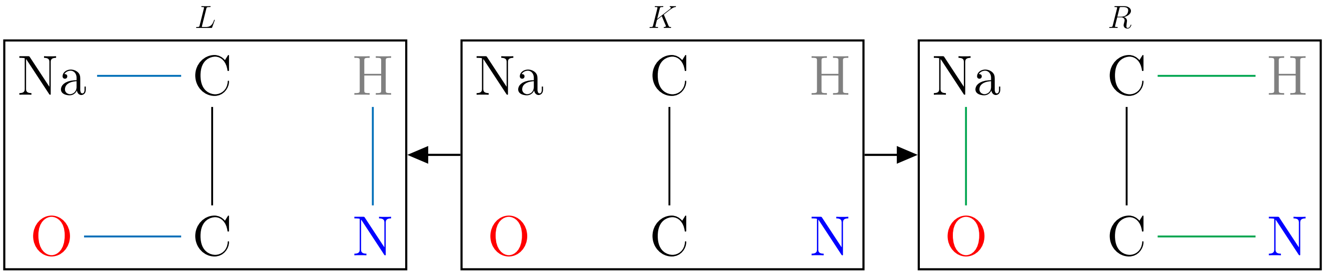

Files: out/1388_r_277_11300100_{L, K, R}

0.0.279 278

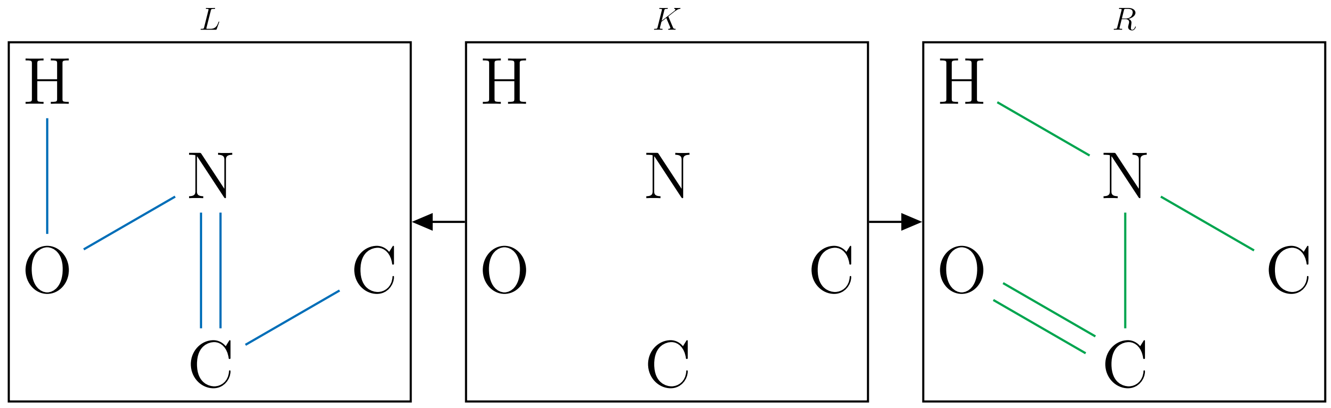

Files: out/1391_r_278_10300000_{L, K, R}

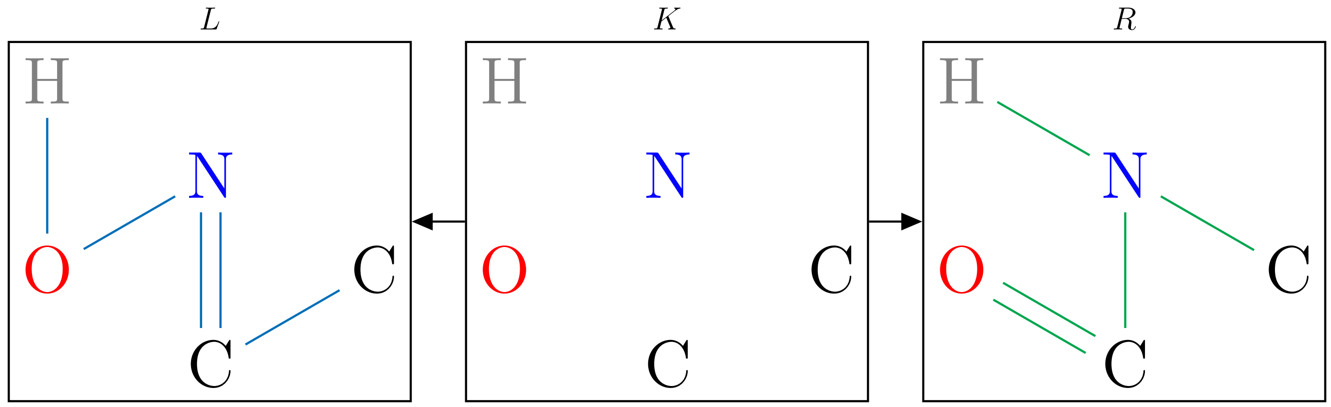

Files: out/1393_r_278_11300100_{L, K, R}

0.0.280 279

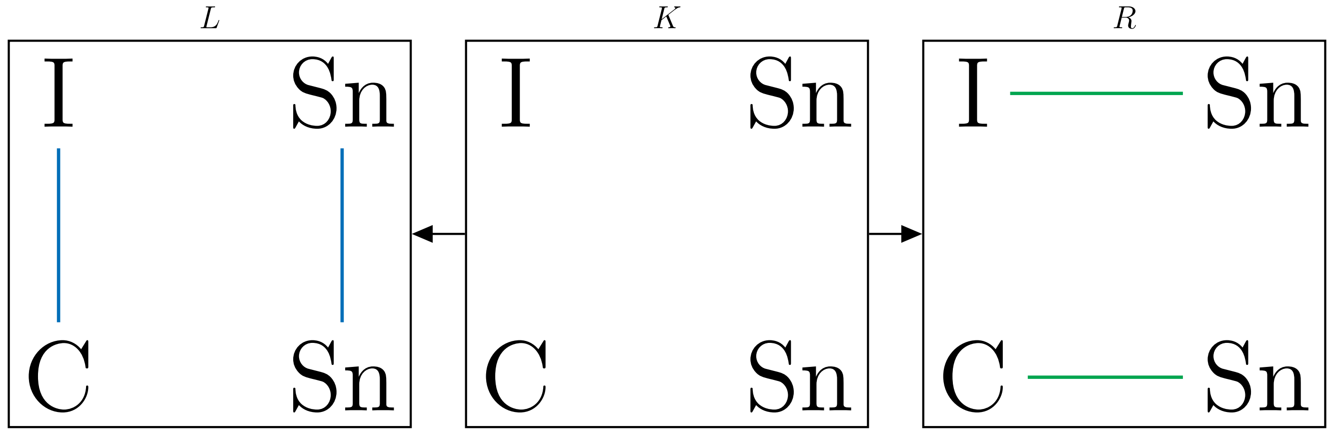

Files: out/1396_r_279_10300000_{L, K, R}

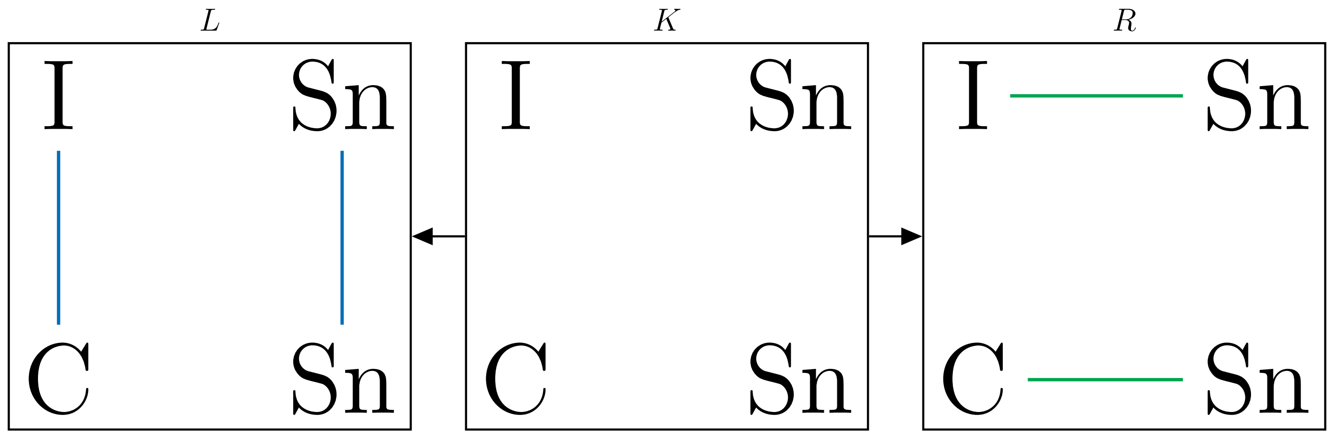

Files: out/1398_r_279_11300100_{L, K, R}

0.0.281 280

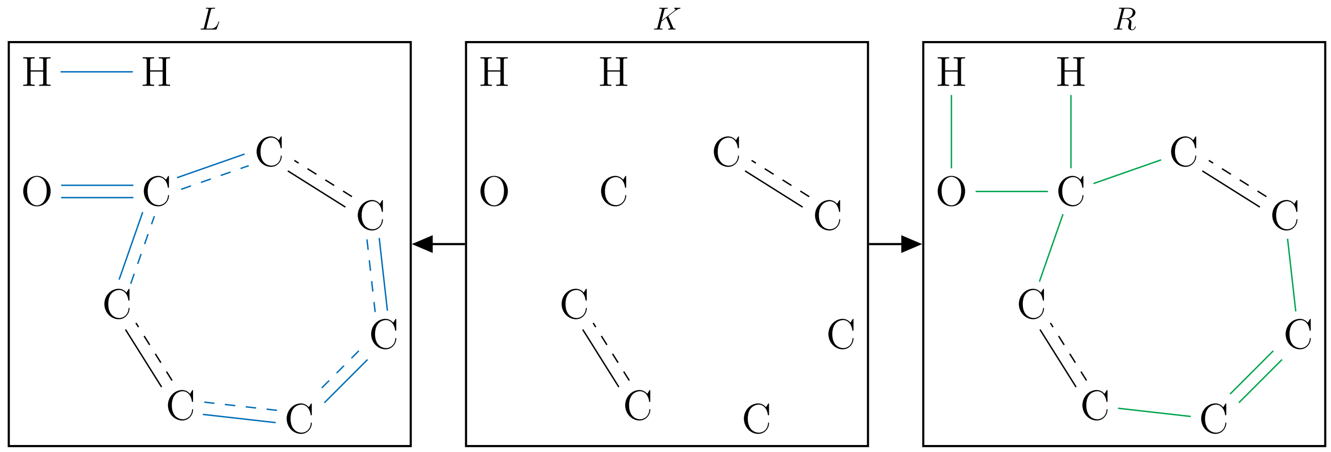

Files: out/1401_r_280_10300000_{L, K, R}

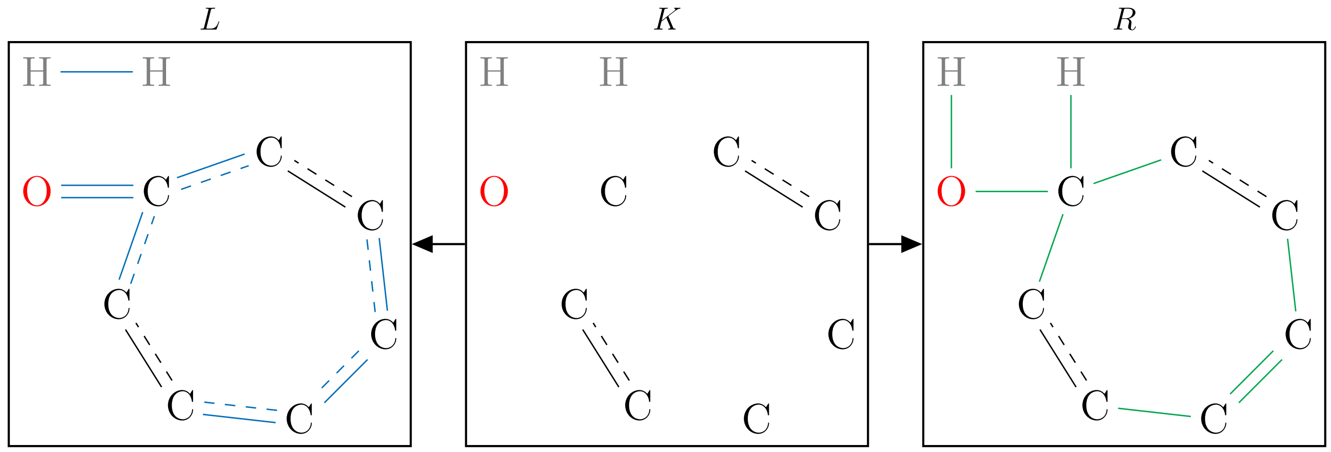

Files: out/1403_r_280_11300100_{L, K, R}

0.0.282 281

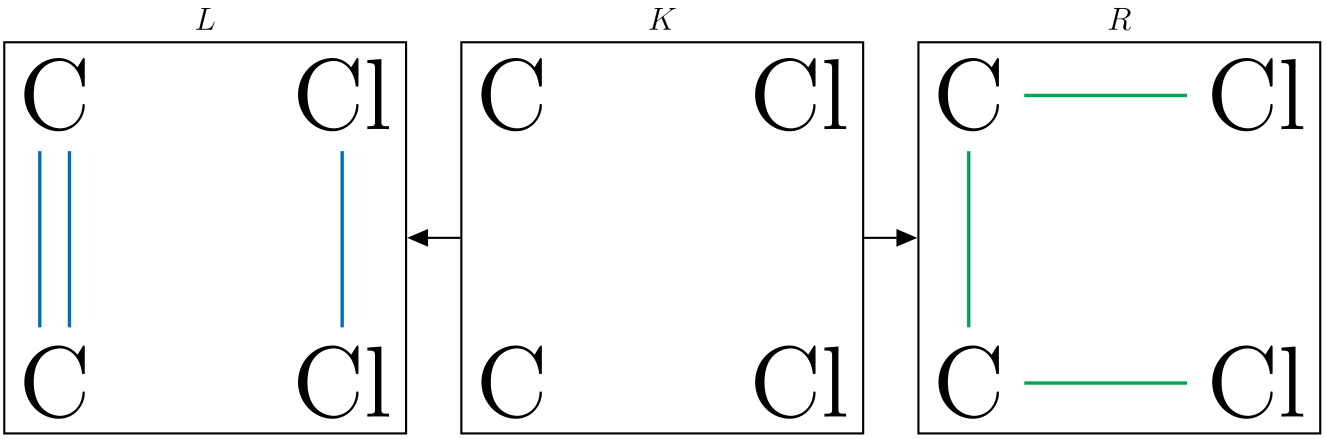

Files: out/1406_r_281_10300000_{L, K, R}

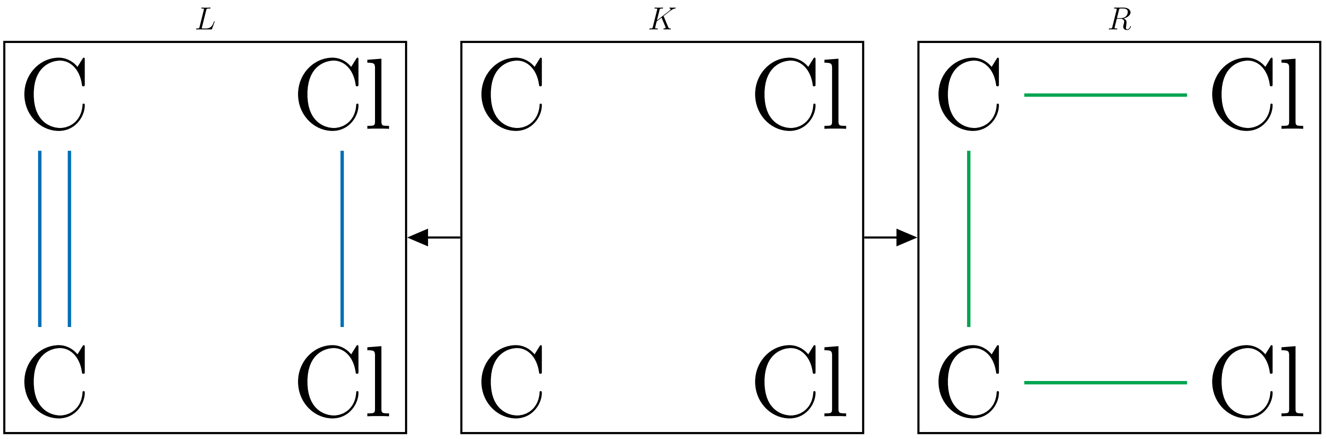

Files: out/1408_r_281_11300100_{L, K, R}

0.0.283 282

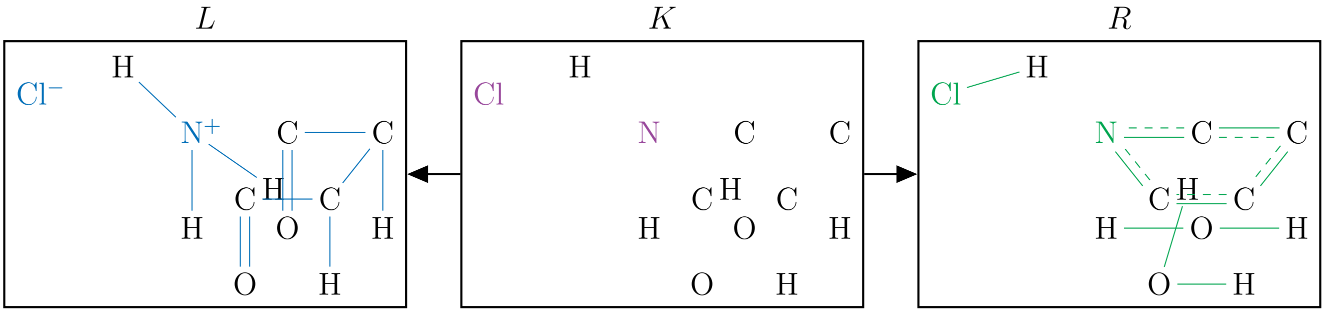

Files: out/1411_r_282_10300000_{L, K, R}

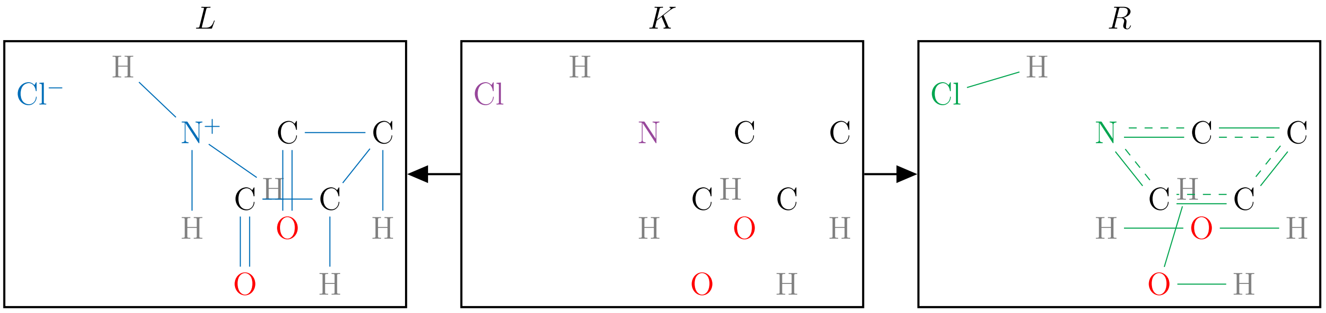

Files: out/1413_r_282_11300100_{L, K, R}

0.0.284 283

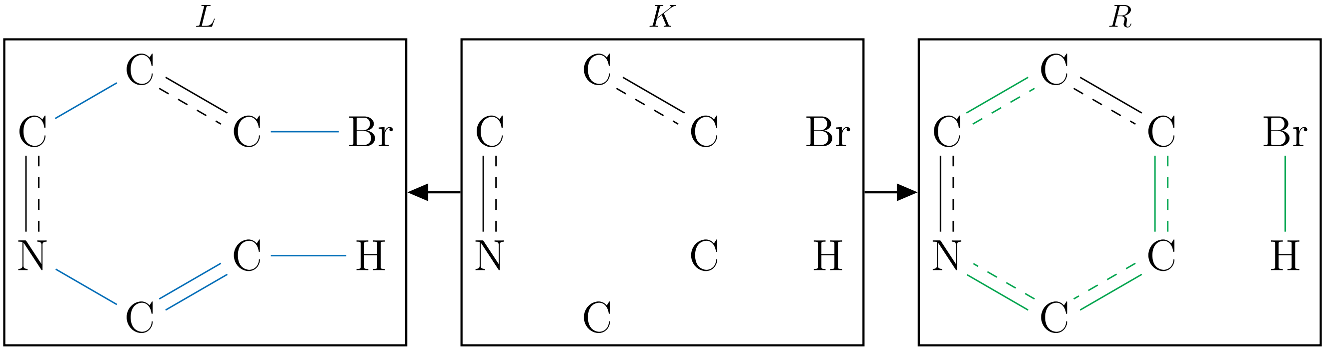

Files: out/1416_r_283_10300000_{L, K, R}

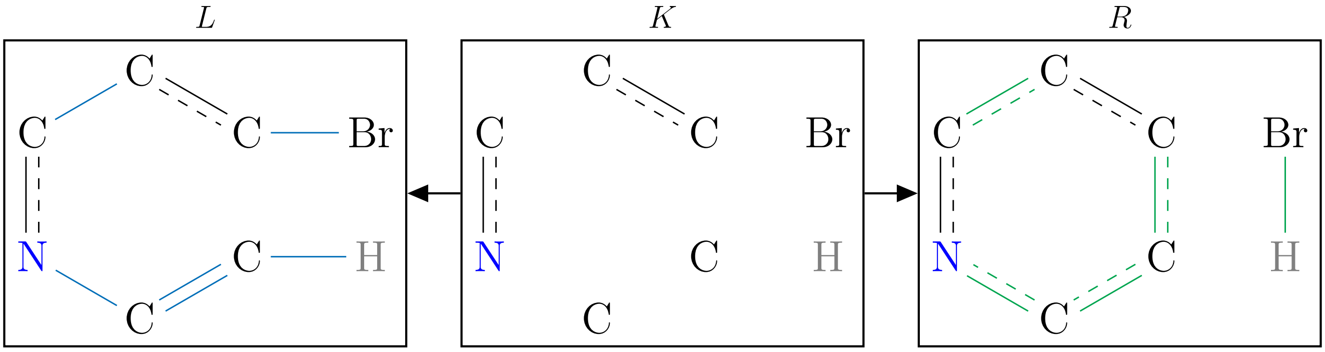

Files: out/1418_r_283_11300100_{L, K, R}

0.0.285 284

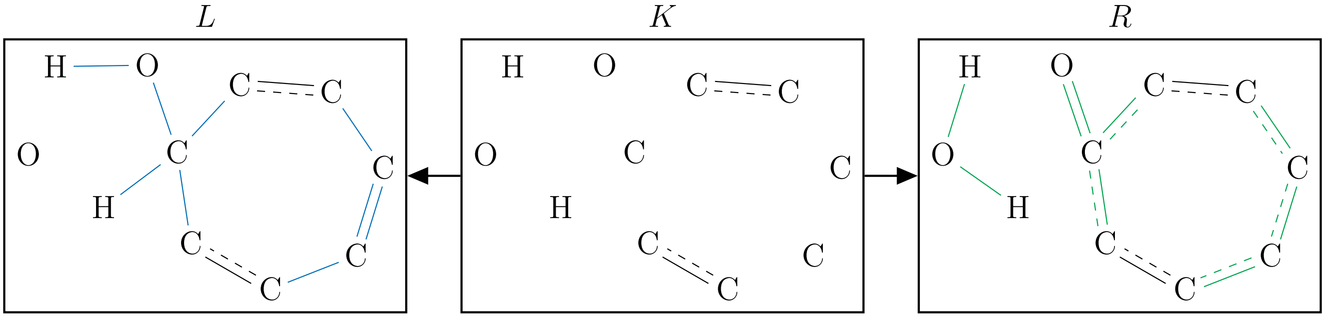

Files: out/1421_r_284_10300000_{L, K, R}

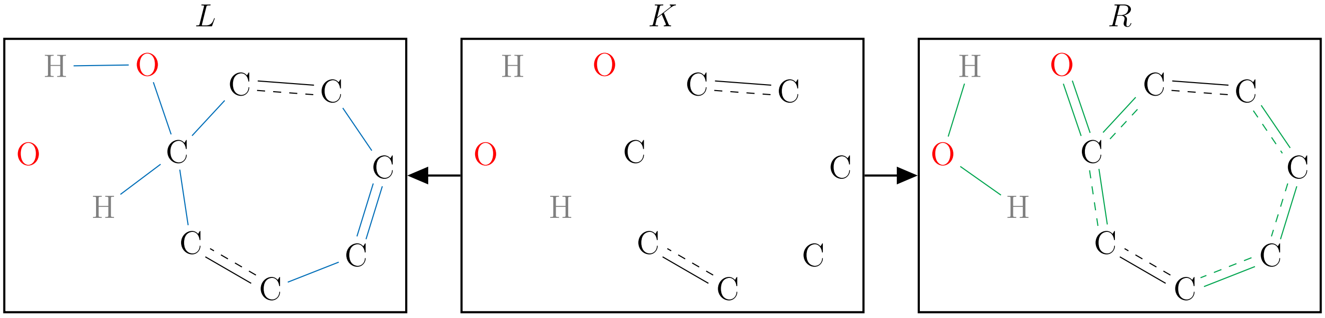

Files: out/1423_r_284_11300100_{L, K, R}

0.0.286 285

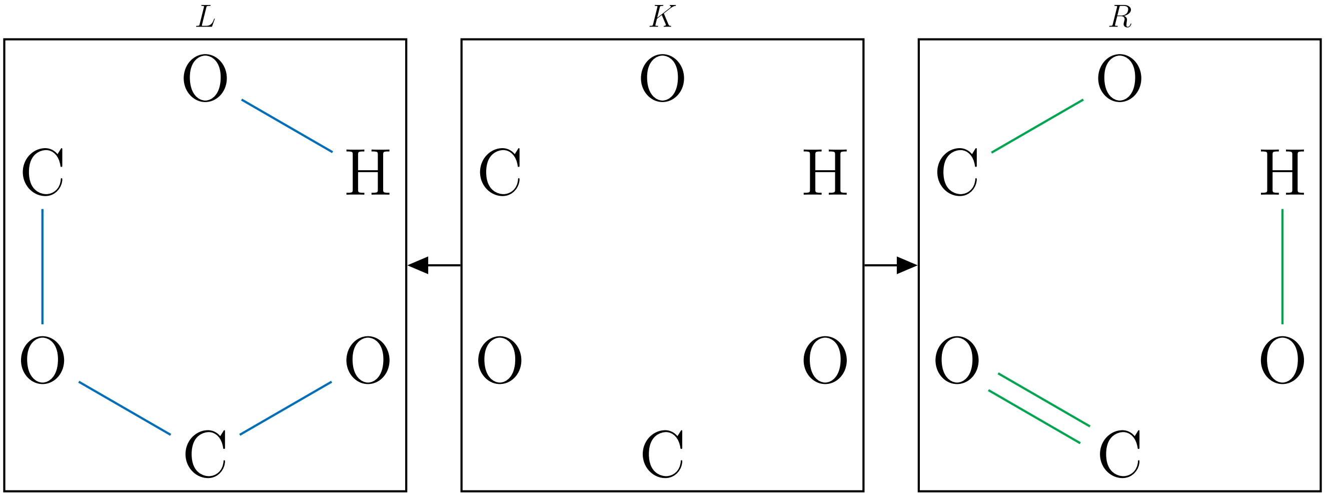

Files: out/1426_r_285_10300000_{L, K, R}

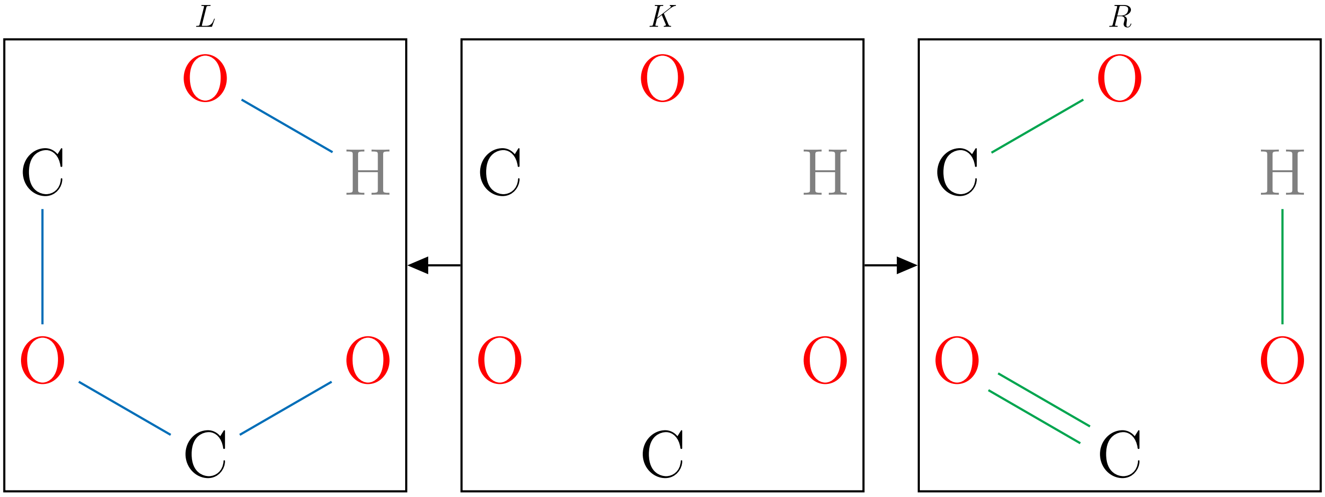

Files: out/1428_r_285_11300100_{L, K, R}

0.0.287 286

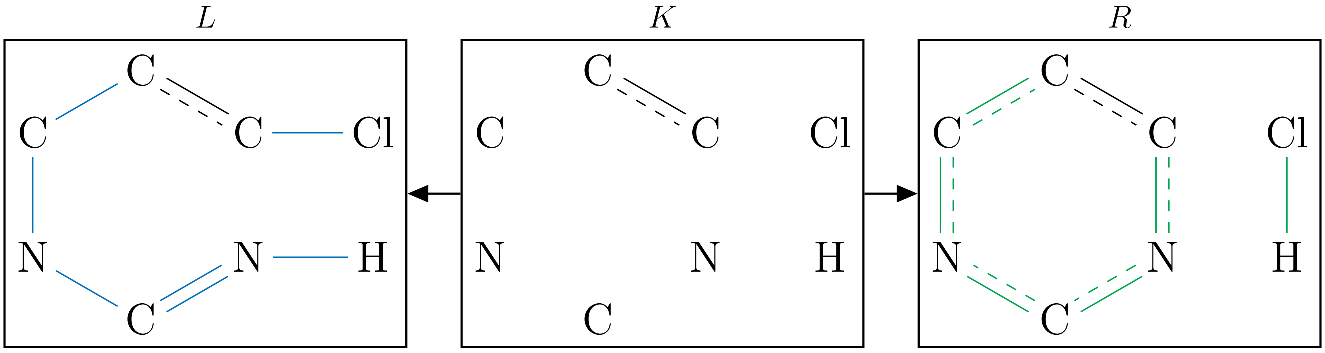

Files: out/1431_r_286_10300000_{L, K, R}

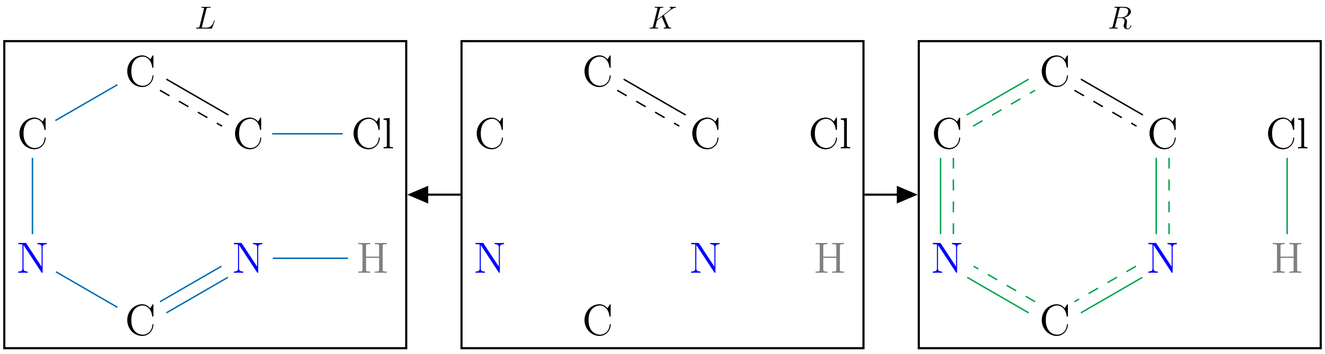

Files: out/1433_r_286_11300100_{L, K, R}

0.0.288 287

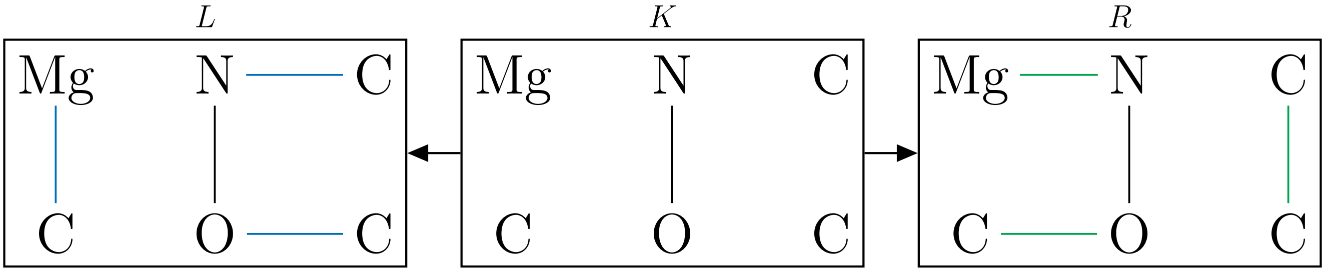

Files: out/1436_r_287_10300000_{L, K, R}

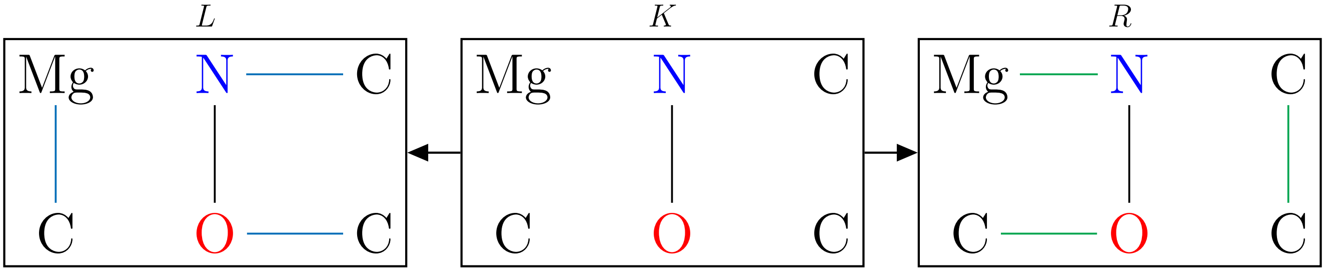

Files: out/1438_r_287_11300100_{L, K, R}

0.0.289 288

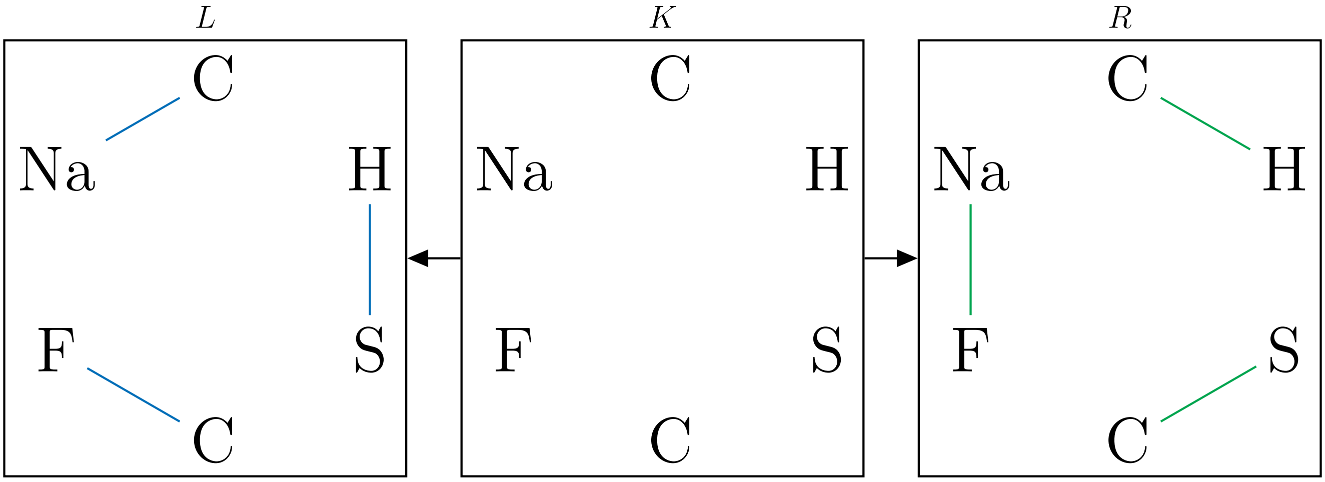

Files: out/1441_r_288_10300000_{L, K, R}

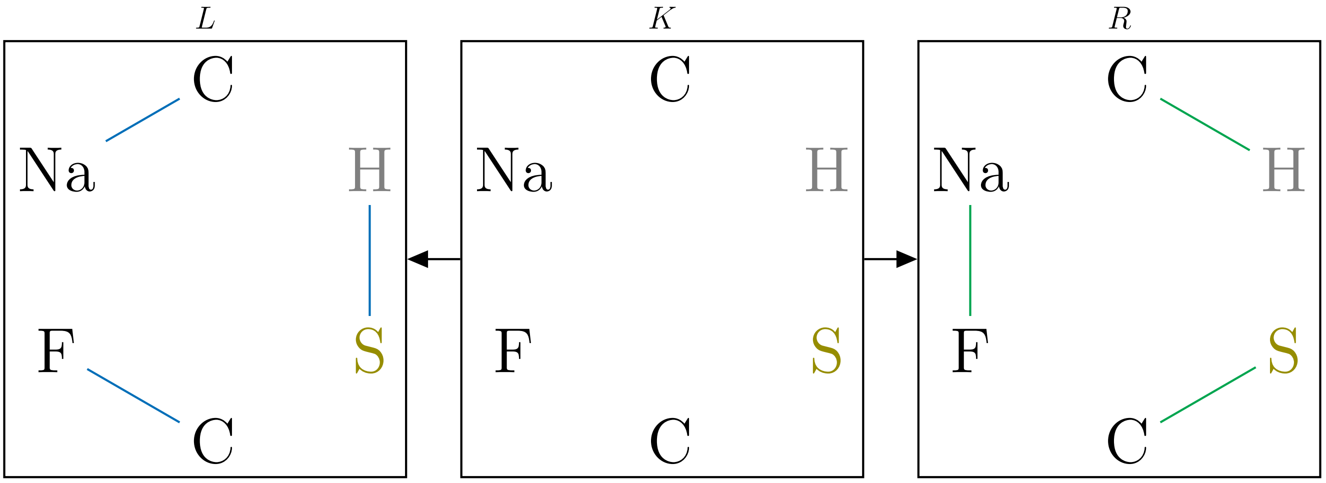

Files: out/1443_r_288_11300100_{L, K, R}

0.0.290 289

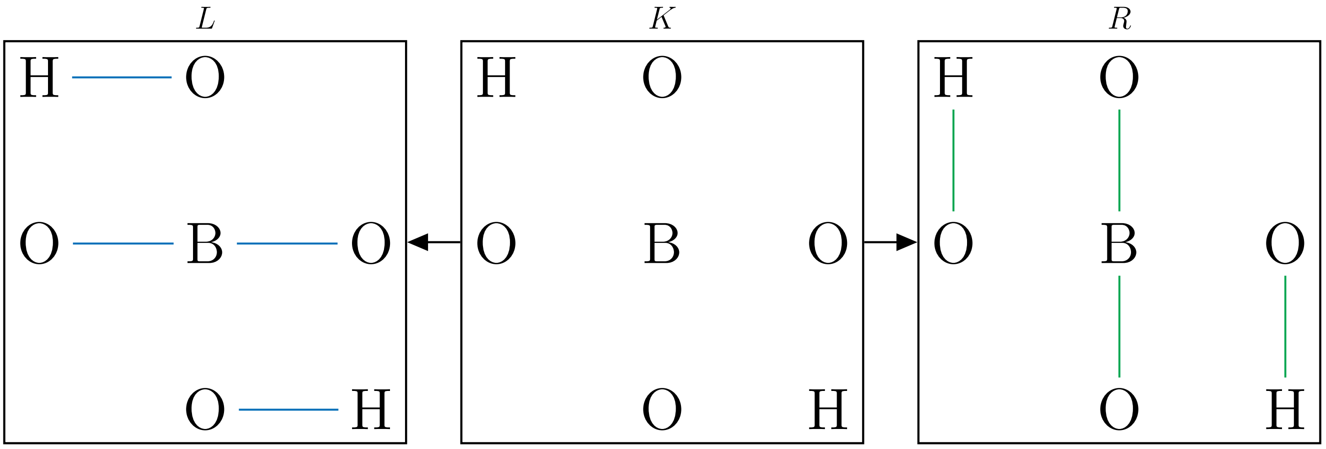

Files: out/1446_r_289_10300000_{L, K, R}

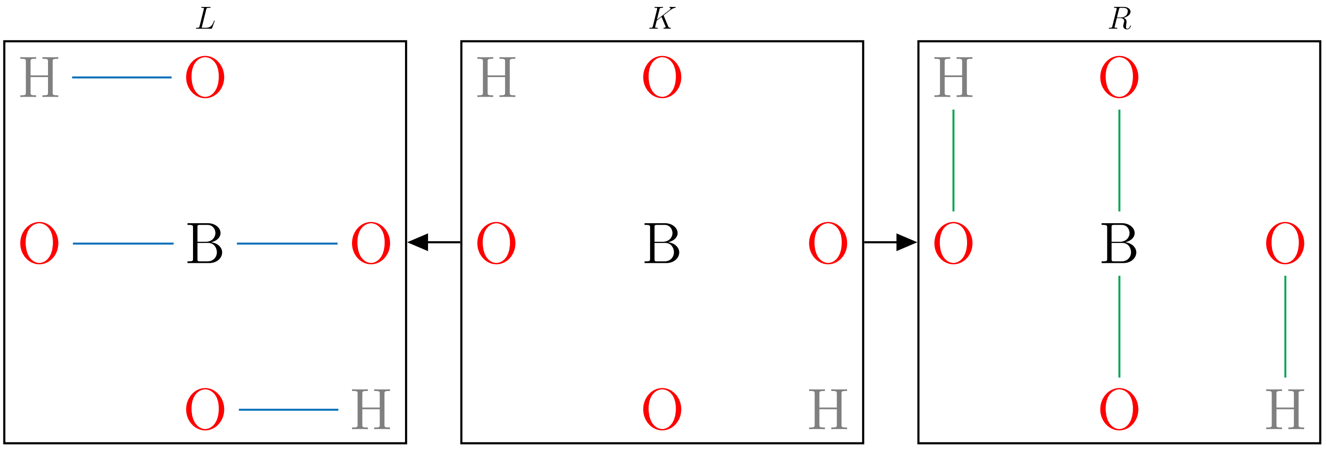

Files: out/1448_r_289_11300100_{L, K, R}

0.0.291 290

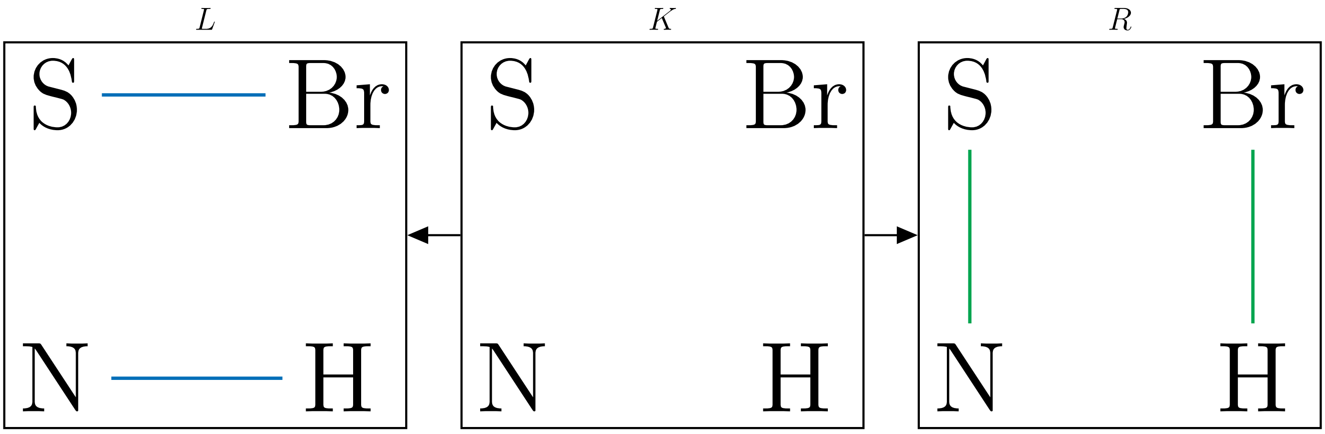

Files: out/1451_r_290_10300000_{L, K, R}

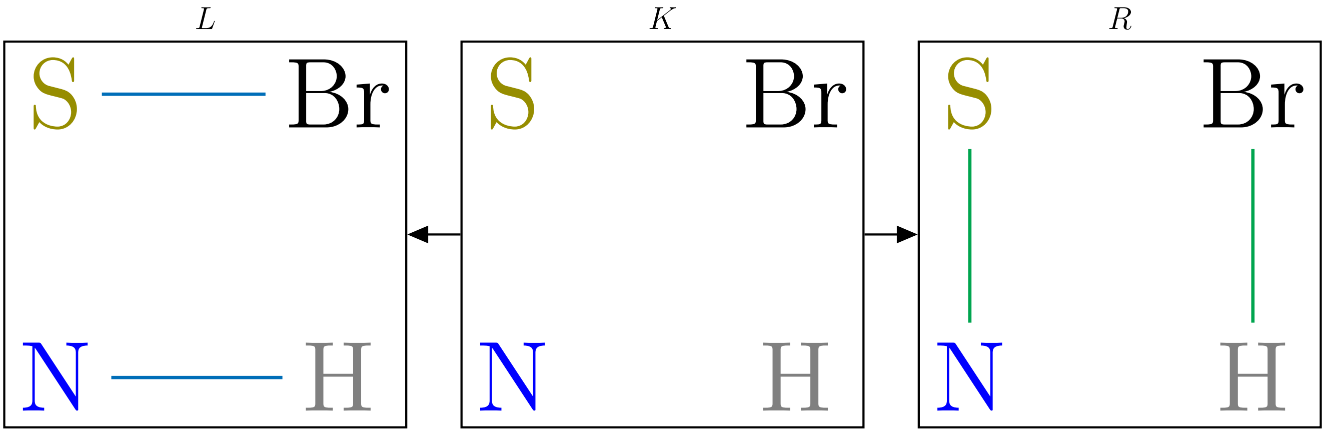

Files: out/1453_r_290_11300100_{L, K, R}

0.0.292 291

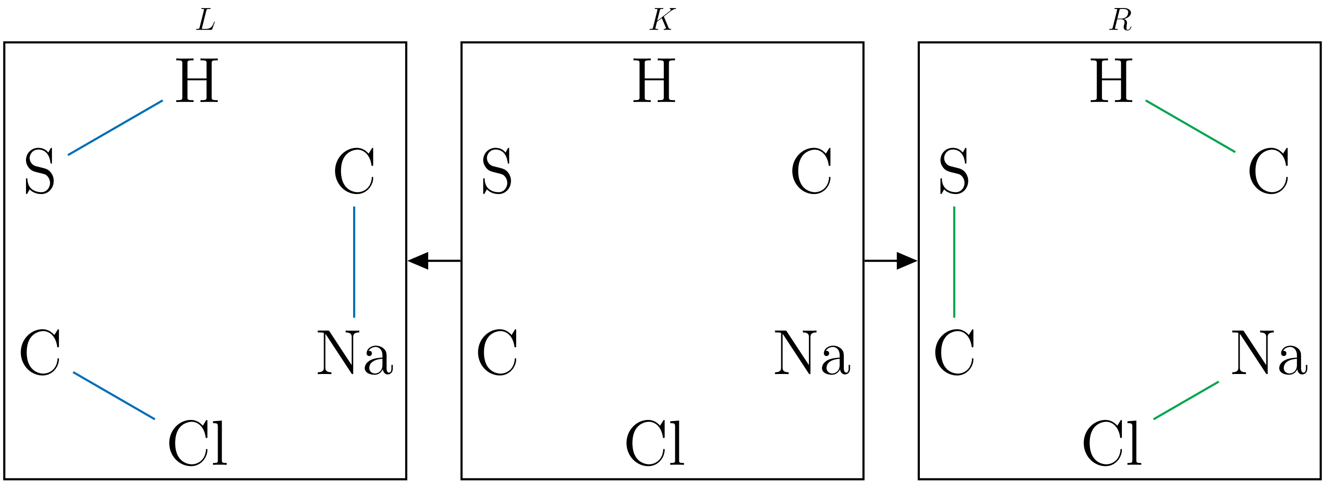

Files: out/1456_r_291_10300000_{L, K, R}

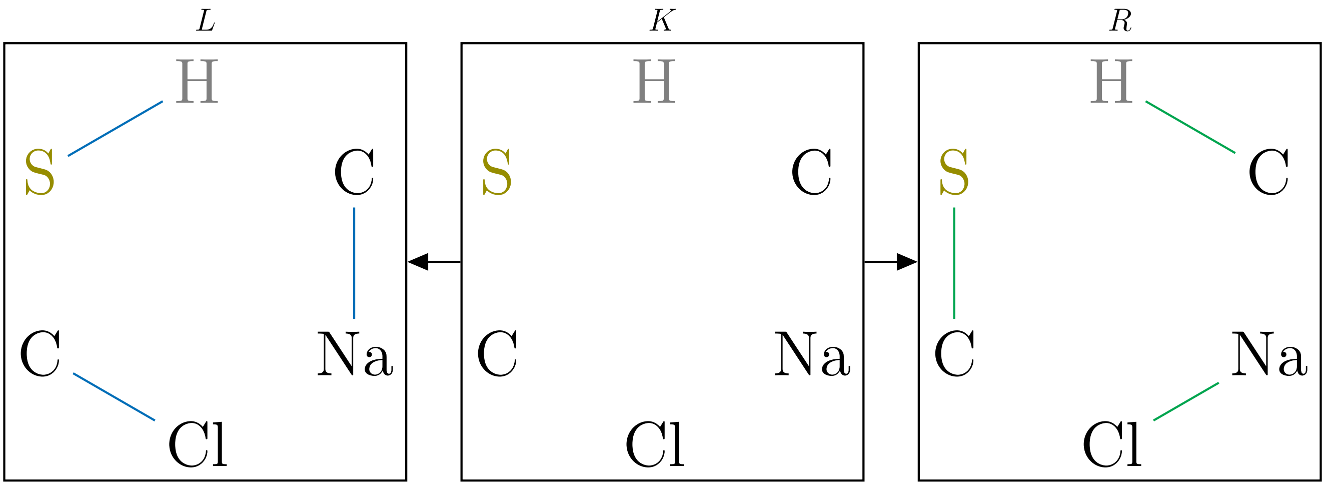

Files: out/1458_r_291_11300100_{L, K, R}

0.0.293 292

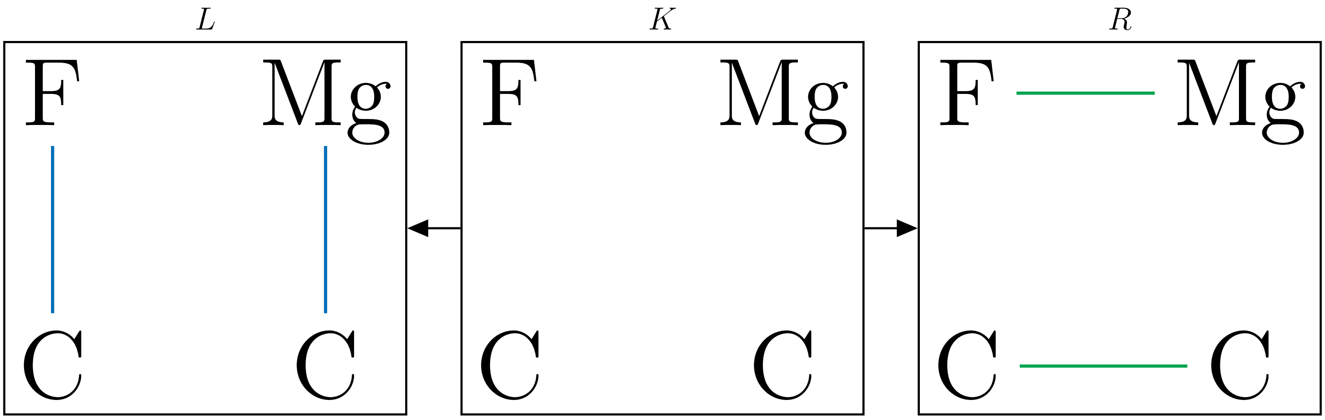

Files: out/1461_r_292_10300000_{L, K, R}

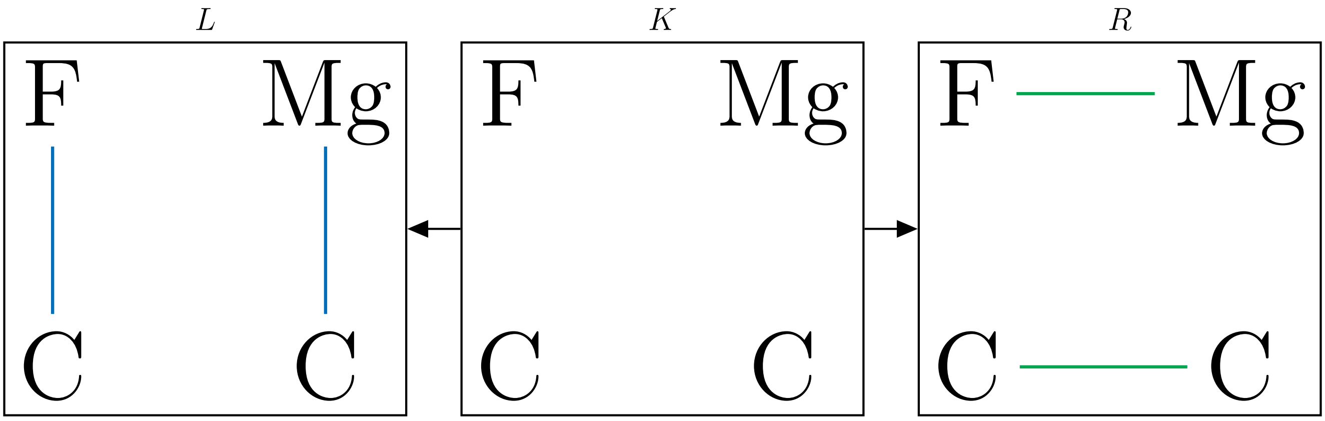

Files: out/1463_r_292_11300100_{L, K, R}

0.0.294 293

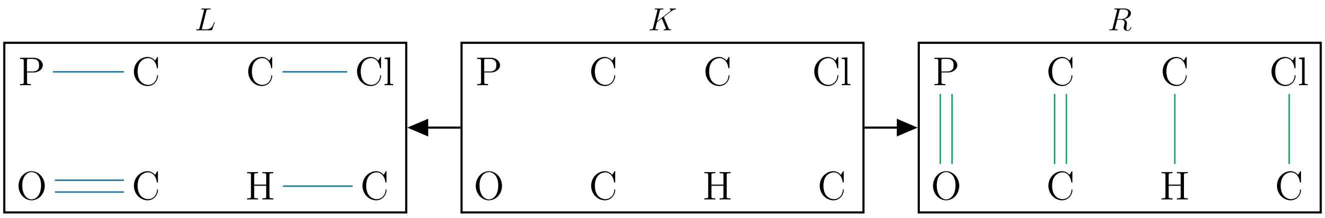

Files: out/1466_r_293_10300000_{L, K, R}

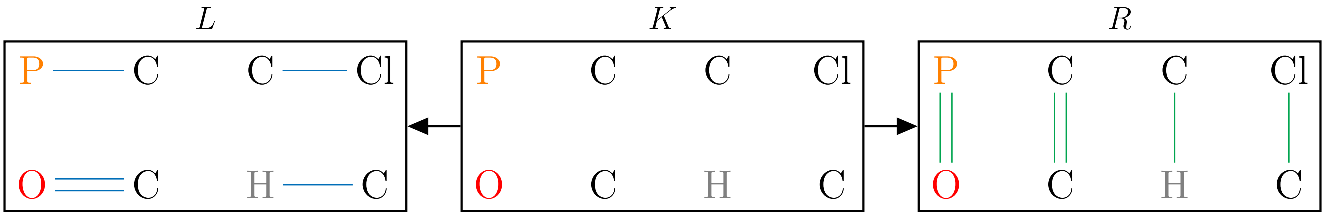

Files: out/1468_r_293_11300100_{L, K, R}

0.0.295 294

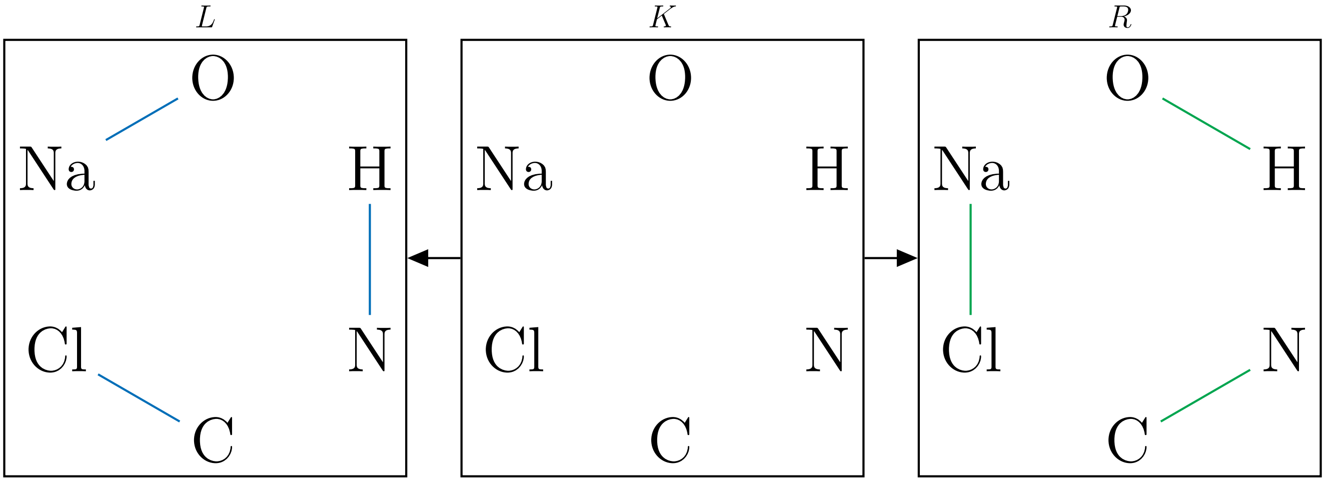

Files: out/1471_r_294_10300000_{L, K, R}

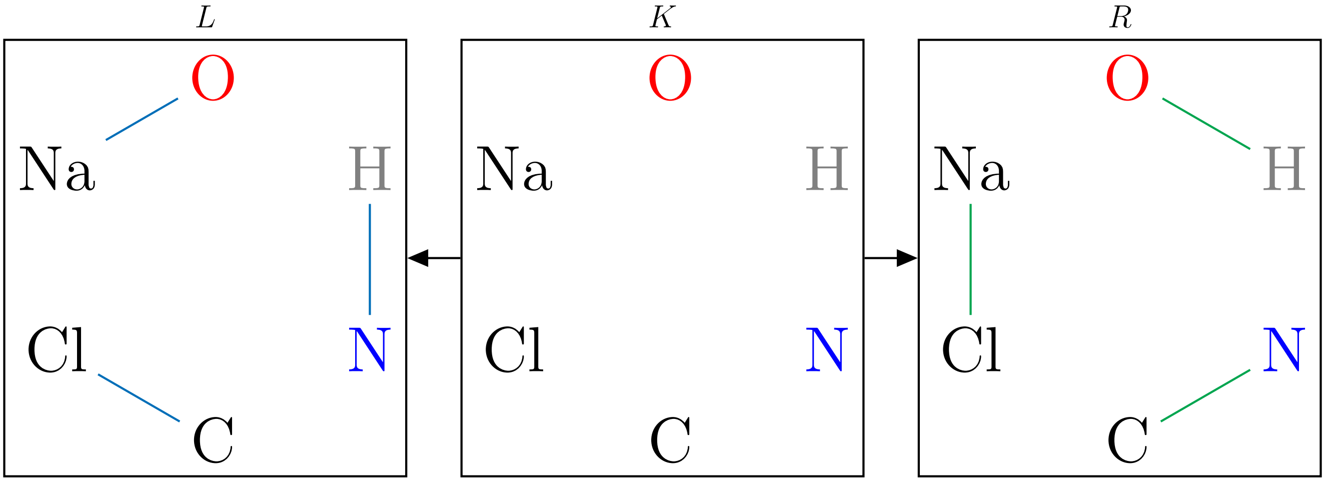

Files: out/1473_r_294_11300100_{L, K, R}

0.0.296 295

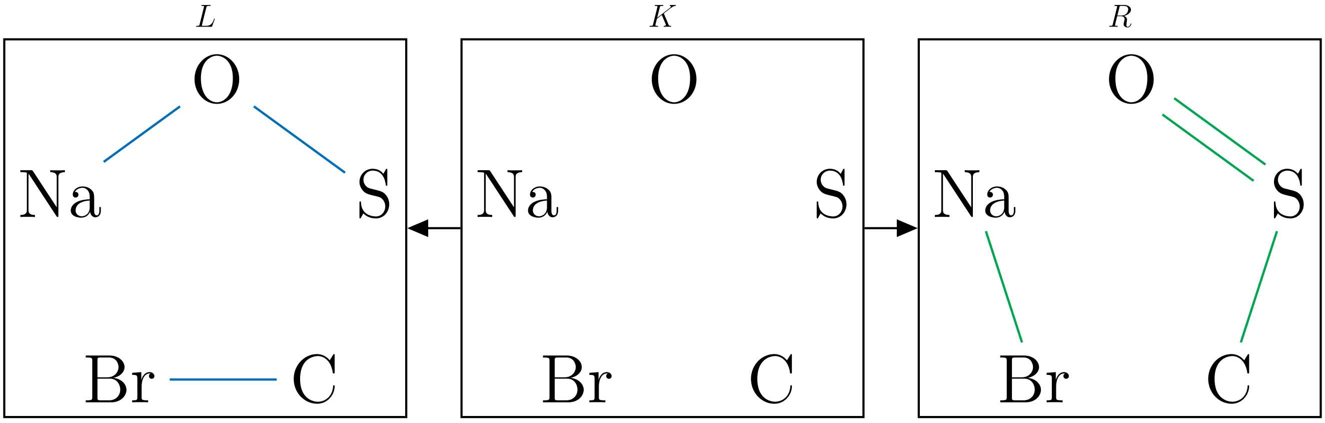

Files: out/1476_r_295_10300000_{L, K, R}

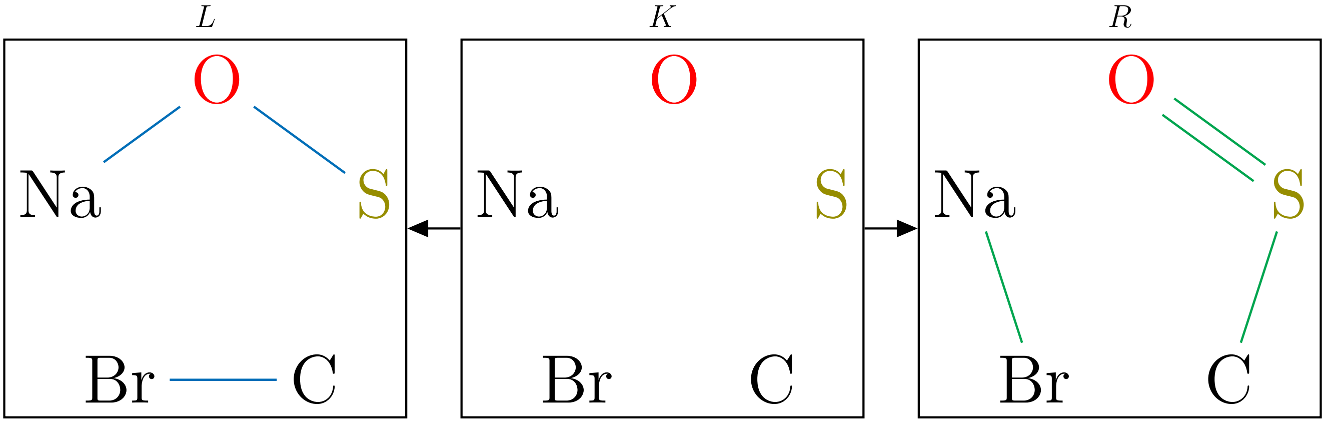

Files: out/1478_r_295_11300100_{L, K, R}

0.0.297 296

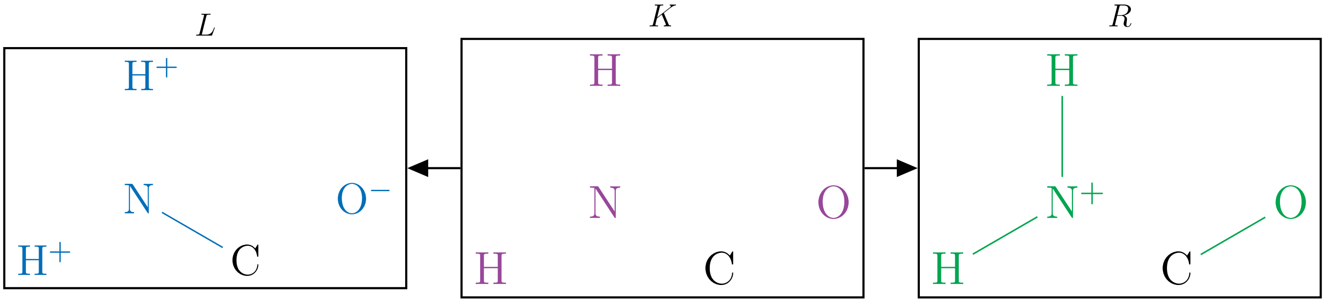

Files: out/1481_r_296_10300000_{L, K, R}

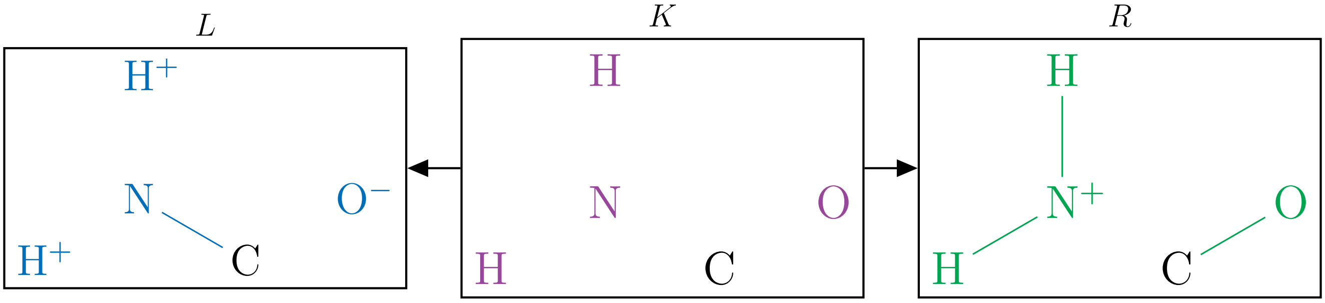

Files: out/1483_r_296_11300100_{L, K, R}

0.0.298 297

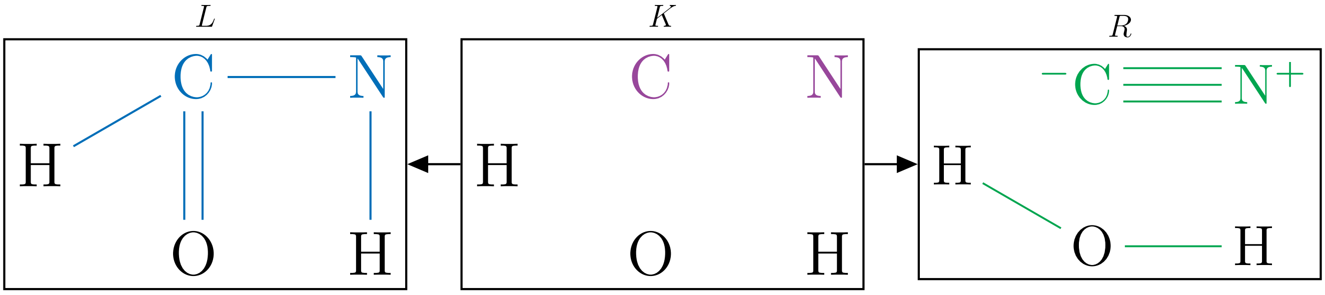

Files: out/1486_r_297_10300000_{L, K, R}

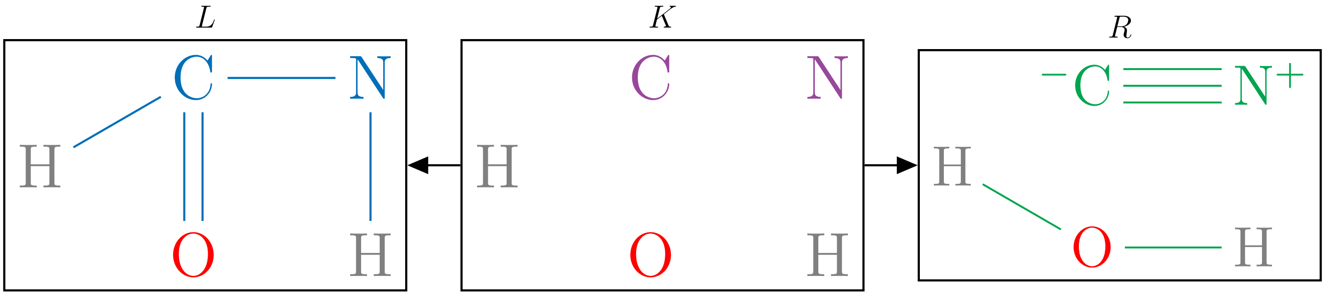

Files: out/1488_r_297_11300100_{L, K, R}

0.0.299 298

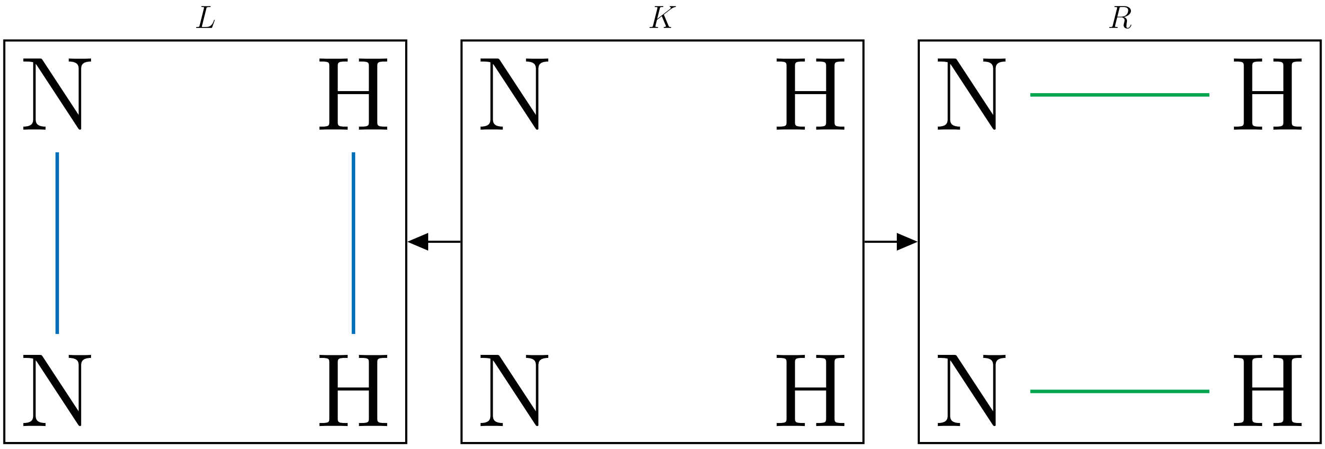

Files: out/1491_r_298_10300000_{L, K, R}

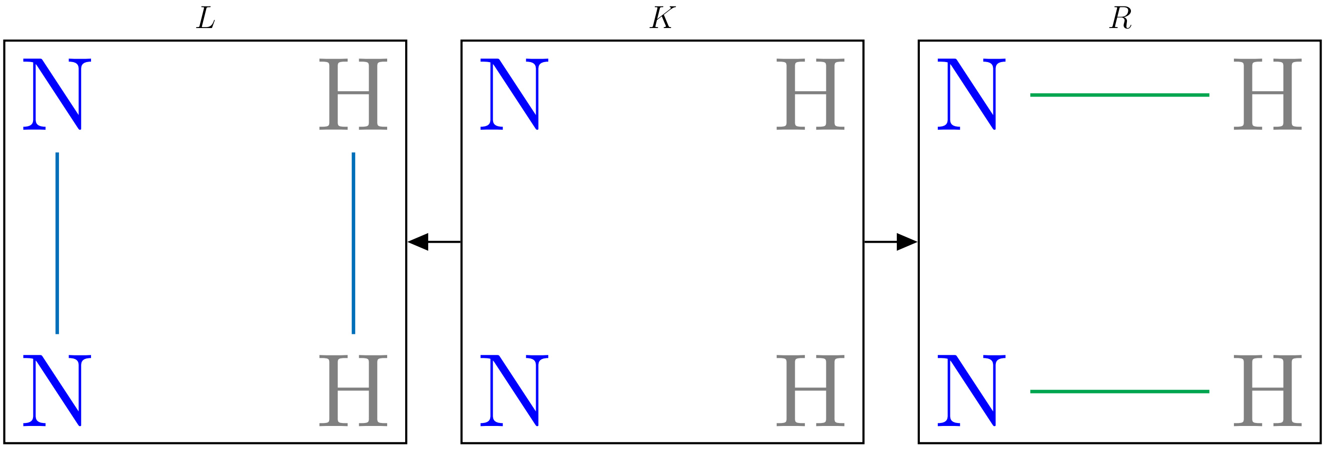

Files: out/1493_r_298_11300100_{L, K, R}

0.0.300 299

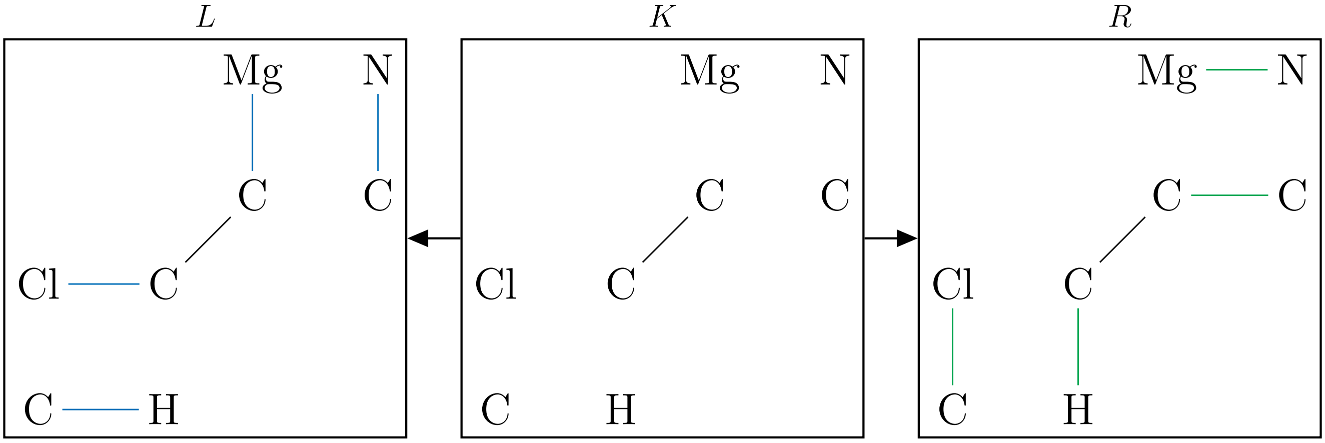

Files: out/1496_r_299_10300000_{L, K, R}

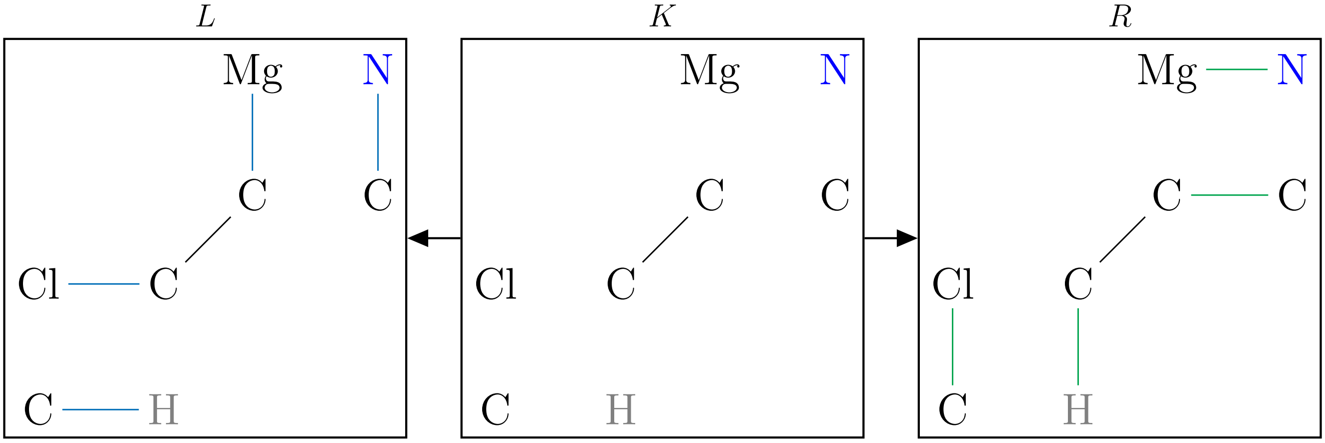

Files: out/1498_r_299_11300100_{L, K, R}

0.0.301 300

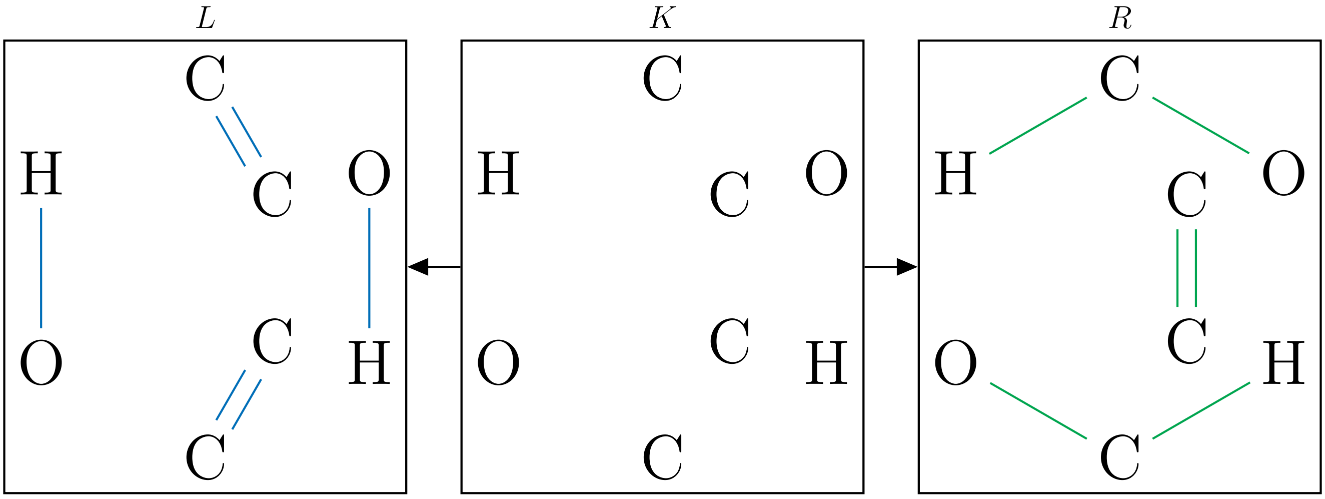

Files: out/1501_r_300_10300000_{L, K, R}

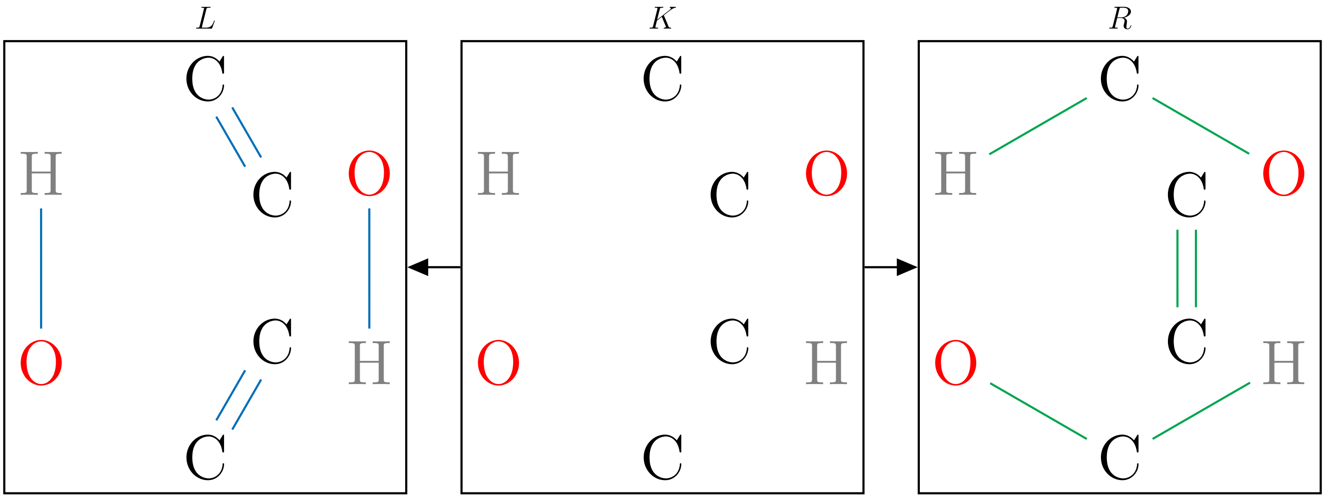

Files: out/1503_r_300_11300100_{L, K, R}

0.0.302 301

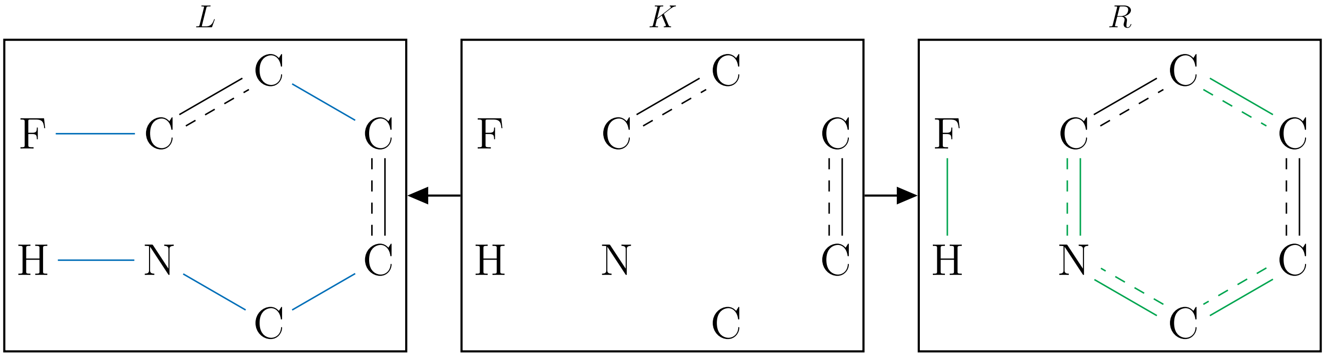

Files: out/1506_r_301_10300000_{L, K, R}

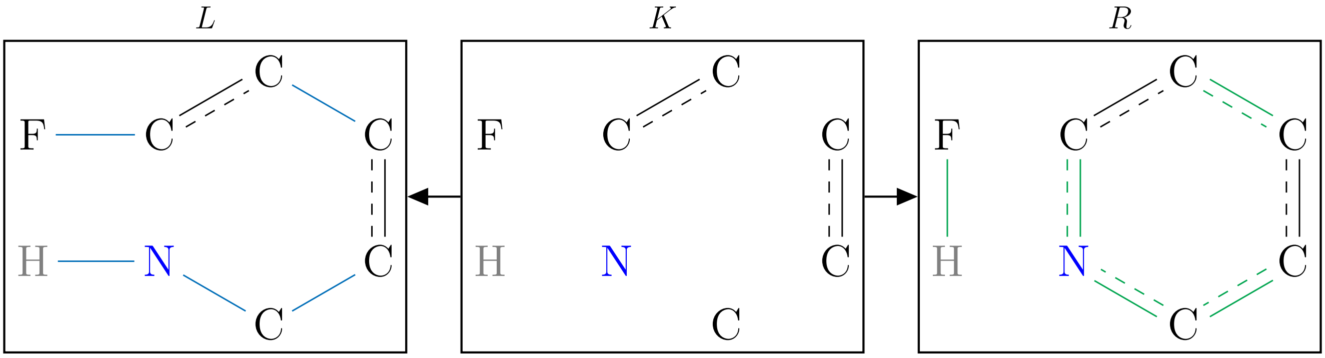

Files: out/1508_r_301_11300100_{L, K, R}

0.0.303 302

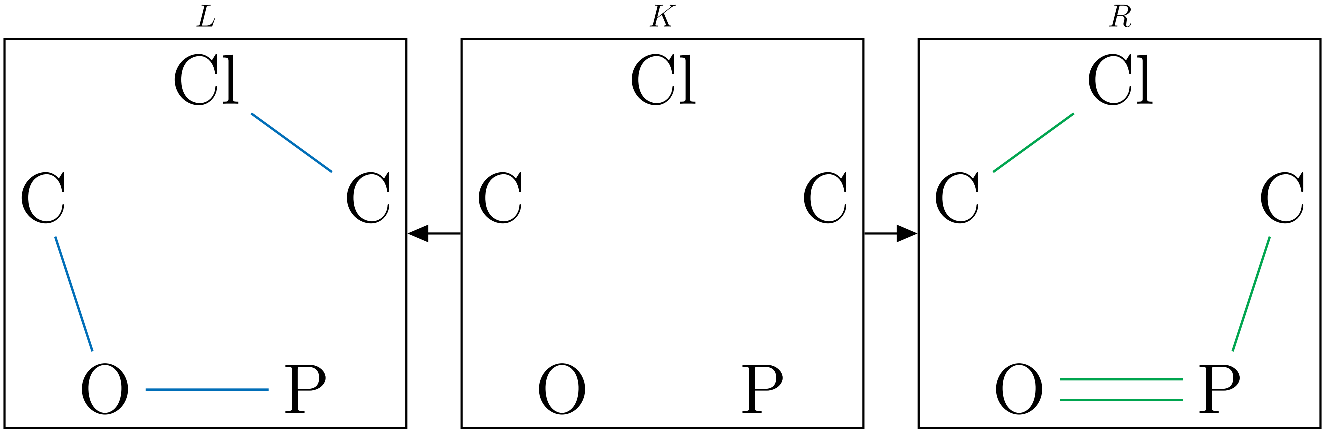

Files: out/1511_r_302_10300000_{L, K, R}

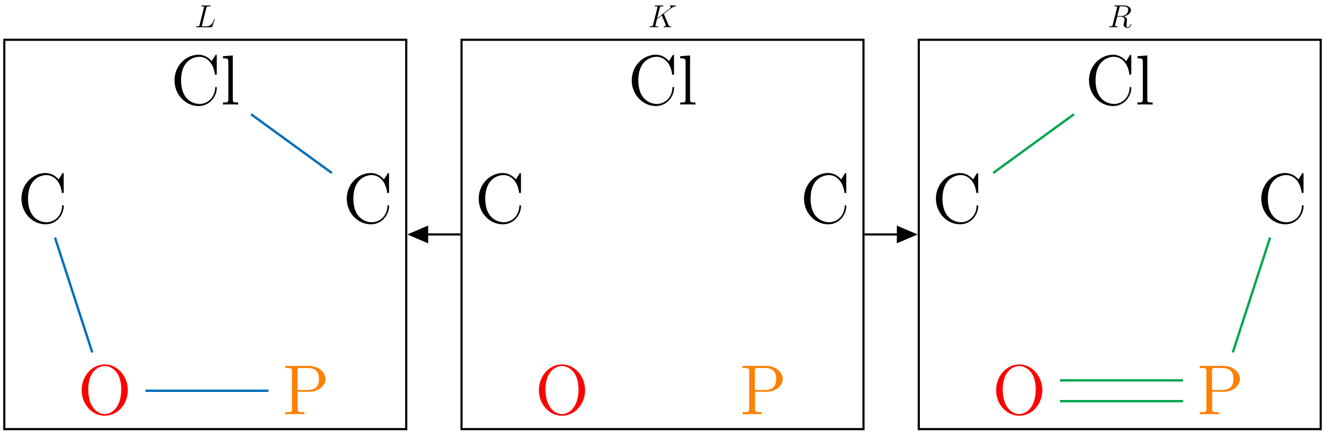

Files: out/1513_r_302_11300100_{L, K, R}

0.0.304 303

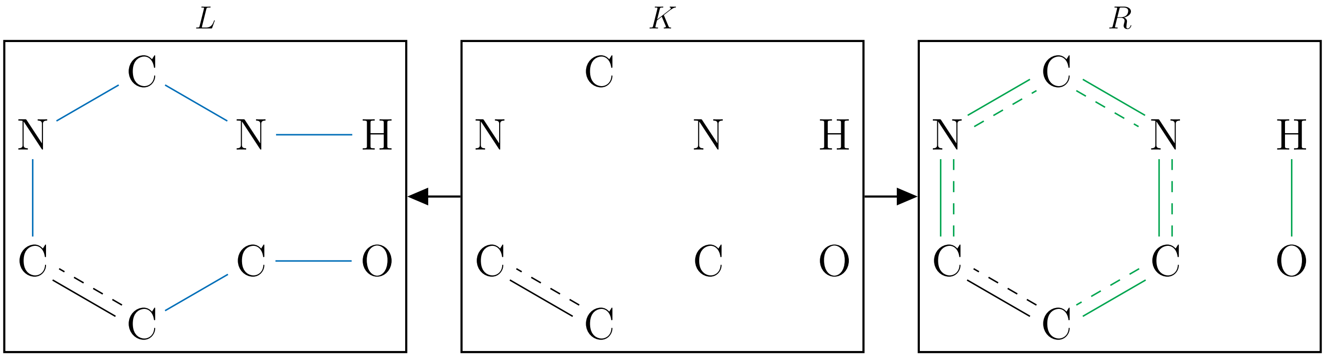

Files: out/1516_r_303_10300000_{L, K, R}

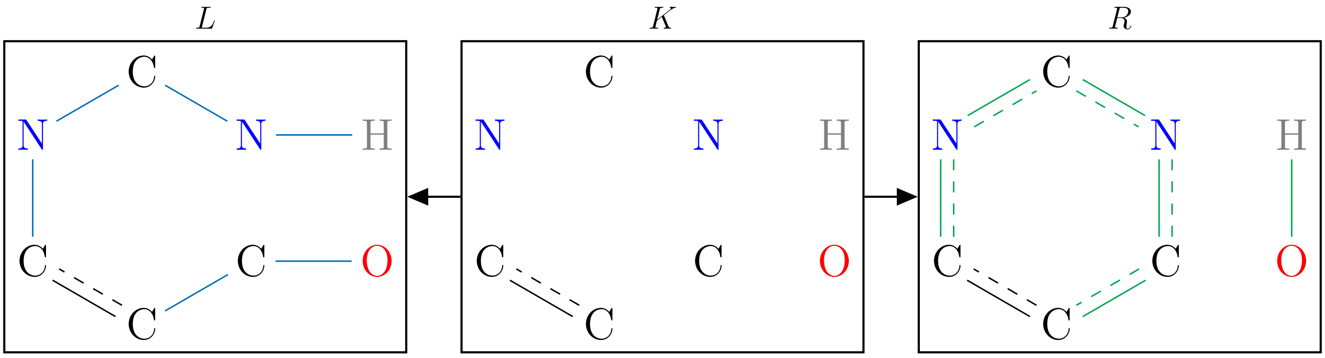

Files: out/1518_r_303_11300100_{L, K, R}

0.0.305 304

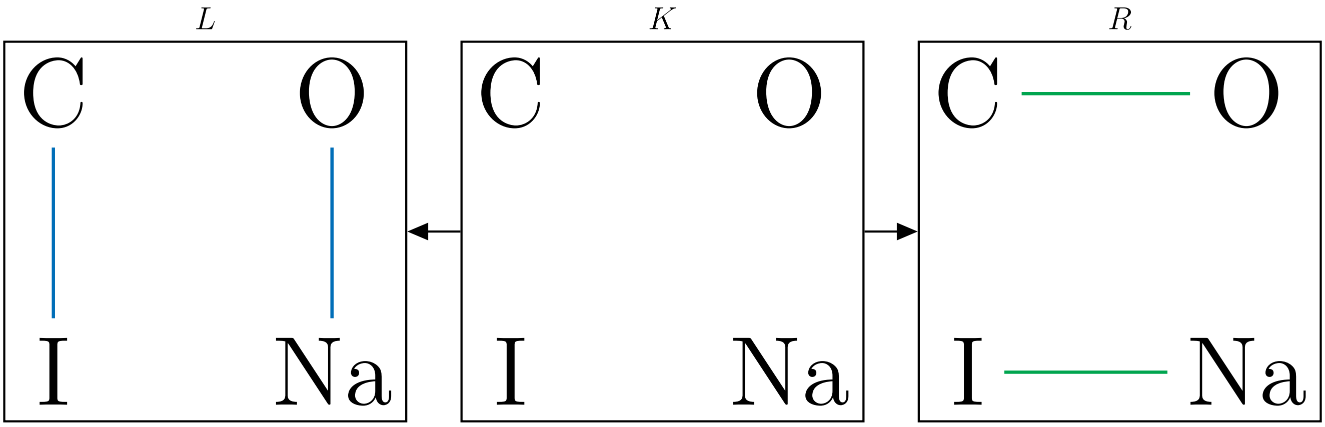

Files: out/1521_r_304_10300000_{L, K, R}

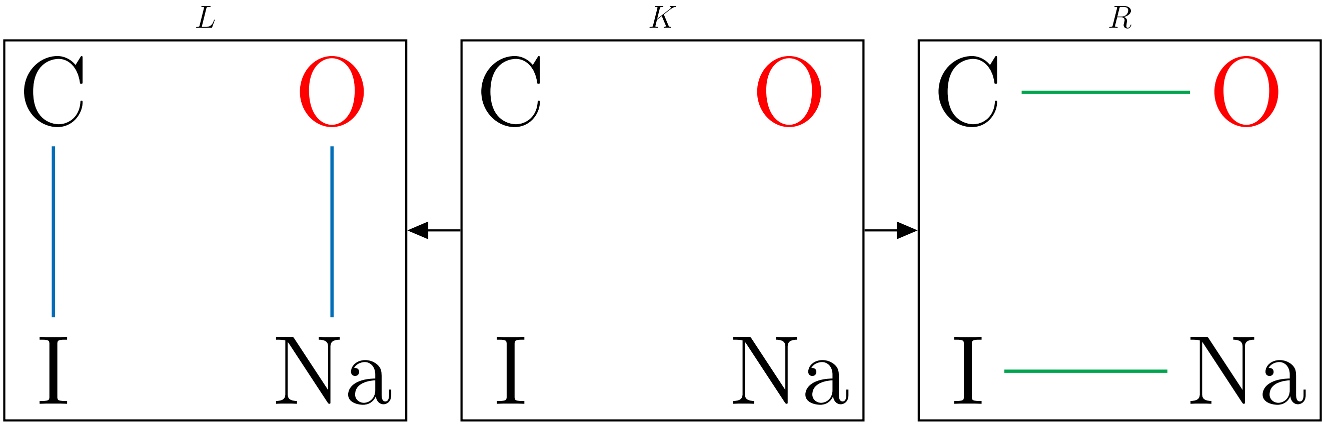

Files: out/1523_r_304_11300100_{L, K, R}

0.0.306 305

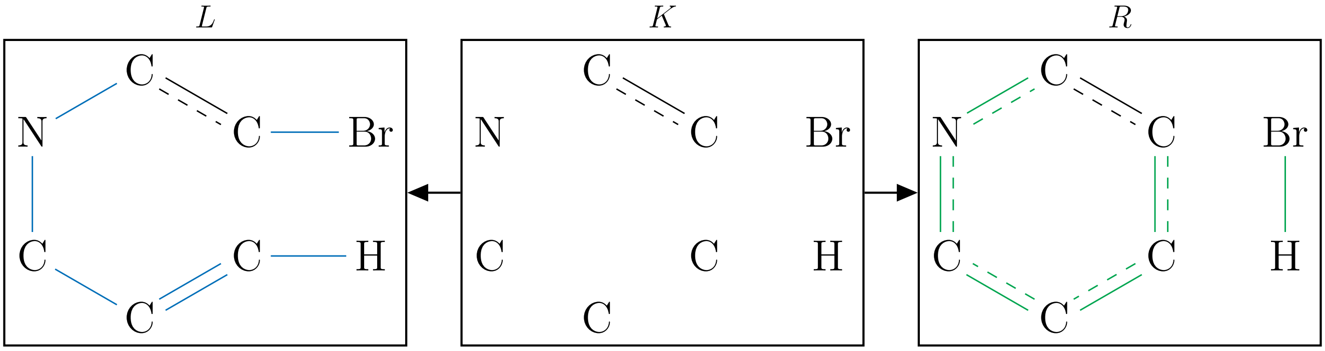

Files: out/1526_r_305_10300000_{L, K, R}

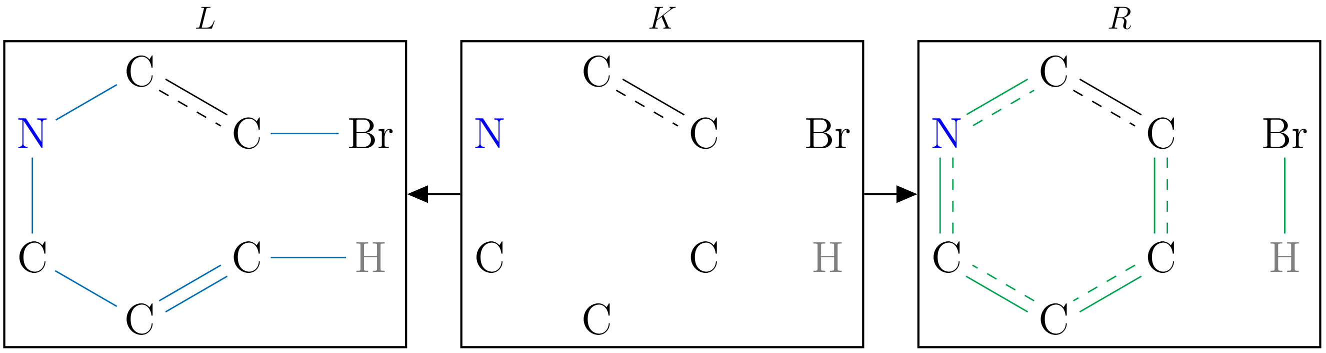

Files: out/1528_r_305_11300100_{L, K, R}

0.0.307 306

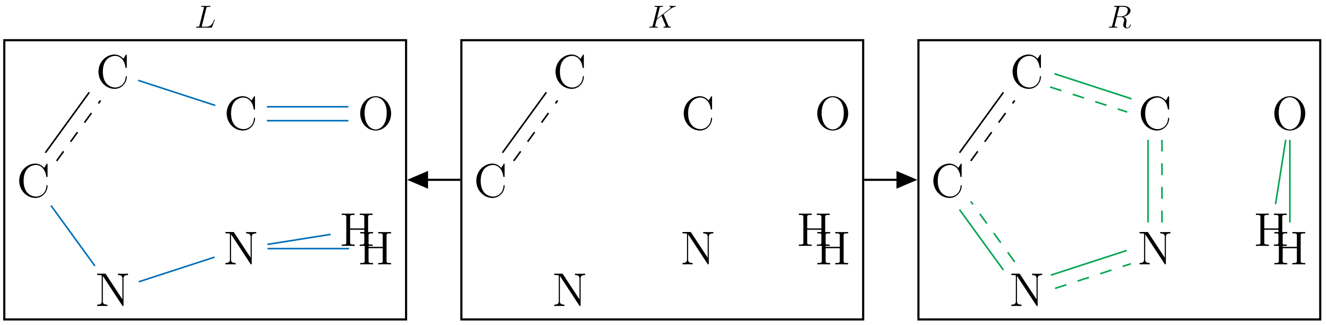

Files: out/1531_r_306_10300000_{L, K, R}

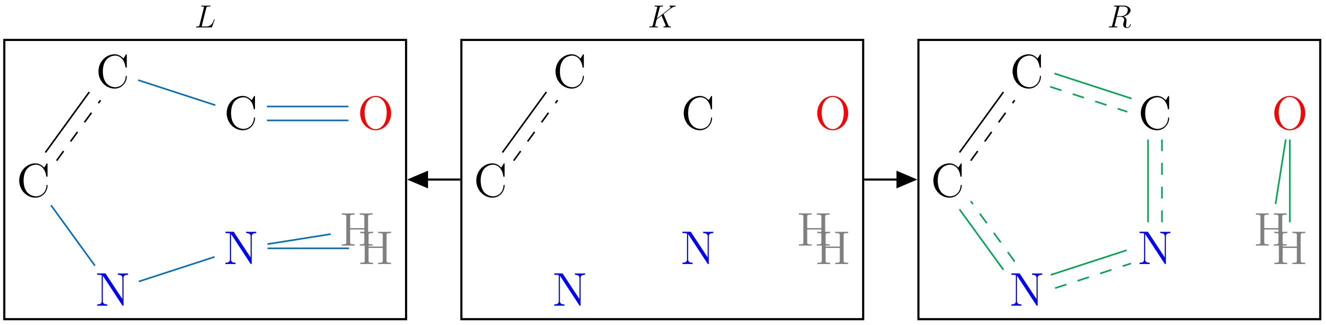

Files: out/1533_r_306_11300100_{L, K, R}

0.0.308 307

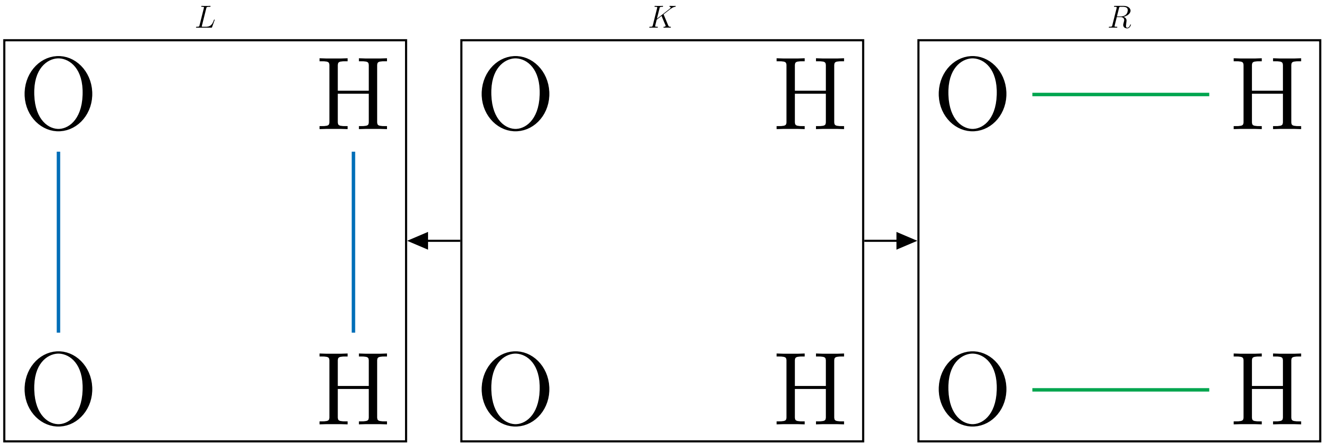

Files: out/1536_r_307_10300000_{L, K, R}

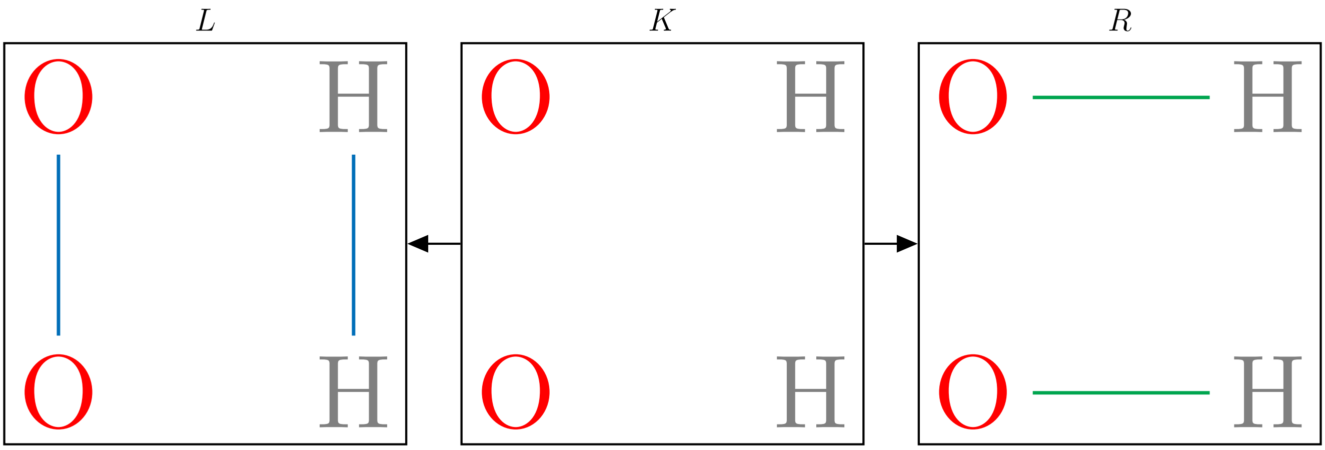

Files: out/1538_r_307_11300100_{L, K, R}

0.0.309 308

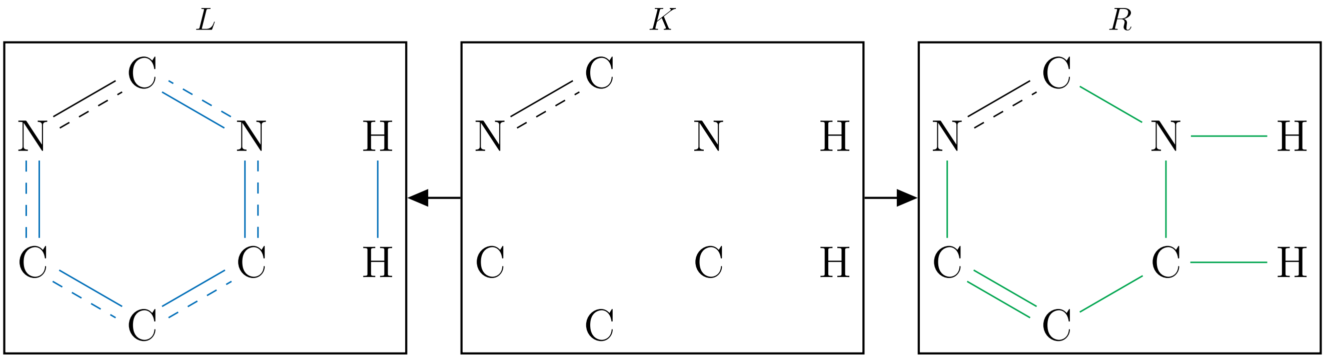

Files: out/1541_r_308_10300000_{L, K, R}

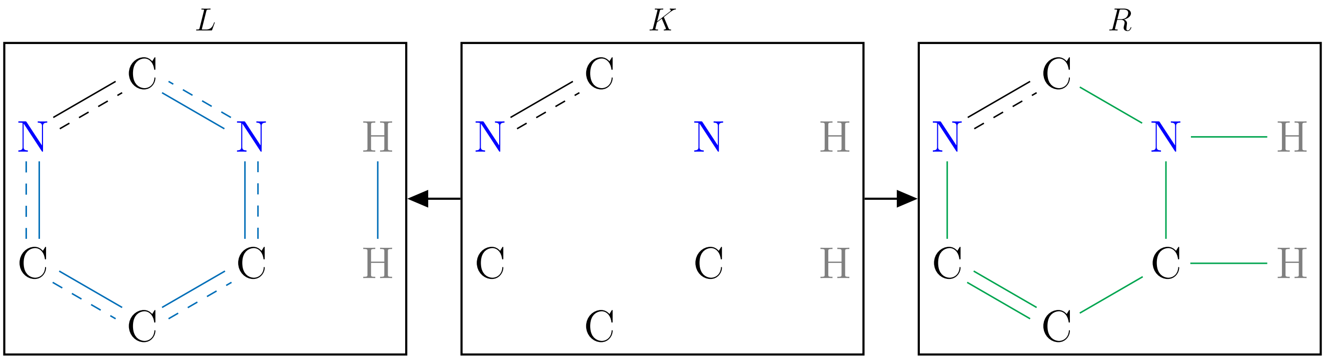

Files: out/1543_r_308_11300100_{L, K, R}

0.0.310 309

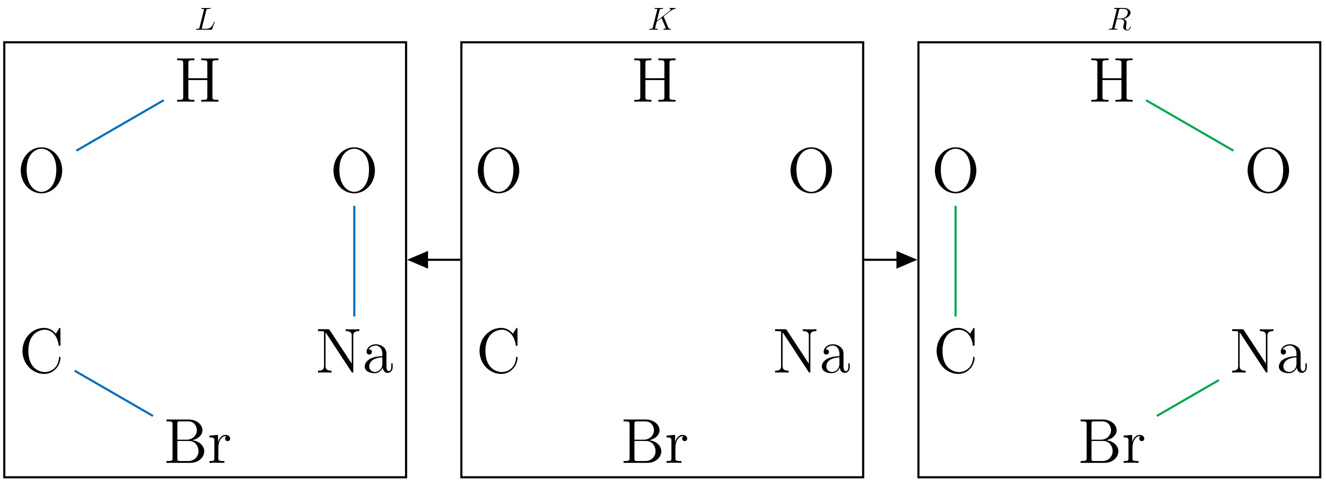

Files: out/1546_r_309_10300000_{L, K, R}

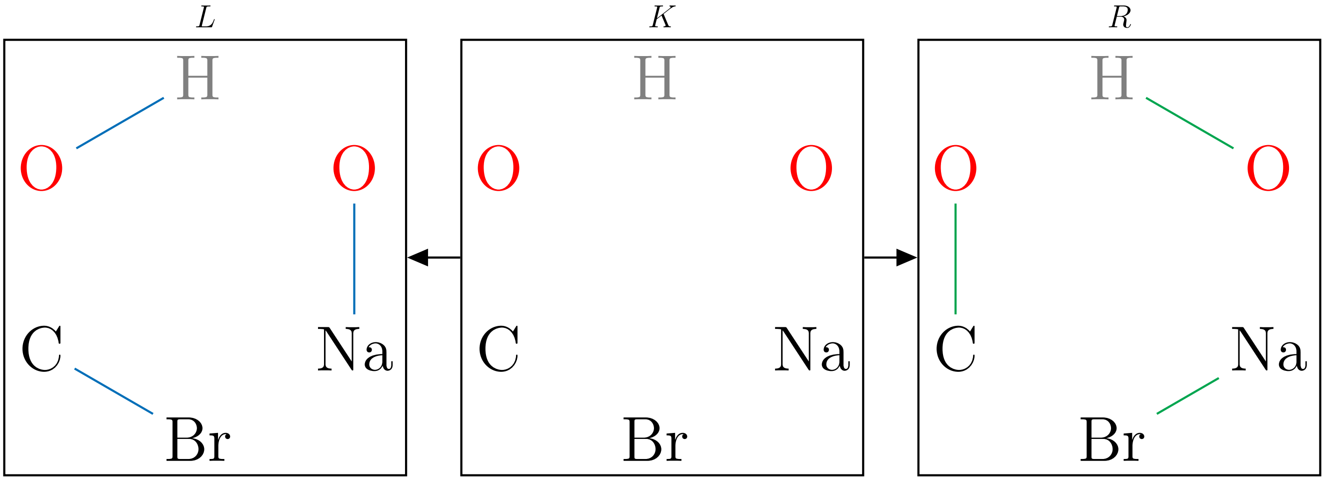

Files: out/1548_r_309_11300100_{L, K, R}

0.0.311 310

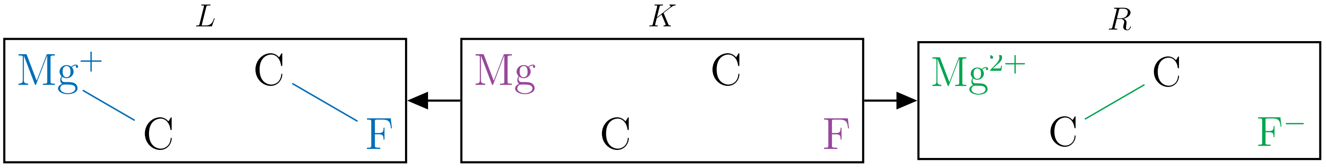

Files: out/1551_r_310_10300000_{L, K, R}

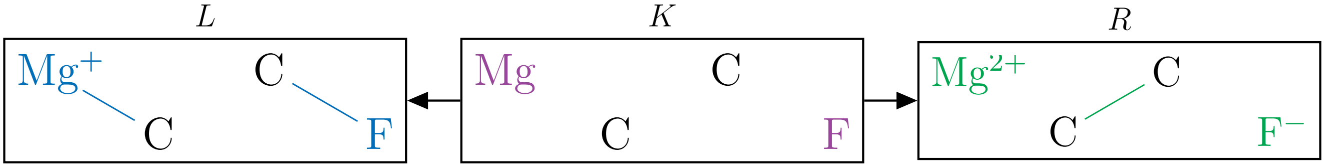

Files: out/1553_r_310_11300100_{L, K, R}
